# Pan-cancer transcriptomic analysis dissects immune and proliferative functions of APOBEC3 cytidine deaminases

**DOI:** 10.1093/nar/gky1316

**Published:** 2019-01-09

**Authors:** Joseph C F Ng, Jelmar Quist, Anita Grigoriadis, Michael H Malim, Franca Fraternali

**Affiliations:** 1 Randall Centre for Cell and Molecular Biophysics, King's College London, London, UK; 2 Cancer Bioinformatics, School of Cancer and Pharmaceutical Sciences, CRUK King's Health Partners Centre, Breast Cancer Now Research Unit, King's College London, London, UK; 3 Department of Infectious Diseases, School of Immunology and Microbial Sciences, King's College London, London, UK

## Abstract

APOBEC3 cytidine deaminases are largely known for their innate immune protection from viral infections. Recently, members of the family have been associated with a distinct mutational activity in some cancer types. We report a pan-tissue, pan-cancer analysis of RNA-seq data specific to the APOBEC3 genes in 8,951 tumours, 786 cancer cell lines and 6,119 normal tissues. By deconvolution of levels of different cell types in tumour admixtures, we demonstrate that *APOBEC3B* (*A3B*), the primary candidate as a cancer mutagen, shows little association with immune cell types compared to its paralogues. We present a pipeline called RESPECTEx (REconstituting SPecific Cell-Type Expression) and use it to deconvolute cell-type specific expression levels in a given cohort of tumour samples. We functionally annotate APOBEC3 co-expressing genes, and create an interactive visualization tool which ‘barcodes’ the functional enrichment (http://fraternalilab.kcl.ac.uk/apobec-barcodes/). These analyses reveal that *A3B* expression correlates with cell cycle and DNA repair genes, whereas the other APOBEC3 members display specificity for immune processes and immune cell populations. We offer molecular insights into the functions of individual APOBEC3 proteins in antiviral and proliferative contexts, and demonstrate the diversification this family of enzymes displays at the transcriptomic level, despite their high similarity in protein sequences and structures.

## INTRODUCTION

Human APOBEC3 (apolipoprotein B mRNA editing catalytic polypeptide-like 3) proteins are a family of seven cytidine deaminases capable of causing cytidine-to-uridine (C>U) mutations on single-stranded DNA molecules. Though described as restriction factors that impede replication of many viruses such as HIV-1 (human immunodeficiency virus-1) (1, 2), this family of enzymes has also been associated with a distinct mutational signature in the genomes of many cancers, particularly those which localize to the breast, lung, bladder, cervix and head and neck, amongst other organs (3–5). APOBEC3-signature mutations have been thought to contribute to subclonal diversity in tumours (6), thereby potentially promoting drug resistance (7–9). *In vitro* work has demonstrated that overexpression of the *APOBEC3B* (*A3B*) gene results in extensive C>T mutagenesis and an increase in genomic uracil level (10). *A3B* overexpression has been documented in breast cancer cell lines and many other tumours, and shows a weak correlation with the level of APOBEC3-signature mutations (5, 10). However, little has been done to unravel the biological basis of APOBEC3 activation *in vivo*, the regulation of their expression and functions of the different family members, or the mechanisms under which the enzyme interacts with and mutates human genomic DNA. Multiple APOBEC3 proteins, including APOBEC3A (A3A) (11) and haplotype I of APOBEC3H (A3H) (12), have been implicated as the genomic mutators in cancer, alongside A3B. Human APOBEC3 proteins are remarkably similar to each other, as their pairwise sequence identity can exceed 80% (13). Moreover, all of them induce mutations on retroviral genomes, and all (except APOBEC3G, or A3G) deaminate the same single-stranded DNA (ssDNA) substrate (5’-TC, where C denotes the deaminated cytosine) (14–17). Therefore, the analysis of DNA sequencing data alone is inadequate to pinpoint the exact APOBEC3 member(s) responsible for generating these somatic mutations.

Here, we look at other dimensions of molecular data from cancer samples that could offer insights on the involvement of different APOBEC3 members in creating these mutational signatures. One such example is gene expression: this has been studied by quantitative reverse-transcription polymerase chain reaction (qRT-PCR) in different organs (18) and, particularly, among haematopoietic cell subsets (19). The ever-growing repository of RNA sequencing (RNA-seq) expression data from human tumours, cancer cell lines and normal tissues enables us to study this subject in far greater depth by employing computational approaches. Transcriptome-wide profiling now enables a relatively unbiased comparison of the expression levels of the APOBEC3 genes. Moreover, it also permits analyses of their co-expression patterns with other genes, which could suggest differential involvement in biological pathways specific to each APOBEC3 gene. We analyse here gene expression patterns and the functional annotation of these co-expressing genes, revealing new insights into the involvement of different APOBEC3 family members in immune and/or tumourigenic (proliferative) processes.

We report a comprehensive pan-tissue, pan-cancer survey of RNA-seq tumour data from The Cancer Genome Atlas (TCGA), compared against data from cancer cell lines and normal tissues. The analysis defines distinct gene expression patterns of the APOBEC3 family members in cancer, and uses estimates of the proportion of tumour cells and infiltrated immune cells in tumours to interpret these differences. We have devised a bioinformatics pipeline called RESPECTEx (REconstituting SPecific Cell-Type Expression) which take estimated cell type levels in tumours further and deconvolute cell-type specific expression for a given cohort of tumours. We also analyse gene co-expression, i.e. genes that correlate, in expression terms, with each APOBEC3 member. By using both public databases and expert-curated gene sets to annotate these gene co-expression, we suggest functional pathways specific to or shared between particular APOBEC3 genes. We have created a way of visualizing functional annotations, which we term ‘functional barcodes’, and use these to compare functions of APOBEC3 co-expressing genes. Surprisingly, these analyses highlight a diversification, consistent across cancer and tissue types, in the roles of APOBEC3 family members with respect to immune-related and cell cycle/DNA-repair-related functions in both cancer and non-cancer samples. Accordingly, these analyses have extracted a wealth of gene expression correlations for the biological community to mine and design targeted experiments for validating and probing A3B’s involvement in cancer mutagenesis. We have made available the data generated in these analyses in the Supplementary Data of this paper, and an applet accessible on http://fraternalilab.kcl.ac.uk/apobec-barcodes/, where users can interactively browse functional barcodes for any APOBEC3 gene in the different cohorts we have examined.

## MATERIALS AND METHODS

### Data sources

#### Bulk RNA-seq transcript quantification data

**Figure 1.**
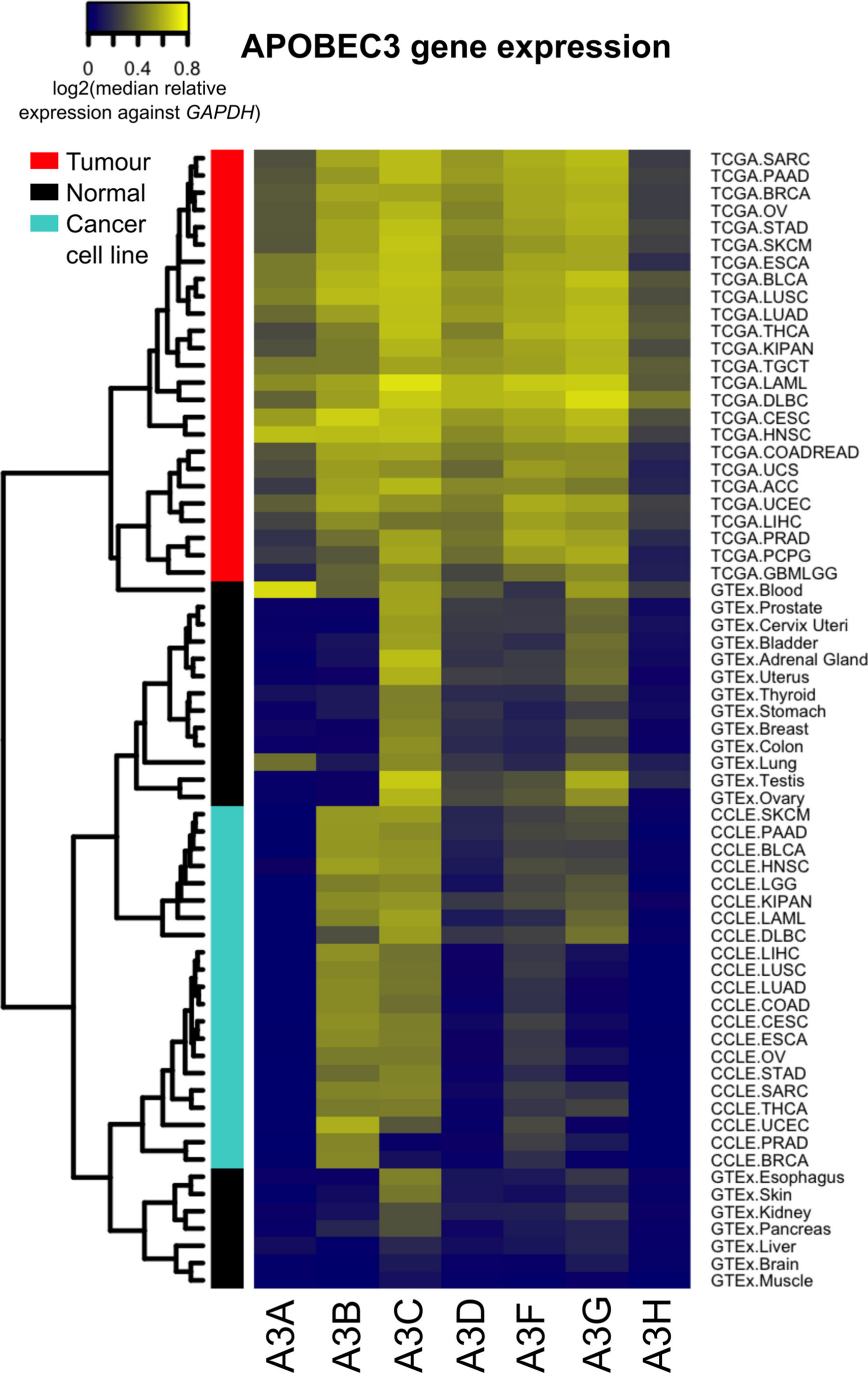
APOBEC3 gene expression in tumours, cancer cell lines and normal tissues of different organs. The median expression value of each APOBEC3 gene in each cohort was normalized against the *GAPDH* gene. In the heatmap, cancer/tissue-types are organized by rows and APOBEC3 (A3) genes by columns. The nature of a cohort (tumour/cancer cell-line/normal) is indicated by the vertical colour-coded bar: red, tumour; black, normal tissues; turquoise, cancer cell lines.

All data analysed in this study are publicly and freely available. RNA-seq data from three publicly available datasets were collected from online sources. For cancer tissues, TCGA expression data (RSEM_v2 version normalized by gene) of 25 cancer types, as detailed in Supplementary Table S1, were downloaded from the Broad GDAC Firehose database (https://gdac.broadinstitute.org/, 28 January 2016 run). For cancer cell lines, RNA-seq data of CCLE (Transcript per Million [TPM] values) were downloaded from the CTD^2^ data portal (https://ocg.cancer.gov/programs/ctd2/data-portal) on 3 February 2017. For normal tissues, GTEx RNA-seq data (v6p) (Reads per Kilobase per Million Reads [RPKM]) were downloaded from the GTEx data portal (https://gtexportal.org/home/). Each cancer/tissue type was quantile-normalized and log_2_-transformed independently. Matching of cancer cell lines against TCGA cancer types was manually curated against the annotation by CCLE and data from the COSMIC database. All cohorts of TCGA were matched with cell lines in our CCLE dataset, except Adrenocortical carcinoma (ACC), Pheochromocytoma and Paraganglioma (PCPG) and Testicular Germ Cell Tumours (TGCT). Owing to the differences in the processing and transcript quantification for the three databases, all expression comparisons across the three types of samples (e.g. Figure 1) were made after normalizing expression values to *GAPDH*. This was not a concern to the co-expression analysis, which were calculated independently per cancer/tissue type per cohort. For calculating correlations, only cohorts with *n* > 3 were included; for this reason, there were no cell line co-expression analysis for Uterine Corpus Endometrial Carcinoma (UCEC) and Uterine Carcinosarcoma (UCS) (Supplementary Table S1). Gene names were mapped to Human Genome Organization Gene Nomenclature Committee (HGNC) symbols wherever possible; symbols provided the original data were retained otherwise. All abbreviations of cancer types are given in Supplementary Table S1.

#### Single-cell RNA-seq transcript quantification data

Two single-cell RNA-seq datasets were downloaded from the NCBI Gene Expression Omnibus (GEO) database: (i) A dataset of 11 primary breast tumours with two lymph node metastasis samples (20) (Accession GSE75688), and (ii) a dataset of two lung adenocarcinoma patient-derived xenografts (PDX) and 1 lung cancer cell line (H358) control (21) (Accession GSE69405). Dataset (ii) was enriched for tumour cells while dataset (i) was not. For dataset (i), the original publication (20) described blacklisting a subset of single cells for reasons of data quality; these blacklisted cells were excluded in this analysis here. For both datasets the matrices of TPM across the transcriptome were quantile-normalized and log2-transformed. Visualization was produced after normalizing expression of selected genes (Figure 4C) against *GAPDH* expression level in each cell. Dataset (i) (the breast cancer dataset) was further utilized in testing the RESPECTEx pipeline (see section ‘The RESPECTEx pipeline’).

#### Tumour purity estimates

We obtained estimates of purity for each TCGA sample from the work of Aran *et al.* (22), who compiled for each sample a composite purity estimates (CPE), by integrating purity information gathered form several approaches, including clinicopathological tumour purity assessments based on immunohistochemistry (IHC), and results from several tumour purity algorithms including ESTIMATE (23). Here the CPE, ESTIMATE and IHC-based tumour content data were taken and independently examined. To perform the correlation analyses of purity against APOBEC3 expression in the GTEx samples as a control, an estimate of ‘normal purity’ (defined as the proportion of non-immune cells in GTEx samples) was also calculated, by using the ESTIMATE algorithm (23) and following procedures detailed in Aran *et al.* (22). Briefly, ESTIMATE outputs three scores for each sample: an immune score, a stromal score and an ESTIMATE score (which is a composite score summarizing the former two scores), all of which are demonstrated to be inversely proportional to tumour purity according to the authors (23). The CPE estimates and immune scores for the TCGA samples were taken together and their relationships determined by fitting a smooth spline using the loess.smooth function in R. The ‘normal purity’ of GTEx samples were then predicted with its immune score using this fitted spline. For consistency, all analyses related to tumour/normal purity estimates in the main article were produced with results from ESTIMATE unless otherwise stated. Analyses on the TCGA samples with the CPE and/or IHC-based estimates were included in Supplementary Figures S2 and S3.

#### Gene sets

GO Cell Cycle and Immune Response gene sets were downloaded from the MSigDB database (24,25) (v5.2). Curated gene sets of DNA damage repair genes were taken from the publication by Pearl *et al.* (26). Gene sets of immune cell populations were taken from the work of Angelova *et al.* (27). Sets of genes expressed specifically in each cell cycle phase were curated from a meta-analysis by Fischer and colleagues (28). For each cell cycle phase, genes that were classified to be expressed at that phase in ≥3 studies were included. These gene sets generally have little overlap with each other (Supplementary Figure S10). Two additional gene sets were curated from mass-spectrometry proteome studies of lysine acetylation (29) and SUMOylation (30) respectively. For the lysine acetylation dataset, all genes whose products contain a mapped peptide with acetylated lysine were extracted to form the gene set. For the SUMOylation dataset, all genes whose products contain a mapped and SUMOylated peptide (‘SUMO target score’ defined by the authors (30) ≥30) were included in the gene set.

### Deconvolution of immune cell subpopulations with CIBERSORT

The CIBERSORT (31) R source code (v1.04) was downloaded with the authors’ permission. CIBERSORT inferred, using the gene expression data for each sample, the proportion of each immune cell subpopulation as defined an expression matrix of marker genes in each cell type to be examined. The LM22 matrix of marker genes representative of 22 immune subpopulations curated by the authors (31) were used, except that the APOBEC3 genes were removed from the matrix prior to the inference (*A3A* and *A3G* were found in the original matrix). The algorithm estimated a P-value of the inference for each sample. Only samples with a significant P-value (*P* < 0.05) in CIBERSORT results were considered. The number of samples available for analysis in each cohort after such filtering is included in Supplementary Table S1. The Spearman correlation between the inferred level of each immune cell type with the expression level of each APOBEC3 gene was calculated for each cohort.

### The RESPECTEx pipeline

We created a pipeline called RESPECTEx (REconstituting SPecific Cell-Type Expression) which took tumour and immune cell type proportion estimates further, and deconvoluted the observed gene expression level by means of a linear regression approach. We reasoned that in each sample, each cell type present contributed a variable level of gene expression to the observed value, the contribution of each cell type weighted by the proportion of the cell type present. Therefore, by regressing the observed gene expression level against the proportions of each cell type in the cell mixture, the resulting set of coefficients represented the mean expression level in each of the cell types. Mathematically, this is represented as follows (Equation 1):

(1) \begin{equation*}{Y_{g,s}} = \left[ {\begin{array}{ccccc} {\beta _{g,1}} & {\beta _{g,2}} & {\beta _{g,3}}& \ldots & {\beta _{g,c}} \end{array}}\right]\ \left[ {\begin{array}{c}{x_{1,s}}\\ {x_{2,s}} \\ {x_{3,s}}\\ \vdots \\ {x_{c,s}} \end{array}}\right]\end{equation*}

where *Y_g,s_* = observed gene expression value for gene *g* in sample *s*, *β_g,1_, β_g,2_ … β_g,c_* = the mean expression value for gene *g* in a pure population of each of the *c* cell types, and, *x_1,s_, x_2,s_ … x_c,s_* = the proportion of each of the *c* cell types in sample *s*.

For the TCGA samples we took these values from the proportion of tumour cells (22) (the tumour purity, corresponding to cell type #1) and the 22 inferred immune subpopulations (cell types #2 to #23; i.e. *c* = 23) using CIBERSORT (31) as discussed. For each case the size of the immune component is defined as (1 – tumour purity) and the proportion of each immune subpopulation was adjusted by multiplying this value.

Hence, the *β* corresponding to the cell type of interest is the desired quantity. In particular, *β_g_*_,1_ was taken as the estimated expression level corresponding to the tumour cell component. We repeated these procedures on the GTEx samples, taking the ‘normal purity’ (see above) instead to adjust the infiltrated component. Here *β_g_*_,1_ represents the expression level contributed by the non-immune (and thus the tissue-specific cell type according to our definition) cells in the sample. In contrast the sum of *β_g_*,*_n_* (where *n* ≠ 1) represent the total contribution by the immune component.

The cell-type proportion matrix was constructed by concatenating the estimated cell-type proportions (i.e. tumour plus the 22 immune cell types inferred in CIBERSORT) for each case. This was taken as the feature matrix on which the linear regression was performed. The coefficients in the regression model were constrained ≥0 (such that the estimated mean expression levels were non-negative) by using non-negative least squares regression implemented in the nnls package (https://CRAN.R-project.org/package=nnls) (v1.4) in R. The coefficient corresponding to each cell type was taken and weighted by the median sample proportion for the respective cell type to reflect the realistic expression level in tissue/tumour samples. For this analysis, the gene expression levels of the seven APOBEC3 genes were deconvoluted and quantified as described in the Results section.

To test the performance of RESPECTEx we utilized the breast cancer dataset (20) described in section ‘Single-cell RNA-seq transcript quantification data’ in ‘Data sources’, in which the authors also generated RNA-seq data on pooled single cells from each tumour as validation of scRNAseq data quality. Of note, the lung cancer dataset (21) was not used in validating RESPECTEx, because an enrichment step to capture tumour cells was performed in that study. Therefore it was expected that little immune cells would have left in the sample sequenced, which would impact on the performance of immune deconvolution. We treat these pooled cell data as tumour admixtures, and estimate the tumour purity with ESTIMATE (23) (using identical procedures when we processed GTEx data) and immune cell proportion estimation with CIBERSORT (31). The quantile-normalized, log2-transformed pooled RNA-seq data and these cell type proportion estimates were subjected to the RESPECTEx pipeline, and mean expression in each cell type for each gene were reconstituted. These values were compared with single-cell data, in which we extracted single cells expressing certain gene markers, calculated their mean expression per gene, and visualized and computed their correlations (Figure 4D; Supplementary Figure S9).

### Gene co-expression analysis and functional barcoding

To extract co-expressing genes, we calculated the Spearman correlation of the expression value of itself against each APOBEC3 gene for each gene in the expression matrix. Here all expression values were taken from tumour bulk, without adjustment by the RESPECTEx pipeline. From the correlation values, we constructed an expression correlation network for each cancer/tissue type in each dataset (Figure 5A). For each APOBEC3, genes that correlated with the expression of the APOBEC3 gene with an absolute standard score ≥2 were defined as co-expressing genes of the APOBEC3 gene. Correlations were calculated per cancer/tissue type. The extraction of co-expressing genes is dependent on the distribution of correlation values: the number of co-expressed genes extracted for each APOBEC3 gene in each cancer/tissue type varies (Supplementary Table S10), and could be 0 if no genes satisfy the criterion of the standard score stated above. This ensures that co-expressing genes extracted in this pipeline are genes that show significantly stronger associations with the APOBEC3 gene in question, relative to other genes. We devised for this analysis a visualization pipeline to plot these gene co-expression data and display their functional annotations. This consists of two visualizations: first, a Circos plot to display gene co-expression and highlight co-expressing genes common to multiple APOBEC3 genes; second, a functional barcoding method to display functional annotation of the co-expressing gene.

For displaying co-expressing genes, initially we visualized the gene co-expression network with conventional hairball visualizations, but found it challenging to compare networks derived across different cohorts. We therefore devised a Circos plot to show this (Figure 5A), fixing the positions of all APOBEC3 genes and their co-expressing genes along the circular axis. Here the circle was divided into seven segments, each corresponding to each APOBEC3 gene. Co-expressing genes of each APOBEC3 gene are listed along the axis in the respective segment, sorted numerically by their correlation coefficients with the APOBEC3 gene in question. Shared co-expressing genes were listed only once in the plot, in the segment corresponding to the APOBEC3 gene with which the gene correlated the most strongly.

For the functional barcodes, mapping and annotation of the co-expressing genes to gene sets was described in the Results section (Figure 5B). Functional barcodes are a visualization means which allows for qualitative comparisons; for quantitative assessment, the enrichment of co-expressing genes in different gene sets was assessed by ranking each list based on their correlation coefficients, and tested statistically with the standard GSEA method using the R fgsea (v0.99.6) package (*bioRxiv*, https://doi.org/10.1101/060012) and the GSEA java application (v2.0) from MSigDB (24). For GO BP gene sets, only those of size within the default cut-offs in the GSEA MSigDB java application (between 15 and 500) were considered.

### Extraction of distinguishing genes

We divided the TCGA cancer types into two groups, one group with a reported, widespread APOBEC3-mediated mutational signature (4) versus those tumour types without such report. For each APOBEC3 gene, we assessed, for each co-expressing gene, the difference between the Spearman correlation (with the particular APOBEC3 gene) in the two groups of cancer types. Those genes with a significant (*P* < 0.05) difference (Wilcoxon test) were termed ‘distinguishing genes’. Tumour types with a widespread APOBEC3-mediated mutational signature were defined as the six cohorts identified in Roberts and colleagues’ analysis (4) of TCGA data: breast invasive carcinoma (BRCA), lung adenocarcinoma (LUAD), lung squamous cell carcinoma (LUSC), bladder urothelial carcinoma (BLCA), cervical and endocervical cancers (CESC) and head and neck squamous cell carcinoma (HNSC). The same procedure was taken to analyse the correlation data for the GTEx samples; here a set of four tissue types matching the cancer types mentioned above (breast, bladder, cervix uteri and lung) are compared against the rest (Supplementary Table S1).

### Sampling controls

**Figure 2.**
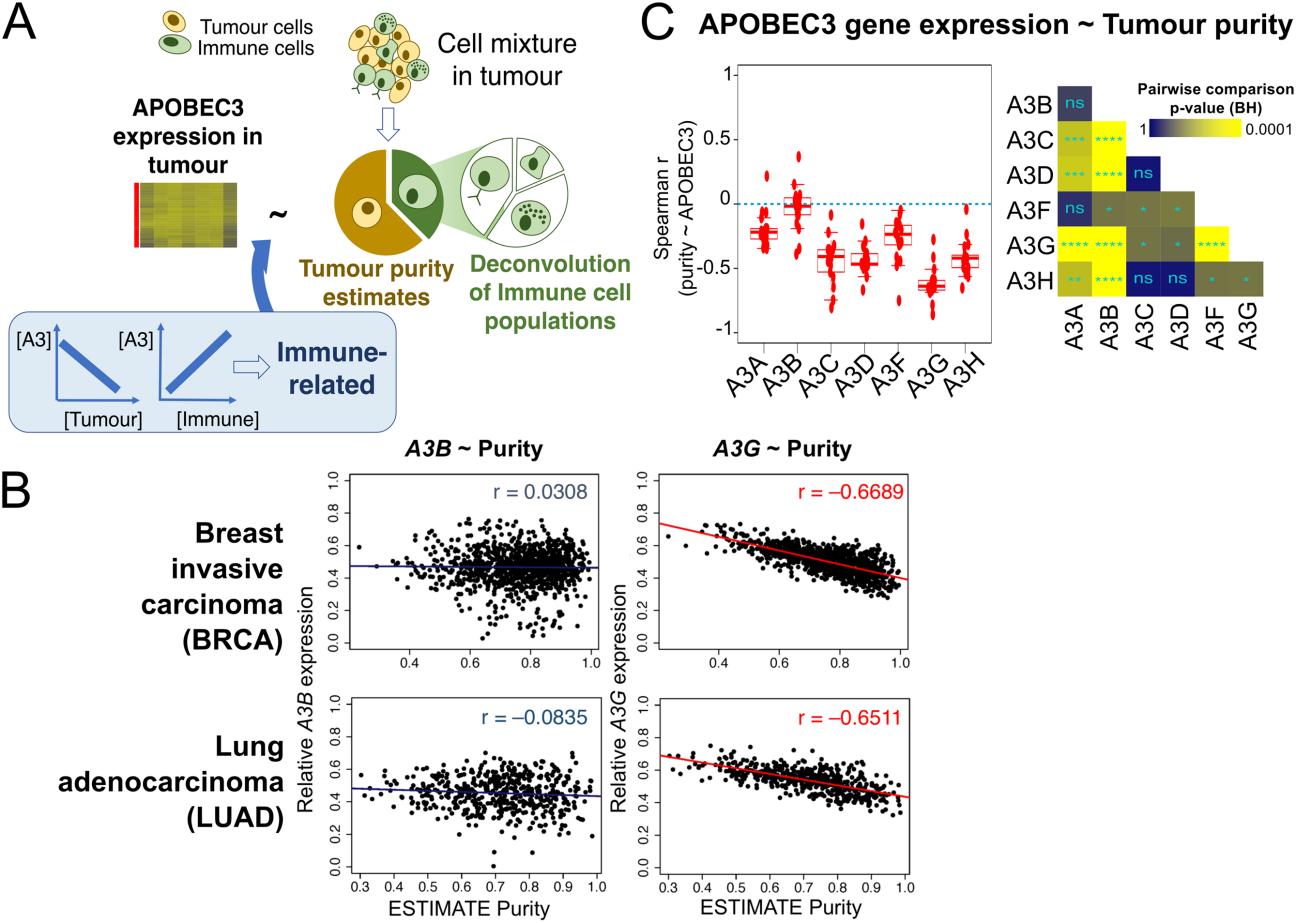
Relationship between APOBEC3 gene expression and cell type composition of samples. (**A**) The relationship between APOBEC3 (A3) gene expression in tumours with the proportion of tumour cells and infiltrated immune cells. If the expression of an A3 gene is negatively associated with tumour purity (that is, the level of tumour cells within the sample), but positively associated with immune cell level, the gene is likely to be immune-related. (**B**) *A3G* expression, but not *A3B*, is negatively associated with tumour purity. Here we plot the relationship between tumour purity (ESTIMATE algorithm) and APOBEC3 gene expression (normalized against *GAPDH* expression) for TCGA breast invasive carcinoma (BRCA) and lung adenocarcinoma (LUAD) cohorts. Spearman correlation values are shown and color-coded by statistical significance (red indicates *P* < 0.05; blue otherwise). See Supplementary Figure S2 and Supplementary Table S2 for the correlation analysis using other tumour content measurements/estimates. (**C**) Over all TCGA cohorts the expression of all APOBEC3 genes, except *A3B*, are anti-correlated with tumour purity levels. Spearman correlation coefficients calculated as in (B) but for all TCGA cohorts examined. On the left the distributions of Spearman correlation values for each APOBEC3 gene are displayed as boxplots; each data point represents one TCGA cohort. Pairwise tests of differences of these distributions (Dunn's test) are displayed on the right. ****Benjamini–Hochberg (BH)-adjusted *P*-value < 0.0001; ***P < 0.001; **P < 0.01; *P < 0.05; ns, not significant. See Supplementary Figure S3 for the correlation analysis using other tumour content measurements/estimates, and results from the respective randomized controls. The exact adjusted p-values are given in Supplementary Table S3.

**Figure 3.**
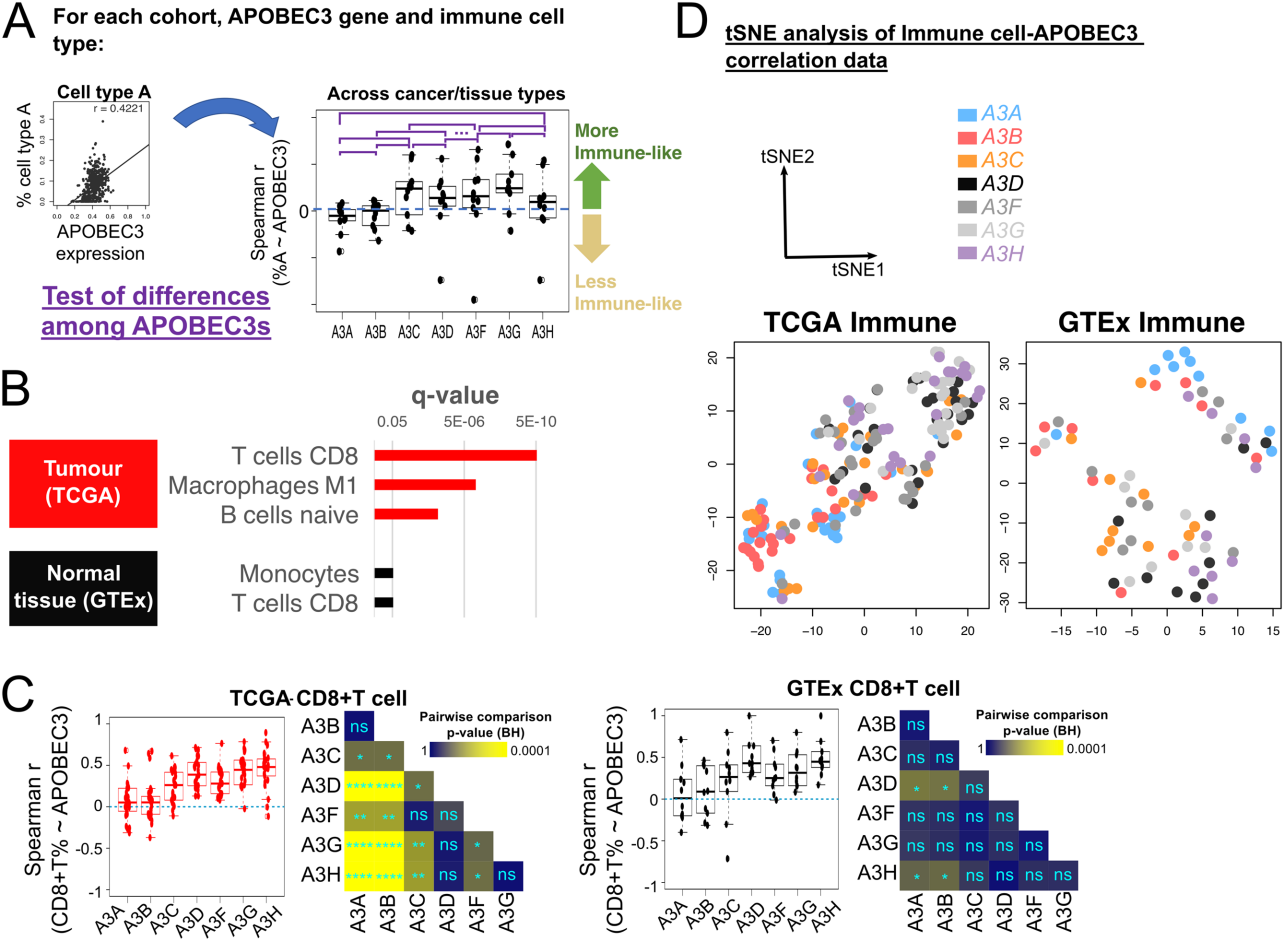
Differential association of APOBEC3 genes with an activated immune environment. (**A**) Schematic to illustrate correlation calculations and tests of differences of the distributions of correlation coefficients for each APOBEC3 gene with an immune cell type. (**B**) Immune cell types whose levels show different correlations with different APOBEC3 genes in TCGA and GTEx cohorts. P-values were obtained via the test of difference as illustrated in panel (A) using a Kruskal-Wallis test, and corrected using the Benjamini-Hochberg method. Only cell types with a significant difference across APOBEC3 are shown. See Supplementary Table S4 for the complete tables of these results. (**C**) Correlation between CD8^+^ T cell level and APOBEC3 gene expression in TCGA and GTEx cohorts. Each boxplot represents the distribution of Spearman correlation values (individual data points represent individual cancer/tissue-types). Pairwise test and statistical significance was evaluated identical to Figure 2C. See Supplementary Figure S4 for results for other immune cell types shown in panel (B), and Supplementary Table S6 for the p-values depicted in the grids. (**D**) tSNE dimensionality reduction of APOBEC3-immune correlation data. One data point corresponds to the correlation data of immune cell levels with the respective APOBEC3 gene in one cancer/tissue type. The segregation of *A3B* (pink) and *A3G* (light grey) data points are clear especially for TCGA. See Supplementary Figure S6 for t-SNE results over a range of parameters (Methods).

**Figure 4.**
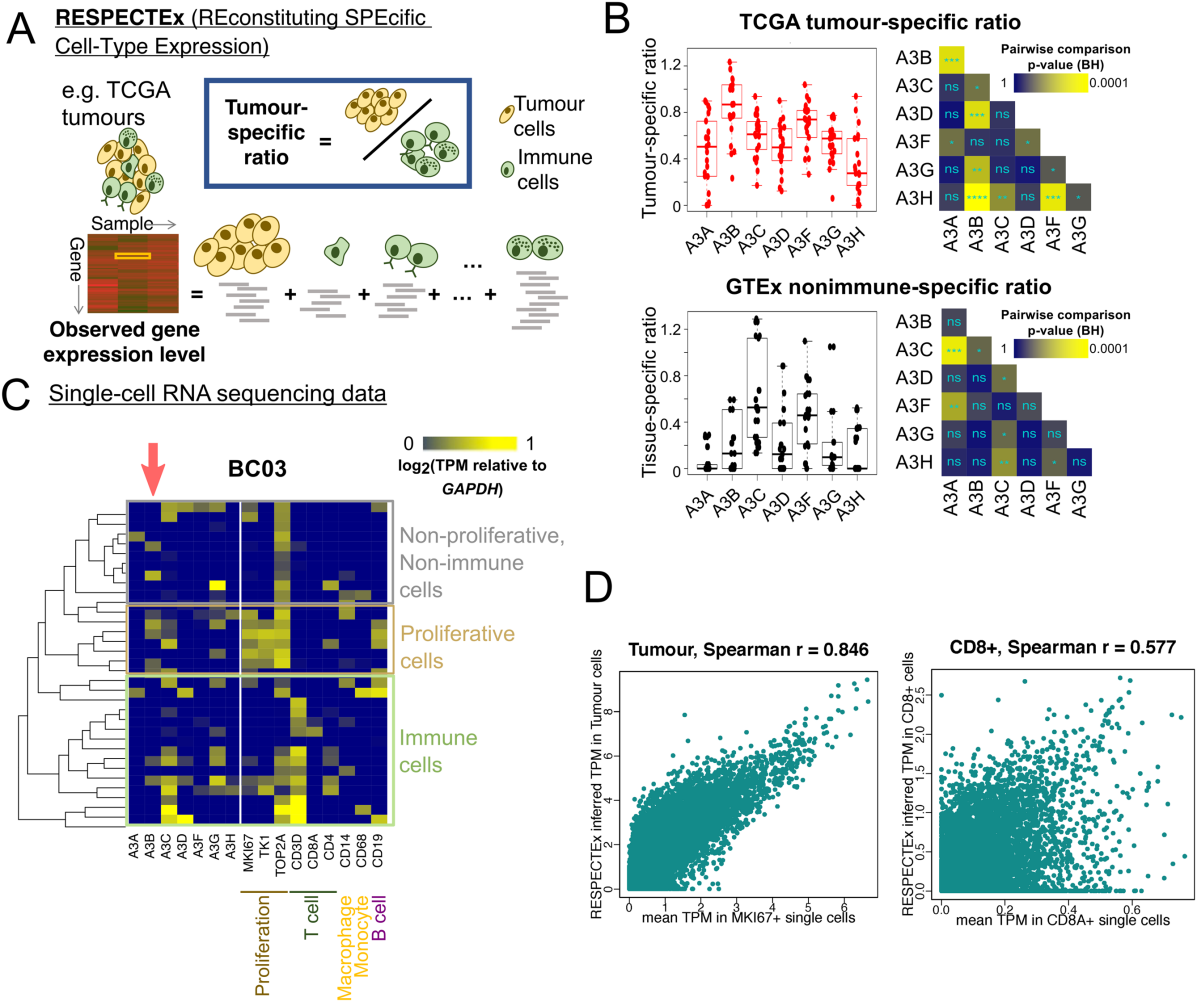
Deconvolution of cell-type-specific APOBEC3 gene expression. (**A**) Schematic of the RESPECTEx pipeline to deconvolute cell-type-specific gene expression, by regressing the observed gene expression level in a sample (the cell mixture) against the proportions of cell types. See main text and Methods for details. (**B**) Distributions of tumour/nonimmune-specific ratio calculated using RESPECTEx-reconstituted expression values, for each APOBEC3 gene in TCGA and GTEx cohorts. Each data point represents one individual cancer/tissue type. Pairwise tests of differences and statistical significance as evaluated identical to Figures 2C and 3C. (**C**) A representative case (sample BC03) of single-cell RNA sequencing (scRNAseq) data from a breast tumour cohort (data from GSE75688). Relative transcript per million (TPM) values (normalized against *GAPDH*) were plotted. Marker genes for respective cell types (see annotations) are shown alongside expression of the APOBEC3 genes. The column corresponding to *A3B* is highlighted with a pink arrow. Expression heatmaps of scRNAseq data from other cases examined can be found in Supplementary Figure S8. (**D**) Comparison of RESPECTEx-attributed specific gene expression for tumour cells and CD8+ T cells, with expression profiles collated from marker gene (*MKI67* for tumour cells; *CD8A* for CD8+ T cells) positive single cells from the breast tumour cohort GSE75688 visualized in panel C and Supplementary Figure S8. Here, the RESPECTEx-inferred TPM values were plotted against the mean TPM from marker gene-positive single cells. Each data point represents one gene. Spearman correlation values were stated in the plot titles. See Supplementary Figure S9 for similar plots for other marker genes displayed in panel C.

Sampling controls for the following analyses were described in this section:

- Correlation analysis between APOBEC3 gene expression and tumour purity (Figure 2);
- Correlation analysis between APOBEC3 gene expression and immune cell proportions (Figure 3);
- Expression deconvolution analysis (Figure 4);
- APOBEC3 co-expression analysis (Figure 6).

For (i) and (ii), the case labels for the cell type proportion data were randomized for 100 times, the correlation of gene expression with the randomized cell type proportion was calculated in each randomization. The median from these randomized correlations were taken to represent each cohort. These randomized controls gave random correlations as expected (Supplementary Figures S3, S5; Supplementary Tables S2 and S5). For (iii), the case labels for the feature matrix were randomized for 100 times and the regression procedure (see above) was performed on each randomized matrix. The resulting coefficients from each iteration were independently analysed and the tumour/tissue-specific ratio (Figure 4B) for each iteration, before taking the median to represent each cohort. Additionally, a randomized gene control was taken to monitor the dependence of these analyses on the gene identity, in which for each case-randomizing iteration the gene labels were also randomized before the extraction of deconvoluted expression values (Supplementary Figure S7; Supplementary Table S7). For (iv), to monitor the extraction of co-expression partners, correlation calculations were also calculated for a randomly sampled population, in which for each cancer/tissue type 10% of the cases or 10 cases, whichever more, were randomly sampled for 100 times. The correlation of expression with the APOBEC3 genes was examined in each iteration. Cohorts with *n* < 10 were not considered in this randomization approach. Each sampling population typically has low (∼10–30%) overlap in terms of the co-expression partners extracted in comparison to those extracted based on correlations calculated over the entire cancer/tissue type (Supplementary Table S11). Co-expression partners were also extracted over the median correlation across this set of subpopulations. Using this approach, the co-expression network and functional barcodes are very comparable to that extracted over the entire cancer/tissue type without bootstrapping (cf. Figure 6 and Supplementary Figures S13–S17).

### Statistics and data visualization

All analyses were performed in the R statistical programming environment. Normalization of expression matrices was performed using the normalize.quantile function in the preprocessCore R package (https://github.com/bmbolstad/preprocessCore). Spearman correlations were calculated and assessed with two-way tests with the cor.test function in R wherever indicated. Two-way statistical significance (*P* or *q* < 0.05) was evaluated either with Wilcoxon (pairwise comparisons) or Kruskal–Wallis rank sum (one-way group comparisons) tests and corrected using Storey's method for multiple testing (32) or the method by Benjamini and Hochberg (33) as stated in the relevant description. Trend lines in scatter plots were fitted with the lm function in R unless otherwise stated. *t*-Distributed Stochastic Neighbour Embedding (t-SNE) was performed using the Rtsne package (v.0.13), using parameters max_iter = 1000. Values were centred and scaled by cohort before performing this procedure on a list of perplexity values: [2, 5, 10, 20, 30, 40, 50, 100]. For each perplexity value in the list, unless it is large enough to invoke error messages from the algorithm, 100 fittings were performed, and the fitting which gave the lowest Kullback–Leibler divergence was taken and analysed. A representative t-SNE visualization was selected and included in the Results section. Visualizations under other perplexity values were included in Supplementary Figure S6. Heatmaps were produced with the heatmap.2 function in the gplots package (https://CRAN.R-project.org/package=gplots) (v.3.0.1), in which clustering, wherever shown, was performed with hierarchical clustering (function hclust) using default parameters in R. Networks were visualized using the Circos package (34) (v.0.69-6) in Perl. All other plots were produced with plotting utilities in base R. A standard colour code is adopted throughout the manuscript: (1) sample type (TCGA/GTEx/CCLE, see Figure 1); (2) APOBEC3 gene (see e.g. Figures 3, 6 and 7); (3) Gene sets (Figures 6 and 7). All scripts for data analysis and visualization are available upon request.

## RESULTS

This analysis considered transcriptomic data from 25 cancer types, including 8,951 TCGA tumours, 786 cancer cell lines (from the Cancer Cell-line Encyclopaedia [CCLE]), as well as 6,119 tissue samples from nominally healthy individuals in the Genotype-Tissue Expression (GTEx) project (Supplementary Table S1). The aims were to compare the expression patterns of the APOBEC3 genes in tumours, cancer cell lines and normal samples, and extract functional pathways under which each APOBEC3 gene is expressed, by annotating gene co-expression data (Supplementary Figure S1). Throughout these analyses, we use the following colour code in our figures: blue, *A3A* gene; pink, *A3B*; orange, *APOBEC3C* (*A3C*); black, *APOBEC3D* (*A3D*); dark grey, *APOBEC3F* (*A3F*); light grey, *A3G*; purple, *A3H*.

### Cell-type composition of tumours influence APOBEC3 gene expression

The expression levels of APOBEC3 genes in tumours, cancer cell lines and normal tissues are visualized in a heatmap (Figure 1). This provides an initial classification for the different roles played by the seven APOBEC3 genes: (i) in normal healthy tissues the expression levels of APOBEC3 genes are distinct from one another. Typically, *A3A, A3B* and *A3H* levels are low in most tissues, while *A3C* and, to a lesser extent, *A3G*, are highly expressed in general. On the other hand, *A3A* appears to be very tissue-specific, and shows high expression in healthy lung and blood samples, as previously reported (10,35). (ii) *A3B* and *A3C* are highly expressed in most cancer cell lines. Of note, *A3B* is the only APOBEC3 gene to be upregulated, across cancer types, in cancer cell lines versus normal samples. The ubiquitous overexpression of *A3B* in cancer samples supports the argument (5,10) for this family member being the APOBEC3 deaminase that most likely causes mutational signatures in cancers. (iii) The levels of all seven APOBEC3 mRNAs are high in tumours, in contrast to the specific expression patterns observed in cancer cell lines (Figure 1). The specificity of *A3A*, as observed in the normal tissues, is lost in tumours, where it is upregulated to varying degrees across cancer types.

We reasoned that for the analysis of bulk samples, especially the tumour samples, the expression data may be heavily influenced by infiltrating cells, which include immune cells that can contribute APOBEC3 mRNAs to the overall transcript counts. To address this, we asked how the cell type composition of a sample influences the observed APOBEC3 gene expression levels (Figure 2A). While non-immune cells (e.g. cancer-associated fibroblasts (36)) are also present in the stromal component of tumours, here we focus on deconvolving immune cell populations of tumour cell admixtures to delineate APOBEC3 gene expression in a cancer context from their transcripts in immune cells in which they are known to be expressed (18,19). We collected estimates and measurements of tumour purity (22,23) (in the case of normal healthy tissues, estimates of the proportion of the non-immune component in a sample, see Methods), and examined the correlation between these quantities and APOBEC3 gene expression. If the expression of an APOBEC3 gene is negatively correlated with tumour (or non-immune) purity but positively correlated with the level of immune populations, the expression is likely to be attributed to infiltrating immune cells (Figure 2A). From this analysis, we observe that the expression of all APOBEC3 genes, particularly *A3G*, negatively correlates with tumour purity in multiple cancer types. The only exception is *A3B*, whose expression exhibits either no or weakly positive correlations with tumour purity (Figures 2B–C; Supplementary Figures S2 and S3; Supplementary Tables S2 and S3). Interestingly, *A3F* and *A3A* correlate with tumour purity less negatively than other APOBEC3 genes aside from *A3B*. Moreover, the distinction between APOBEC3 genes is much weaker in GTEx samples (Supplementary Figure S3). We observe that the differences amongst APOBEC3 genes are weaker when we consider calculations using immunohistochemistry (IHC) based tumour purity estimates, probably reflecting the nature of such qualitative assessments in estimating tumour purity, in comparison with the other methods (22,23) that we considered which utilize measurements of tumour-specific genomic aberrations (Supplementary Figure S2 and S3). We next examined the association of APOBEC3 gene expression with the immune component of tumour tissues. We performed immune cell deconvolution using CIBERSORT (31), extracted immune cell types which show different extents of correlation with expression levels of the APOBEC3 genes, and statistically evaluated these differences (see Methods, and Figures 3A and B; Supplementary Table S3). The expression of *A3G* and *A3H* correlate with levels of CD8^+^ T cell and other immune cell types more strongly than *A3B*, which generally exhibits no such correlation (Figure 3C; Supplementary Table S5). We evaluated also the correlations with APOBEC3 gene expression for other immune cell types (Supplementary Figures S4 and S5; Supplementary Table S5). By using t-Distributed Stochastic Neighbour Embedding (t-SNE) (37), we projected these correlation data onto a 2-dimensional plot. When we consider TCGA tumours, the correlation of APOBEC3 gene expression and immune cell levels are distributed across a spectrum, with the correlations involving *A3B* and *A3G* occupying the two ends (Figure 3D; Supplementary Figure S6). Data on specific APOBEC3 gene tends to be clustered, with the exception of *A3C*, whose data points are dispersed across the spectrum. For GTEx, distinctions between APOBEC3 genes are much weaker. Of note, fewer GTEx samples were successful for immune cell deconvolution (Supplementary Table S1), possibly due to the nature of the samples such that they are typically devoid of immune infiltration. Taken together, these results reveal that APOBEC3 expression patterns vary with the cell type composition of a tumour. A3G, a cytidine deaminase extensively studied in antiviral responses (1,38–40), shows immune-related transcription profiles in the tumour mixture. Importantly, not all APOBEC3 genes have the same correlation patterns: in particular, for all immune cell types listed in Figure 3B, no correlation is found between their levels and the expression of *A3B*, the cancer mutagen.

### A pipeline to deconvolute cell-type specific gene expression patterns of APOBEC3 members

Realizing the differential contribution of infiltrated immune cell populations to APOBEC3 expression in tumours, we sought to deconvolute cell-type-specific expression levels by making further use of the tumour purity and CIBERSORT immune cell proportion estimates. While the estimation of the levels of various cell types, tumour, stromal or immune, has become routine in many cancer bioinformatics analyses, few algorithms exist to infer, from gene expression levels in the bulk tumour, contribution from each cell type, and they vary in performance (41) (*bioRxiv*, https://doi.org/10.1101/437533). Existing software packages require, along with tumour bulk expression profiles, normal (matched or unmatched) RNA-seq samples (42,43), which could be problematic since far fewer normal samples were sequenced in RNA-seq profiling generated from large cancer cohorts. Here we devised a pipeline called RESPECTEx (pronounced ‘Respect-X’, ‘REconstituting SPecific Cell-Type Expression’), which use data of the levels of different cell type in a given sample (extracted from CIBERSORT (31)) to estimate the contribution from each cell type to the bulk gene expression level. This is achieved by using a linear regression approach, treating the cell-type proportion estimates as covariates when modelling the bulk gene expression levels (Figure 4A; see Materials and Methods). Hence, we obtain for each cohort the mean estimated expression levels specific to each cell type, which are a straightforward, simple statistic for comparisons across cohorts. RESPECTEx also allows downstream analyses specific to our purposes: we can quantify whether the expression of an APOBEC3 gene is immune or tumour (non-immune) specific, by calculating the ratio of the expression in non-immune cells to that in the immune component. A high value indicates that the gene tends to be expressed more by the non-immune, but less by the immune component of the cell mixture (Figure 4A). A comparison of this measure indicates that *A3B* expression is more specific to tumour cells but less so to normal cells for the GTEx normals (Figure 4B; Supplementary Figure S7; Supplementary Table S7). To validate these comparisons on experimental data and to obtain further insights into the cell-type specificity of APOBEC3 gene expression in cancer, we also acquired single-cell RNA-seq (scRNAseq) data from two studies, one from breast tumours (20) and the other from lung cancer xenograft models (21), and produced heatmap visualizations of the expression levels of the APOBEC3 genes, alongside with a panel of marker genes of different cell populations (Figure 4C). In many single cells the expression pattern of the APOBEC3 genes resemble more closely the cancer cell lines in comparison to the uncorrected tumour expression values. In the scRNAseq data, cells with high expression of the T cell marker *CD3D* often have high *A3G* and *A3C* mRNA expression, while *A3B* mRNA expression is almost exclusive to cells in which proliferative markers are highly expressed (Figure 4C; Supplementary Figure S8). Since RNA-seq data from pooled single cells for each tumour were also available for the breast cancer cohort (20), we decided to test the performance of RESPECTEx on these pooled samples and see whether RESPECTEx-reconstituted gene expression of specific cell-types recapitulate marker gene-positive single-cell samples. We successfully obtained estimates of tumour and various immune cell types (see Methods) for *n* = 8 tumour samples. The expression specific for tumour cells and CD8+ T cells estimated using RESPECTEx were in broad agreement with mean expression levels in *MKI67*+ single cells (for tumour) and *CD8A*+ single cells (for CD8+ T cells) (Figure 4D), confirming the validity of inferences by the RESPECTEx pipeline. Results vary from one cell type to another (Supplementary Figure S9): the inference is most accurate in estimating expression specific to tumour cells (at least ∼ 60% in this dataset), but works less well for cell types which are estimated to be very rare in the tumour admixtures (e.g. monocytes, which in all our cases represent <1% of the tumour cell admixtures, see Supplementary Table S9 and Supplementary Figure S9). Larger cohorts with improved cell type proportion estimation methods could enhance the precision. We envisage that larger datasets on both single- and pooled-cells could verify the accuracy of RESPECTEx-inferred expression.

Altogether, RESPECTEx extends beyond existing tools (31) which are routinely used to obtain cell type proportions in tumour cell mixtures. It can be integrated into analytic pipelines to quantify and compare gene expression levels in tumour and different immune cell types. We demonstrate the distinct expression levels and patterns of the seven APOBEC3 genes in cancer, contrast the immune-related background of *A3G* and other APOBEC3s with the absence of such involvement for *A3B*, and highlight the importance of differentiating signals from immunotypic cells in the observed bulk gene expression data.

### Functional barcoding of gene communities co-expressed with APOBEC3 genes

**Figure 5.**
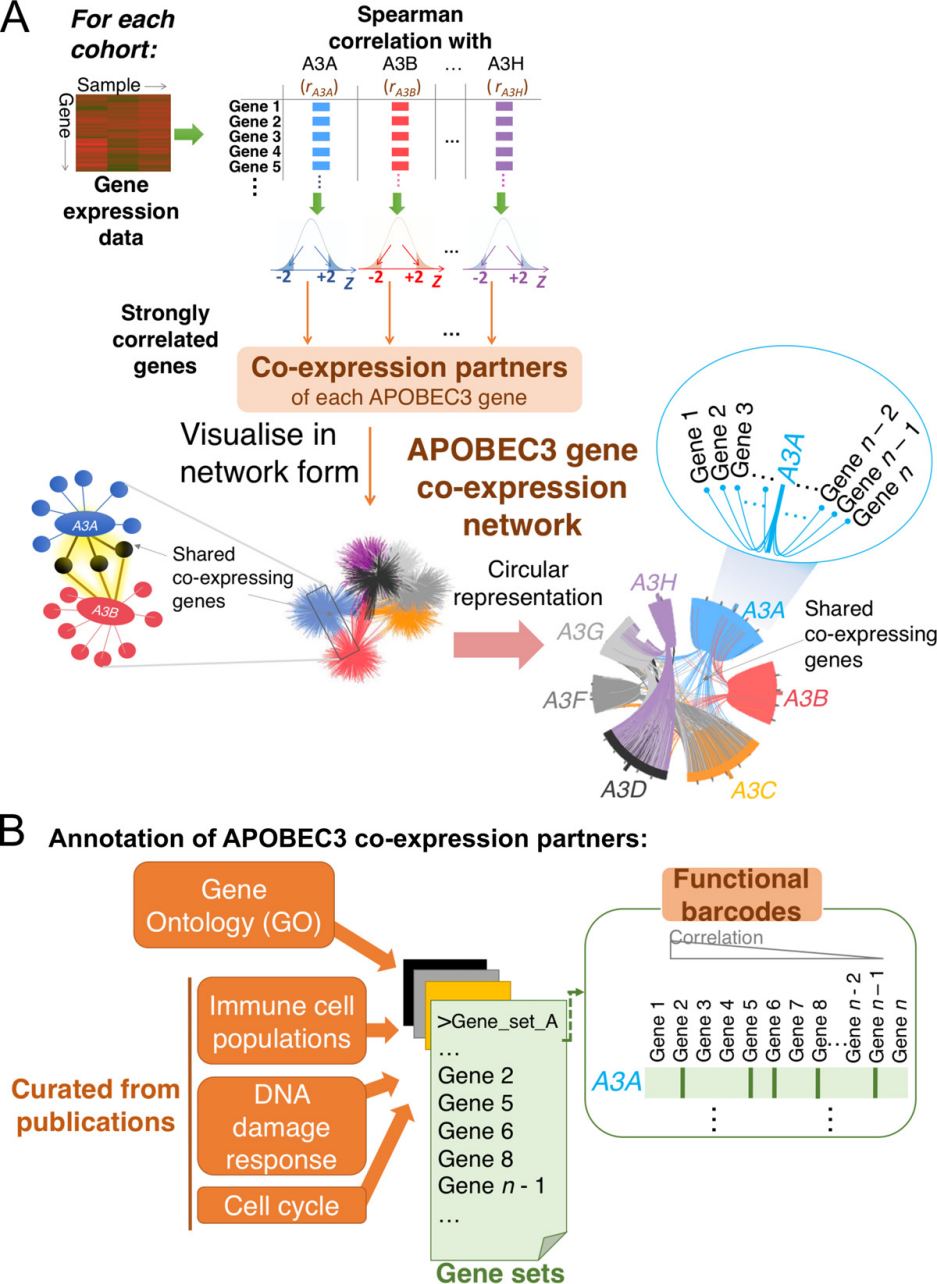
A schematic on extracting and annotating APOBEC3 gene co-expression. (**A**) Co-expressing genes with each APOBEC3 gene were defined as genes that have a stronger correlation with the APOBEC3 gene in question (absolute correlation *Z*-score > 2) than the others. We visualized this co-expression network by fixing the seven APOBEC3 genes on a circular axis and constructing a circular visualization (‘Circos plot’). The co-expressing genes were aligned along the circular axis and connected to the relevant APOBEC3 genes. The extent of shared co-expression networks is illustrated by the number of edges which cross the centre of the Circos plot. (**B**) For functional annotation of the co-expressing genes we used Gene Ontology (GO) gene set collections and other gene sets curated from publications. Overlap of a co-expressing gene with a gene set is indicated with a stroke, eventually generating a ‘functional barcode’.

The results of the expression analysis could imply that each APOBEC3 member is co-expressed with distinct genes, and thus specialises in different biological functions. Therefore, we sought to extract and analyse genes co-expressed with APOBEC3 genes and study their functional annotation. We analysed bulk transcriptomic data, and compared the correlations of the expression values of each gene with that of each APOBEC3 gene. We extracted strong co-expressing genes (normalized *z*-score > 2 or < –2, see Materials and Methods) with each of the APOBEC3 members (Figure 5; Supplementary Figure S10; Supplementary Table S10). Some genes are found to co-express with multiple APOBEC3 genes, while others are unique to one APOBEC3 gene. To aid the comparison of such gene co-expression patterns across cohorts, we have developed a framework to visualize these gene co-expression data. We first deployed a circular representation (Figure 5A; see Materials and Methods), where overlap in the co-expressing genes of APOBEC3 family members are denoted using colour-coded edges which cross over the centre of the plot (Supplementary Figure S13). We then carried out extensive gene set analyses, to characterize functions of these co-expressing genes, by integrating gene sets from databases (e.g. Gene Ontology [GO] Biological Processes), manually-curated gene sets representative of different DNA Damage Response (DDR) pathways (26), and different immune cell populations (27). Gene sets representative of each cell cycle phase were also analysed: these were derived from a recent meta-analysis of genes that showed cell cycle phase-specific expression (28) (Supplementary Figure S11). To represent these annotations, we devise ‘functional barcodes’: the co-expressing genes were first sorted according to their correlation with the APOBEC3 gene in question, and then annotated by considering whether each gene overlaps with our collected gene sets: such mapping was represented by strokes drawn next to the co-expressing gene (Figure 5B). Eventually this constructed ‘functional barcodes’ that summarized the functional enrichment of the gene co-expression of each APOBEC3 gene (Supplementary Figure S12). This is akin to electrophoretic methods in molecular biology, where molecules are resolved on a gel by means of their sizes and generates unique visual patterns: here the co-expressing genes are ‘resolved’ by the correlations they exhibit with APOBEC3 genes, and unique ‘barcodes’ are generated based on their functional annotation. Such stroke annotations have been routinely featured in visualizing statistical evaluation of biological pathway enrichment (24); here we take extensive use of them to compare co-expression functional enrichments across APOBEC3 genes, and identify similarities (and/or differences) in their functional involvement.

**Figure 6.**
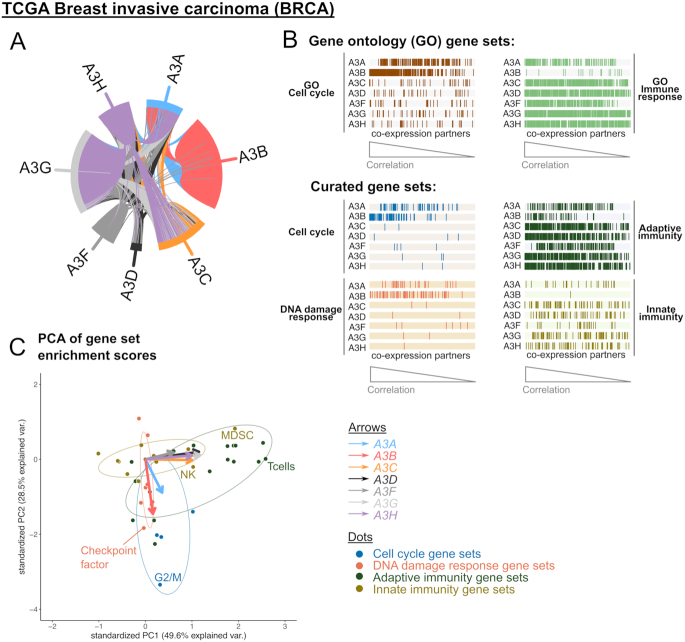
APOBEC3 genes have different co-expression partners. Data from the TCGA breast invasive carcinoma (BRCA) cohort is shown as an example. (**A**) Circos plot representation of the gene co-expression network. (**B**) Functional barcodes of co-expression partners of the seven APOBEC3 genes. For each of the APOBEC3 gene, its co-expressing genes were sorted from left to right by decreasing correlation values. (**C**) Gene set enrichment analysis (GSEA) of the co-expression partners. The normalized enrichment scores of the GSEA tests over our curated gene sets for co-expression partners of all seven APOBEC3 gene were projected onto the first two principle components in a Principle Component Analysis (PCA). One dot represents one gene set (color-coded as per classes of gene sets, see figure legend). Factor loadings (indicated in arrows) show the distinction in pathway enrichment for the co-expression partners of different APOBEC3 genes. Some data points were labelled in the plot; see Supplementary Figure S15 for a completely labelled version. MDSC, myeloid-derived suppressor cells; NK, natural killer cells.

The functional barcodes for co-expressing genes in all examined cohorts can be browsed interactively on http://fraternalilab.kcl.ac.uk/apobec-barcodes/. By querying the online applet (created using R shiny), users can browse the barcodes interactively to look in detail at the annotation of each gene depicted, and download the underlying data for their own further analyses. Users can also go into finer granularity, and browse on the applet functional barcodes generated for gene set signature for a specific immune cell type (say, CD8+ T cells). Here, as an example, an APOBEC3 gene co-expression network for TCGA breast tumours can be found in Figure 6A. We observe differences between APOBEC3 members in the co-expression data. A striking feature is the isolation of the *A3B* co-expressing genes: only a few connections with those of other APOBEC3s are observed. We then compared the functional barcodes of these co-expressing genes: *A3B* co-expressing genes are typically related to cell cycle and DDR pathways; in contrast, *A3G* and *A3H* have co-expressing genes that are strongly enriched in immune processes, and in adaptive and innate immune cell populations (Figure 6B). All other *APOBEC3* genes have similar immune-related gene co-expression partners as in the cases of *A3G* and *A3H*; the only exception is *A3A*, which has additional cell cycle/DDR related co-expressing genes (Supplementary Figure S12). A statistical Gene Set Enrichment Analysis (GSEA) procedure reinforces this observation; here we subject these data to a Principle Component Analysis (PCA) and present it in a bi-plot (Figure 6C). It shows that the loadings which represent co-expressing genes of *A3A* and *A3B* are orthogonal to other APOBEC3 genes in terms of functional pathway enrichment. The co-expression partners of the former are enriched in cell cycle and DDR gene sets, while co-expression partners of the other APOBEC3s are, in contrast, characterized by enrichment in different adaptive (e.g. T cells) and innate immunity (e.g. myeloid-derived suppressor cells [MDSC], natural killer cells) populations (Figure 6C; Supplementary Figure S15). The distinct co-expression of *A3B* is consistent across different cancer types and sample types (tumour/cancer cell line/normal, Supplementary Figure S13) from which co-expressed genes could be successfully extracted. In our quantification of the overlap of co-expressing genes from different APOBEC3 across each cohort, the genes that are co-expressed with *A3C, A3D, A3F, A3G* or *A3H* overlap extensively, especially in the tumours (Supplementary Figure S17). Data from cancer cell lines typically display weaker distinctions among APOBEC3 genes (Supplementary Figure S12; possibly related to the extensive variations inherent to gene expression data collected from this type of samples, see Discussion), in TCGA and GTEx cohorts we observe consistently the distinctiveness in terms of the functional annotation of *A3B*-coexpressing genes. Many genes which co-express with *A3B* amongst most tumour types are known to be involved in processes such as cell cycle regulation (*CDC25C, FOXM1*), DNA replication (*CDC6, CDC45*) and the maintenance of the mitotic spindle (*AURKB, CDCA5, CDCA8*), while the examination of some immune markers shows clear correlation of their expression with that of *A3G* and *A3H*, but not with *A3B* (Supplementary Figure S18). Here, our analyses provide a perspective of the functional consequence of such transcriptomic phenomena: the difference in gene co-expression amongst APOBEC3 family members is consistent across different cohorts, and reflective of their diversity in terms of the biological processes in which they function and the cell types in which they are expressed. Importantly, the functional diversification of APOBEC3 genes is not exclusive to cancers, and specifically those cancer types with reported APOBEC3 mutagenesis (4).

### Annotating genes which distinguish APOBEC3 activation and mutational signature

**Figure 7.**
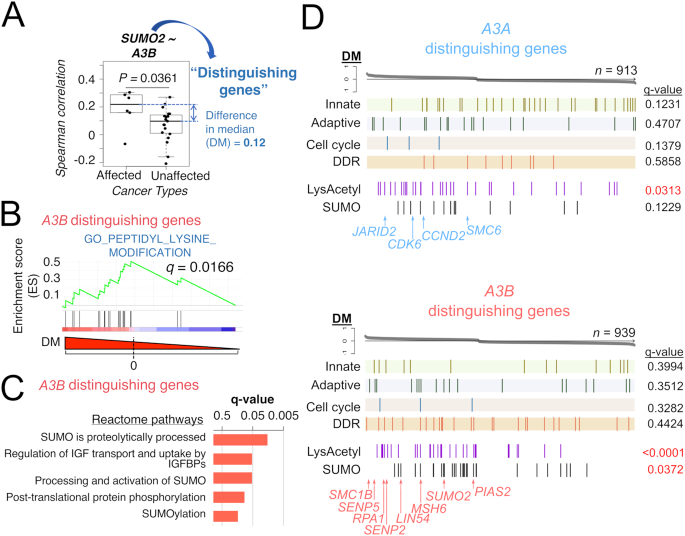
Distinct molecular signatures of genes that distinguish cancer types with widespread APOBEC3-mediated mutational signatures. (**A**) An example of a ‘distinguishing gene’. The correlation of *SUMO2* with *A3B* expression in all TCGA cohorts is plotted. The difference in median between the two groups, here denoted ‘DM’, was taken to rank the gene list for the generation of functional barcodes and GSEA. (**B**) GSEA result for the *A3B* distinguishing genes in the GO_PEPTIDYL_LYSINE_MODIFICATION gene set is shown. This is the only GO biological process with *q* < 0.05 in the GSEA analysis. (**C**) Results from Reactome pathway analysis of *A3B* distinguishing genes. *q*-values for the top 5 pathways are shown. (**D**) Functional barcodes of the distinguishing genes for *A3A* and *A3B*. *q*-values from the GSEA analyses of these six gene sets with the ranked gene lists are given and colour-coded (red: *q* < 0.05 and blue otherwise). Genes mentioned in the text are labelled. See Supplementary Figure S19 for distinguishing gene barcodes extracted from the TCGA cohorts for other APOBEC3 genes, and Supplementary Figure S20 for those extracted from the GTEx normals.

Considering APOBEC3-associated mutagenesis has been observed in some but not all cancer types (e.g. breast, lung etc., see Introduction), and that the co-expression analysis did not discriminate across cancer types (Figure 6B; Supplementary Figures S13–S17), we decide to further investigate this. We focus on those cancer types for which a widespread APOBEC3-mediated mutational signature has been reported (4), and extract the co-expressing genes which display significantly different correlations with an APOBEC3 gene, when compared to those cancer types without such reported signatures (Figure 7A). Next, we annotate the functions of these ‘distinguishing genes’. When we consider the *A3B* distinguishing genes, we observe an over-representation of genes involved in SUMOylation and lysine modification (Figures 7B and C). This includes *SUMO2*, the SENP genes (*SENP2* and *SENP5*, which encode peptidases activating SUMO), and *PIAS2* (whose product is a E3 SUMO ligase). Functional barcodes of these *A3B* distinguishing genes reveal the enrichment of proteins that have been found SUMOylated (30) or lysine-acetylated (29) in cancers (Figure 7D). The same is also observed for *A3A* distinguishing genes. Importantly, such enrichment is absent in the control where we analysed GTEx healthy samples (Supplementary Figure S20; these visualizations can also be browsed interactively on our online applet). This analysis suggests that in tumour types where an APOBEC3-mediated mutational signature has been documented, the co-expressing genes of some APOBEC3s are distinguishable from analogous gene co-expression networks found in tumour types which lack such a signature. While *A3B* was not detected to harbour these post-translational modifications (PTMs) in the mass-spectrometry profiling experiments from which the gene sets were derived (29,30), the PTM signature of *A3B* distinguishing genes reflects a possible connection of the A3B enzyme with regulation of activated processes in these cancers, such as DDR and the cell cycle. In fact, A3B is phosphorylated in the G1 phase according to a mass-spectrometry phosphoproteomics study (44), which might represent one possible mechanism of regulation. More broadly, this analysis of the ‘distinguishing genes’ lead us to conclude that APOBEC3 genes are clearly different from one another in terms of the biological contexts where they are likely to be activated. This analysis also complements the expression deconvolution analysis (Figures 2 and 4) in detailing the distinct roles these play in different cell types in a tumour. In general, our in-depth gene co-expression analyses and ‘functional barcoding’ framework can be exploited in prioritizing genes for experimental investigations in dissecting the involvement of APOBEC3 genes in both cell proliferation and immunity in tumours.

## DISCUSSION

**Figure 8.**
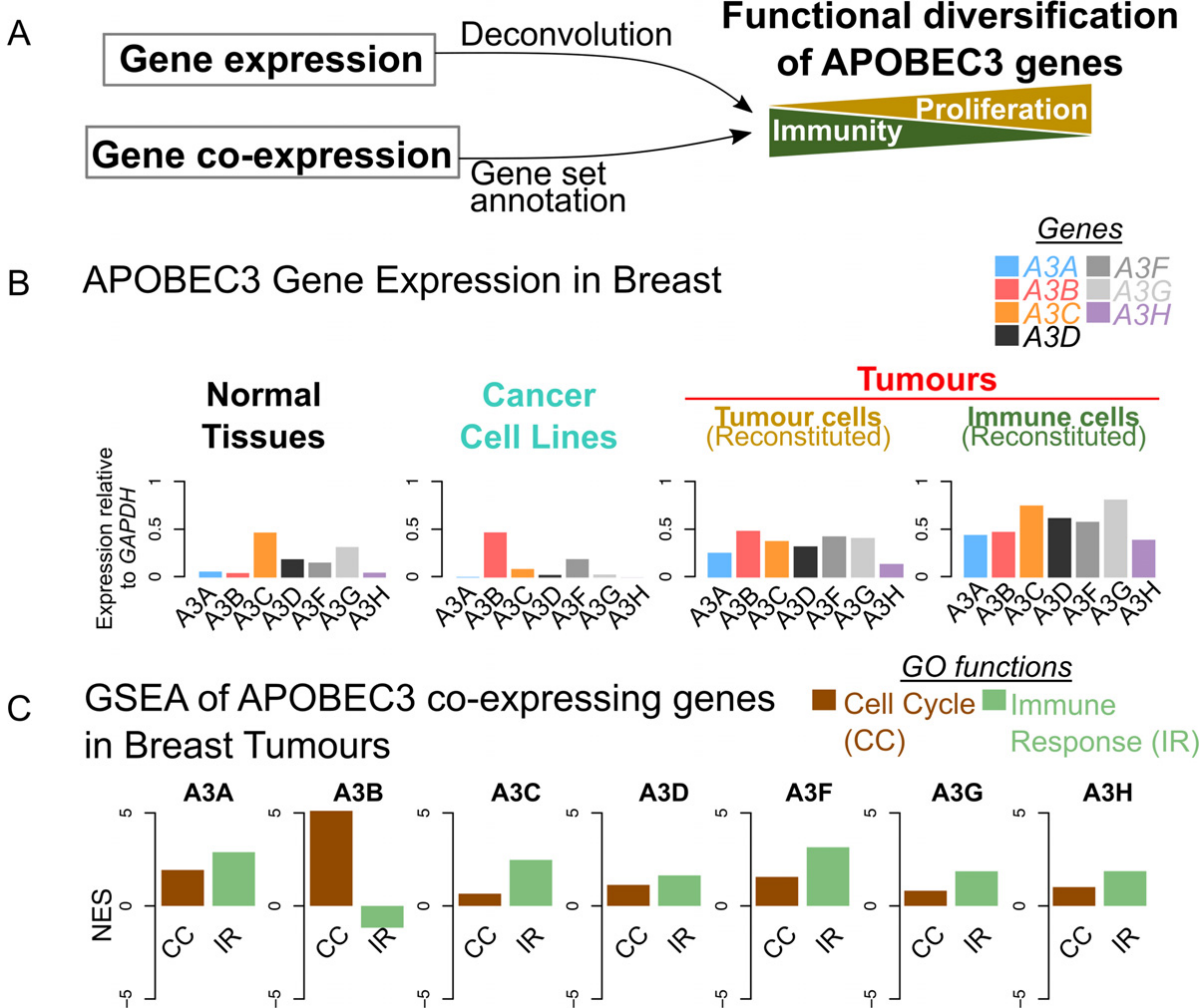
Summary of analyses. (**A**) This work incorporates gene expression and co-expression data, presents bioinformatics analyses of the data (deconvolution of expression and gene set annotation respectively), and infers functional diversification of the APOBEC3 genes in cancer and non-cancer samples. (**B**) A schematic of gene expression analyses in this paper. Data from breast tissues/cells are shown here as illustration. The comparison between data from cancer cell lines, normal tissues (cf Figure 1), and RESPECTEx-reconstituted expression levels in the cancer and immune components of tumours (cf Figure 4) displays substantial differences in the expression patterns of APOBEC3 genes across various cell types. Data shown here are identical to that in Figures 1 (for normal and cancer cell lines) and 4 (for the RESPECTEx-reconstituted cell populations in tumours). See Supplementary Figures S22 (for all examined cohorts) and S23 (RESPECTEx-reconstituted expression profiles for immune and non-immune component of TCGA tumours and GTEx normal tissues) for similar bar-plots. (**C**) A schematic of Gene Set Enrichment Analysis (GSEA) of APOBEC3 co-expressing genes. Here data on TCGA Breast Tumours were depicted. The enrichment of two GO gene sets, Cell Cycle and Immune Response, were shown, and the Normalized Enrichment Score (NES) of these gene sets for the co-expressing genes of each APOBEC3 are plotted. Positive NES denotes an enrichment in the gene set; negative denotes depletion. See Supplementary Figure S24 for similar bar-plots visualized for all examined cohorts.

APOBEC3 genes have been characterized as important inhibitors of retroviral infections and retrotranspositions (1,38–40,45–51), and transcription of these genes are activated in response to immune signals e.g. interferon-alpha (IFNα) stimulation (19). Little is understood about the mechanism of APOBEC3 activation in cancer, except that a few signalling pathways (e.g. NF-κB and Protein Kinase C (PKC) (52)) and driver events (e.g. *ERBB2* amplification, *PTEN* loss (53)) have been suggested to be associated with *A3B* expression and/or the APOBEC3 mutational signatures. Cescon and colleagues (54) sought to analyse *A3B* co-expression in breast cancer, yet a systematic and extensive analysis to uncover functional differences among all APOBEC3 genes in large datasets is lacking. Here we present a pan-cancer, pan-tissue analysis capitalizing on large repositories of gene expression data, and show that the seven APOBEC3 genes can be distinguished both by their expression patterns and their co-expression with other genes (Figure 8). Our ‘functional barcodes’ effectively highlight the striking functional differences of the APOBEC3 genes. We have made these available online as a R shiny application, where users can browse interactively the gene annotation data, and download the underlying data tables for further analyses (via http://fraternalilab.kcl.ac.uk/apobec-barcodes/).

The major finding of this study is that not all the APOBEC3 genes associate with immune cells and immune-related functions in the same way, but instead correlate in varying degrees with both immune and proliferative processes in cancer (Figures 3 and 6). This is surprising, considering the close homology of the APOBEC3 deaminases (13,55). All seven APOBEC3 members are capable of hypermutating cytosine bases on single-stranded DNA, and even bind to the same DNA sequence (except for A3G, where the substrate preference is only slightly different at the 5’ end) (14–17). The transcriptional diversification of the human APOBEC3 family which we demonstrate here suggests that although the multiple APOBEC3 genes encode very similar enzymes, it is their expression and co-expression patterns that differentiate their role in immune versus proliferative processes. This finding is made possible by our RESPECTEx pipeline, which integrates tumour purity estimates in attributing cell-type-specific gene expression levels. Tumour heterogeneity has been shown to be an important factor to consider in the detection of differential gene expression (56), the discovery of expression quantitative trait loci (eQTL) (57), and gene network mining (58) in cancer. We believe RESPECTEx can be integrated into the routine assessments of tumour purity in cancer RNA-seq analyses, thereby helping derive new biological insights from the analysis of such transcriptomic datasets.

Another observation we have made, which is consistent across datasets, is that *A3B* is associated with different biological processes than the other APOBEC3 genes. In all our analyses *A3B* consistently demonstrates its association with proliferative cells and processes, in contrast to other APOBEC3s, especially *A3G* and *A3H*, which are revealed in our analysis as more immune cell related (Figure 6), congruent with knowledge about their expression patterns in these cells (18,19). Taken as a whole, these findings suggest a unique role of A3B in cancer through an inherent involvement in the cell cycle and DDR processes, and reinforce previous reports which implicate A3B as the causative agent for mutations in cancers (5,10,59,60). Our analysis adds to this by providing insights into the molecular mechanism, by extracting genes and pathways that may functionally cooperate to cause A3B mutagenesis in cancer. A comprehensive mapping of transcription factor binding sites of the APOBEC3 genes and their co-expressing genes could further our understanding towards the mechanistic bases of such gene co-expression that we have observed. It has been shown that the transcription of the APOBEC3 family is controlled by p53 (61), and that the recruitment of the DREAM complex downstream of p53 to the *A3B* gene promoter controls its expression and explains its cell-cycle-dependent expression pattern (62). However, other APOBEC3s which can enter the nucleus, e.g. A3A (63), might also contribute to mutagenesis in the cancer genome. IFNα is known to potently activate APOBEC3 gene expression, particularly *A3A* (19). In tumours, where interferon signalling is deregulated and modulatory to the growth and survival of cancer cells (64–66), the relevance of IFNα to APOBEC3 expression in tumour tissues awaits further studies. It has been shown that p53 modulates the effect of type-I interferon on APOBEC3 expression (61). This suggests one way in which the expression of APOBEC3 genes could possibly be altered in cancer, where both *TP53* mutations and inflammation are common. A possible example is *A3H*: it has been identified as p53-responsive (28,67–69). Here we observed immune-related gene co-expression (Figure 6) for *A3H*, but it was correlated more with cell cycle/DDR genes in the affected tumour types with elevated APOBEC3-signature mutations (Supplementary Figure S19). The dual perspective of analysing gene expression and co-expression suggests highlights distinctive characteristics of *A3C*, which is expressed highly across tissue and cancer types (Figure 1). *A3C*, however, is evidently immune-related when we analysed the association of the change of its expression with respect to cell type composition (Figures 2 and 3), which is confirmed by the immune-response functional barcodes (Figure 7B and C; Supplementary Figure S12) we observe across tissue/cancer types. A more holistic understanding of the regulation of APOBEC3 expression will explain the multifaceted roles of this gene family in both proliferative and antiviral contexts, and the relationships between APOBEC3-mediated mutational signatures, immune infiltration and tumour progression (70,71).

Our analyses have been partially limited by the nature of the data and the samples. Firstly, the interpretation of the cancer cell line data has been challenging, as the co-expression data on cancer cell line cohorts are not reflective of the TCGA datasets (Supplementary Figures S13–S17). We examined the variations in mRNA expression levels of genes, classified by their functions in cancer cell lines and tumours, and found extensive differences in terms of the extent of such variations between these two types of samples (Supplementary Figure S21). This could impact on the extraction of expression correlations. Nevertheless, the single-cell RNA-seq data of tumours (Figure 4C; Supplementary Figure S8) reveals that different cell types, immune or proliferative, express different sets of APOBEC3 genes. Secondly, some of our analyses were limited by the size of cohorts. For instance, the accuracy of reconstituted expression obtained from the RESPECTEx pipeline, when applied to a small cohort (e.g. in Figure 4D where we applied to the breast cancer cohort (20) with *n* = 8 tumours), appears to vary from one cell type to the other (Supplementary Figure S9). We suggest that the RESPECTEx deconvolution reflects the expression landscape of the seven APOBEC3 genes in the tumour cell admixture, as when taken together with our correlation analysis of tumour bulk gene expression with estimates of tumour purity (Figure 2) and immune cell levels (Figure 3), they all support the same trend of diversification in terms of their cell-type specificity of expression. This shows the importance of interpreting these results together with other analyses. Thirdly, one might question whether the cell cycle/DDR functional enrichment of *A3B* co-expressing genes that we describe is the cause or the consequence of its activation. While the cross-sectional nature of our RNA-seq data poses limitations for this type of analysis, our results suggest a G2/M-enriched gene co-expression signature for *A3B* in tumours (Figure 6B; Supplementary Figures S12, S15). This is consistent with a recent report on the effects of *A3B* overexpression in a cancer cell line (59), where an extensive G2/M arrest of the cells was observed. Further experimental and theoretical investigations could be directed to verify whether the A3B protein is activated in specific phase(s) of the cell cycle, and the mechanism behind the role of this enzyme in targeting of genomic regions to perform mutagenesis.

To conclude, we have presented results using our pan-cancer analysis pipeline to delineate cell type specificity of APOBEC3 gene expression in a tumour cell mixture, by examining gene expression and co-expression data and their correlations with inferred cell type composition of tumour samples. This analysis deeply annotates an additional level of biological information, the transcriptome, adding to our functional understanding of the APOBEC3 family. By estimating immune cell proportions using CIBERSORT (31) coupled with RESPECTEx, we have addressed the well-recognized issue of tumours as admixtures of cancerous and infiltrated cells, and attributed cell-type-specific expression to different cell populations in tumours. Using curated gene sets and annotating gene co-expression, we developed analyses and visualization tools to functionally ‘barcode’ gene co-expression data. The approach developed here can be applied more broadly to the analysis of cell-type-specific gene expression and gene function, and such an approach can assist cancer biologists in prioritizing gene targets to be investigated, in a biological context appropriate to the cell types in which these genes are likely to be expressed.

## DATA AVAILABILITY

All data generated from these analyses are included in the Supplementary Data accompanying this paper. The code for RESPECTEx, along with test input and expected output (based on the GSE75688 breast cancer cohort, cf. Figure 4), is available on http://github.com/fraternalilab/RESPECTEx. We have produced an applet using R shiny (http://shiny.rstudio.com) (v1.0.5), specifically designed to visualize the functional barcode visualizations, for both the co-expressing gene analysis (Figure 6B) and the distinguishing gene analysis (Figure 7D). This is accessible via http://fraternalilab.kcl.ac.uk/apobec-barcodes/. Users can view these visualizations, hover over the barcodes to view interactively the genes annotated in the barcodes for each gene set and each data cohort we have examined, and download the underlying data tables for their own further investigations.

## Supplementary Material

Supplementary Data
