# Supplementary material for: Pan-cancer transcriptomic analysis dissects immune and proliferative functions of APOBEC3 cytidine deaminases
Source: Nucleic Acids Res. 2019 Jan 9;47(3):1178–94. doi: 10.1093/nar/gky1316 (PMC6379723; doi:10.1093/nar/gky1316)
Supplement: Supplementary Data [file gky1316_supplemental_files.zip › FigureS12_FunctionalBarcodes.pdf]

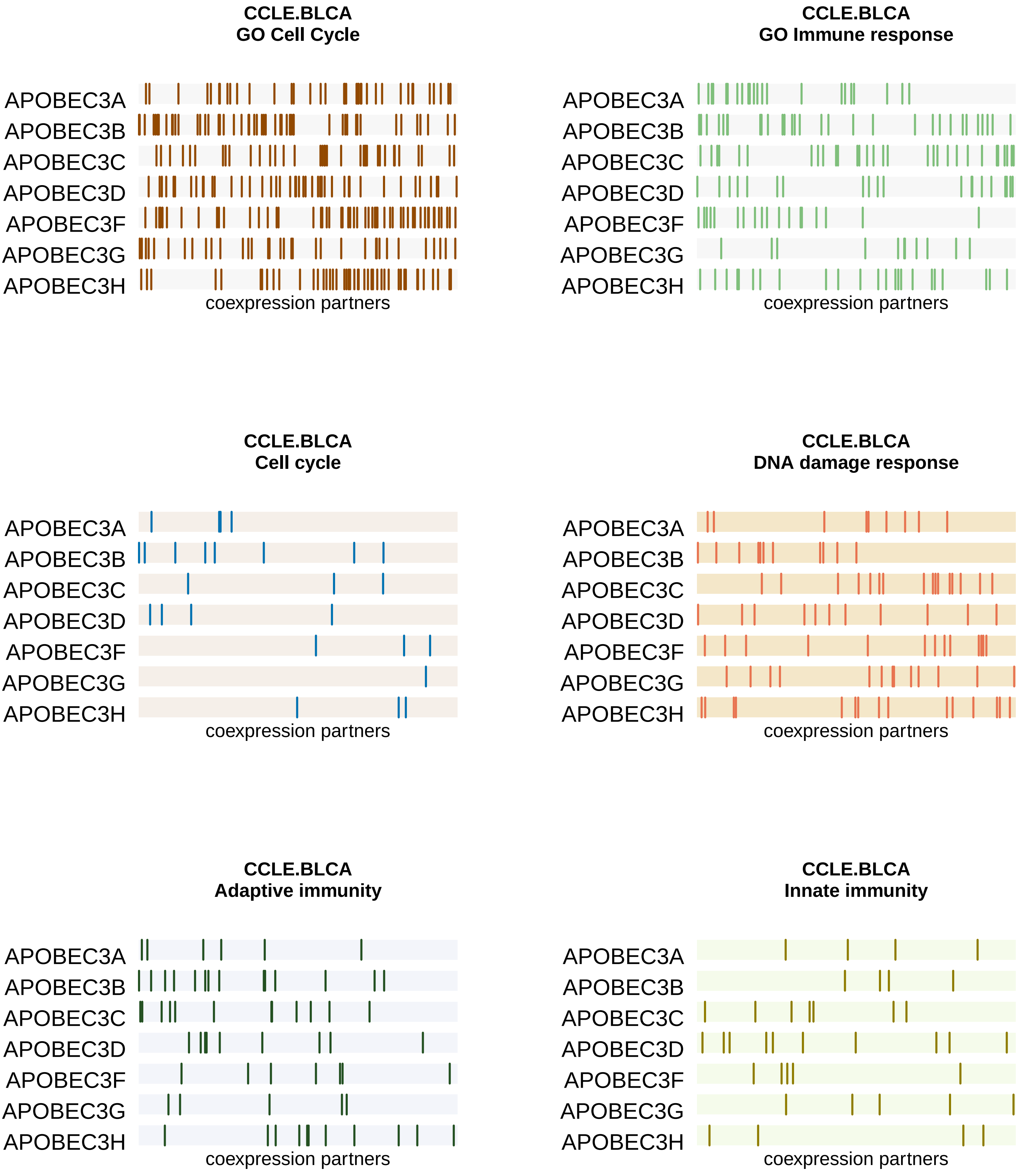
CCLE.BLCA
GO Cell Cycle
APOBEC3A
APOBEC3B
APOBEC3C
APOBEC3D
APOBEC3F
APOBEC3G
APOBEC3H
coexpression partners
CCLE.BLCA
GO Immune response
APOBEC3A
APOBEC3B
APOBEC3C
APOBEC3D
APOBEC3F
APOBEC3G
APOBEC3H
coexpression partners
CCLE.BLCA
Cell cycle
APOBEC3A
APOBEC3B
APOBEC3C
APOBEC3D
APOBEC3F
APOBEC3G
APOBEC3H
coexpression partners
CCLE.BLCA
DNA damage response
APOBEC3A
APOBEC3B
APOBEC3C
APOBEC3D
APOBEC3F
APOBEC3G
APOBEC3H
coexpression partners
CCLE.BLCA
Adaptive immunity
APOBEC3A
APOBEC3B
APOBEC3C
APOBEC3D
APOBEC3F
APOBEC3G
APOBEC3H
coexpression partners
CCLE.BLCA
Innate immunity
APOBEC3A
APOBEC3B
APOBEC3C
APOBEC3D
APOBEC3F
APOBEC3G
APOBEC3H
coexpression partners

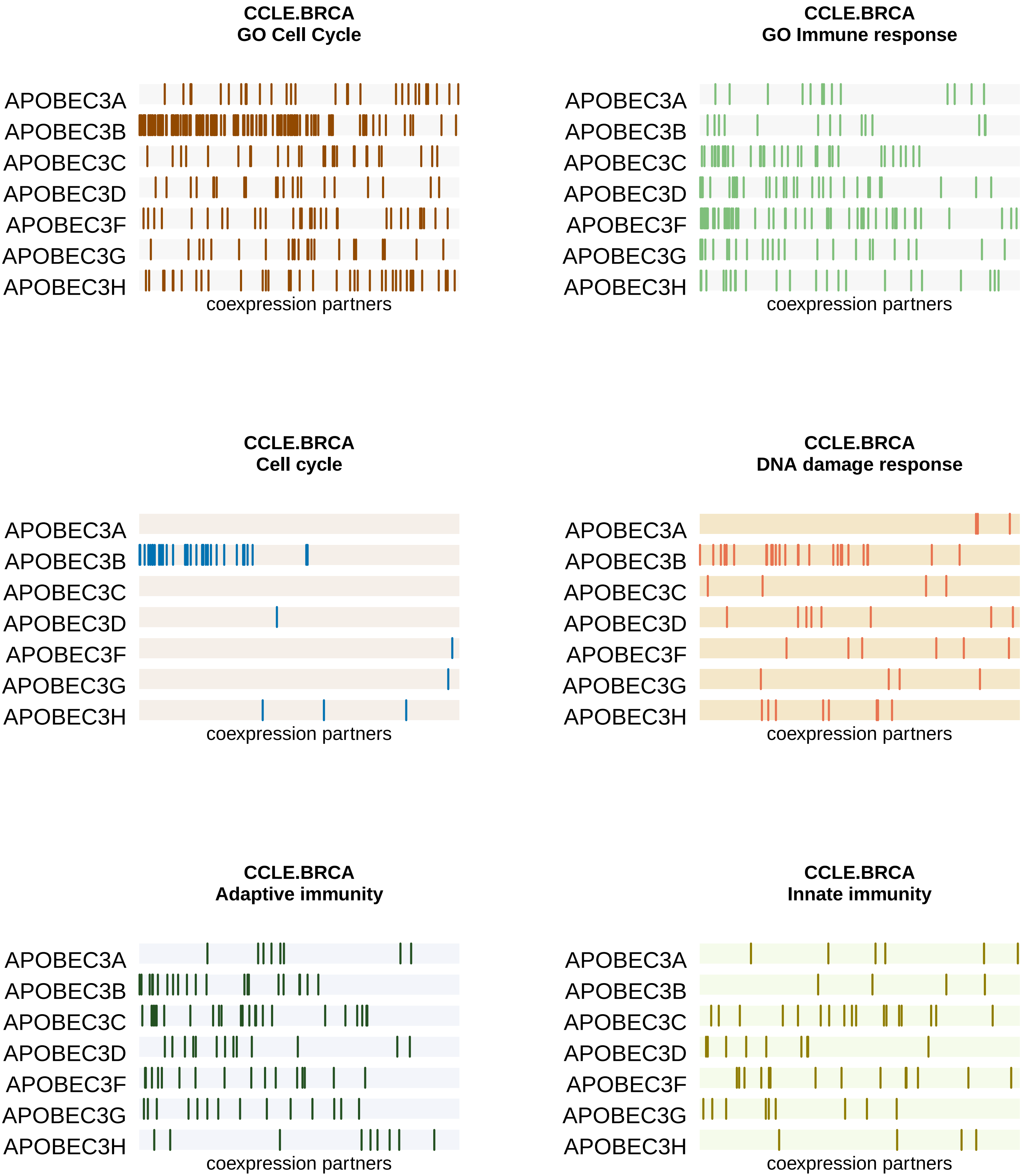
CCLE.BRCA
GO Cell Cycle
CCLE.BRCA
GO Immune response
CCLE.BRCA
Cell cycle
CCLE.BRCA
DNA damage response
CCLE.BRCA
Adaptive immunity
CCLE.BRCA
Innate immunity
APOBEC3A
APOBEC3B
APOBEC3C
APOBEC3D
APOBEC3F
APOBEC3G
APOBEC3H
coexpression partners

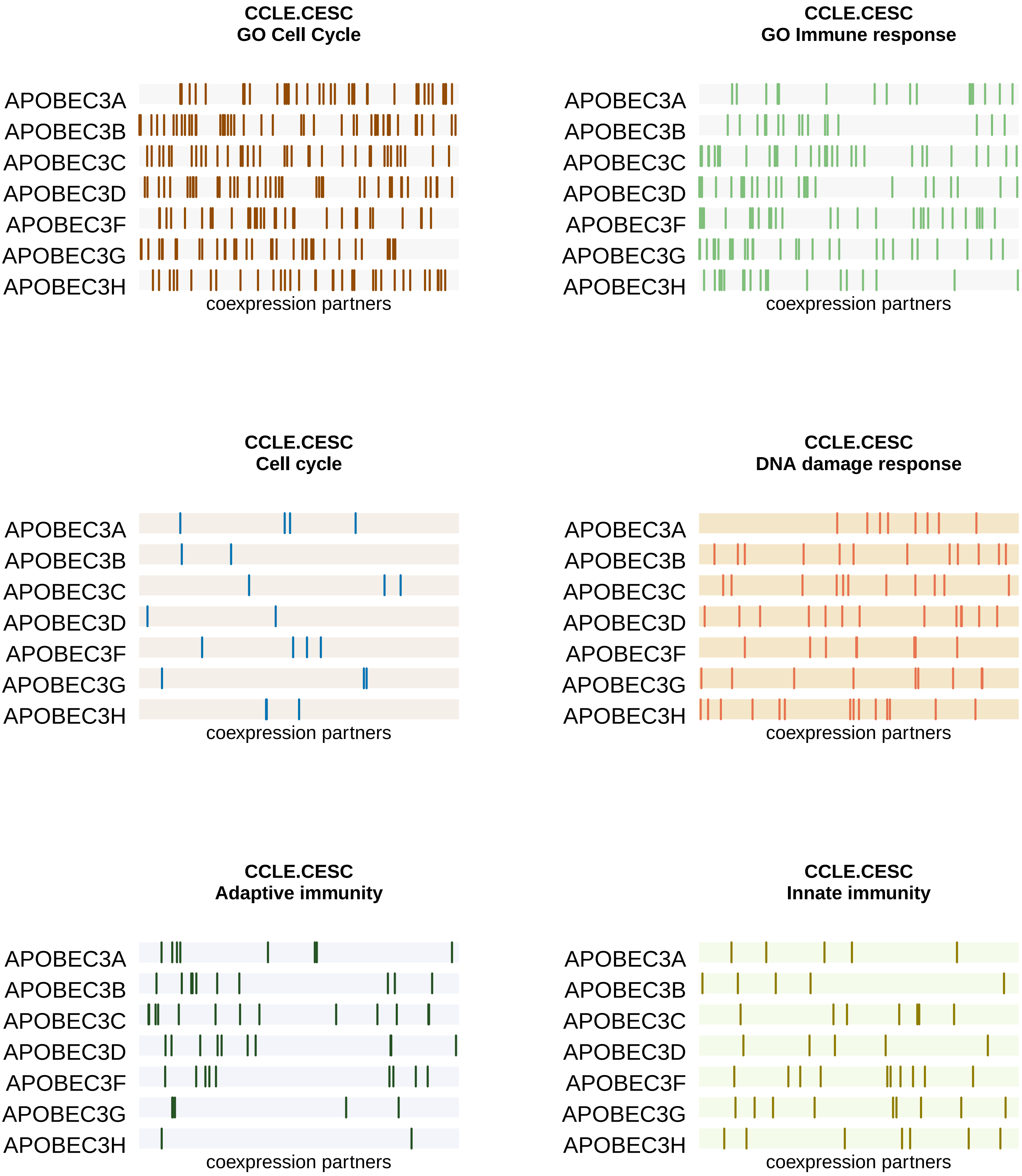
CCLE.CESC
GO Cell Cycle
CCLE.CESC
GO Immune response
CCLE.CESC
Cell cycle
CCLE.CESC
DNA damage response
CCLE.CESC
Adaptive immunity
CCLE.CESC
Innate immunity
APOBEC3A
APOBEC3B
APOBEC3C
APOBEC3D
APOBEC3F
APOBEC3G
APOBEC3H
coexpression partners

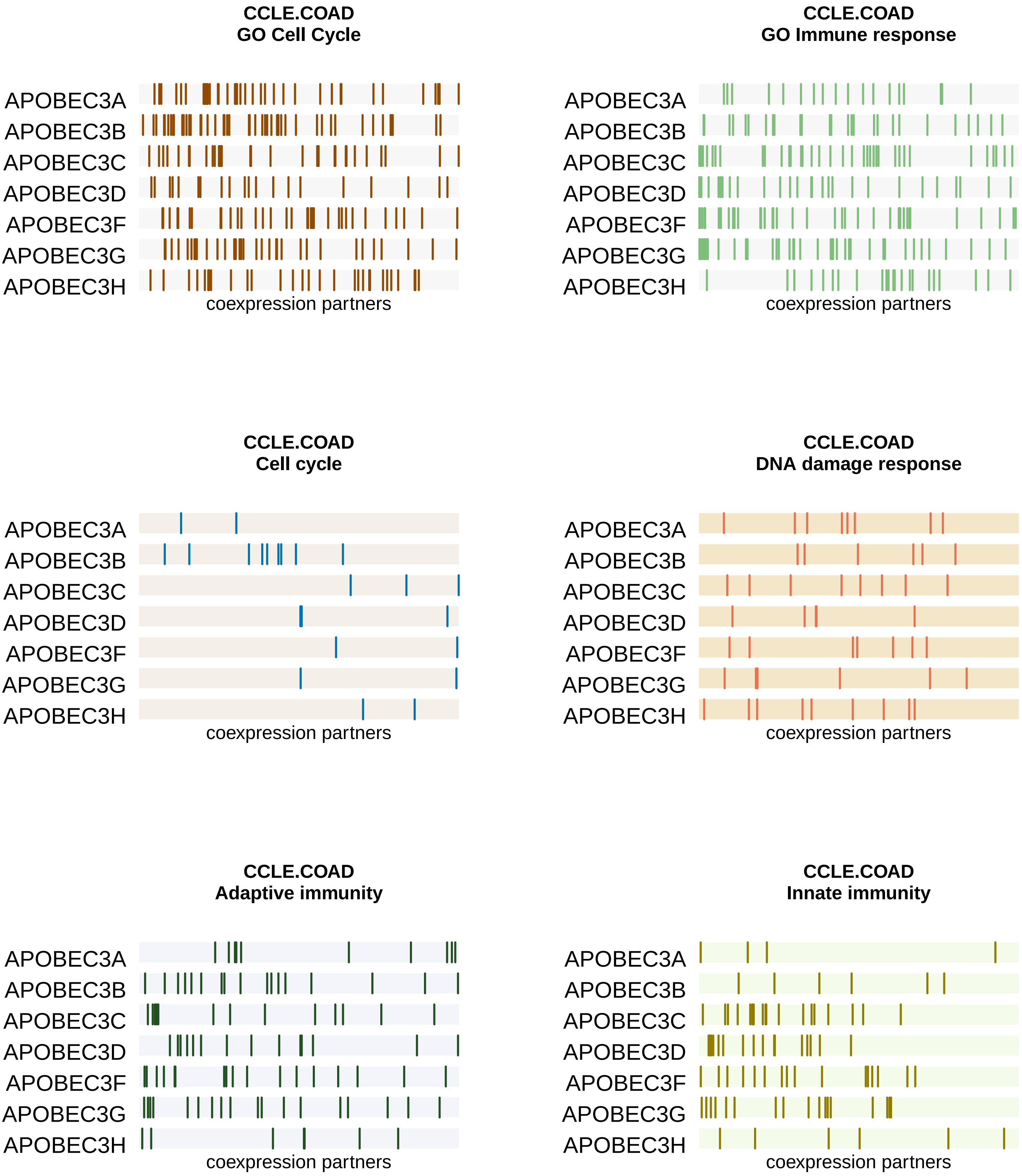
CCLE.COAD
GO Cell Cycle
APOBEC3A
APOBEC3B
APOBEC3C
APOBEC3D
APOBEC3F
APOBEC3G
APOBEC3H
coexpression partners
CCLE.COAD
GO Immune response
APOBEC3A
APOBEC3B
APOBEC3C
APOBEC3D
APOBEC3F
APOBEC3G
APOBEC3H
coexpression partners
CCLE.COAD
Cell cycle
APOBEC3A
APOBEC3B
APOBEC3C
APOBEC3D
APOBEC3F
APOBEC3G
APOBEC3H
coexpression partners
CCLE.COAD
DNA damage response
APOBEC3A
APOBEC3B
APOBEC3C
APOBEC3D
APOBEC3F
APOBEC3G
APOBEC3H
coexpression partners
CCLE.COAD
Adaptive immunity
APOBEC3A
APOBEC3B
APOBEC3C
APOBEC3D
APOBEC3F
APOBEC3G
APOBEC3H
coexpression partners
CCLE.COAD
Innate immunity
APOBEC3A
APOBEC3B
APOBEC3C
APOBEC3D
APOBEC3F
APOBEC3G
APOBEC3H
coexpression partners

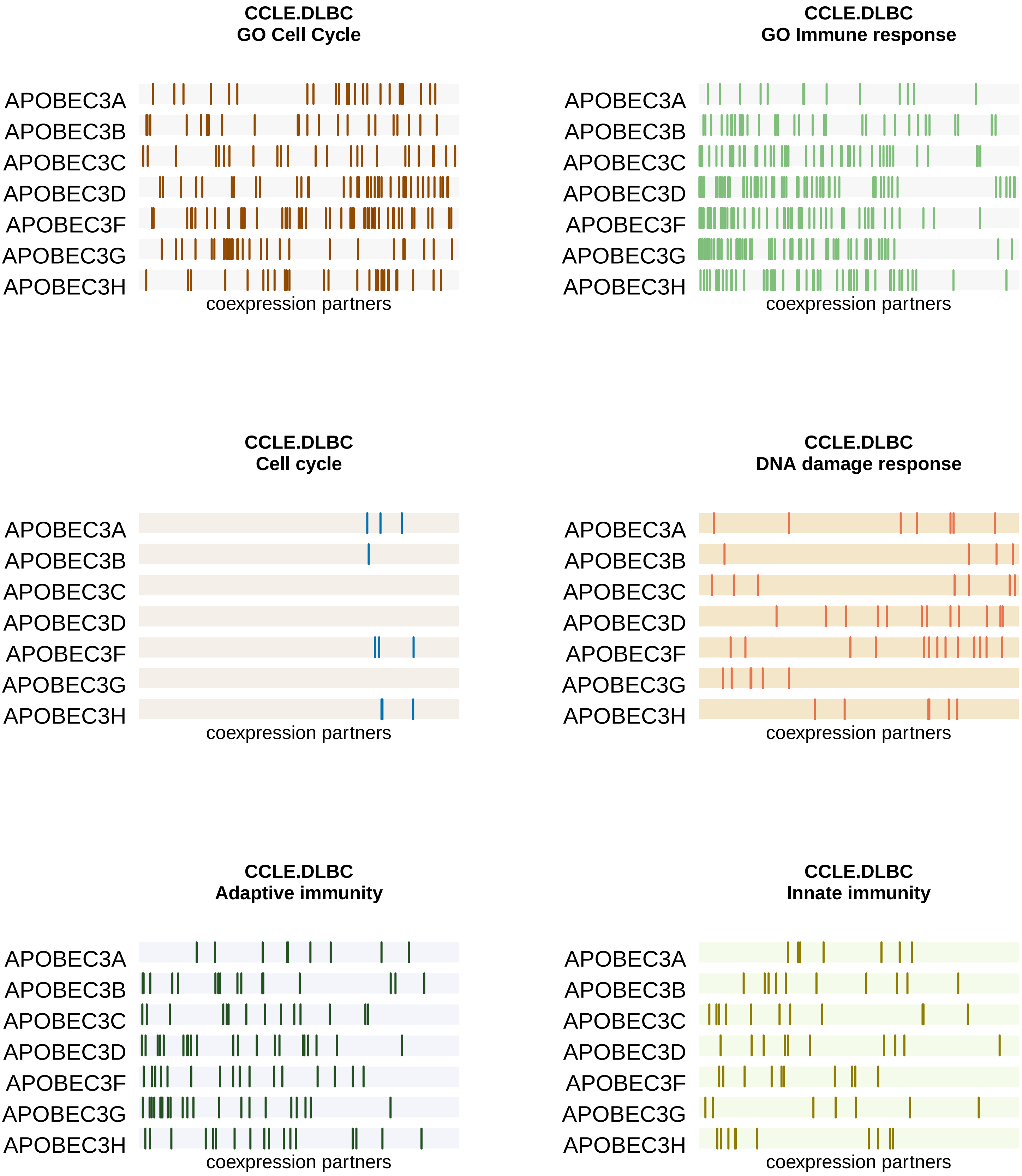
CCLE.DLBC
GO Cell Cycle
CCLE.DLBC
GO Immune response
CCLE.DLBC
Cell cycle
CCLE.DLBC
DNA damage response
CCLE.DLBC
Adaptive immunity
CCLE.DLBC
Innate immunity
APOBEC3A
APOBEC3B
APOBEC3C
APOBEC3D
APOBEC3F
APOBEC3G
APOBEC3H
coexpression partners

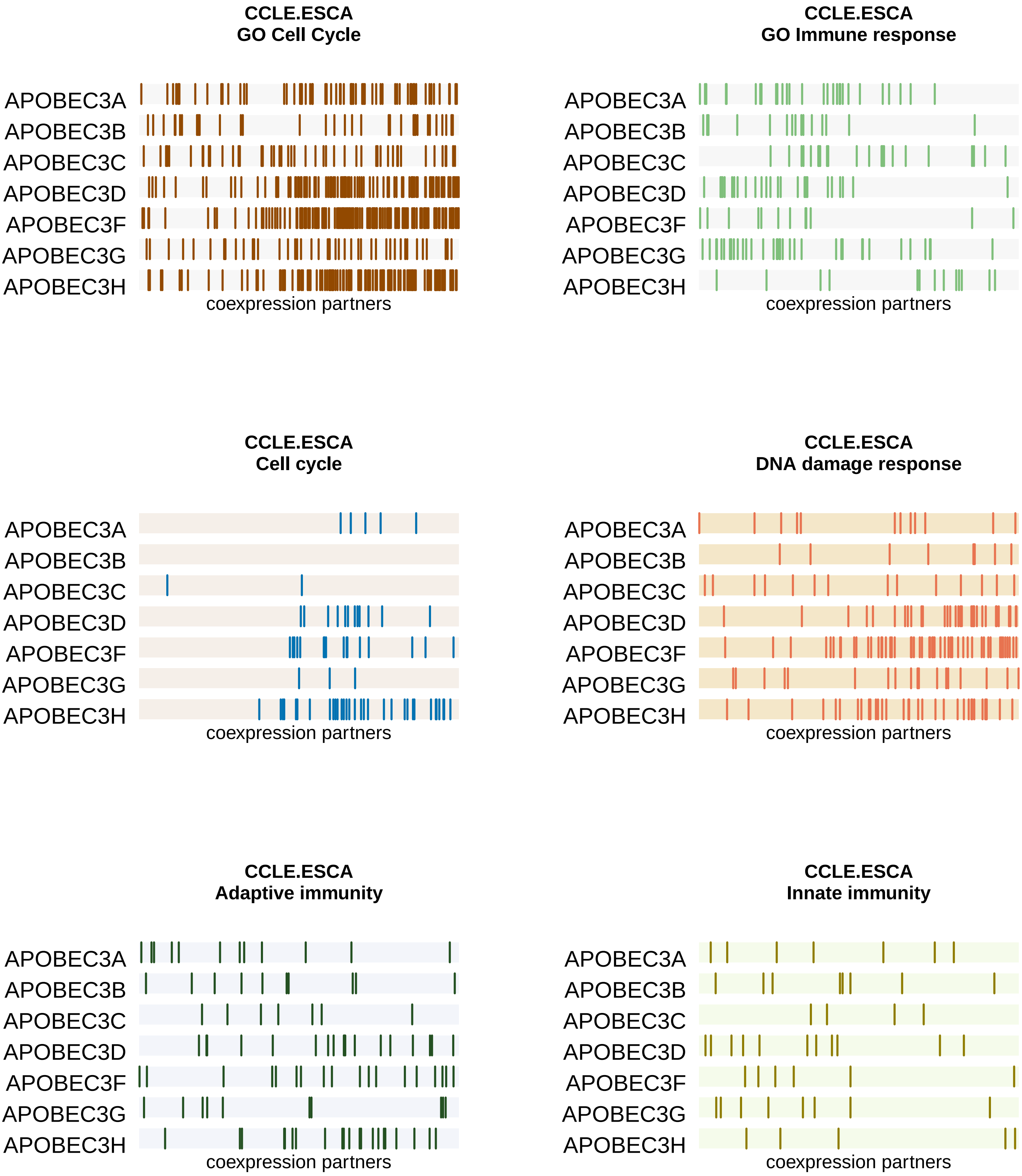
CCLE.ESCA
GO Cell Cycle
APOBEC3A
APOBEC3B
APOBEC3C
APOBEC3D
APOBEC3F
APOBEC3G
APOBEC3H
coexpression partners
CCLE.ESCA
GO Immune response
APOBEC3A
APOBEC3B
APOBEC3C
APOBEC3D
APOBEC3F
APOBEC3G
APOBEC3H
coexpression partners
CCLE.ESCA
Cell cycle
APOBEC3A
APOBEC3B
APOBEC3C
APOBEC3D
APOBEC3F
APOBEC3G
APOBEC3H
coexpression partners
CCLE.ESCA
DNA damage response
APOBEC3A
APOBEC3B
APOBEC3C
APOBEC3D
APOBEC3F
APOBEC3G
APOBEC3H
coexpression partners
CCLE.ESCA
Adaptive immunity
APOBEC3A
APOBEC3B
APOBEC3C
APOBEC3D
APOBEC3F
APOBEC3G
APOBEC3H
coexpression partners
CCLE.ESCA
Innate immunity
APOBEC3A
APOBEC3B
APOBEC3C
APOBEC3D
APOBEC3F
APOBEC3G
APOBEC3H
coexpression partners

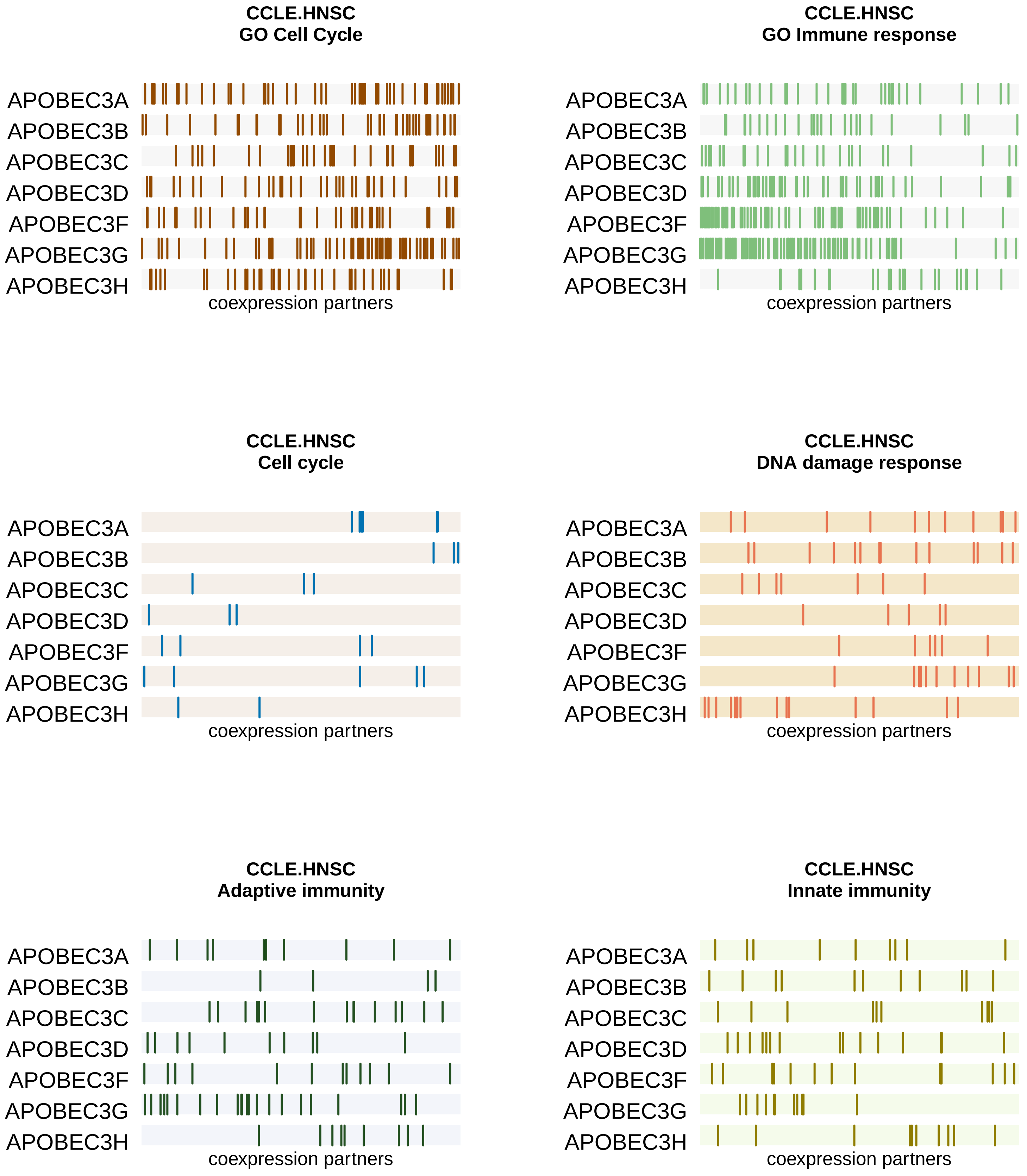
CCLE.HNSC
GO Cell Cycle
APOBEC3A
APOBEC3B
APOBEC3C
APOBEC3D
APOBEC3F
APOBEC3G
APOBEC3H
coexpression partners
CCLE.HNSC
GO Immune response
APOBEC3A
APOBEC3B
APOBEC3C
APOBEC3D
APOBEC3F
APOBEC3G
APOBEC3H
coexpression partners
CCLE.HNSC
Cell cycle
APOBEC3A
APOBEC3B
APOBEC3C
APOBEC3D
APOBEC3F
APOBEC3G
APOBEC3H
coexpression partners
CCLE.HNSC
DNA damage response
APOBEC3A
APOBEC3B
APOBEC3C
APOBEC3D
APOBEC3F
APOBEC3G
APOBEC3H
coexpression partners
CCLE.HNSC
Adaptive immunity
APOBEC3A
APOBEC3B
APOBEC3C
APOBEC3D
APOBEC3F
APOBEC3G
APOBEC3H
coexpression partners
CCLE.HNSC
Innate immunity
APOBEC3A
APOBEC3B
APOBEC3C
APOBEC3D
APOBEC3F
APOBEC3G
APOBEC3H
coexpression partners

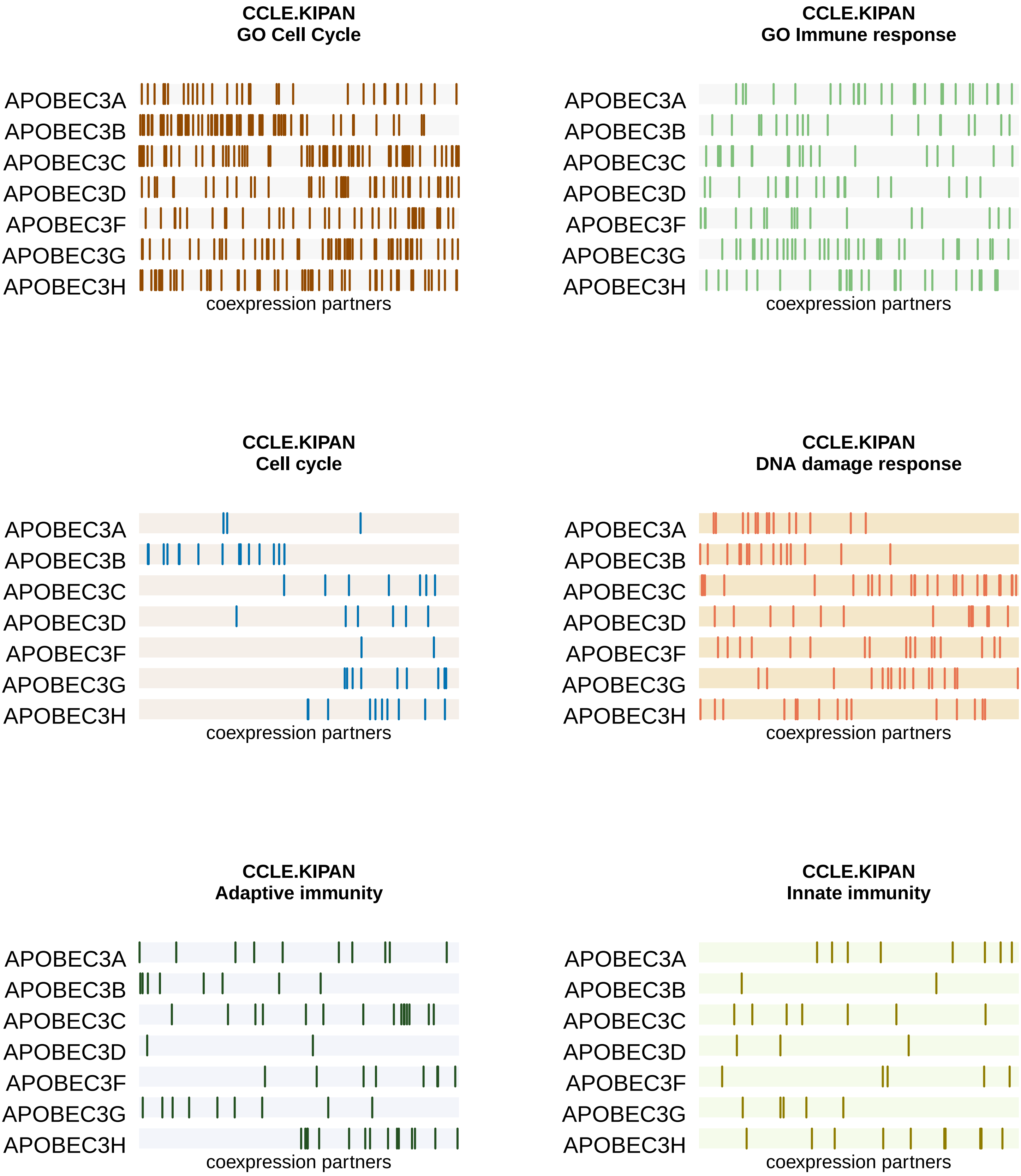
CCLE.KIPAN
GO Cell Cycle
CCLE.KIPAN
GO Immune response
CCLE.KIPAN
Cell cycle
CCLE.KIPAN
DNA damage response
CCLE.KIPAN
Adaptive immunity
CCLE.KIPAN
Innate immunity
APOBEC3A
APOBEC3B
APOBEC3C
APOBEC3D
APOBEC3F
APOBEC3G
APOBEC3H
coexpression partners

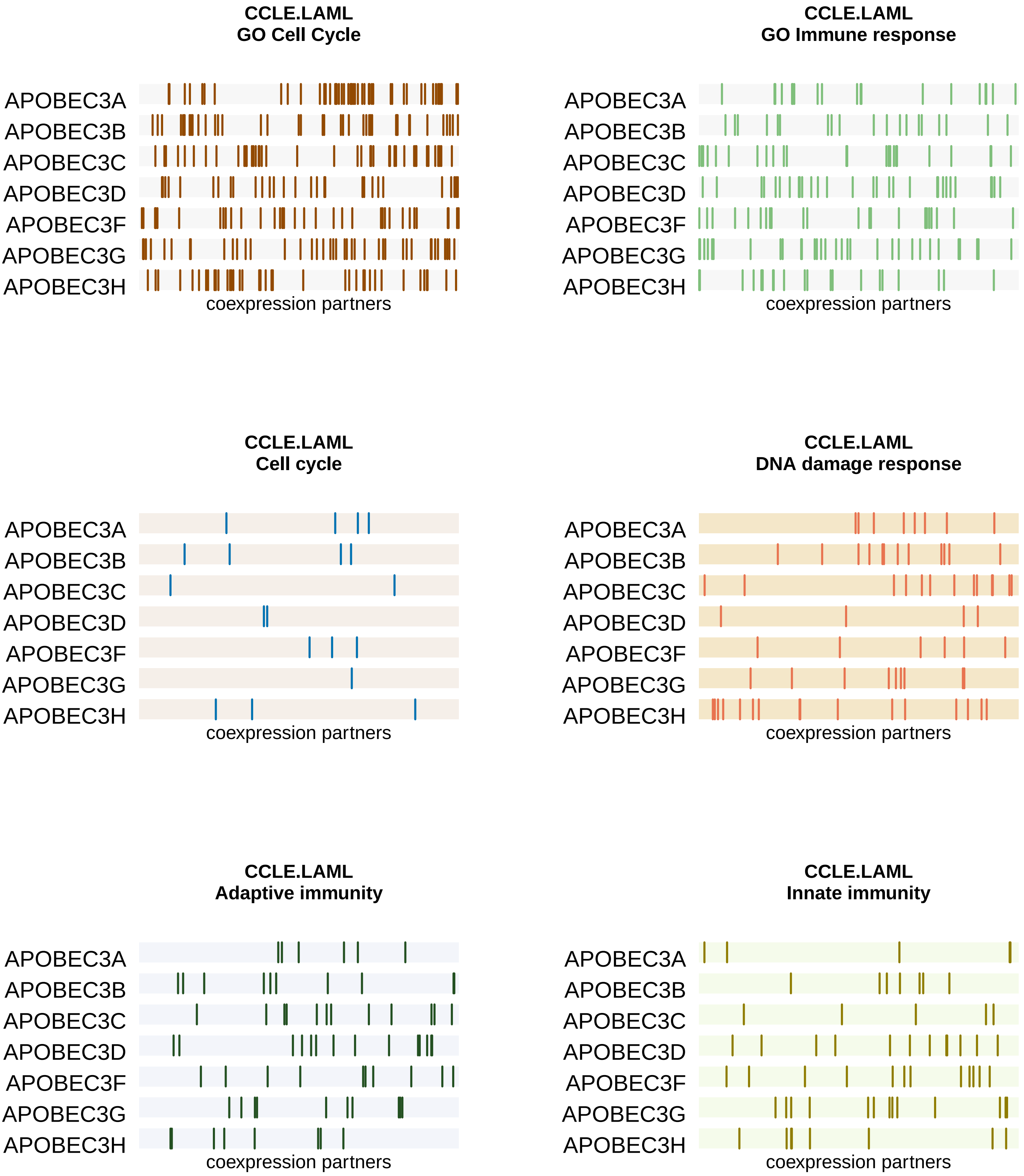

CCLE.LAML
GO Cell Cycle
CCLE.LAML
GO Immune response
CCLE.LAML
Cell cycle
CCLE.LAML
DNA damage response
CCLE.LAML
Adaptive immunity
CCLE.LAML
Innate immunity
APOBEC3A
APOBEC3B
APOBEC3C
APOBEC3D
APOBEC3F
APOBEC3G
APOBEC3H
coexpression partners

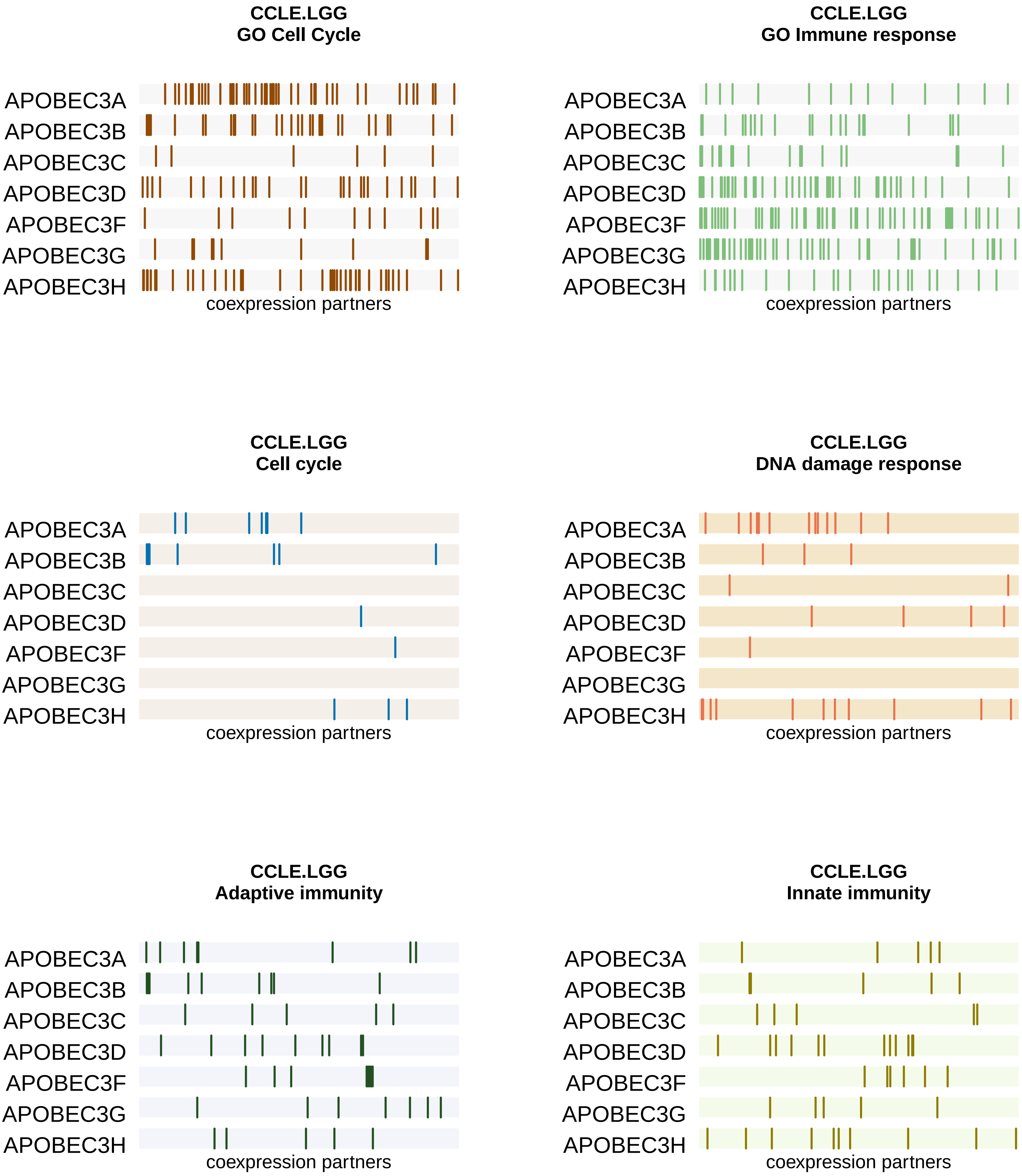
CCLE.LGG
GO Cell Cycle
APOBEC3A
APOBEC3B
APOBEC3C
APOBEC3D
APOBEC3F
APOBEC3G
APOBEC3H
coexpression partners
CCLE.LGG
GO Immune response
APOBEC3A
APOBEC3B
APOBEC3C
APOBEC3D
APOBEC3F
APOBEC3G
APOBEC3H
coexpression partners
CCLE.LGG
Cell cycle
APOBEC3A
APOBEC3B
APOBEC3C
APOBEC3D
APOBEC3F
APOBEC3G
APOBEC3H
coexpression partners
CCLE.LGG
DNA damage response
APOBEC3A
APOBEC3B
APOBEC3C
APOBEC3D
APOBEC3F
APOBEC3G
APOBEC3H
coexpression partners
CCLE.LGG
Adaptive immunity
APOBEC3A
APOBEC3B
APOBEC3C
APOBEC3D
APOBEC3F
APOBEC3G
APOBEC3H
coexpression partners
CCLE.LGG
Innate immunity
APOBEC3A
APOBEC3B
APOBEC3C
APOBEC3D
APOBEC3F
APOBEC3G
APOBEC3H
coexpression partners

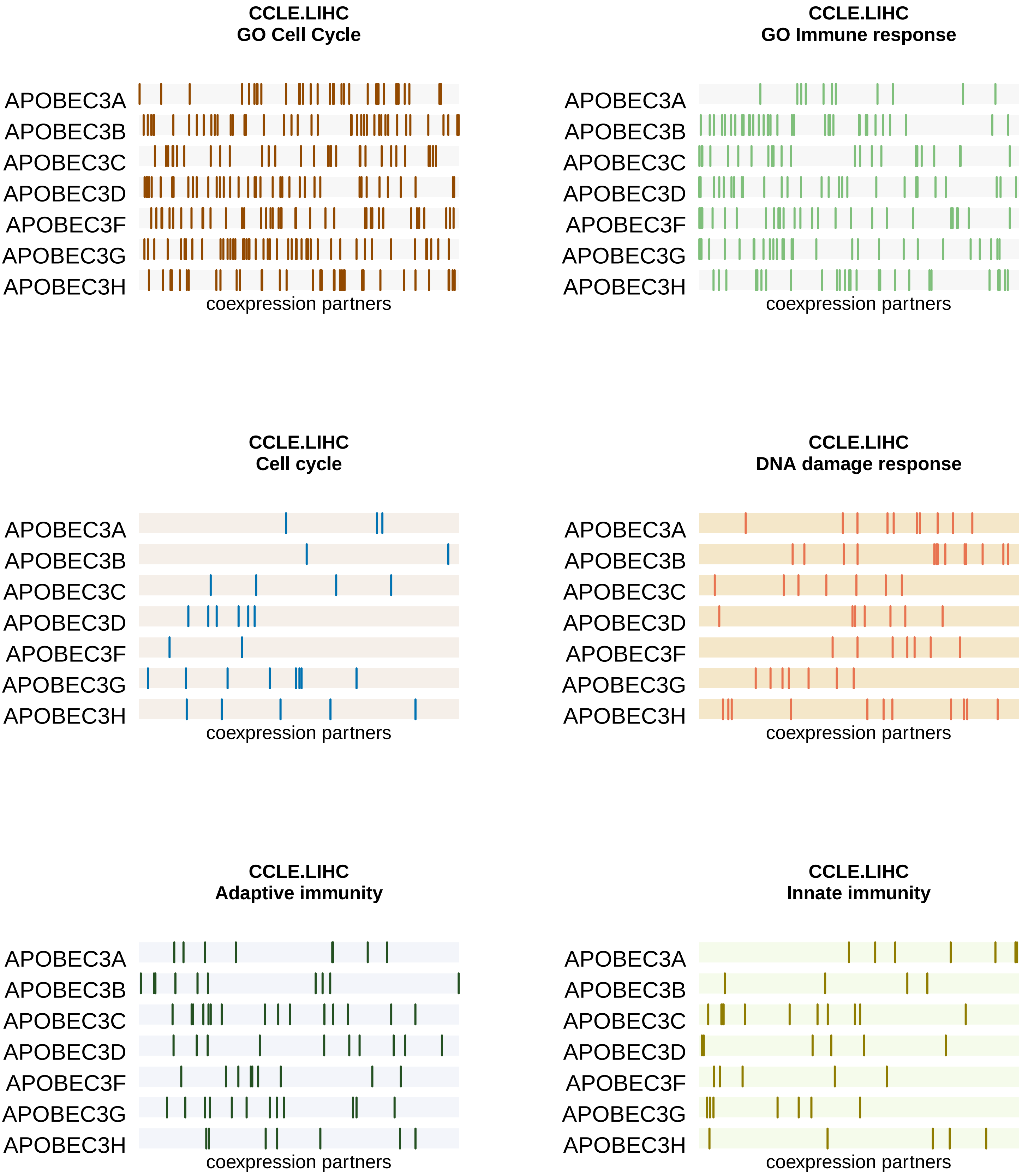
CCLE.LIHC
GO Cell Cycle
APOBEC3A
APOBEC3B
APOBEC3C
APOBEC3D
APOBEC3F
APOBEC3G
APOBEC3H
coexpression partners
CCLE.LIHC
GO Immune response
APOBEC3A
APOBEC3B
APOBEC3C
APOBEC3D
APOBEC3F
APOBEC3G
APOBEC3H
coexpression partners
CCLE.LIHC
Cell cycle
APOBEC3A
APOBEC3B
APOBEC3C
APOBEC3D
APOBEC3F
APOBEC3G
APOBEC3H
coexpression partners
CCLE.LIHC
DNA damage response
APOBEC3A
APOBEC3B
APOBEC3C
APOBEC3D
APOBEC3F
APOBEC3G
APOBEC3H
coexpression partners
CCLE.LIHC
Adaptive immunity
APOBEC3A
APOBEC3B
APOBEC3C
APOBEC3D
APOBEC3F
APOBEC3G
APOBEC3H
coexpression partners
CCLE.LIHC
Innate immunity
APOBEC3A
APOBEC3B
APOBEC3C
APOBEC3D
APOBEC3F
APOBEC3G
APOBEC3H
coexpression partners

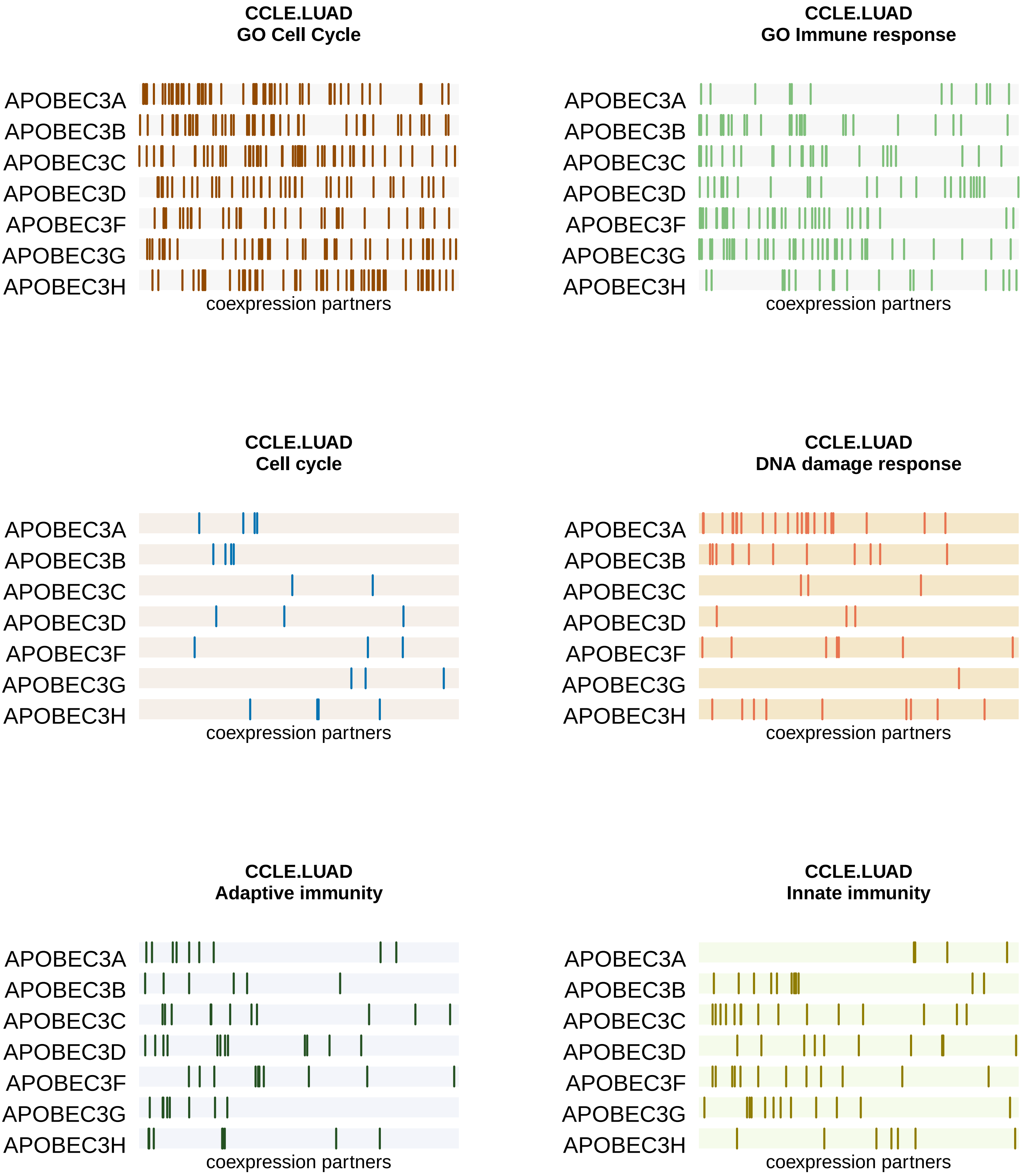
CCLE.LUAD
GO Cell Cycle
CCLE.LUAD
GO Immune response
CCLE.LUAD
Cell cycle
CCLE.LUAD
DNA damage response
CCLE.LUAD
Adaptive immunity
CCLE.LUAD
Innate immunity
APOBEC3A
APOBEC3B
APOBEC3C
APOBEC3D
APOBEC3F
APOBEC3G
APOBEC3H
coexpression partners

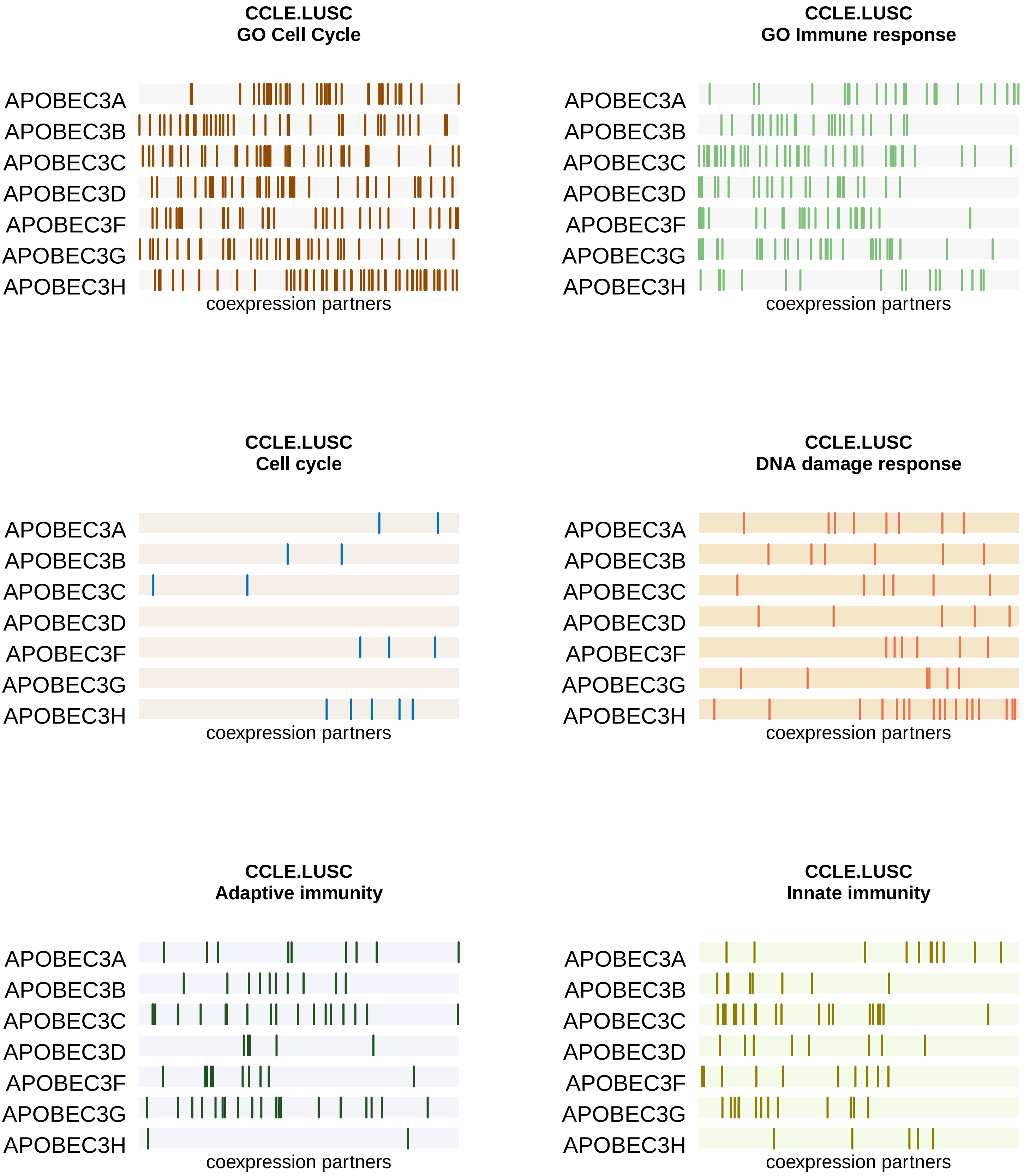
CCLE.LUSC
GO Cell Cycle
APOBEC3A
APOBEC3B
APOBEC3C
APOBEC3D
APOBEC3F
APOBEC3G
APOBEC3H
coexpression partners
CCLE.LUSC
GO Immune response
APOBEC3A
APOBEC3B
APOBEC3C
APOBEC3D
APOBEC3F
APOBEC3G
APOBEC3H
coexpression partners
CCLE.LUSC
Cell cycle
APOBEC3A
APOBEC3B
APOBEC3C
APOBEC3D
APOBEC3F
APOBEC3G
APOBEC3H
coexpression partners
CCLE.LUSC
DNA damage response
APOBEC3A
APOBEC3B
APOBEC3C
APOBEC3D
APOBEC3F
APOBEC3G
APOBEC3H
coexpression partners
CCLE.LUSC
Adaptive immunity
APOBEC3A
APOBEC3B
APOBEC3C
APOBEC3D
APOBEC3F
APOBEC3G
APOBEC3H
coexpression partners
CCLE.LUSC
Innate immunity
APOBEC3A
APOBEC3B
APOBEC3C
APOBEC3D
APOBEC3F
APOBEC3G
APOBEC3H
coexpression partners

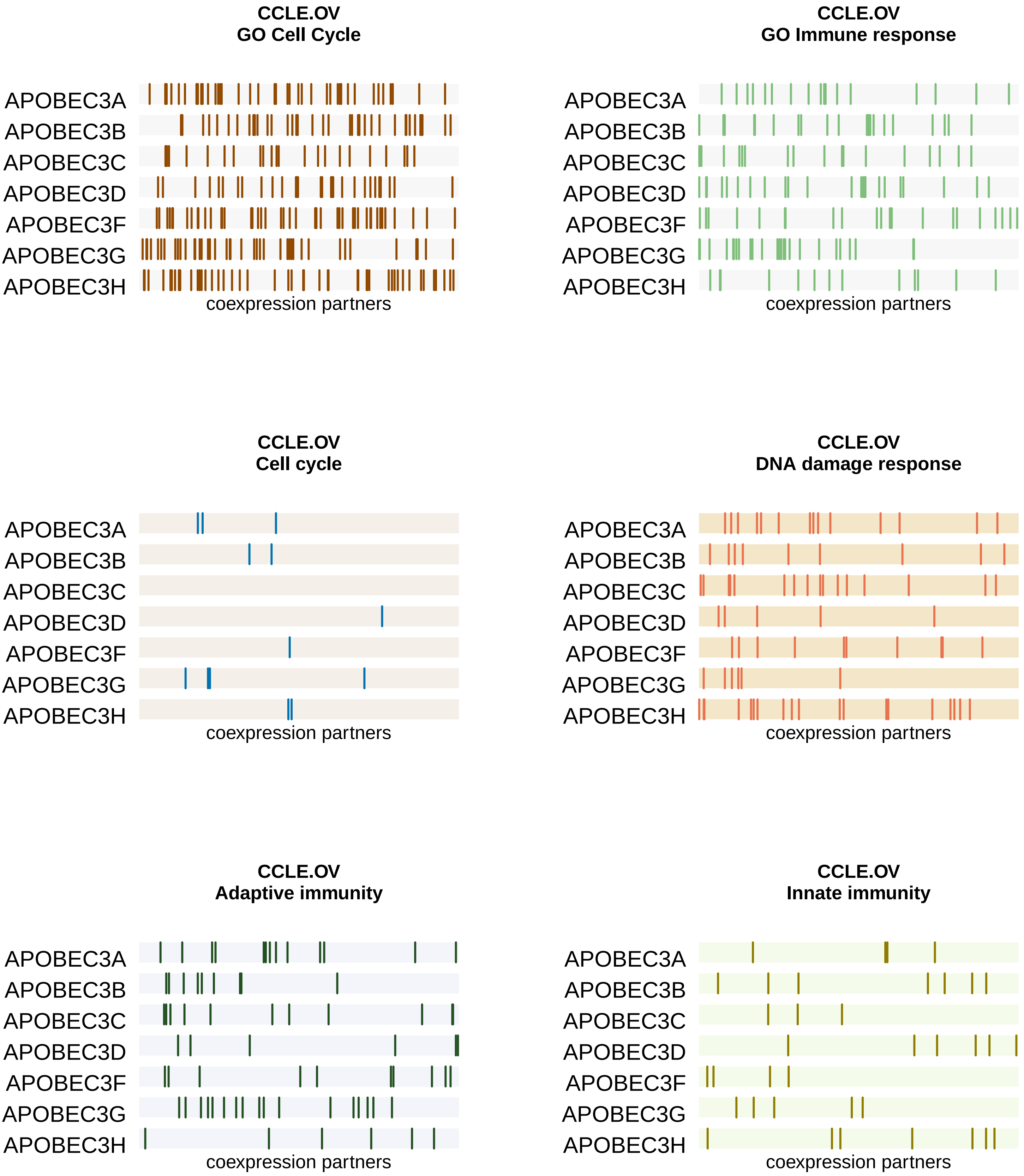
CCLE.OV
GO Cell Cycle
APOBEC3A
APOBEC3B
APOBEC3C
APOBEC3D
APOBEC3F
APOBEC3G
APOBEC3H
coexpression partners
CCLE.OV
GO Immune response
APOBEC3A
APOBEC3B
APOBEC3C
APOBEC3D
APOBEC3F
APOBEC3G
APOBEC3H
coexpression partners
CCLE.OV
Cell cycle
APOBEC3A
APOBEC3B
APOBEC3C
APOBEC3D
APOBEC3F
APOBEC3G
APOBEC3H
coexpression partners
CCLE.OV
DNA damage response
APOBEC3A
APOBEC3B
APOBEC3C
APOBEC3D
APOBEC3F
APOBEC3G
APOBEC3H
coexpression partners
CCLE.OV
Adaptive immunity
APOBEC3A
APOBEC3B
APOBEC3C
APOBEC3D
APOBEC3F
APOBEC3G
APOBEC3H
coexpression partners
CCLE.OV
Innate immunity
APOBEC3A
APOBEC3B
APOBEC3C
APOBEC3D
APOBEC3F
APOBEC3G
APOBEC3H
coexpression partners

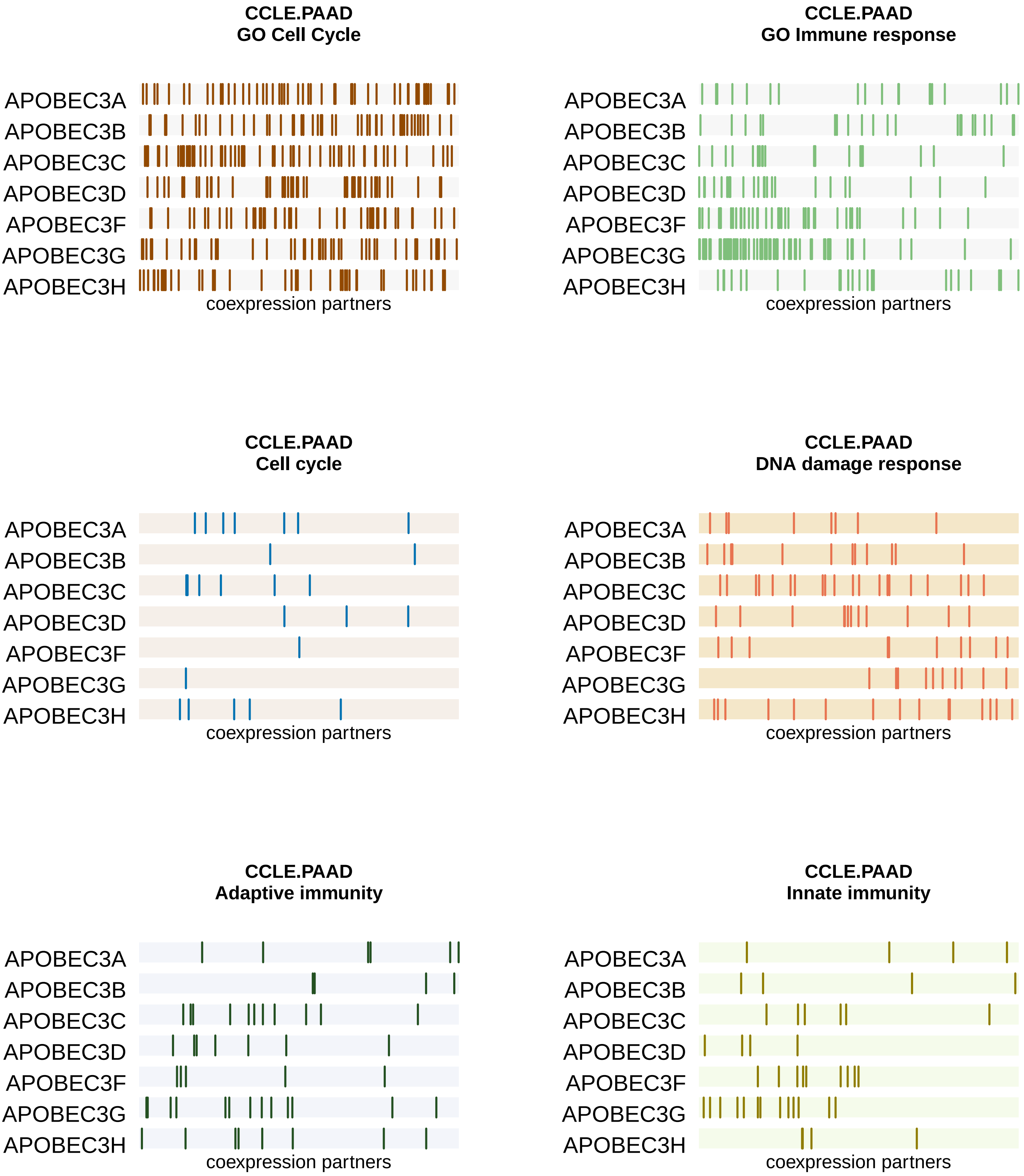
CCLE.PAAD
GO Cell Cycle
CCLE.PAAD
GO Immune response
CCLE.PAAD
Cell cycle
CCLE.PAAD
DNA damage response
CCLE.PAAD
Adaptive immunity
CCLE.PAAD
Innate immunity
APOBEC3A
APOBEC3B
APOBEC3C
APOBEC3D
APOBEC3F
APOBEC3G
APOBEC3H
coexpression partners

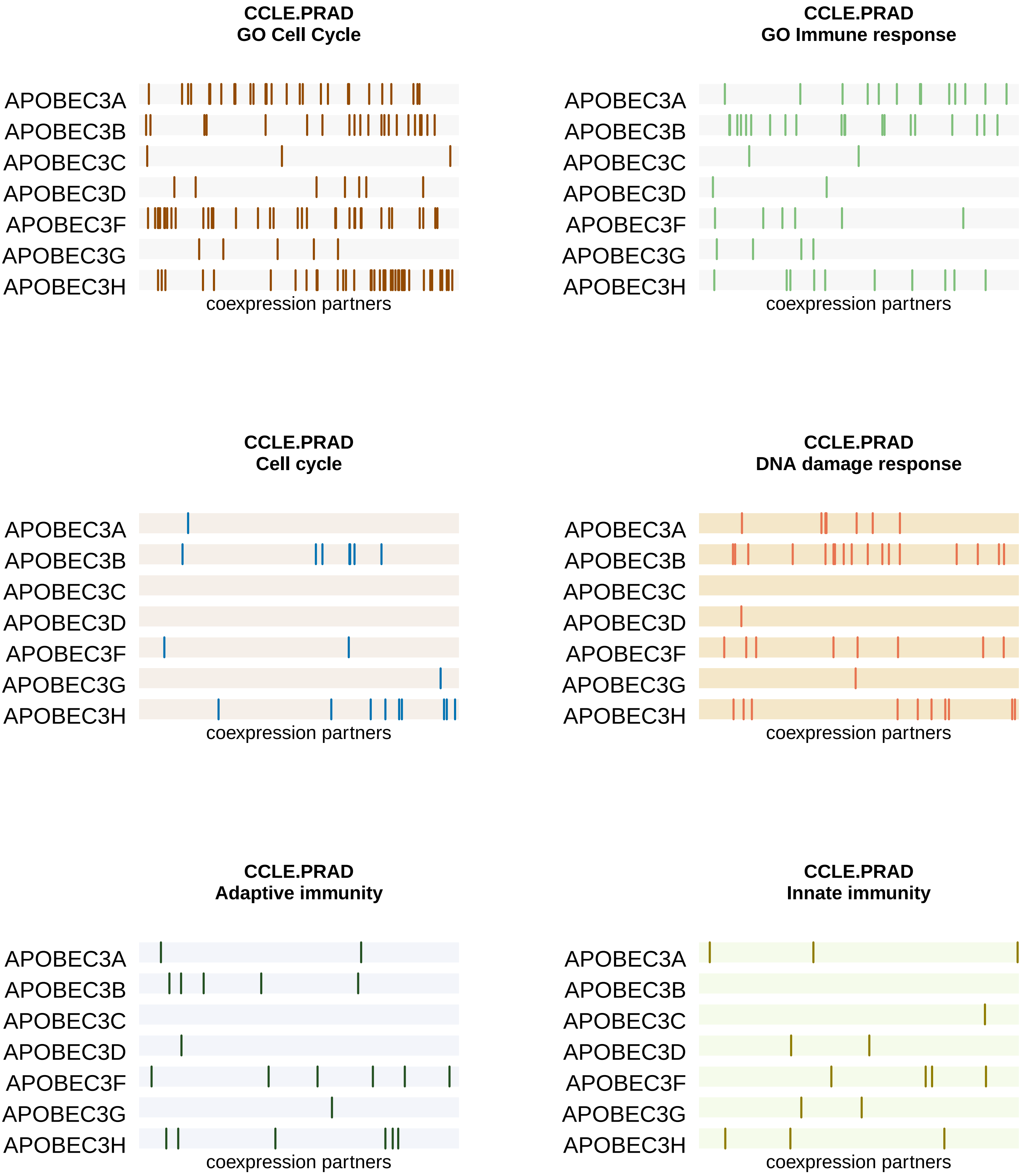
CCLE.PRAD
GO Cell Cycle
CCLE.PRAD
GO Immune response
CCLE.PRAD
Cell cycle
CCLE.PRAD
DNA damage response
CCLE.PRAD
Adaptive immunity
CCLE.PRAD
Innate immunity
APOBEC3A
APOBEC3B
APOBEC3C
APOBEC3D
APOBEC3F
APOBEC3G
APOBEC3H
coexpression partners

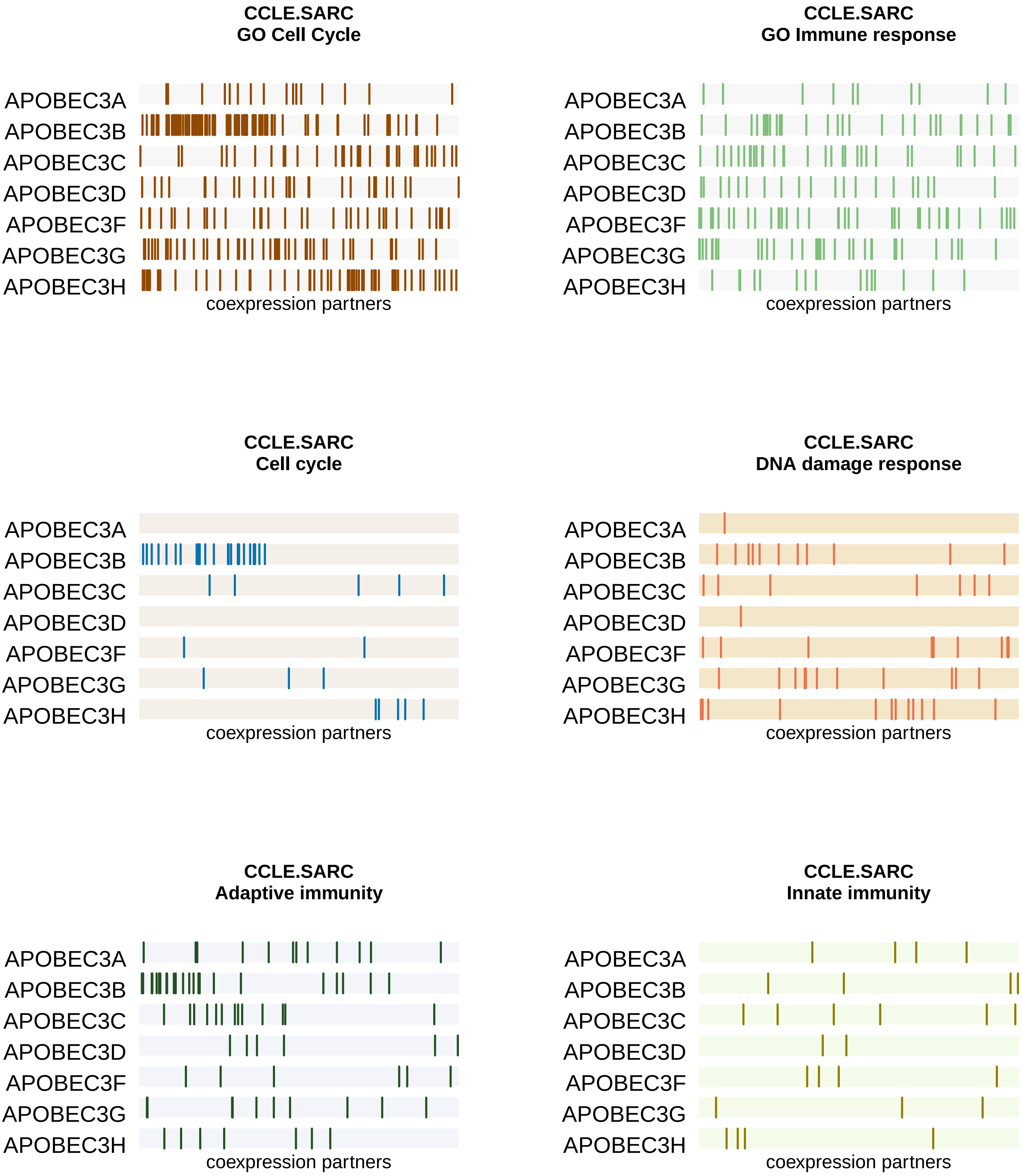
CCLE.SARC
GO Cell Cycle
CCLE.SARC
GO Immune response
CCLE.SARC
Cell cycle
CCLE.SARC
DNA damage response
CCLE.SARC
Adaptive immunity
CCLE.SARC
Innate immunity
APOBEC3A
APOBEC3B
APOBEC3C
APOBEC3D
APOBEC3F
APOBEC3G
APOBEC3H
coexpression partners

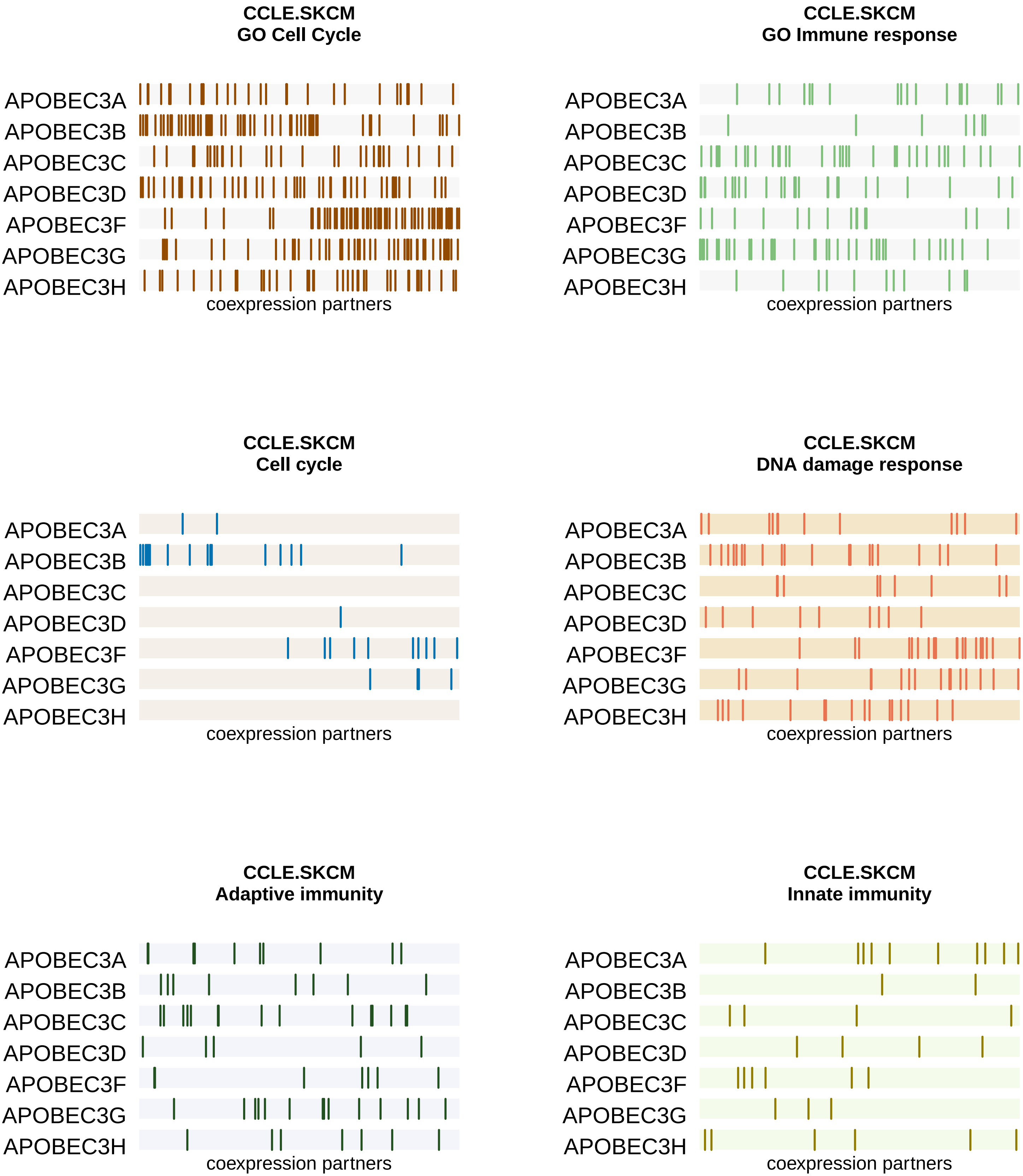
CCLE.SKCM
GO Cell Cycle
CCLE.SKCM
GO Immune response
CCLE.SKCM
Cell cycle
CCLE.SKCM
DNA damage response
CCLE.SKCM
Adaptive immunity
CCLE.SKCM
Innate immunity
APOBEC3A
APOBEC3B
APOBEC3C
APOBEC3D
APOBEC3F
APOBEC3G
APOBEC3H
coexpression partners

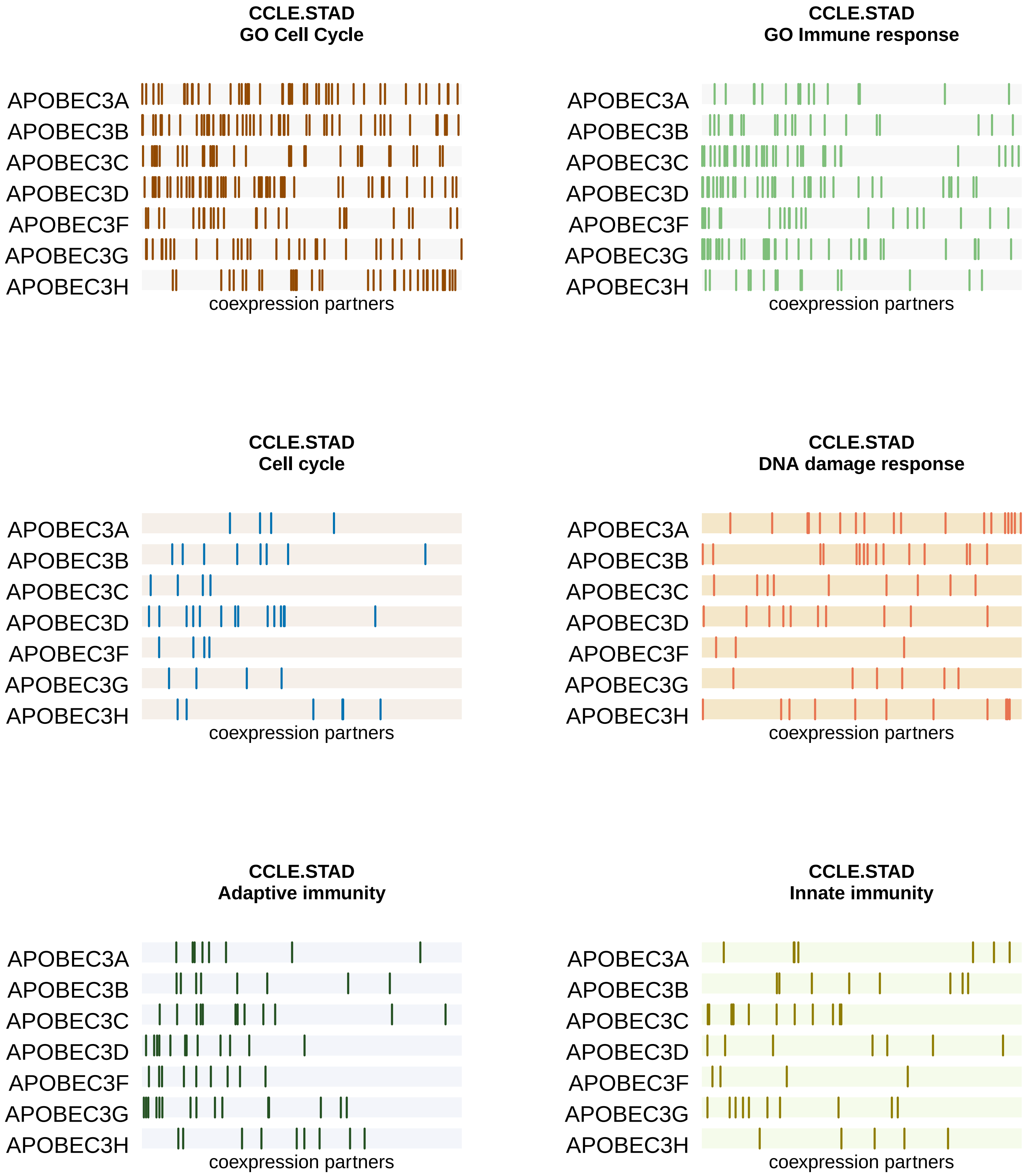

CCLE.STAD
GO Cell Cycle
CCLE.STAD
GO Immune response
CCLE.STAD
Cell cycle
CCLE.STAD
DNA damage response
CCLE.STAD
Adaptive immunity
CCLE.STAD
Innate immunity
APOBEC3A
APOBEC3B
APOBEC3C
APOBEC3D
APOBEC3F
APOBEC3G
APOBEC3H
coexpression partners

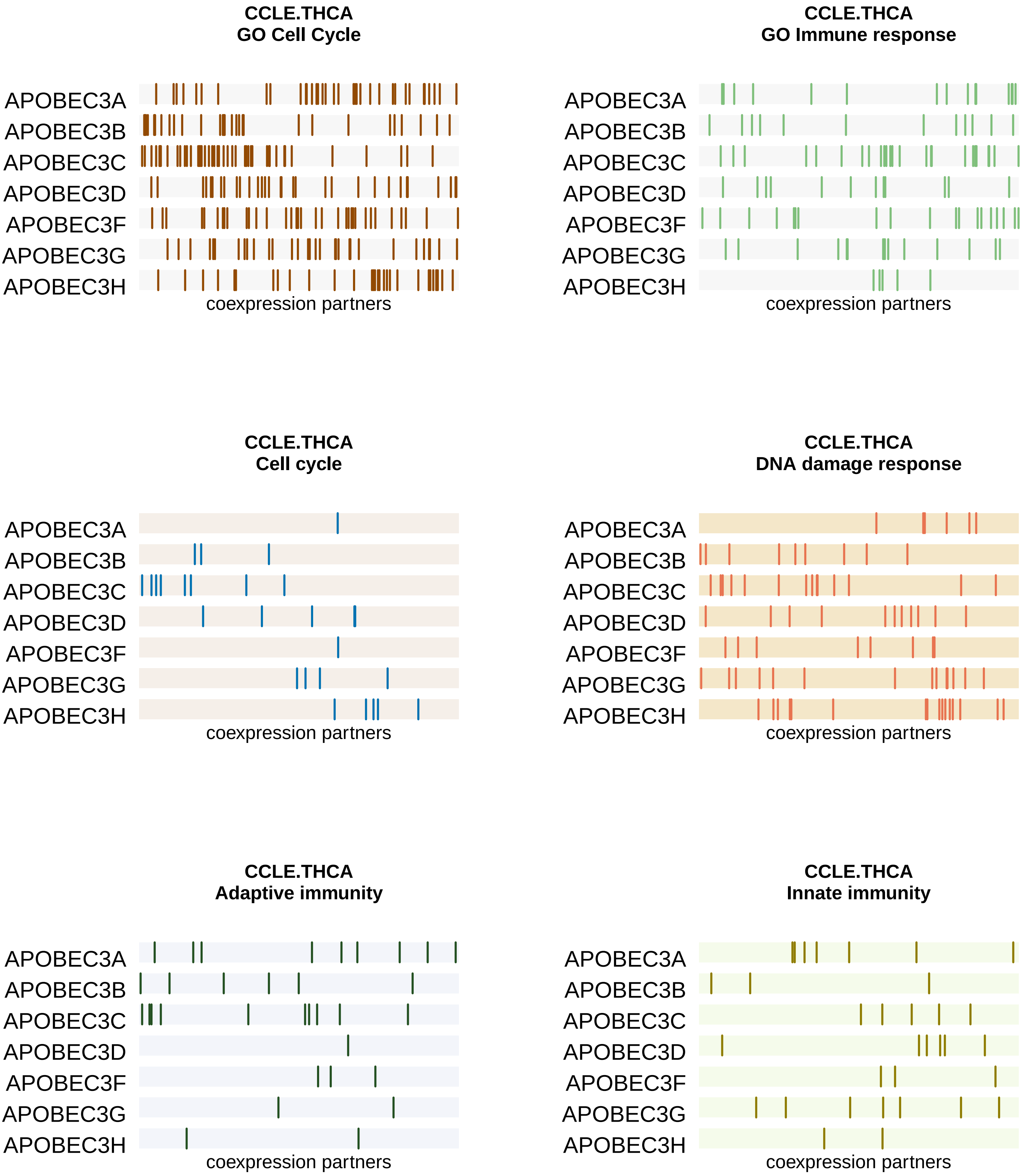
CCLE.THCA
GO Cell Cycle
CCLE.THCA
GO Immune response
CCLE.THCA
Cell cycle
CCLE.THCA
DNA damage response
CCLE.THCA
Adaptive immunity
CCLE.THCA
Innate immunity
APOBEC3A
APOBEC3B
APOBEC3C
APOBEC3D
APOBEC3F
APOBEC3G
APOBEC3H
coexpression partners

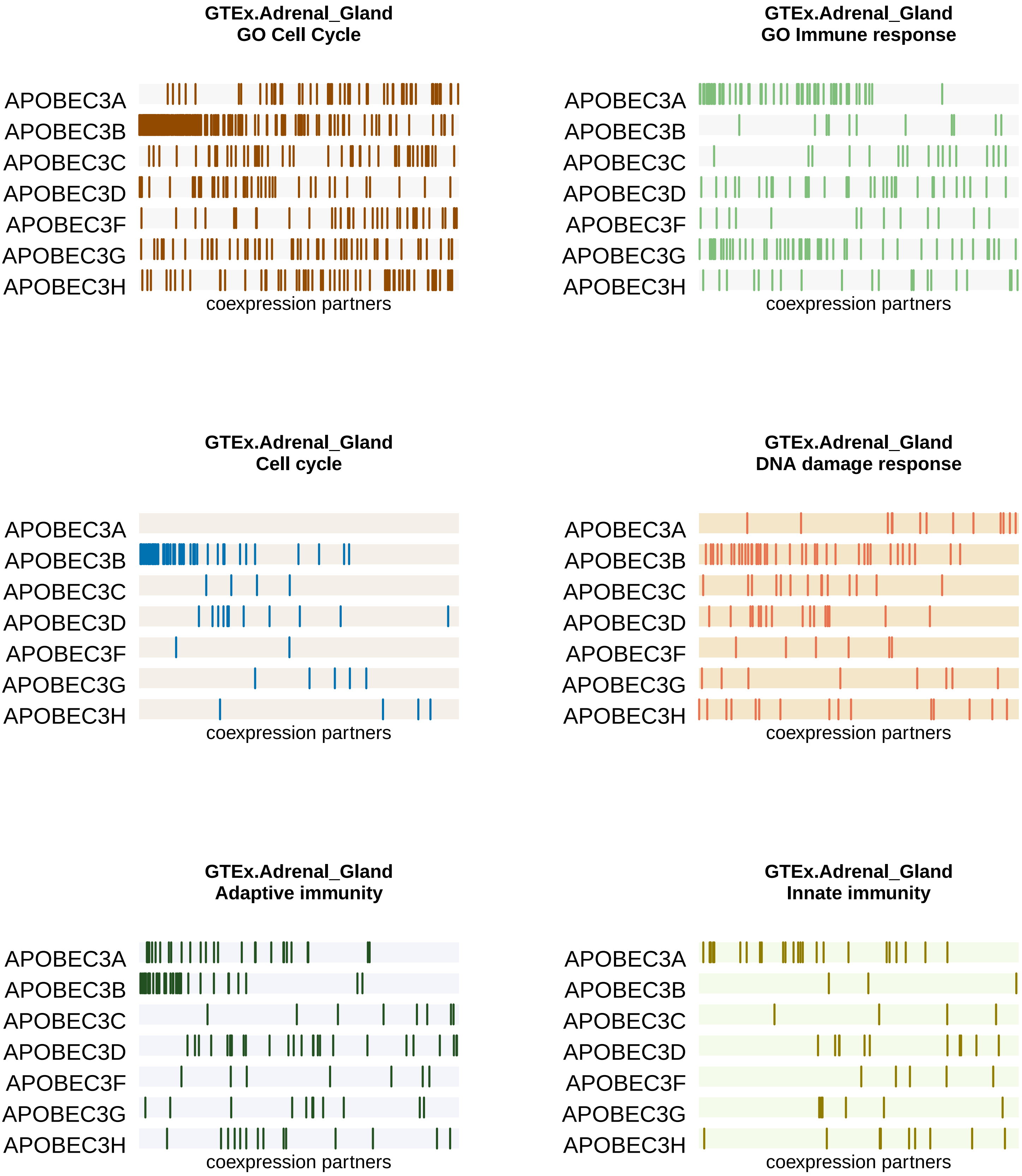
GTEx.Adrenal_Gland
GO Cell Cycle
APOBEC3A
APOBEC3B
APOBEC3C
APOBEC3D
APOBEC3F
APOBEC3G
APOBEC3H
coexpression partners
GTEx.Adrenal_Gland
GO Immune response
APOBEC3A
APOBEC3B
APOBEC3C
APOBEC3D
APOBEC3F
APOBEC3G
APOBEC3H
coexpression partners
GTEx.Adrenal_Gland
Cell cycle
APOBEC3A
APOBEC3B
APOBEC3C
APOBEC3D
APOBEC3F
APOBEC3G
APOBEC3H
coexpression partners
GTEx.Adrenal_Gland
DNA damage response
APOBEC3A
APOBEC3B
APOBEC3C
APOBEC3D
APOBEC3F
APOBEC3G
APOBEC3H
coexpression partners
GTEx.Adrenal_Gland
Adaptive immunity
APOBEC3A
APOBEC3B
APOBEC3C
APOBEC3D
APOBEC3F
APOBEC3G
APOBEC3H
coexpression partners
GTEx.Adrenal_Gland
Innate immunity
APOBEC3A
APOBEC3B
APOBEC3C
APOBEC3D
APOBEC3F
APOBEC3G
APOBEC3H
coexpression partners

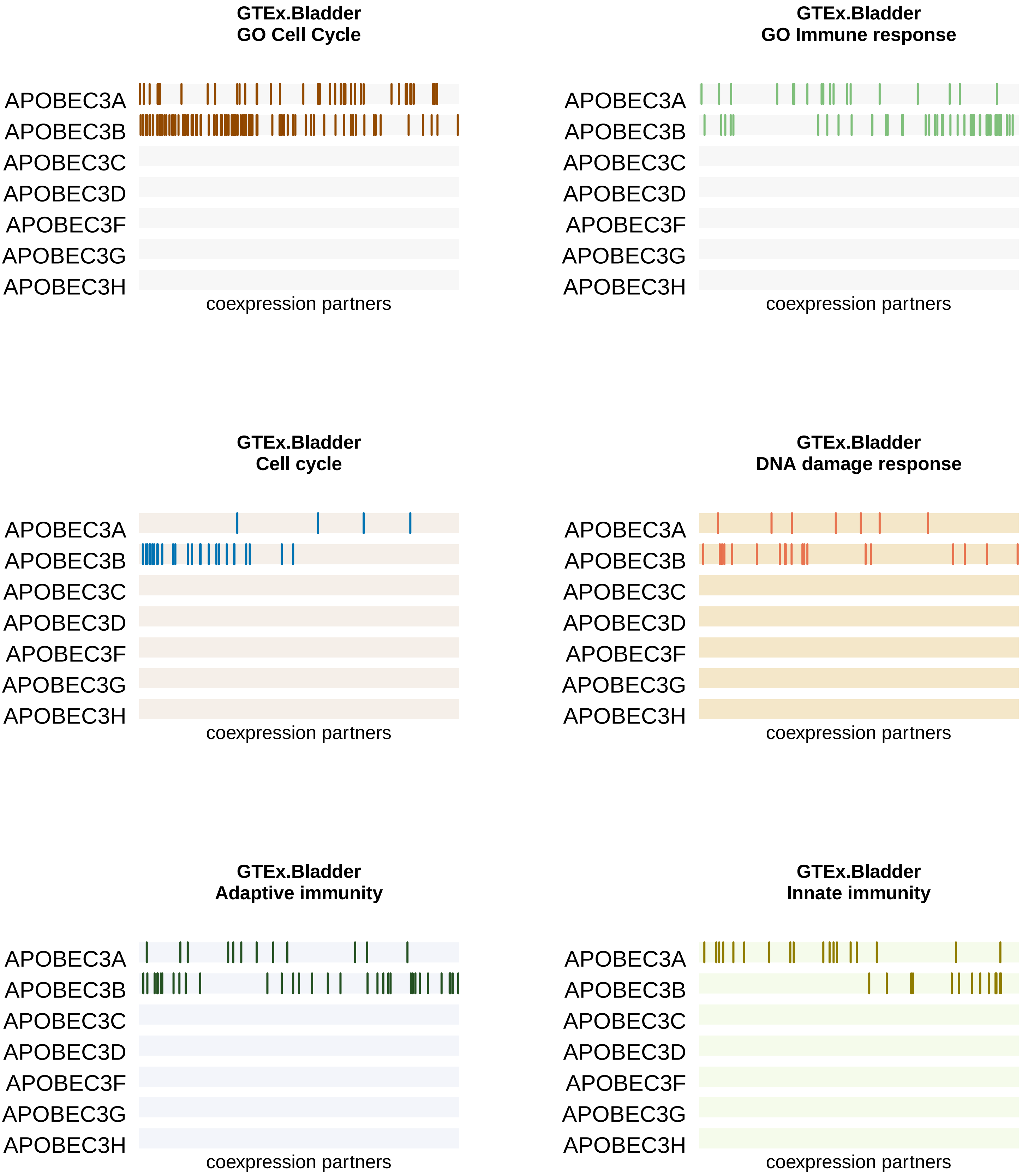
GTEx.Bladder
GO Cell Cycle
GTEx.Bladder
GO Immune response
GTEx.Bladder
Cell cycle
GTEx.Bladder
DNA damage response
GTEx.Bladder
Adaptive immunity
GTEx.Bladder
Innate immunity
APOBEC3A
APOBEC3B
APOBEC3C
APOBEC3D
APOBEC3F
APOBEC3G
APOBEC3H
coexpression partners

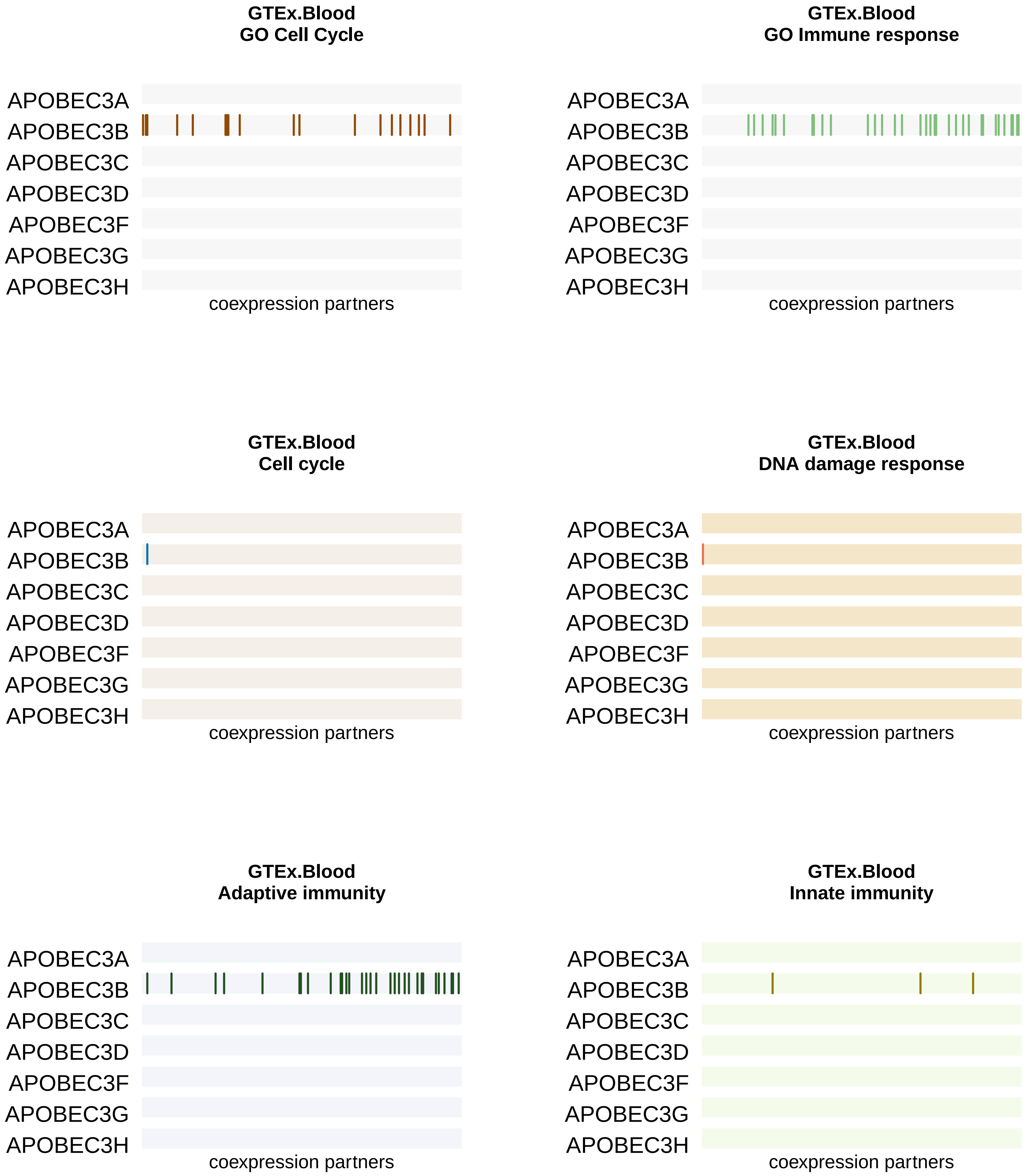
GTEx.Blood
GO Cell Cycle
APOBEC3A
APOBEC3B
APOBEC3C
APOBEC3D
APOBEC3F
APOBEC3G
APOBEC3H
coexpression partners
GTEx.Blood
GO Immune response
APOBEC3A
APOBEC3B
APOBEC3C
APOBEC3D
APOBEC3F
APOBEC3G
APOBEC3H
coexpression partners
GTEx.Blood
Cell cycle
APOBEC3A
APOBEC3B
APOBEC3C
APOBEC3D
APOBEC3F
APOBEC3G
APOBEC3H
coexpression partners
GTEx.Blood
DNA damage response
APOBEC3A
APOBEC3B
APOBEC3C
APOBEC3D
APOBEC3F
APOBEC3G
APOBEC3H
coexpression partners
GTEx.Blood
Adaptive immunity
APOBEC3A
APOBEC3B
APOBEC3C
APOBEC3D
APOBEC3F
APOBEC3G
APOBEC3H
coexpression partners
GTEx.Blood
Innate immunity
APOBEC3A
APOBEC3B
APOBEC3C
APOBEC3D
APOBEC3F
APOBEC3G
APOBEC3H
coexpression partners

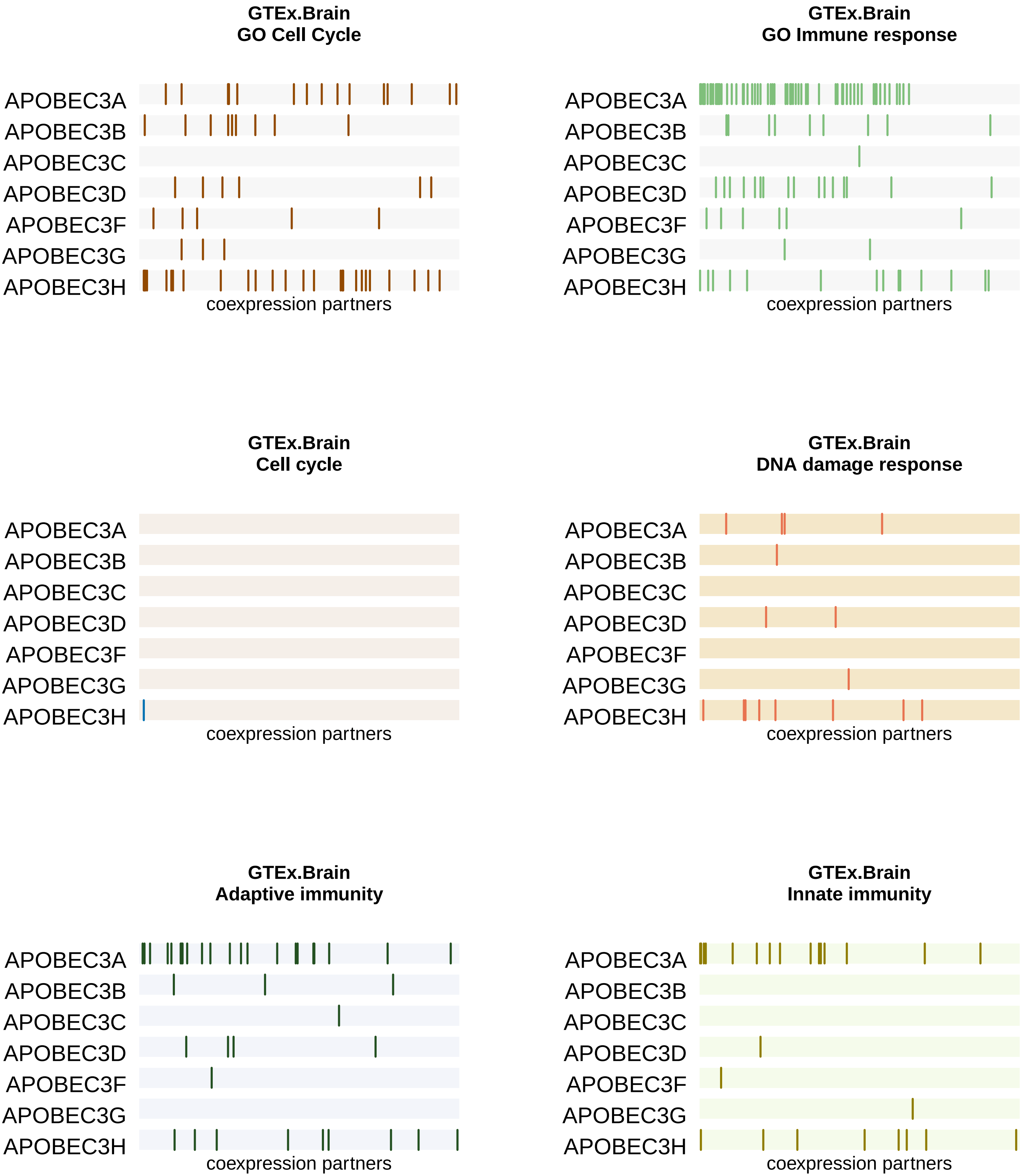
GTEx.Brain
GO Cell Cycle
APOBEC3A
APOBEC3B
APOBEC3C
APOBEC3D
APOBEC3F
APOBEC3G
APOBEC3H
coexpression partners
GTEx.Brain
GO Immune response
APOBEC3A
APOBEC3B
APOBEC3C
APOBEC3D
APOBEC3F
APOBEC3G
APOBEC3H
coexpression partners
GTEx.Brain
Cell cycle
APOBEC3A
APOBEC3B
APOBEC3C
APOBEC3D
APOBEC3F
APOBEC3G
APOBEC3H
coexpression partners
GTEx.Brain
DNA damage response
APOBEC3A
APOBEC3B
APOBEC3C
APOBEC3D
APOBEC3F
APOBEC3G
APOBEC3H
coexpression partners
GTEx.Brain
Adaptive immunity
APOBEC3A
APOBEC3B
APOBEC3C
APOBEC3D
APOBEC3F
APOBEC3G
APOBEC3H
coexpression partners
GTEx.Brain
Innate immunity
APOBEC3A
APOBEC3B
APOBEC3C
APOBEC3D
APOBEC3F
APOBEC3G
APOBEC3H
coexpression partners

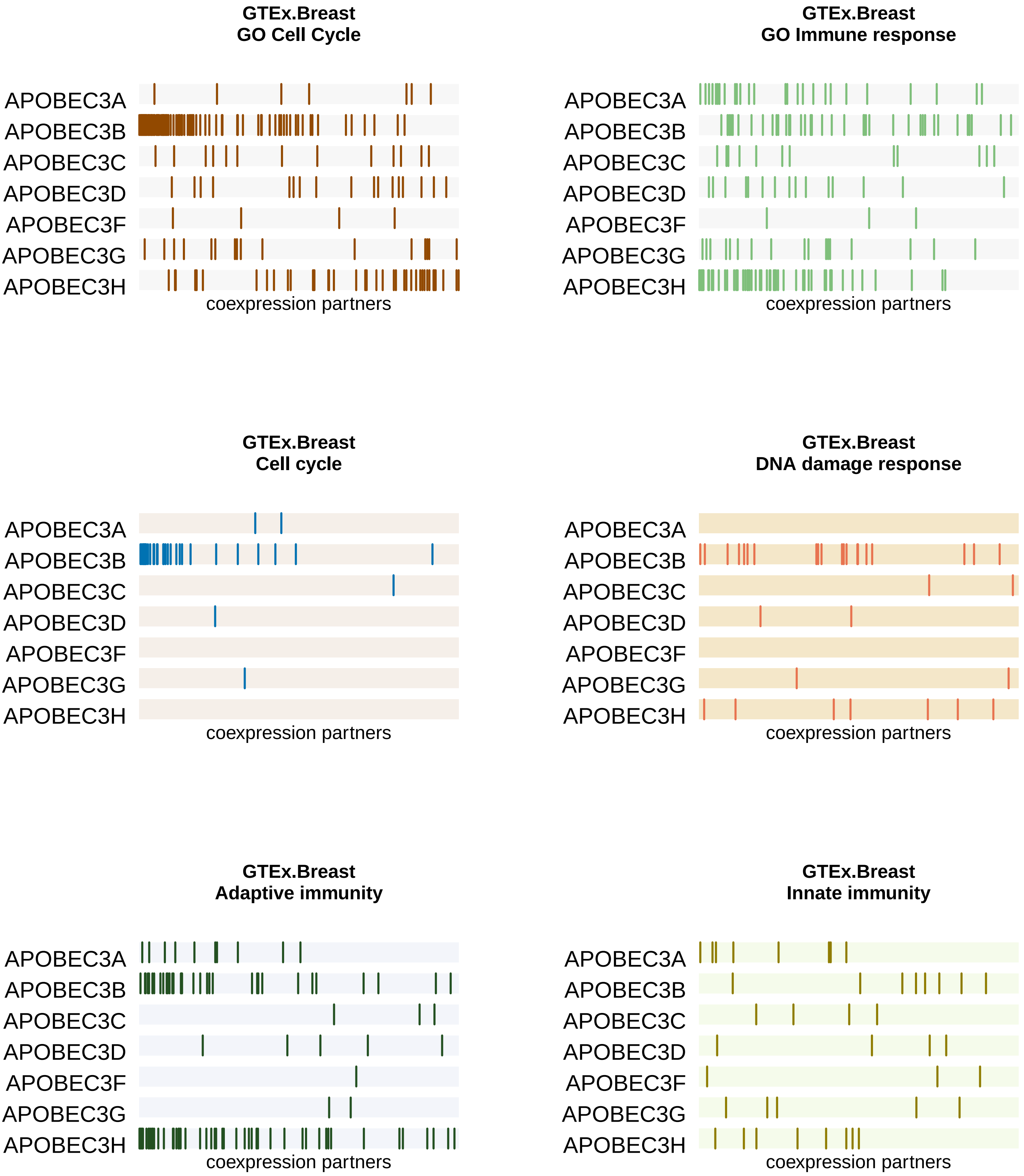
GTEx.Breast
GO Cell Cycle
APOBEC3A
APOBEC3B
APOBEC3C
APOBEC3D
APOBEC3F
APOBEC3G
APOBEC3H
coexpression partners
GTEx.Breast
GO Immune response
APOBEC3A
APOBEC3B
APOBEC3C
APOBEC3D
APOBEC3F
APOBEC3G
APOBEC3H
coexpression partners
GTEx.Breast
Cell cycle
APOBEC3A
APOBEC3B
APOBEC3C
APOBEC3D
APOBEC3F
APOBEC3G
APOBEC3H
coexpression partners
GTEx.Breast
DNA damage response
APOBEC3A
APOBEC3B
APOBEC3C
APOBEC3D
APOBEC3F
APOBEC3G
APOBEC3H
coexpression partners
GTEx.Breast
Adaptive immunity
APOBEC3A
APOBEC3B
APOBEC3C
APOBEC3D
APOBEC3F
APOBEC3G
APOBEC3H
coexpression partners
GTEx.Breast
Innate immunity
APOBEC3A
APOBEC3B
APOBEC3C
APOBEC3D
APOBEC3F
APOBEC3G
APOBEC3H
coexpression partners

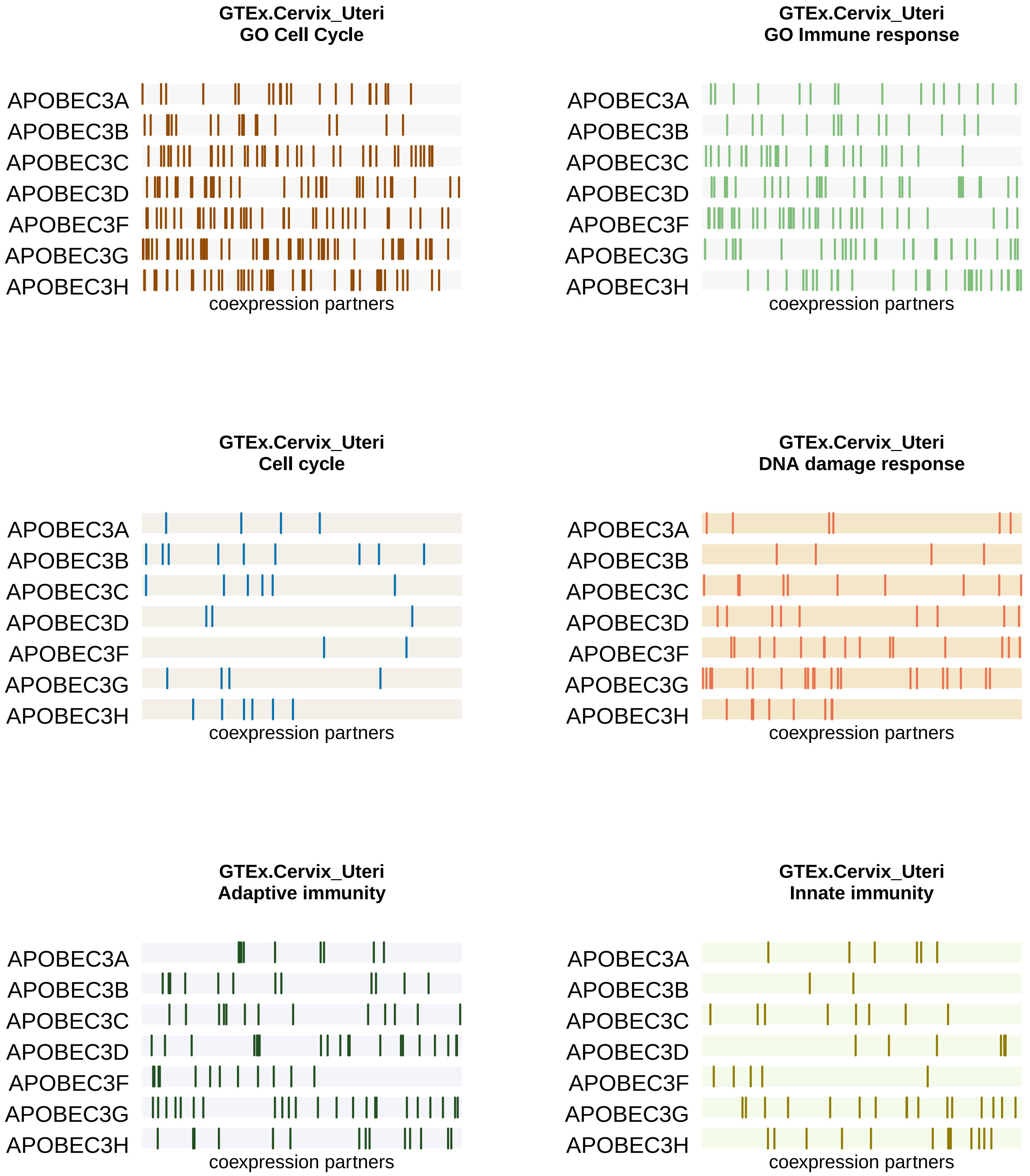

GTEx.Cervix_Uteri
GO Cell Cycle
APOBEC3A
APOBEC3B
APOBEC3C
APOBEC3D
APOBEC3F
APOBEC3G
APOBEC3H
coexpression partners
GTEx.Cervix_Uteri
GO Immune response
APOBEC3A
APOBEC3B
APOBEC3C
APOBEC3D
APOBEC3F
APOBEC3G
APOBEC3H
coexpression partners
GTEx.Cervix_Uteri
Cell cycle
APOBEC3A
APOBEC3B
APOBEC3C
APOBEC3D
APOBEC3F
APOBEC3G
APOBEC3H
coexpression partners
GTEx.Cervix_Uteri
DNA damage response
APOBEC3A
APOBEC3B
APOBEC3C
APOBEC3D
APOBEC3F
APOBEC3G
APOBEC3H
coexpression partners
GTEx.Cervix_Uteri
Adaptive immunity
APOBEC3A
APOBEC3B
APOBEC3C
APOBEC3D
APOBEC3F
APOBEC3G
APOBEC3H
coexpression partners
GTEx.Cervix_Uteri
Innate immunity
APOBEC3A
APOBEC3B
APOBEC3C
APOBEC3D
APOBEC3F
APOBEC3G
APOBEC3H
coexpression partners

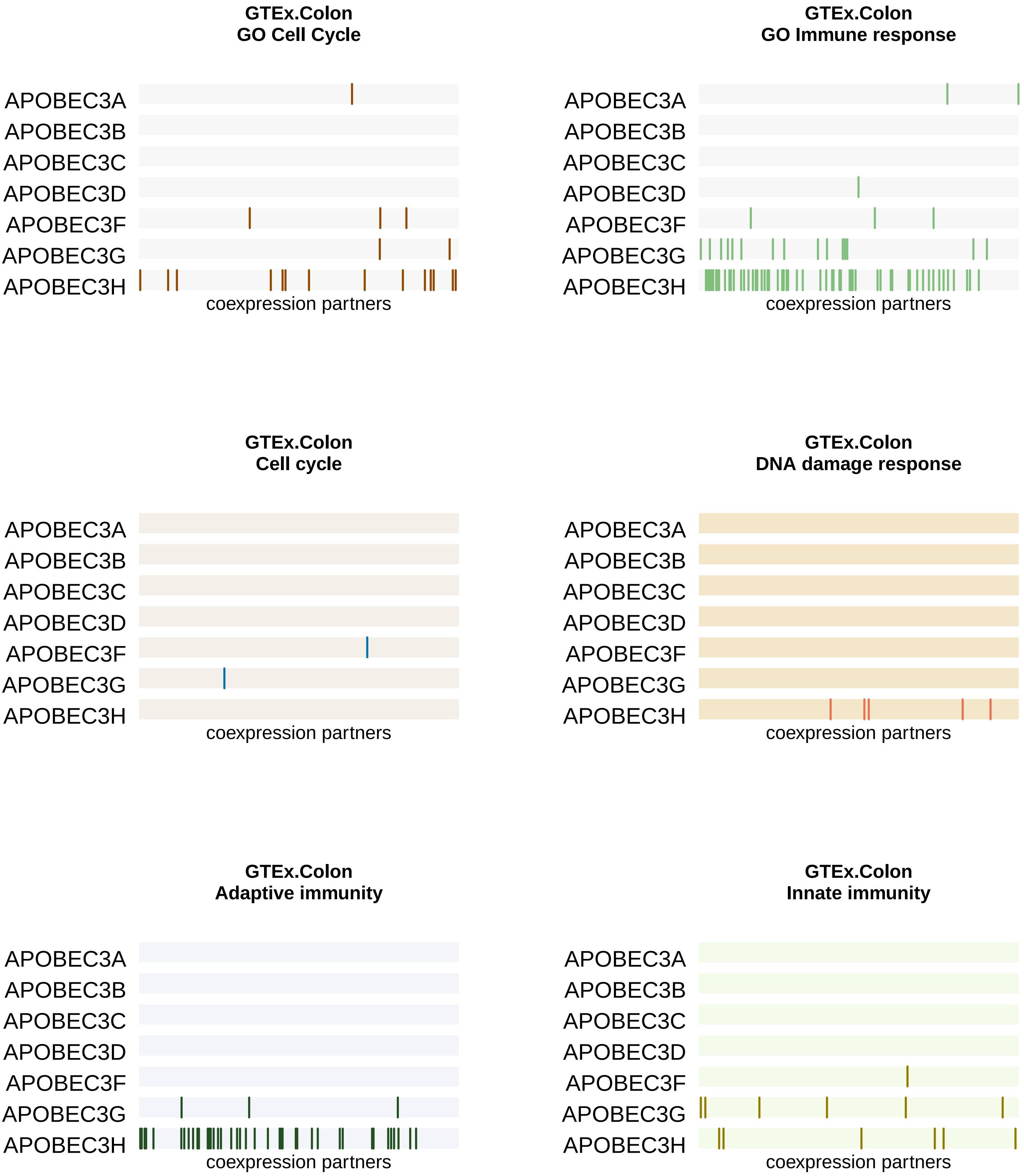
GTEx.Colon
GO Cell Cycle
APOBEC3A
APOBEC3B
APOBEC3C
APOBEC3D
APOBEC3F
APOBEC3G
APOBEC3H
coexpression partners
GTEx.Colon
GO Immune response
APOBEC3A
APOBEC3B
APOBEC3C
APOBEC3D
APOBEC3F
APOBEC3G
APOBEC3H
coexpression partners
GTEx.Colon
Cell cycle
APOBEC3A
APOBEC3B
APOBEC3C
APOBEC3D
APOBEC3F
APOBEC3G
APOBEC3H
coexpression partners
GTEx.Colon
DNA damage response
APOBEC3A
APOBEC3B
APOBEC3C
APOBEC3D
APOBEC3F
APOBEC3G
APOBEC3H
coexpression partners
GTEx.Colon
Adaptive immunity
APOBEC3A
APOBEC3B
APOBEC3C
APOBEC3D
APOBEC3F
APOBEC3G
APOBEC3H
coexpression partners
GTEx.Colon
Innate immunity
APOBEC3A
APOBEC3B
APOBEC3C
APOBEC3D
APOBEC3F
APOBEC3G
APOBEC3H
coexpression partners

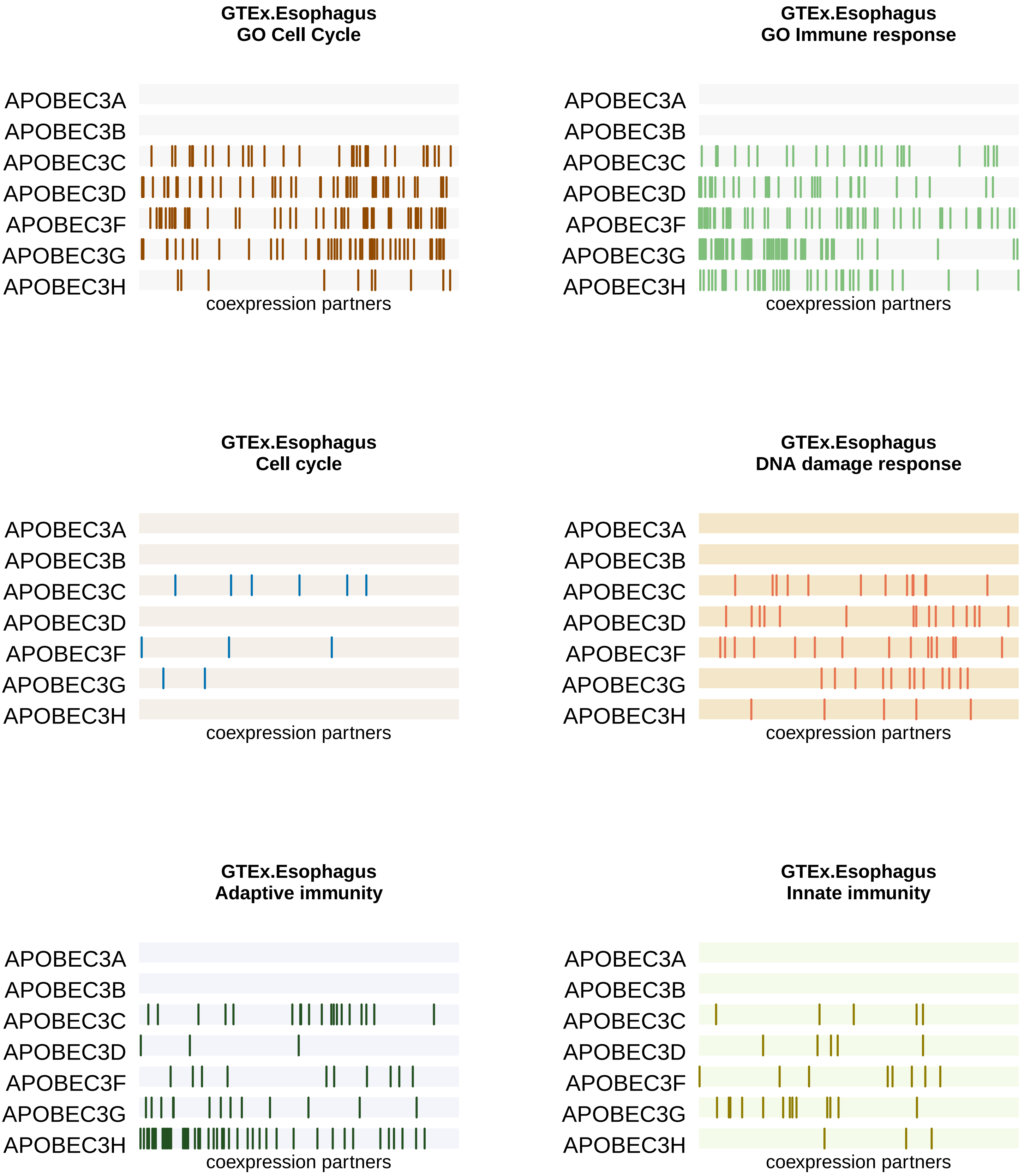
GTEx.Esophagus
GO Cell Cycle
APOBEC3A
APOBEC3B
APOBEC3C
APOBEC3D
APOBEC3F
APOBEC3G
APOBEC3H
coexpression partners
GTEx.Esophagus
GO Immune response
APOBEC3A
APOBEC3B
APOBEC3C
APOBEC3D
APOBEC3F
APOBEC3G
APOBEC3H
coexpression partners
GTEx.Esophagus
Cell cycle
APOBEC3A
APOBEC3B
APOBEC3C
APOBEC3D
APOBEC3F
APOBEC3G
APOBEC3H
coexpression partners
GTEx.Esophagus
DNA damage response
APOBEC3A
APOBEC3B
APOBEC3C
APOBEC3D
APOBEC3F
APOBEC3G
APOBEC3H
coexpression partners
GTEx.Esophagus
Adaptive immunity
APOBEC3A
APOBEC3B
APOBEC3C
APOBEC3D
APOBEC3F
APOBEC3G
APOBEC3H
coexpression partners
GTEx.Esophagus
Innate immunity
APOBEC3A
APOBEC3B
APOBEC3C
APOBEC3D
APOBEC3F
APOBEC3G
APOBEC3H
coexpression partners

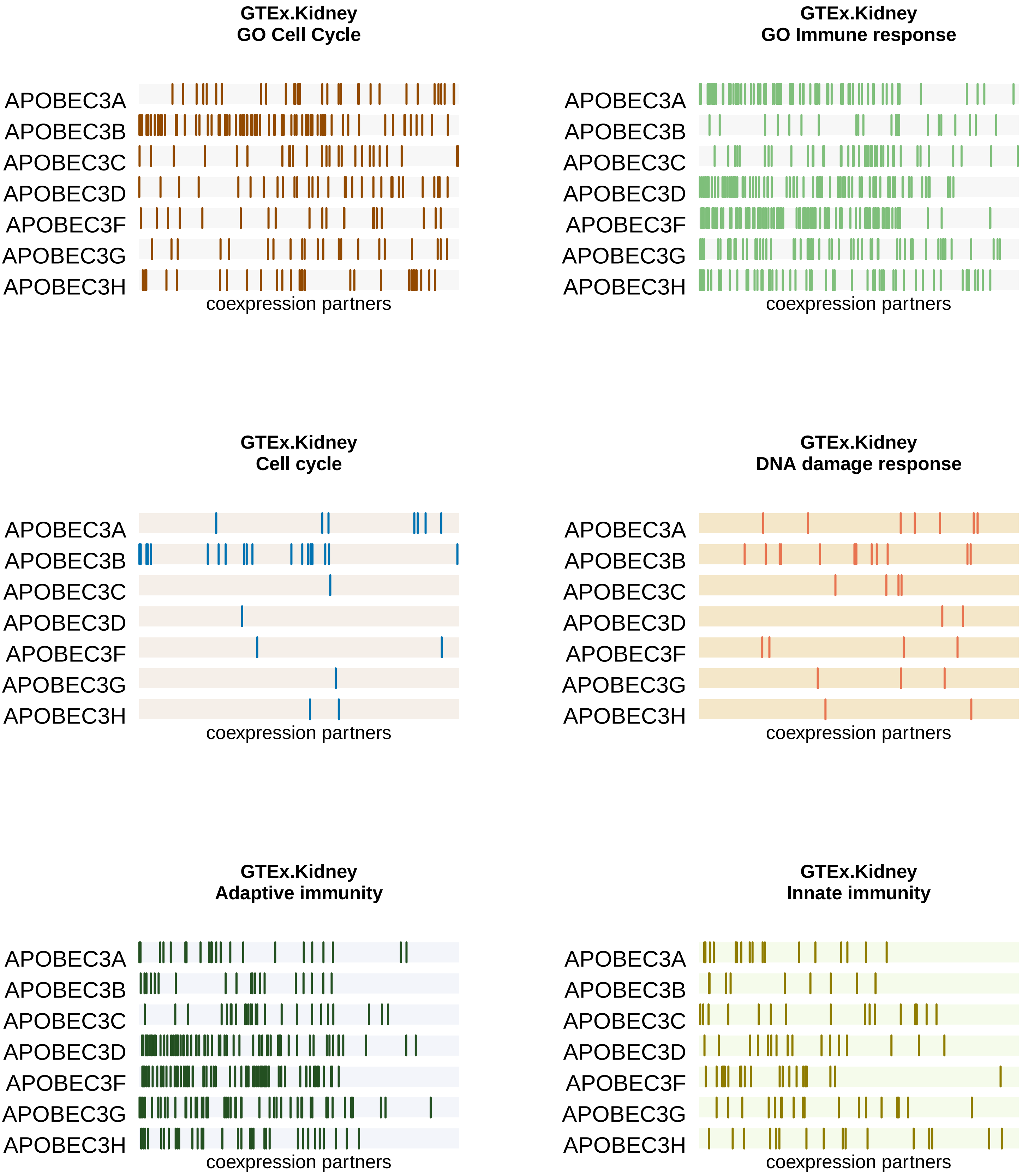
GTEx.Kidney
GO Cell Cycle
GTEx.Kidney
GO Immune response
GTEx.Kidney
Cell cycle
GTEx.Kidney
DNA damage response
GTEx.Kidney
Adaptive immunity
GTEx.Kidney
Innate immunity
APOBEC3A
APOBEC3B
APOBEC3C
APOBEC3D
APOBEC3F
APOBEC3G
APOBEC3H
coexpression partners

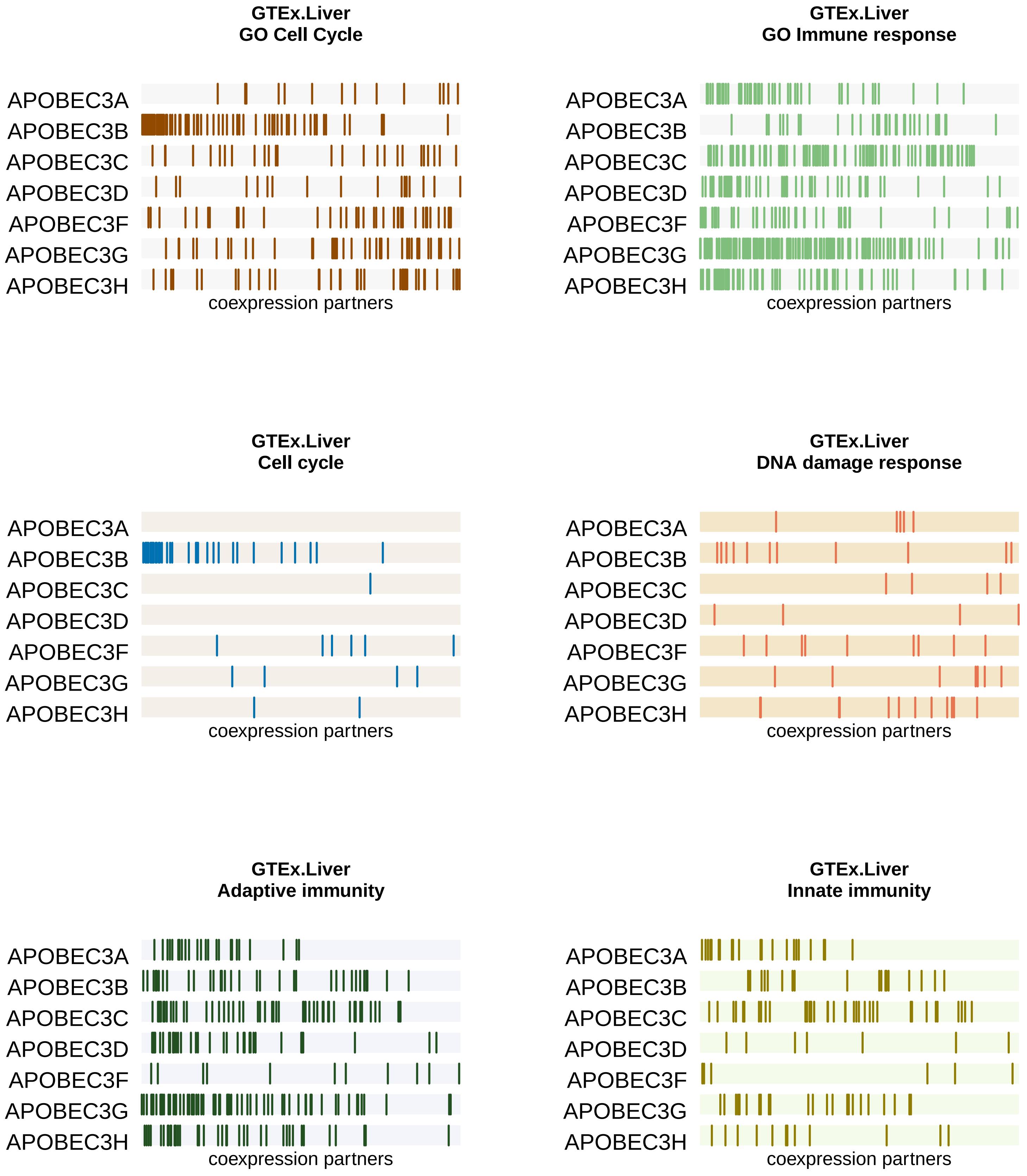
GTEx.Liver
GO Cell Cycle
APOBEC3A
APOBEC3B
APOBEC3C
APOBEC3D
APOBEC3F
APOBEC3G
APOBEC3H
coexpression partners
GTEx.Liver
GO Immune response
APOBEC3A
APOBEC3B
APOBEC3C
APOBEC3D
APOBEC3F
APOBEC3G
APOBEC3H
coexpression partners
GTEx.Liver
Cell cycle
APOBEC3A
APOBEC3B
APOBEC3C
APOBEC3D
APOBEC3F
APOBEC3G
APOBEC3H
coexpression partners
GTEx.Liver
DNA damage response
APOBEC3A
APOBEC3B
APOBEC3C
APOBEC3D
APOBEC3F
APOBEC3G
APOBEC3H
coexpression partners
GTEx.Liver
Adaptive immunity
APOBEC3A
APOBEC3B
APOBEC3C
APOBEC3D
APOBEC3F
APOBEC3G
APOBEC3H
coexpression partners
GTEx.Liver
Innate immunity
APOBEC3A
APOBEC3B
APOBEC3C
APOBEC3D
APOBEC3F
APOBEC3G
APOBEC3H
coexpression partners

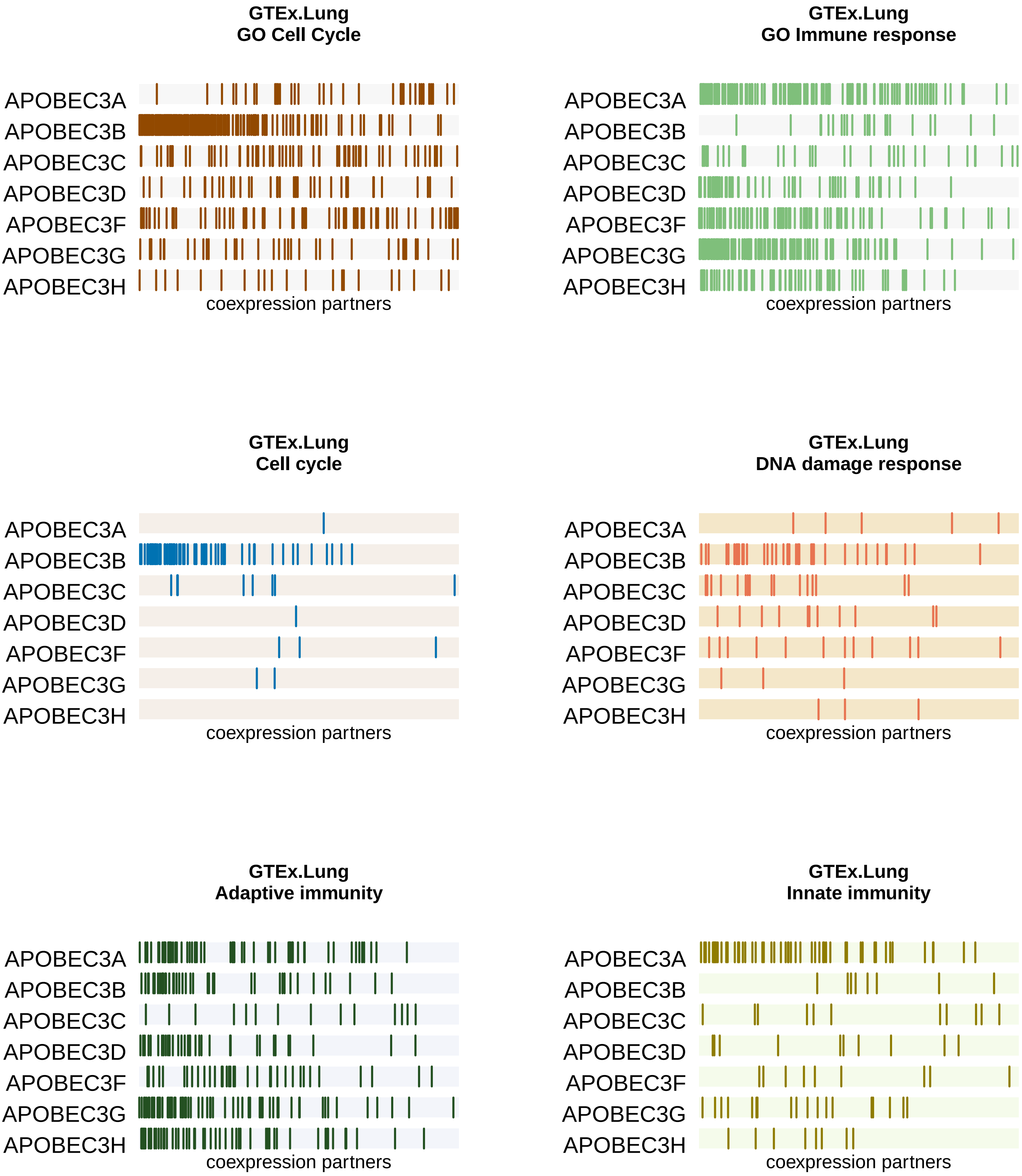
GTEx.Lung
GO Cell Cycle
GTEx.Lung
GO Immune response
GTEx.Lung
Cell cycle
GTEx.Lung
DNA damage response
GTEx.Lung
Adaptive immunity
GTEx.Lung
Innate immunity
APOBEC3A
APOBEC3B
APOBEC3C
APOBEC3D
APOBEC3F
APOBEC3G
APOBEC3H
coexpression partners

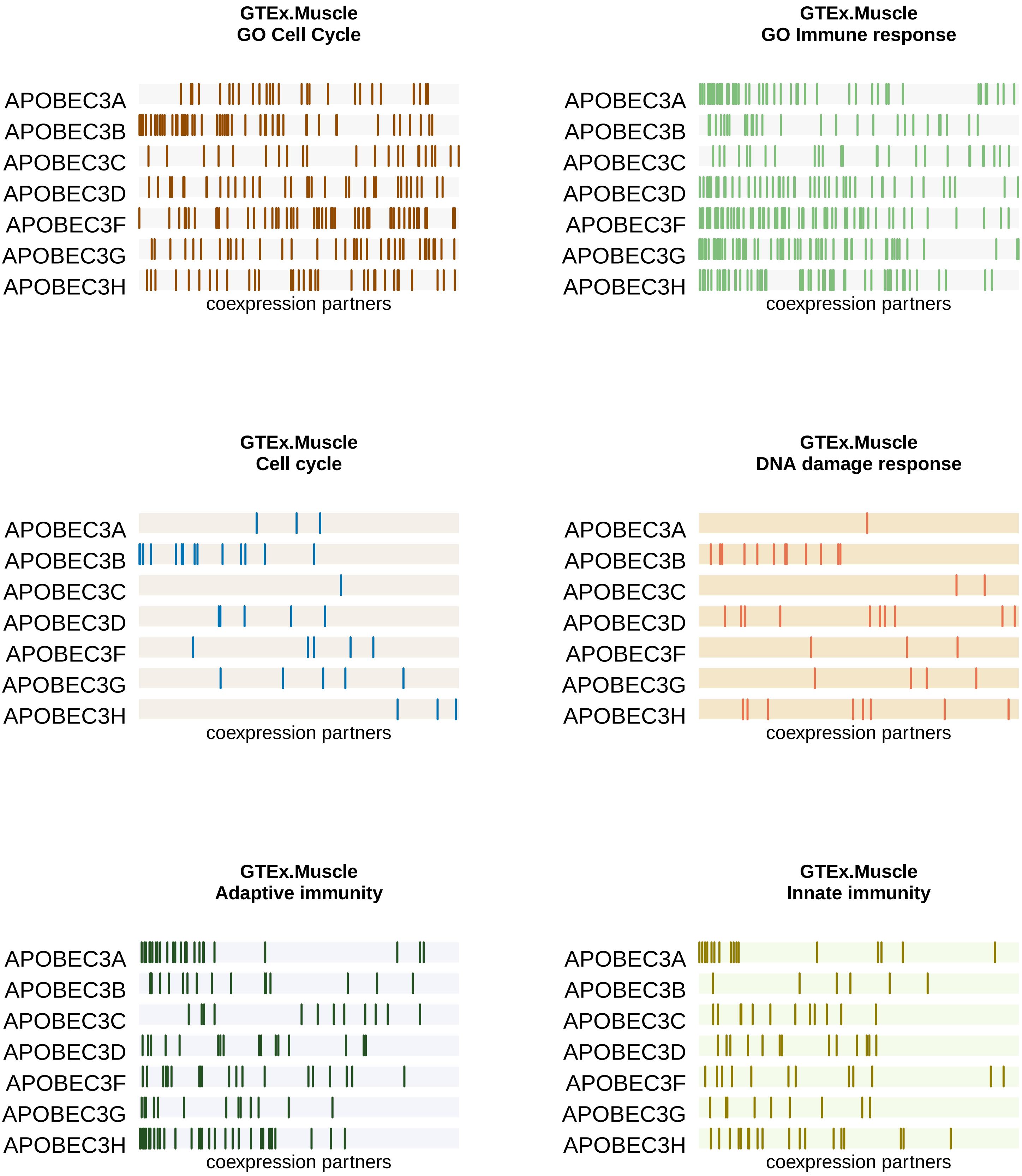
GTEx.Muscle
GO Cell Cycle
GTEx.Muscle
GO Immune response
GTEx.Muscle
Cell cycle
GTEx.Muscle
DNA damage response
GTEx.Muscle
Adaptive immunity
GTEx.Muscle
Innate immunity
APOBEC3A
APOBEC3B
APOBEC3C
APOBEC3D
APOBEC3F
APOBEC3G
APOBEC3H
coexpression partners

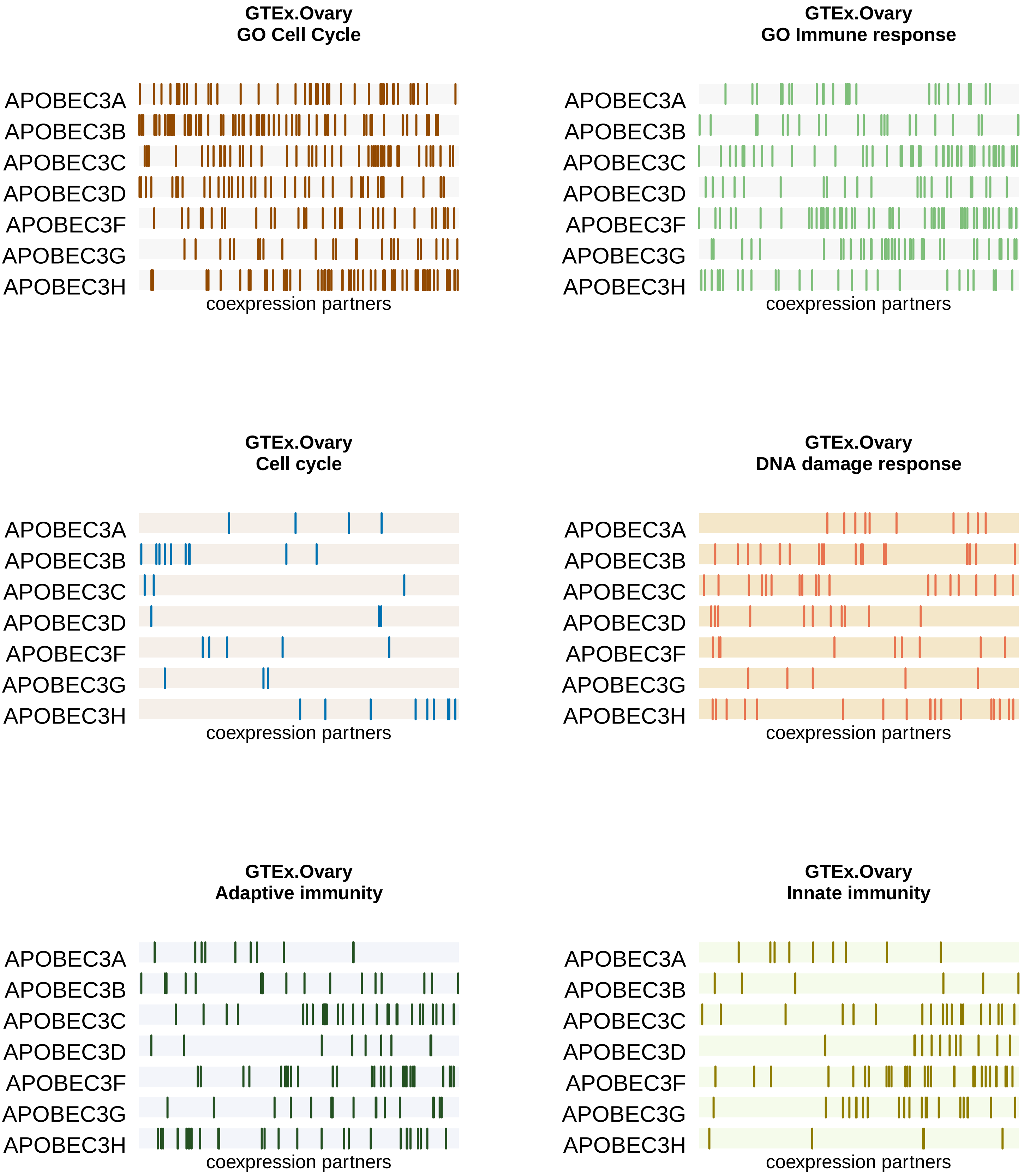
GTEx.Ovary
GO Cell Cycle
APOBEC3A
APOBEC3B
APOBEC3C
APOBEC3D
APOBEC3F
APOBEC3G
APOBEC3H
coexpression partners
GTEx.Ovary
GO Immune response
APOBEC3A
APOBEC3B
APOBEC3C
APOBEC3D
APOBEC3F
APOBEC3G
APOBEC3H
coexpression partners
GTEx.Ovary
Cell cycle
APOBEC3A
APOBEC3B
APOBEC3C
APOBEC3D
APOBEC3F
APOBEC3G
APOBEC3H
coexpression partners
GTEx.Ovary
DNA damage response
APOBEC3A
APOBEC3B
APOBEC3C
APOBEC3D
APOBEC3F
APOBEC3G
APOBEC3H
coexpression partners
GTEx.Ovary
Adaptive immunity
APOBEC3A
APOBEC3B
APOBEC3C
APOBEC3D
APOBEC3F
APOBEC3G
APOBEC3H
coexpression partners
GTEx.Ovary
Innate immunity
APOBEC3A
APOBEC3B
APOBEC3C
APOBEC3D
APOBEC3F
APOBEC3G
APOBEC3H
coexpression partners

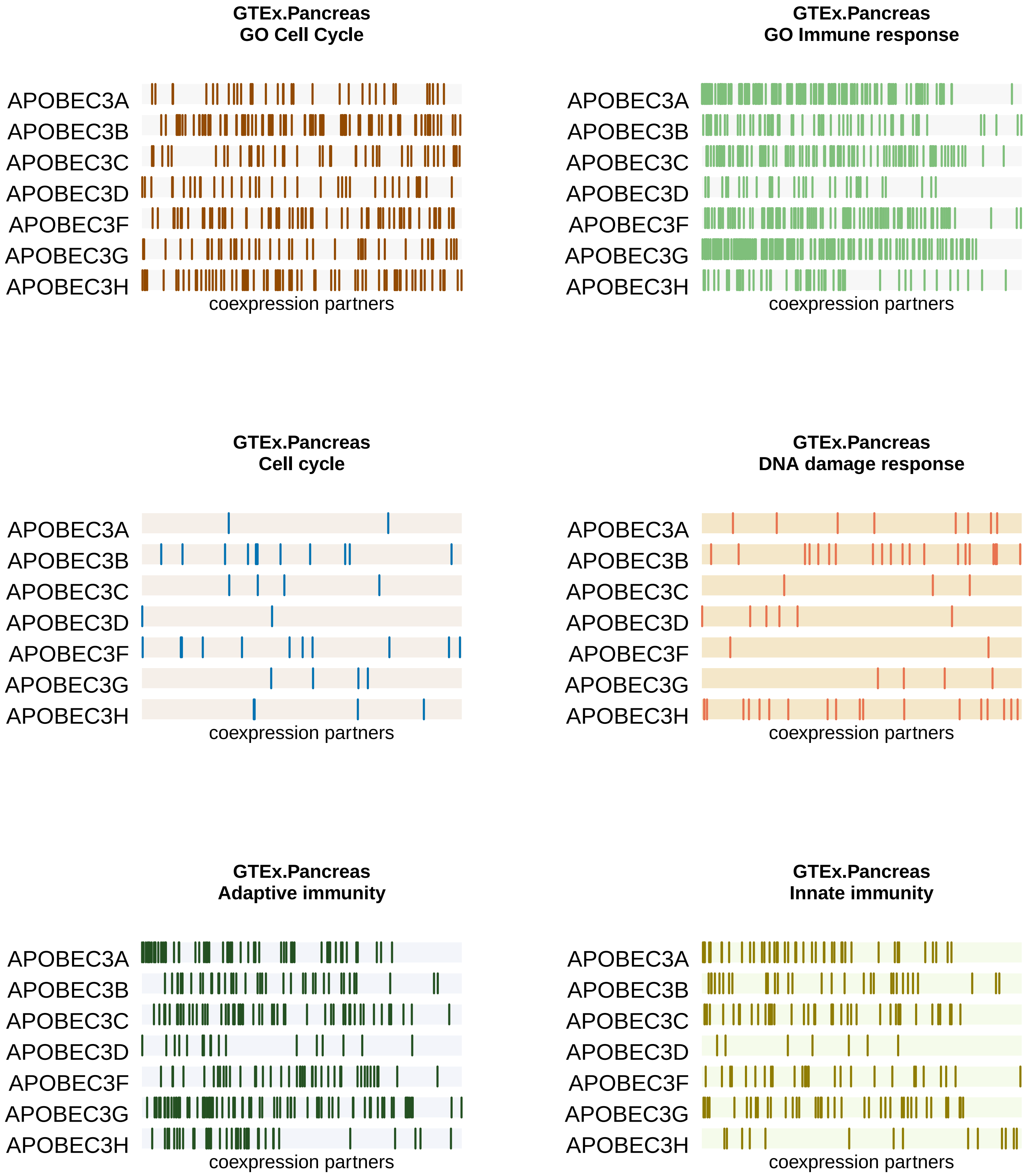

GTEx.Pancreas
GO Cell Cycle
GTEx.Pancreas
GO Immune response
GTEx.Pancreas
Cell cycle
GTEx.Pancreas
DNA damage response
GTEx.Pancreas
Adaptive immunity
GTEx.Pancreas
Innate immunity
APOBEC3A
APOBEC3B
APOBEC3C
APOBEC3D
APOBEC3F
APOBEC3G
APOBEC3H
coexpression partners

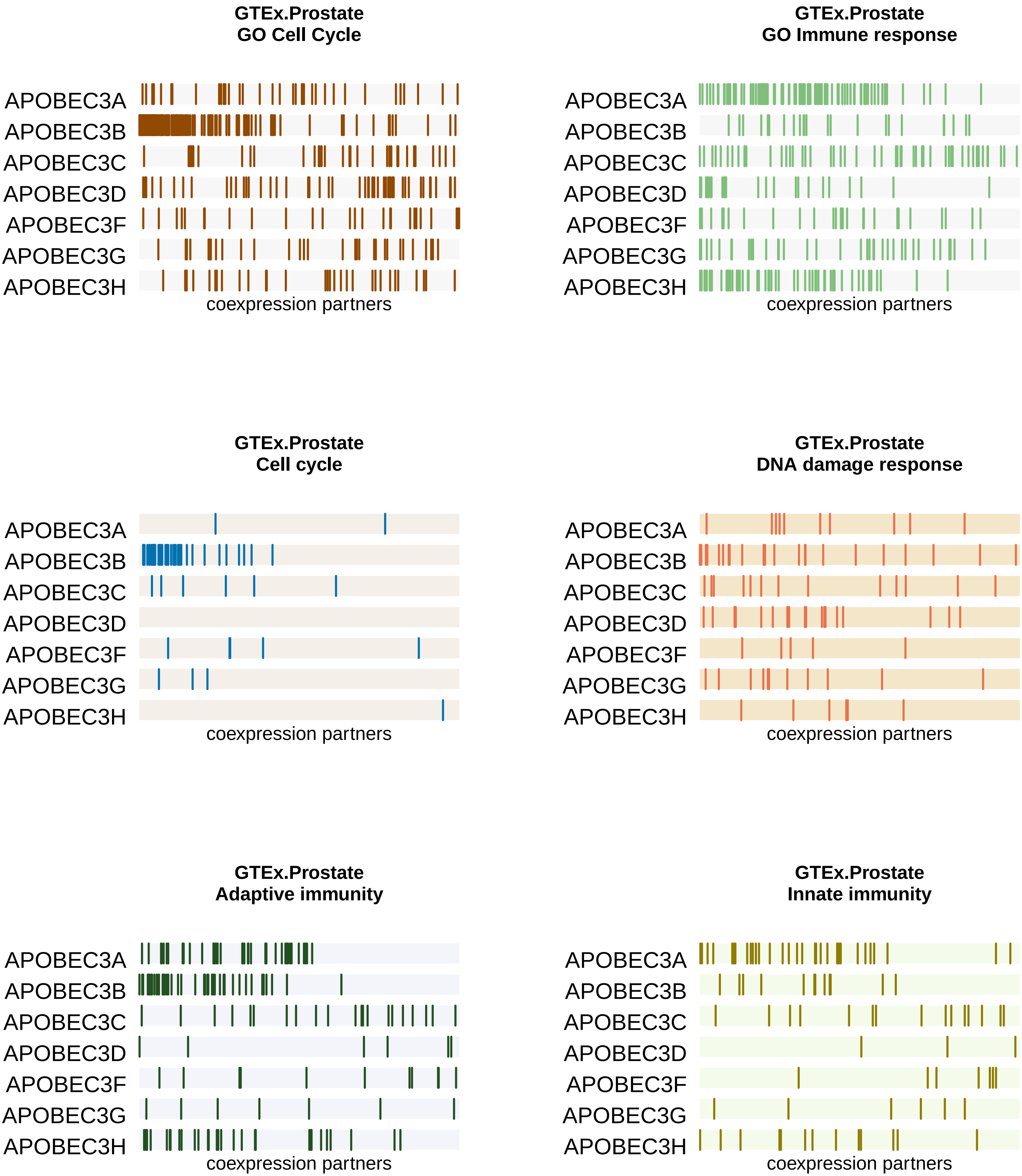
GTEx.Prostate
GO Cell Cycle
APOBEC3A
APOBEC3B
APOBEC3C
APOBEC3D
APOBEC3F
APOBEC3G
APOBEC3H
coexpression partners
GTEx.Prostate
GO Immune response
APOBEC3A
APOBEC3B
APOBEC3C
APOBEC3D
APOBEC3F
APOBEC3G
APOBEC3H
coexpression partners
GTEx.Prostate
Cell cycle
APOBEC3A
APOBEC3B
APOBEC3C
APOBEC3D
APOBEC3F
APOBEC3G
APOBEC3H
coexpression partners
GTEx.Prostate
DNA damage response
APOBEC3A
APOBEC3B
APOBEC3C
APOBEC3D
APOBEC3F
APOBEC3G
APOBEC3H
coexpression partners
GTEx.Prostate
Adaptive immunity
APOBEC3A
APOBEC3B
APOBEC3C
APOBEC3D
APOBEC3F
APOBEC3G
APOBEC3H
coexpression partners
GTEx.Prostate
Innate immunity
APOBEC3A
APOBEC3B
APOBEC3C
APOBEC3D
APOBEC3F
APOBEC3G
APOBEC3H
coexpression partners

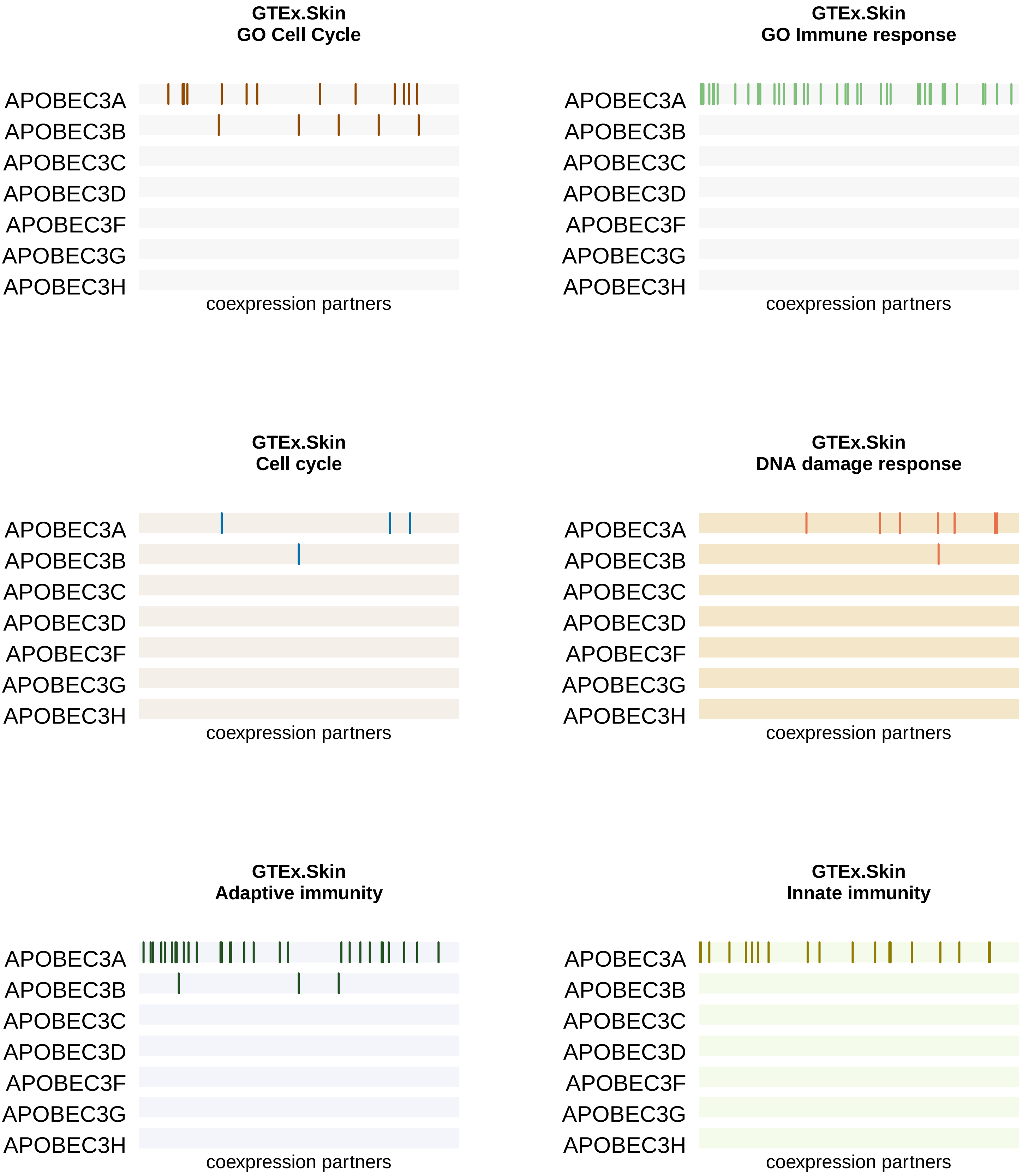
GTEx.Skin
GO Cell Cycle
APOBEC3A
APOBEC3B
APOBEC3C
APOBEC3D
APOBEC3F
APOBEC3G
APOBEC3H
coexpression partners
GTEx.Skin
GO Immune response
APOBEC3A
APOBEC3B
APOBEC3C
APOBEC3D
APOBEC3F
APOBEC3G
APOBEC3H
coexpression partners
GTEx.Skin
Cell cycle
APOBEC3A
APOBEC3B
APOBEC3C
APOBEC3D
APOBEC3F
APOBEC3G
APOBEC3H
coexpression partners
GTEx.Skin
DNA damage response
APOBEC3A
APOBEC3B
APOBEC3C
APOBEC3D
APOBEC3F
APOBEC3G
APOBEC3H
coexpression partners
GTEx.Skin
Adaptive immunity
APOBEC3A
APOBEC3B
APOBEC3C
APOBEC3D
APOBEC3F
APOBEC3G
APOBEC3H
coexpression partners
GTEx.Skin
Innate immunity
APOBEC3A
APOBEC3B
APOBEC3C
APOBEC3D
APOBEC3F
APOBEC3G
APOBEC3H
coexpression partners

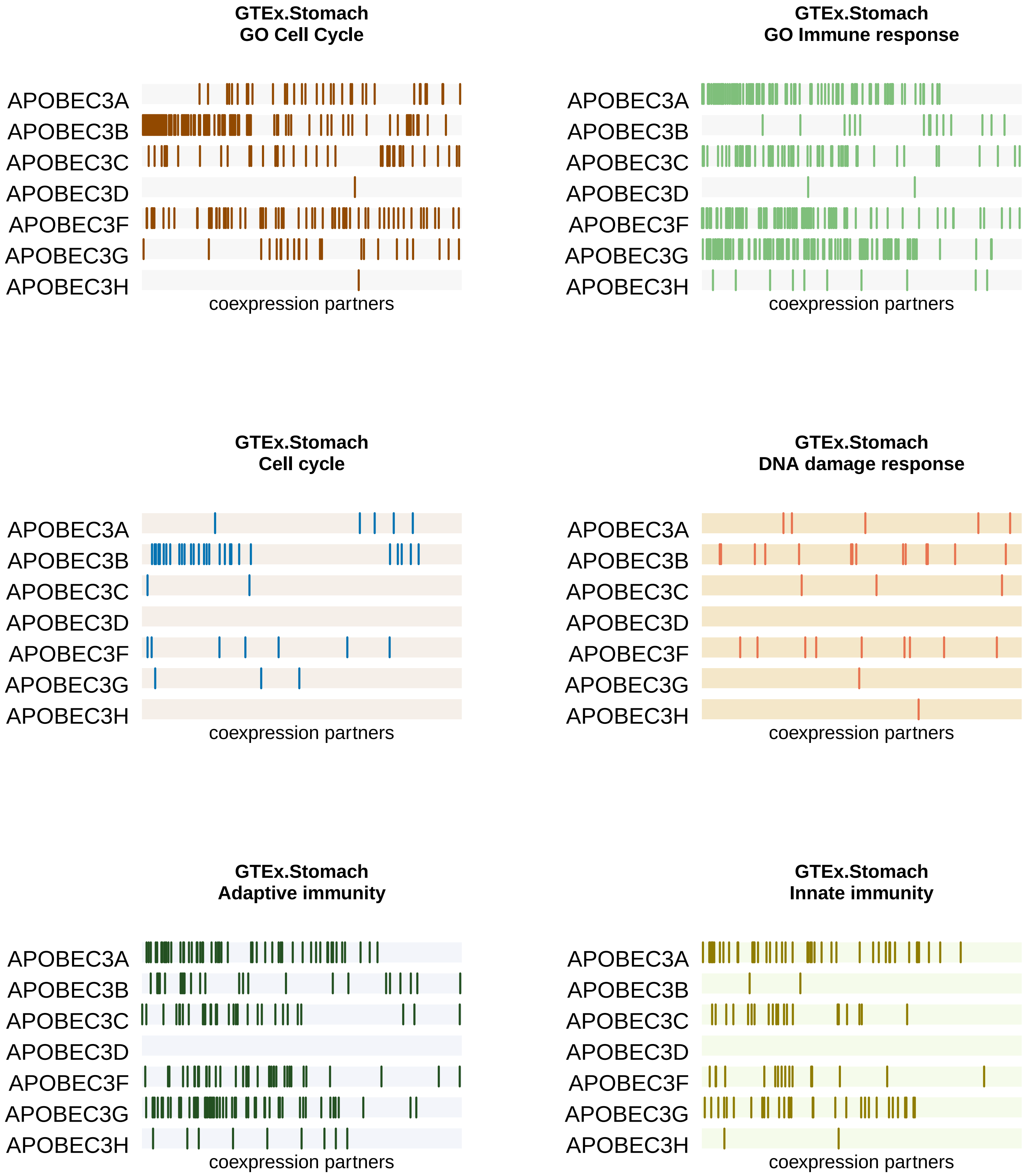
GTEx.Stomach
GO Cell Cycle
APOBEC3A
APOBEC3B
APOBEC3C
APOBEC3D
APOBEC3F
APOBEC3G
APOBEC3H
coexpression partners
GTEx.Stomach
GO Immune response
APOBEC3A
APOBEC3B
APOBEC3C
APOBEC3D
APOBEC3F
APOBEC3G
APOBEC3H
coexpression partners
GTEx.Stomach
Cell cycle
APOBEC3A
APOBEC3B
APOBEC3C
APOBEC3D
APOBEC3F
APOBEC3G
APOBEC3H
coexpression partners
GTEx.Stomach
DNA damage response
APOBEC3A
APOBEC3B
APOBEC3C
APOBEC3D
APOBEC3F
APOBEC3G
APOBEC3H
coexpression partners
GTEx.Stomach
Adaptive immunity
APOBEC3A
APOBEC3B
APOBEC3C
APOBEC3D
APOBEC3F
APOBEC3G
APOBEC3H
coexpression partners
GTEx.Stomach
Innate immunity
APOBEC3A
APOBEC3B
APOBEC3C
APOBEC3D
APOBEC3F
APOBEC3G
APOBEC3H
coexpression partners

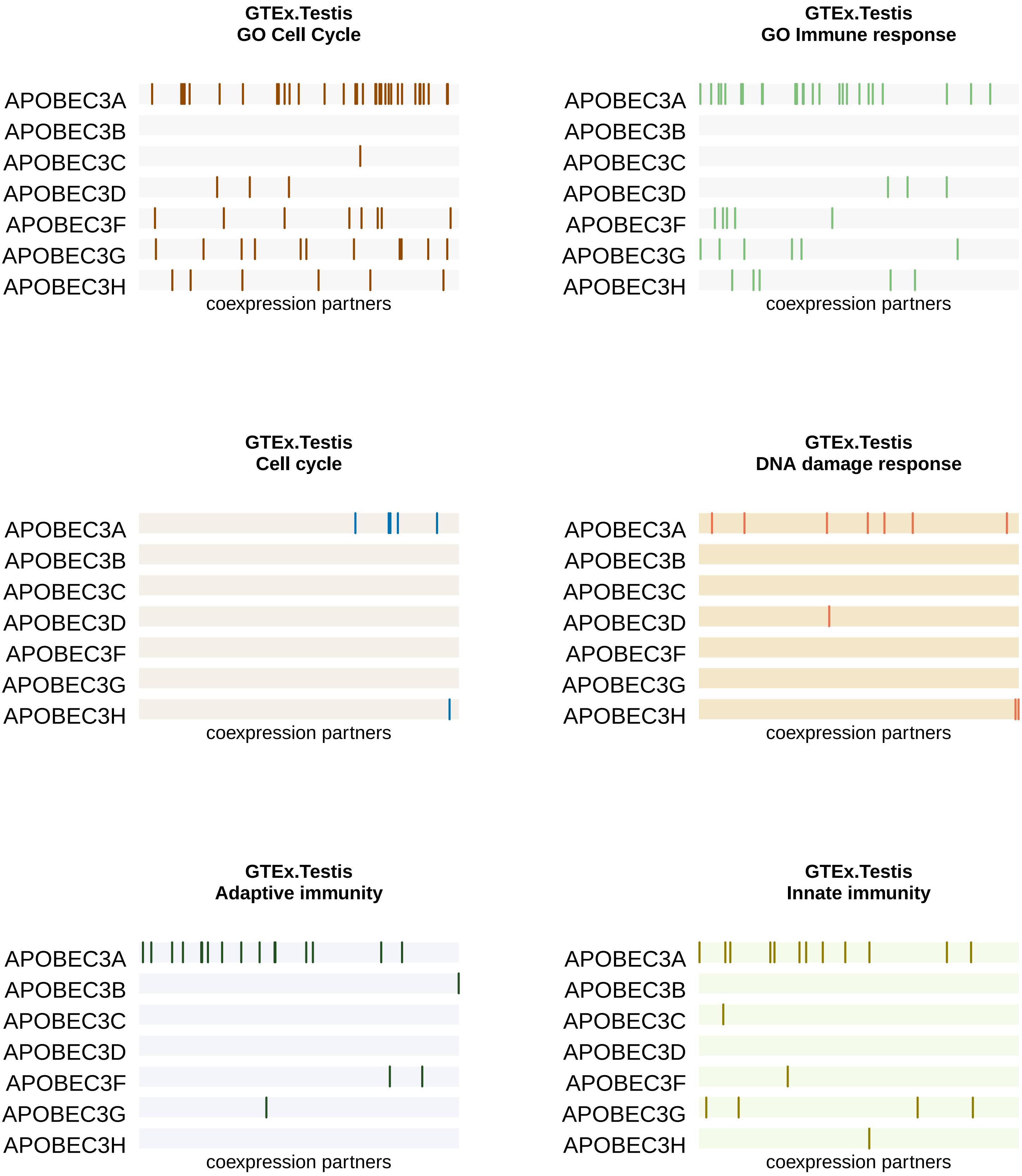
GTEx.Testis
GO Cell Cycle
GTEx.Testis
GO Immune response
GTEx.Testis
Cell cycle
GTEx.Testis
DNA damage response
GTEx.Testis
Adaptive immunity
GTEx.Testis
Innate immunity
APOBEC3A
APOBEC3B
APOBEC3C
APOBEC3D
APOBEC3F
APOBEC3G
APOBEC3H
coexpression partners

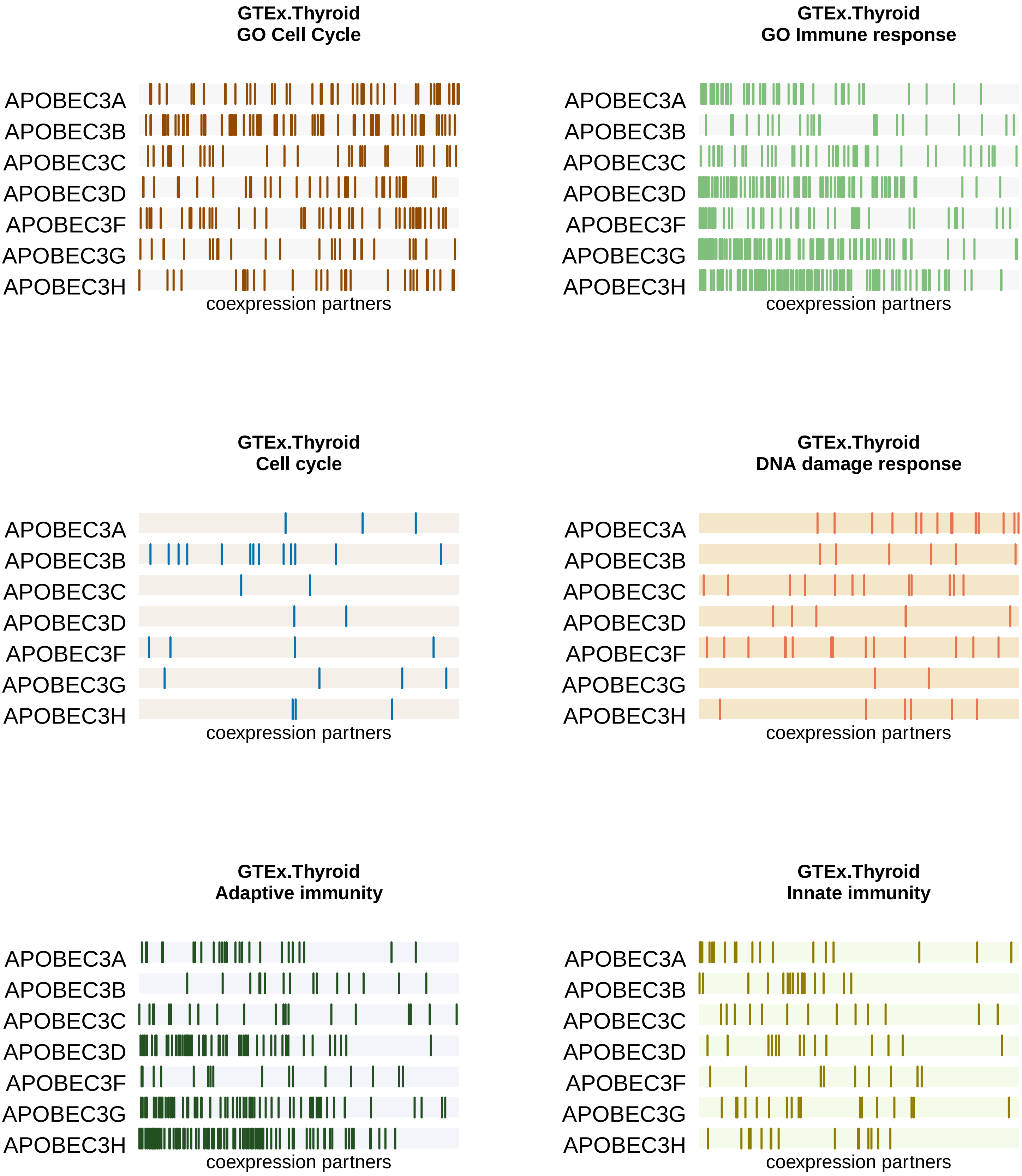
GTEx.Thyroid
GO Cell Cycle
GTEx.Thyroid
GO Immune response
GTEx.Thyroid
Cell cycle
GTEx.Thyroid
DNA damage response
GTEx.Thyroid
Adaptive immunity
GTEx.Thyroid
Innate immunity
APOBEC3A
APOBEC3B
APOBEC3C
APOBEC3D
APOBEC3F
APOBEC3G
APOBEC3H
coexpression partners

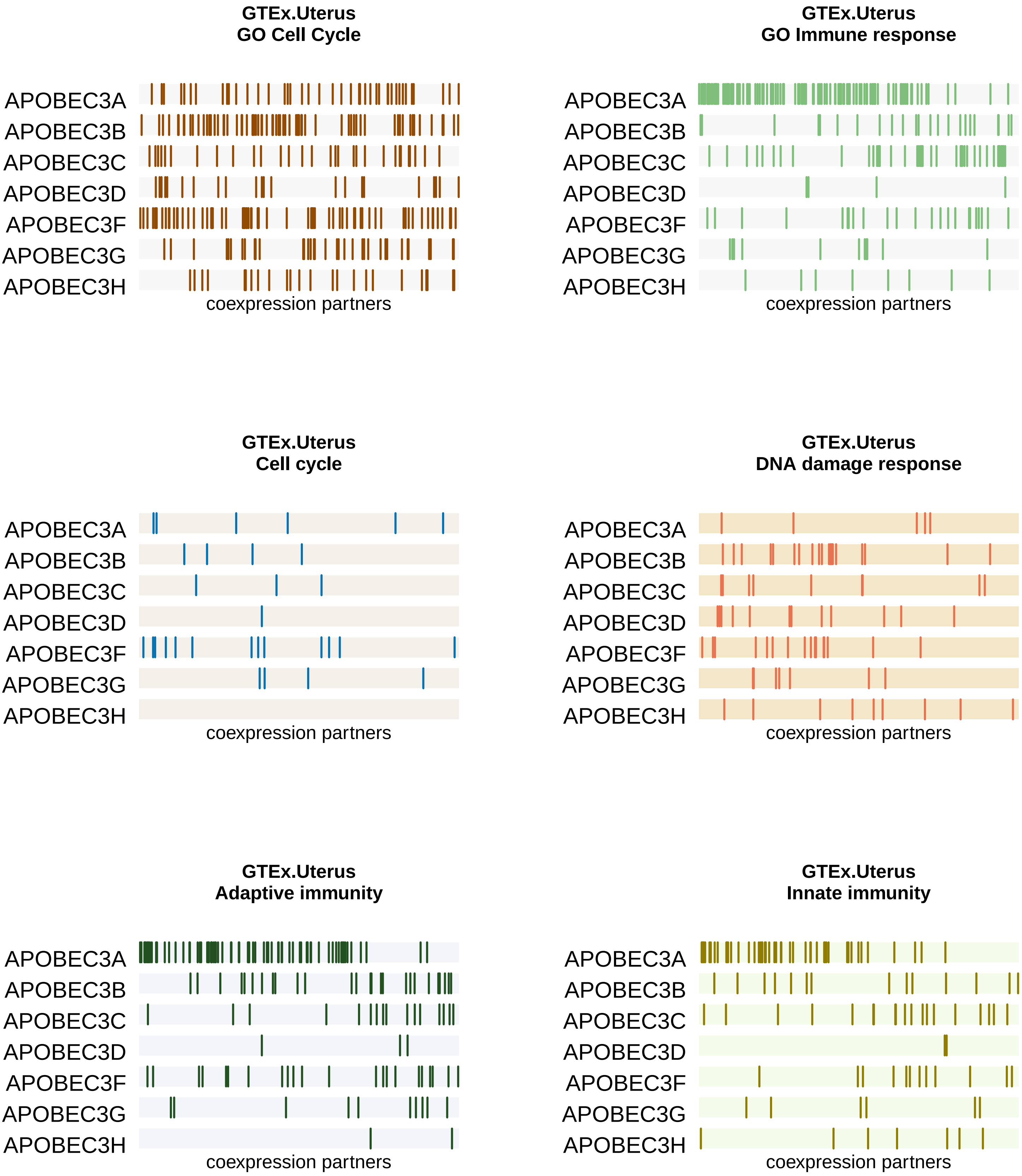
GTEx.Uterus
GO Cell Cycle
APOBEC3A
APOBEC3B
APOBEC3C
APOBEC3D
APOBEC3F
APOBEC3G
APOBEC3H
coexpression partners
GTEx.Uterus
GO Immune response
APOBEC3A
APOBEC3B
APOBEC3C
APOBEC3D
APOBEC3F
APOBEC3G
APOBEC3H
coexpression partners
GTEx.Uterus
Cell cycle
APOBEC3A
APOBEC3B
APOBEC3C
APOBEC3D
APOBEC3F
APOBEC3G
APOBEC3H
coexpression partners
GTEx.Uterus
DNA damage response
APOBEC3A
APOBEC3B
APOBEC3C
APOBEC3D
APOBEC3F
APOBEC3G
APOBEC3H
coexpression partners
GTEx.Uterus
Adaptive immunity
APOBEC3A
APOBEC3B
APOBEC3C
APOBEC3D
APOBEC3F
APOBEC3G
APOBEC3H
coexpression partners
GTEx.Uterus
Innate immunity
APOBEC3A
APOBEC3B
APOBEC3C
APOBEC3D
APOBEC3F
APOBEC3G
APOBEC3H
coexpression partners

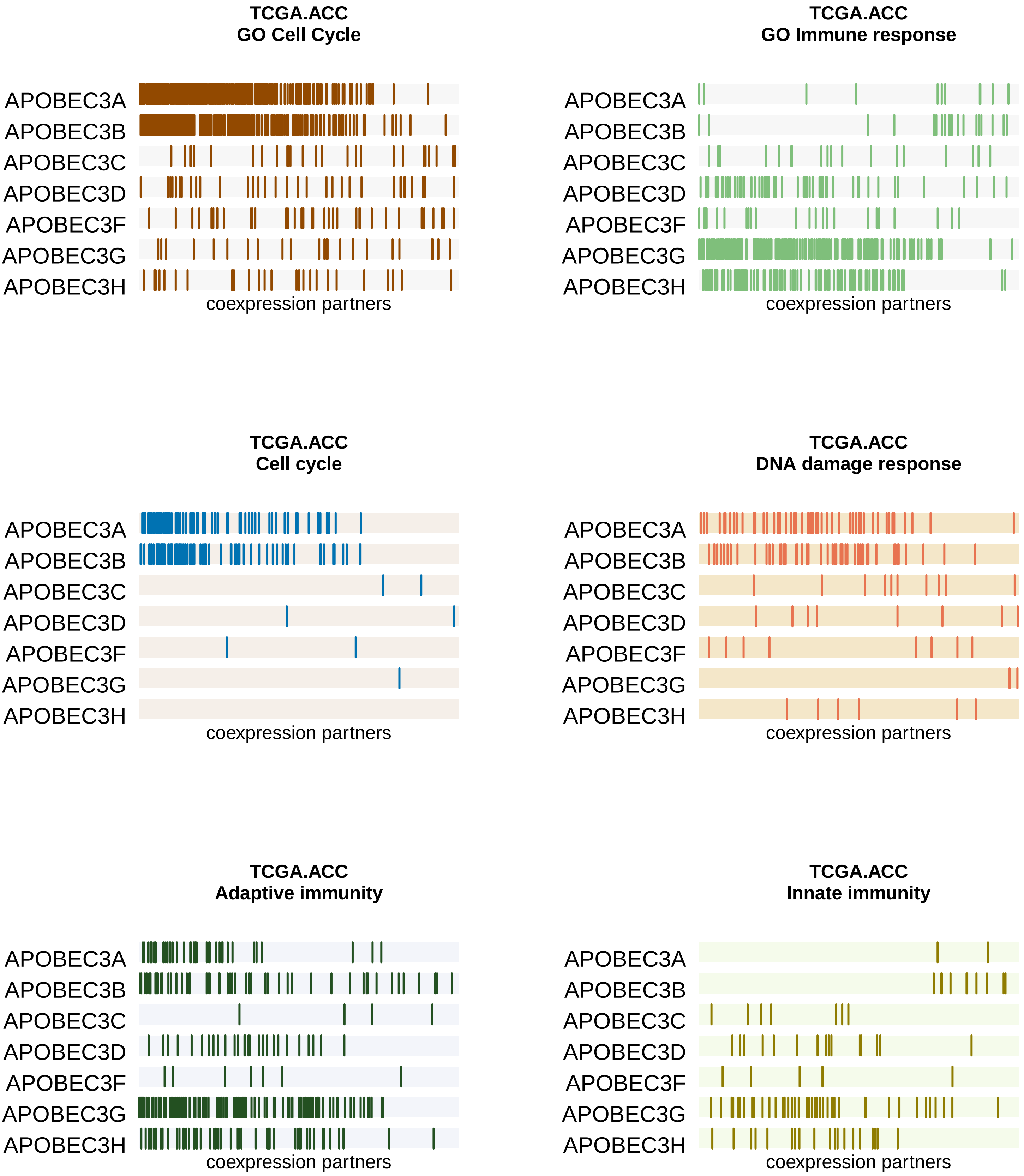
TCGA.ACC
GO Cell Cycle
TCGA.ACC
GO Immune response
TCGA.ACC
Cell cycle
TCGA.ACC
DNA damage response
TCGA.ACC
Adaptive immunity
TCGA.ACC
Innate immunity
APOBEC3A
APOBEC3B
APOBEC3C
APOBEC3D
APOBEC3F
APOBEC3G
APOBEC3H
coexpression partners

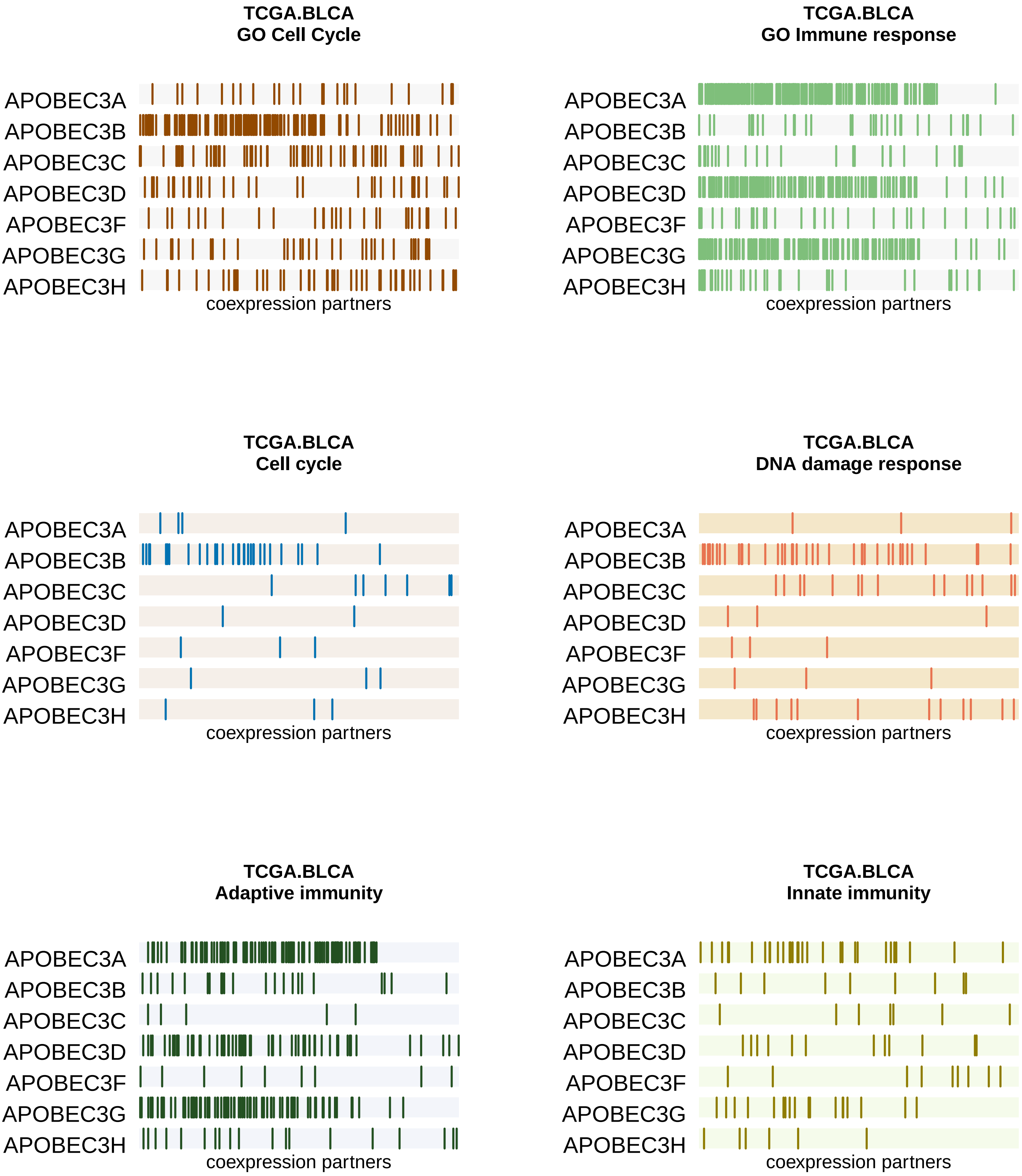
TCGA.BLCA
GO Cell Cycle
APOBEC3A
APOBEC3B
APOBEC3C
APOBEC3D
APOBEC3F
APOBEC3G
APOBEC3H
coexpression partners
TCGA.BLCA
GO Immune response
APOBEC3A
APOBEC3B
APOBEC3C
APOBEC3D
APOBEC3F
APOBEC3G
APOBEC3H
coexpression partners
TCGA.BLCA
Cell cycle
APOBEC3A
APOBEC3B
APOBEC3C
APOBEC3D
APOBEC3F
APOBEC3G
APOBEC3H
coexpression partners
TCGA.BLCA
DNA damage response
APOBEC3A
APOBEC3B
APOBEC3C
APOBEC3D
APOBEC3F
APOBEC3G
APOBEC3H
coexpression partners
TCGA.BLCA
Adaptive immunity
APOBEC3A
APOBEC3B
APOBEC3C
APOBEC3D
APOBEC3F
APOBEC3G
APOBEC3H
coexpression partners
TCGA.BLCA
Innate immunity
APOBEC3A
APOBEC3B
APOBEC3C
APOBEC3D
APOBEC3F
APOBEC3G
APOBEC3H
coexpression partners

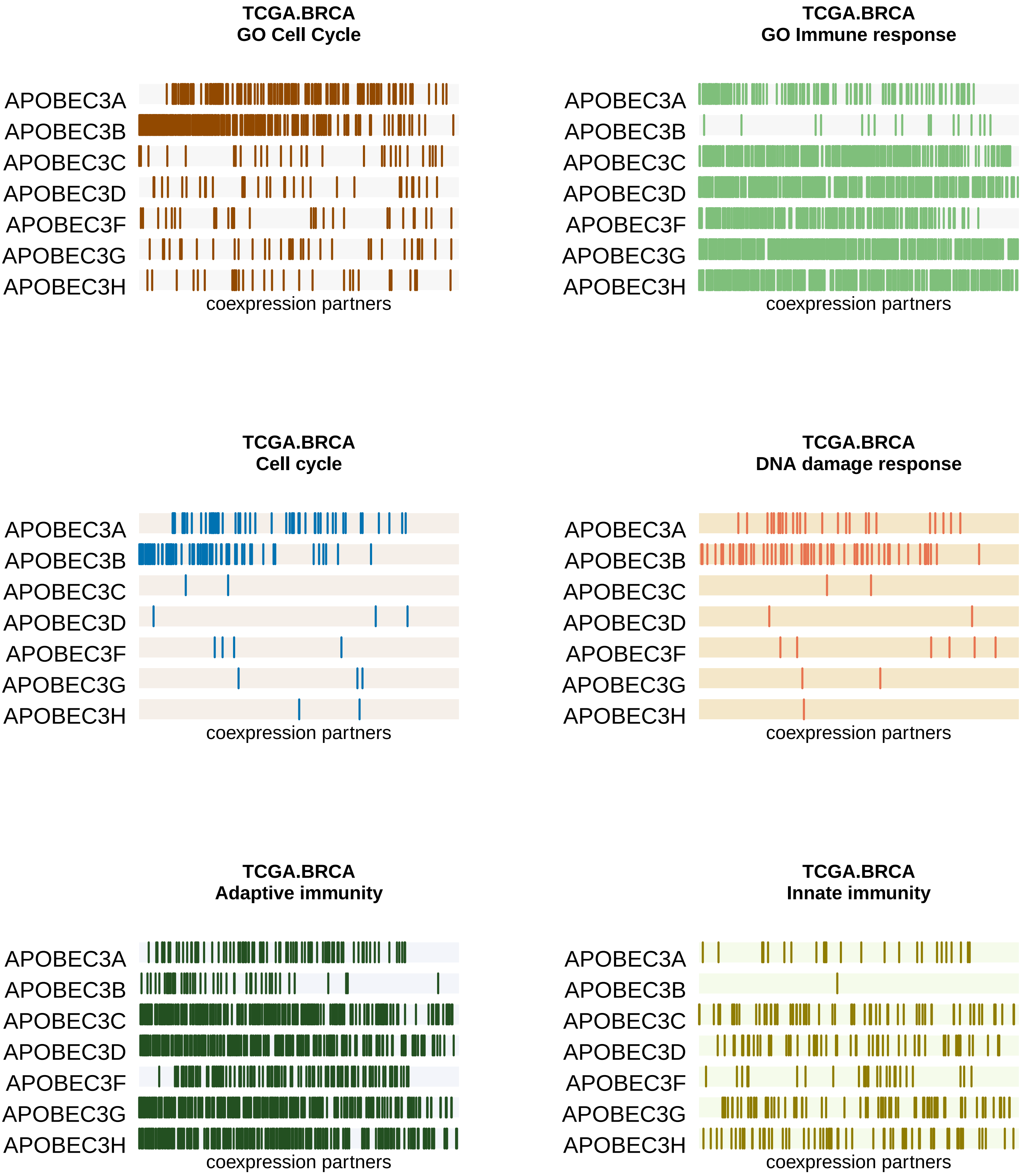
TCGA.BRCA
GO Cell Cycle
APOBEC3A
APOBEC3B
APOBEC3C
APOBEC3D
APOBEC3F
APOBEC3G
APOBEC3H
coexpression partners
TCGA.BRCA
GO Immune response
APOBEC3A
APOBEC3B
APOBEC3C
APOBEC3D
APOBEC3F
APOBEC3G
APOBEC3H
coexpression partners
TCGA.BRCA
Cell cycle
APOBEC3A
APOBEC3B
APOBEC3C
APOBEC3D
APOBEC3F
APOBEC3G
APOBEC3H
coexpression partners
TCGA.BRCA
DNA damage response
APOBEC3A
APOBEC3B
APOBEC3C
APOBEC3D
APOBEC3F
APOBEC3G
APOBEC3H
coexpression partners
TCGA.BRCA
Adaptive immunity
APOBEC3A
APOBEC3B
APOBEC3C
APOBEC3D
APOBEC3F
APOBEC3G
APOBEC3H
coexpression partners
TCGA.BRCA
Innate immunity
APOBEC3A
APOBEC3B
APOBEC3C
APOBEC3D
APOBEC3F
APOBEC3G
APOBEC3H
coexpression partners

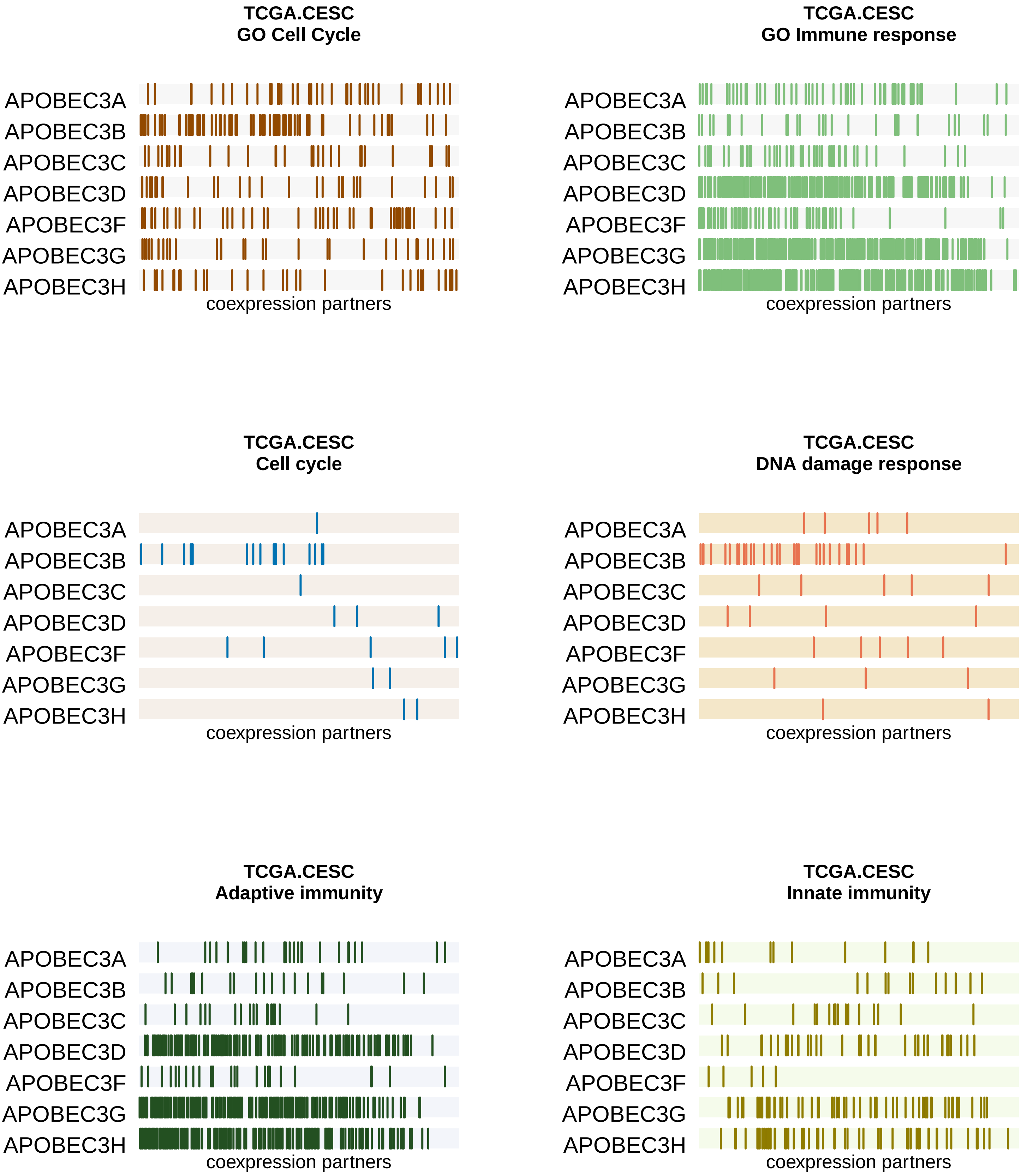
TCGA.CESC
GO Cell Cycle
APOBEC3A
APOBEC3B
APOBEC3C
APOBEC3D
APOBEC3F
APOBEC3G
APOBEC3H
coexpression partners
TCGA.CESC
GO Immune response
APOBEC3A
APOBEC3B
APOBEC3C
APOBEC3D
APOBEC3F
APOBEC3G
APOBEC3H
coexpression partners
TCGA.CESC
Cell cycle
APOBEC3A
APOBEC3B
APOBEC3C
APOBEC3D
APOBEC3F
APOBEC3G
APOBEC3H
coexpression partners
TCGA.CESC
DNA damage response
APOBEC3A
APOBEC3B
APOBEC3C
APOBEC3D
APOBEC3F
APOBEC3G
APOBEC3H
coexpression partners
TCGA.CESC
Adaptive immunity
APOBEC3A
APOBEC3B
APOBEC3C
APOBEC3D
APOBEC3F
APOBEC3G
APOBEC3H
coexpression partners
TCGA.CESC
Innate immunity
APOBEC3A
APOBEC3B
APOBEC3C
APOBEC3D
APOBEC3F
APOBEC3G
APOBEC3H
coexpression partners

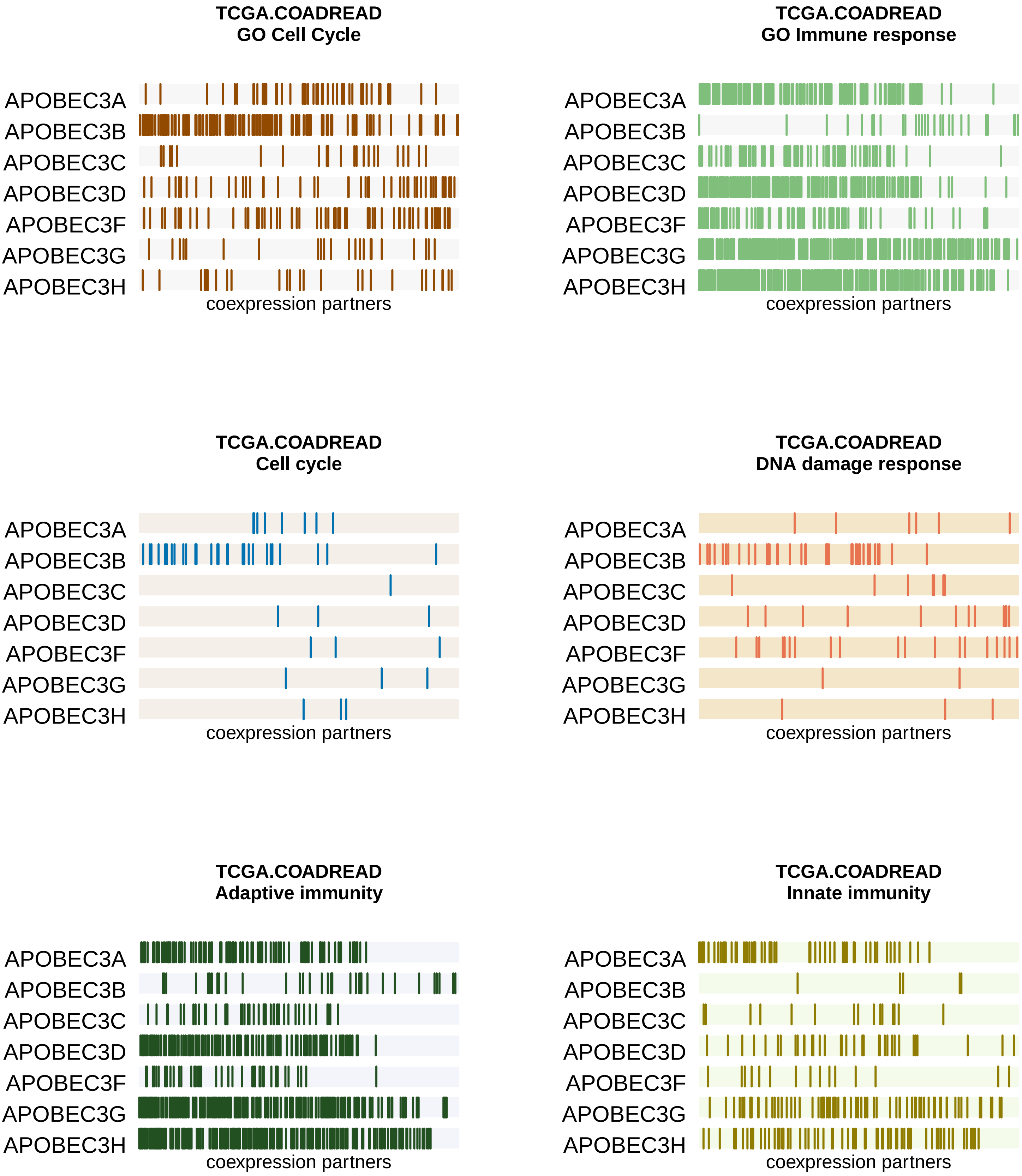
TCGA.COADREAD
GO Cell Cycle
APOBEC3A
APOBEC3B
APOBEC3C
APOBEC3D
APOBEC3F
APOBEC3G
APOBEC3H
coexpression partners
TCGA.COADREAD
GO Immune response
APOBEC3A
APOBEC3B
APOBEC3C
APOBEC3D
APOBEC3F
APOBEC3G
APOBEC3H
coexpression partners
TCGA.COADREAD
Cell cycle
APOBEC3A
APOBEC3B
APOBEC3C
APOBEC3D
APOBEC3F
APOBEC3G
APOBEC3H
coexpression partners
TCGA.COADREAD
DNA damage response
APOBEC3A
APOBEC3B
APOBEC3C
APOBEC3D
APOBEC3F
APOBEC3G
APOBEC3H
coexpression partners
TCGA.COADREAD
Adaptive immunity
APOBEC3A
APOBEC3B
APOBEC3C
APOBEC3D
APOBEC3F
APOBEC3G
APOBEC3H
coexpression partners
TCGA.COADREAD
Innate immunity
APOBEC3A
APOBEC3B
APOBEC3C
APOBEC3D
APOBEC3F
APOBEC3G
APOBEC3H
coexpression partners

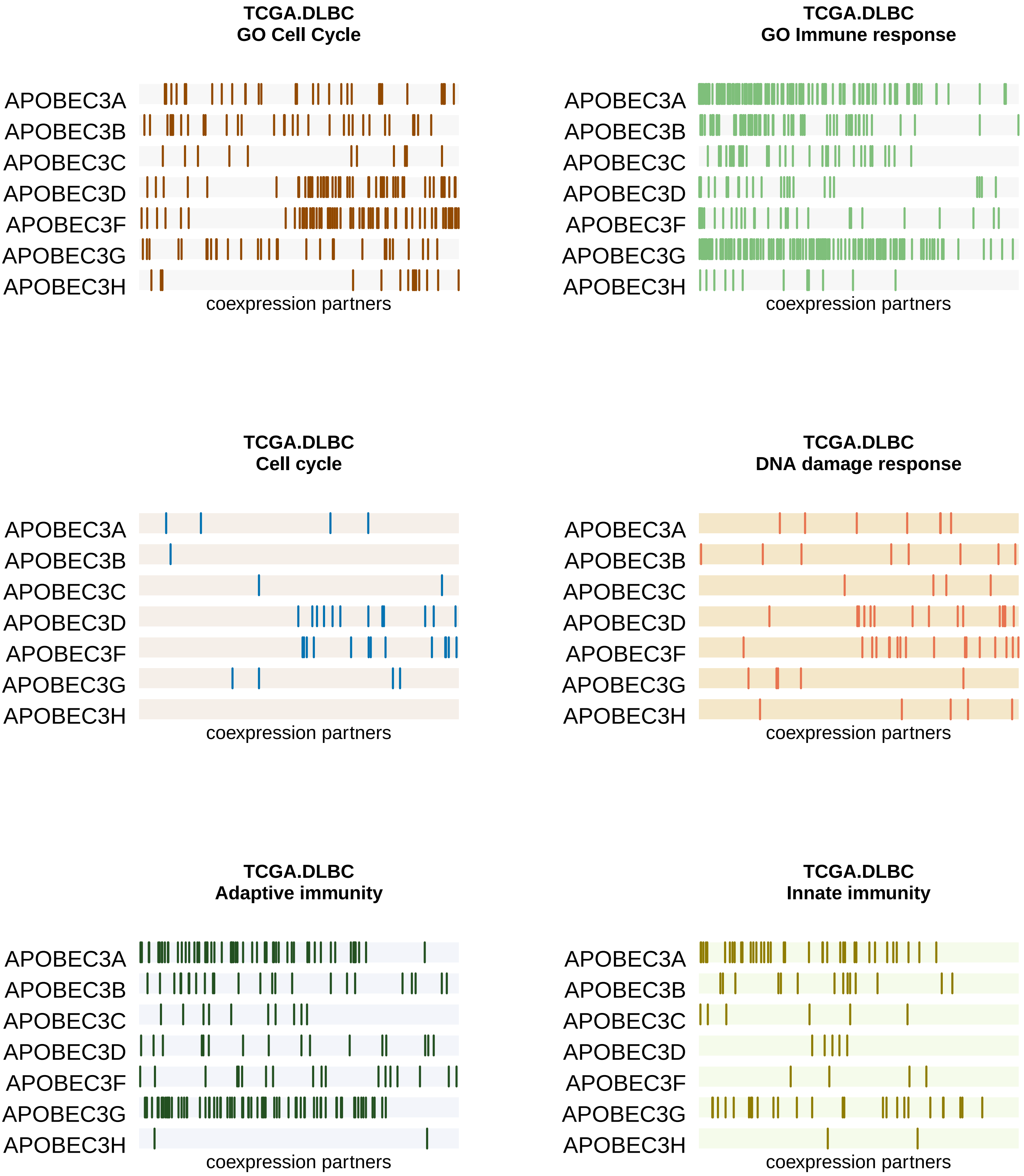

TCGA.DLBC
GO Cell Cycle
APOBEC3A
APOBEC3B
APOBEC3C
APOBEC3D
APOBEC3F
APOBEC3G
APOBEC3H
coexpression partners
TCGA.DLBC
GO Immune response
APOBEC3A
APOBEC3B
APOBEC3C
APOBEC3D
APOBEC3F
APOBEC3G
APOBEC3H
coexpression partners
TCGA.DLBC
Cell cycle
APOBEC3A
APOBEC3B
APOBEC3C
APOBEC3D
APOBEC3F
APOBEC3G
APOBEC3H
coexpression partners
TCGA.DLBC
DNA damage response
APOBEC3A
APOBEC3B
APOBEC3C
APOBEC3D
APOBEC3F
APOBEC3G
APOBEC3H
coexpression partners
TCGA.DLBC
Adaptive immunity
APOBEC3A
APOBEC3B
APOBEC3C
APOBEC3D
APOBEC3F
APOBEC3G
APOBEC3H
coexpression partners
TCGA.DLBC
Innate immunity
APOBEC3A
APOBEC3B
APOBEC3C
APOBEC3D
APOBEC3F
APOBEC3G
APOBEC3H
coexpression partners

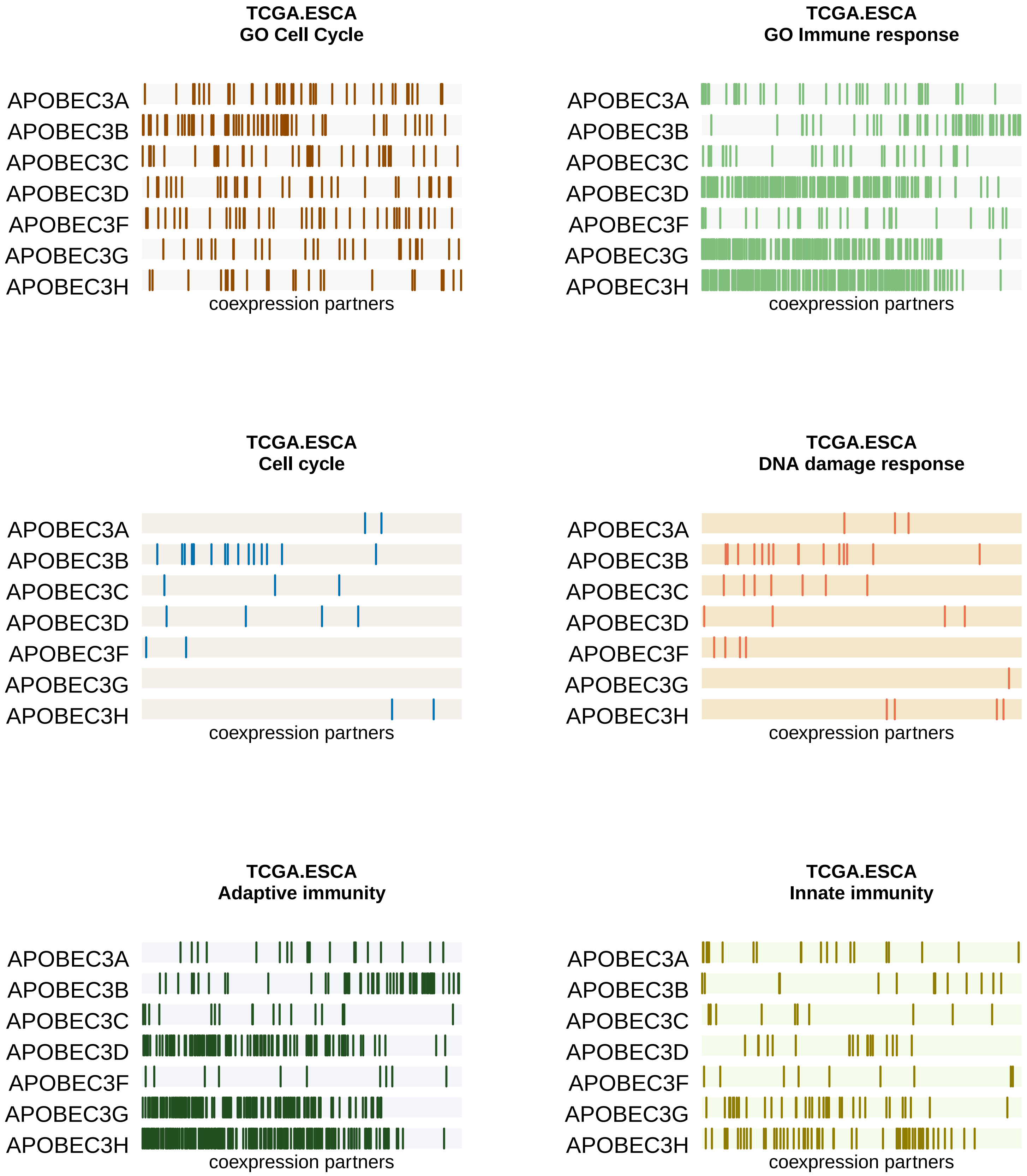
TCGA.ESCA
GO Cell Cycle
APOBEC3A
APOBEC3B
APOBEC3C
APOBEC3D
APOBEC3F
APOBEC3G
APOBEC3H
coexpression partners
TCGA.ESCA
GO Immune response
APOBEC3A
APOBEC3B
APOBEC3C
APOBEC3D
APOBEC3F
APOBEC3G
APOBEC3H
coexpression partners
TCGA.ESCA
Cell cycle
APOBEC3A
APOBEC3B
APOBEC3C
APOBEC3D
APOBEC3F
APOBEC3G
APOBEC3H
coexpression partners
TCGA.ESCA
DNA damage response
APOBEC3A
APOBEC3B
APOBEC3C
APOBEC3D
APOBEC3F
APOBEC3G
APOBEC3H
coexpression partners
TCGA.ESCA
Adaptive immunity
APOBEC3A
APOBEC3B
APOBEC3C
APOBEC3D
APOBEC3F
APOBEC3G
APOBEC3H
coexpression partners
TCGA.ESCA
Innate immunity
APOBEC3A
APOBEC3B
APOBEC3C
APOBEC3D
APOBEC3F
APOBEC3G
APOBEC3H
coexpression partners

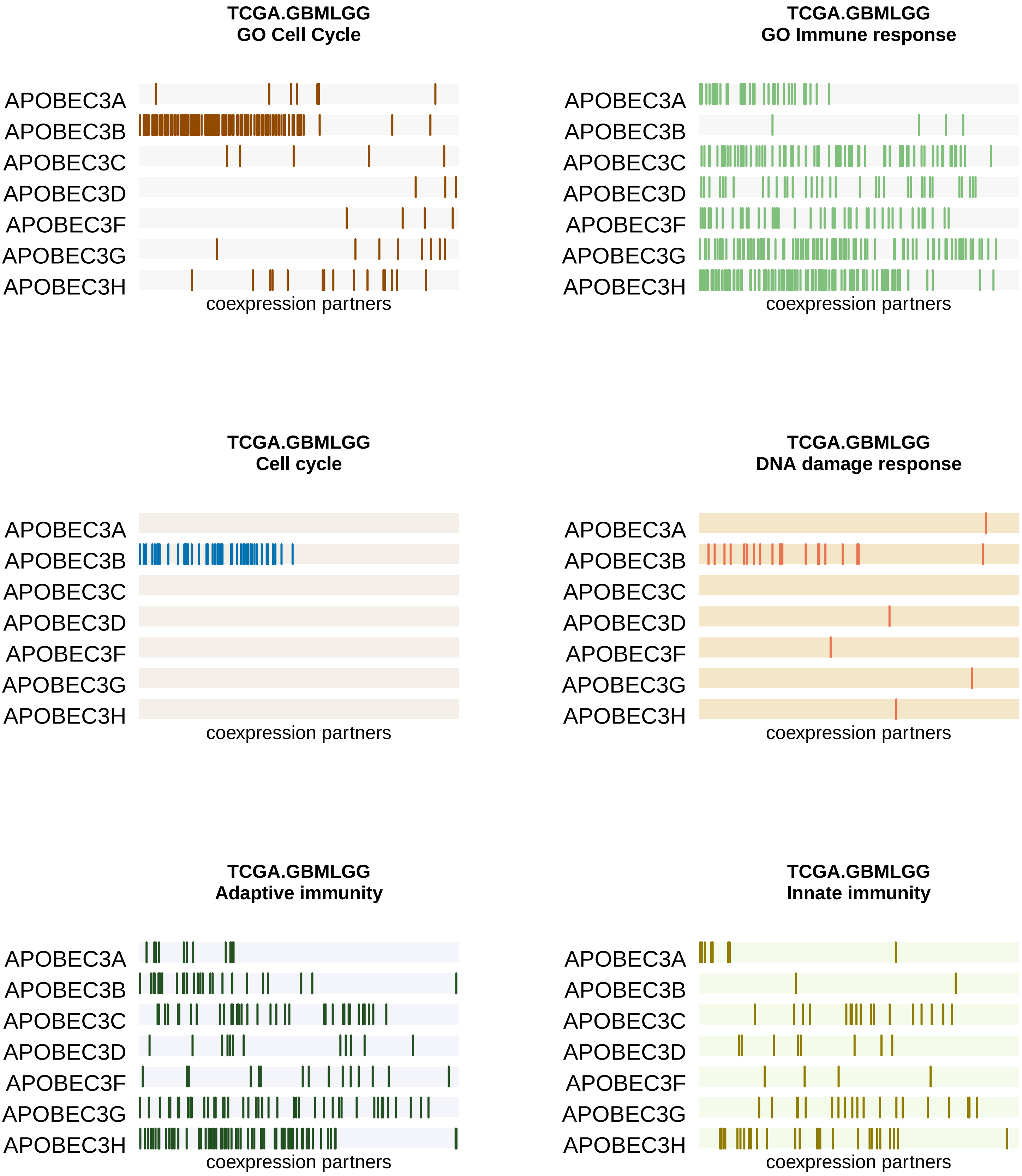
TCGA.GBMLGG
GO Cell Cycle
TCGA.GBMLGG
GO Immune response
TCGA.GBMLGG
Cell cycle
TCGA.GBMLGG
DNA damage response
TCGA.GBMLGG
Adaptive immunity
TCGA.GBMLGG
Innate immunity
APOBEC3A
APOBEC3B
APOBEC3C
APOBEC3D
APOBEC3F
APOBEC3G
APOBEC3H
coexpression partners

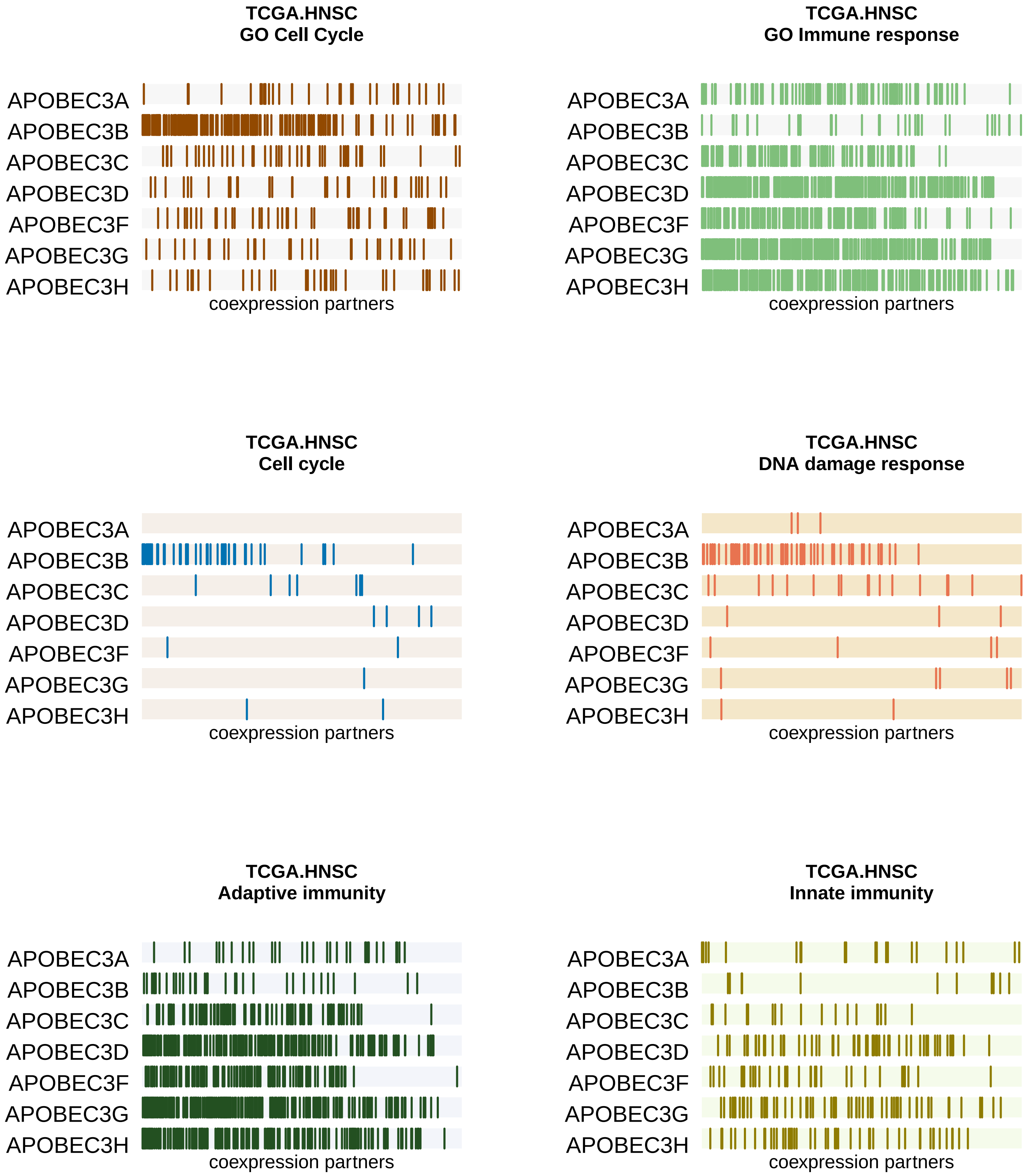
TCGA.HNSC
GO Cell Cycle
APOBEC3A
APOBEC3B
APOBEC3C
APOBEC3D
APOBEC3F
APOBEC3G
APOBEC3H
coexpression partners
TCGA.HNSC
GO Immune response
APOBEC3A
APOBEC3B
APOBEC3C
APOBEC3D
APOBEC3F
APOBEC3G
APOBEC3H
coexpression partners
TCGA.HNSC
Cell cycle
APOBEC3A
APOBEC3B
APOBEC3C
APOBEC3D
APOBEC3F
APOBEC3G
APOBEC3H
coexpression partners
TCGA.HNSC
DNA damage response
APOBEC3A
APOBEC3B
APOBEC3C
APOBEC3D
APOBEC3F
APOBEC3G
APOBEC3H
coexpression partners
TCGA.HNSC
Adaptive immunity
APOBEC3A
APOBEC3B
APOBEC3C
APOBEC3D
APOBEC3F
APOBEC3G
APOBEC3H
coexpression partners
TCGA.HNSC
Innate immunity
APOBEC3A
APOBEC3B
APOBEC3C
APOBEC3D
APOBEC3F
APOBEC3G
APOBEC3H
coexpression partners

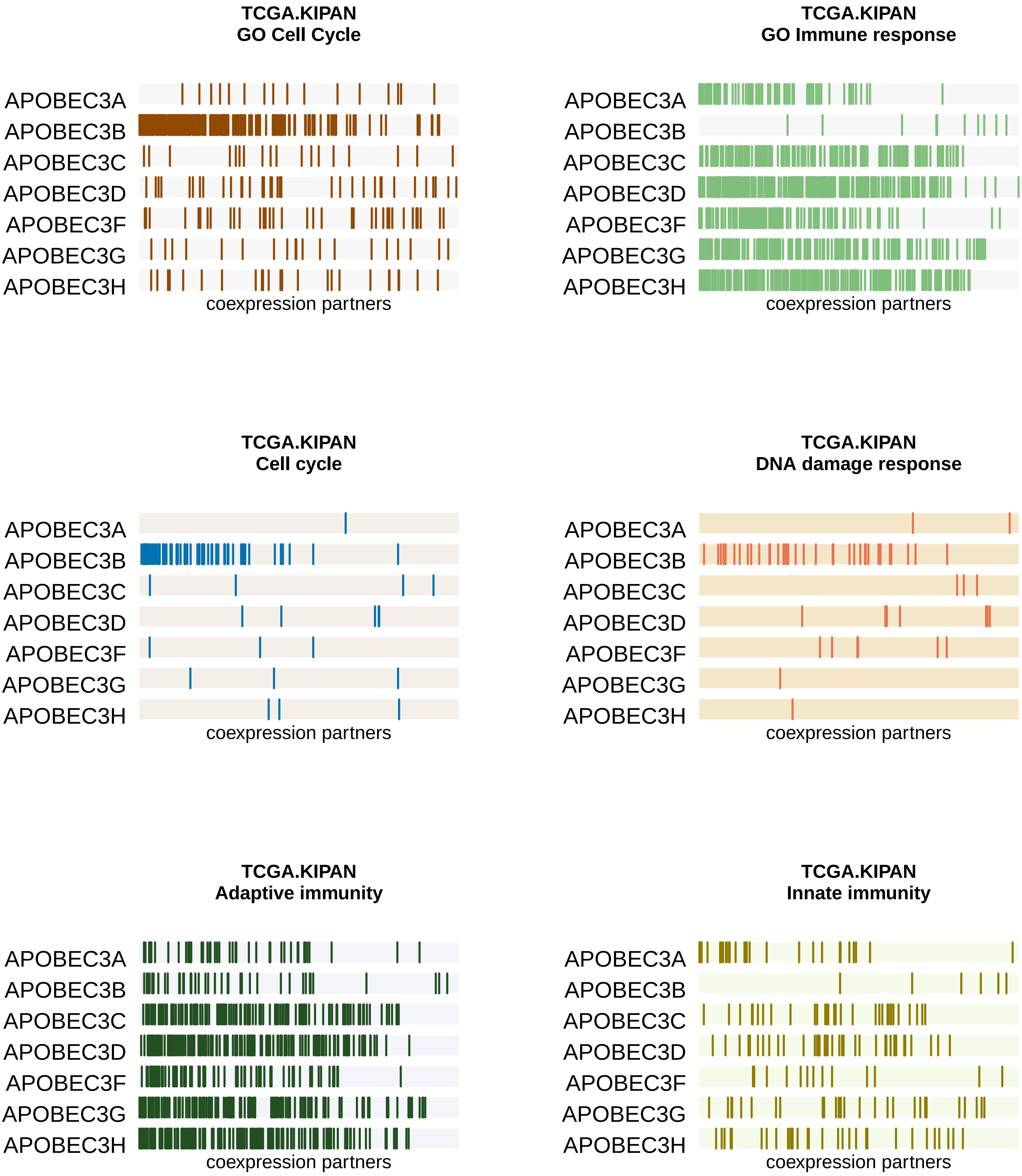
TCGA.KIPAN
GO Cell Cycle
APOBEC3A
APOBEC3B
APOBEC3C
APOBEC3D
APOBEC3F
APOBEC3G
APOBEC3H
coexpression partners
TCGA.KIPAN
GO Immune response
APOBEC3A
APOBEC3B
APOBEC3C
APOBEC3D
APOBEC3F
APOBEC3G
APOBEC3H
coexpression partners
TCGA.KIPAN
Cell cycle
APOBEC3A
APOBEC3B
APOBEC3C
APOBEC3D
APOBEC3F
APOBEC3G
APOBEC3H
coexpression partners
TCGA.KIPAN
DNA damage response
APOBEC3A
APOBEC3B
APOBEC3C
APOBEC3D
APOBEC3F
APOBEC3G
APOBEC3H
coexpression partners
TCGA.KIPAN
Adaptive immunity
APOBEC3A
APOBEC3B
APOBEC3C
APOBEC3D
APOBEC3F
APOBEC3G
APOBEC3H
coexpression partners
TCGA.KIPAN
Innate immunity
APOBEC3A
APOBEC3B
APOBEC3C
APOBEC3D
APOBEC3F
APOBEC3G
APOBEC3H
coexpression partners

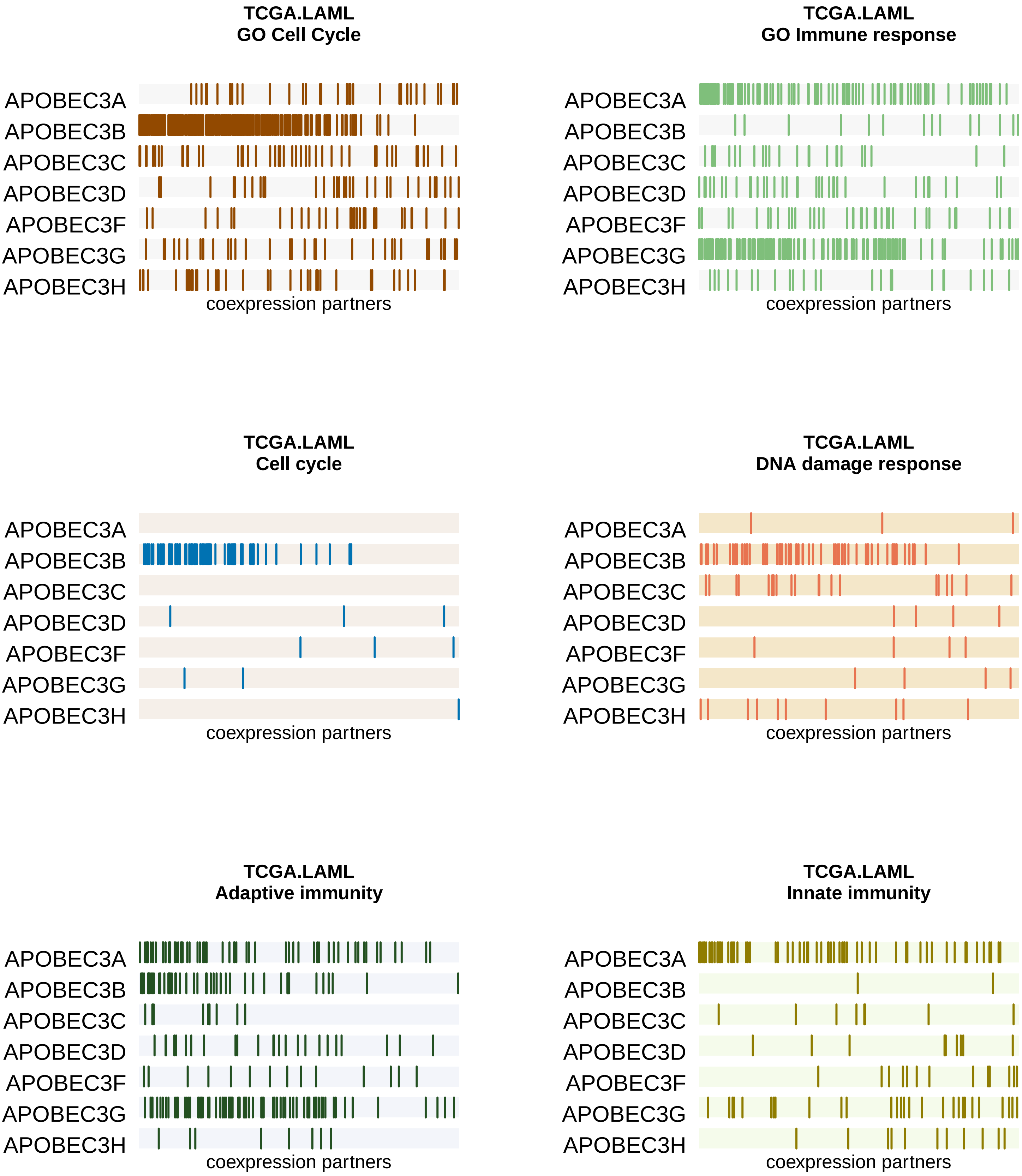

TCGA.LAML
GO Cell Cycle
APOBEC3A
APOBEC3B
APOBEC3C
APOBEC3D
APOBEC3F
APOBEC3G
APOBEC3H
coexpression partners
TCGA.LAML
GO Immune response
APOBEC3A
APOBEC3B
APOBEC3C
APOBEC3D
APOBEC3F
APOBEC3G
APOBEC3H
coexpression partners
TCGA.LAML
Cell cycle
APOBEC3A
APOBEC3B
APOBEC3C
APOBEC3D
APOBEC3F
APOBEC3G
APOBEC3H
coexpression partners
TCGA.LAML
DNA damage response
APOBEC3A
APOBEC3B
APOBEC3C
APOBEC3D
APOBEC3F
APOBEC3G
APOBEC3H
coexpression partners
TCGA.LAML
Adaptive immunity
APOBEC3A
APOBEC3B
APOBEC3C
APOBEC3D
APOBEC3F
APOBEC3G
APOBEC3H
coexpression partners
TCGA.LAML
Innate immunity
APOBEC3A
APOBEC3B
APOBEC3C
APOBEC3D
APOBEC3F
APOBEC3G
APOBEC3H
coexpression partners

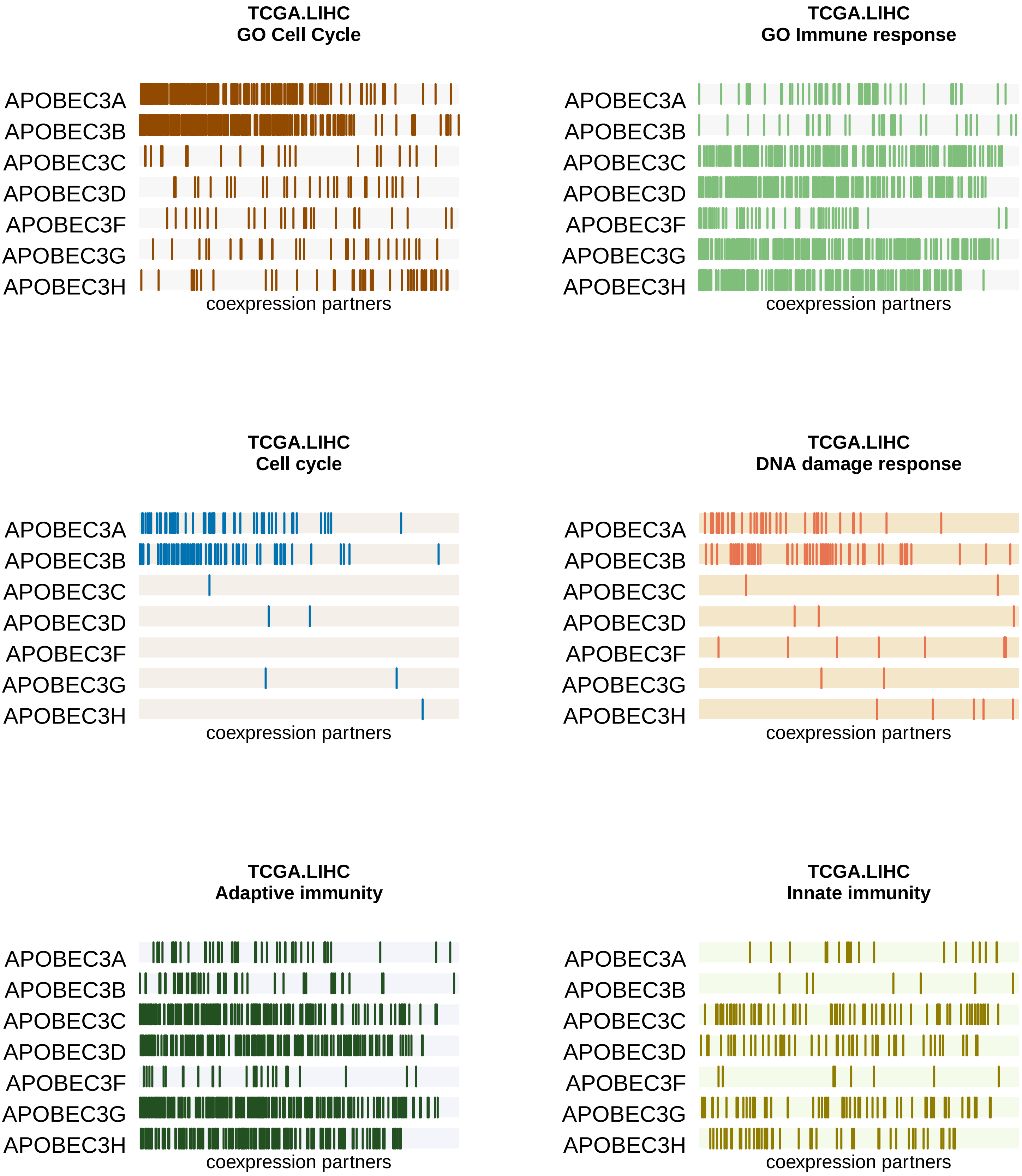
TCGA.LIHC
GO Cell Cycle
TCGA.LIHC
GO Immune response
TCGA.LIHC
Cell cycle
TCGA.LIHC
DNA damage response
TCGA.LIHC
Adaptive immunity
TCGA.LIHC
Innate immunity
APOBEC3A
APOBEC3B
APOBEC3C
APOBEC3D
APOBEC3F
APOBEC3G
APOBEC3H
coexpression partners

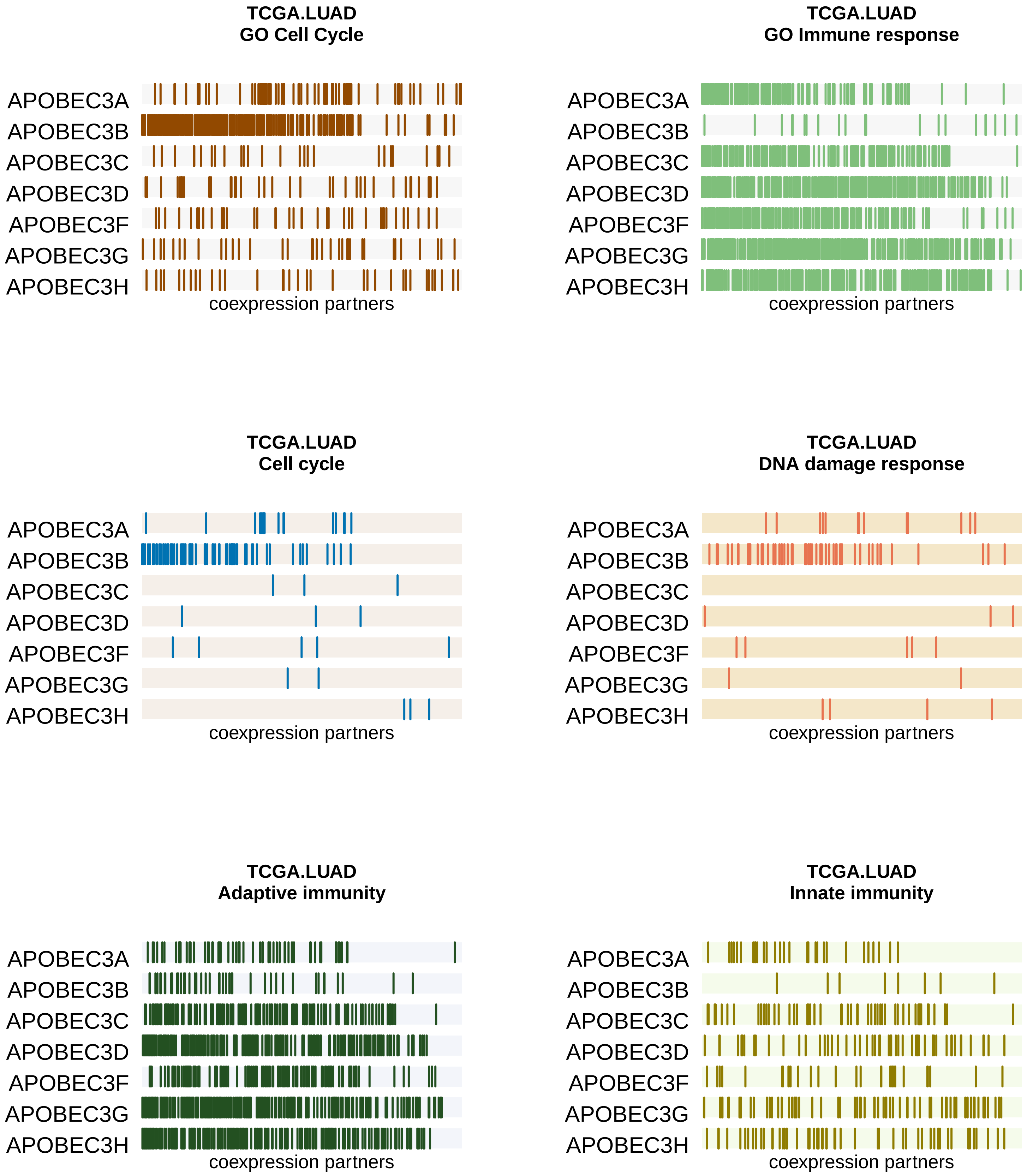
TCGA.LUAD
GO Cell Cycle
TCGA.LUAD
GO Immune response
TCGA.LUAD
Cell cycle
TCGA.LUAD
DNA damage response
TCGA.LUAD
Adaptive immunity
TCGA.LUAD
Innate immunity
APOBEC3A
APOBEC3B
APOBEC3C
APOBEC3D
APOBEC3F
APOBEC3G
APOBEC3H
coexpression partners

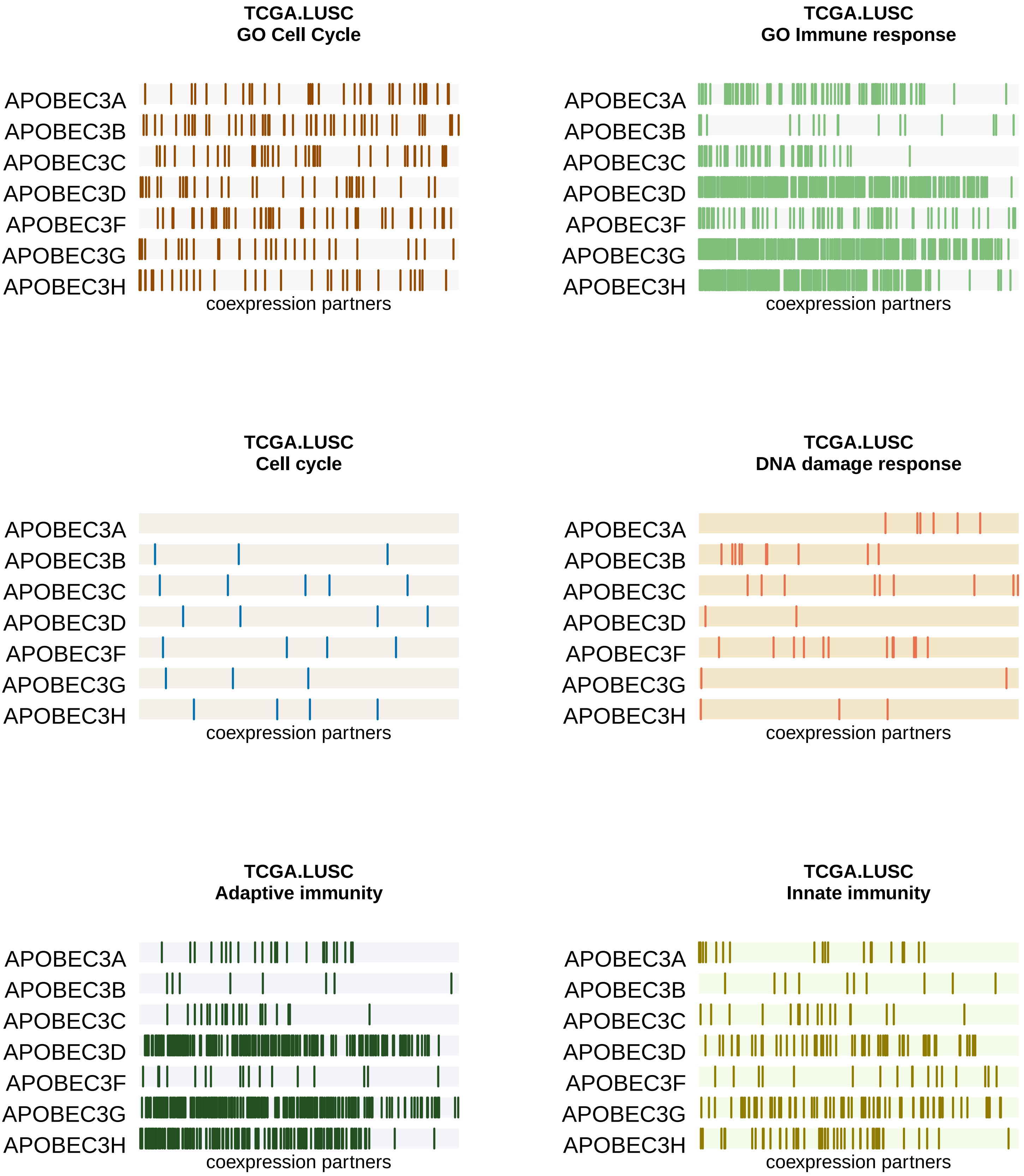
TCGA.LUSC
GO Cell Cycle
TCGA.LUSC
GO Immune response
TCGA.LUSC
Cell cycle
TCGA.LUSC
DNA damage response
TCGA.LUSC
Adaptive immunity
TCGA.LUSC
Innate immunity
APOBEC3A
APOBEC3B
APOBEC3C
APOBEC3D
APOBEC3F
APOBEC3G
APOBEC3H
coexpression partners

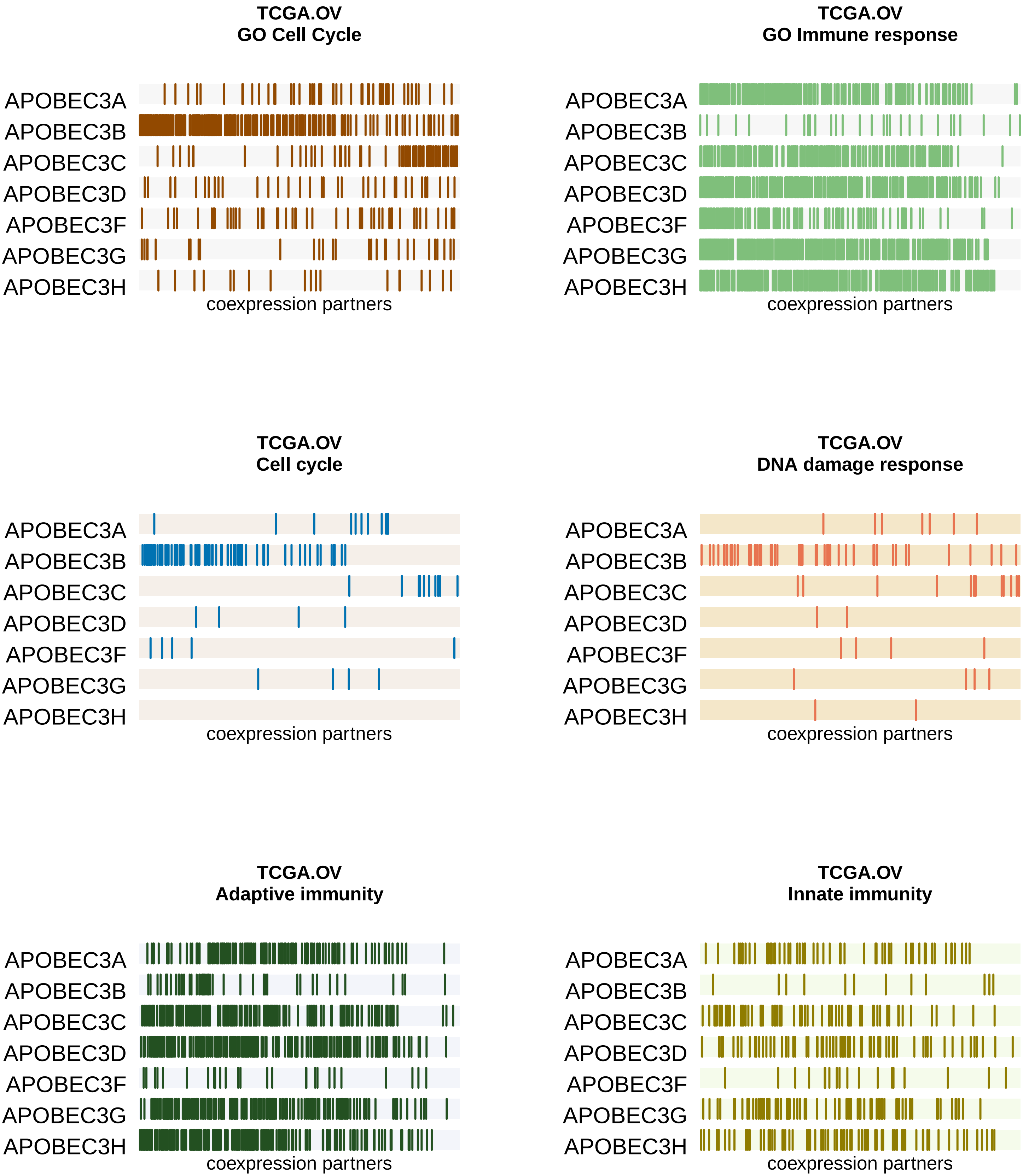

TCGA.OV
GO Cell Cycle
TCGA.OV
GO Immune response
TCGA.OV
Cell cycle
TCGA.OV
DNA damage response
TCGA.OV
Adaptive immunity
TCGA.OV
Innate immunity
APOBEC3A
APOBEC3B
APOBEC3C
APOBEC3D
APOBEC3F
APOBEC3G
APOBEC3H
coexpression partners

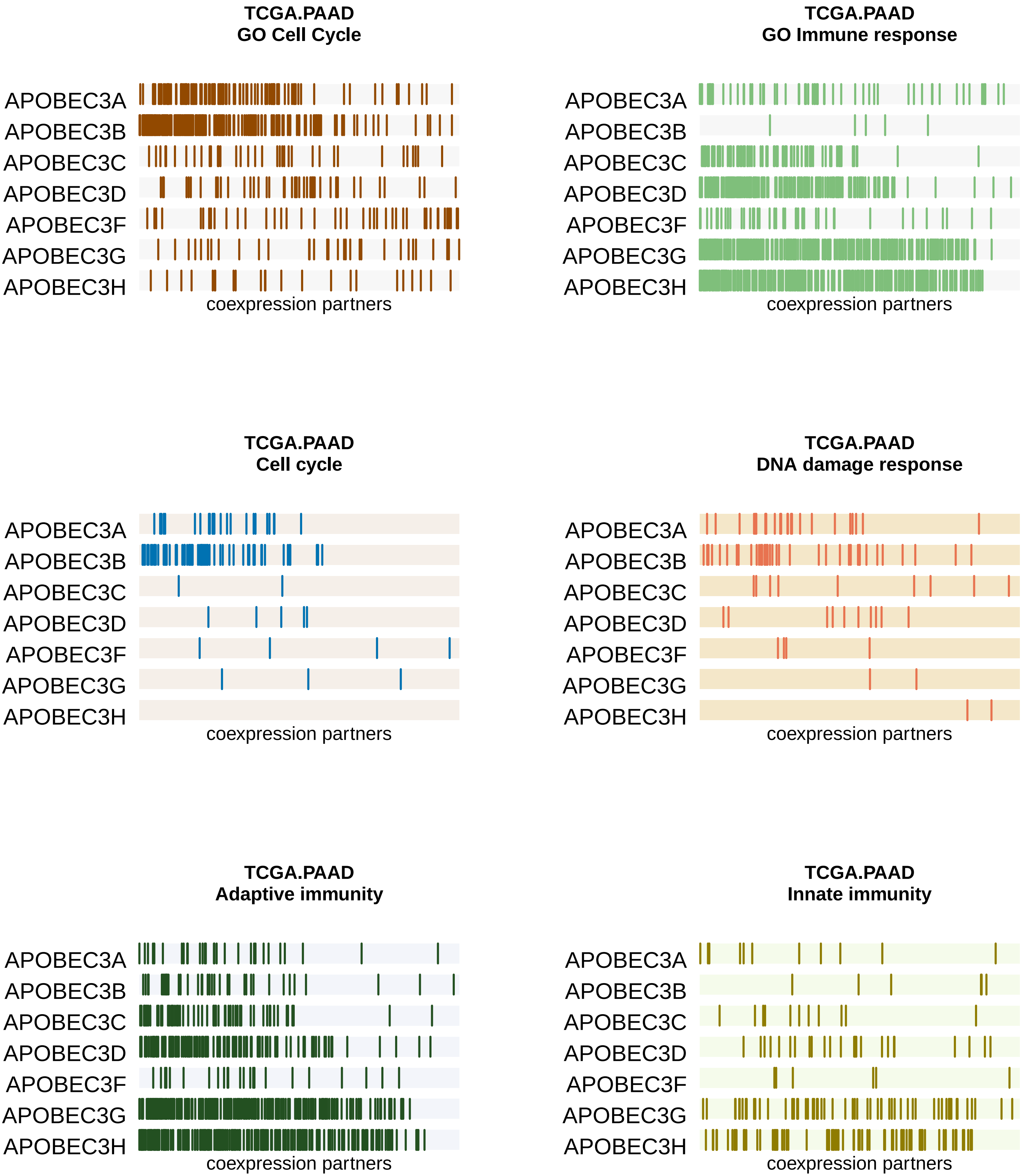
TCGA.PAAD
GO Cell Cycle
APOBEC3A
APOBEC3B
APOBEC3C
APOBEC3D
APOBEC3F
APOBEC3G
APOBEC3H
coexpression partners
TCGA.PAAD
GO Immune response
APOBEC3A
APOBEC3B
APOBEC3C
APOBEC3D
APOBEC3F
APOBEC3G
APOBEC3H
coexpression partners
TCGA.PAAD
Cell cycle
APOBEC3A
APOBEC3B
APOBEC3C
APOBEC3D
APOBEC3F
APOBEC3G
APOBEC3H
coexpression partners
TCGA.PAAD
DNA damage response
APOBEC3A
APOBEC3B
APOBEC3C
APOBEC3D
APOBEC3F
APOBEC3G
APOBEC3H
coexpression partners
TCGA.PAAD
Adaptive immunity
APOBEC3A
APOBEC3B
APOBEC3C
APOBEC3D
APOBEC3F
APOBEC3G
APOBEC3H
coexpression partners
TCGA.PAAD
Innate immunity
APOBEC3A
APOBEC3B
APOBEC3C
APOBEC3D
APOBEC3F
APOBEC3G
APOBEC3H
coexpression partners

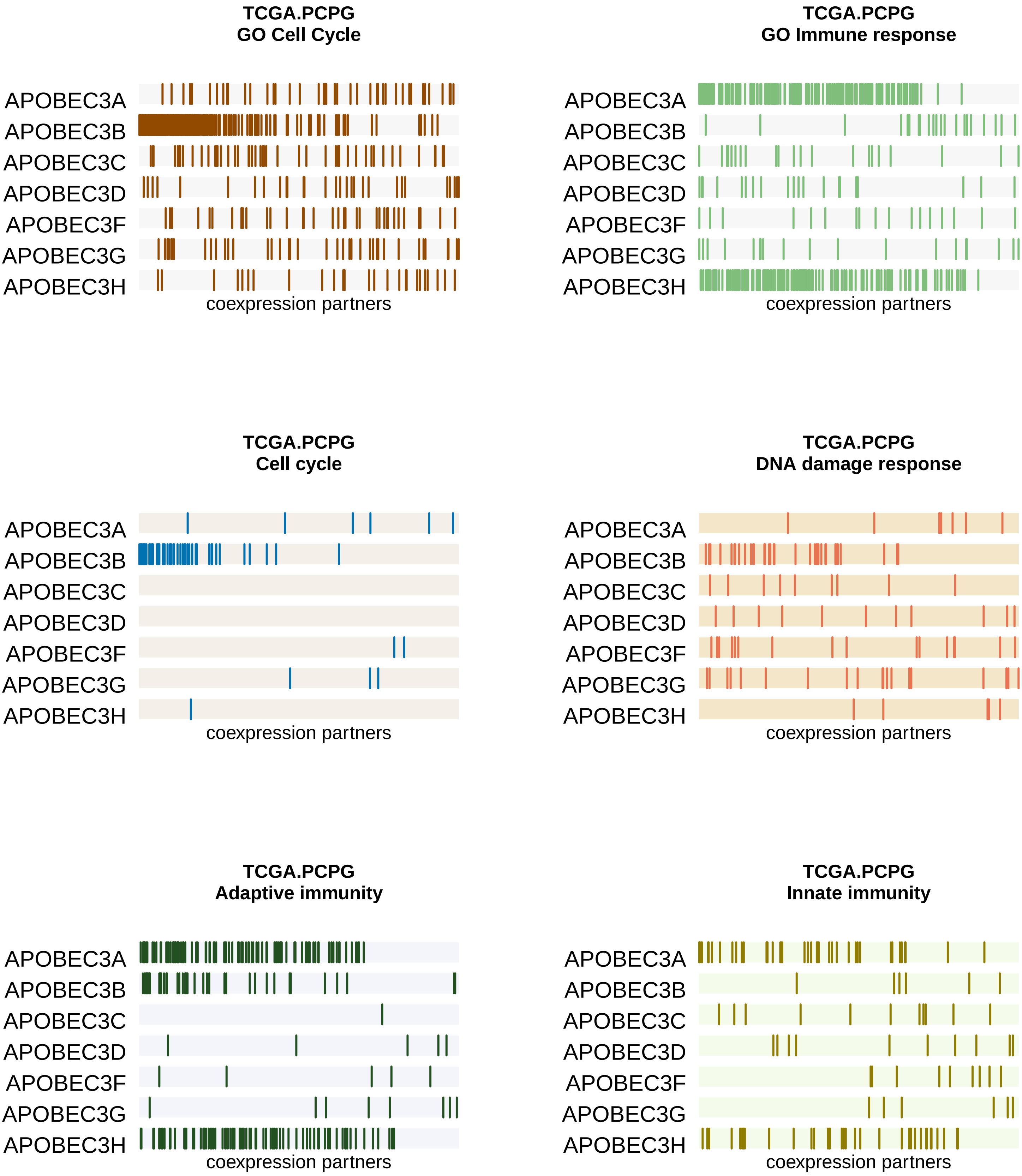
TCGA.PCPG
GO Cell Cycle
APOBEC3A
APOBEC3B
APOBEC3C
APOBEC3D
APOBEC3F
APOBEC3G
APOBEC3H
coexpression partners
TCGA.PCPG
GO Immune response
APOBEC3A
APOBEC3B
APOBEC3C
APOBEC3D
APOBEC3F
APOBEC3G
APOBEC3H
coexpression partners
TCGA.PCPG
Cell cycle
APOBEC3A
APOBEC3B
APOBEC3C
APOBEC3D
APOBEC3F
APOBEC3G
APOBEC3H
coexpression partners
TCGA.PCPG
DNA damage response
APOBEC3A
APOBEC3B
APOBEC3C
APOBEC3D
APOBEC3F
APOBEC3G
APOBEC3H
coexpression partners
TCGA.PCPG
Adaptive immunity
APOBEC3A
APOBEC3B
APOBEC3C
APOBEC3D
APOBEC3F
APOBEC3G
APOBEC3H
coexpression partners
TCGA.PCPG
Innate immunity
APOBEC3A
APOBEC3B
APOBEC3C
APOBEC3D
APOBEC3F
APOBEC3G
APOBEC3H
coexpression partners

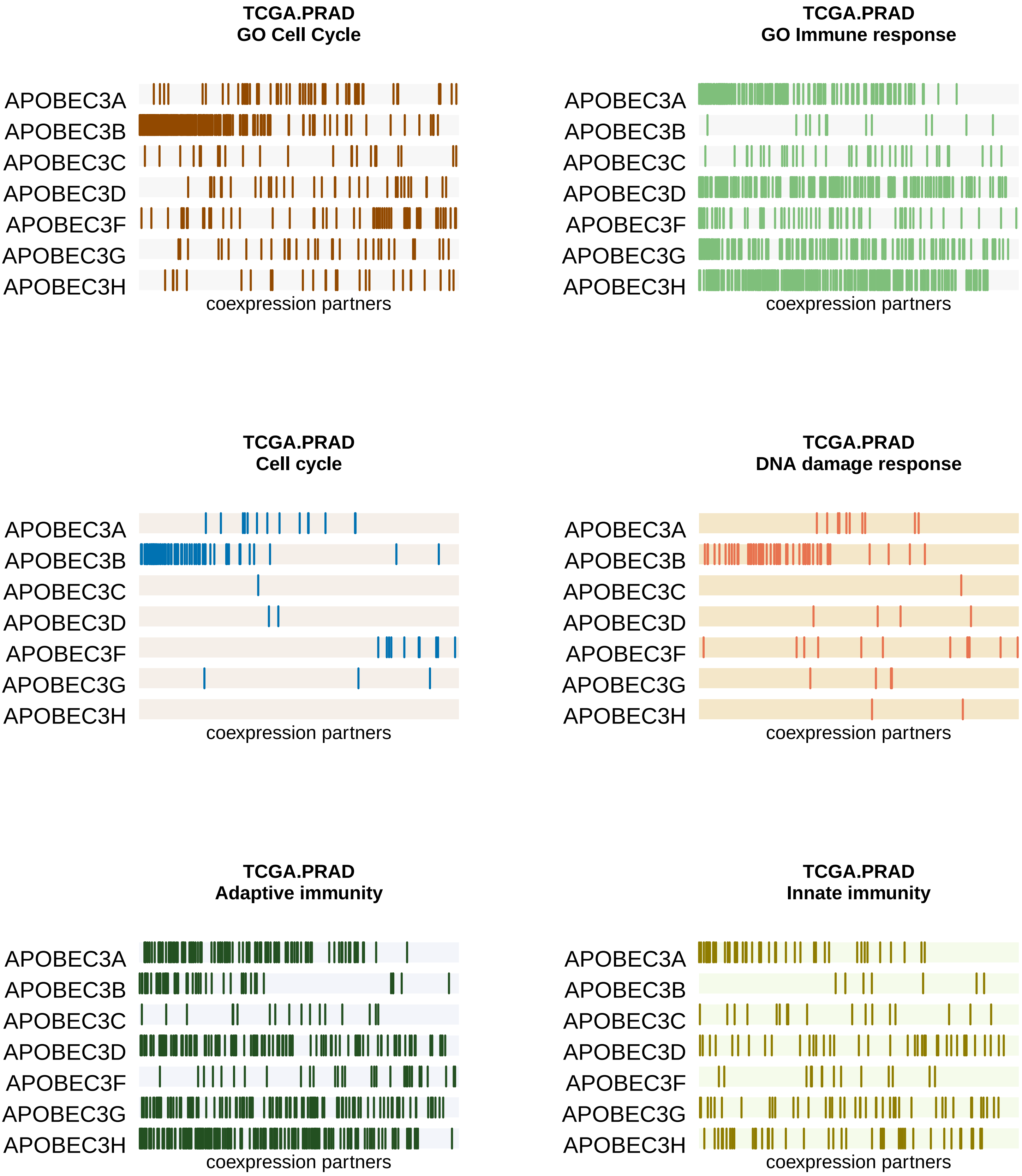
TCGA.PRAD
GO Cell Cycle
APOBEC3A
APOBEC3B
APOBEC3C
APOBEC3D
APOBEC3F
APOBEC3G
APOBEC3H
coexpression partners
TCGA.PRAD
GO Immune response
APOBEC3A
APOBEC3B
APOBEC3C
APOBEC3D
APOBEC3F
APOBEC3G
APOBEC3H
coexpression partners
TCGA.PRAD
Cell cycle
APOBEC3A
APOBEC3B
APOBEC3C
APOBEC3D
APOBEC3F
APOBEC3G
APOBEC3H
coexpression partners
TCGA.PRAD
DNA damage response
APOBEC3A
APOBEC3B
APOBEC3C
APOBEC3D
APOBEC3F
APOBEC3G
APOBEC3H
coexpression partners
TCGA.PRAD
Adaptive immunity
APOBEC3A
APOBEC3B
APOBEC3C
APOBEC3D
APOBEC3F
APOBEC3G
APOBEC3H
coexpression partners
TCGA.PRAD
Innate immunity
APOBEC3A
APOBEC3B
APOBEC3C
APOBEC3D
APOBEC3F
APOBEC3G
APOBEC3H
coexpression partners

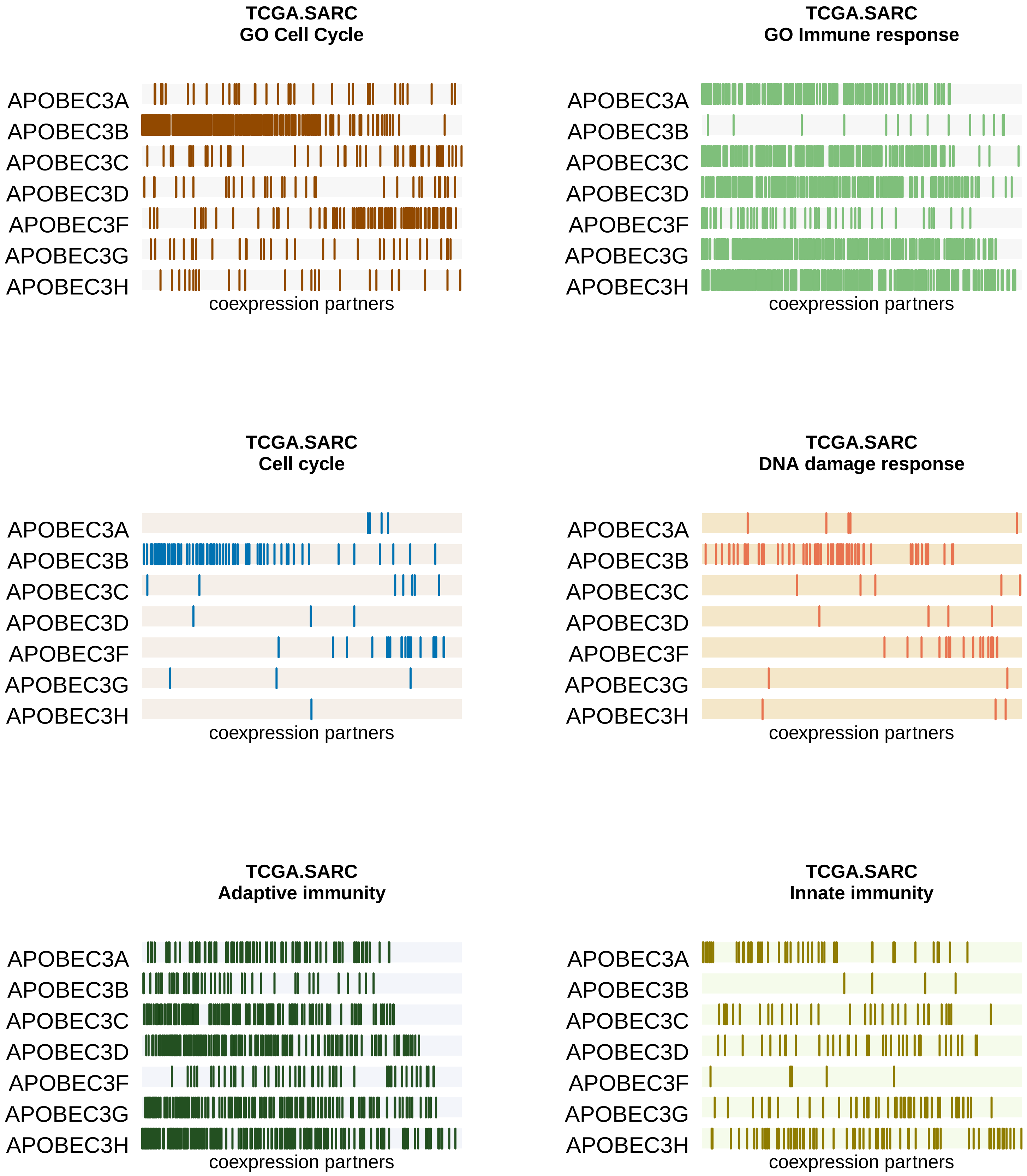
TCGA.SARC
GO Cell Cycle
TCGA.SARC
GO Immune response
TCGA.SARC
Cell cycle
TCGA.SARC
DNA damage response
TCGA.SARC
Adaptive immunity
TCGA.SARC
Innate immunity
APOBEC3A
APOBEC3B
APOBEC3C
APOBEC3D
APOBEC3F
APOBEC3G
APOBEC3H
coexpression partners

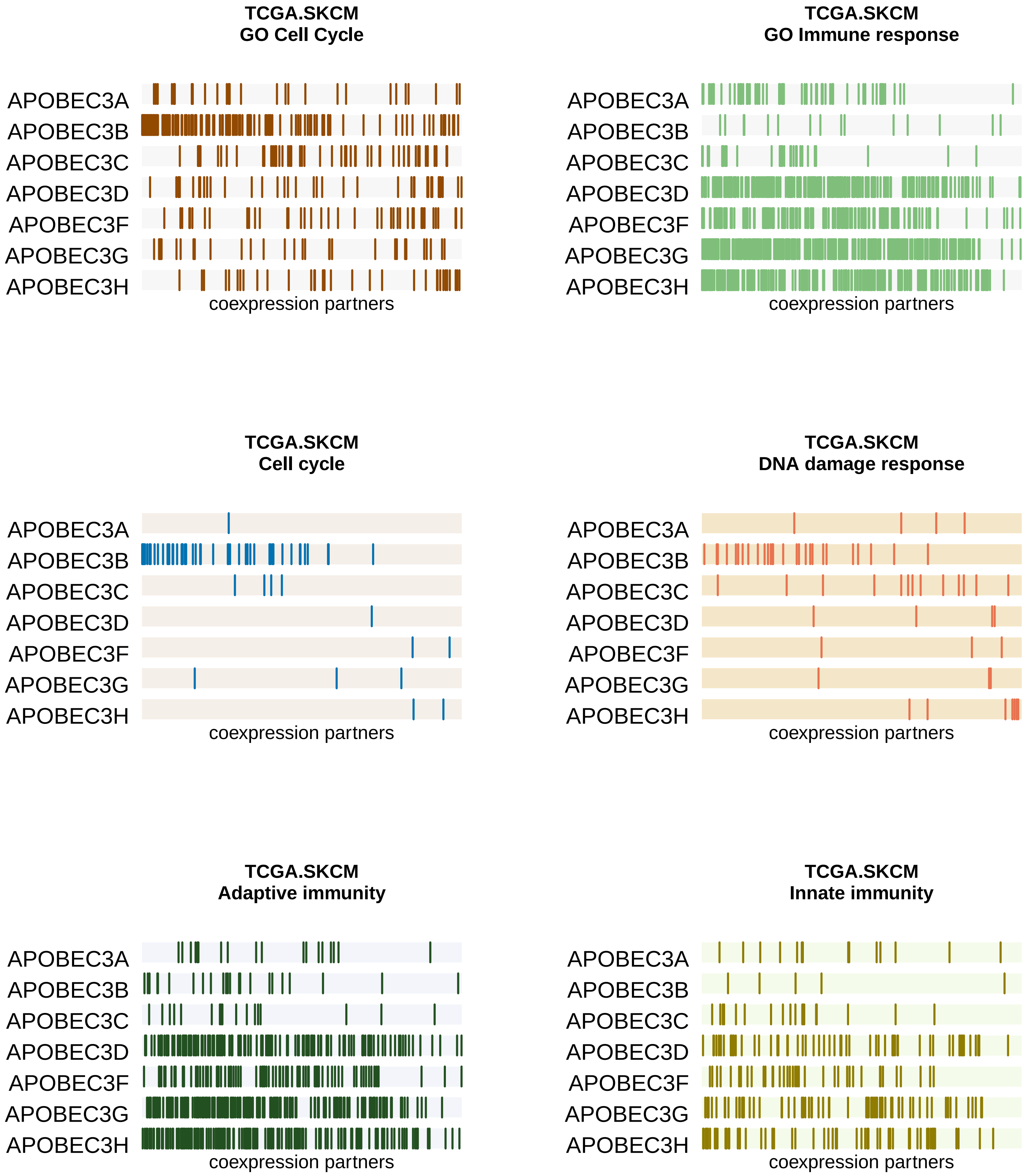
TCGA.SKCM
GO Cell Cycle
TCGA.SKCM
GO Immune response
TCGA.SKCM
Cell cycle
TCGA.SKCM
DNA damage response
TCGA.SKCM
Adaptive immunity
TCGA.SKCM
Innate immunity
APOBEC3A
APOBEC3B
APOBEC3C
APOBEC3D
APOBEC3F
APOBEC3G
APOBEC3H
coexpression partners

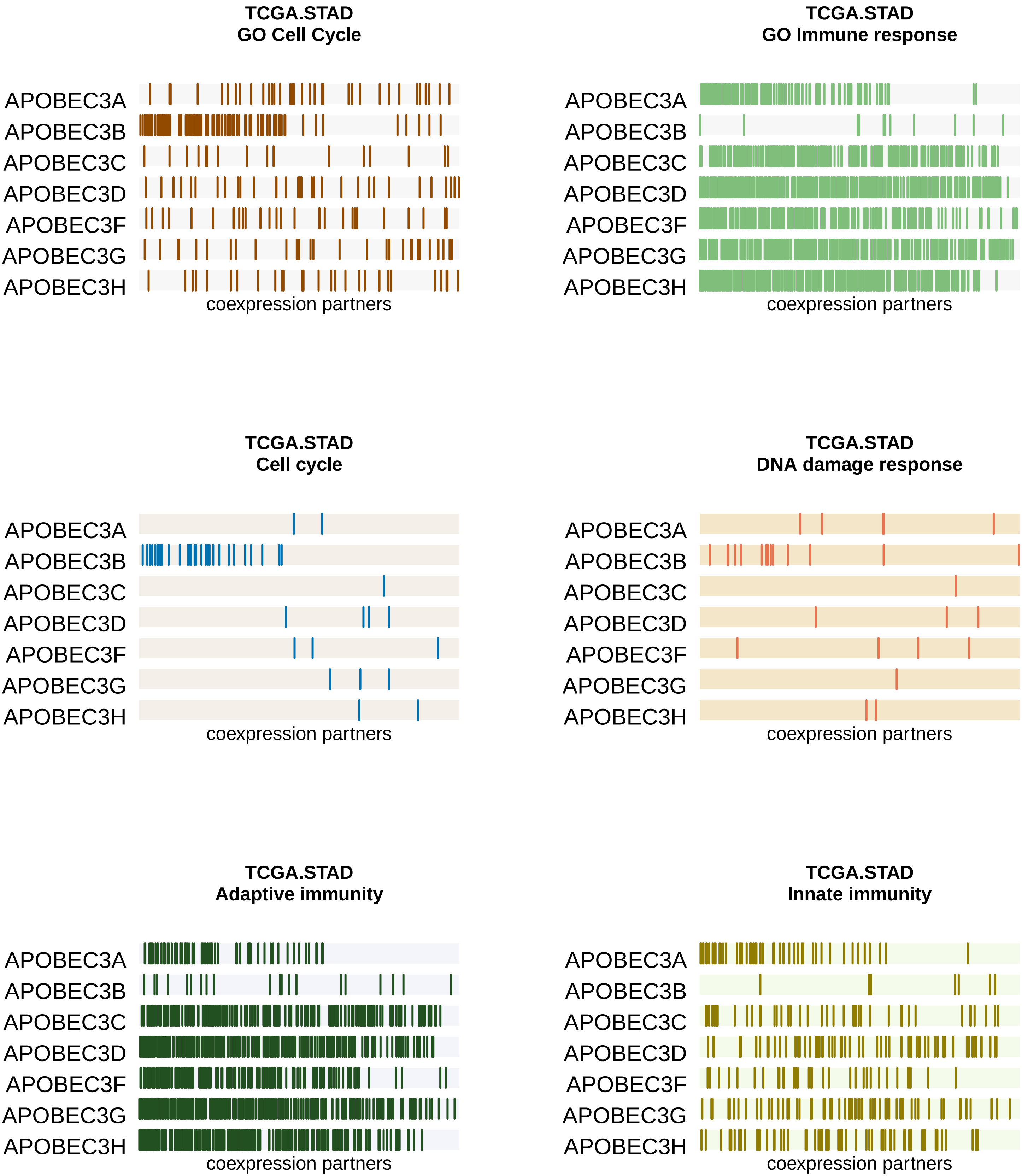
TCGA.STAD
GO Cell Cycle
APOBEC3A
APOBEC3B
APOBEC3C
APOBEC3D
APOBEC3F
APOBEC3G
APOBEC3H
coexpression partners
TCGA.STAD
GO Immune response
APOBEC3A
APOBEC3B
APOBEC3C
APOBEC3D
APOBEC3F
APOBEC3G
APOBEC3H
coexpression partners
TCGA.STAD
Cell cycle
APOBEC3A
APOBEC3B
APOBEC3C
APOBEC3D
APOBEC3F
APOBEC3G
APOBEC3H
coexpression partners
TCGA.STAD
DNA damage response
APOBEC3A
APOBEC3B
APOBEC3C
APOBEC3D
APOBEC3F
APOBEC3G
APOBEC3H
coexpression partners
TCGA.STAD
Adaptive immunity
APOBEC3A
APOBEC3B
APOBEC3C
APOBEC3D
APOBEC3F
APOBEC3G
APOBEC3H
coexpression partners
TCGA.STAD
Innate immunity
APOBEC3A
APOBEC3B
APOBEC3C
APOBEC3D
APOBEC3F
APOBEC3G
APOBEC3H
coexpression partners

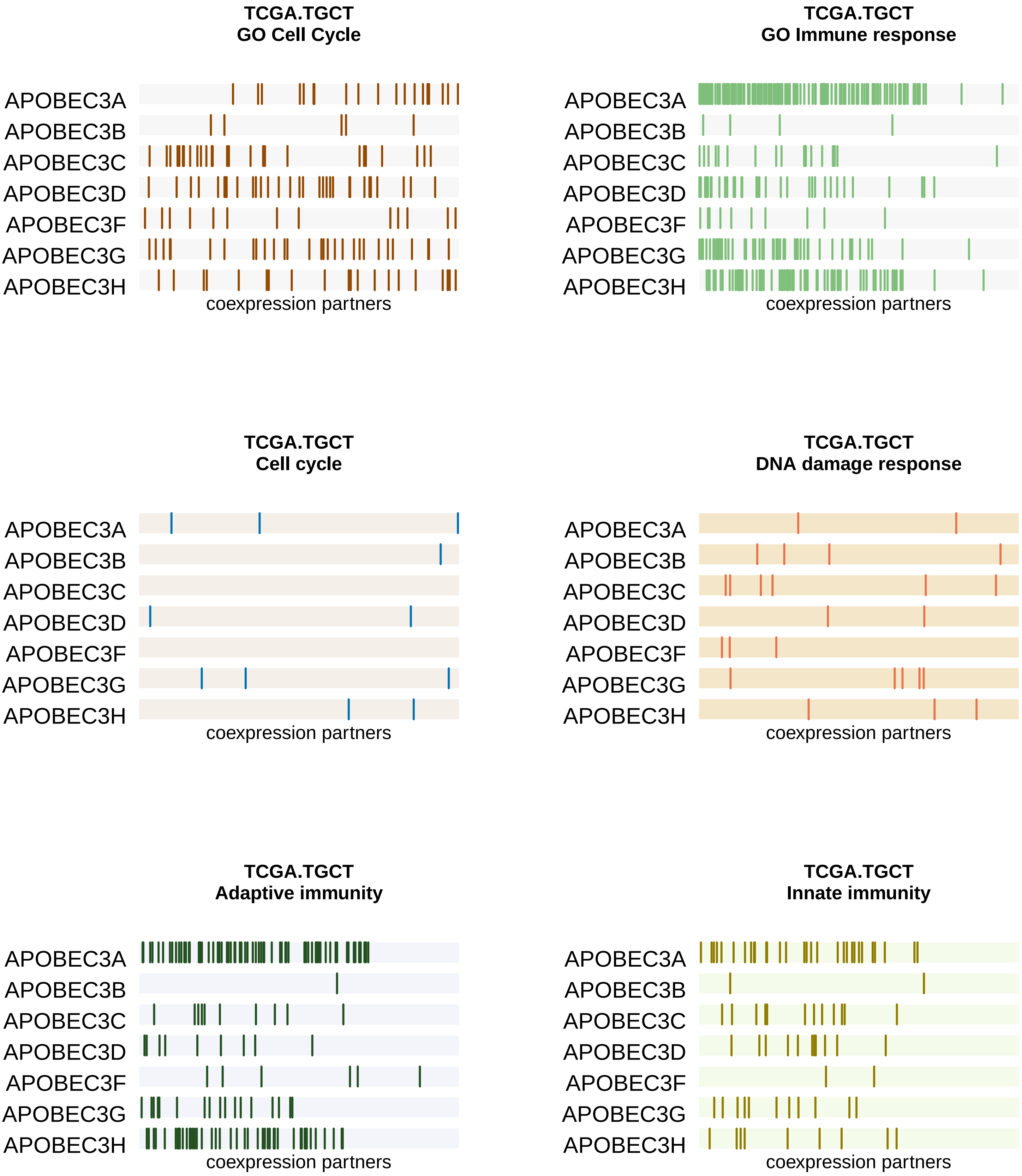
TCGA.TGCT
GO Cell Cycle
TCGA.TGCT
GO Immune response
TCGA.TGCT
Cell cycle
TCGA.TGCT
DNA damage response
TCGA.TGCT
Adaptive immunity
TCGA.TGCT
Innate immunity
APOBEC3A
APOBEC3B
APOBEC3C
APOBEC3D
APOBEC3F
APOBEC3G
APOBEC3H
coexpression partners

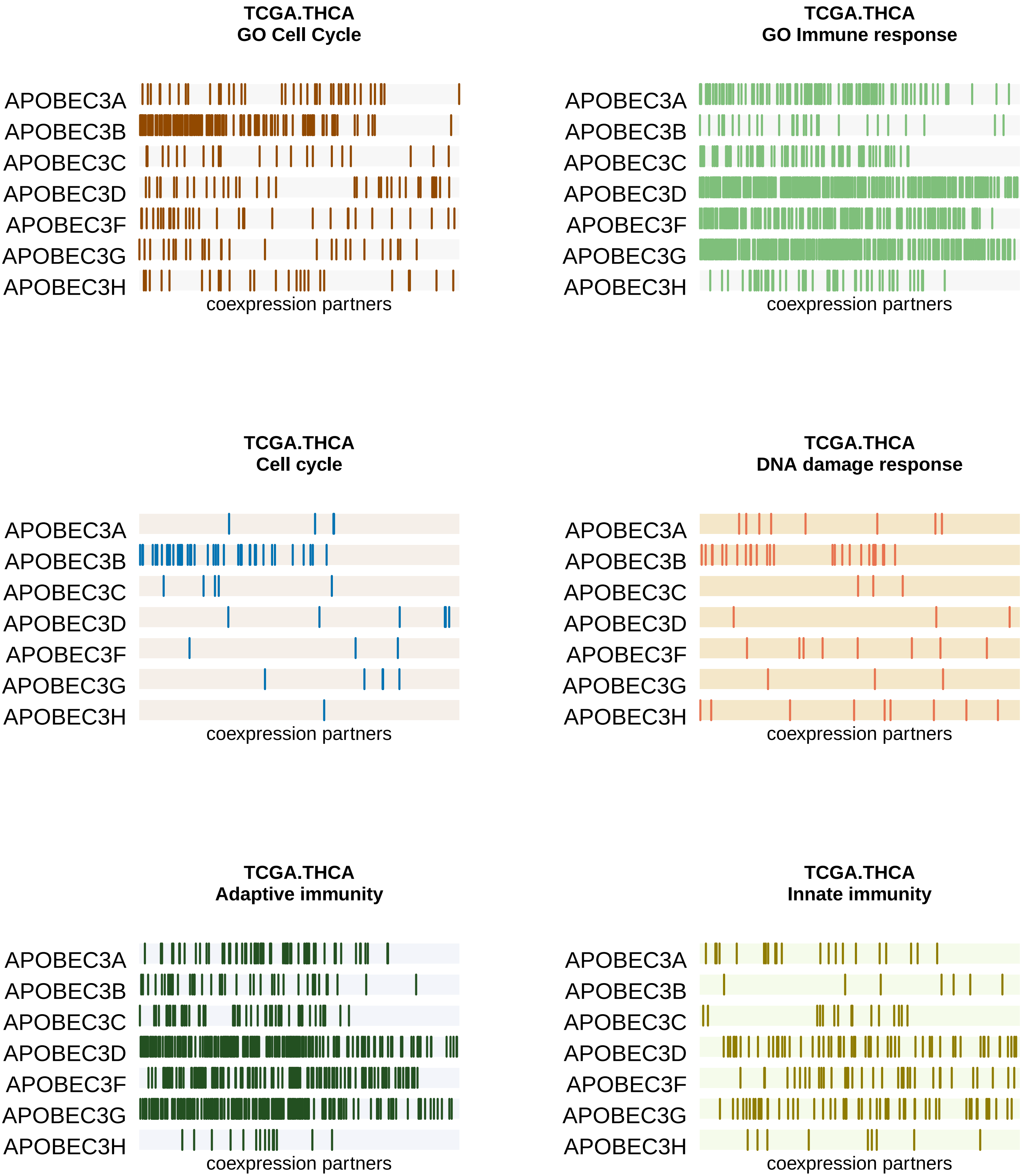
TCGA.THCA
GO Cell Cycle
TCGA.THCA
GO Immune response
TCGA.THCA
Cell cycle
TCGA.THCA
DNA damage response
TCGA.THCA
Adaptive immunity
TCGA.THCA
Innate immunity
APOBEC3A
APOBEC3B
APOBEC3C
APOBEC3D
APOBEC3F
APOBEC3G
APOBEC3H
coexpression partners

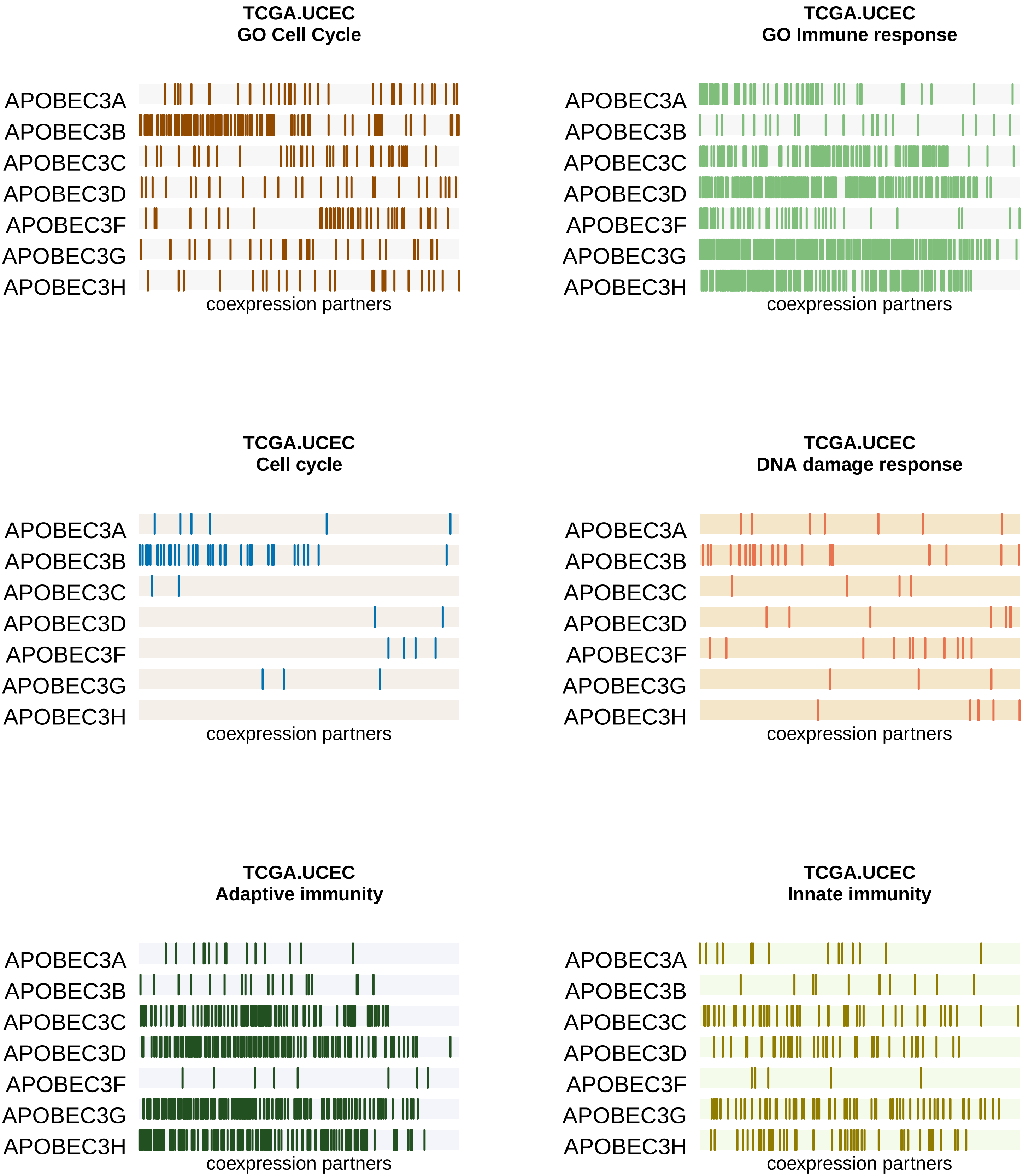
TCGA.UCEC
GO Cell Cycle
TCGA.UCEC
GO Immune response
TCGA.UCEC
Cell cycle
TCGA.UCEC
DNA damage response
TCGA.UCEC
Adaptive immunity
TCGA.UCEC
Innate immunity
APOBEC3A
APOBEC3B
APOBEC3C
APOBEC3D
APOBEC3F
APOBEC3G
APOBEC3H
coexpression partners

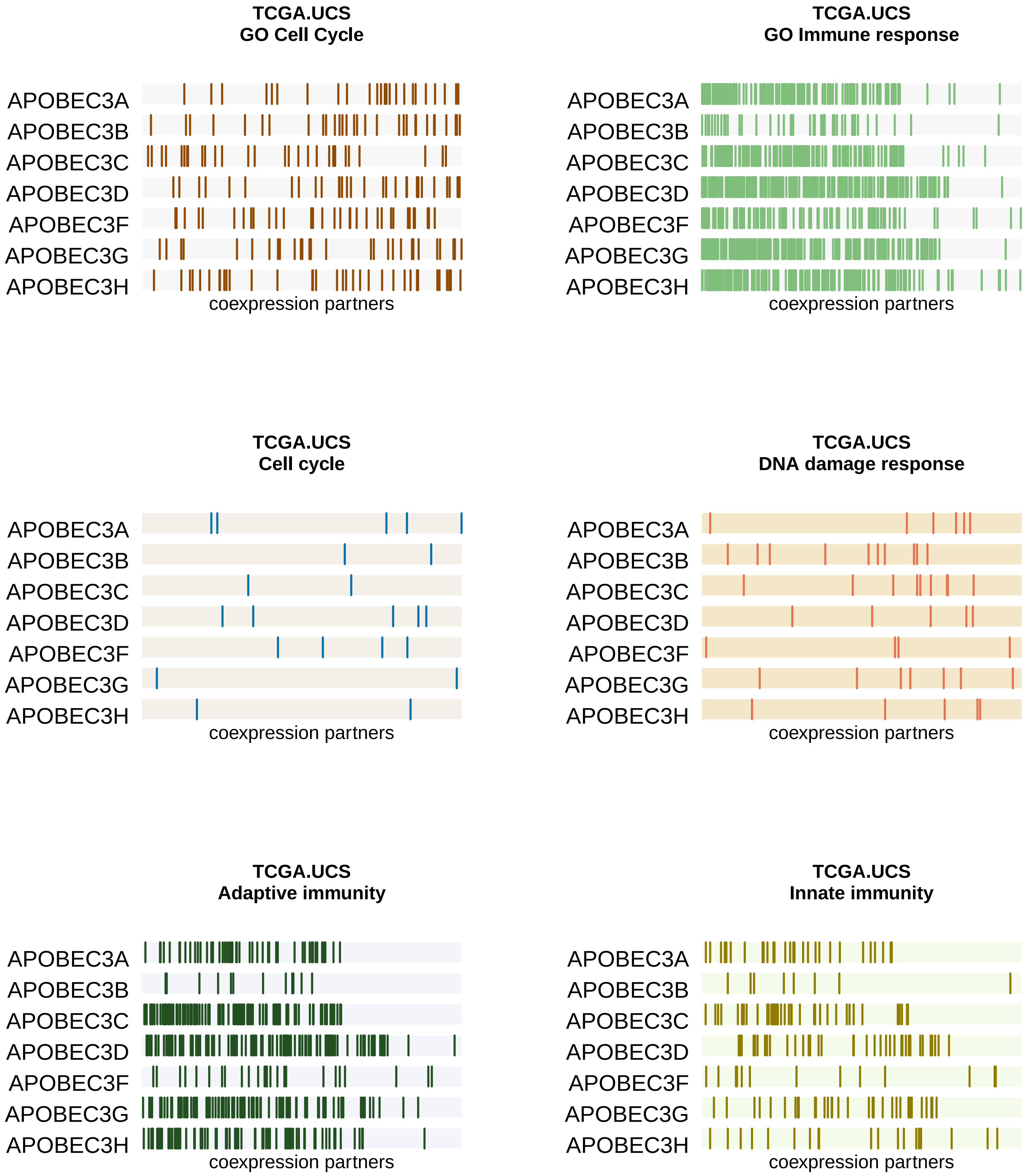
TCGA.UCS
GO Cell Cycle
APOBEC3A
APOBEC3B
APOBEC3C
APOBEC3D
APOBEC3F
APOBEC3G
APOBEC3H
coexpression partners
TCGA.UCS
GO Immune response
APOBEC3A
APOBEC3B
APOBEC3C
APOBEC3D
APOBEC3F
APOBEC3G
APOBEC3H
coexpression partners
TCGA.UCS
Cell cycle
APOBEC3A
APOBEC3B
APOBEC3C
APOBEC3D
APOBEC3F
APOBEC3G
APOBEC3H
coexpression partners
TCGA.UCS
DNA damage response
APOBEC3A
APOBEC3B
APOBEC3C
APOBEC3D
APOBEC3F
APOBEC3G
APOBEC3H
coexpression partners
TCGA.UCS
Adaptive immunity
APOBEC3A
APOBEC3B
APOBEC3C
APOBEC3D
APOBEC3F
APOBEC3G
APOBEC3H
coexpression partners
TCGA.UCS
Innate immunity
APOBEC3A
APOBEC3B
APOBEC3C
APOBEC3D
APOBEC3F
APOBEC3G
APOBEC3H
coexpression partners
