# Supplementary material for: Pan-cancer transcriptomic analysis dissects immune and proliferative functions of APOBEC3 cytidine deaminases
Source: Nucleic Acids Res. 2019 Jan 9;47(3):1178–94. doi: 10.1093/nar/gky1316 (PMC6379723; doi:10.1093/nar/gky1316)
Supplement: Supplementary Data [file gky1316_supplemental_files.zip › FigureS15_gsea_pca.pdf]

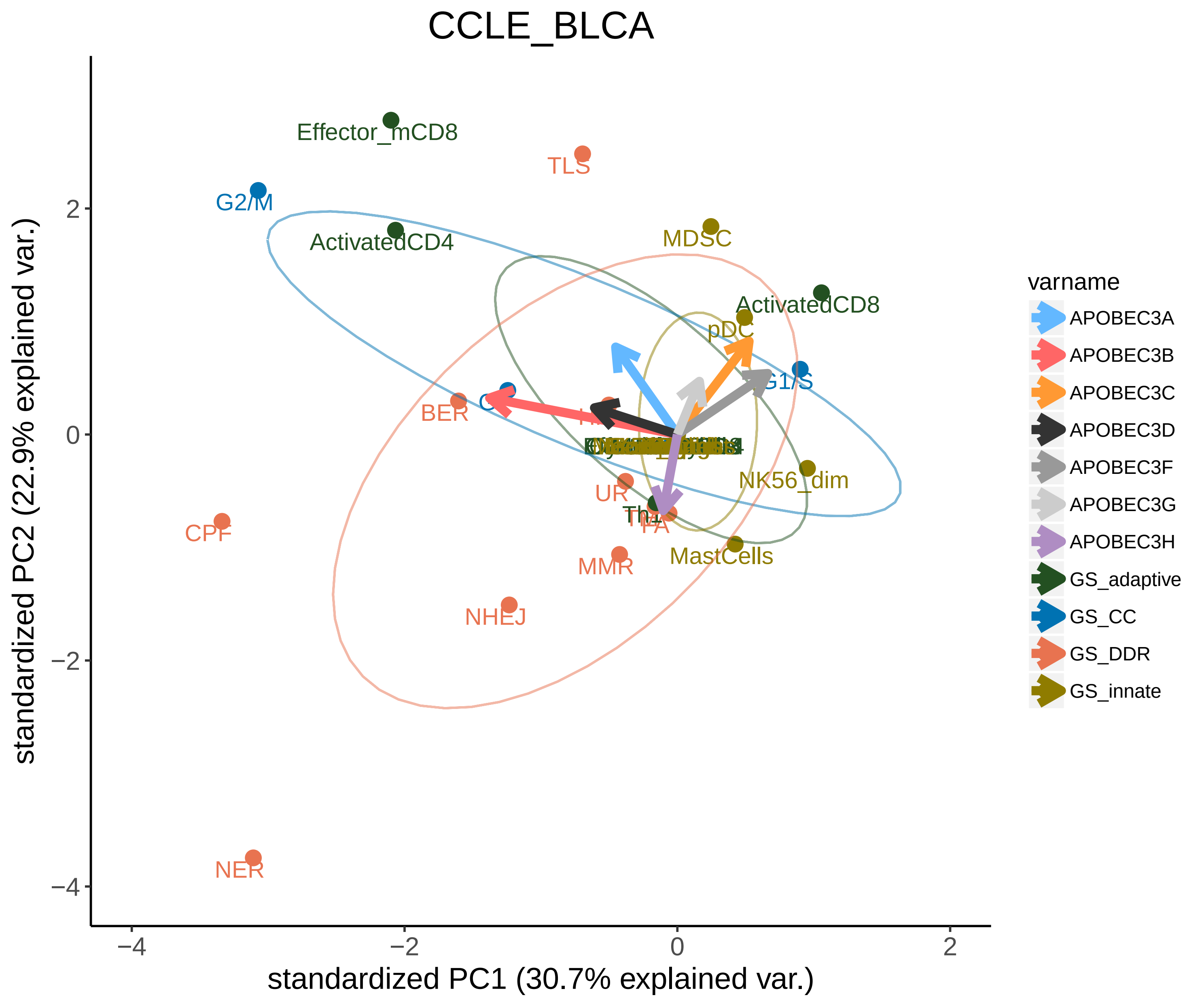

CCLE_BLCA
Effector_mCD8
TLS
G2/M
ActivatedCD4
MDSC
ActivatedCD8
pDC
G1/S
BER
NK56_dim
UR
CPF
MMR
MastCells
NHEJ
NER
standardized PC1 (30.7% explained var.)
standardized PC2 (22.9% explained var.)
−4
−2
0
2
varname
APOBEC3A
APOBEC3B
APOBEC3C
APOBEC3D
APOBEC3F
APOBEC3G
APOBEC3H
GS_adaptive
GS_CC
GS_DDR
GS_innate

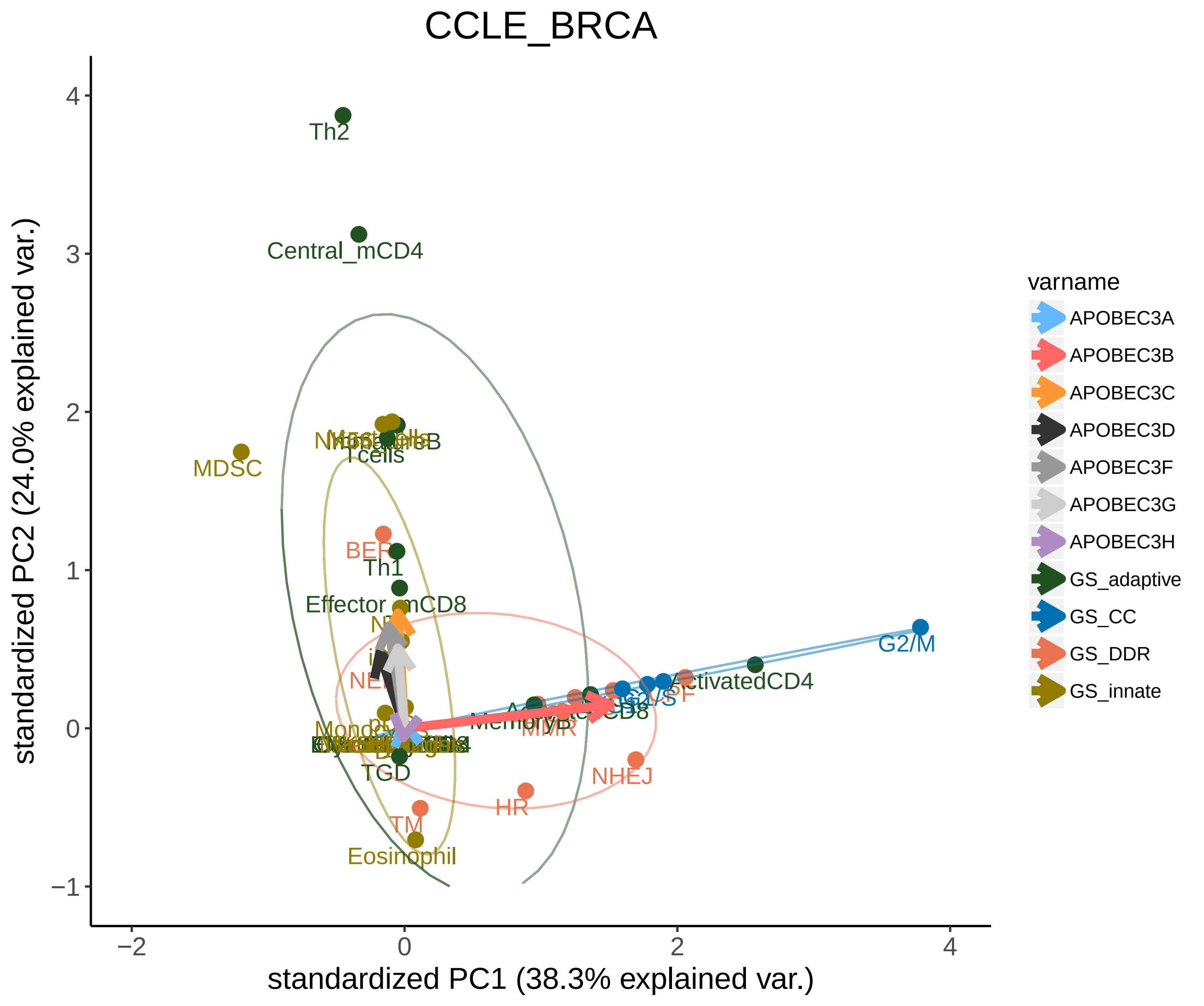

CCLE_BRCA
standardized PC1 (38.3% explained var.)
standardized PC2 (24.0% explained var.)
varname
APOBEC3A
APOBEC3B
APOBEC3C
APOBEC3D
APOBEC3F
APOBEC3G
APOBEC3H
GS_adaptive
GS_CC
GS_DDR
GS_innate
Th2
Central_mCD4
MDSC
Tcells
BER
Th1
Effector_mCD8
G2/M
NHEJ
HR
TM
TGD
Eosinophil
MMR

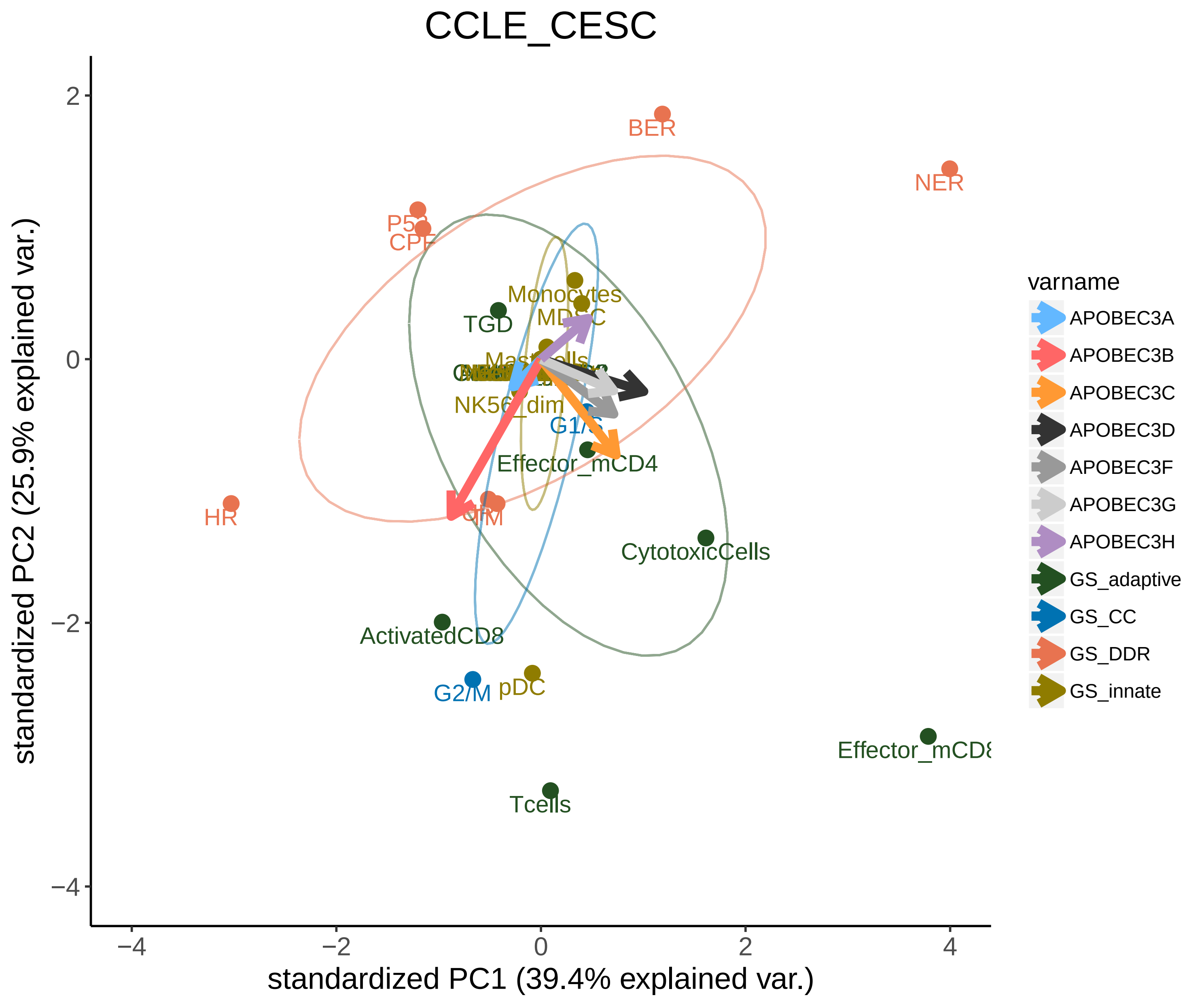

CCLE_CESC
standardized PC1 (39.4% explained var.)
standardized PC2 (25.9% explained var.)
varname
APOBEC3A
APOBEC3B
APOBEC3C
APOBEC3D
APOBEC3F
APOBEC3G
APOBEC3H
GS_adaptive
GS_CC
GS_DDR
GS_innate
BER
NER
HR
CPF
Monocytes
TGD
NK56_dim
Effector_mCD4
CytotoxicCells
ActivatedCD8
G2/M
pDC
Effector_mCD8
Tcells
2
0
−2
−4
4

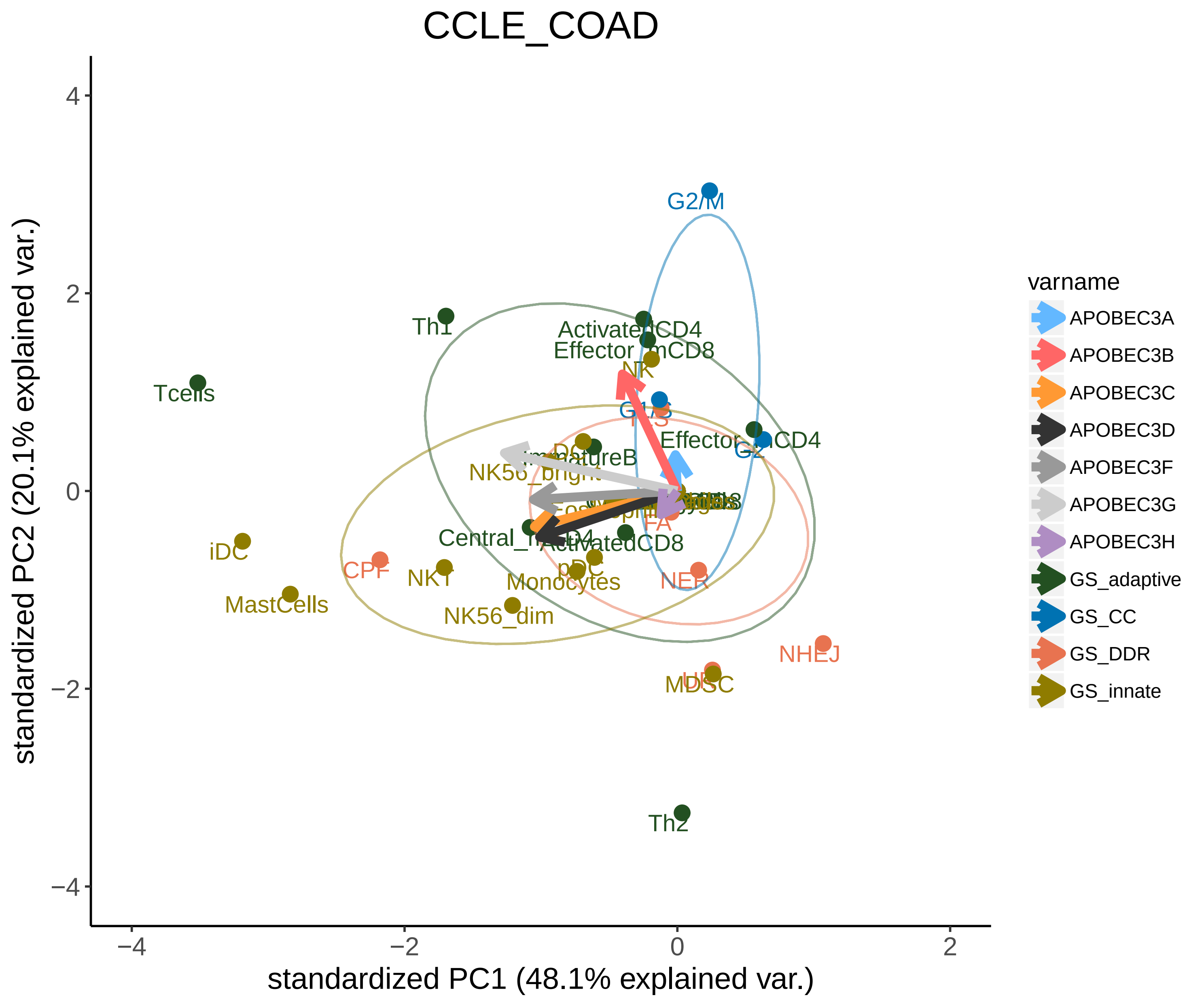
CCLE_COAD
standardized PC2 (20.1% explained var.)
standardized PC1 (48.1% explained var.)
4
2
0
−2
−4
−4
−2
0
2
G2/M
Th1
Tcells
ActivatedCD4
Effector_mCD8
NK
G1/S
Effector_mCD4
G2
NK56_bright
iDC
CPF
NK1
MastCells
Monocytes
NK56_dim
NER
ActivatedCD8
pDC
NHEJ
MDSC
Th2
FA
varname
APOBEC3A
APOBEC3B
APOBEC3C
APOBEC3D
APOBEC3F
APOBEC3G
APOBEC3H
GS_adaptive
GS_CC
GS_DDR
GS_innate

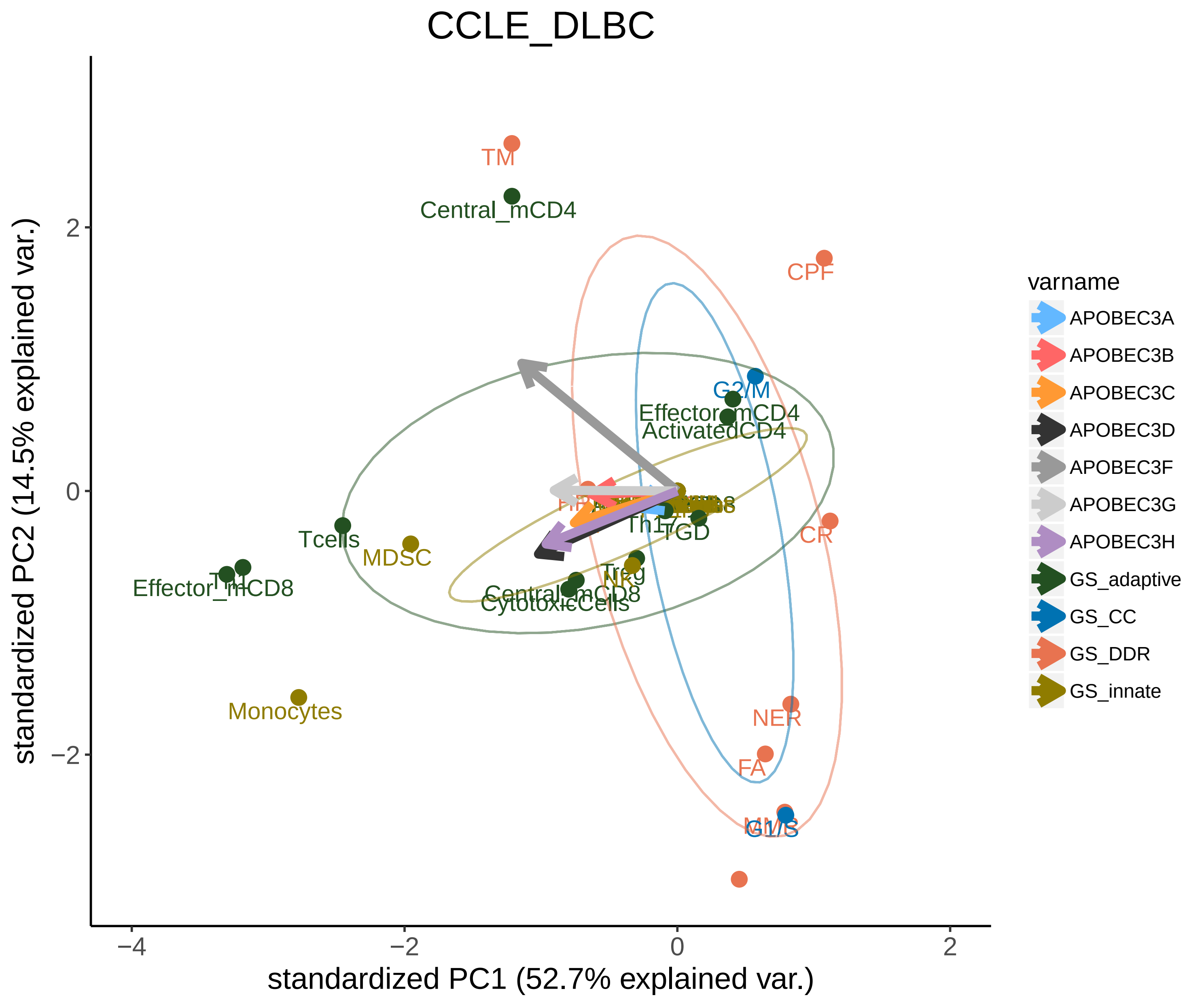
CCLE_DLBC
TM
Central_mCD4
CPF
G2/M
Effector_mCD4
ActivatedCD4
Tcells
MDSC
Effector_mCD8
Th1
TGD
CR
Treg
NK
Central_mCD8
CytotoxicCells
Monocytes
NER
FA
G1/S
standardized PC2 (14.5% explained var.)
standardized PC1 (52.7% explained var.)
2
0
−2
−4
varname
APOBEC3A
APOBEC3B
APOBEC3C
APOBEC3D
APOBEC3F
APOBEC3G
APOBEC3H
GS_adaptive
GS_CC
GS_DDR
GS_innate

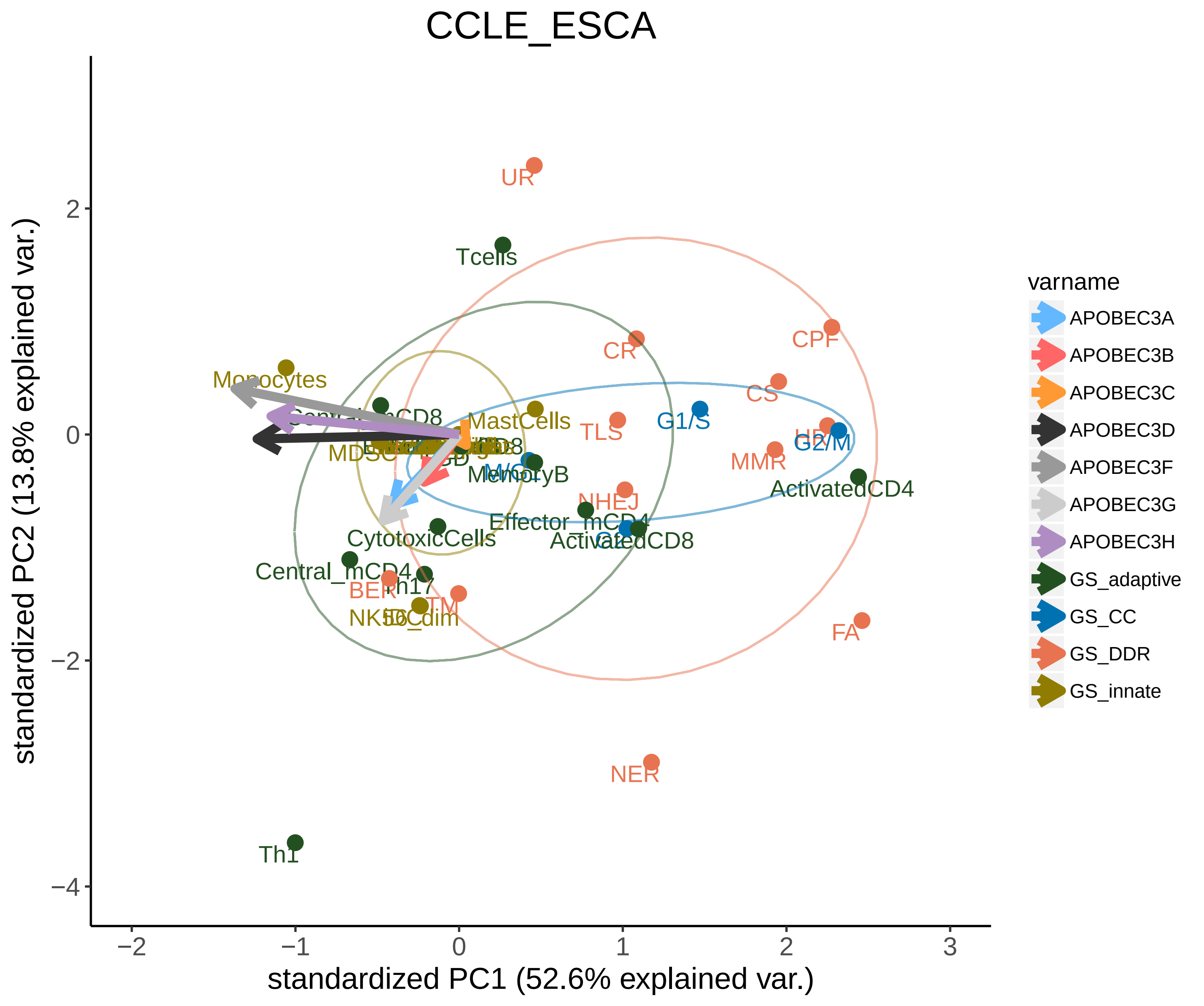
CCLE_ESCA
standardized PC1 (52.6% explained var.)
standardized PC2 (13.8% explained var.)
varname
APOBEC3A
APOBEC3B
APOBEC3C
APOBEC3D
APOBEC3F
APOBEC3G
APOBEC3H
GS_adaptive
GS_CC
GS_DDR
GS_innate
UR
Tcells
CR
CPF
Monocytes
CS
MastCells
TLS
G1/S
MMR
G2/M
MemoryB
ActivatedCD4
NHEJ
Effector_mCD4
ActivatedCD8
CytotoxicCells
Central_mCD4
Th17
BER
NK56Cdim
FA
NER
Th1

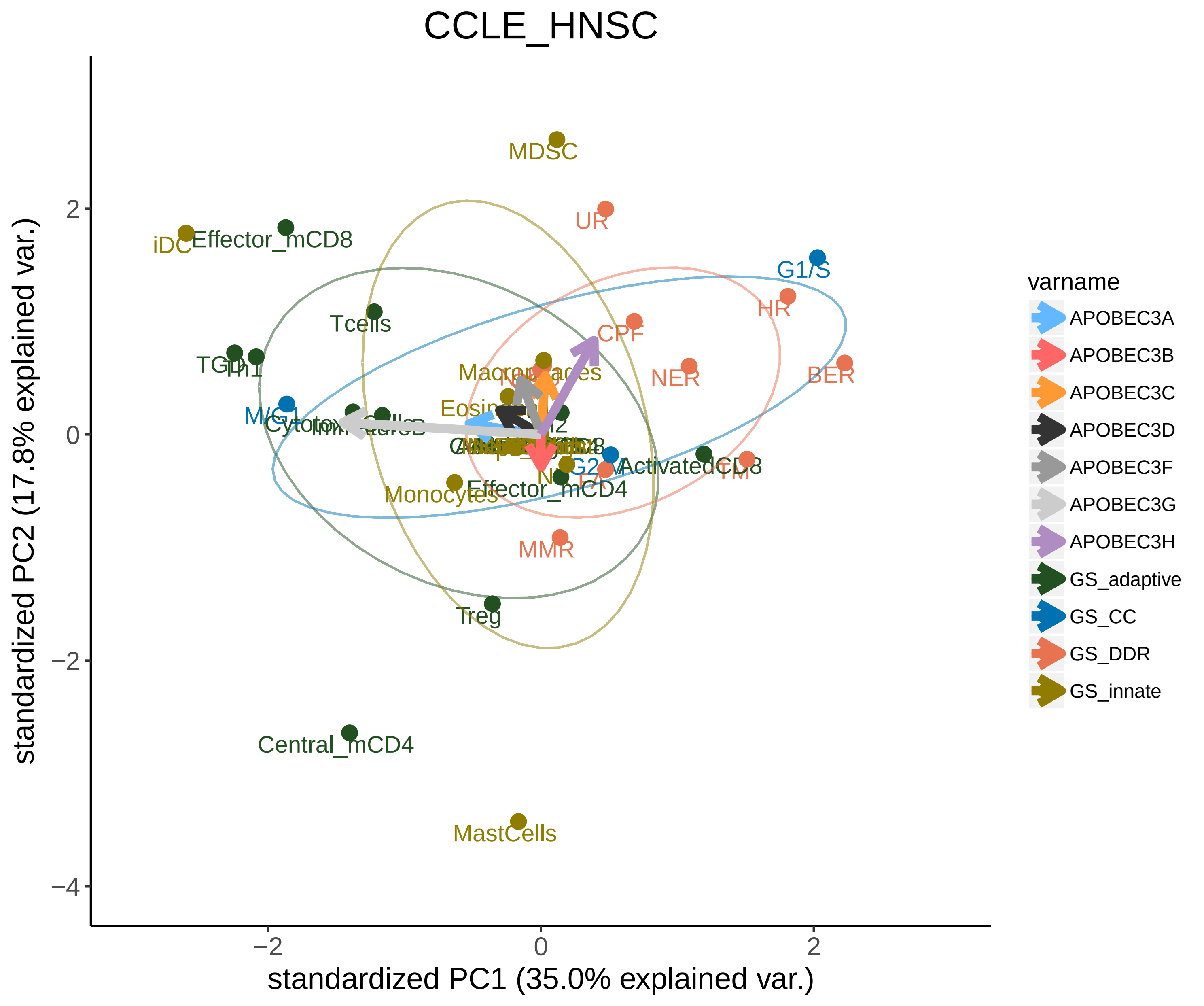
CCLE_HNSC
standardized PC2 (17.8% explained var.)
standardized PC1 (35.0% explained var.)
2
0
−2
−4
−2
0
2
MDSC
UR
iDC
Effector_mCD8
G1/S
HR
Tcells
CPF
TGD
Th1
NER
BER
Macrophages
M/G1
Eosin
ActivatedCD8
TM
Monocytes
Effector_mCD4
MMR
Treg
Central_mCD4
MastCells
varname
APOBEC3A
APOBEC3B
APOBEC3C
APOBEC3D
APOBEC3F
APOBEC3G
APOBEC3H
GS_adaptive
GS_CC
GS_DDR
GS_innate

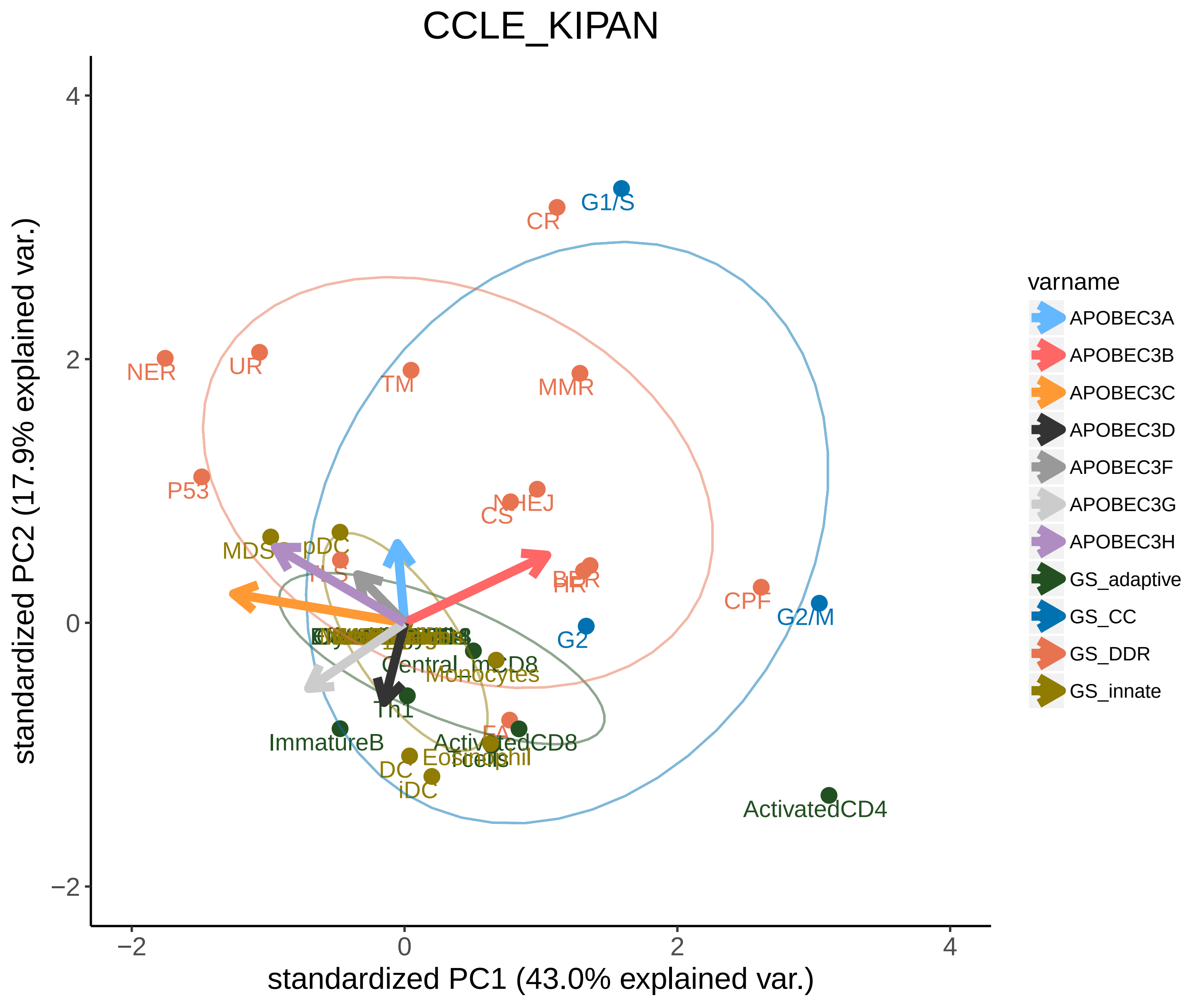

CCLE_KIPAN
standardized PC1 (43.0% explained var.)
standardized PC2 (17.9% explained var.)
−2
0
2
4
−2
0
2
4
varname
APOBEC3A
APOBEC3B
APOBEC3C
APOBEC3D
APOBEC3F
APOBEC3G
APOBEC3H
GS_adaptive
GS_CC
GS_DDR
GS_innate
G1/S
CR
NER
UR
TM
MMR
P53
NHEJ
CS
MDSC
pDC
BER
HR
CPF
G2/M
G2
Central_mCD8
Monocytes
Th1
ImmatureB
ActivatedCD8
Eosinophil
DC
iDC
ActivatedCD4

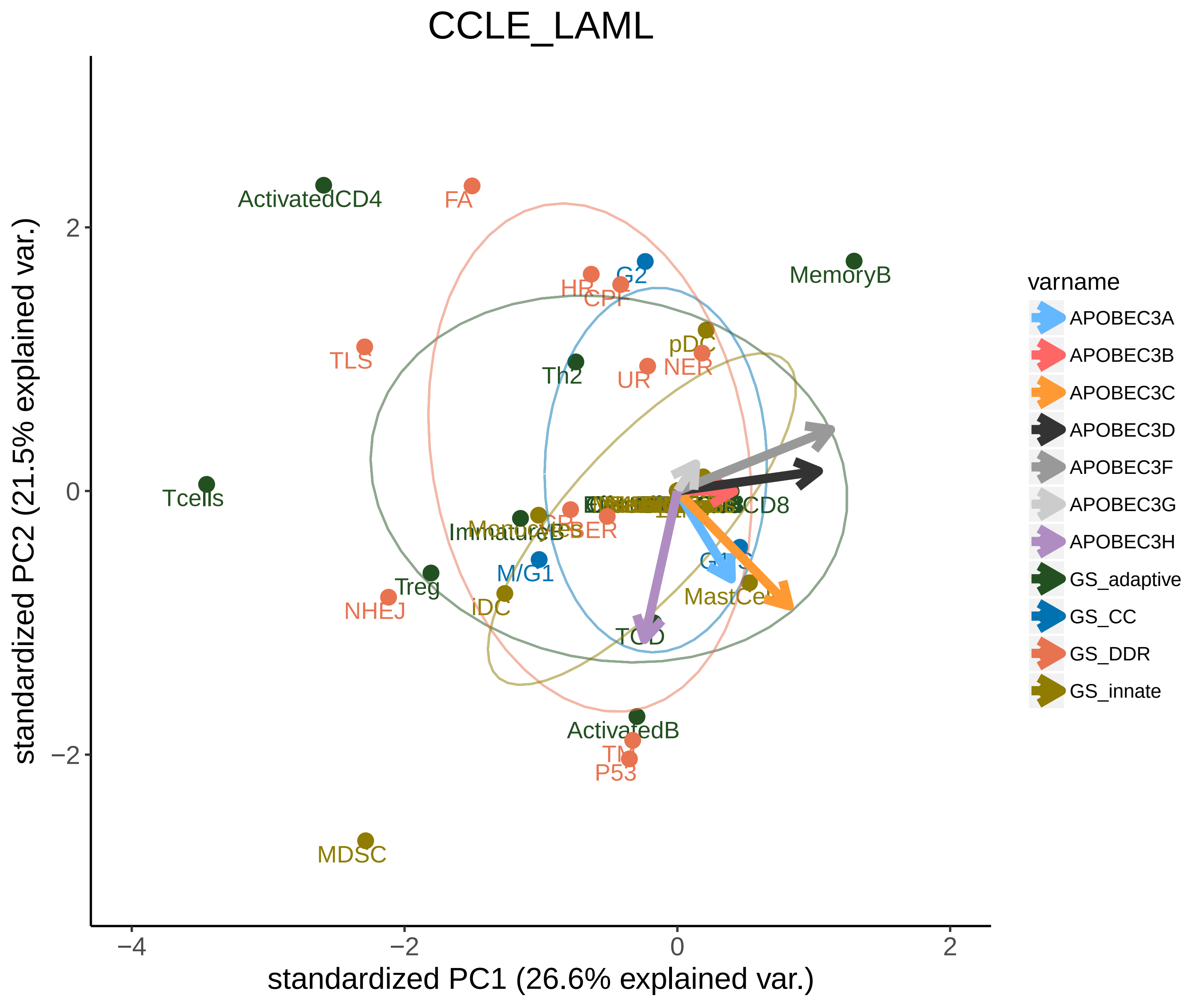
CCLE_LAML
standardized PC1 (26.6% explained var.)
standardized PC2 (21.5% explained var.)
−4
−2
0
2
−2
0
2
ActivatedCD4
FA
MemoryB
G2
HR
CPF
TLS
pDC
Th2
UR
NER
Tcells
CD8
ImmatureB
Monocytes
BER
M/G1
Treg
NHEJ
iDC
MastCell
TCD
ActivatedB
P53
MDSC
varname
APOBEC3A
APOBEC3B
APOBEC3C
APOBEC3D
APOBEC3F
APOBEC3G
APOBEC3H
GS_adaptive
GS_CC
GS_DDR
GS_innate

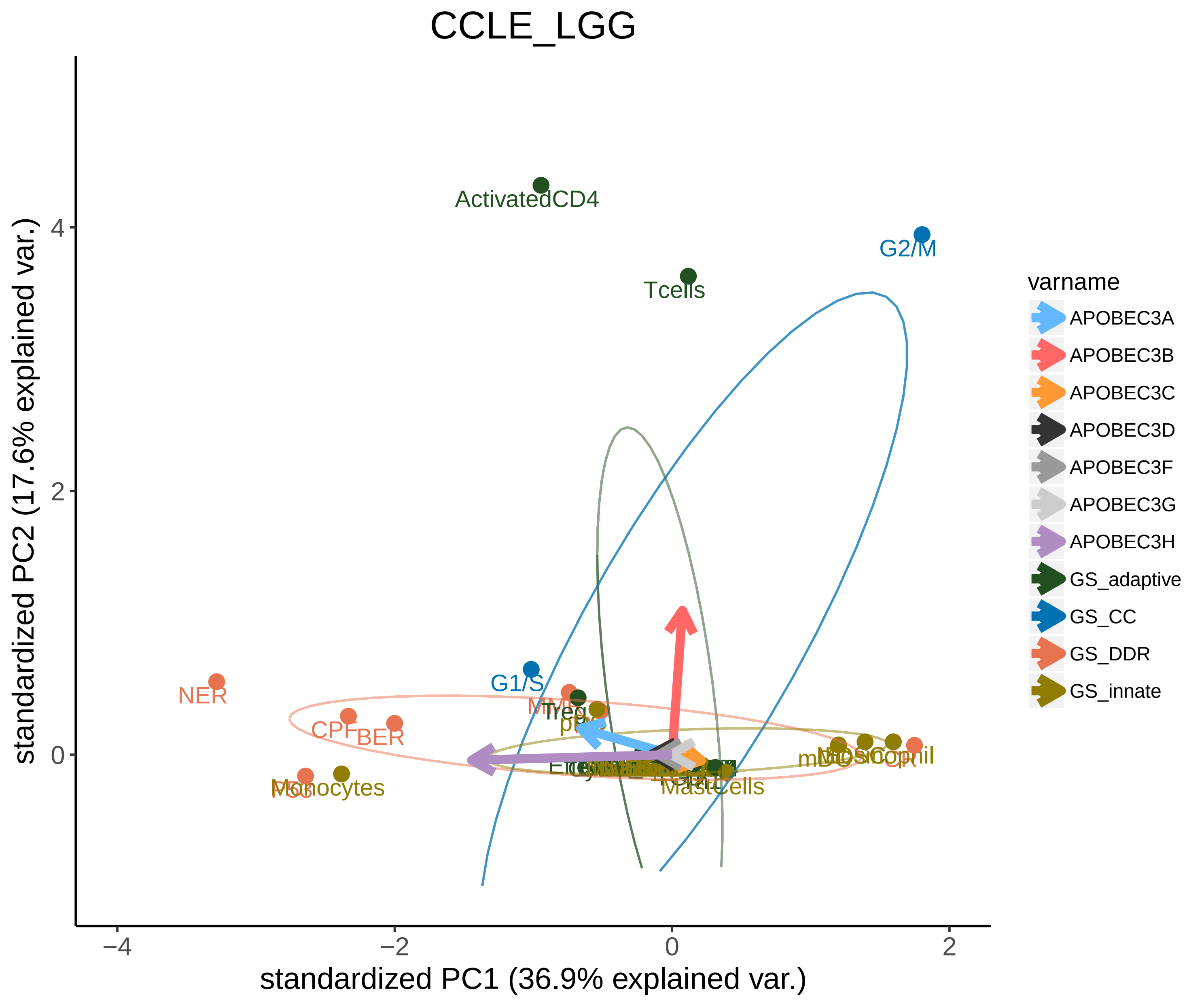
CCLE_LGG
ActivatedCD4
G2/M
Tcells
G1/S
NER
CPF
BER
Treg
Monocytes
MastCells
standardized PC2 (17.6% explained var.)
standardized PC1 (36.9% explained var.)
4
2
0
−4
−2
0
2
varname
APOBEC3A
APOBEC3B
APOBEC3C
APOBEC3D
APOBEC3F
APOBEC3G
APOBEC3H
GS_adaptive
GS_CC
GS_DDR
GS_innate

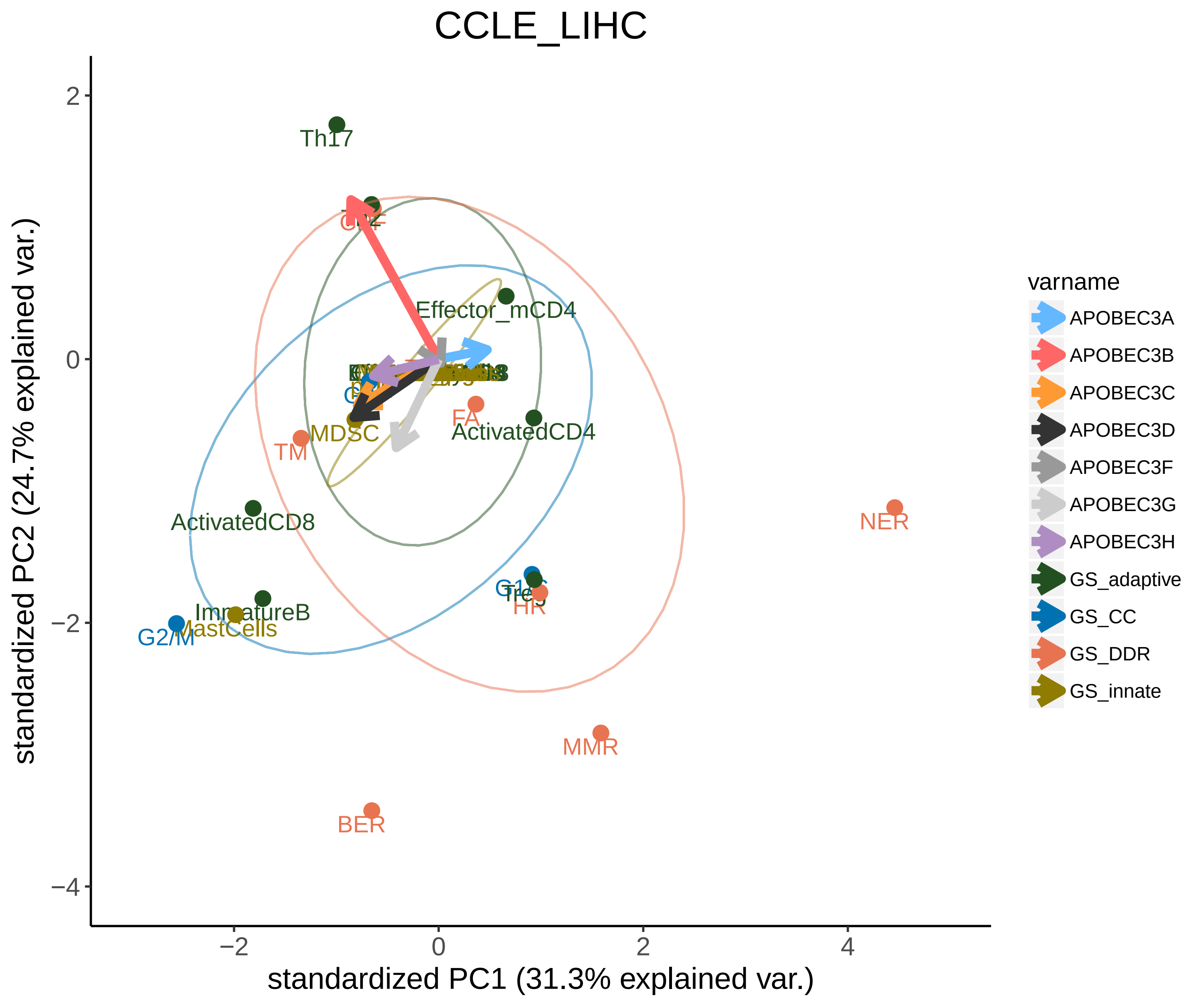

CCLE_LIHC
standardized PC1 (31.3% explained var.)
standardized PC2 (24.7% explained var.)
−2
0
2
4
−4
Th17
Effector_mCD4
MDSC
TM
FA
ActivatedCD4
ActivatedCD8
NER
ImmatureB
MastCells
G2/M
HR
MMR
BER
varname
APOBEC3A
APOBEC3B
APOBEC3C
APOBEC3D
APOBEC3F
APOBEC3G
APOBEC3H
GS_adaptive
GS_CC
GS_DDR
GS_innate

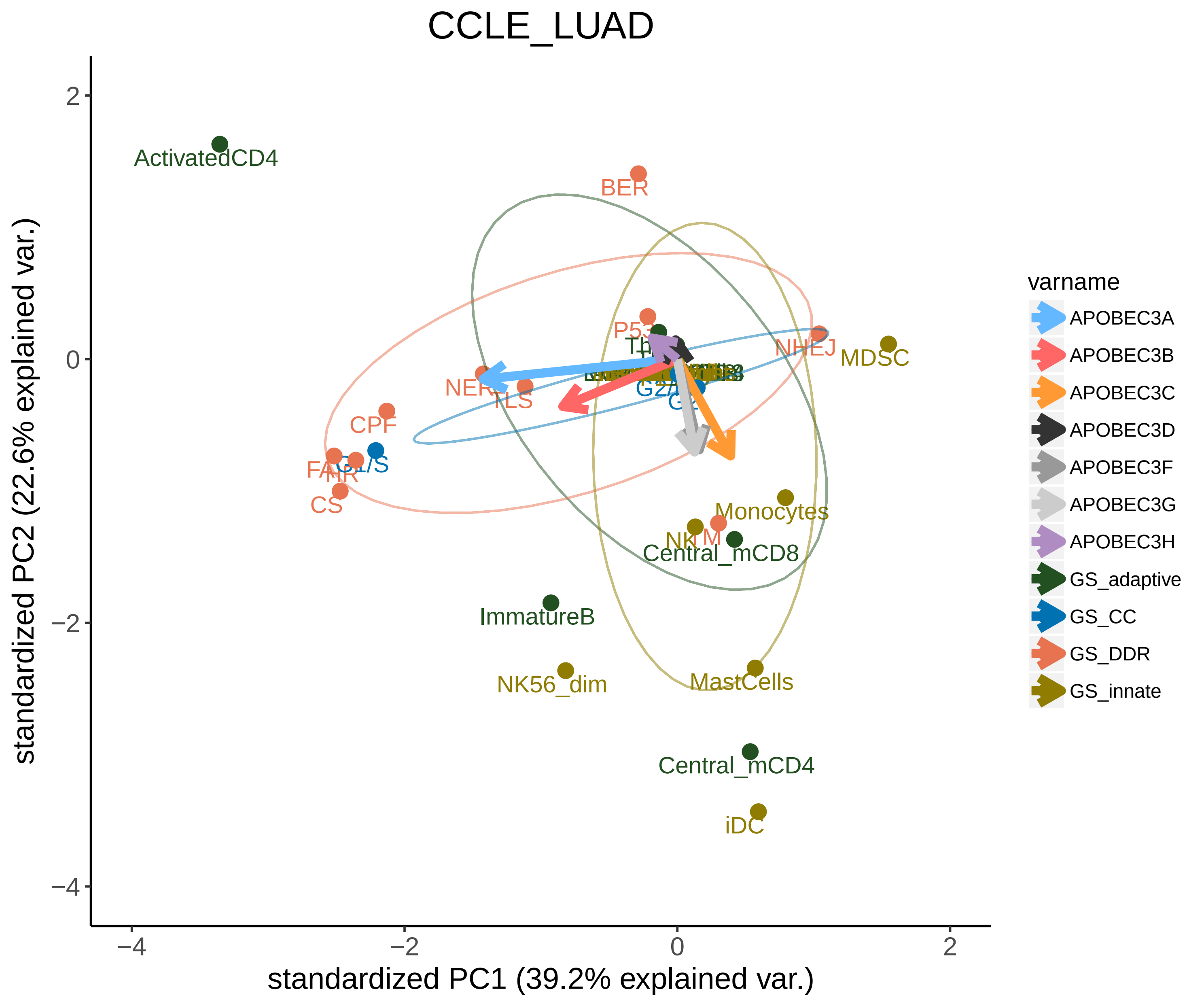

CCLE_LUAD
standardized PC1 (39.2% explained var.)
standardized PC2 (22.6% explained var.)
ActivatedCD4
BER
P53
NHEJ
MDSC
NER
TLS
CPF
G1/S
FA
HR
CS
Monocytes
Central_mCD8
ImmatureB
NK56_dim
MastCells
Central_mCD4
iDC
varname
APOBEC3A
APOBEC3B
APOBEC3C
APOBEC3D
APOBEC3F
APOBEC3G
APOBEC3H
GS_adaptive
GS_CC
GS_DDR
GS_innate
−4
−2
0
2

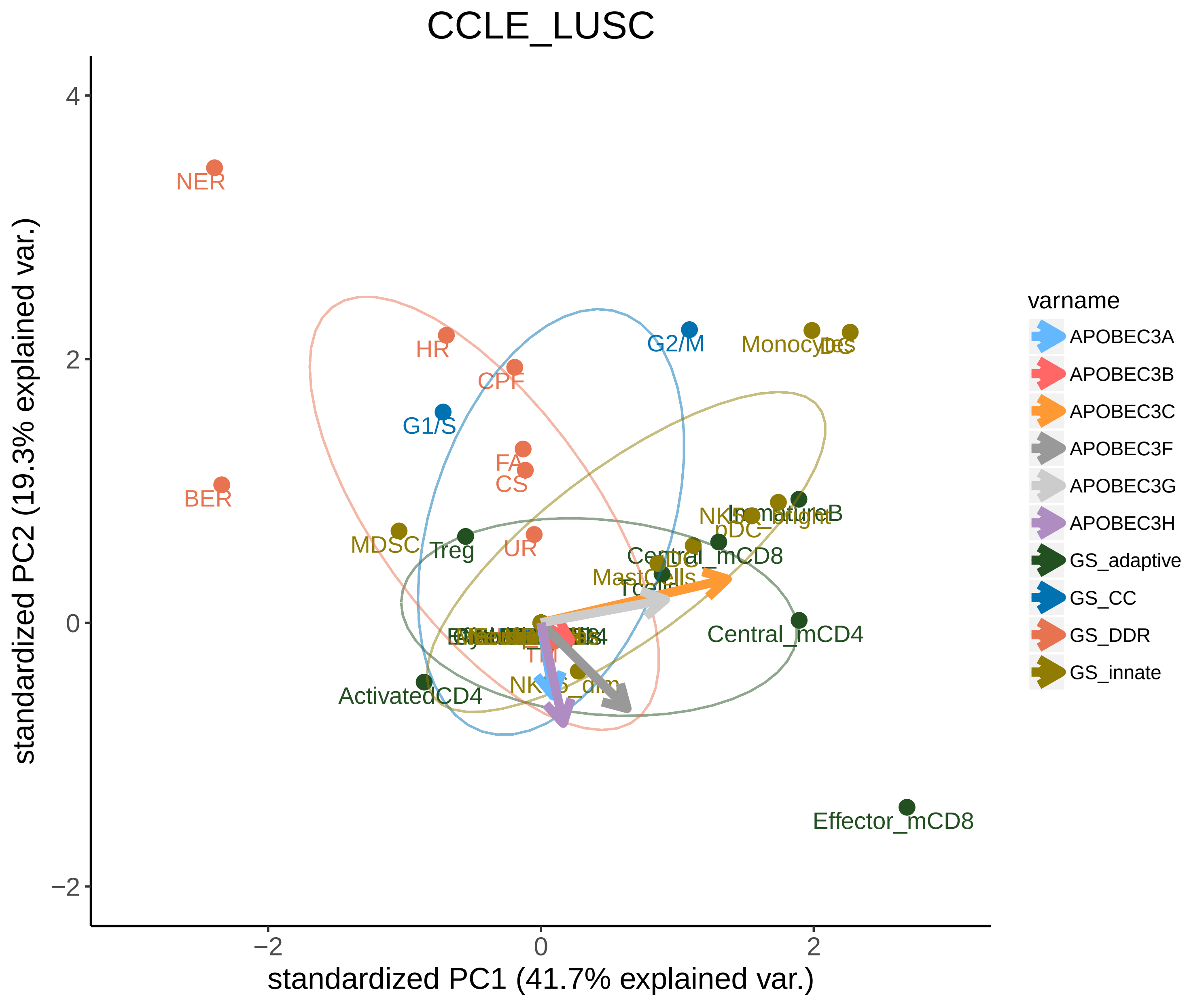

CCLE_LUSC
standardized PC2 (19.3% explained var.)
standardized PC1 (41.7% explained var.)
4
2
0
−2
−2
0
2
NER
HR
CPF
G1/S
FA
CS
BER
UR
G2/M
Monocytes
DCs
MDSC
Treg
Central_mCD8
MastCells
Central_mCD4
ActivatedCD4
Effector_mCD8
ImmatureB
varname
APOBEC3A
APOBEC3B
APOBEC3C
APOBEC3F
APOBEC3G
APOBEC3H
GS_adaptive
GS_CC
GS_DDR
GS_innate

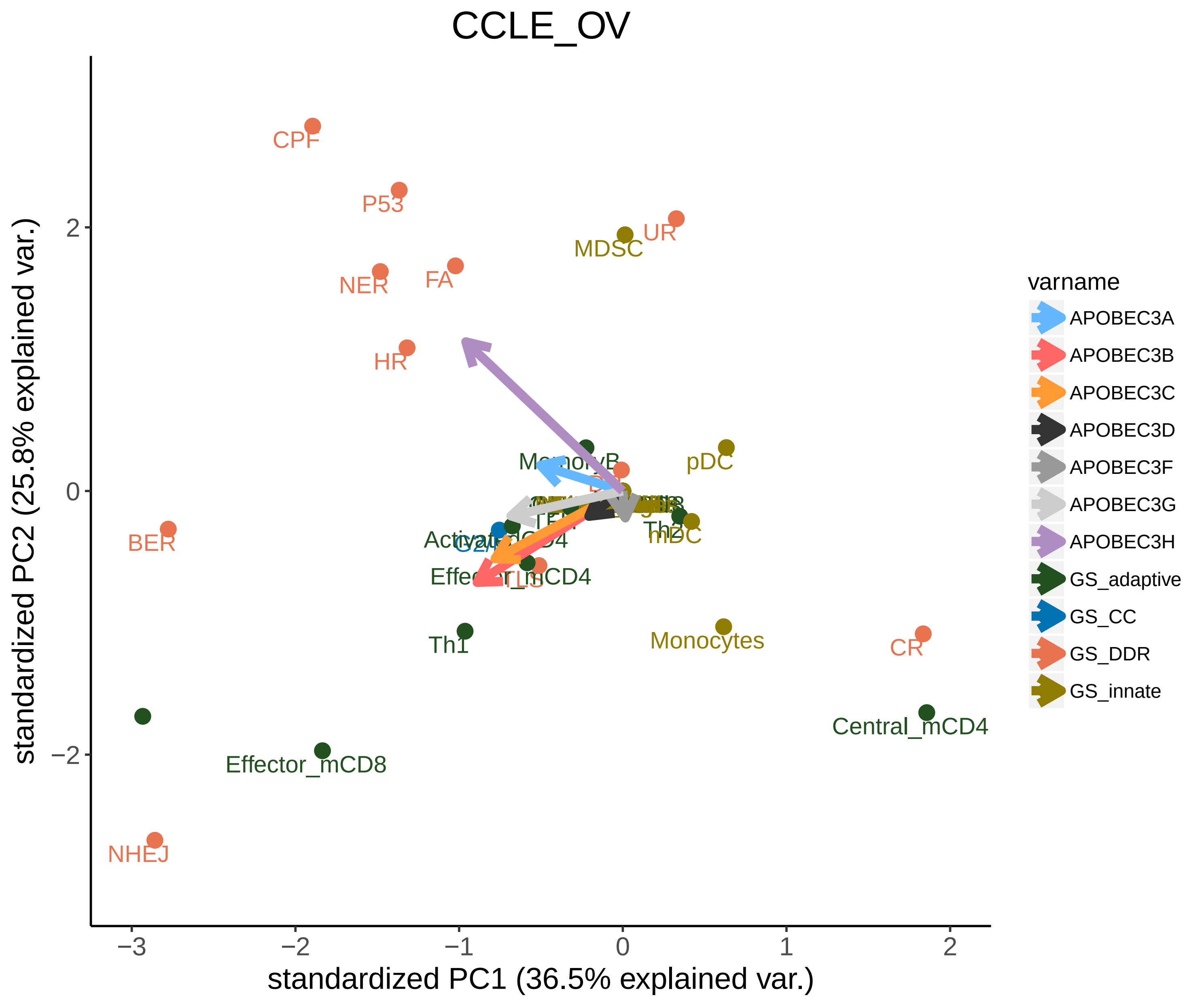

CCLE_OV
standardized PC1 (36.5% explained var.)
standardized PC2 (25.8% explained var.)
−3
−2
−1
0
1
2
−2
0
2
CPF
P53
UR
MDSC
NER
FA
HR
pDC
BER
Effector_mCD4
Th1
Monocytes
CR
Central_mCD4
Effector_mCD8
NHEJ
mDC
varname
APOBEC3A
APOBEC3B
APOBEC3C
APOBEC3D
APOBEC3F
APOBEC3G
APOBEC3H
GS_adaptive
GS_CC
GS_DDR
GS_innate

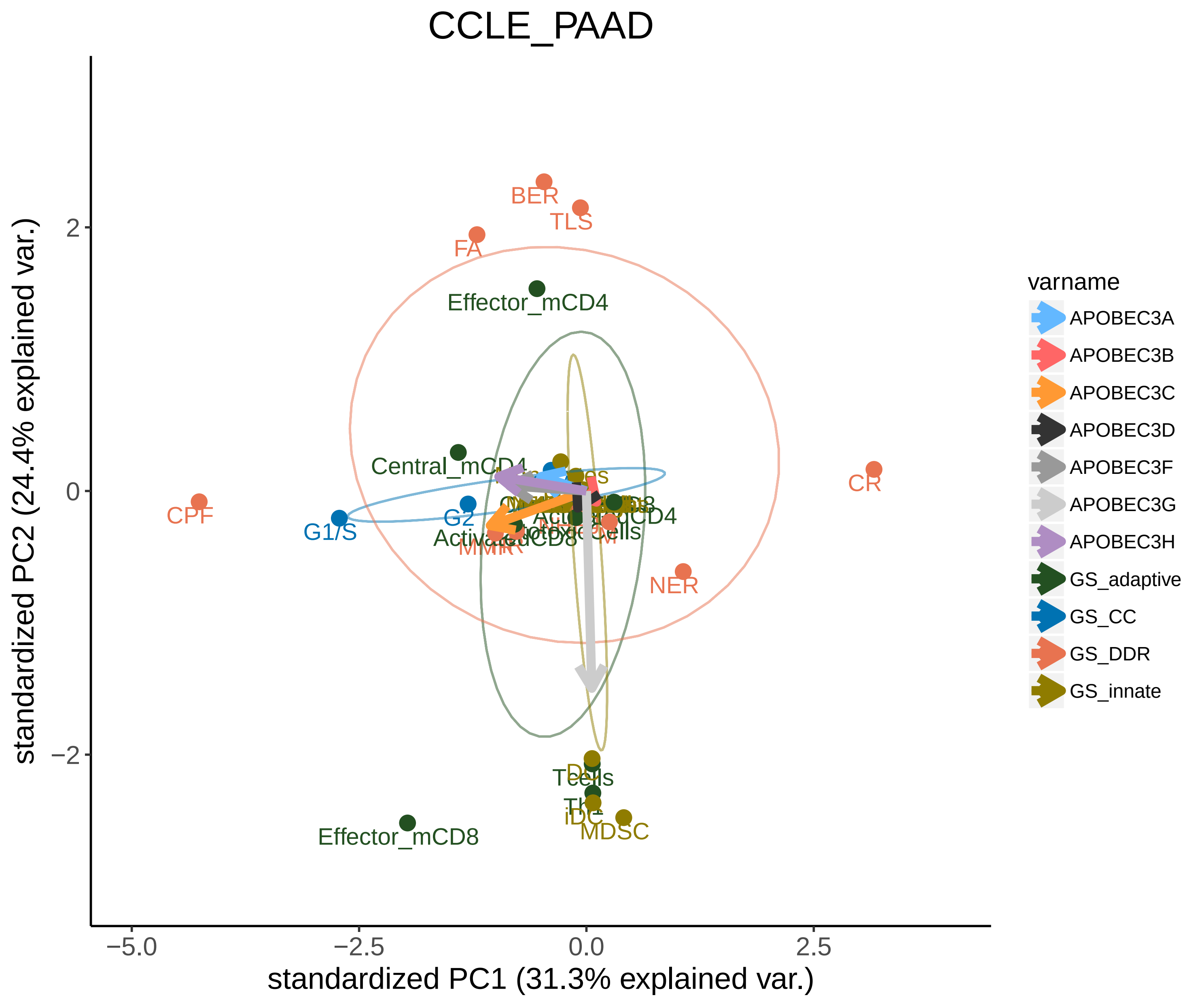

CCLE_PAAD
standardized PC1 (31.3% explained var.)
standardized PC2 (24.4% explained var.)
−5.0
−2.5
0.0
2.5
2
0
−2
BER
TLS
FA
Effector_mCD4
Central_mCD4
CR
CPF
G1/S
G2
NER
Effector_mCD8
MDSC
iDC
Tcells
MMR
varname
APOBEC3A
APOBEC3B
APOBEC3C
APOBEC3D
APOBEC3F
APOBEC3G
APOBEC3H
GS_adaptive
GS_CC
GS_DDR
GS_innate

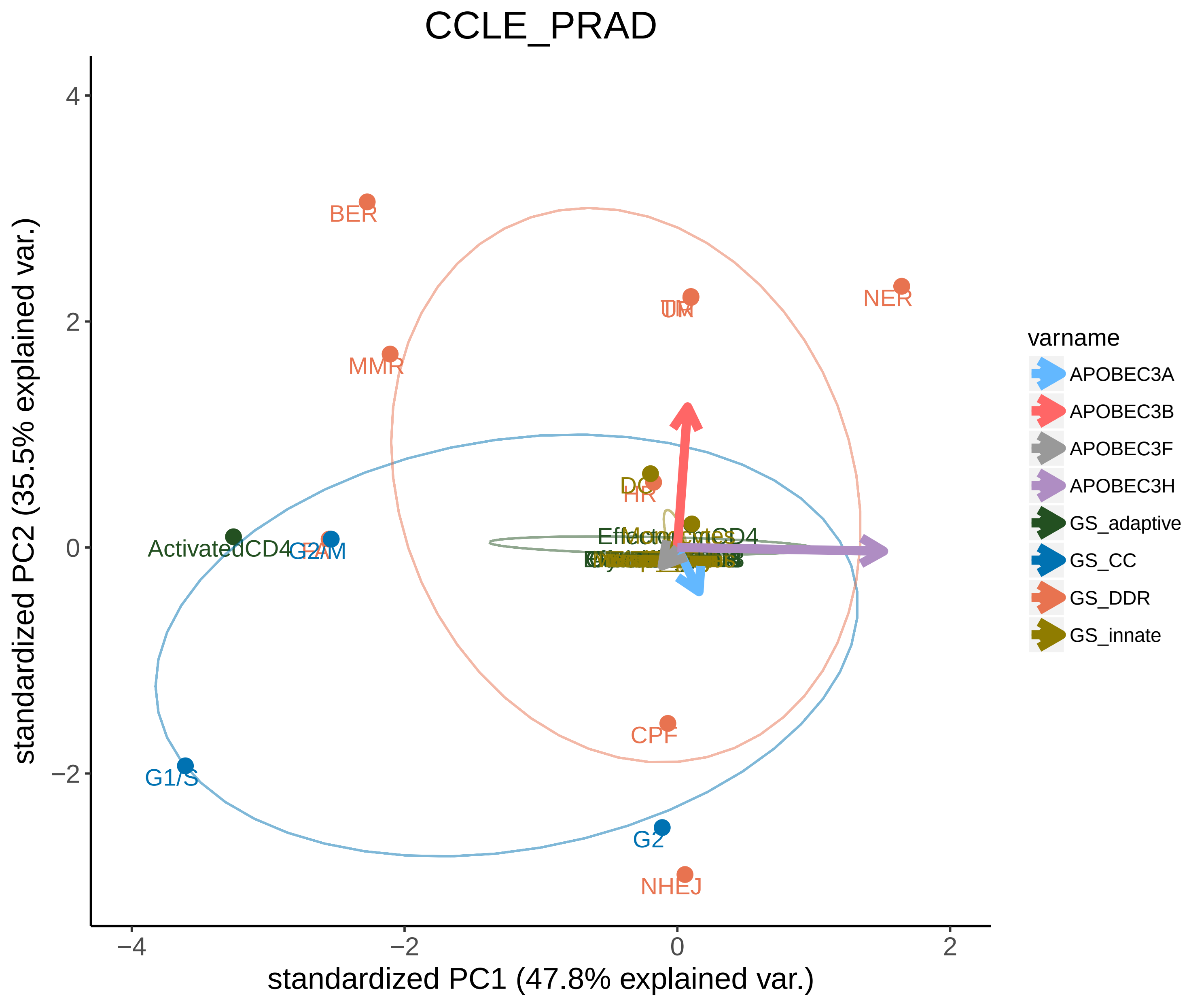
CCLE_PRAD
standardized PC2 (35.5% explained var.)
standardized PC1 (47.8% explained var.)
4
2
0
−2
−4
−2
0
2
BER
MMR
NER
UM
DC
HR
CPF
NHEJ
G1/S
G2
G2/M
ActivatedCD4
Effector memCD4
varname
APOBEC3A
APOBEC3B
APOBEC3F
APOBEC3H
GS_adaptive
GS_CC
GS_DDR
GS_innate

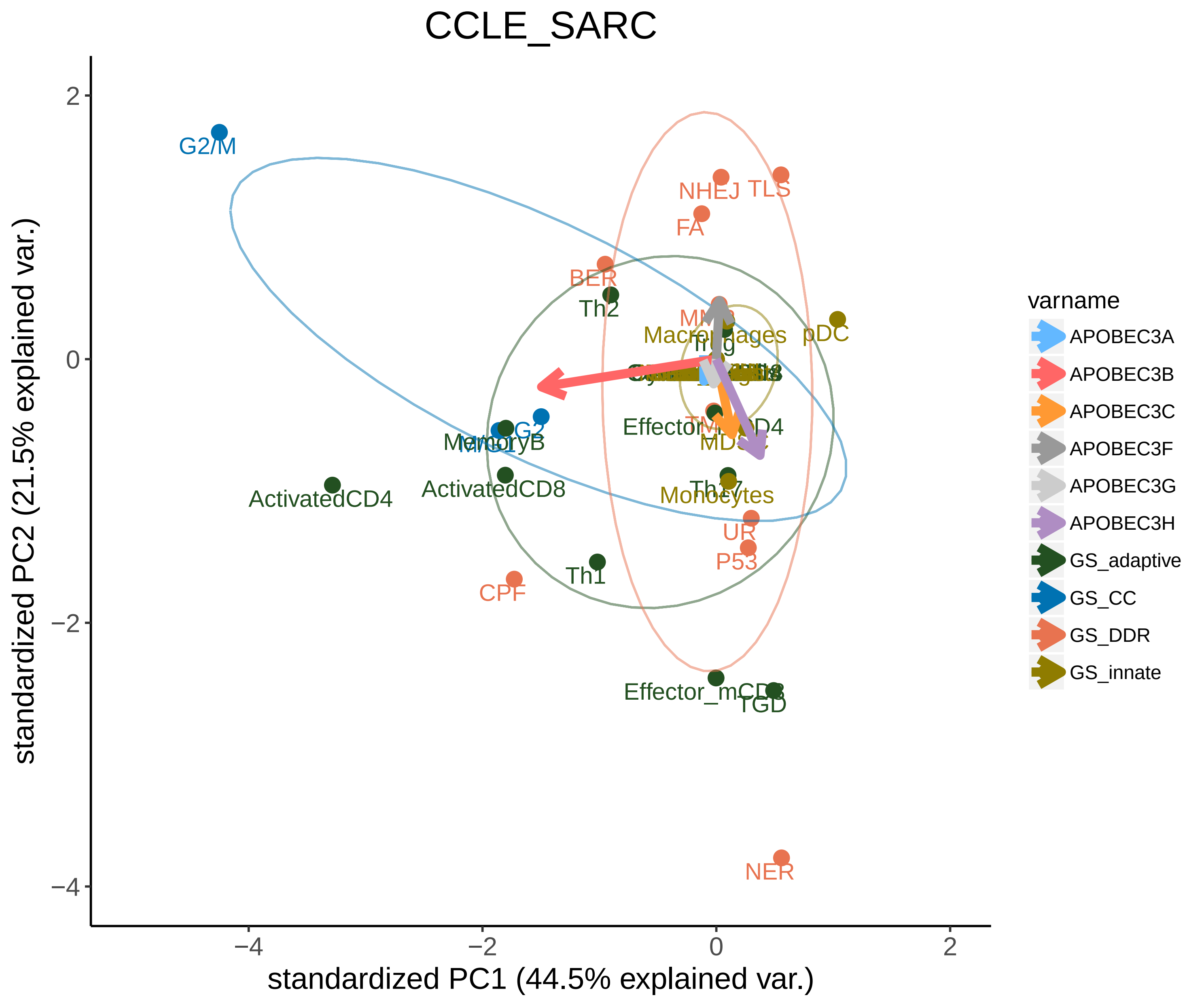

CCLE_SARC
standardized PC1 (44.5% explained var.)
standardized PC2 (21.5% explained var.)
varname
APOBEC3A
APOBEC3B
APOBEC3C
APOBEC3F
APOBEC3G
APOBEC3H
GS_adaptive
GS_CC
GS_DDR
GS_innate
G2/M
NHEJ
TLS
FA
BER
Th2
pDC
Macrophages
ActivatedCD4
ActivatedCD8
MemoryB
G2
Monocytes
UR
P53
Th1
CPF
Effector_mCD8
TGD
NER

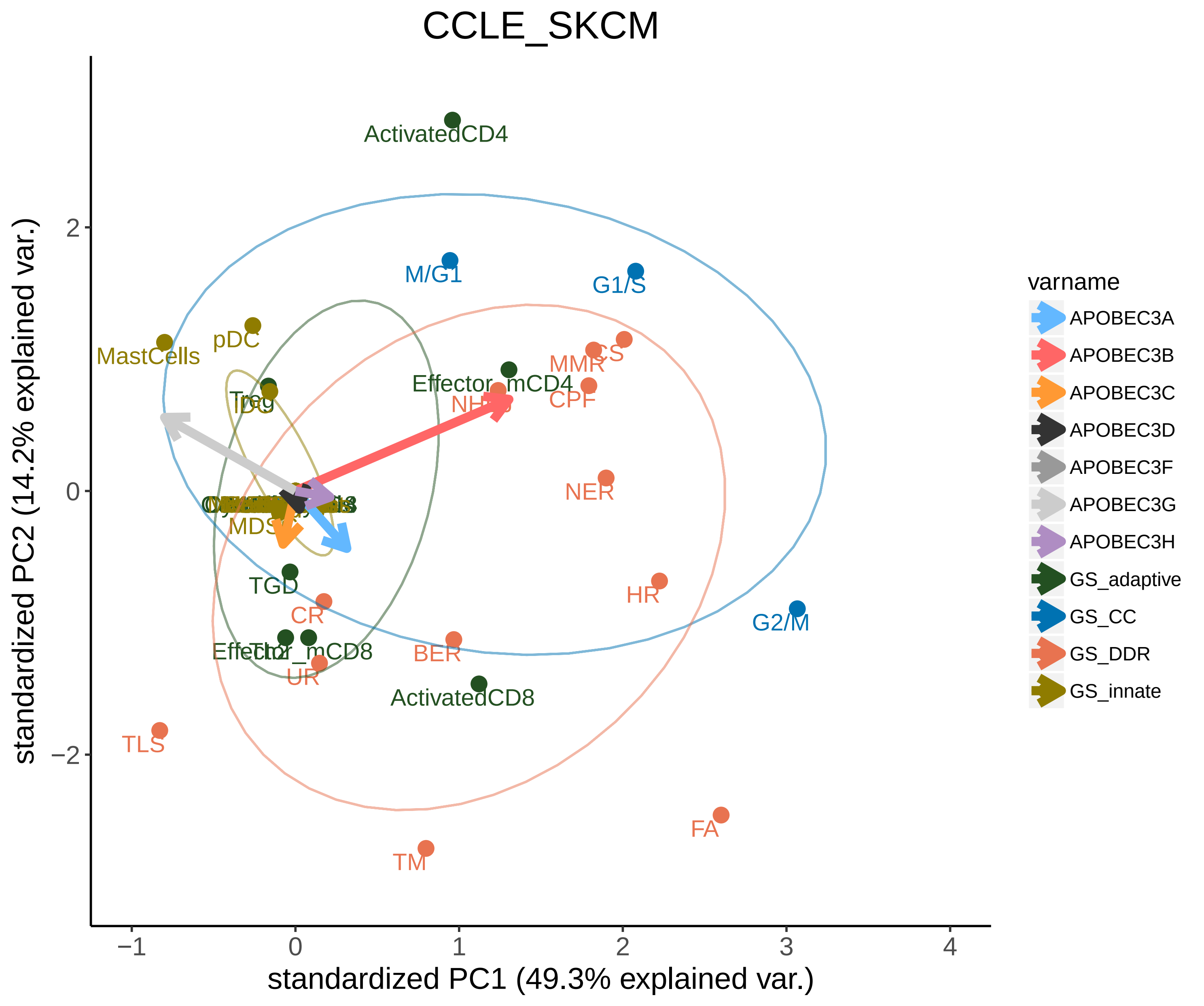
CCLE_SKCM
standardized PC1 (49.3% explained var.)
standardized PC2 (14.2% explained var.)
−1
0
1
2
3
4
2
0
−2
ActivatedCD4
M/G1
G1/S
pDC
MastCells
MMR
CS
Effector_mCD4
CPF
Treg
IDC
NER
MDS
TGD
HR
CR
G2/M
BER
UR
ActivatedCD8
TLS
FA
TM
varname
APOBEC3A
APOBEC3B
APOBEC3C
APOBEC3D
APOBEC3F
APOBEC3G
APOBEC3H
GS_adaptive
GS_CC
GS_DDR
GS_innate

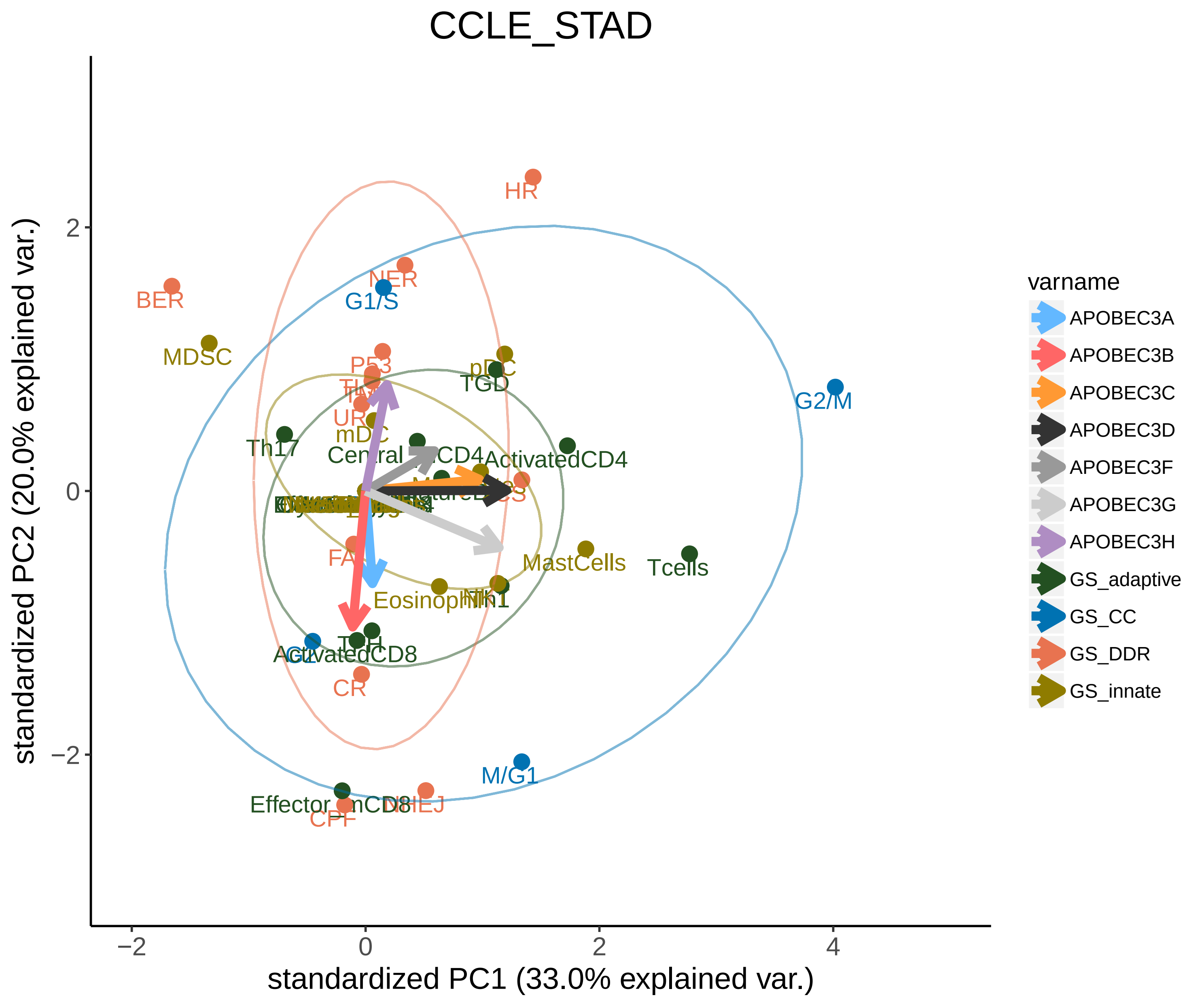

CCLE_STAD
standardized PC1 (33.0% explained var.)
standardized PC2 (20.0% explained var.)
−2
0
2
4
varname
APOBEC3A
APOBEC3B
APOBEC3C
APOBEC3D
APOBEC3F
APOBEC3G
APOBEC3H
GS_adaptive
GS_CC
GS_DDR
GS_innate
HR
BER
NER
G1/S
MDSC
pDC
TGD
G2/M
Th17
mDC
ActivatedCD4
MastCells
Tcells
FA
CR
M/G1
Effector_mCD8
CPF
NHEJ

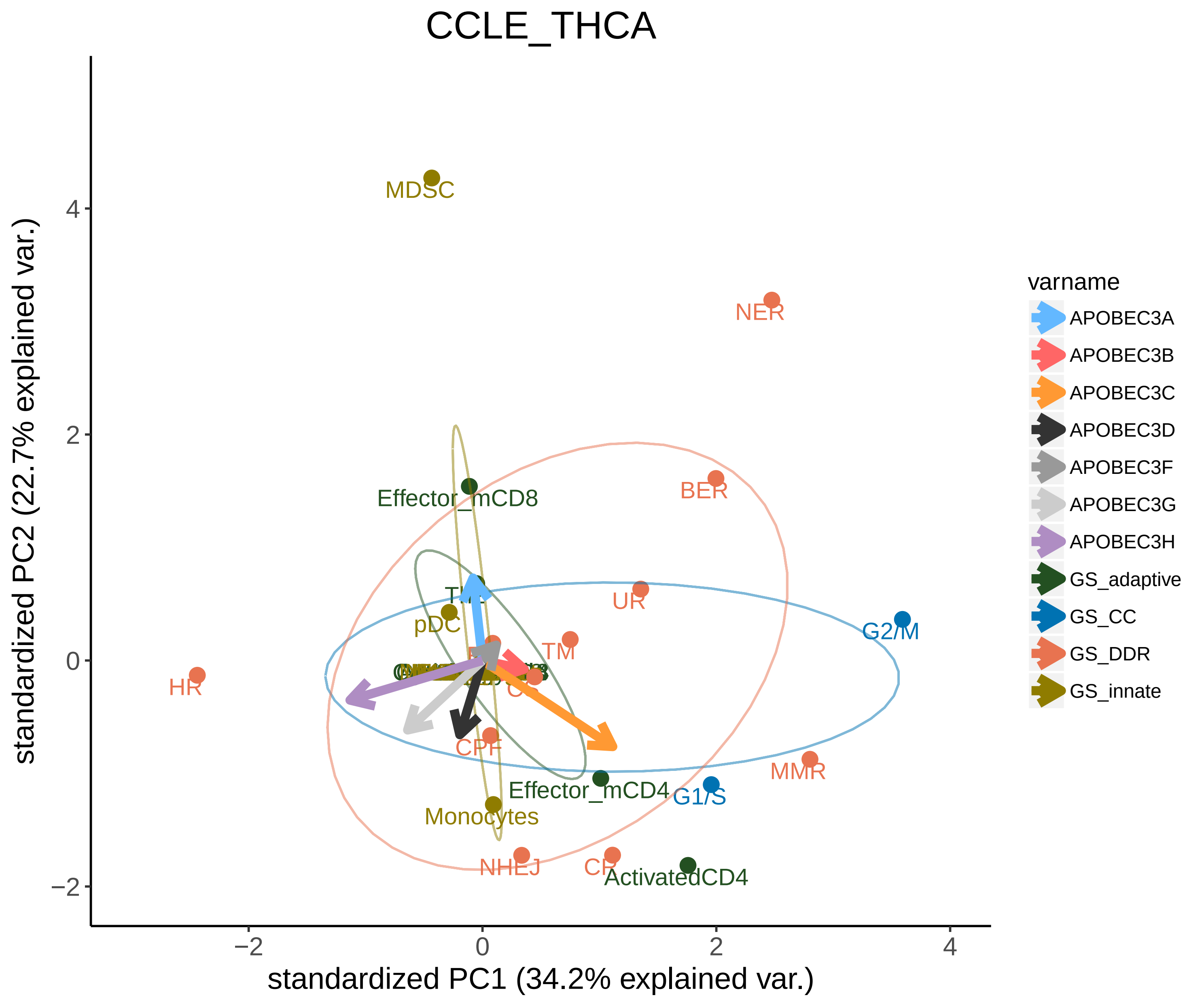

CCLE_THCA
standardized PC2 (22.7% explained var.)
standardized PC1 (34.2% explained var.)
4
2
0
−2
−2
0
2
4
MDSC
NER
BER
Effector_mCD8
UR
G2/M
pDC
TM
HR
CPF
MMR
Effector_mCD4
G1/S
Monocytes
NHEJ
ActivatedCD4
varname
APOBEC3A
APOBEC3B
APOBEC3C
APOBEC3D
APOBEC3F
APOBEC3G
APOBEC3H
GS_adaptive
GS_CC
GS_DDR
GS_innate

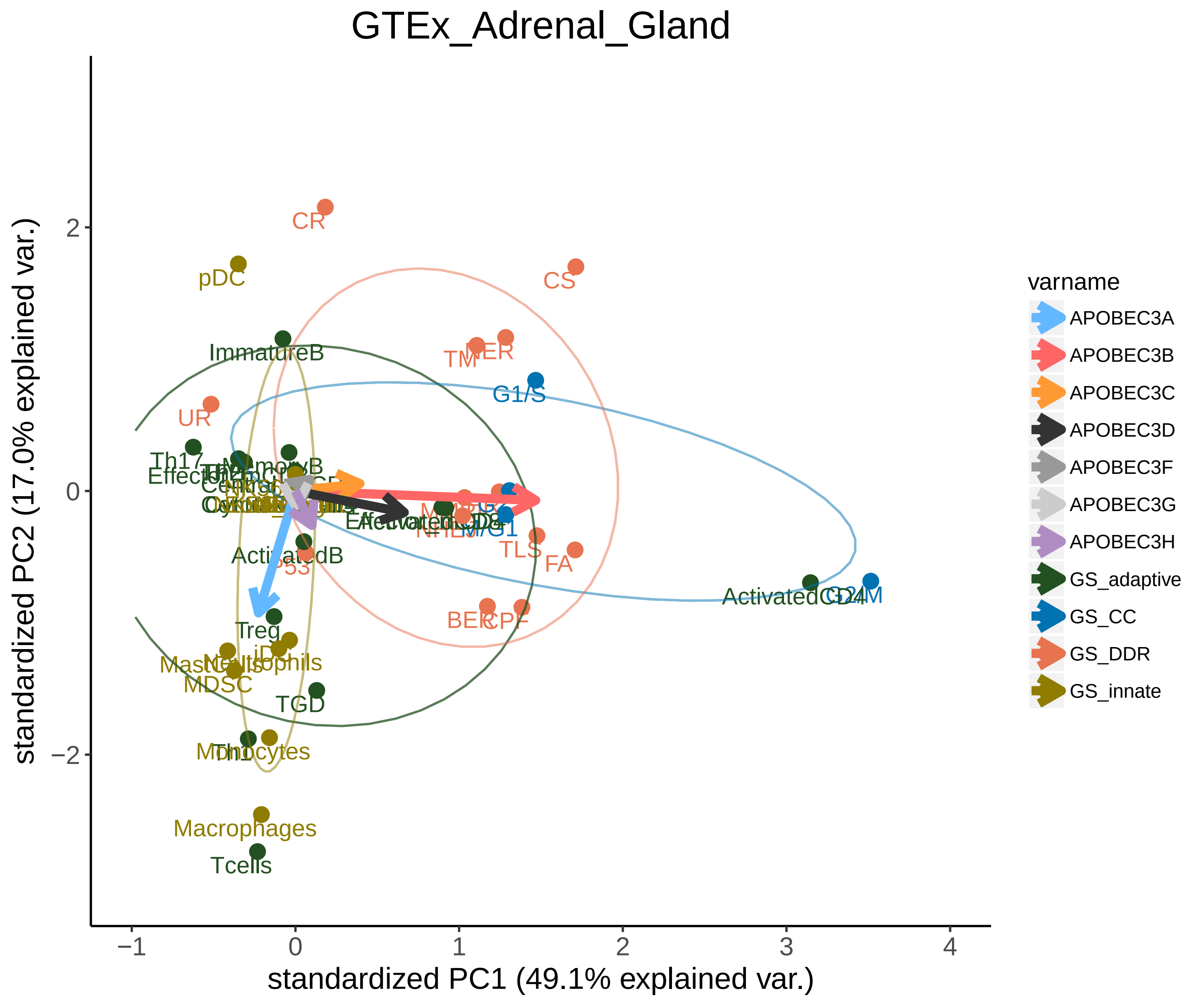

GTEx_Adrenal_Gland
standardized PC1 (49.1% explained var.)
standardized PC2 (17.0% explained var.)
varname
APOBEC3A
APOBEC3B
APOBEC3C
APOBEC3D
APOBEC3F
APOBEC3G
APOBEC3H
GS_adaptive
GS_CC
GS_DDR
GS_innate
CR
pDC
CS
ImmatureB
TM
G1/S
UR
Th17
ActivatedB
ActivatedCD4
Treg
MDSC
TGD
Macrophages
Tcells
TLS
FA
BER

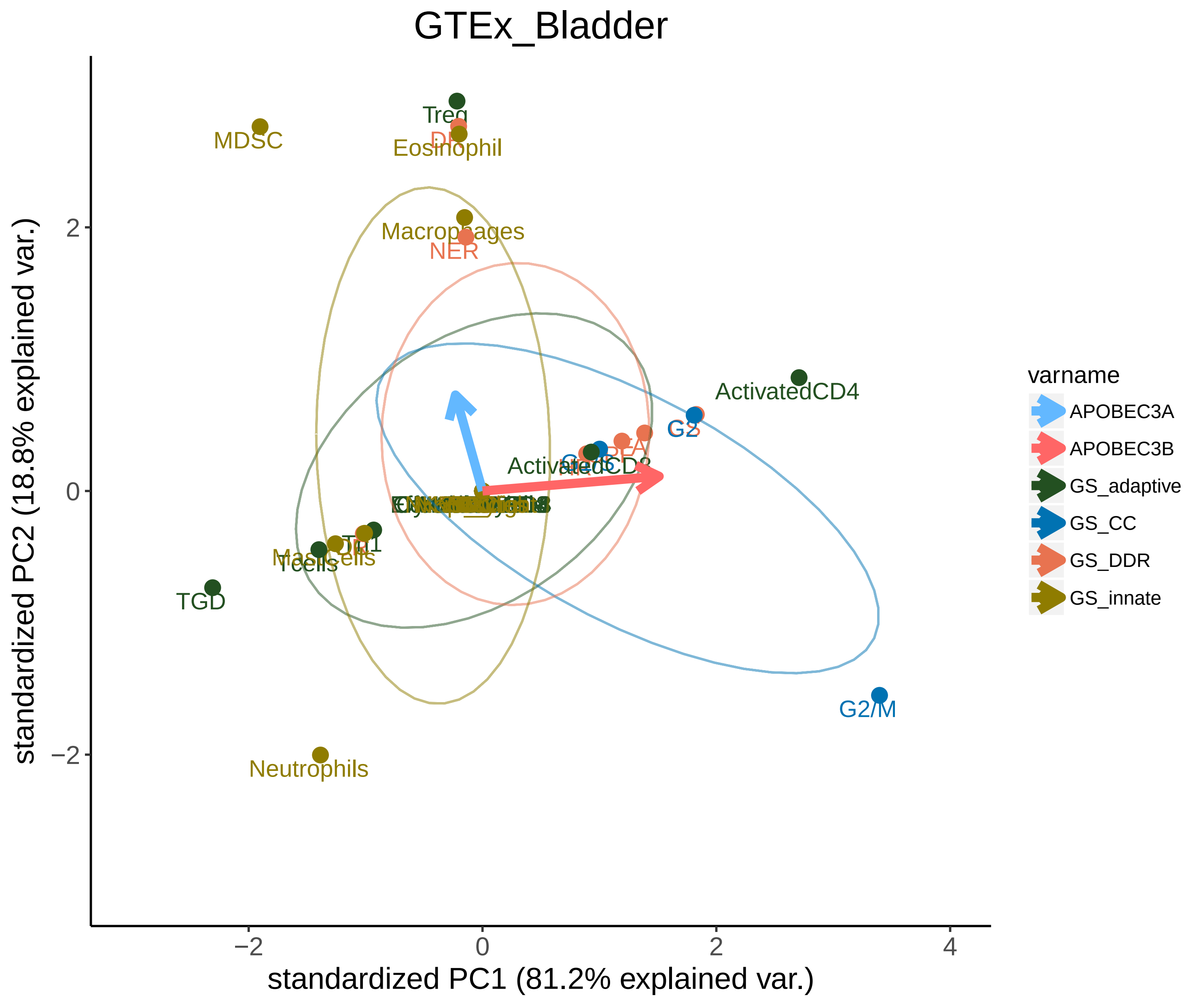
GTEx_Bladder
standardized PC1 (81.2% explained var.)
standardized PC2 (18.8% explained var.)
−2
0
2
4
2
0
−2
Treg
MDSC
Eosinophil
Macrophages
NER
ActivatedCD4
G2
TGD
Neutrophils
G2/M
varname
APOBEC3A
APOBEC3B
GS_adaptive
GS_CC
GS_DDR
GS_innate

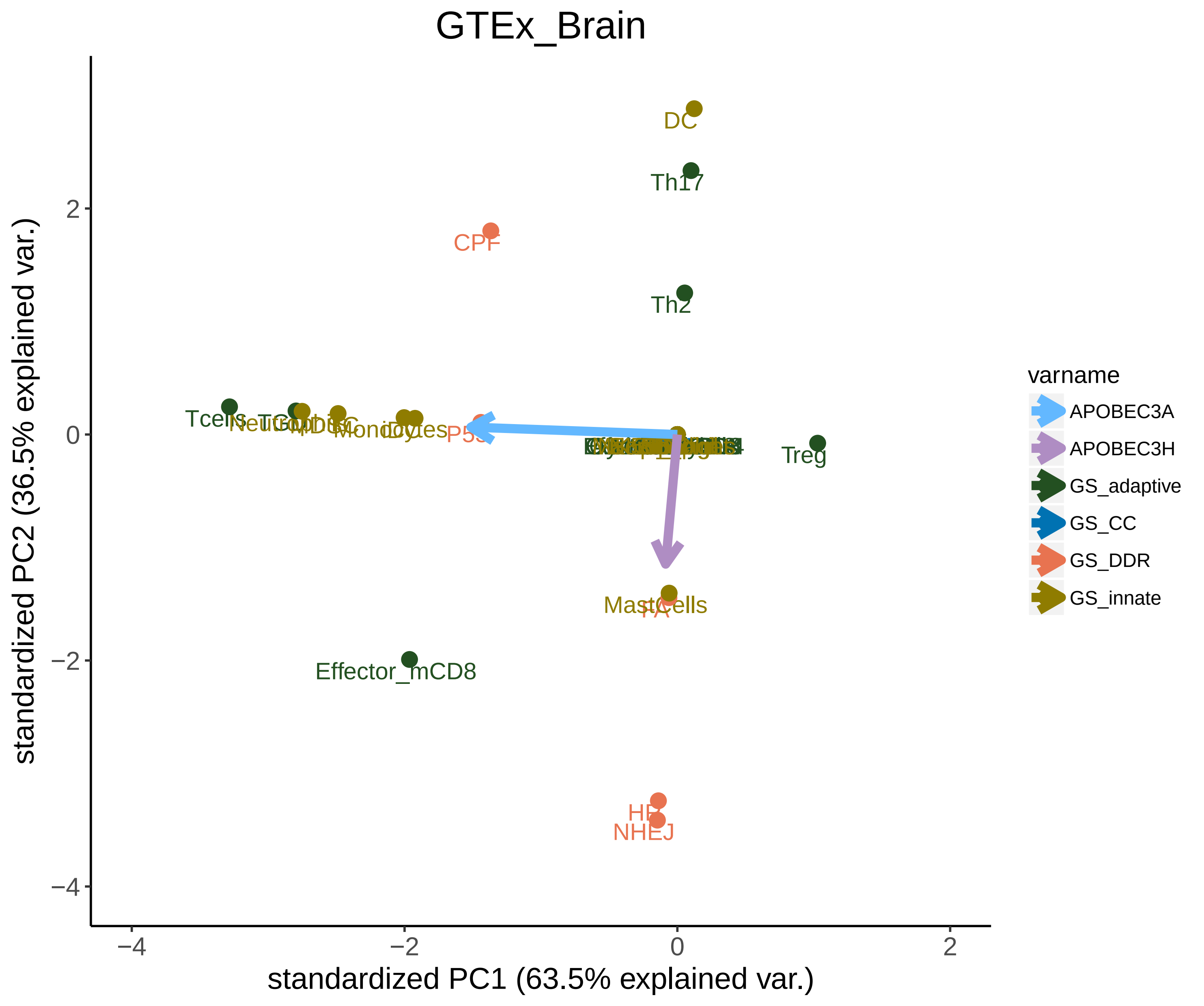
GTEx_Brain
standardized PC2 (36.5% explained var.)
standardized PC1 (63.5% explained var.)
2
0
−2
−4
−4
−2
0
2
DC
Th17
CPF
Th2
Tcells
Treg
Mast Cells
FA
Effector_mCD8
HR
NHEJ
varname
APOBEC3A
APOBEC3H
GS_adaptive
GS_CC
GS_DDR
GS_innate

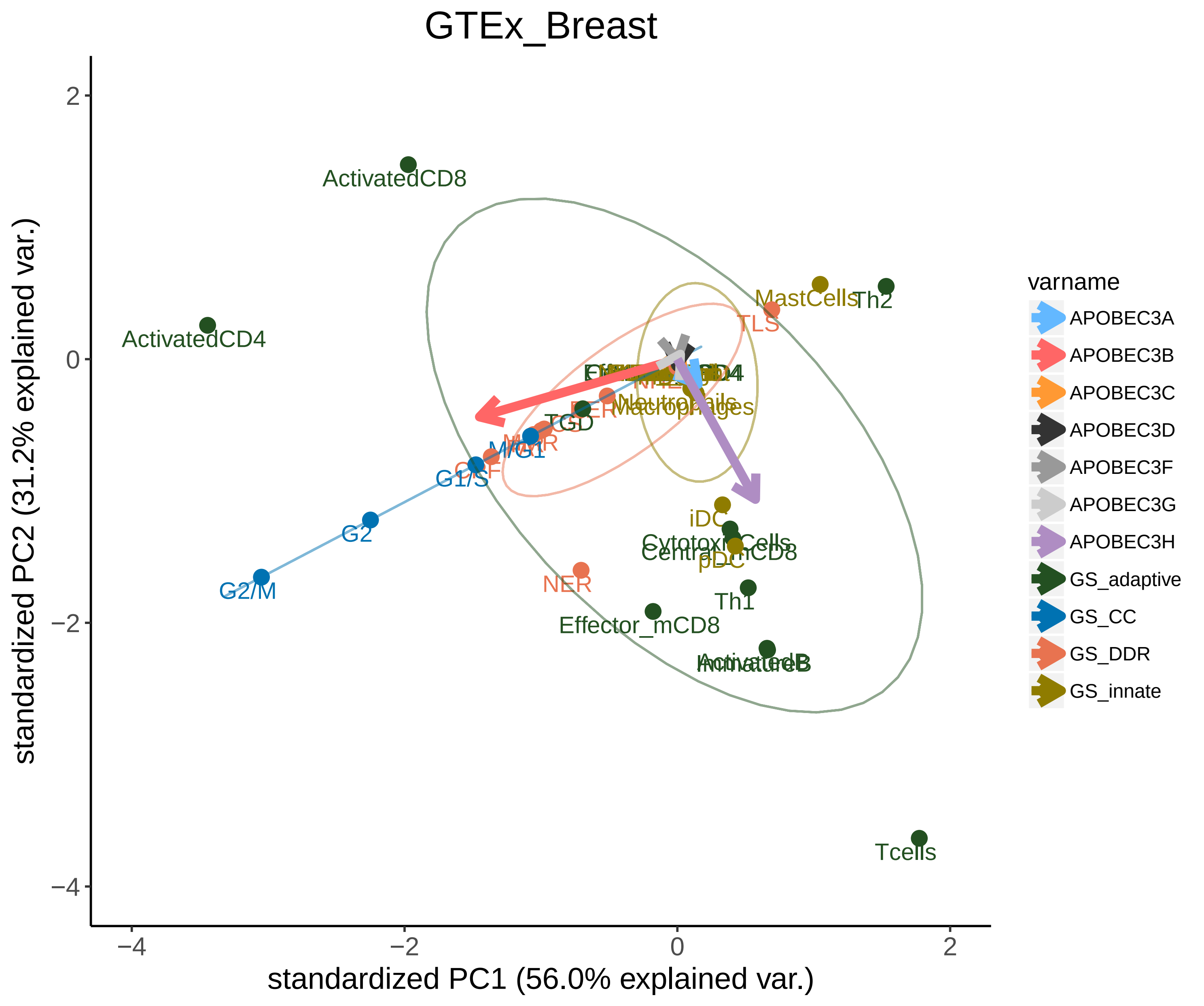
GTEx_Breast
standardized PC2 (31.2% explained var.)
standardized PC1 (56.0% explained var.)
2
0
−2
−4
−4
−2
0
2
ActivatedCD8
ActivatedCD4
MastCells
Th2
TLS
Neutrophils
Macrophages
TGD
M/G1
G1/S
G2
G2/M
iDC
pDC
NER
Th1
Effector_mCD8
Tcells
varname
APOBEC3A
APOBEC3B
APOBEC3C
APOBEC3D
APOBEC3F
APOBEC3G
APOBEC3H
GS_adaptive
GS_CC
GS_DDR
GS_innate

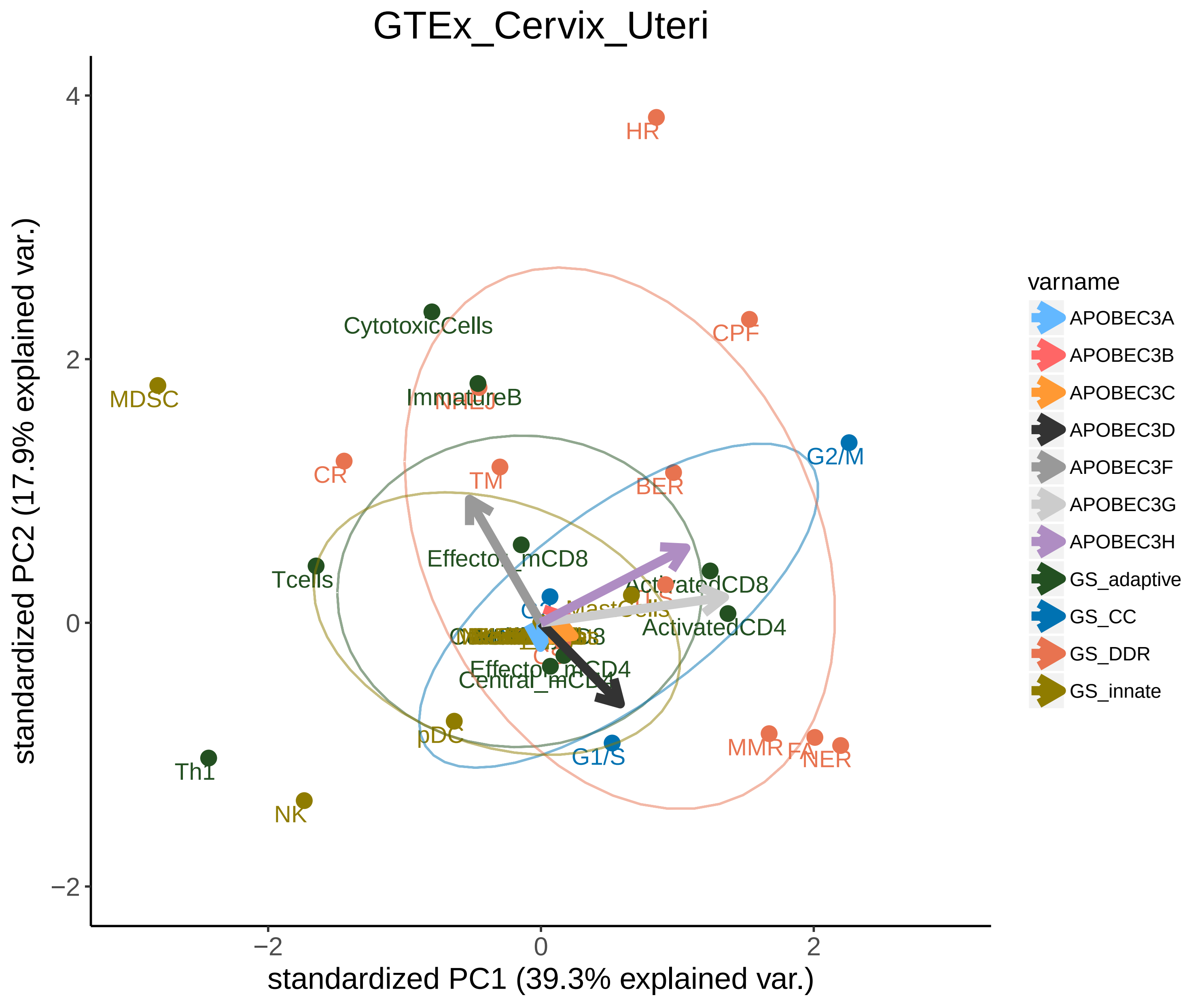
GTEx_Cervix_Uteri
standardized PC2 (17.9% explained var.)
standardized PC1 (39.3% explained var.)
4
2
0
−2
−2
0
2
HR
CytotoxicCells
CPF
MDSC
ImmatureB
NHEJ
G2/M
CR
TM
BER
Effector_mCD8
Tcells
ActivatedCD8
MastCells
ActivatedCD4
Effector_mCD4
Central_mCD4
pDC
MMR
FA
NER
G1/S
Th1
NK
varname
APOBEC3A
APOBEC3B
APOBEC3C
APOBEC3D
APOBEC3F
APOBEC3G
APOBEC3H
GS_adaptive
GS_CC
GS_DDR
GS_innate

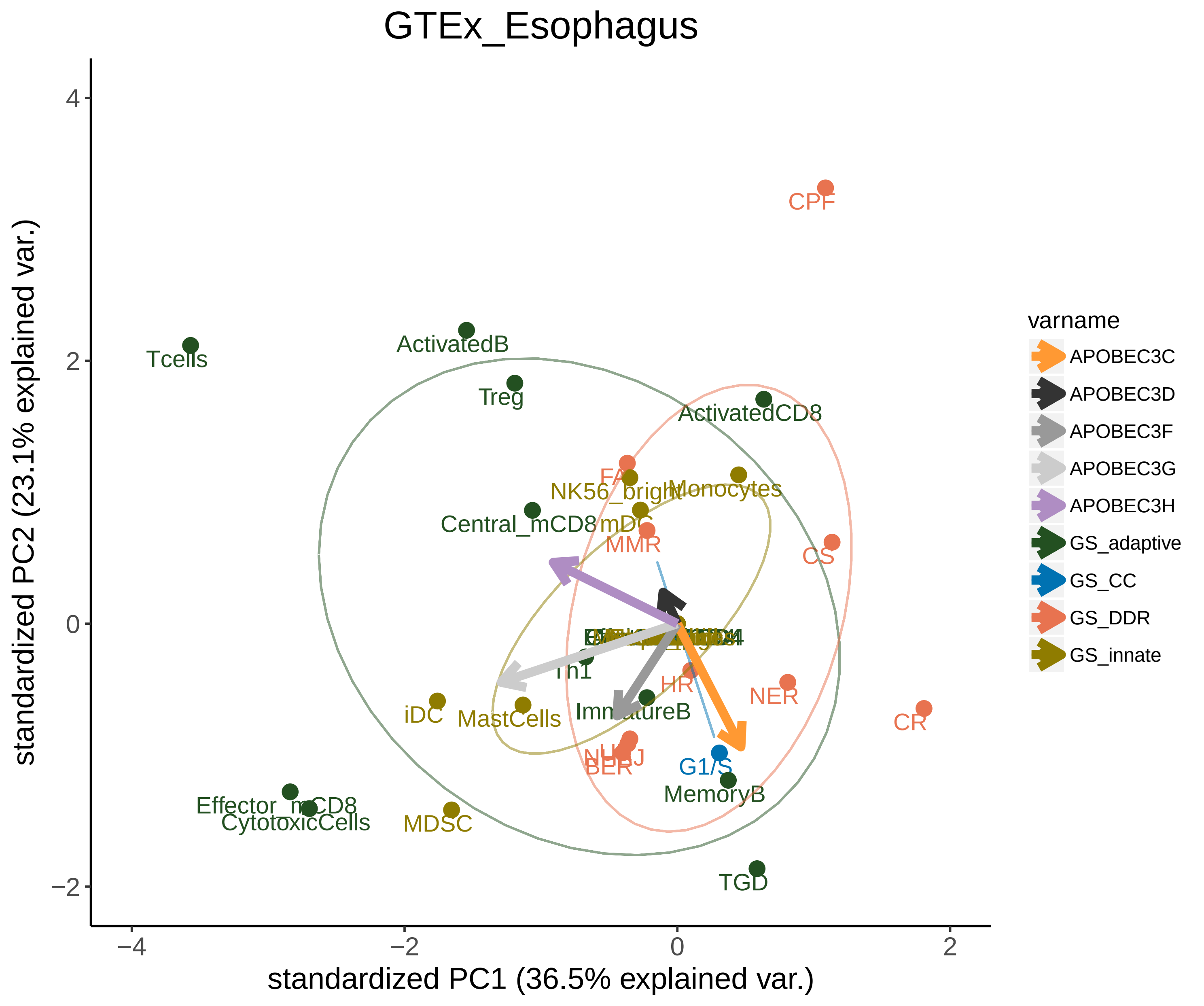

GTEx_Esophagus
standardized PC1 (36.5% explained var.)
standardized PC2 (23.1% explained var.)
varname
APOBEC3C
APOBEC3D
APOBEC3F
APOBEC3G
APOBEC3H
GS_adaptive
GS_CC
GS_DDR
GS_innate
Tcells
ActivatedB
Treg
ActivatedCD8
CPF
Monocytes
Central_mCD8
MMR
CS
Th1
HR
NER
iDC
MastCells
CR
G1/S
BER
MemoryB
Effector_mCD8
CytotoxicCells
MDSC
TGD

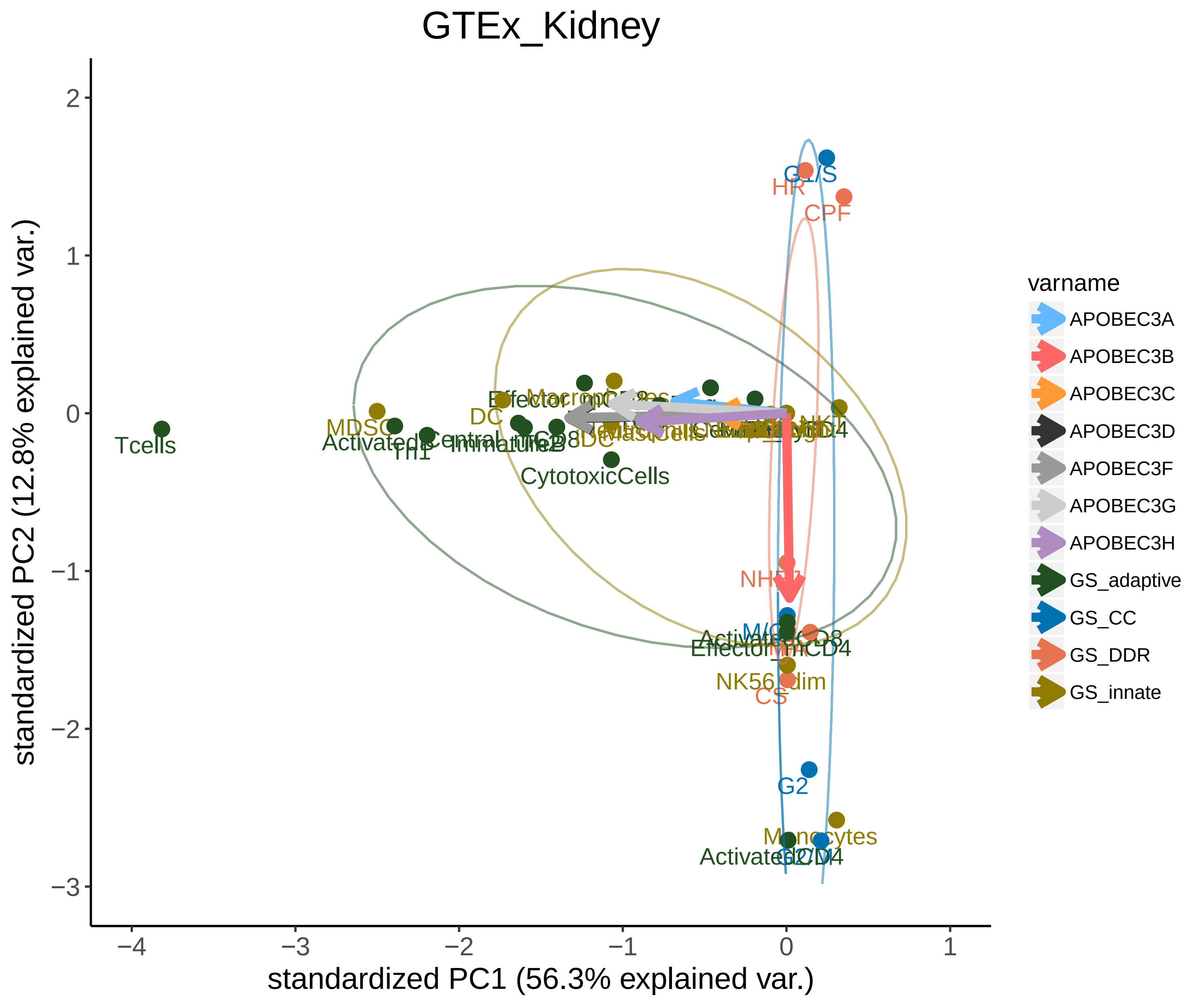
GTEx_Kidney
standardized PC1 (56.3% explained var.)
standardized PC2 (12.8% explained var.)
varname
APOBEC3A
APOBEC3B
APOBEC3C
APOBEC3D
APOBEC3F
APOBEC3G
APOBEC3H
GS_adaptive
GS_CC
GS_DDR
GS_innate
Tcells
MDSC
DC
CytotoxicCells
G1/S
HR
CPF
G2
Monocytes
NK56_dim
CS

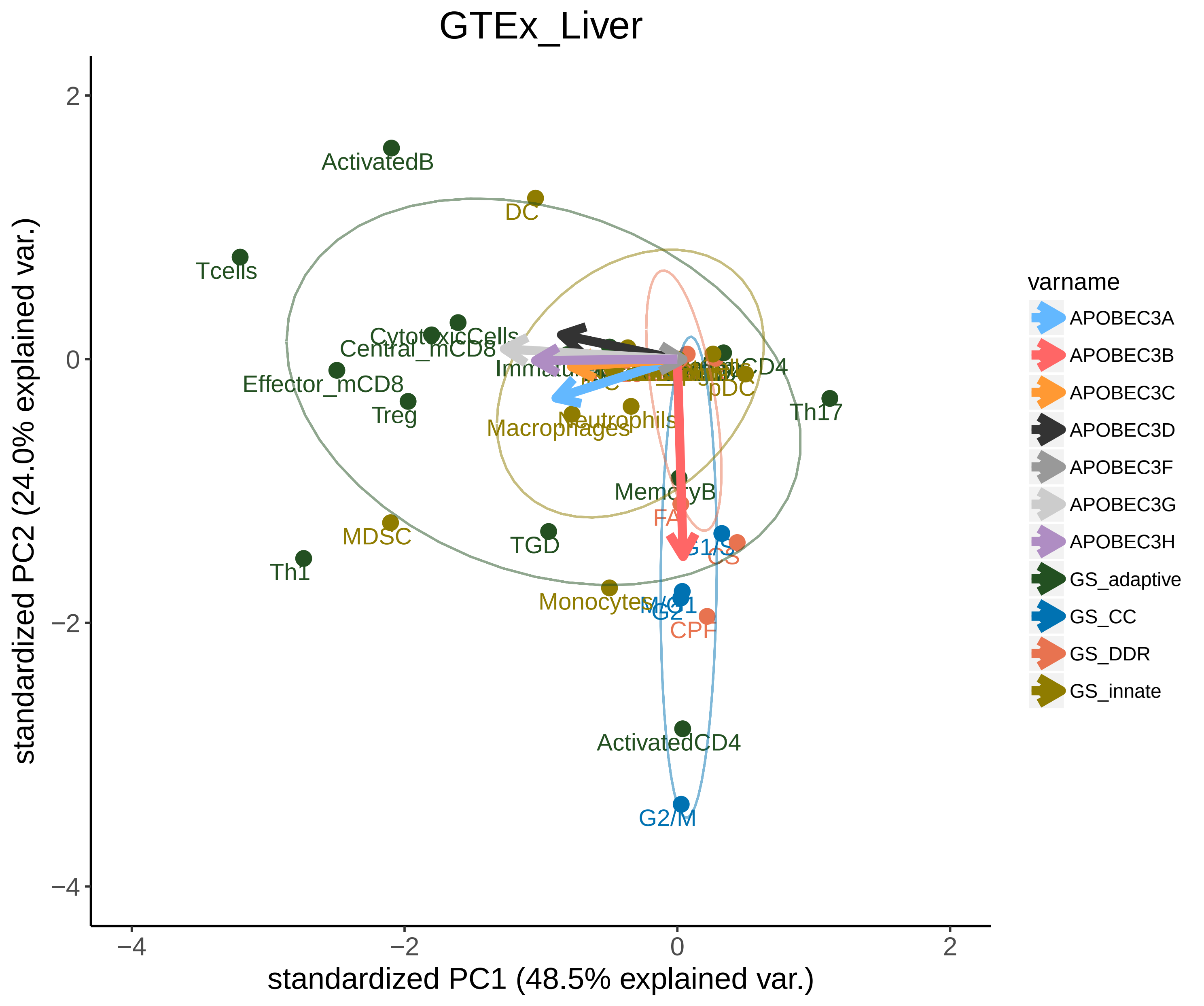

GTEx_Liver
standardized PC1 (48.5% explained var.)
standardized PC2 (24.0% explained var.)
−4
−2
0
2
−4
−2
0
2
varname
APOBEC3A
APOBEC3B
APOBEC3C
APOBEC3D
APOBEC3F
APOBEC3G
APOBEC3H
GS_adaptive
GS_CC
GS_DDR
GS_innate
ActivatedB
DC
Tcells
Central_mCD8
Effector_mCD8
Treg
Macrophages
pDC
Th17
MemoryB
MDSC
TGD
Th1
CPF
ActivatedCD4
G2/M

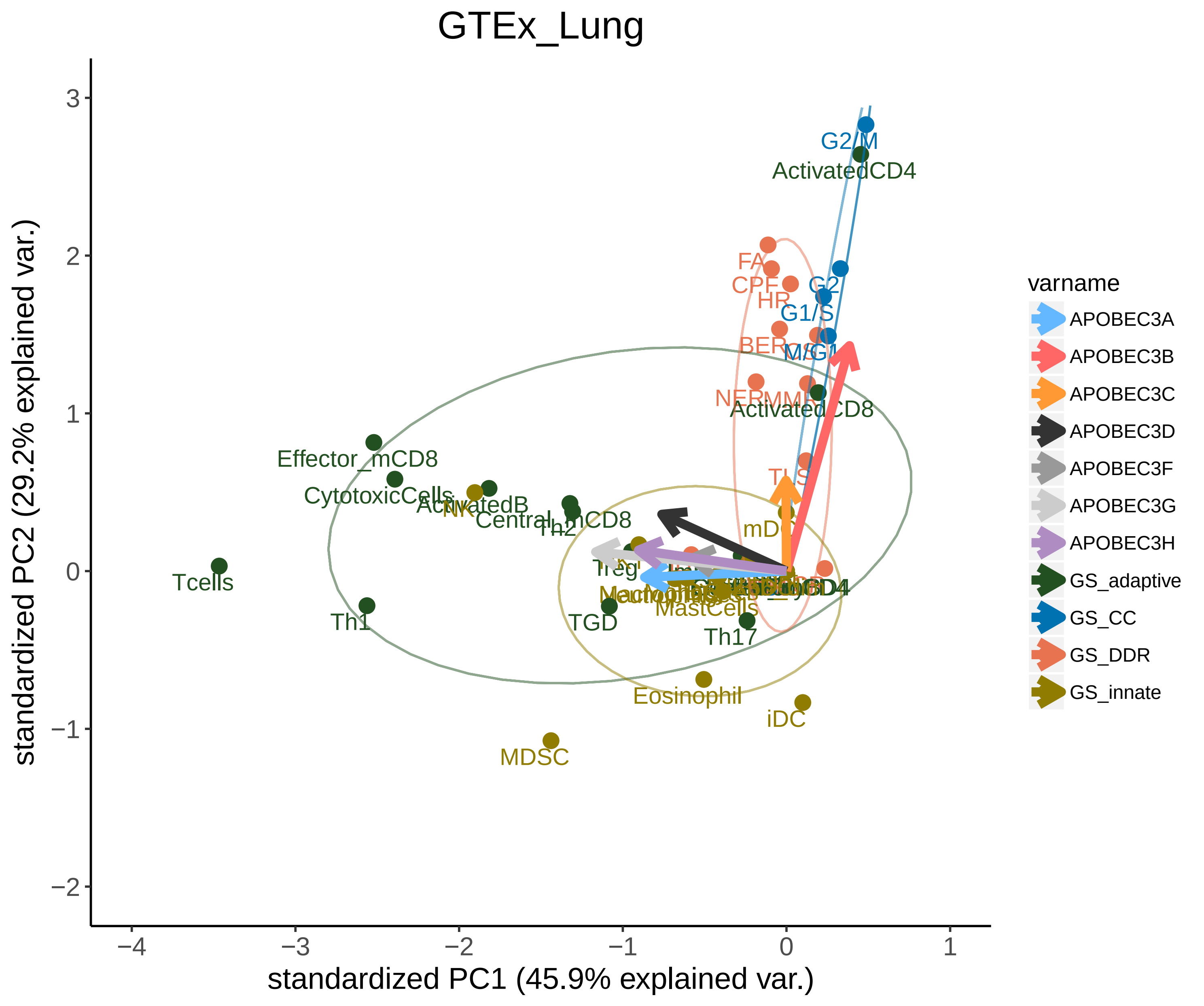
GTEx_Lung
standardized PC2 (29.2% explained var.)
standardized PC1 (45.9% explained var.)
3
2
1
0
−1
−2
−4
−3
−2
−1
0
1
varname
APOBEC3A
APOBEC3B
APOBEC3C
APOBEC3D
APOBEC3F
APOBEC3G
APOBEC3H
GS_adaptive
GS_CC
GS_DDR
GS_innate
G2/M
ActivatedCD4
FA
CPF
HR
G2
G1/S
BER
M/G1
ActivatedCD8
Effector_mCD8
CytotoxicCells
Tcells
Th1
TGD
Th17
MastCells
Eosinophil
iDC
MDSC
mDC

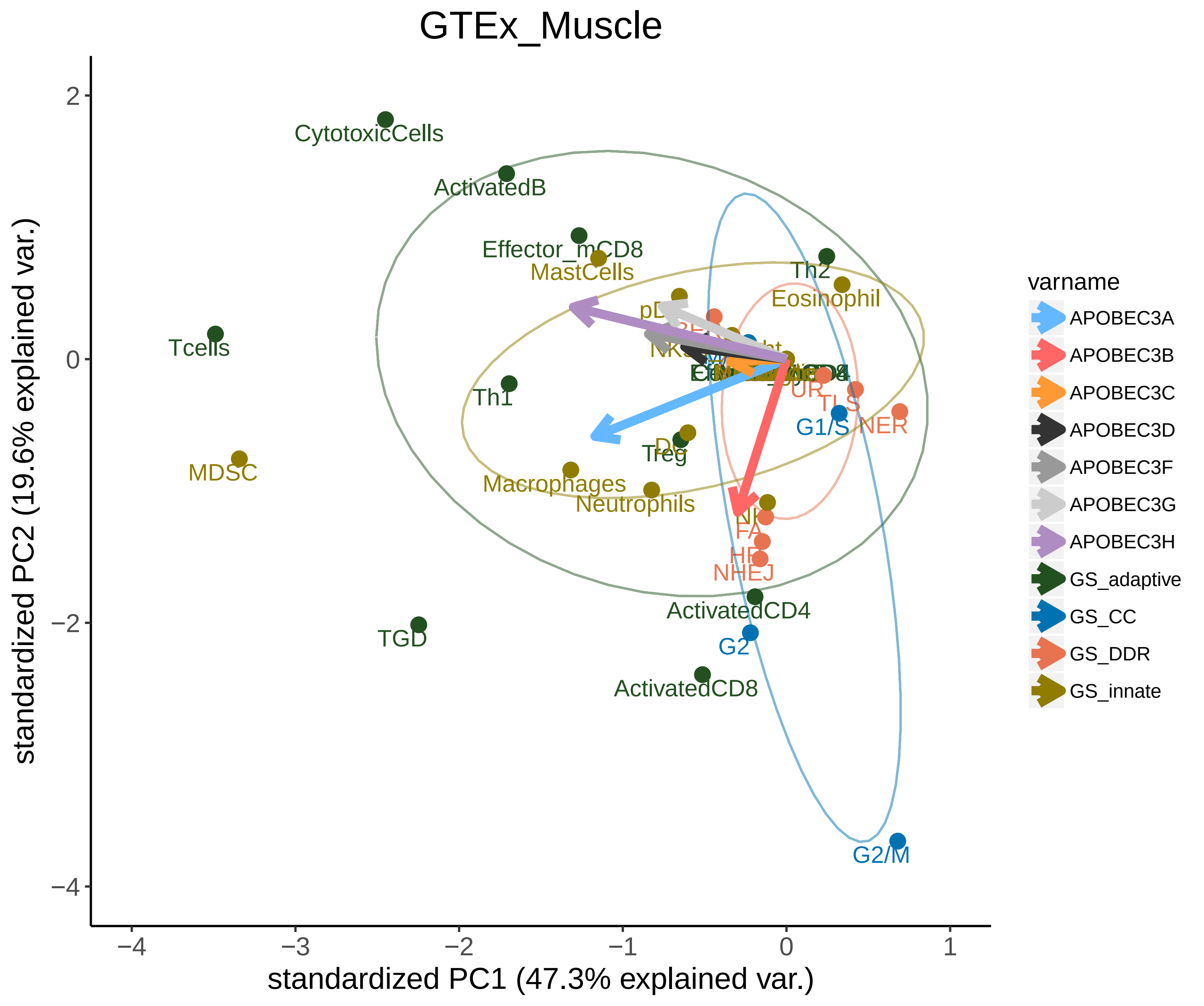

GTEx_Muscle
standardized PC1 (47.3% explained var.)
standardized PC2 (19.6% explained var.)
−4
−3
−2
−1
0
1
2
0
−2
−4
CytotoxicCells
ActivatedB
Effector_mCD8
MastCells
Th2
Eosinophil
Tcells
Th1
MDSC
Macrophages
Neutrophils
Treg
TGD
ActivatedCD4
ActivatedCD8
G1/S
NER
TLS
G2
G2/M
NHEJ
varname
APOBEC3A
APOBEC3B
APOBEC3C
APOBEC3D
APOBEC3F
APOBEC3G
APOBEC3H
GS_adaptive
GS_CC
GS_DDR
GS_innate

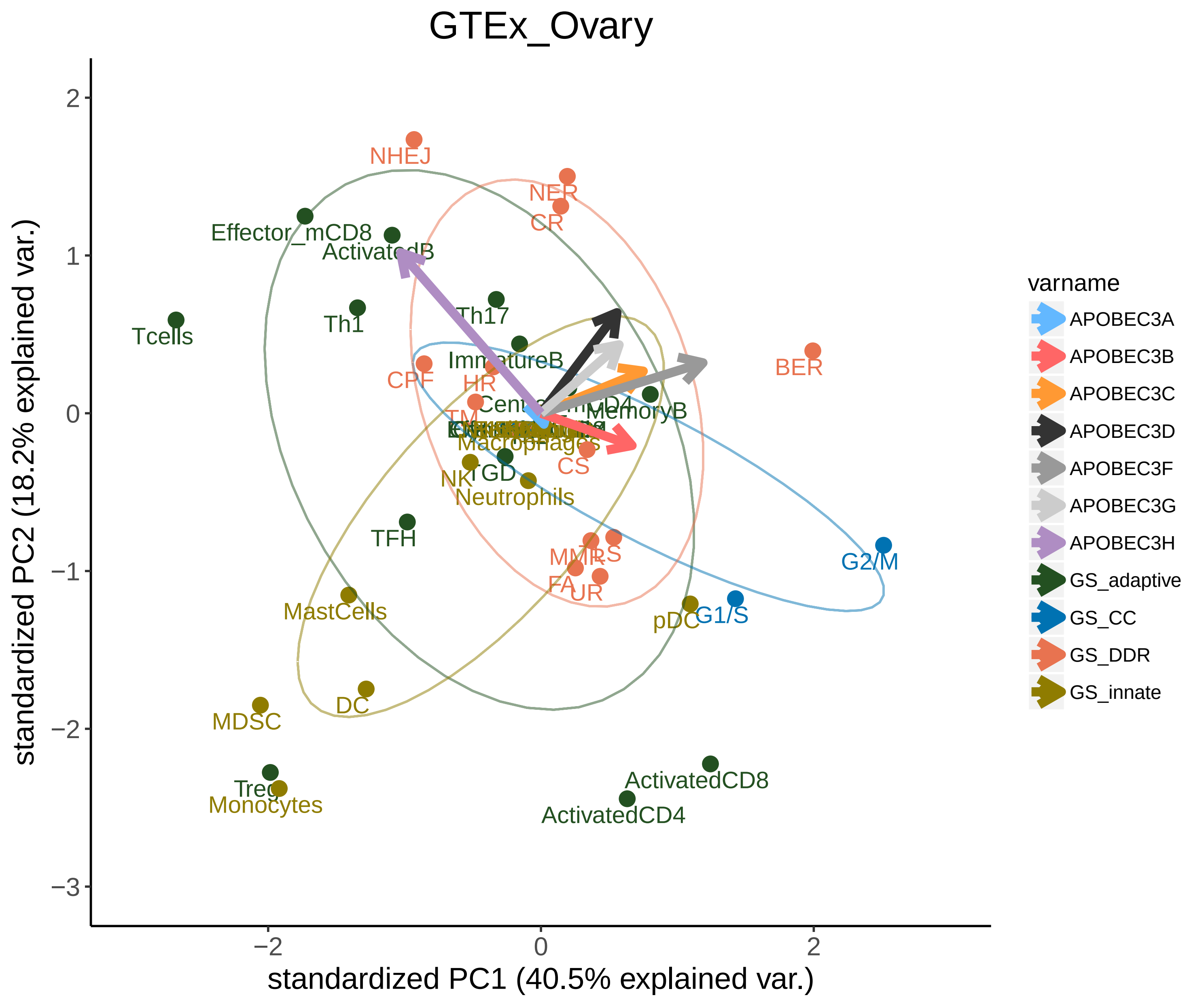
GTEx_Ovary
standardized PC1 (40.5% explained var.)
standardized PC2 (18.2% explained var.)
varname
APOBEC3A
APOBEC3B
APOBEC3C
APOBEC3D
APOBEC3F
APOBEC3G
APOBEC3H
GS_adaptive
GS_CC
GS_DDR
GS_innate
NHEJ
NER
CR
Effector_mCD8
Tcells
Th1
Th17
CPF
HR
MemoryB
Macrophages
CS
NK
TGD
Neutrophils
TFH
BER
G2/M
MMR
FA
UR
pDC
G1/S
MastCells
DC
MDSC
Treg
Monocytes
ActivatedCD8
ActivatedCD4
2
1
0
−1
−2
−3
−2
0
2

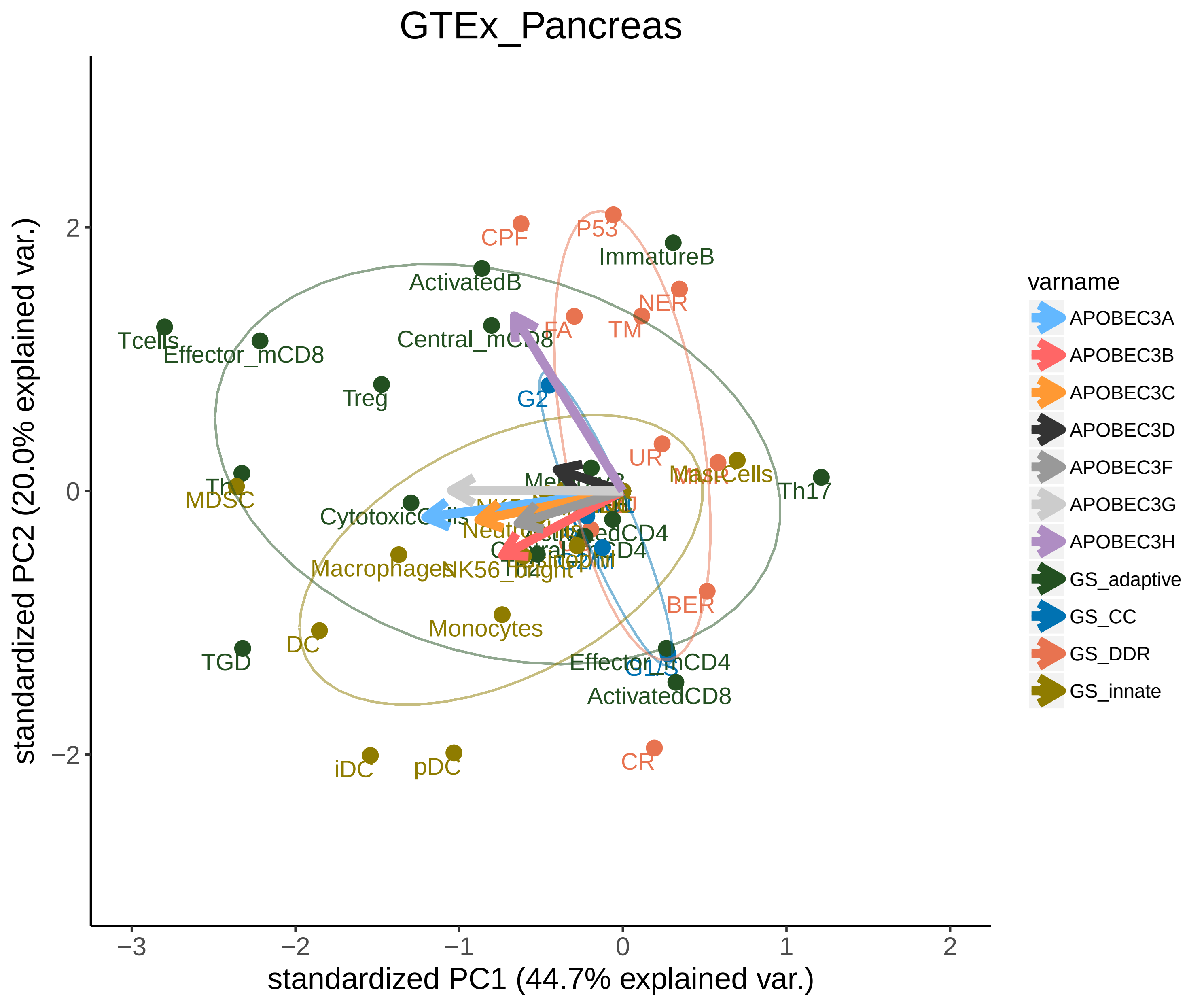
GTEx_Pancreas
standardized PC1 (44.7% explained var.)
standardized PC2 (20.0% explained var.)
−3
−2
−1
0
1
2
−2
0
2
varname
APOBEC3A
APOBEC3B
APOBEC3C
APOBEC3D
APOBEC3F
APOBEC3G
APOBEC3H
GS_adaptive
GS_CC
GS_DDR
GS_innate
CPF
P53
ImmatureB
ActivatedB
NER
Tcells
Effector_mCD8
FA
TM
Treg
G2
UR
Th17
MDSC
Macrophages
Monocytes
BER
DC
TGD
ActivatedCD8
CR
iDC
pDC

GTEx_Prostate

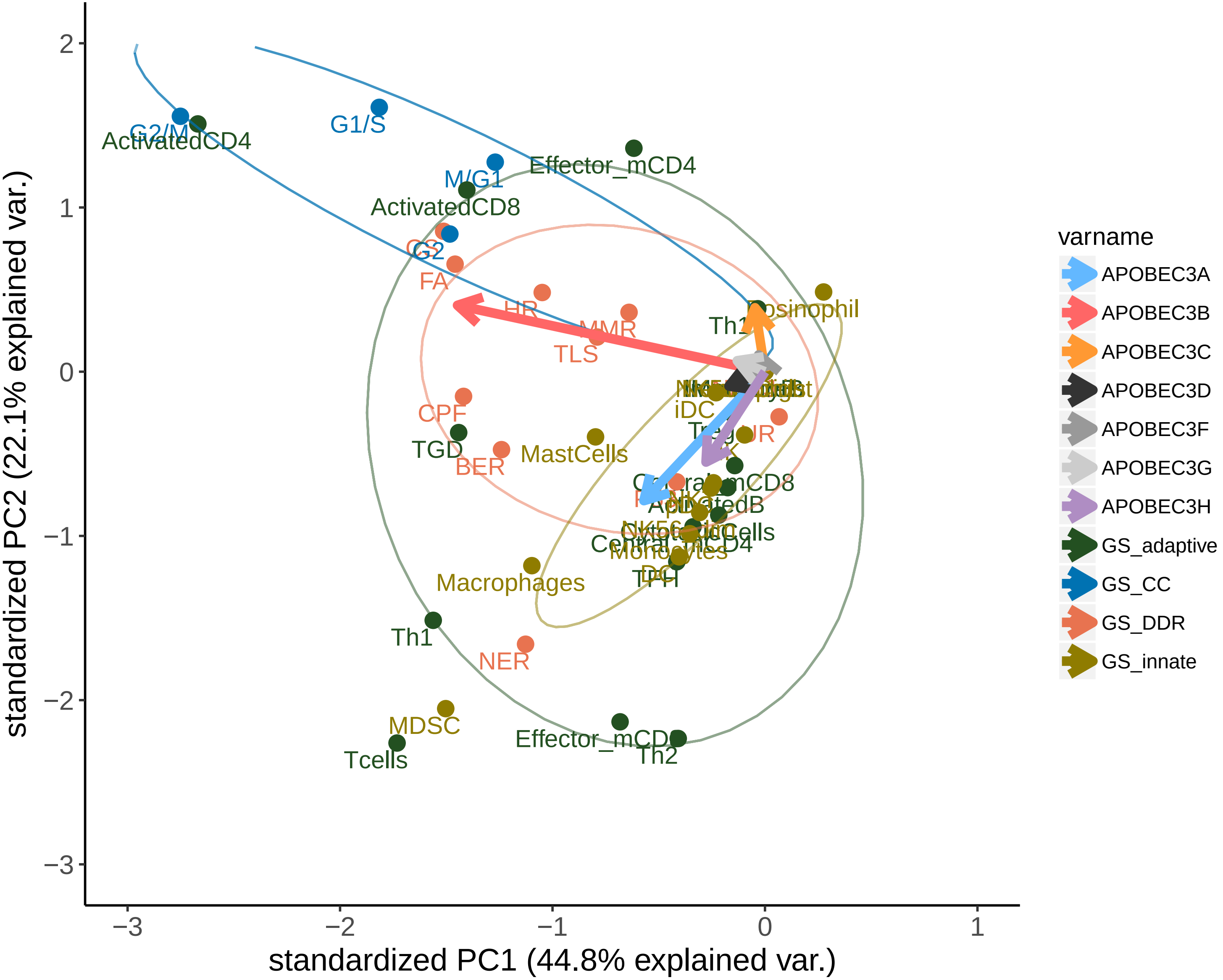

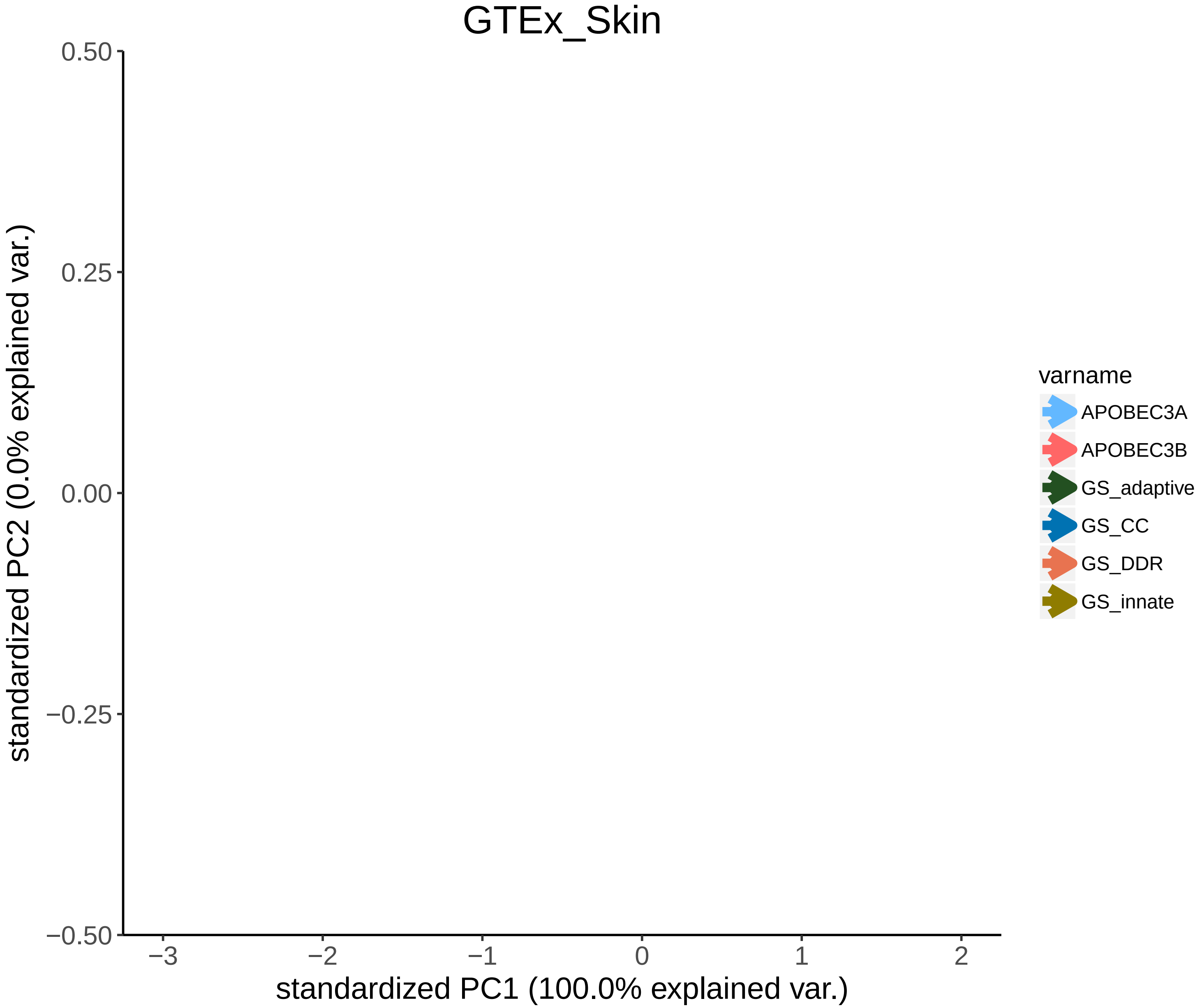
GTEx_Skin
0.50
0.25
0.00
−0.25
−0.50
standardized PC2 (0.0% explained var.)
−3
−2
−1
0
1
2
standardized PC1 (100.0% explained var.)
varname
APOBEC3A
APOBEC3B
GS_adaptive
GS_CC
GS_DDR
GS_innate

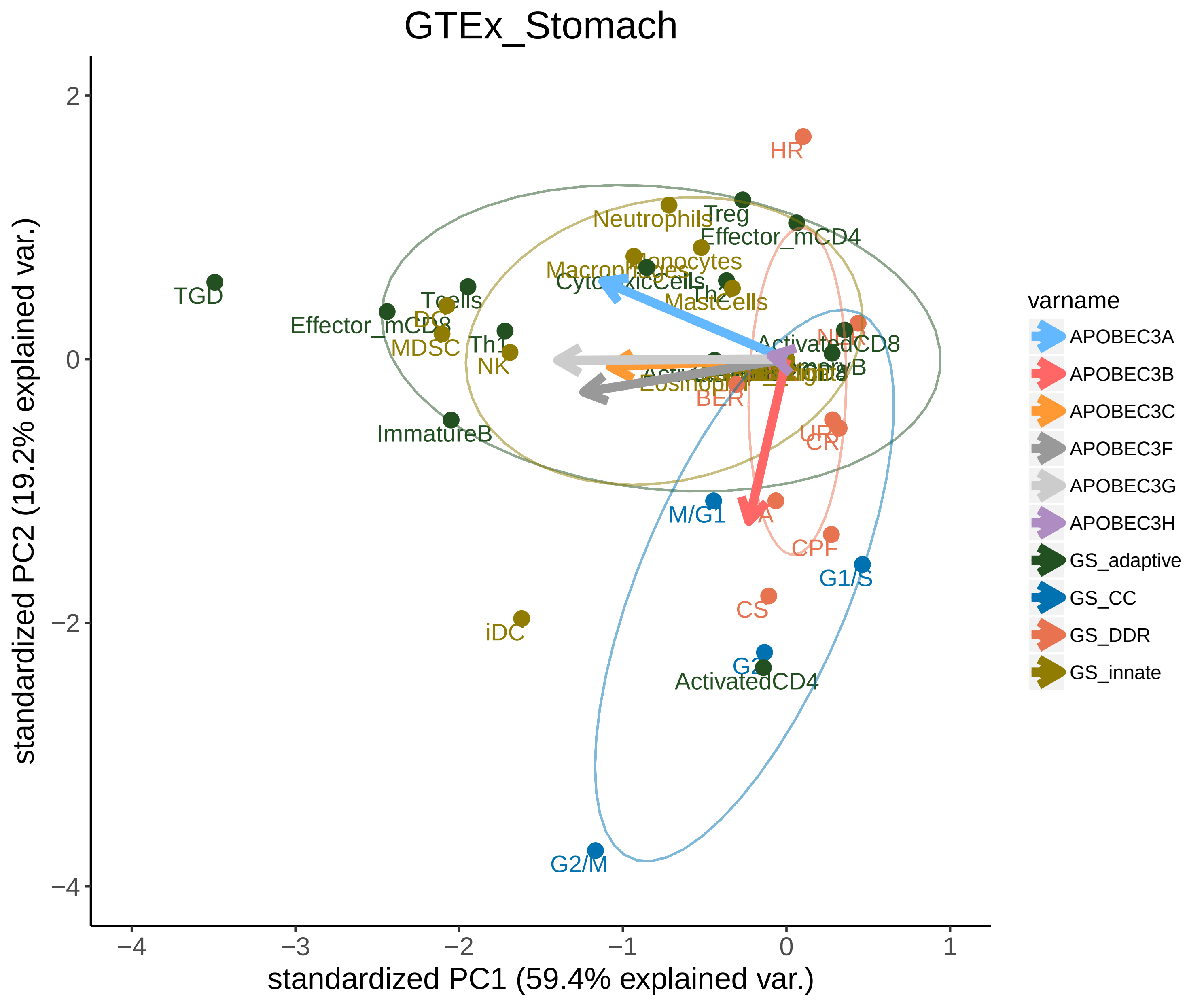
GTEx_Stomach
standardized PC2 (19.2% explained var.)
standardized PC1 (59.4% explained var.)
2
0
−2
−4
−4
−3
−2
−1
0
1
HR
Treg
Neutrophils
Effector_mCD4
Monocytes
Macrophages
CytotoxicCells
Th2
MastCells
TGD
Tcells
DC
Effector_mCD8
MDSC
Th1
NK
ActivatedCD8
NHEJ
MemoryB
BER
ImmatureB
UR
CR
M/G1
A
CPF
G1/S
CS
iDC
G2
ActivatedCD4
G2/M
varname
APOBEC3A
APOBEC3B
APOBEC3C
APOBEC3F
APOBEC3G
APOBEC3H
GS_adaptive
GS_CC
GS_DDR
GS_innate

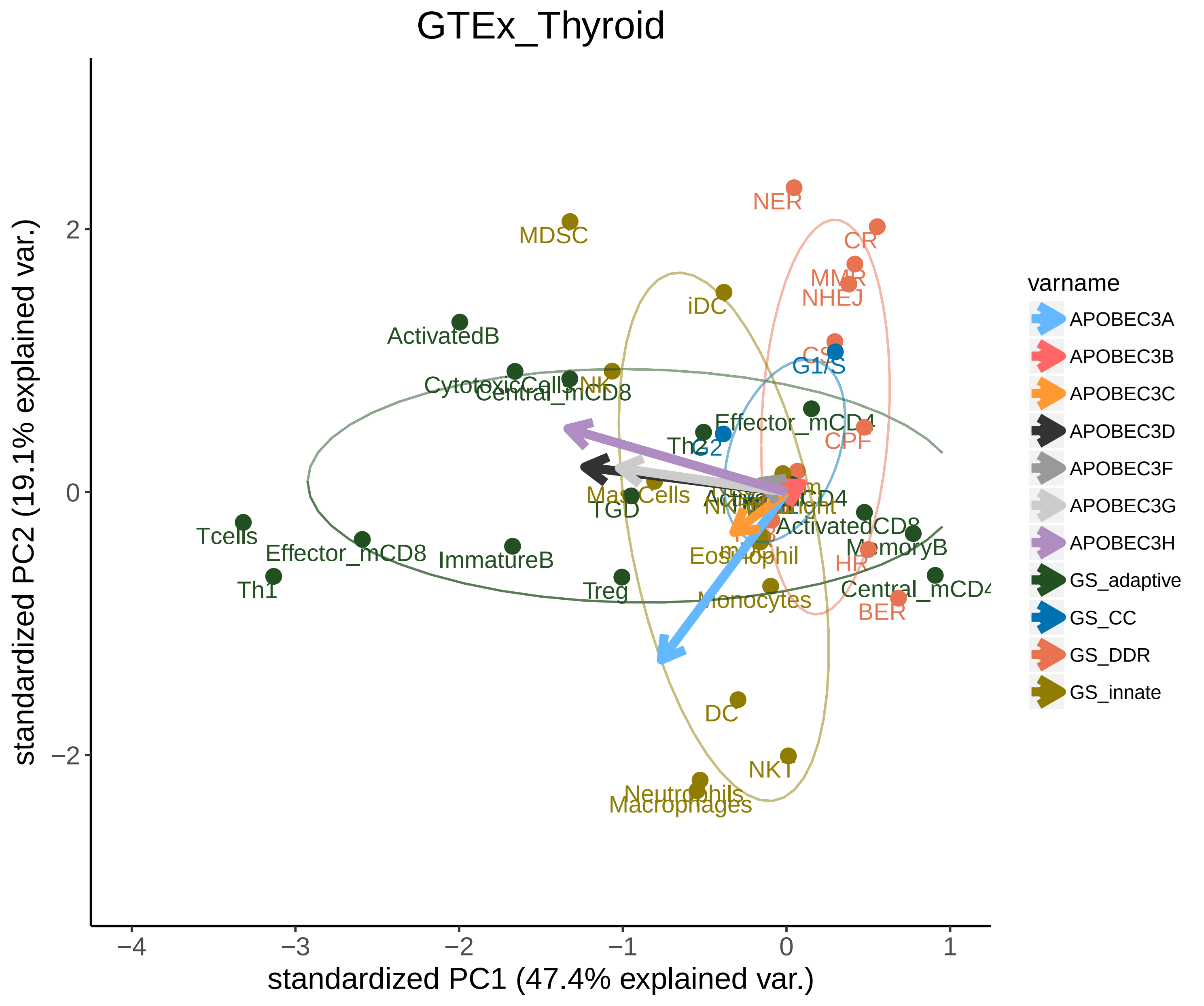
GTEx_Thyroid
standardized PC1 (47.4% explained var.)
standardized PC2 (19.1% explained var.)
varname
APOBEC3A
APOBEC3B
APOBEC3C
APOBEC3D
APOBEC3F
APOBEC3G
APOBEC3H
GS_adaptive
GS_CC
GS_DDR
GS_innate
NER
MDSC
CR
iDC
NHEJ
ActivatedB
G1/S
Central_mCD8
Effector_mCD4
CPF
Mast Cells
TGD
Tcells
Effector_mCD8
ImmatureB
Th1
Treg
ActivatedCD8
MemoryB
HR
Central_mCD4
BER
Monocytes
DC
NKT
Macrophages
−4
−3
−2
−1
0
1
2
0
−2

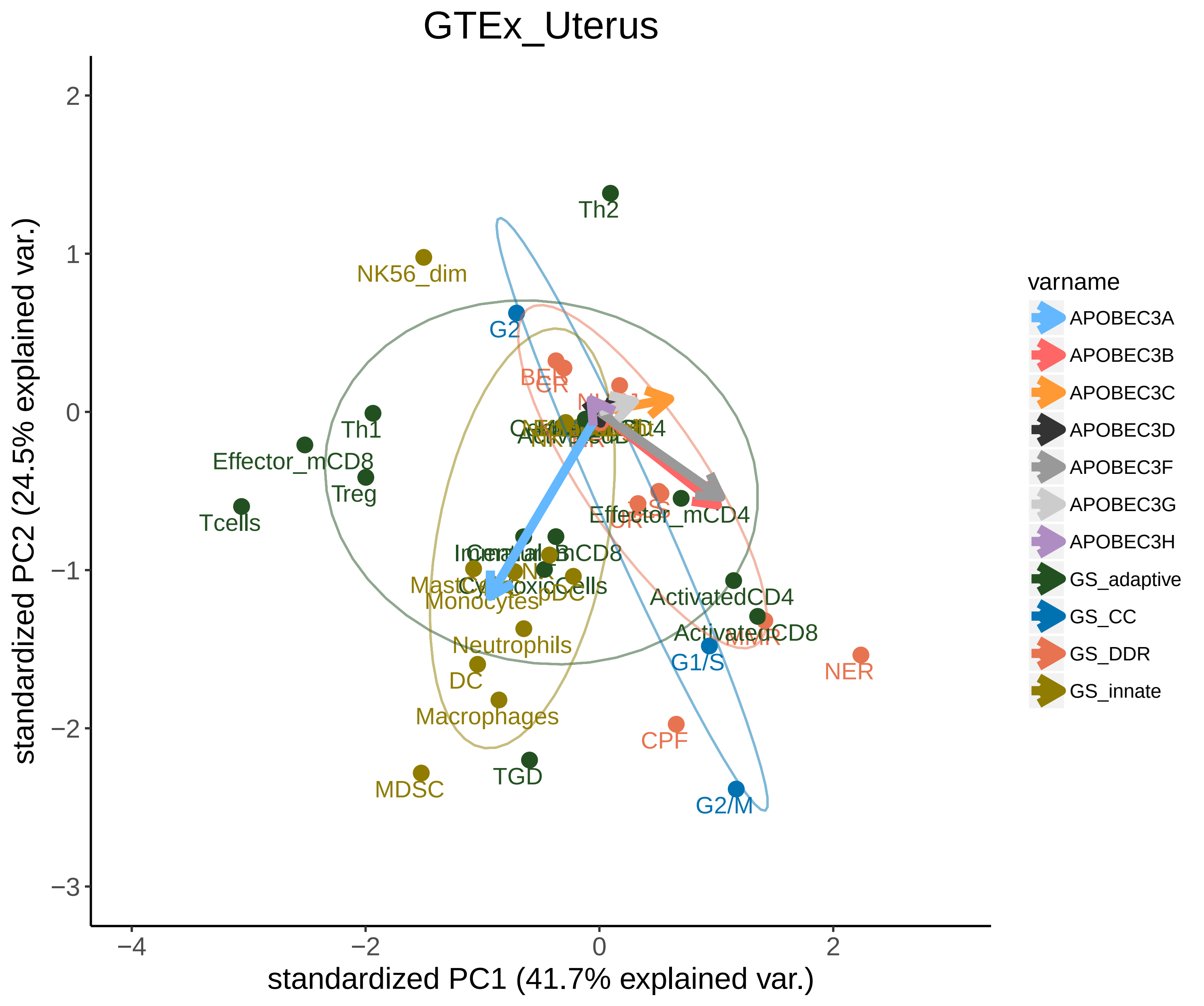
GTEx_Uterus
standardized PC1 (41.7% explained var.)
standardized PC2 (24.5% explained var.)
−4
−2
0
2
−3
−2
−1
0
1
2
varname
APOBEC3A
APOBEC3B
APOBEC3C
APOBEC3D
APOBEC3F
APOBEC3G
APOBEC3H
GS_adaptive
GS_CC
GS_DDR
GS_innate
Th2
NK56_dim
G2
BER
Th1
Effector_mCD8
Treg
Tcells
Effector_mCD4
ActivatedCD4
ActivatedCD8
G1/S
NER
Monocytes
pDC
Neutrophils
DC
Macrophages
CPF
MDSC
TGD
G2/M

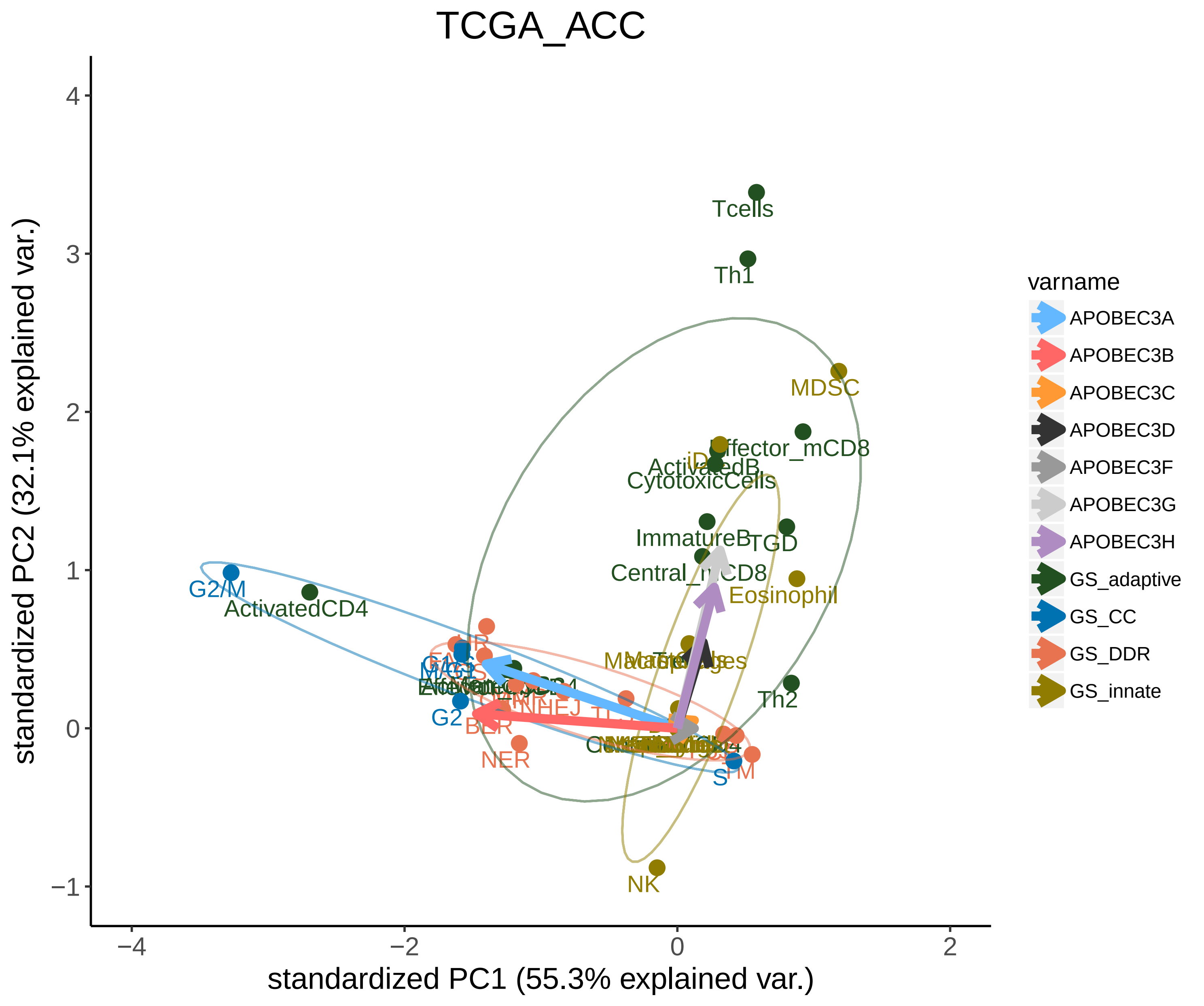

TCGA_ACC
standardized PC2 (32.1% explained var.)
standardized PC1 (55.3% explained var.)
4
3
2
1
0
−1
−4
−2
0
2
Tcells
Th1
MDSC
Effector_mCD8
ActivatedB
CytotoxicCells
ImmatureB
TGD
Central_mCD8
Eosinophil
G2/M
ActivatedCD4
Th2
G2
BER
NER
NHEJ
S
MMR
NK
varname
APOBEC3A
APOBEC3B
APOBEC3C
APOBEC3D
APOBEC3F
APOBEC3G
APOBEC3H
GS_adaptive
GS_CC
GS_DDR
GS_innate

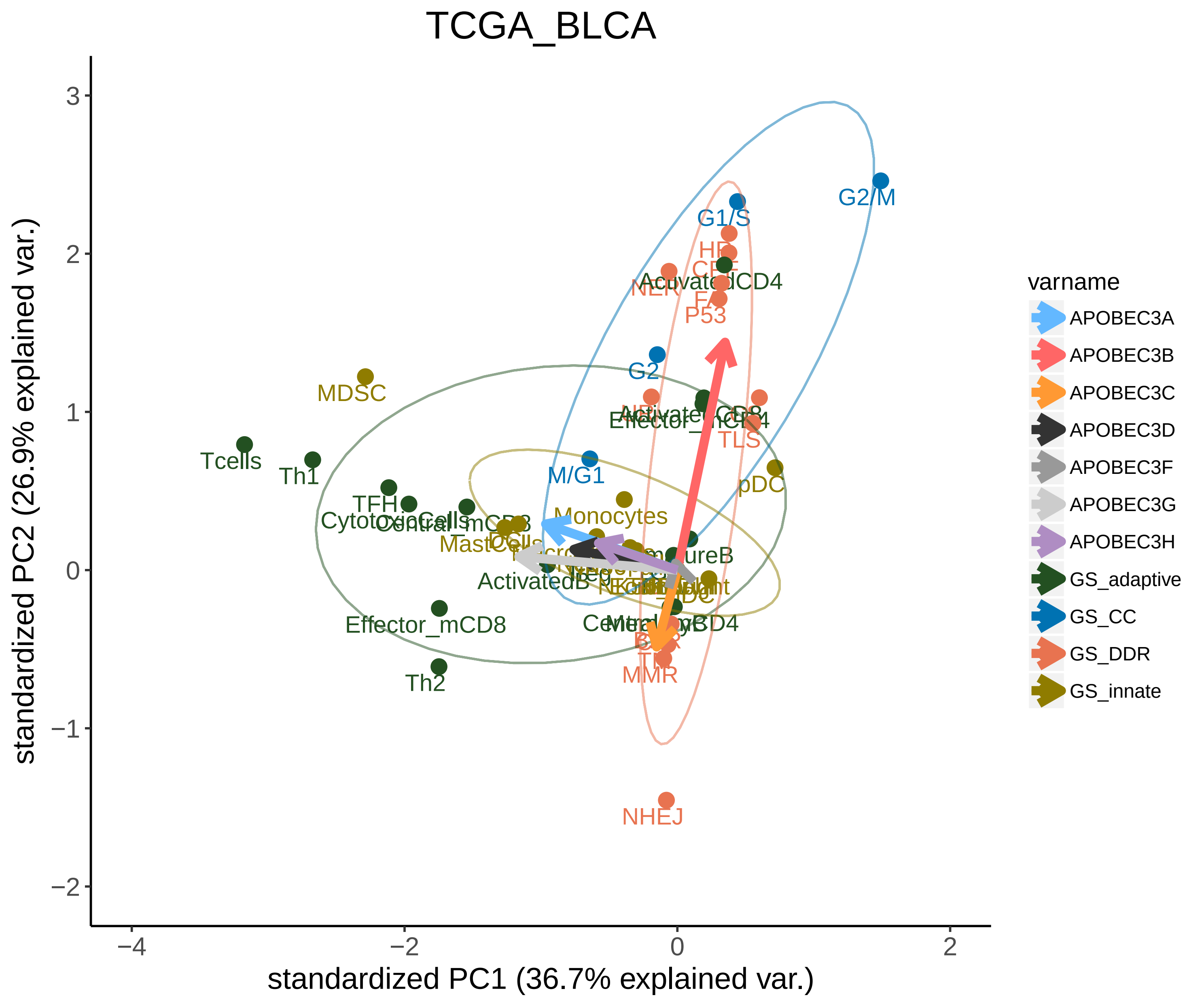
TCGA_BLCA
standardized PC2 (26.9% explained var.)
standardized PC1 (36.7% explained var.)
3
2
1
0
−1
−2
−4
−2
0
2
G2/M
G1/S
HR
NER
ActivatedCD4
P53
G2
MDSC
TLS
Effector_mCD4
Tcells
Th1
M/G1
pDC
TFH
Monocytes
MastCells
ActivatedB
ImmatureB
Effector_mCD8
MMR
Th2
NHEJ
varname
APOBEC3A
APOBEC3B
APOBEC3C
APOBEC3D
APOBEC3F
APOBEC3G
APOBEC3H
GS_adaptive
GS_CC
GS_DDR
GS_innate

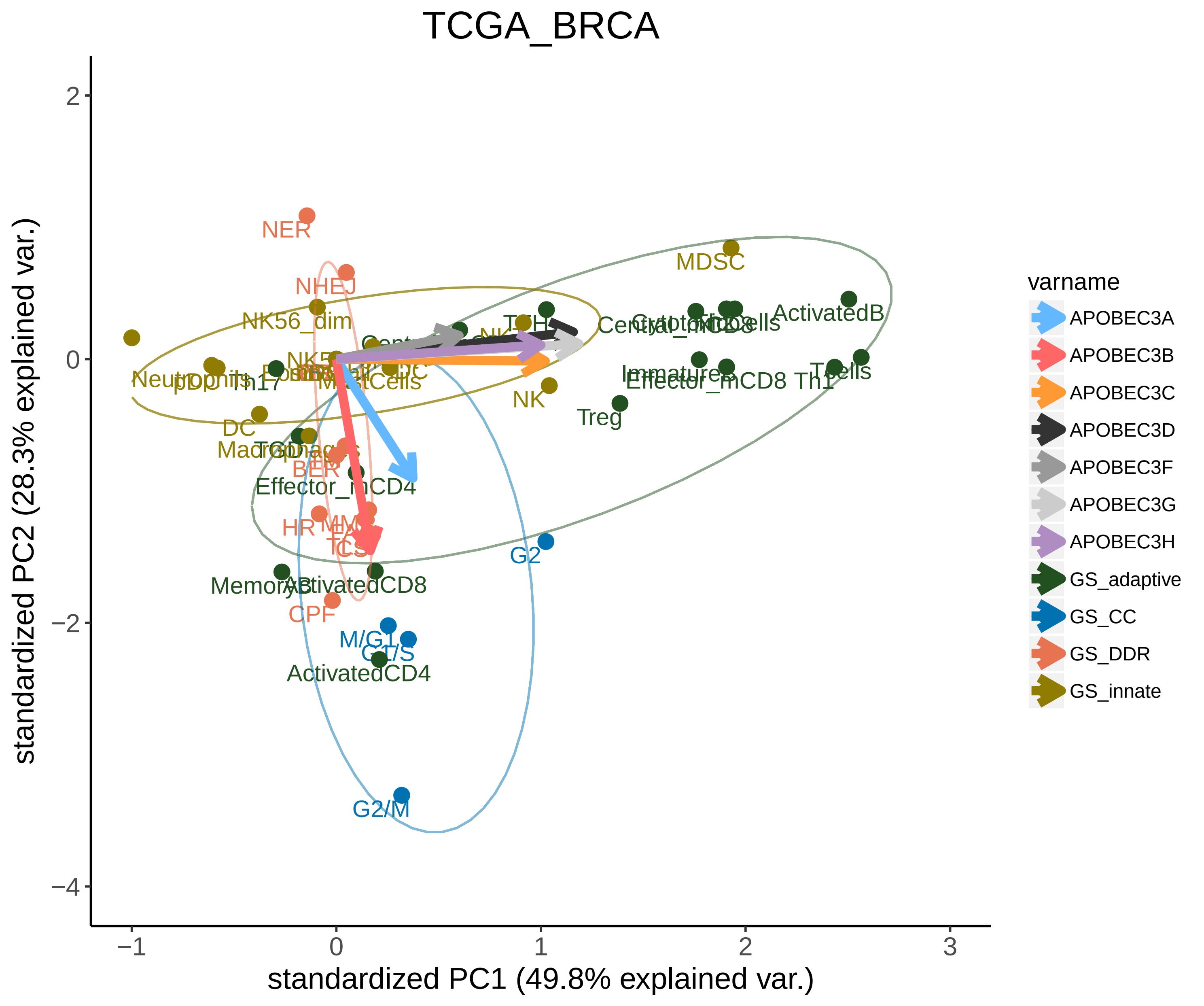

TCGA_BRCA
standardized PC2 (28.3% explained var.)
standardized PC1 (49.8% explained var.)
2
0
−2
−4
−1
0
1
2
3
NER
NHEJ
MDSC
ActivatedB
NK56_dim
TFH
Treg
NK
DC
BER
Effector_mCD4
HR
TCS
G2
MemoryB
ActivatedCD8
CPF
M/G1
G1/S
ActivatedCD4
G2/M
Th1
varname
APOBEC3A
APOBEC3B
APOBEC3C
APOBEC3D
APOBEC3F
APOBEC3G
APOBEC3H
GS_adaptive
GS_CC
GS_DDR
GS_innate

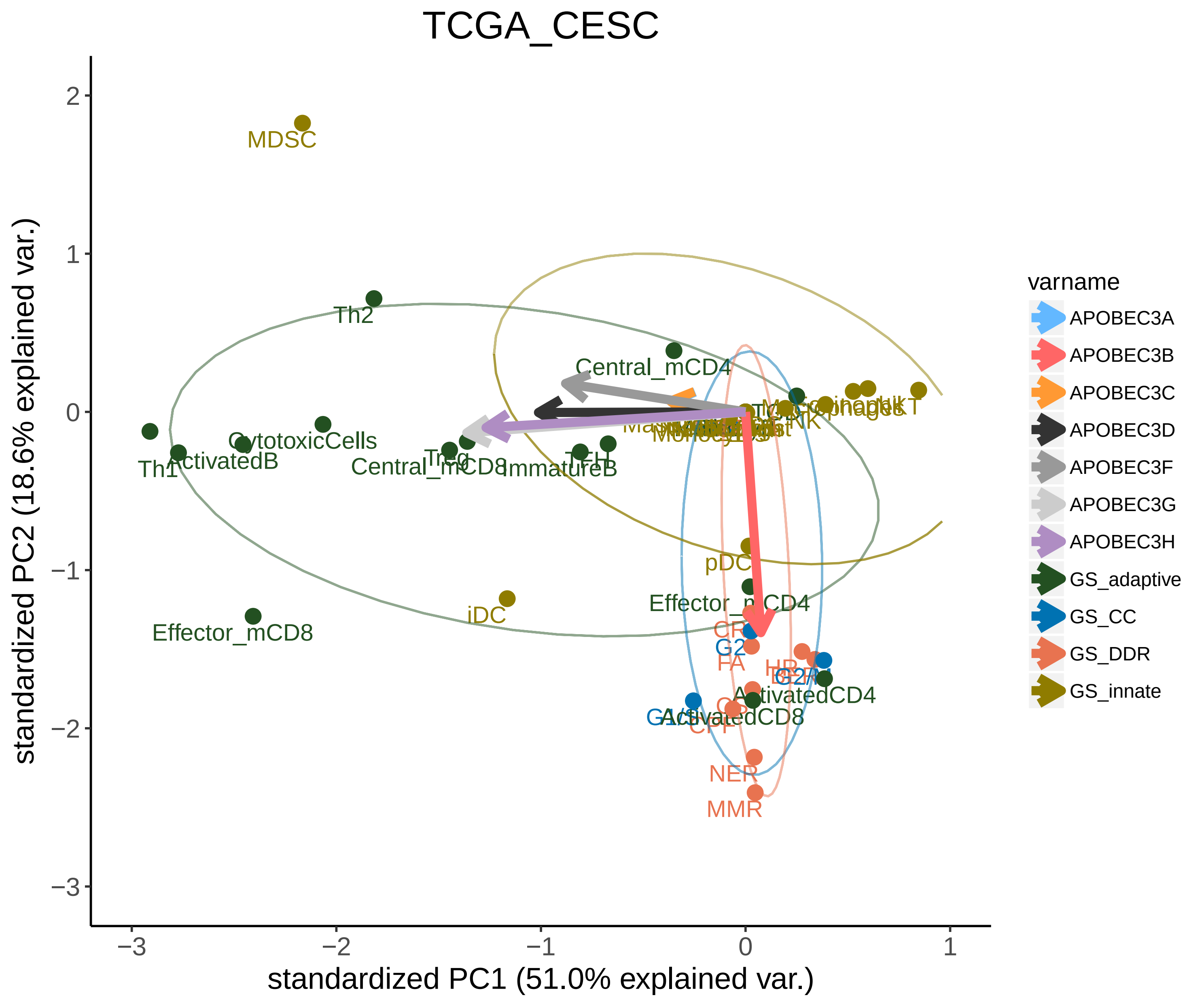
TCGA_CESC
standardized PC2 (18.6% explained var.)
standardized PC1 (51.0% explained var.)
2
1
0
−1
−2
−3
−3
−2
−1
0
1
MDSC
Th2
Central_mCD4
CytotoxicCells
Th1
ActivatedB
Central_mCD8
ImmatureB
Treg
TFH
pDC
Effector_mCD4
Effector_mCD8
iDC
CR
G2
FA
HR
G2M
ActivatedCD4
ActivatedCD8
CPF
NER
MMR
varname
APOBEC3A
APOBEC3B
APOBEC3C
APOBEC3D
APOBEC3F
APOBEC3G
APOBEC3H
GS_adaptive
GS_CC
GS_DDR
GS_innate

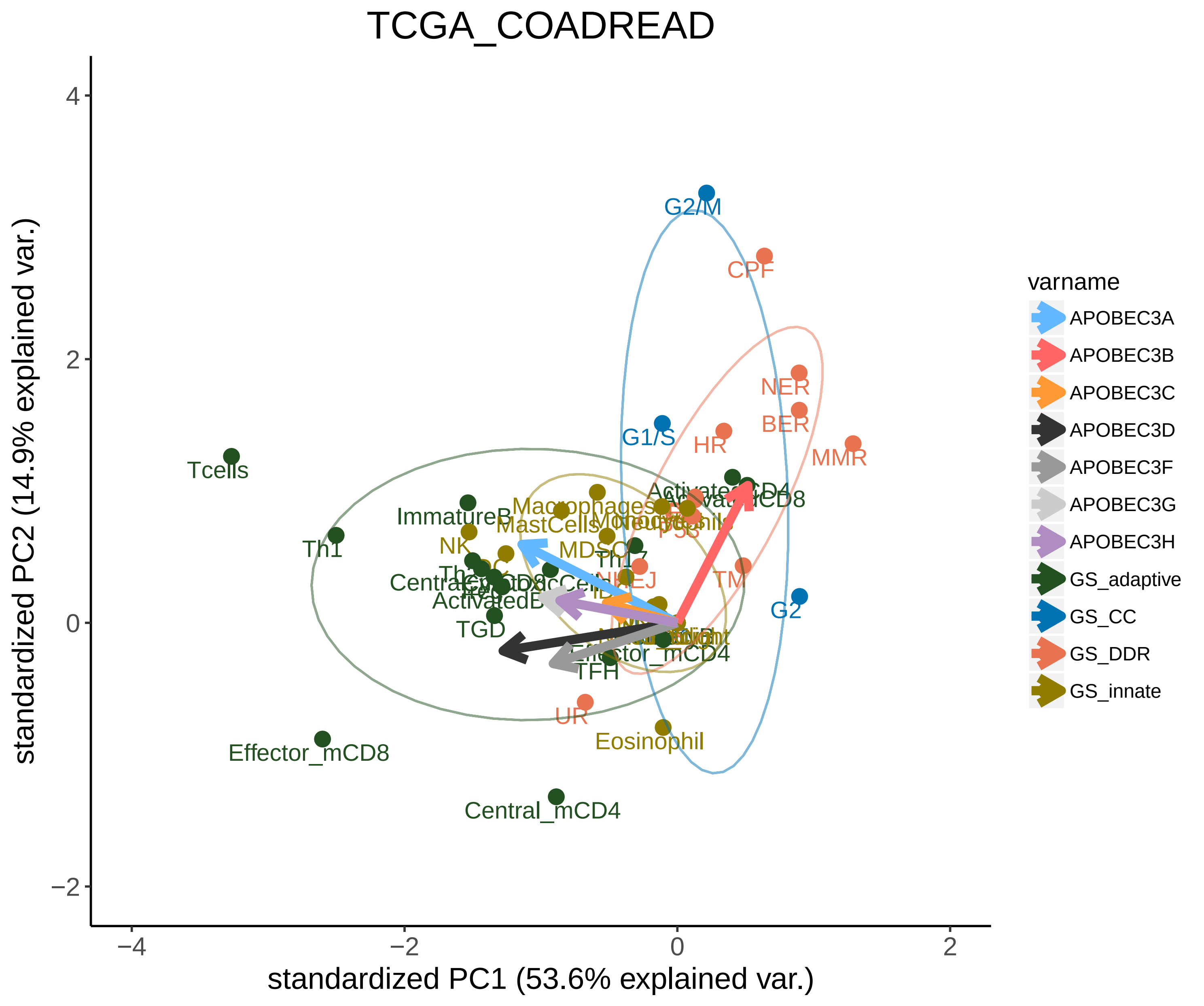
TCGA_COADREAD
standardized PC2 (14.9% explained var.)
standardized PC1 (53.6% explained var.)
4
2
0
−2
−4
−2
0
2
G2/M
CPF
NER
BER
MMR
HR
G1/S
G2
TM
UR
Eosinophil
Tcells
Th1
ImmatureB
NK
MastCells
Macrophages
ActivatedB
TGD
TFH
Effector_mCD4
Effector_mCD8
Central_mCD4
varname
APOBEC3A
APOBEC3B
APOBEC3C
APOBEC3D
APOBEC3F
APOBEC3G
APOBEC3H
GS_adaptive
GS_CC
GS_DDR
GS_innate

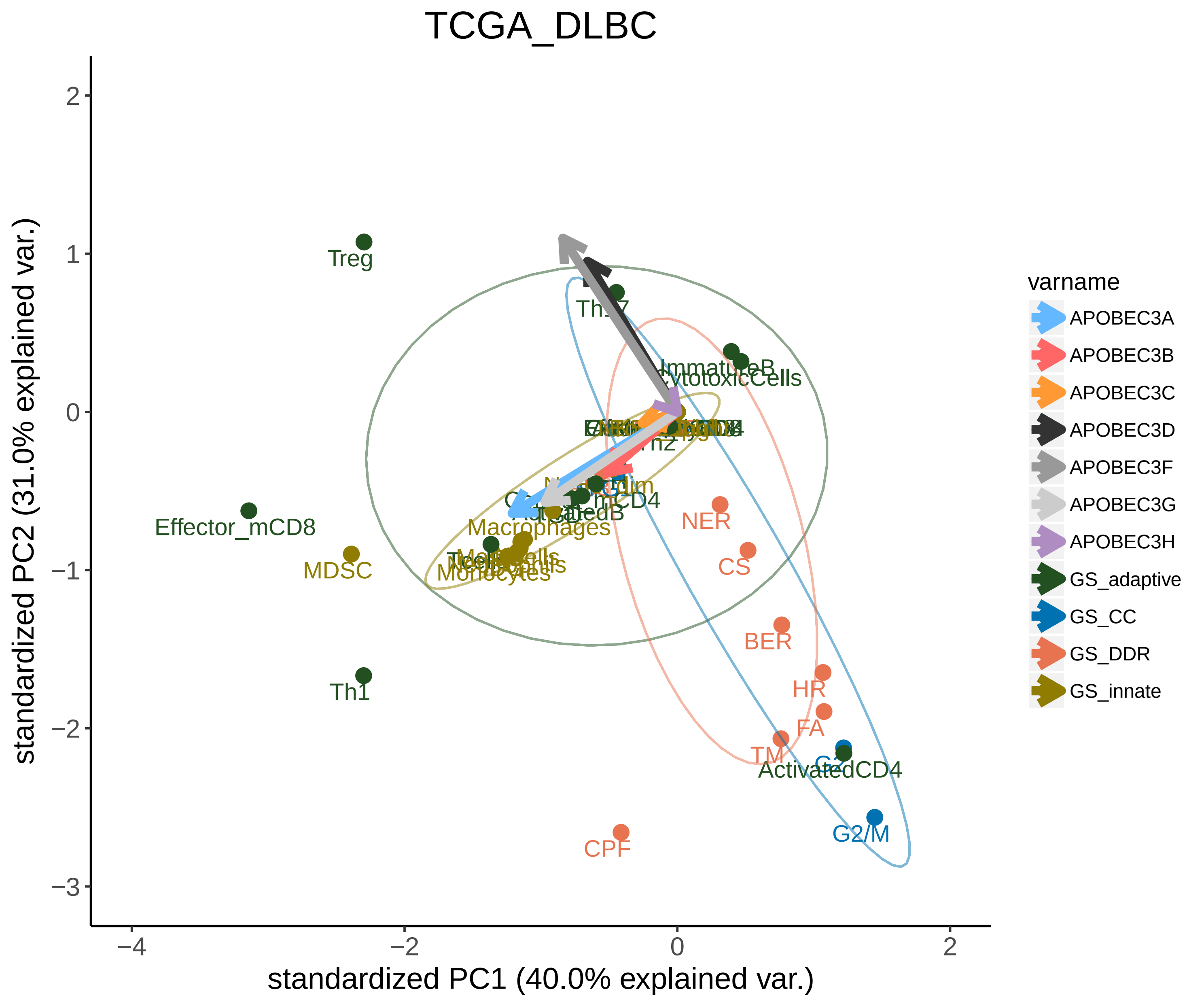
TCGA_DLBC
standardized PC2 (31.0% explained var.)
standardized PC1 (40.0% explained var.)
2
1
0
−1
−2
−3
−4
−2
0
2
Treg
ImmatureB
CytotoxicCells
Effector_mCD8
MDSC
Macrophages
Monocytes
NER
CS
BER
HR
FA
TM
ActivatedCD4
G2/M
CPF
Th1
varname
APOBEC3A
APOBEC3B
APOBEC3C
APOBEC3D
APOBEC3F
APOBEC3G
APOBEC3H
GS_adaptive
GS_CC
GS_DDR
GS_innate

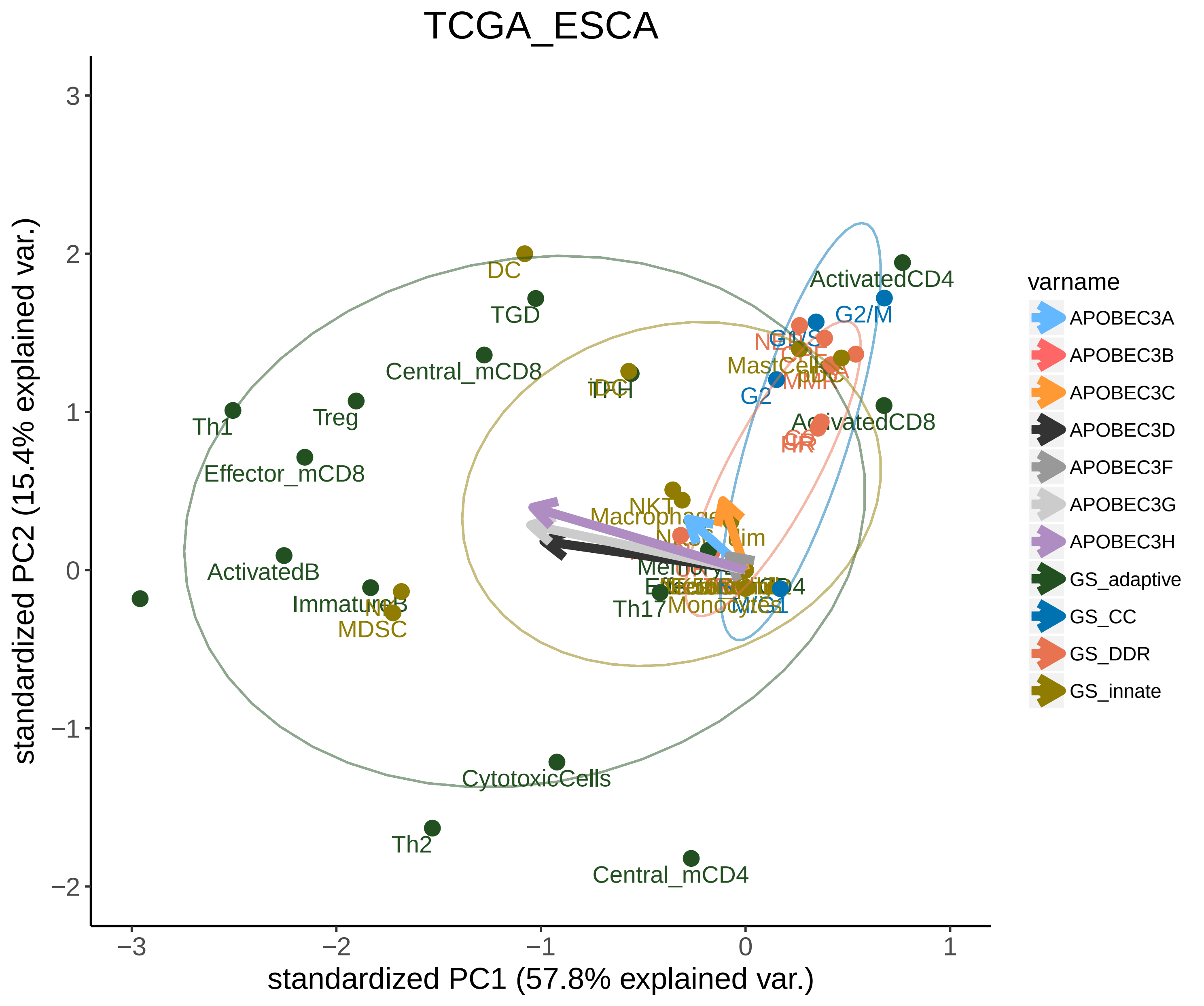

TCGA_ESCA
standardized PC1 (57.8% explained var.)
standardized PC2 (15.4% explained var.)
varname
APOBEC3A
APOBEC3B
APOBEC3C
APOBEC3D
APOBEC3F
APOBEC3G
APOBEC3H
GS_adaptive
GS_CC
GS_DDR
GS_innate
DC
TGD
Central_mCD8
Th1
Treg
Effector_mCD8
ActivatedB
MDSC
CytotoxicCells
Th2
Central_mCD4
ActivatedCD4
G2/M
G2
ActivatedCD8
MastCell
NKT
Th17

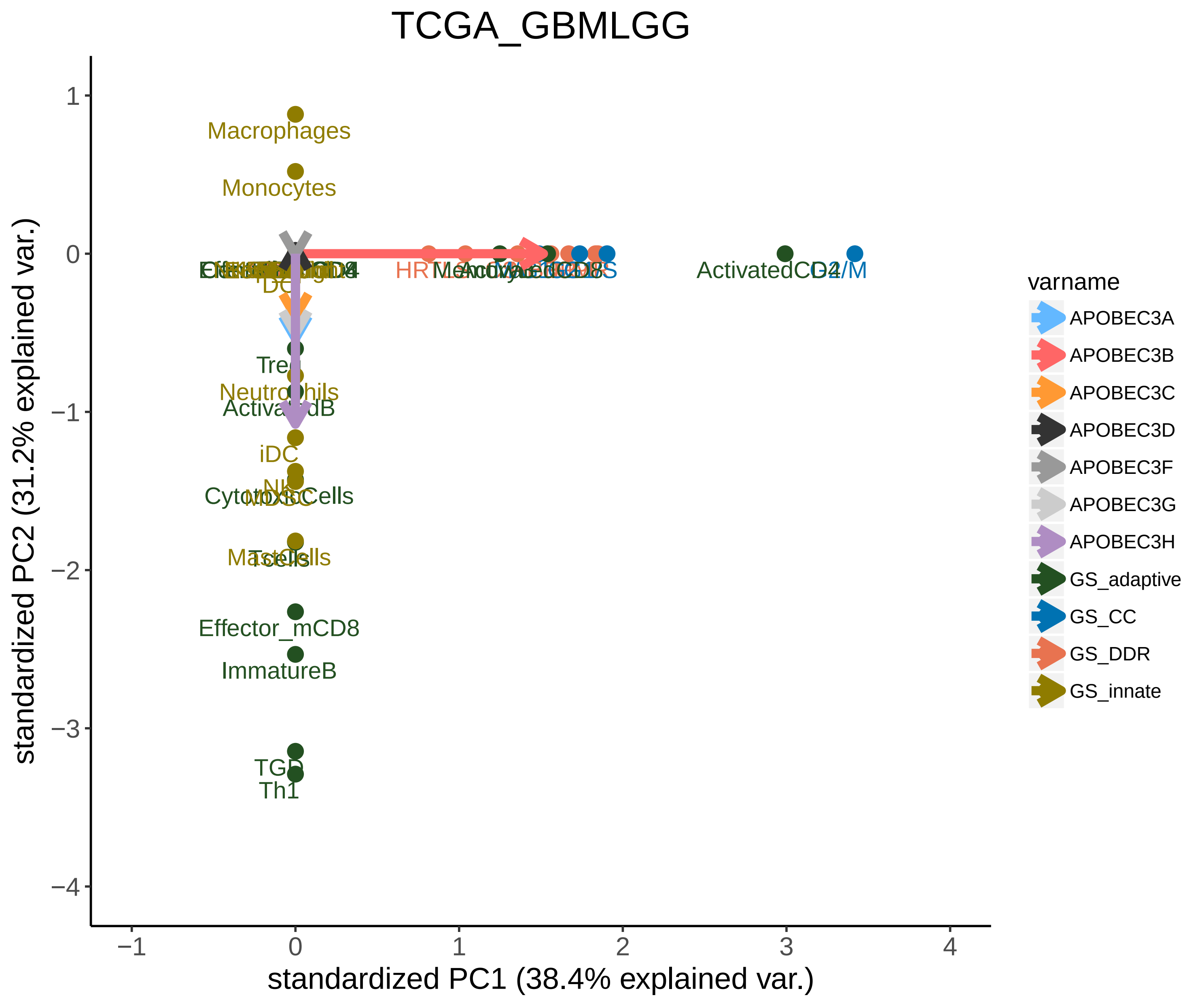
TCGA_GBMLGG
standardized PC2 (31.2% explained var.)
standardized PC1 (38.4% explained var.)
1
0
−1
−2
−3
−4
−1
0
1
2
3
4
Macrophages
Monocytes
DC
Treg
Neutrophils
iDC
Effector_mCD8
ImmatureB
TGD
Th1
ActivatedCD4
G2/M
varname
APOBEC3A
APOBEC3B
APOBEC3C
APOBEC3D
APOBEC3F
APOBEC3G
APOBEC3H
GS_adaptive
GS_CC
GS_DDR
GS_innate

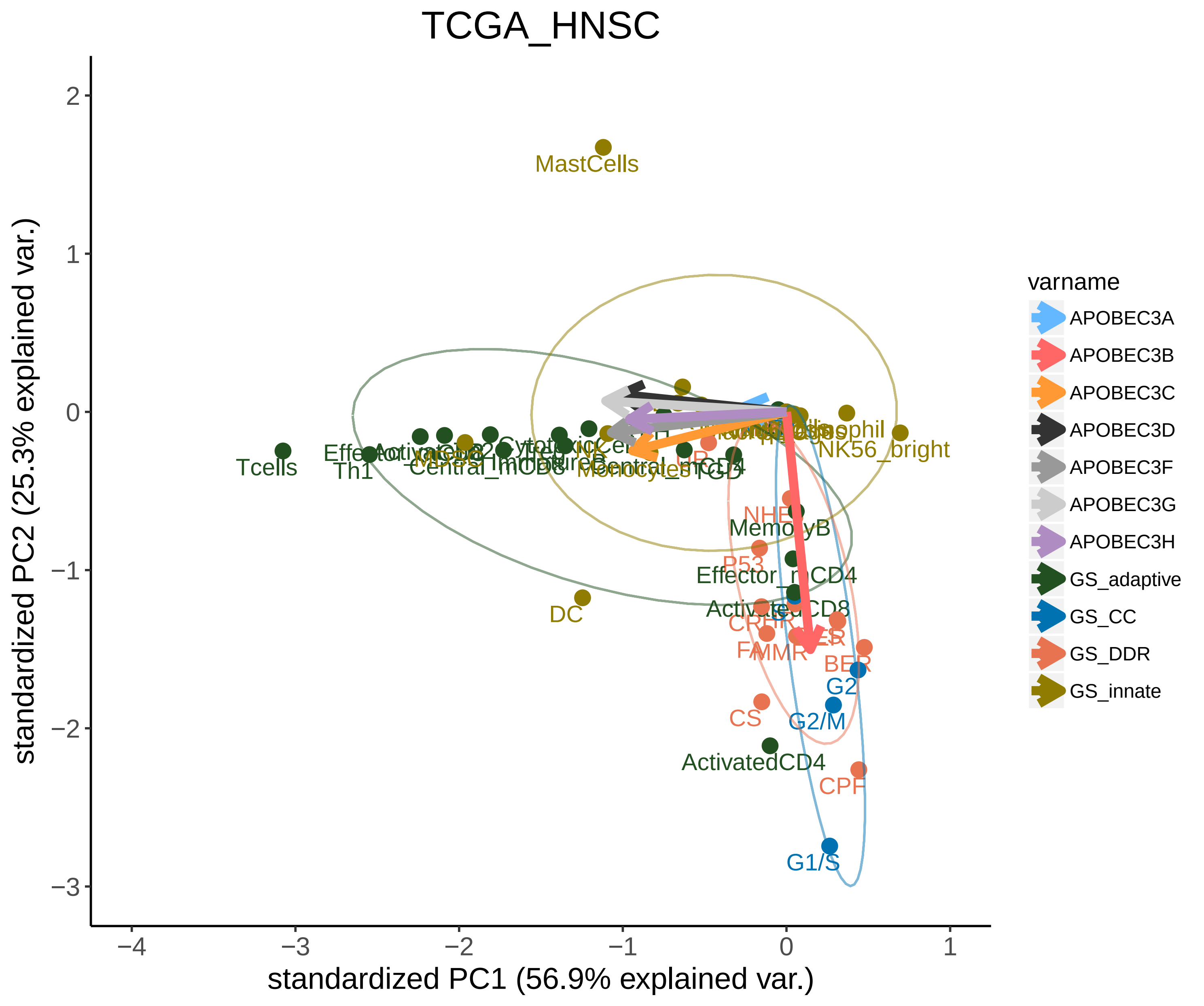
TCGA_HNSC
standardized PC2 (25.3% explained var.)
standardized PC1 (56.9% explained var.)
2
1
0
−1
−2
−3
−4
−3
−2
−1
0
1
MastCells
Tcells
Th1
NK56_bright
MemoryB
Effector_mCD4
DC
ActivatedCD4
CS
G2/M
G2
BER
CPF
G1/S
P53
varname
APOBEC3A
APOBEC3B
APOBEC3C
APOBEC3D
APOBEC3F
APOBEC3G
APOBEC3H
GS_adaptive
GS_CC
GS_DDR
GS_innate

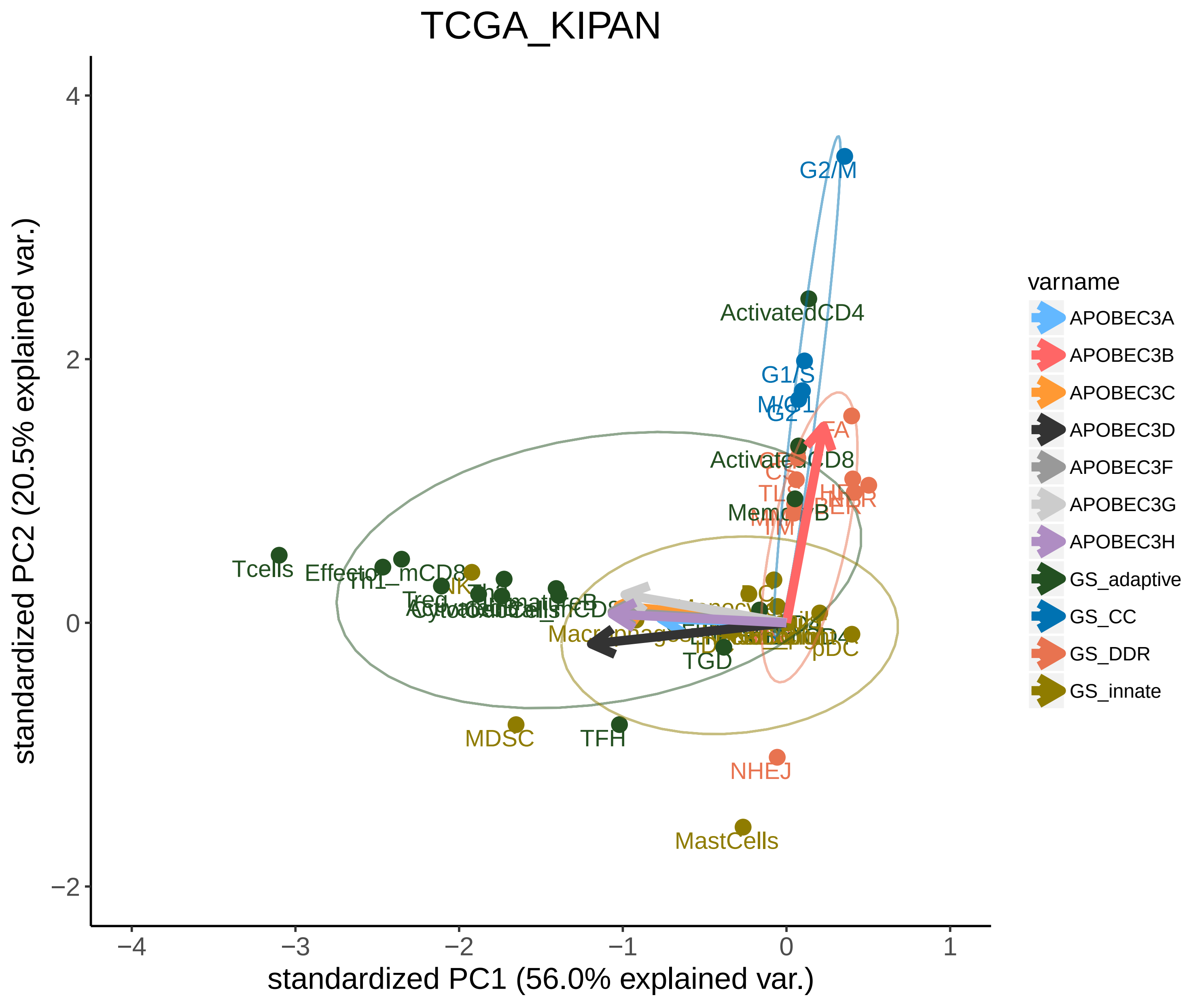
TCGA_KIPAN
standardized PC1 (56.0% explained var.)
standardized PC2 (20.5% explained var.)
−4
−3
−2
−1
0
1
4
2
0
−2
G2/M
ActivatedCD4
G1/S
ActivatedCD8
MemoryB
Tcells
MDSC
TFH
NHEJ
MastCells
TGD
pDC
varname
APOBEC3A
APOBEC3B
APOBEC3C
APOBEC3D
APOBEC3F
APOBEC3G
APOBEC3H
GS_adaptive
GS_CC
GS_DDR
GS_innate

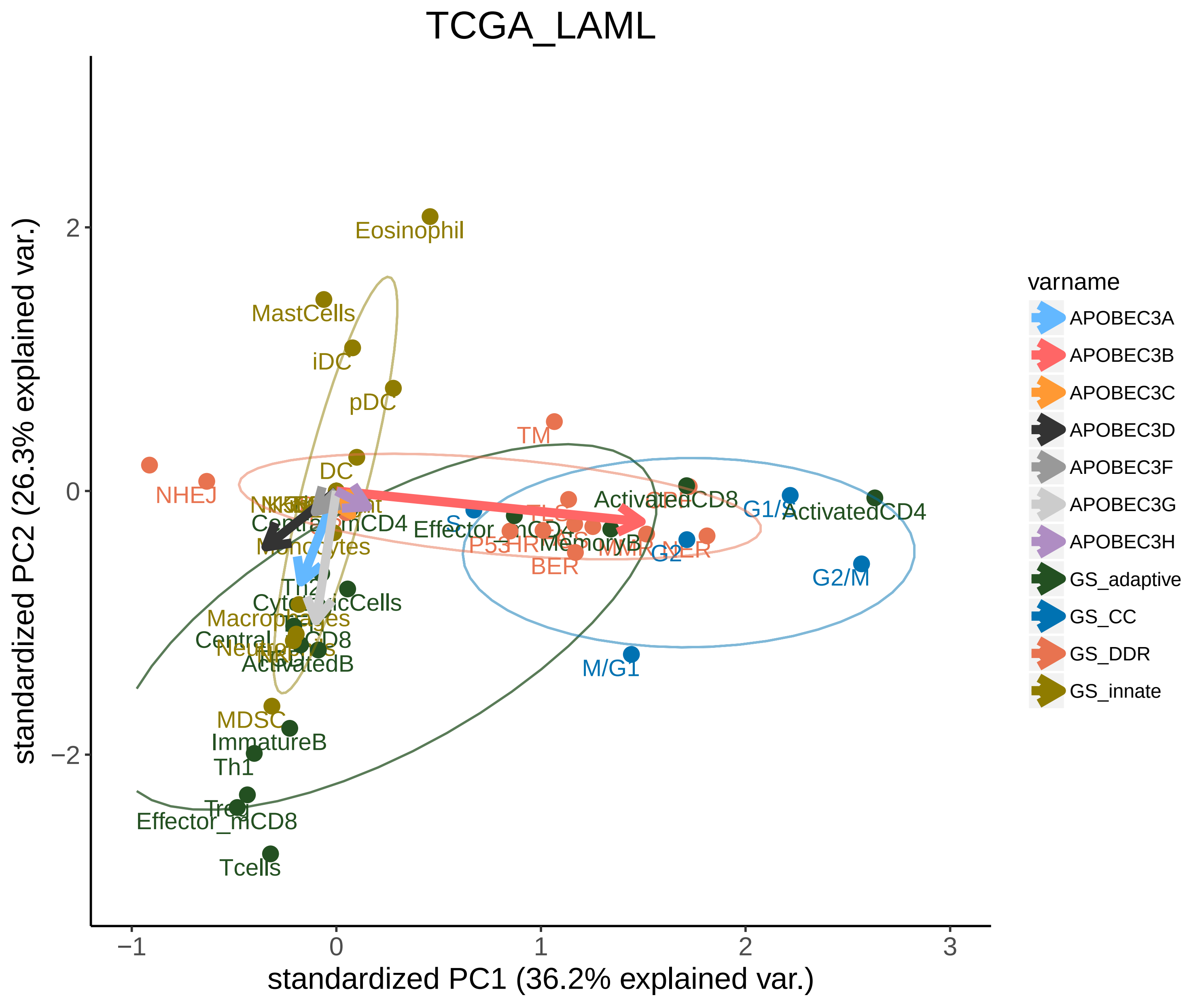
TCGA_LAML
standardized PC1 (36.2% explained var.)
standardized PC2 (26.3% explained var.)
varname
APOBEC3A
APOBEC3B
APOBEC3C
APOBEC3D
APOBEC3F
APOBEC3G
APOBEC3H
GS_adaptive
GS_CC
GS_DDR
GS_innate
Eosinophil
MastCells
iDC
pDC
TM
DC
NHEJ
ActivatedCD8
ActivatedCD4
G1/S
G2/M
M/G1
BER
MDSC
ImmatureB
Th1
Effector_mCD8
Tcells
ActivatedB
−1
0
1
2
3
−2

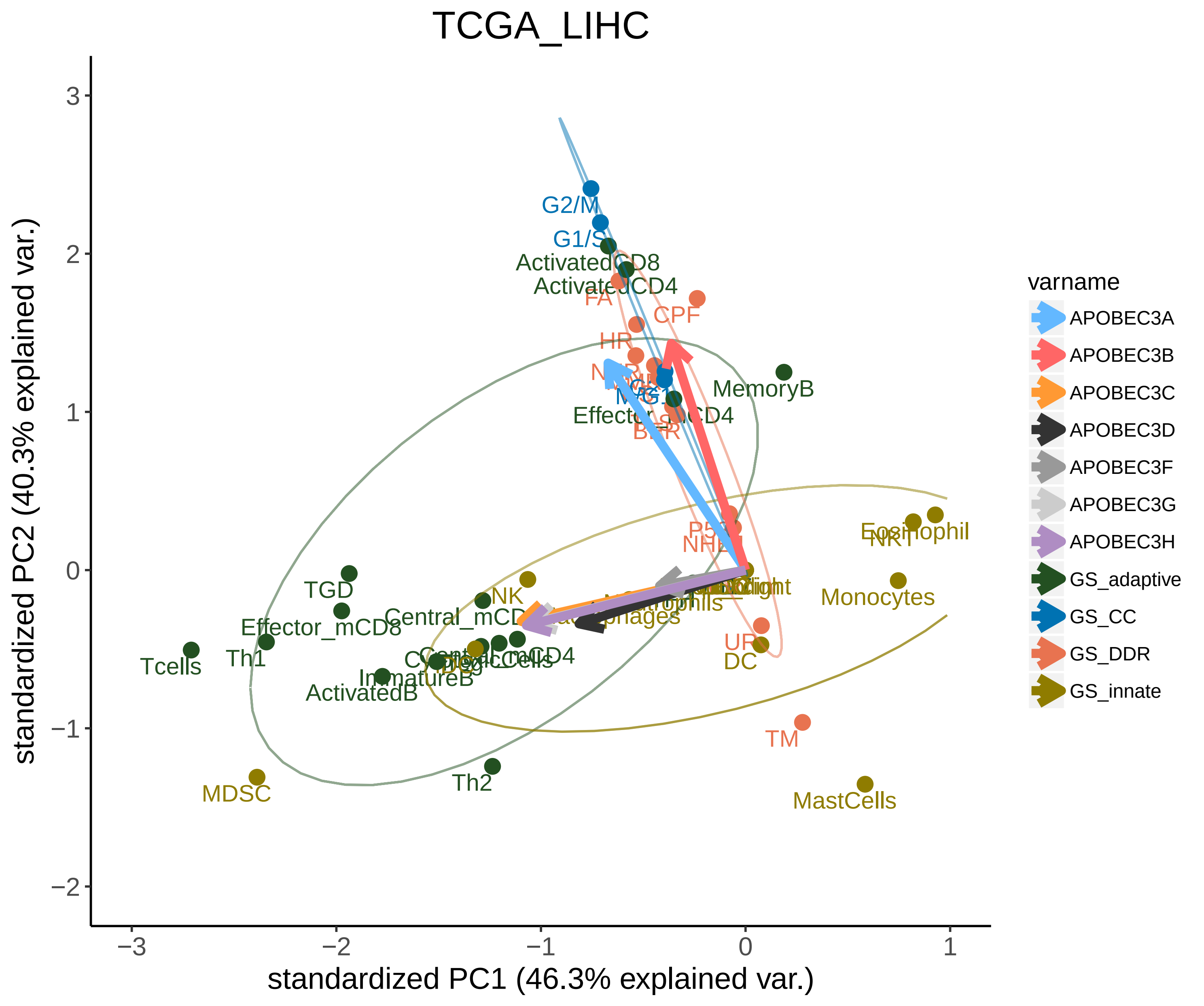

TCGA_LIHC
standardized PC1 (46.3% explained var.)
standardized PC2 (40.3% explained var.)
varname
APOBEC3A
APOBEC3B
APOBEC3C
APOBEC3D
APOBEC3F
APOBEC3G
APOBEC3H
GS_adaptive
GS_CC
GS_DDR
GS_innate
G2/M
G1/S
ActivatedCD8
ActivatedCD4
FA
CPF
HR
MemoryB
TGD
NK
Effector_mCD8
Tcells
Th1
ActivatedB
Th2
MDSC
TM
MastCells
Monocytes
DC
Eosinophil
NKT

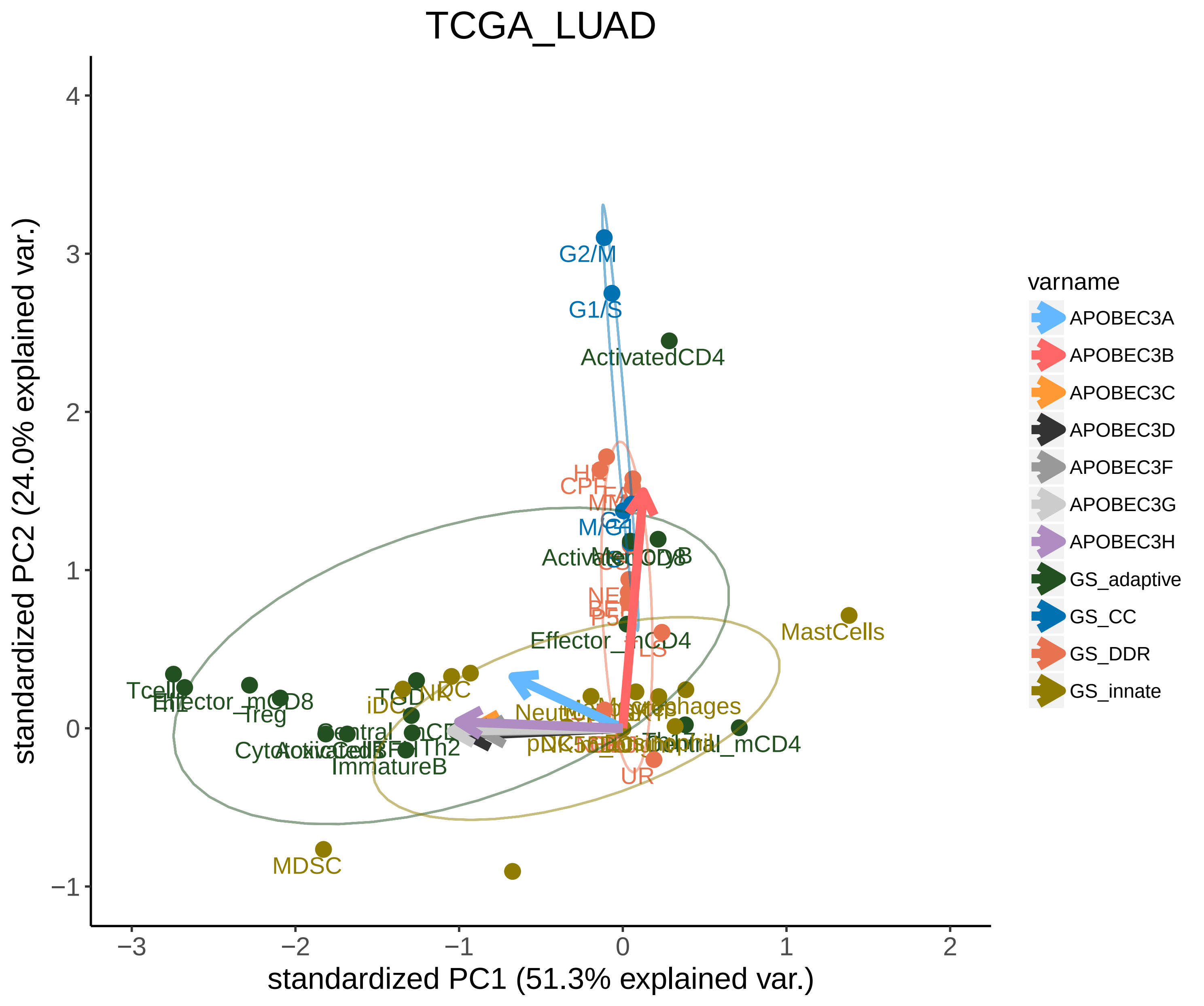
TCGA_LUAD
standardized PC2 (24.0% explained var.)
standardized PC1 (51.3% explained var.)
4
3
2
1
0
−1
−3
−2
−1
0
1
2
varname
APOBEC3A
APOBEC3B
APOBEC3C
APOBEC3D
APOBEC3F
APOBEC3G
APOBEC3H
GS_adaptive
GS_CC
GS_DDR
GS_innate
G2/M
G1/S
ActivatedCD4
M/G1
Effector_mCD4
MastCells
MDSC
ImmatureB
Treg
UR
Neutrophils
Macrophages
iDC

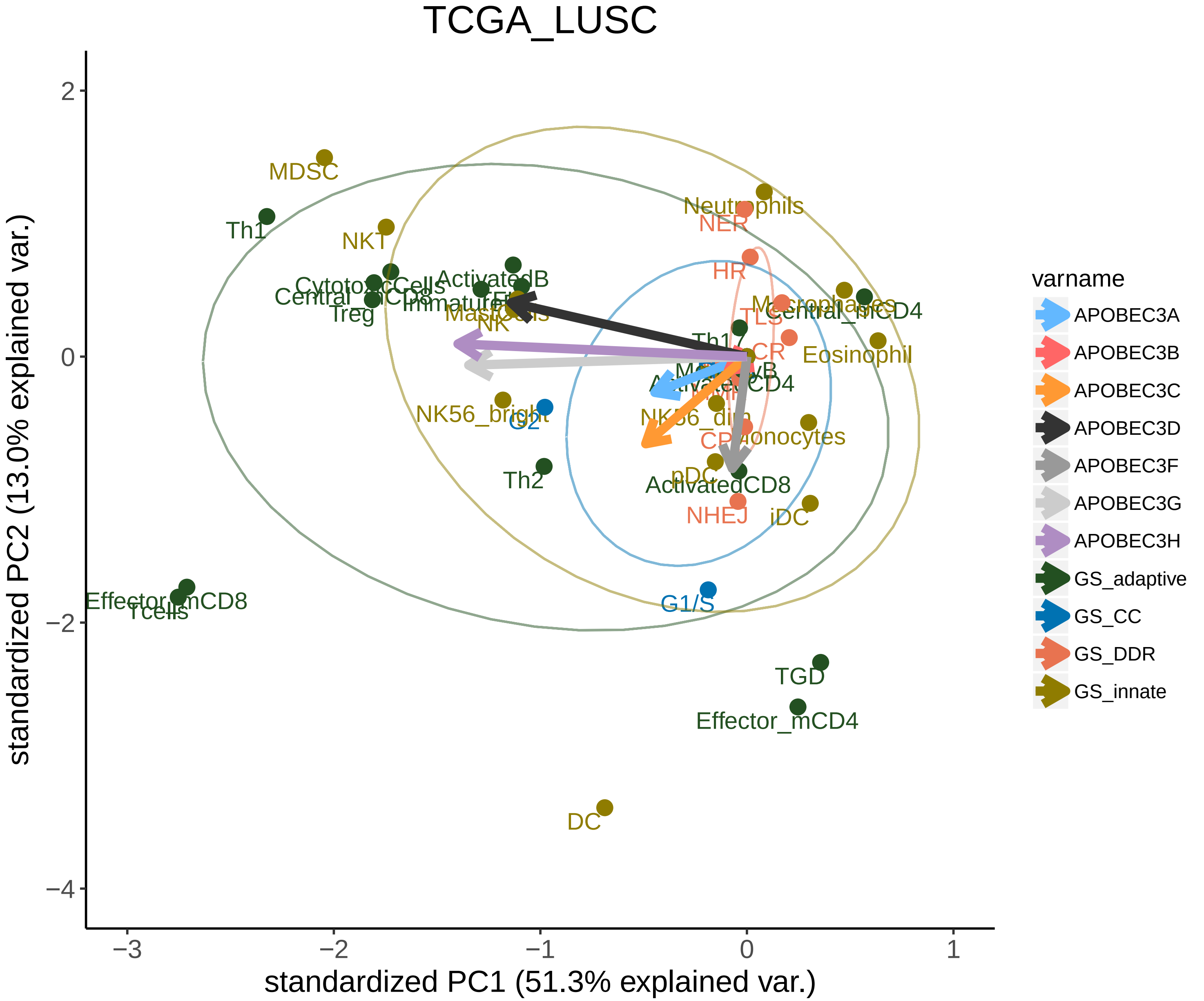

TCGA_LUSC
standardized PC2 (13.0% explained var.)
standardized PC1 (51.3% explained var.)
2
0
−2
−4
−3
−2
−1
0
1
MDSC
Th1
NKT
Neutrophils
NER
HR
ActivatedB
Central_mCD8
Treg
NK
Macrophages
Central_mCD4
TLS
Th17
CR
Eosinophil
NK56_bright
G2
NK56_dim
Monocytes
Th2
pDC
ActivatedCD8
NHEJ
iDC
Effector_mCD8
Tcells
G1/S
TGD
Effector_mCD4
DC
varname
APOBEC3A
APOBEC3B
APOBEC3C
APOBEC3D
APOBEC3F
APOBEC3G
APOBEC3H
GS_adaptive
GS_CC
GS_DDR
GS_innate

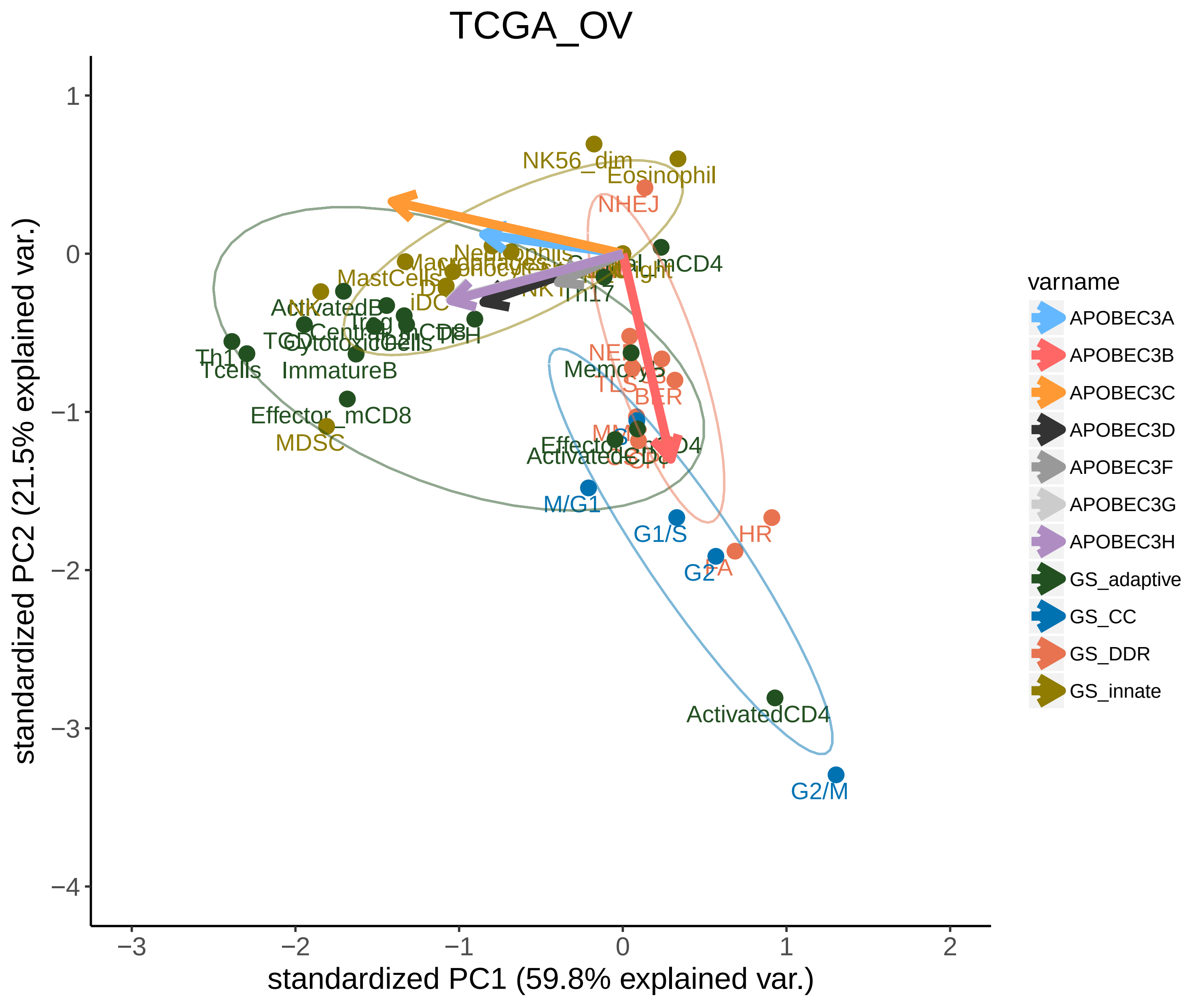
TCGA_OV
standardized PC1 (59.8% explained var.)
standardized PC2 (21.5% explained var.)
1
0
−1
−2
−3
−4
−3
−2
−1
0
1
2
varname
APOBEC3A
APOBEC3B
APOBEC3C
APOBEC3D
APOBEC3F
APOBEC3G
APOBEC3H
GS_adaptive
GS_CC
GS_DDR
GS_innate
NK56_dim
Eosinophil
NHEJ
MastCells
iDC
ActivatedB
Th17
ImmatureB
Th1
Tcells
Effector_mCD8
MDSC
Memory
BER
HR
M/G1
G1/S
G2
G2/M
ActivatedCD4
TFH

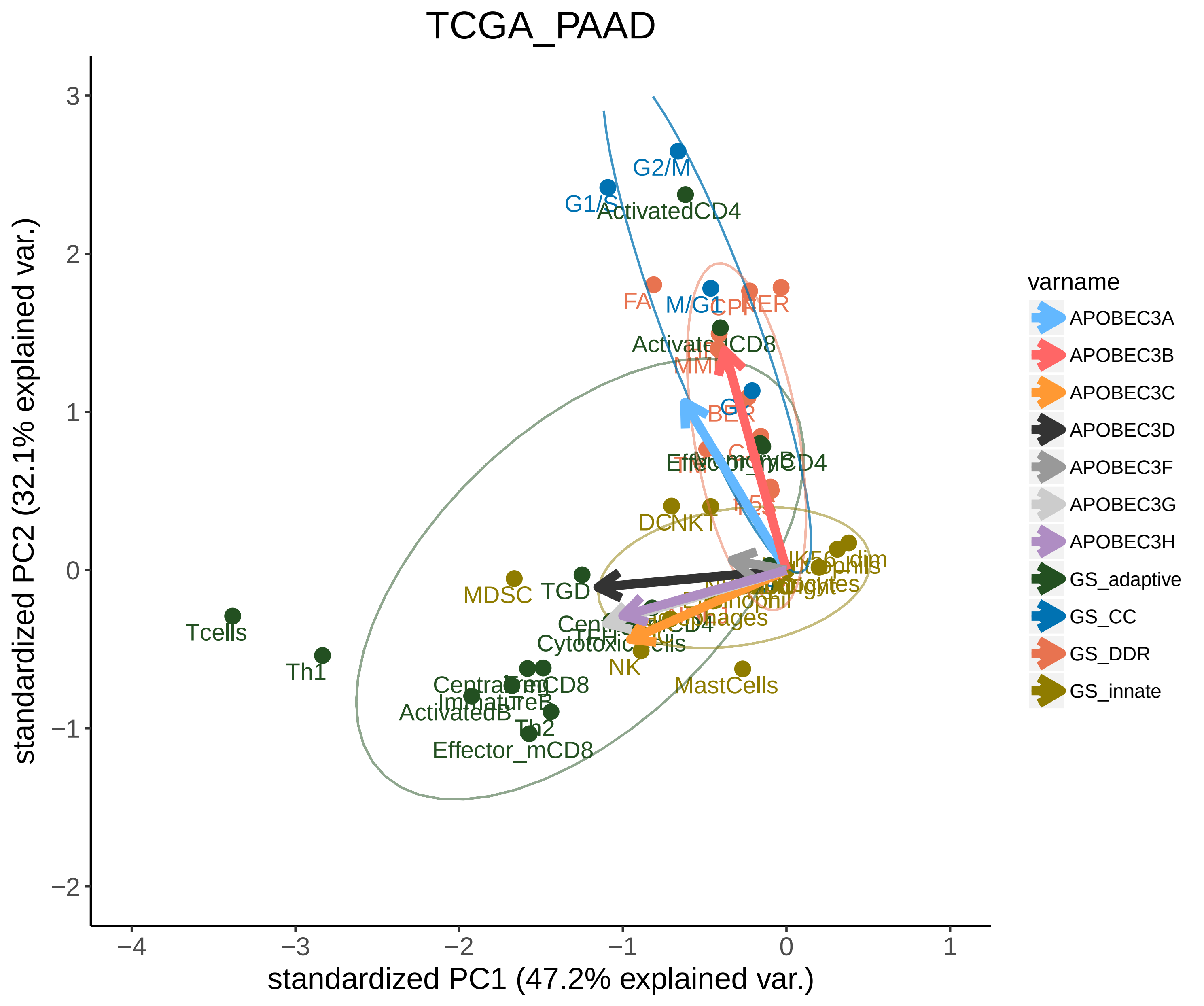
TCGA_PAAD
standardized PC2 (32.1% explained var.)
standardized PC1 (47.2% explained var.)
3
2
1
0
−1
−2
−4
−3
−2
−1
0
1
G2/M
G1/S
ActivatedCD4
FA
M/G1
MDSC
TGD
Tcells
Th1
NK
MastCells
ActivatedB
Th2
Effector_mCD8
varname
APOBEC3A
APOBEC3B
APOBEC3C
APOBEC3D
APOBEC3F
APOBEC3G
APOBEC3H
GS_adaptive
GS_CC
GS_DDR
GS_innate

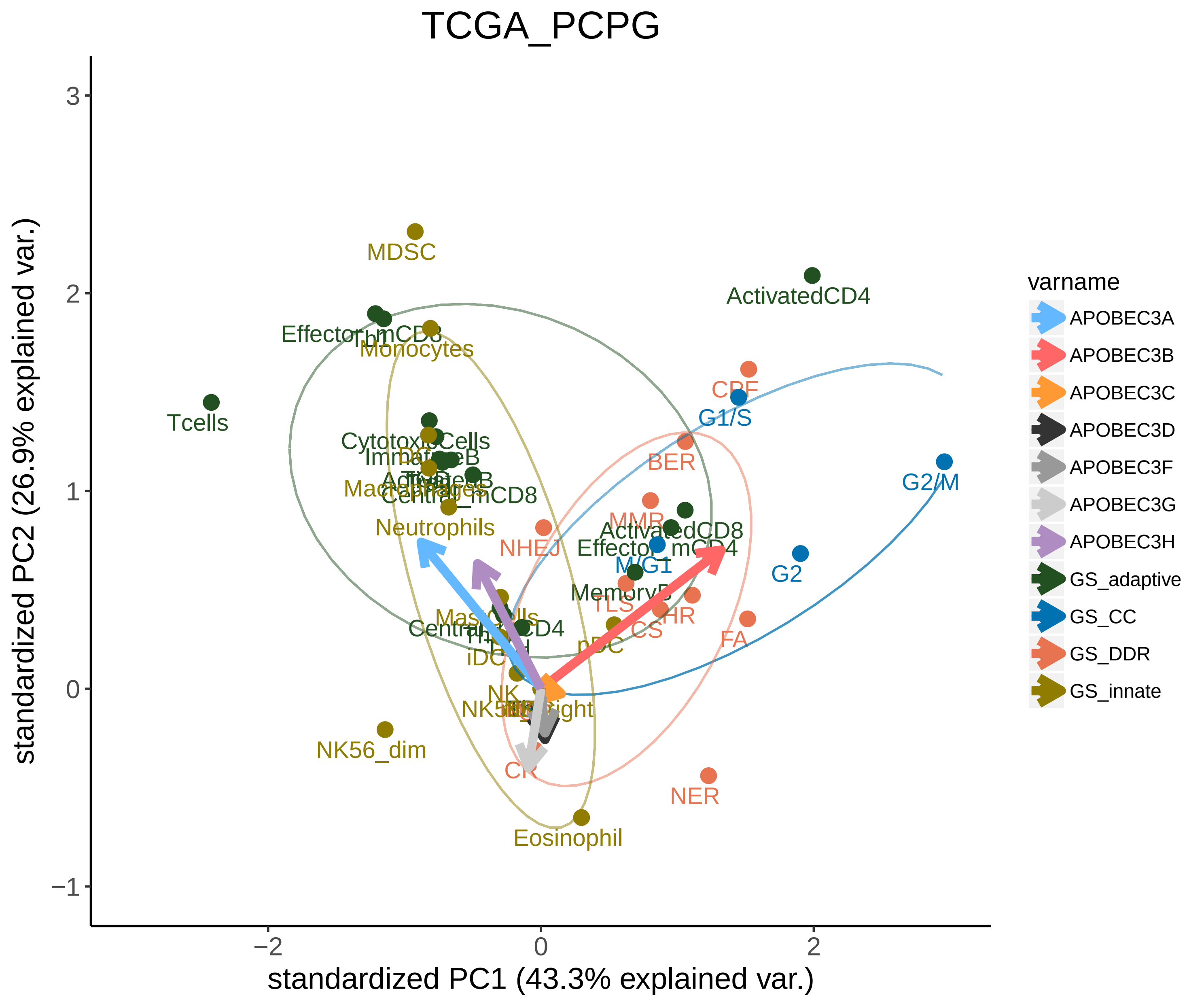
TCGA_PCPG
standardized PC2 (26.9% explained var.)
standardized PC1 (43.3% explained var.)
3
2
1
0
−1
−2
0
2
MDSC
ActivatedCD4
Effector_mCD8
Monocytes
Tcells
CPF
G1/S
BER
G2/M
Neutrophils
NHEJ
MMR
ActivatedCD8
M/G1
G2
TLS
HR
CS
FA
pDC
iDC
NK
NK56_dim
NER
Eosinophil
varname
APOBEC3A
APOBEC3B
APOBEC3C
APOBEC3D
APOBEC3F
APOBEC3G
APOBEC3H
GS_adaptive
GS_CC
GS_DDR
GS_innate

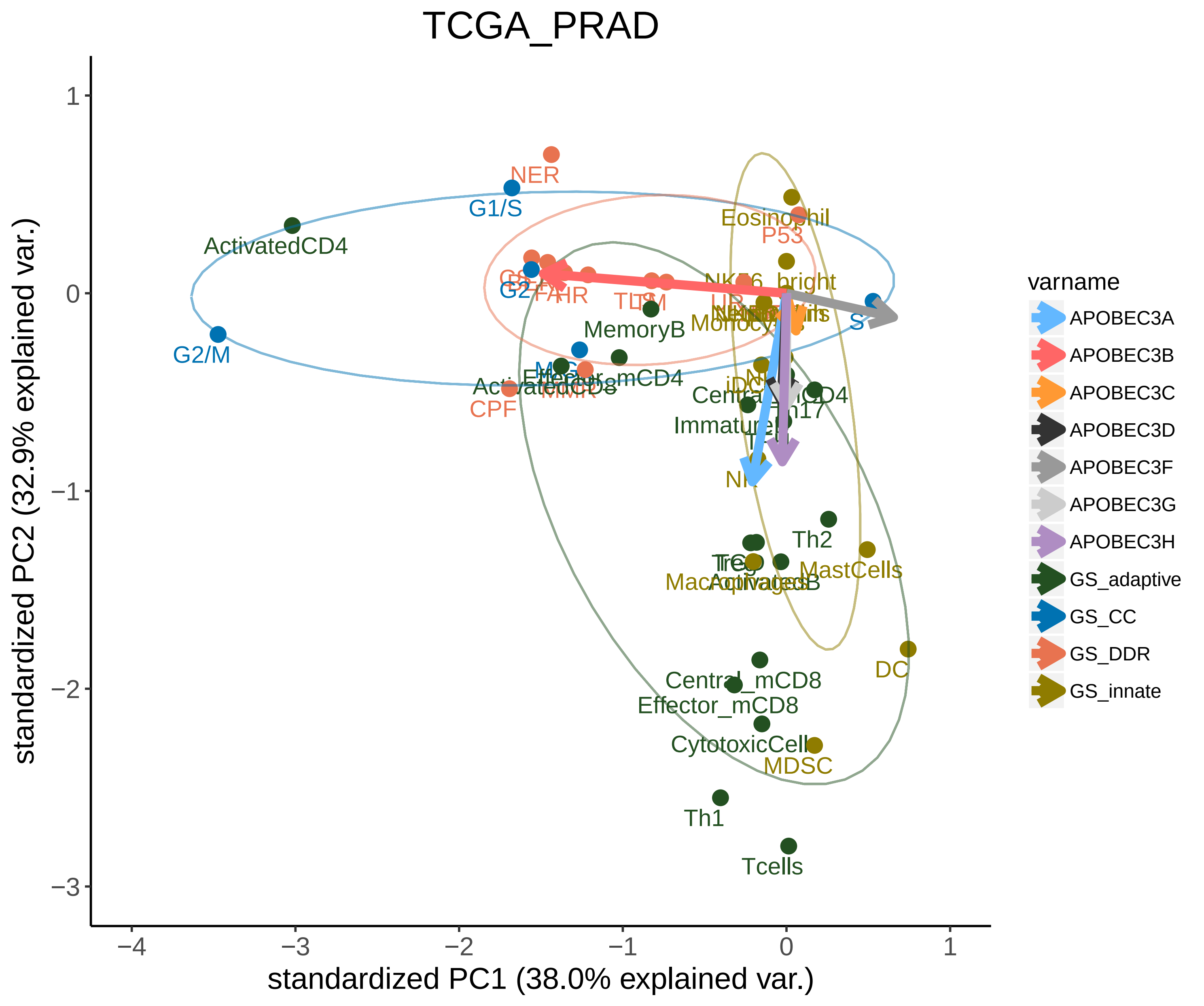

TCGA_PRAD
standardized PC1 (38.0% explained var.)
standardized PC2 (32.9% explained var.)
−4
−3
−2
−1
0
1
1
0
−1
−2
−3
varname
APOBEC3A
APOBEC3B
APOBEC3C
APOBEC3D
APOBEC3F
APOBEC3G
APOBEC3H
GS_adaptive
GS_CC
GS_DDR
GS_innate
NER
G1/S
ActivatedCD4
Eosinophil
P53
G2/M
MemoryB
Effector_mCD4
CPF
Th2
MastCells
DC
Central_mCD8
Effector_mCD8
CytotoxicCell
MDSC
Th1
Tcells

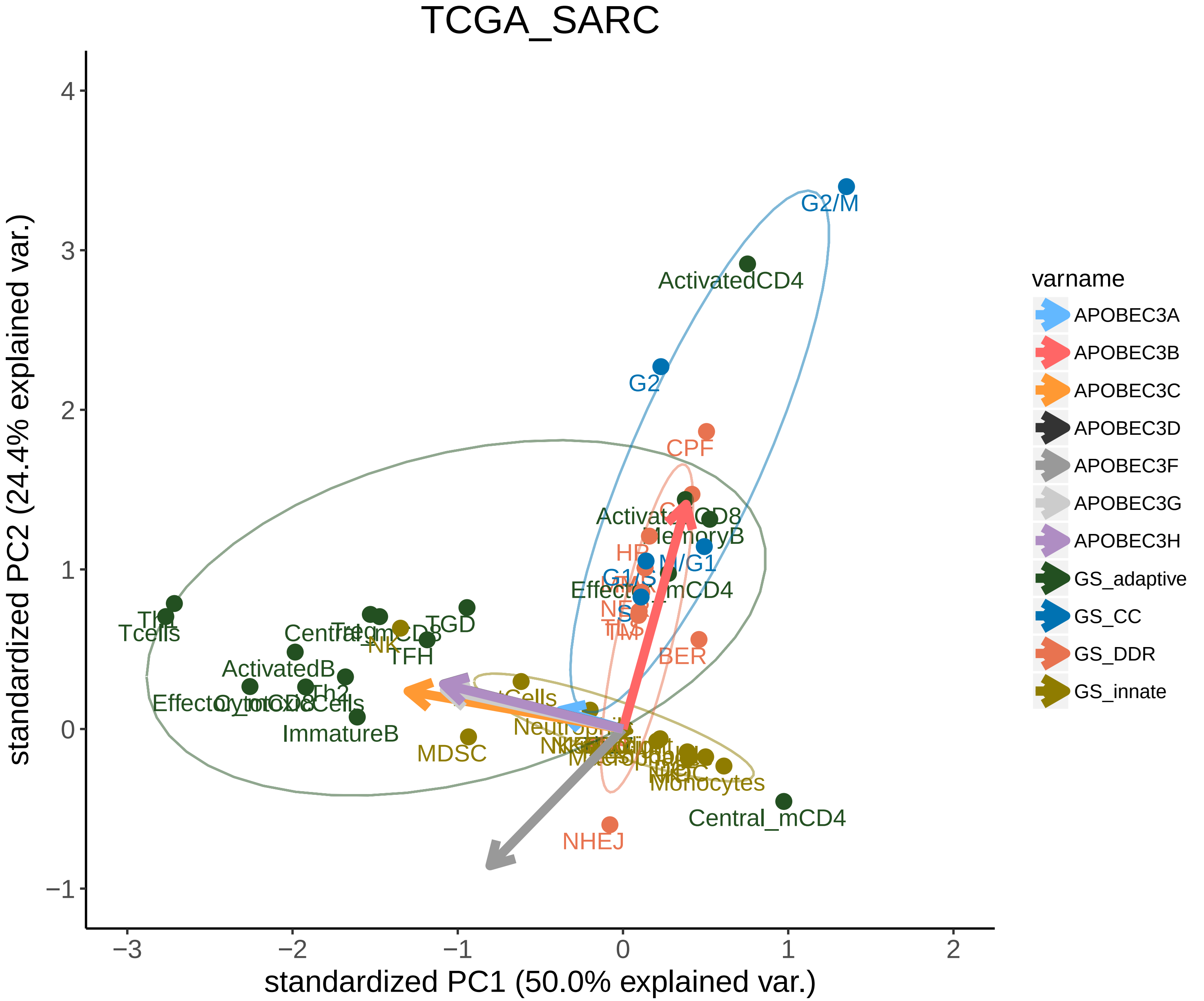

TCGA_SARC
standardized PC2 (24.4% explained var.)
standardized PC1 (50.0% explained var.)
4
3
2
1
0
−1
−3
−2
−1
0
1
2
varname
APOBEC3A
APOBEC3B
APOBEC3C
APOBEC3D
APOBEC3F
APOBEC3G
APOBEC3H
GS_adaptive
GS_CC
GS_DDR
GS_innate
G2/M
ActivatedCD4
G2
CPF
MemoryB
HR
Effector_mCD4
BER
Tcells
Central_mCD8
TGD
TFH
ActivatedB
ImmatureB
MDSC
Monocytes
Central_mCD4
NHEJ

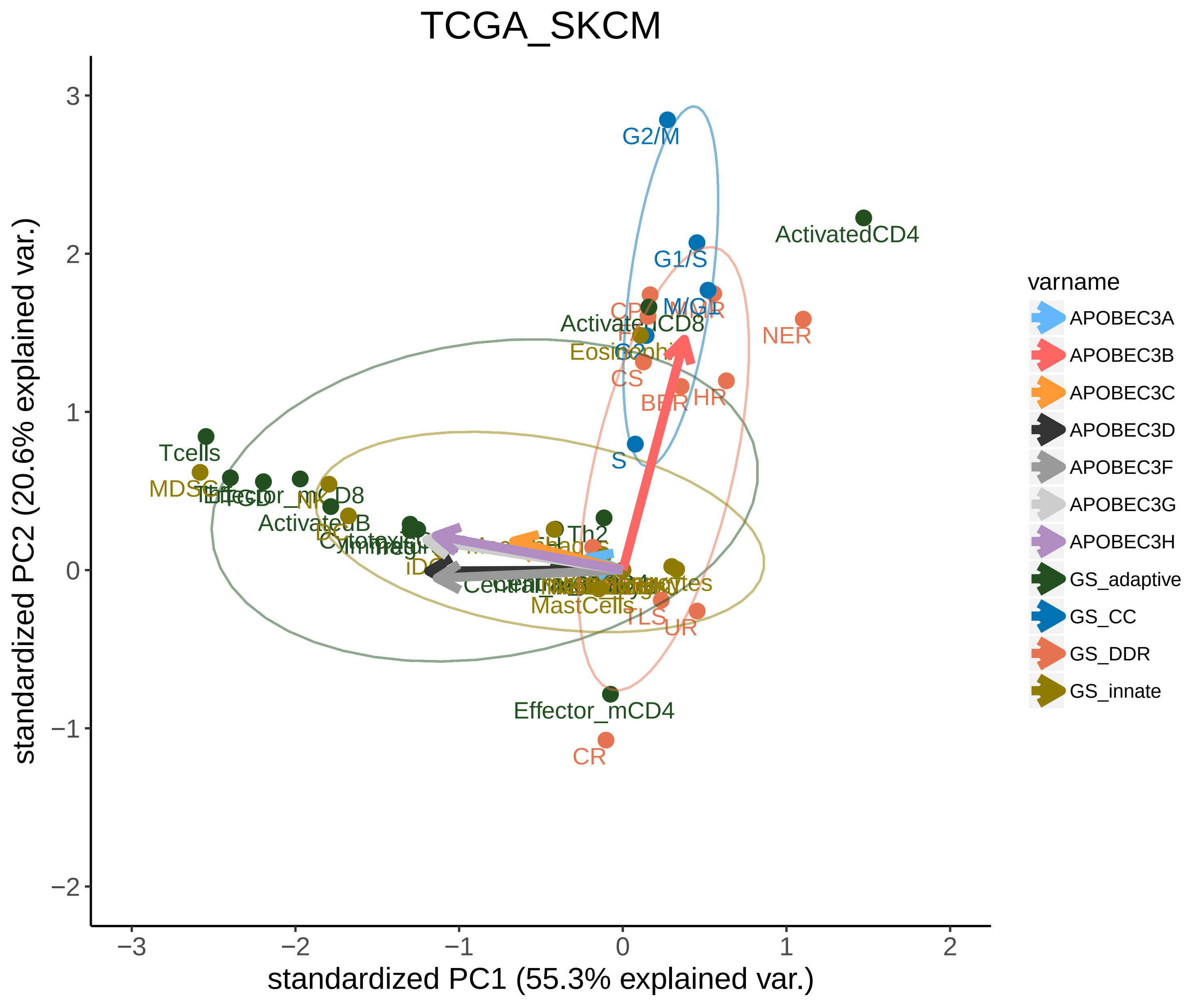
TCGA_SKCM
standardized PC2 (20.6% explained var.)
standardized PC1 (55.3% explained var.)
3
2
1
0
−1
−2
−3
−2
−1
0
1
2
G2/M
ActivatedCD4
G1/S
ActivatedCD8
NER
Eosinophil
CS
BER
HR
S
Tcells
MDSC
ActivatedB
Th2
MastCells
TLS
UR
Effector_mCD4
CR
varname
APOBEC3A
APOBEC3B
APOBEC3C
APOBEC3D
APOBEC3F
APOBEC3G
APOBEC3H
GS_adaptive
GS_CC
GS_DDR
GS_innate

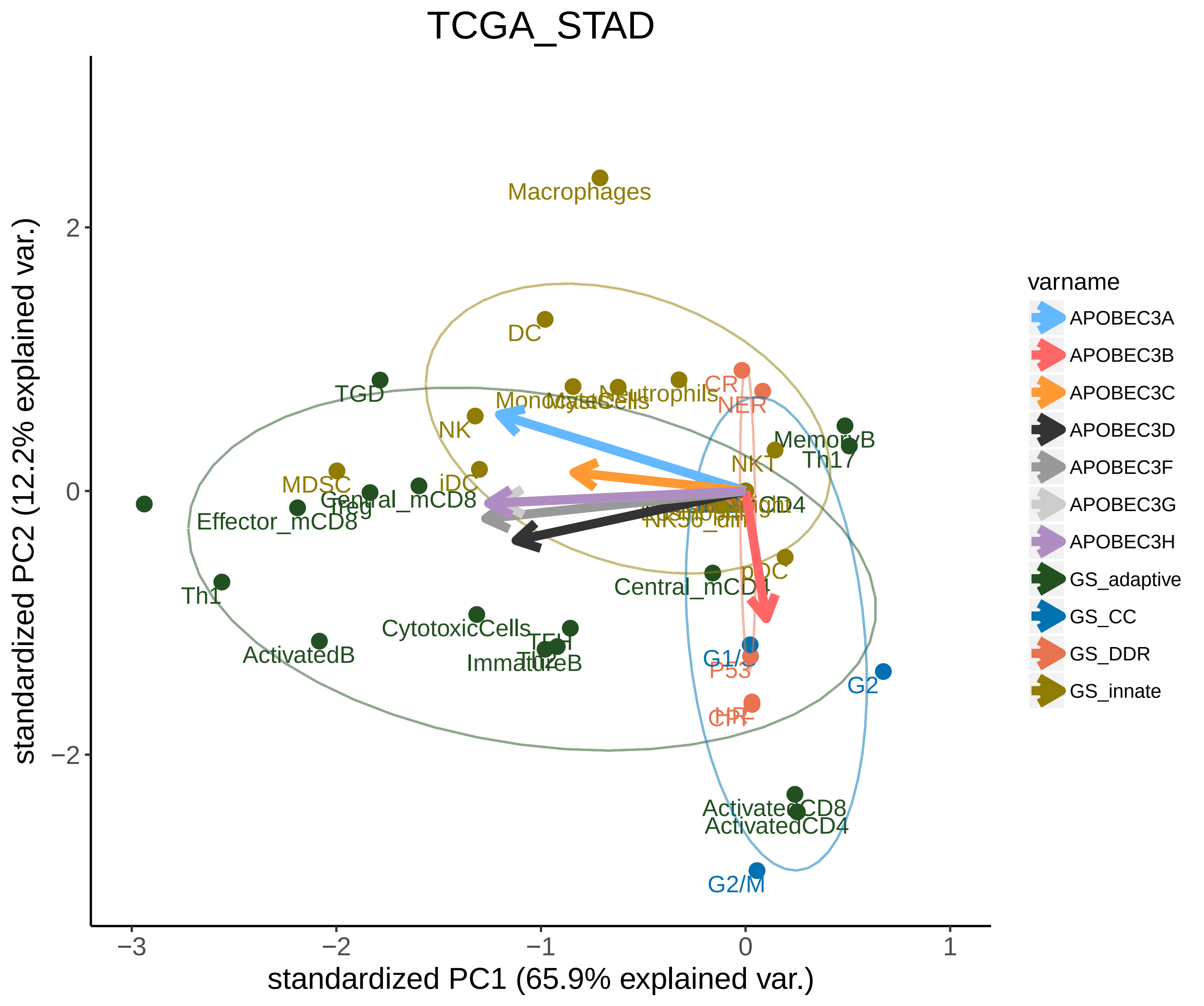
TCGA_STAD
standardized PC2 (12.2% explained var.)
standardized PC1 (65.9% explained var.)
2
0
−2
−3
−2
−1
0
1
Macrophages
DC
TGD
NK
Neutrophils
CR
NER
MemoryB
Th17
NKT
MDSC
iDC
Central_mCD8
Treg
Effector_mCD8
Th1
pDC
Central_mCD4
CytotoxicCells
ActivatedB
ImmatureB
G1/S
P53
G2
ActivatedCD8
ActivatedCD4
G2/M
varname
APOBEC3A
APOBEC3B
APOBEC3C
APOBEC3D
APOBEC3F
APOBEC3G
APOBEC3H
GS_adaptive
GS_CC
GS_DDR
GS_innate

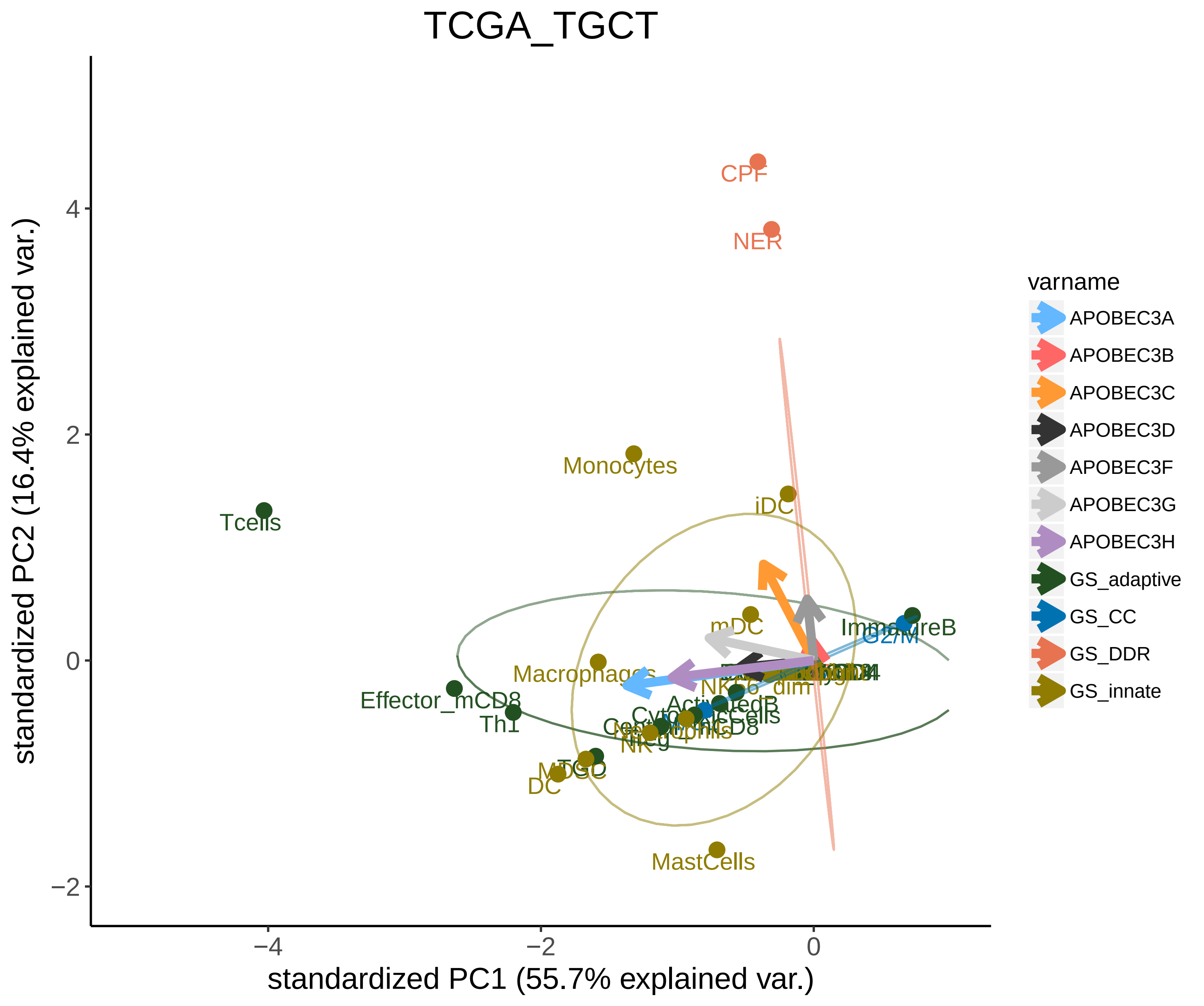
TCGA_TGCT
standardized PC1 (55.7% explained var.)
standardized PC2 (16.4% explained var.)
−4
−2
0
−2
0
2
4
CPF
NER
Monocytes
Tcells
iDC
mDC
ImmatureB
G2/M
Macrophages
Effector_mCD8
Th1
MastCells
DC
varname
APOBEC3A
APOBEC3B
APOBEC3C
APOBEC3D
APOBEC3F
APOBEC3G
APOBEC3H
GS_adaptive
GS_CC
GS_DDR
GS_innate

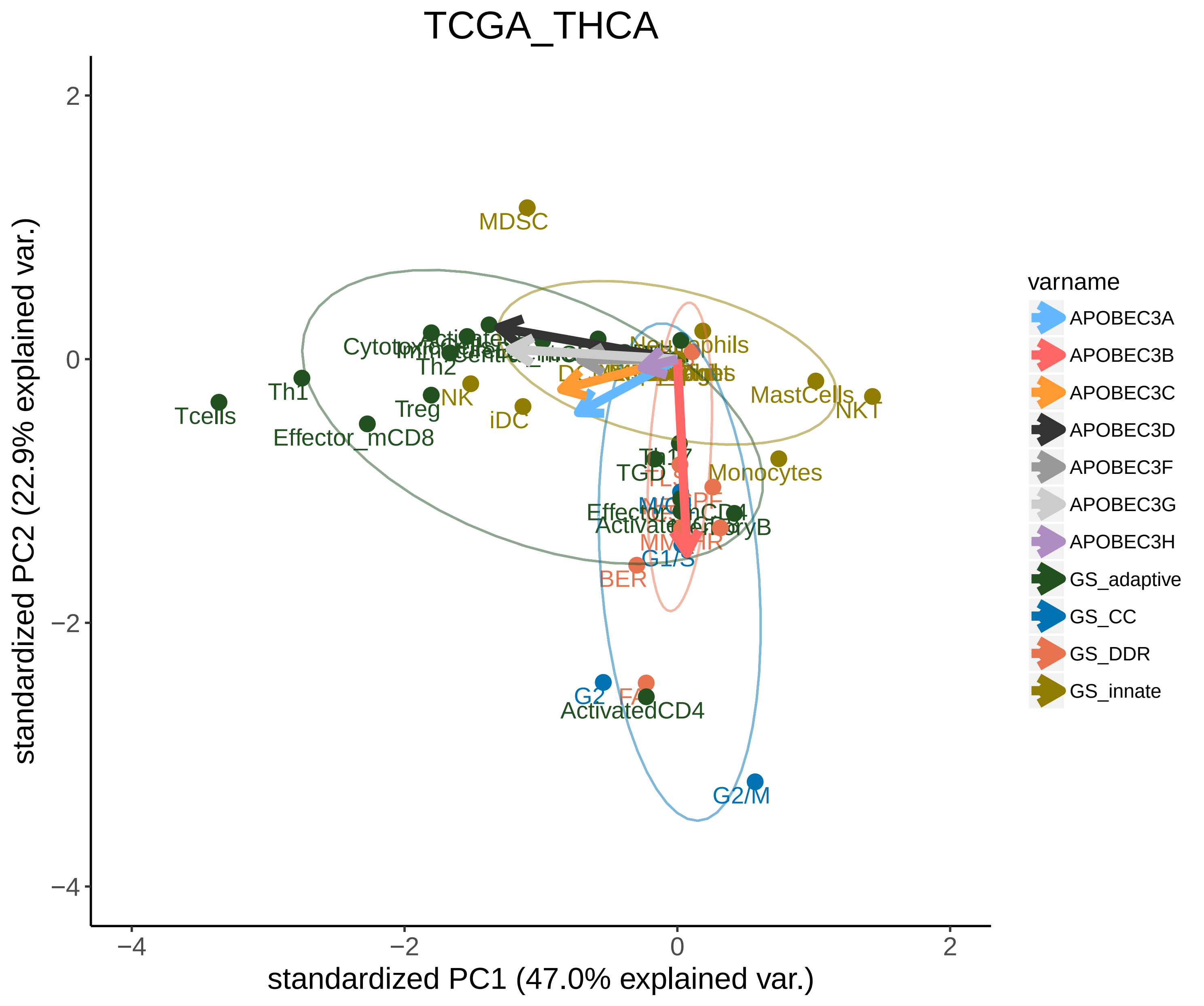
TCGA_THCA
standardized PC1 (47.0% explained var.)
standardized PC2 (22.9% explained var.)
−4
−2
0
2
MDSC
Th2
Th1
Tcells
Treg
NK
iDC
Effector_mCD8
MastCells
NKT
Monocytes
TGD
BER
G1/S
G2
ActivatedCD4
G2/M
varname
APOBEC3A
APOBEC3B
APOBEC3C
APOBEC3D
APOBEC3F
APOBEC3G
APOBEC3H
GS_adaptive
GS_CC
GS_DDR
GS_innate

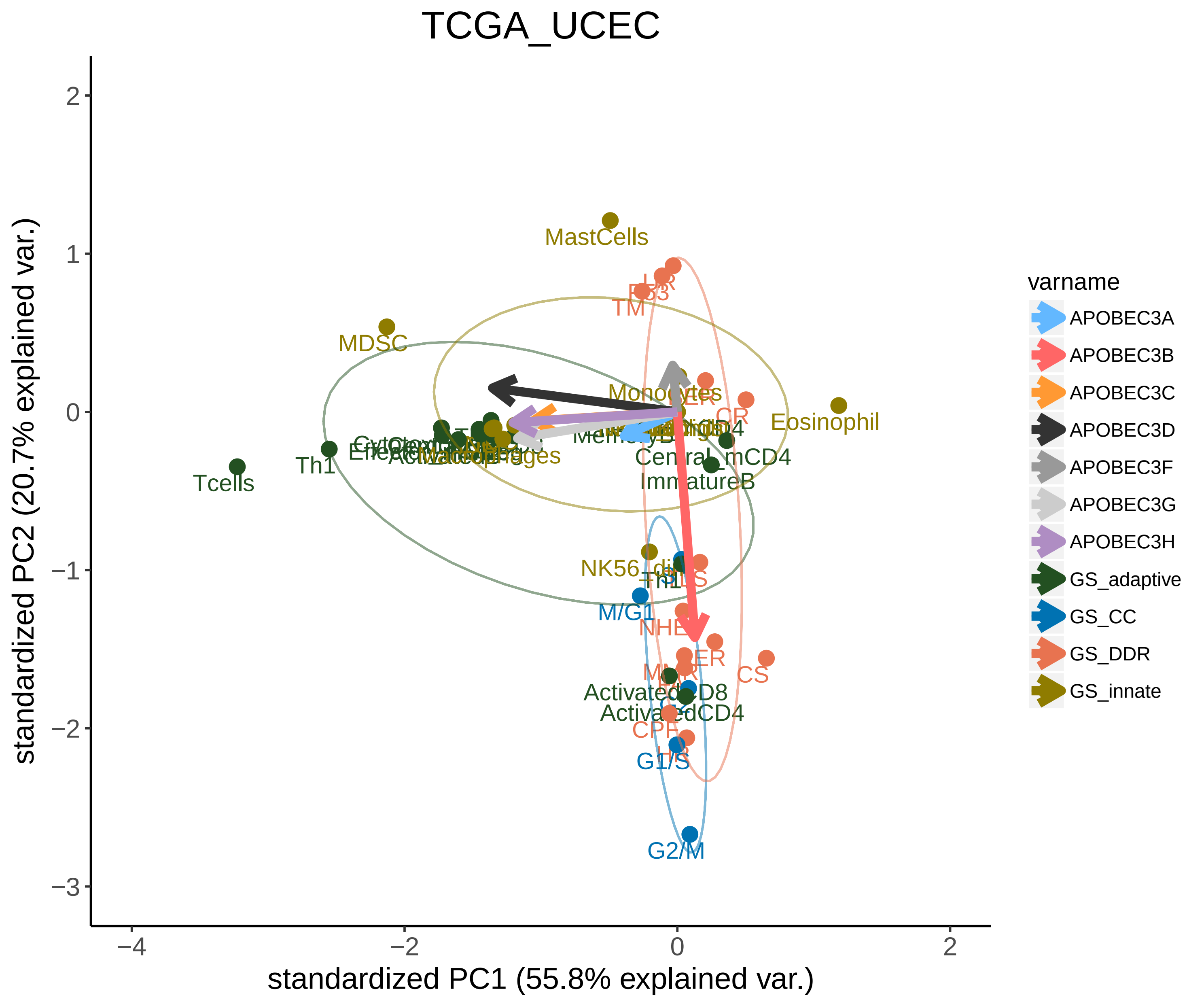
TCGA_UCEC
standardized PC1 (55.8% explained var.)
standardized PC2 (20.7% explained var.)
−4
−2
0
2
−3
−2
−1
0
1
2
varname
APOBEC3A
APOBEC3B
APOBEC3C
APOBEC3D
APOBEC3F
APOBEC3G
APOBEC3H
GS_adaptive
GS_CC
GS_DDR
GS_innate
MastCells
MDSC
Monocytes
Eosinophil
TM
Tcells
Th1
Central_mCD4
ImmatureB
NK56_dim
M/G1
NHEJ
MMR
CS
ActivatedCD8
ActivatedCD4
CPF
G1/S
G2/M

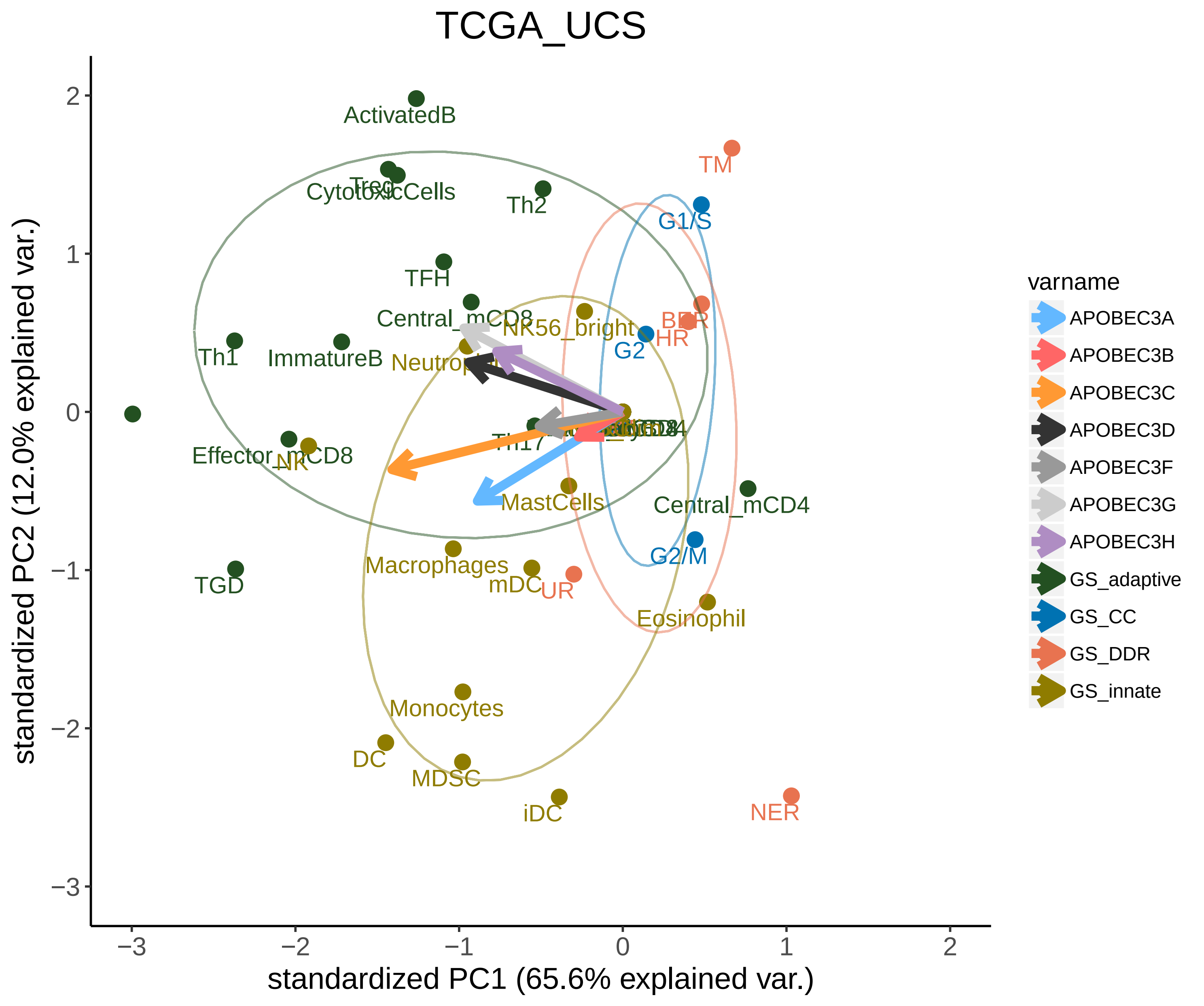

TCGA_UCS
standardized PC2 (12.0% explained var.)
standardized PC1 (65.6% explained var.)
2
1
0
−1
−2
−3
ActivatedB
TM
CytotoxicCells
Th2
G1/S
TFH
Central_mCD8
NK56_bright
HR
G2
Th1
ImmatureB
Th17
Effector_mCD8
NK
MastCells
Central_mCD4
G2/M
TGD
Macrophages
mDC
UR
Eosinophil
Monocytes
DC
MDSC
iDC
NER
varname
APOBEC3A
APOBEC3B
APOBEC3C
APOBEC3D
APOBEC3F
APOBEC3G
APOBEC3H
GS_adaptive
GS_CC
GS_DDR
GS_innate
