# Supplementary material for: Pan-cancer transcriptomic analysis dissects immune and proliferative functions of APOBEC3 cytidine deaminases
Source: Nucleic Acids Res. 2019 Jan 9;47(3):1178–94. doi: 10.1093/nar/gky1316 (PMC6379723; doi:10.1093/nar/gky1316)
Supplement: Supplementary Data [file gky1316_supplemental_files.zip › FigureS16_gsea_bs_pca.pdf]

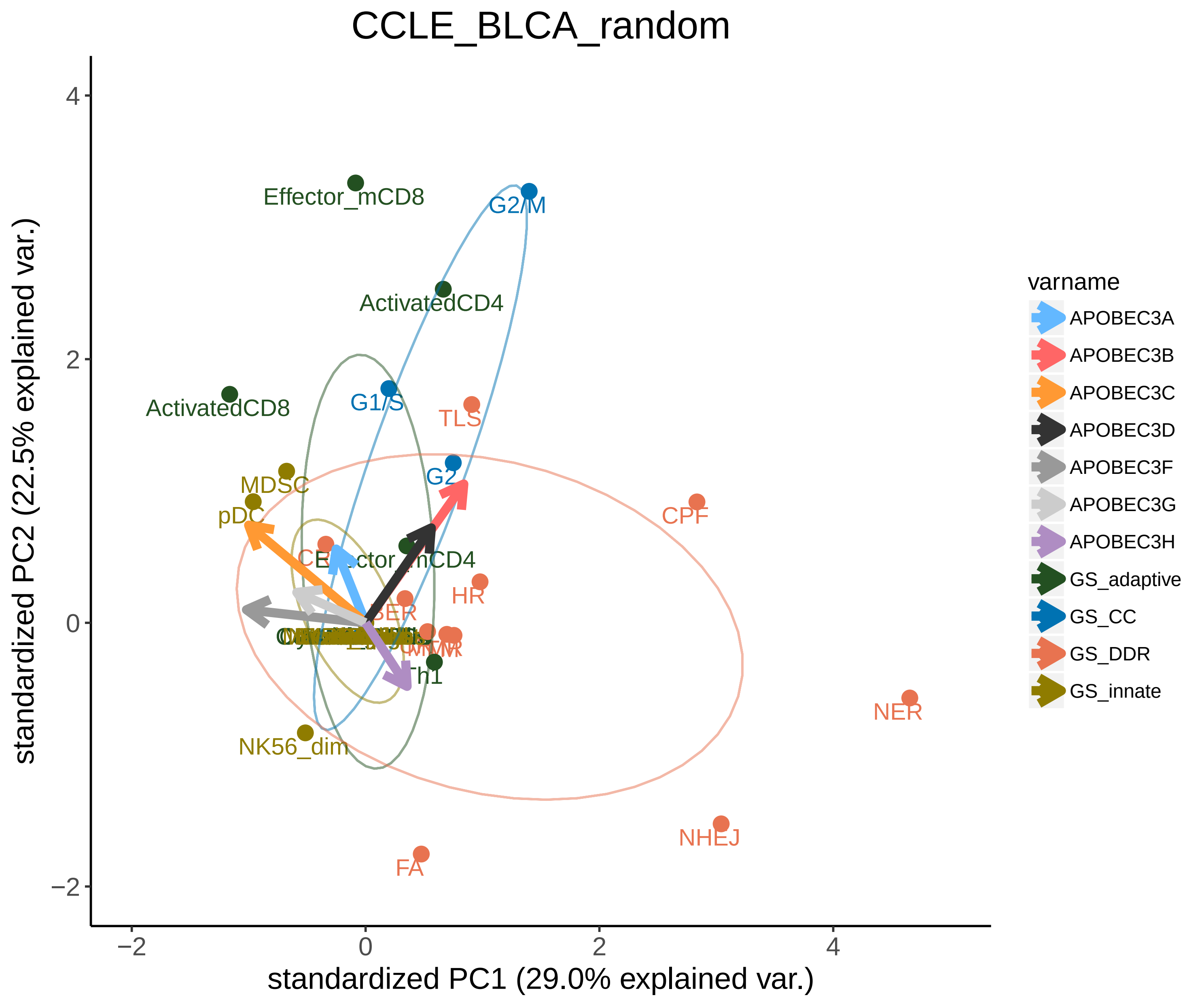

CCLE_BLCA_random
standardized PC1 (29.0% explained var.)
standardized PC2 (22.5% explained var.)
−2
0
2
4
−2
0
2
4
Effector_mCD8
G2/M
ActivatedCD4
ActivatedCD8
G1/S
TLS
MDSC
G2
pDC
CPF
HR
BER
Th1
NER
NK56_dim
NHEJ
FA
varname
APOBEC3A
APOBEC3B
APOBEC3C
APOBEC3D
APOBEC3F
APOBEC3G
APOBEC3H
GS_adaptive
GS_CC
GS_DDR
GS_innate

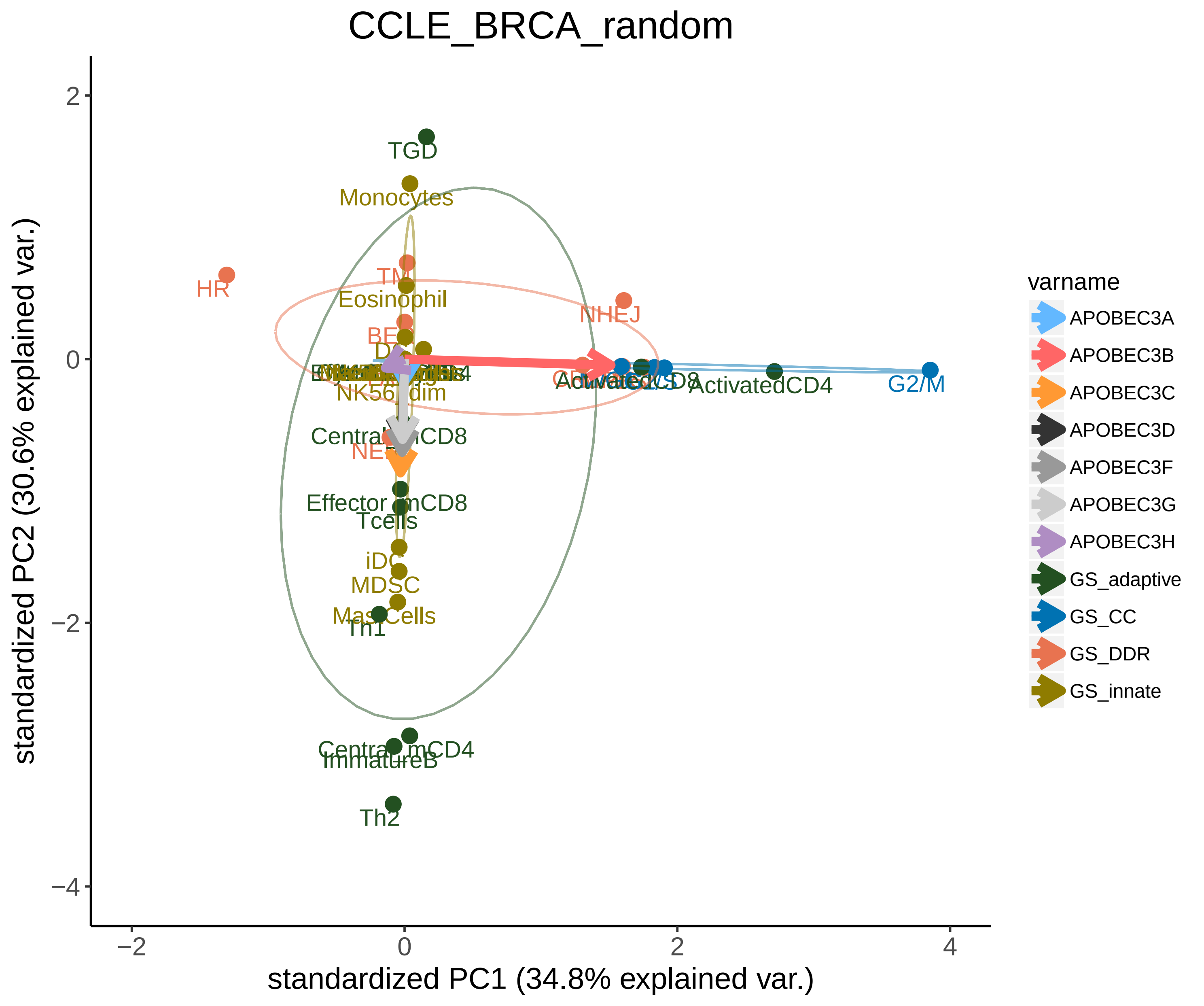
CCLE_BRCA_random
standardized PC1 (34.8% explained var.)
standardized PC2 (30.6% explained var.)
varname
APOBEC3A
APOBEC3B
APOBEC3C
APOBEC3D
APOBEC3F
APOBEC3G
APOBEC3H
GS_adaptive
GS_CC
GS_DDR
GS_innate
TGD
Monocytes
HR
TM
Eosinophil
NHEJ
NK56_dim
ActivatedCD4
G2/M
Effector_mCD8
Tcells
iDC
MDSC
MastCells
Th1
ImmatureB
Th2
2
0
−2
−4
4

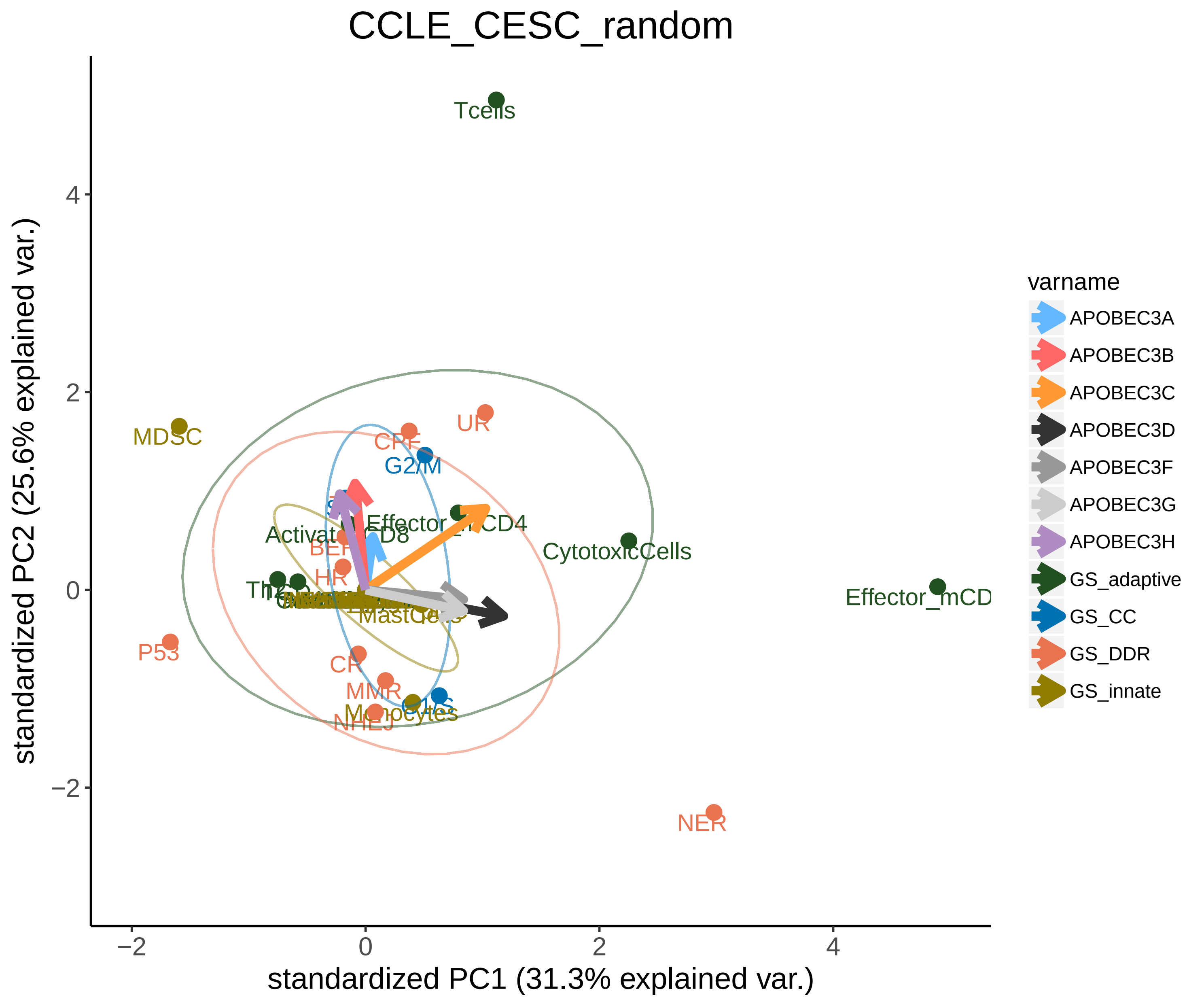
CCLE_CESC_random
standardized PC1 (31.3% explained var.)
standardized PC2 (25.6% explained var.)
Tcells
UR
CPF
MDSC
G2/M
Effector_mCD4
ActivatedCD8
CytotoxicCells
BER
HR
Effector_mCD
P53
MastCells
CR
MMR
Monocytes
NHEJ
NER
varname
APOBEC3A
APOBEC3B
APOBEC3C
APOBEC3D
APOBEC3F
APOBEC3G
APOBEC3H
GS_adaptive
GS_CC
GS_DDR
GS_innate

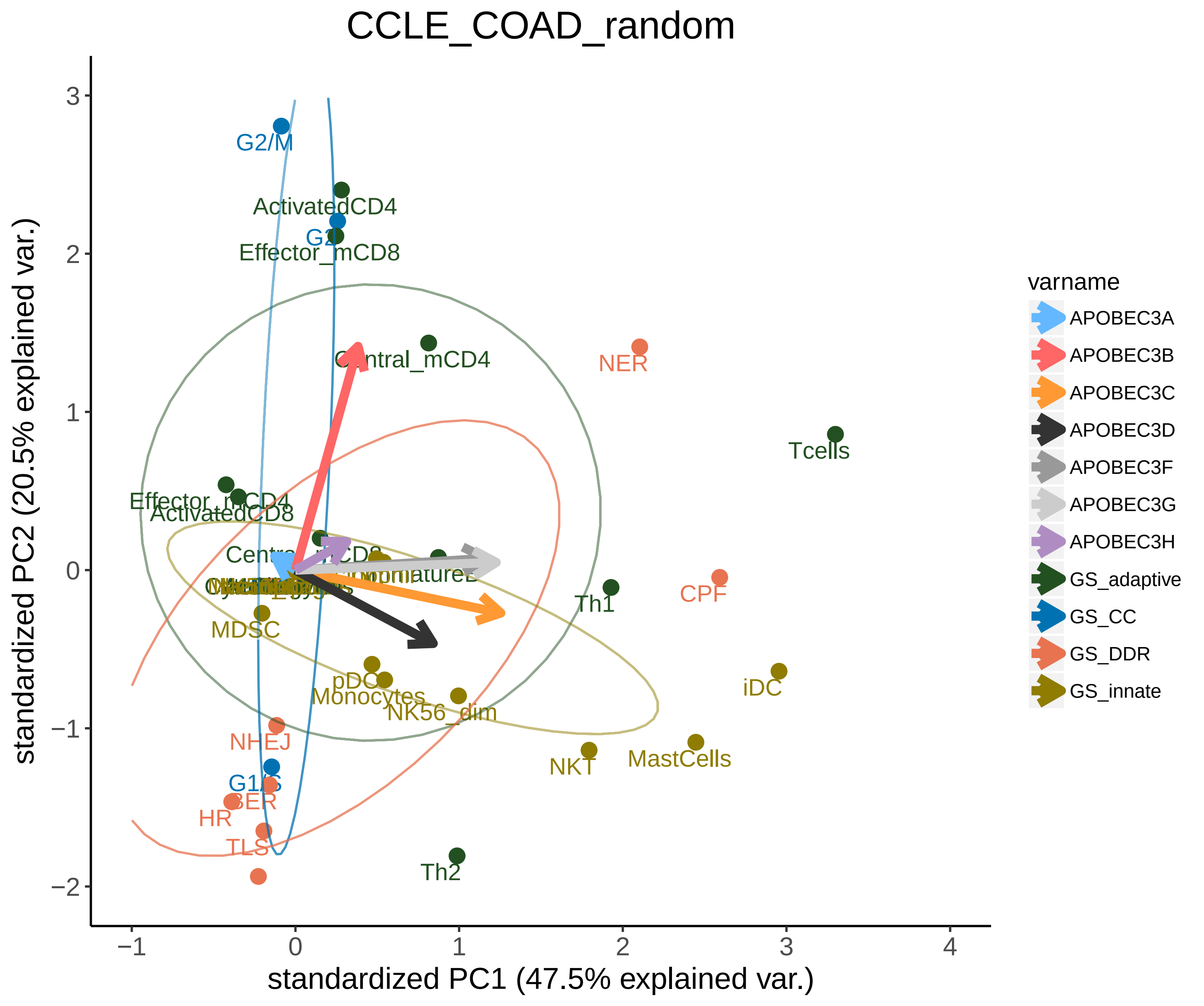

CCLE_COAD_random
standardized PC1 (47.5% explained var.)
standardized PC2 (20.5% explained var.)
G2/M
ActivatedCD4
G2
Effector_mCD8
Central_mCD4
NER
Tcells
Effector_mCD4
ActivatedCD8
MDSC
Th1
CPF
pDC
Monocytes
NK56_dim
iDC
NHEJ
NKT
MastCells
G1/S
BER
HR
TLS
Th2
varname
APOBEC3A
APOBEC3B
APOBEC3C
APOBEC3D
APOBEC3F
APOBEC3G
APOBEC3H
GS_adaptive
GS_CC
GS_DDR
GS_innate

CCLE_DLBC_random

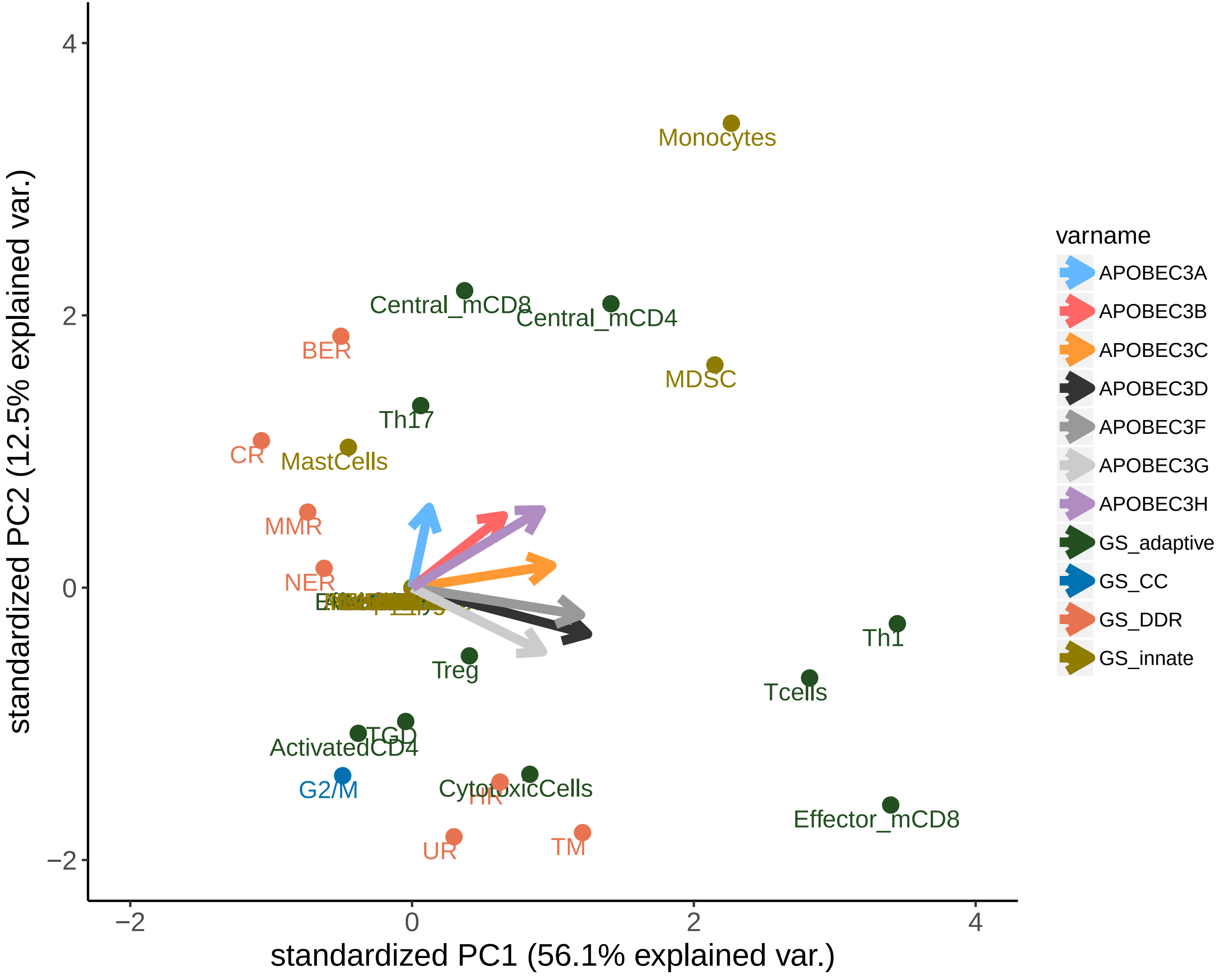

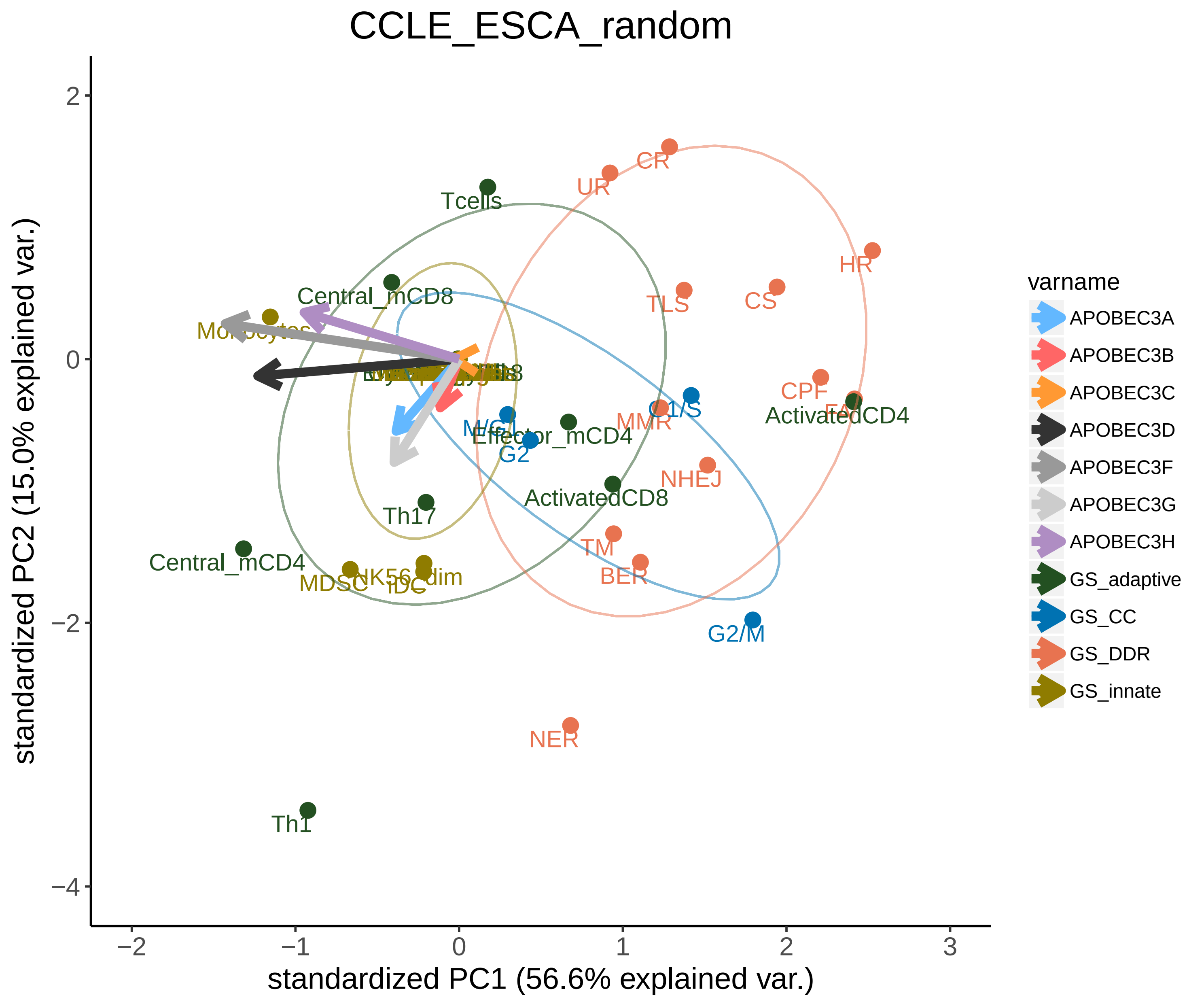

CCLE_ESCA_random
standardized PC1 (56.6% explained var.)
standardized PC2 (15.0% explained var.)
varname
APOBEC3A
APOBEC3B
APOBEC3C
APOBEC3D
APOBEC3F
APOBEC3G
APOBEC3H
GS_adaptive
GS_CC
GS_DDR
GS_innate
Tcells
CR
UR
HR
TLS
CS
Central_mCD8
CPF
G1/S
MMR
ActivatedCD4
M/G1
Effector_mCD4
G2
NHEJ
ActivatedCD8
Th17
TM
BER
Central_mCD4
MDSC
IDC
G2/M
NER
Th1
2
0
−2
−4
−2
−1
0
1
2
3

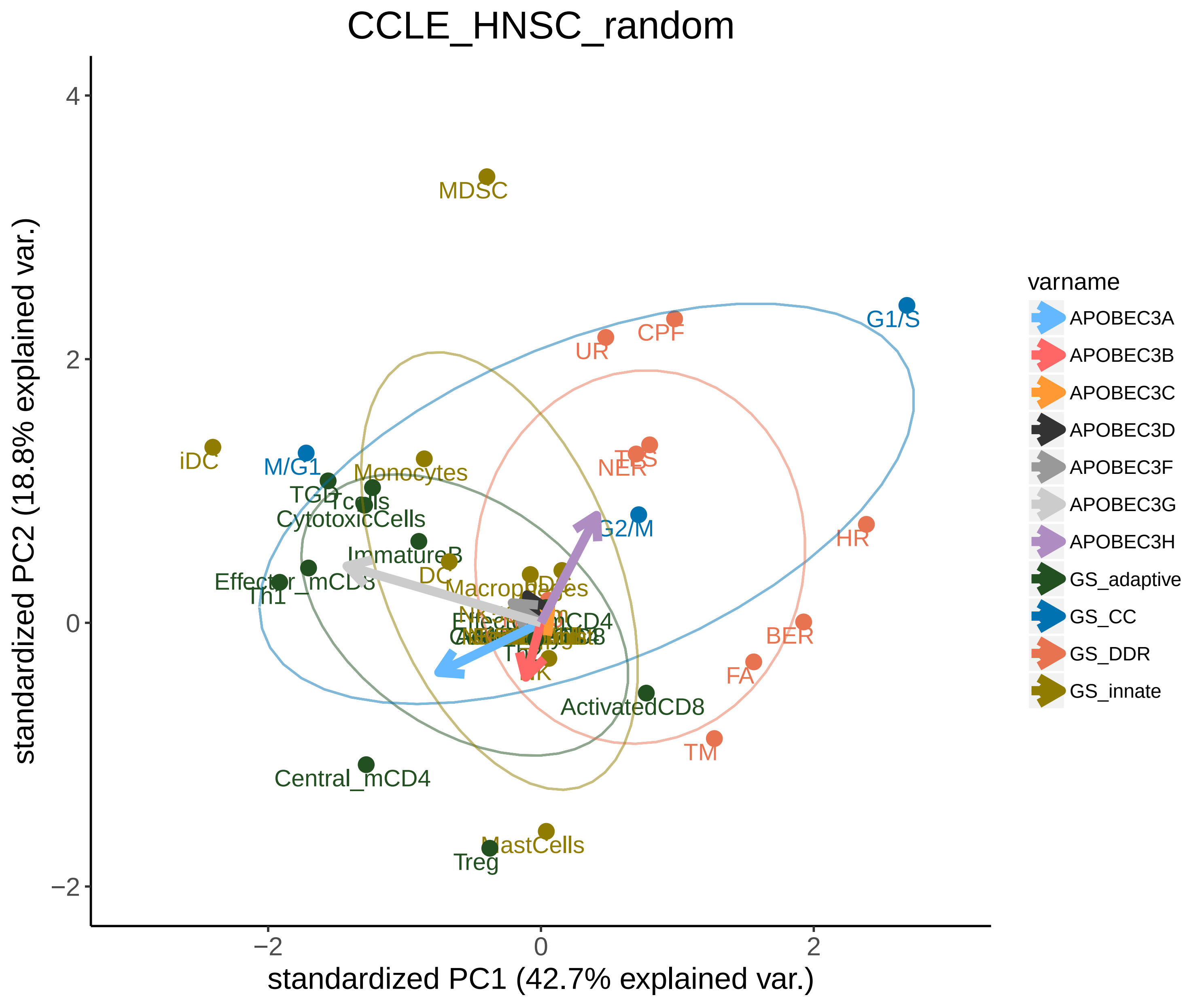

CCLE_HNSC_random
standardized PC2 (18.8% explained var.)
standardized PC1 (42.7% explained var.)
4
2
0
−2
−2
0
2
MDSC
iDC
M/G1
Monocytes
TGD
Tcells
CytotoxicCells
ImmatureB
DC
Effector_mCD8
Th1
Macrophages
Central_mCD4
MastCells
Treg
ActivatedCD8
G1/S
CPF
UR
NER
G2/M
HR
BER
FA
TM
NK
varname
APOBEC3A
APOBEC3B
APOBEC3C
APOBEC3D
APOBEC3F
APOBEC3G
APOBEC3H
GS_adaptive
GS_CC
GS_DDR
GS_innate

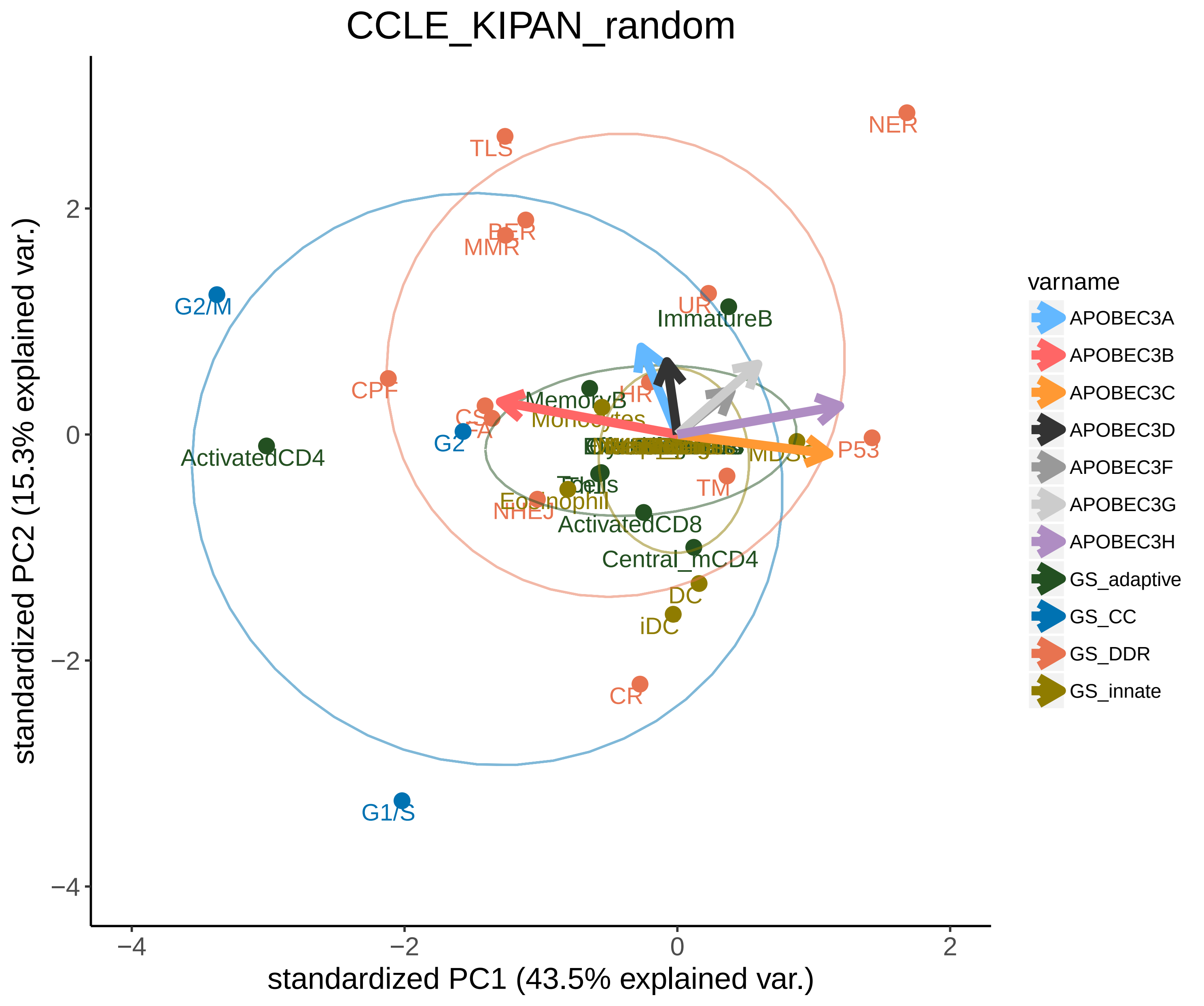

CCLE_KIPAN_random
standardized PC2 (15.3% explained var.)
standardized PC1 (43.5% explained var.)
2
0
−2
−4
−4
−2
0
2
NER
TLS
BER
MMR
G2/M
UR
ImmatureB
CPF
MemoryB
HR
CS
FA
G2
ActivatedCD4
P53
TM
Eosinophil
NHEJ
ActivatedCD8
Central_mCD4
DC
iDC
CR
G1/S
varname
APOBEC3A
APOBEC3B
APOBEC3C
APOBEC3D
APOBEC3F
APOBEC3G
APOBEC3H
GS_adaptive
GS_CC
GS_DDR
GS_innate

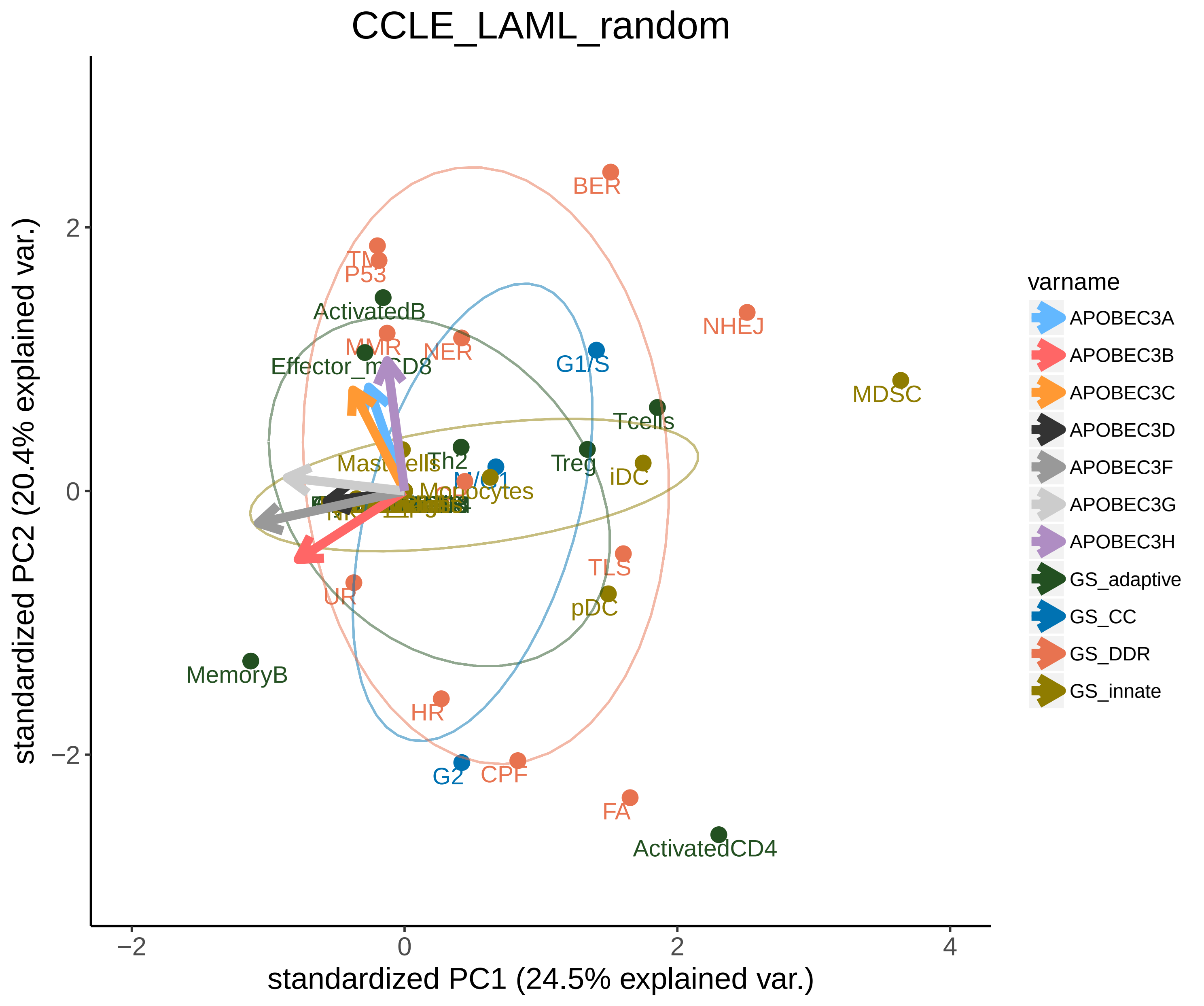

CCLE_LAML_random
standardized PC1 (24.5% explained var.)
standardized PC2 (20.4% explained var.)
−2
0
2
4
2
0
−2
BER
NHEJ
MDSC
ActivatedB
MMR
NER
Effector_memCD8
G1/S
Tcells
Mast cells
Th2
Treg
iDC
Monocytes
TLS
UR
pDC
MemoryB
HR
G2
CPF
FA
ActivatedCD4
P53
varname
APOBEC3A
APOBEC3B
APOBEC3C
APOBEC3D
APOBEC3F
APOBEC3G
APOBEC3H
GS_adaptive
GS_CC
GS_DDR
GS_innate

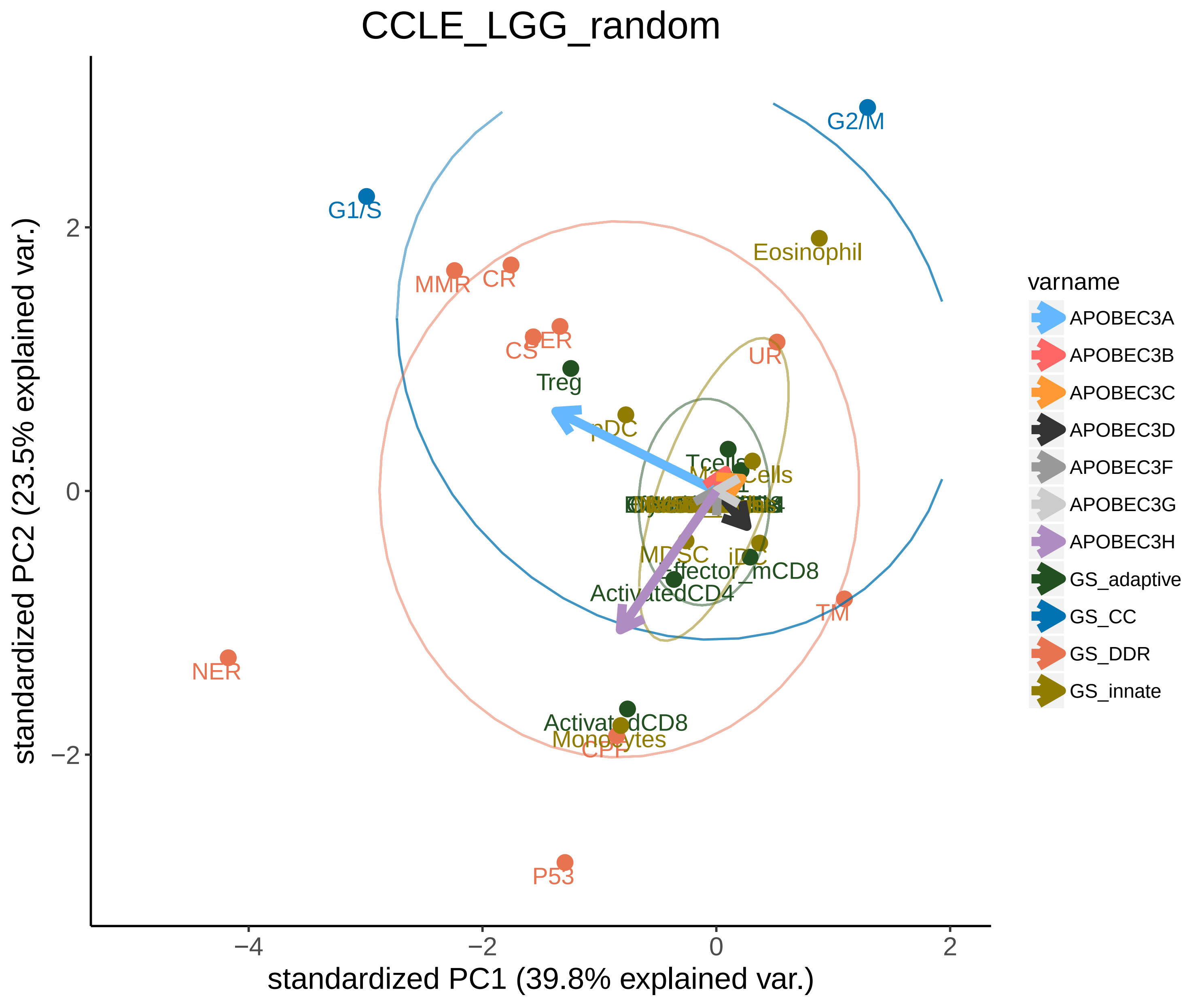

CCLE_LGG_random
standardized PC1 (39.8% explained var.)
standardized PC2 (23.5% explained var.)
varname
APOBEC3A
APOBEC3B
APOBEC3C
APOBEC3D
APOBEC3F
APOBEC3G
APOBEC3H
GS_adaptive
GS_CC
GS_DDR
GS_innate
G2/M
G1/S
Eosinophil
MMR
CR
CS
UR
Treg
pDC
Tcells
iDC
MDSC
Effector_mCD8
ActivatedCD4
TM
NER
ActivatedCD8
Monocytes
CPF
P53

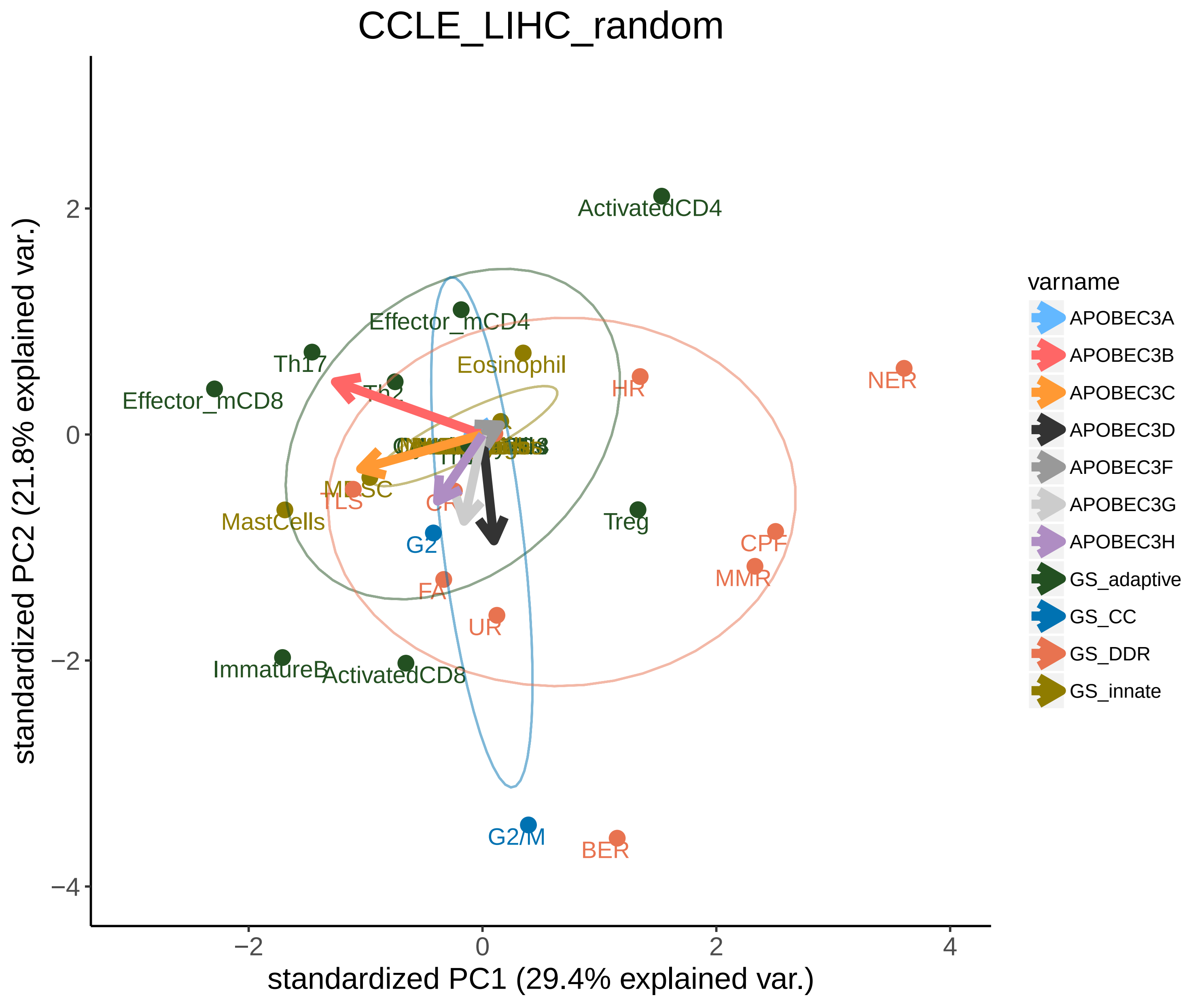

CCLE_LIHC_random
standardized PC1 (29.4% explained var.)
standardized PC2 (21.8% explained var.)
−2
0
2
4
−4
ActivatedCD4
Effector_mCD4
Th17
Effector_mCD8
Th2
Eosinophil
HR
NER
MDSC
TLS
MastCells
CR
Treg
CPF
G2
MMR
FA
UR
ImmatureB
ActivatedCD8
G2/M
BER
varname
APOBEC3A
APOBEC3B
APOBEC3C
APOBEC3D
APOBEC3F
APOBEC3G
APOBEC3H
GS_adaptive
GS_CC
GS_DDR
GS_innate

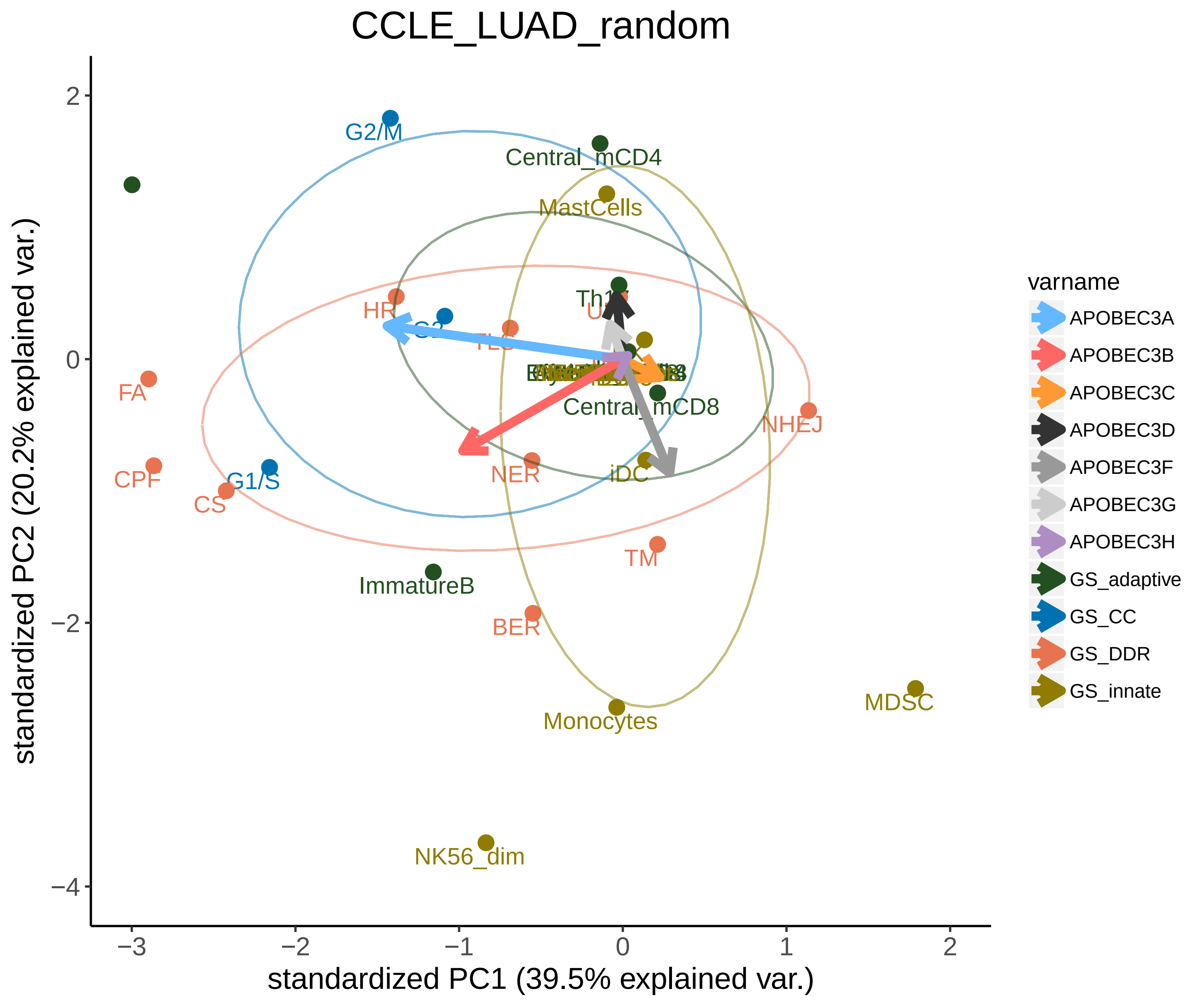

CCLE_LUAD_random
standardized PC1 (39.5% explained var.)
standardized PC2 (20.2% explained var.)
G2/M
Central_mCD4
MastCells
Th17
HR
FLC
FA
CPF
CS
G1/S
NER
iDC
Central_mCD8
NHEJ
TM
ImmatureB
BER
Monocytes
MDSC
NK56_dim
varname
APOBEC3A
APOBEC3B
APOBEC3C
APOBEC3D
APOBEC3F
APOBEC3G
APOBEC3H
GS_adaptive
GS_CC
GS_DDR
GS_innate

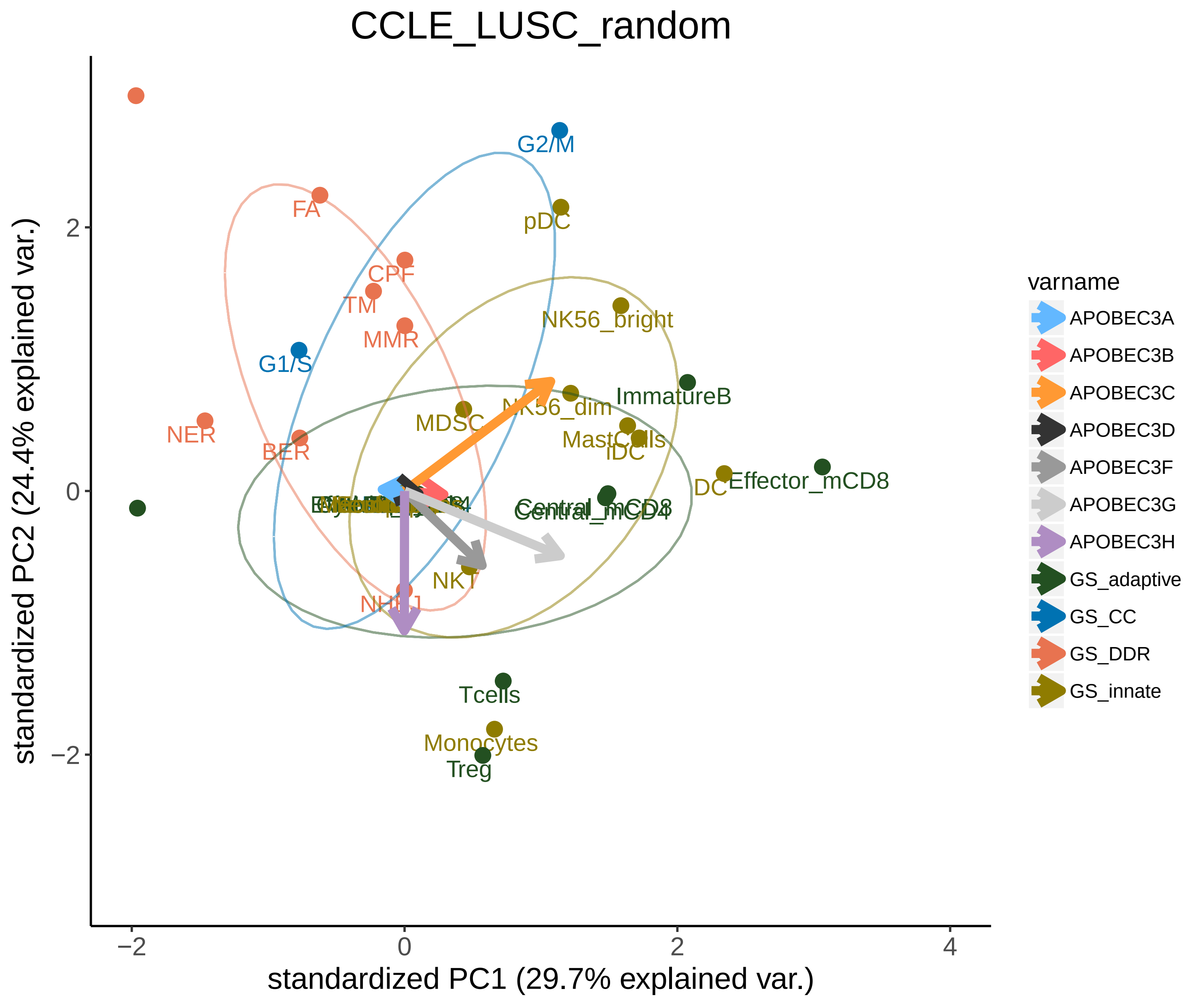

CCLE_LUSC_random
standardized PC2 (24.4% explained var.)
standardized PC1 (29.7% explained var.)
2
0
−2
−2
0
2
4
G2/M
FA
pDC
CPF
TM
NK56_bright
MMR
G1/S
ImmatureB
NK56_dim
MDSC
NER
MastCells
BER
iDC
DC
Effector_mCD8
Central_mCD8
Central_mCD4
NKT
NHEJ
Tcells
Monocytes
Treg
varname
APOBEC3A
APOBEC3B
APOBEC3C
APOBEC3D
APOBEC3F
APOBEC3G
APOBEC3H
GS_adaptive
GS_CC
GS_DDR
GS_innate

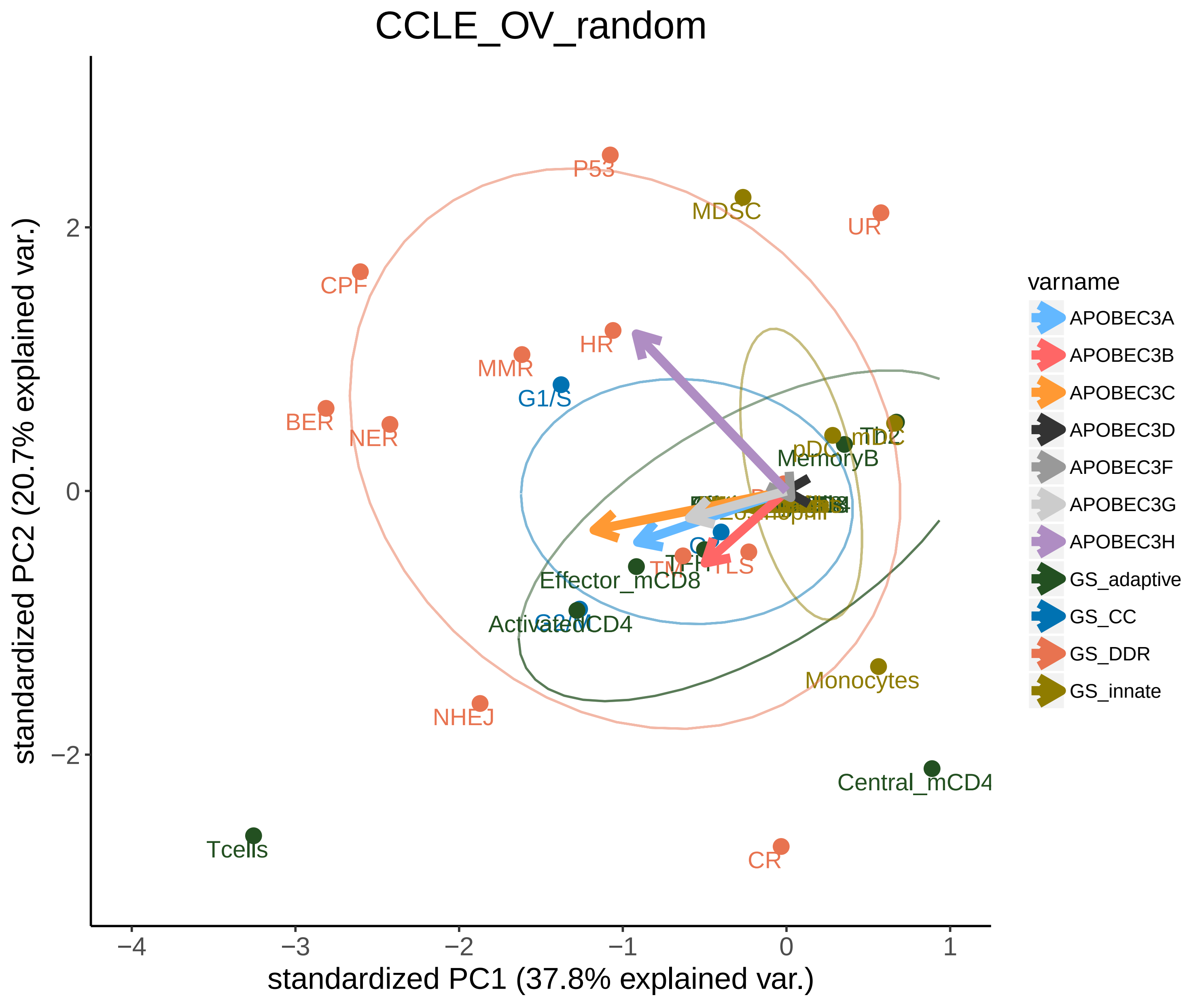

CCLE_OV_random
standardized PC1 (37.8% explained var.)
standardized PC2 (20.7% explained var.)
P53
MDSC
UR
CPF
HR
MMR
G1/S
BER
NER
MemoryB
Effector_mCD8
Activated_mCD4
Monocytes
NHEJ
Central_mCD4
Tcells
CR
varname
APOBEC3A
APOBEC3B
APOBEC3C
APOBEC3D
APOBEC3F
APOBEC3G
APOBEC3H
GS_adaptive
GS_CC
GS_DDR
GS_innate
2
0
−2
−4
−3
−2
−1
0
1

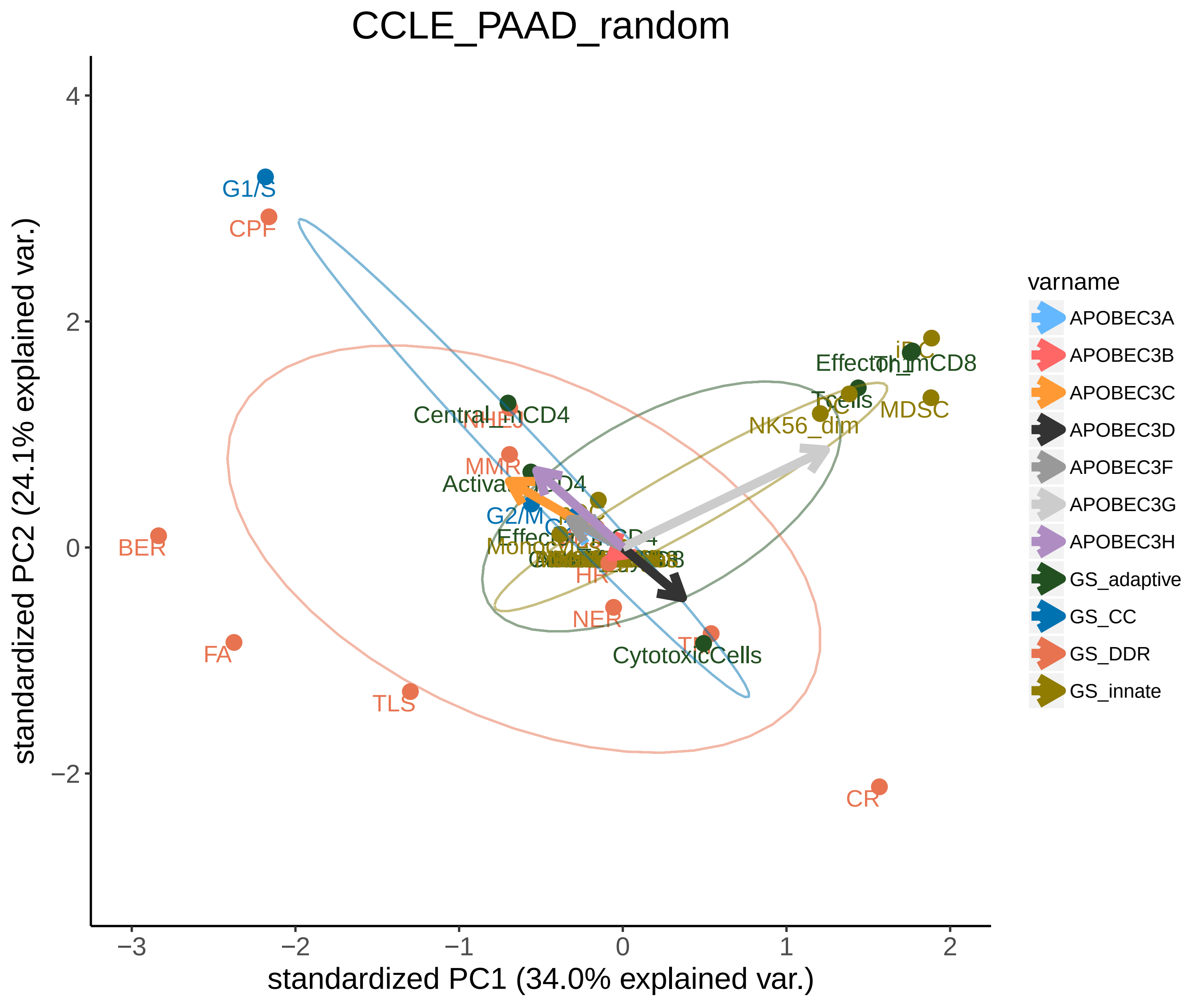

CCLE_PAAD_random
standardized PC1 (34.0% explained var.)
standardized PC2 (24.1% explained var.)
−3
−2
−1
0
1
2
4
2
0
−2
G1/S
CPF
BER
FA
TLS
MMR
NER
HR
CR
G2/M
Central_mCD4
CytotoxicCells
NK56_dim
MDSC
varname
APOBEC3A
APOBEC3B
APOBEC3C
APOBEC3D
APOBEC3F
APOBEC3G
APOBEC3H
GS_adaptive
GS_CC
GS_DDR
GS_innate

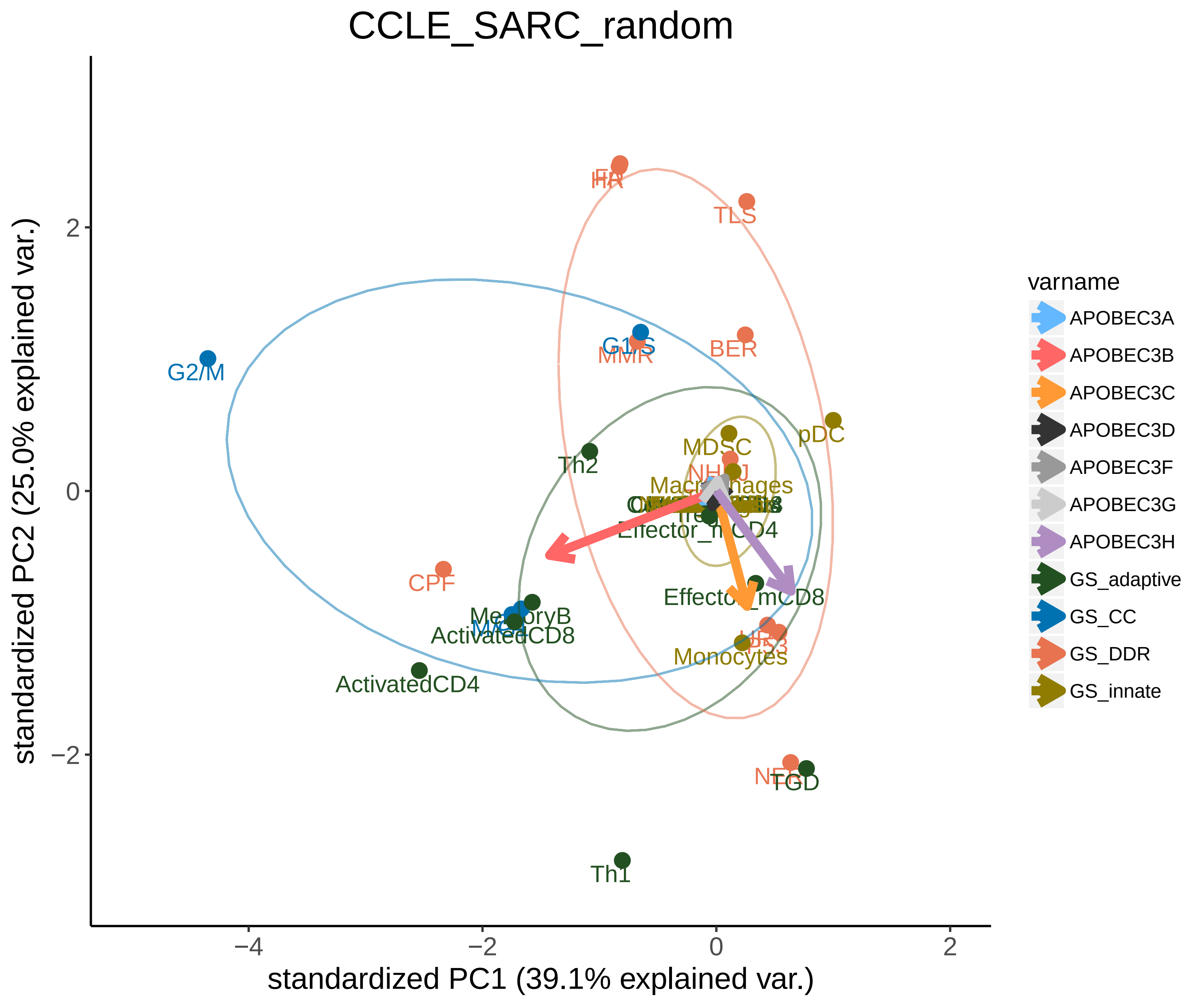

CCLE_SARC_random
standardized PC1 (39.1% explained var.)
standardized PC2 (25.0% explained var.)
−4
−2
0
2
−2
0
2
G2/M
G1/S
MMR
BER
TLS
HR
FA
CPF
NHEJ
MDSC
pDC
Macrophages
Th2
Th1
Treg
Effector_mCD4
Effector_mCD8
MemoryB
ActivatedCD8
ActivatedCD4
Monocytes
TGD
varname
APOBEC3A
APOBEC3B
APOBEC3C
APOBEC3D
APOBEC3F
APOBEC3G
APOBEC3H
GS_adaptive
GS_CC
GS_DDR
GS_innate

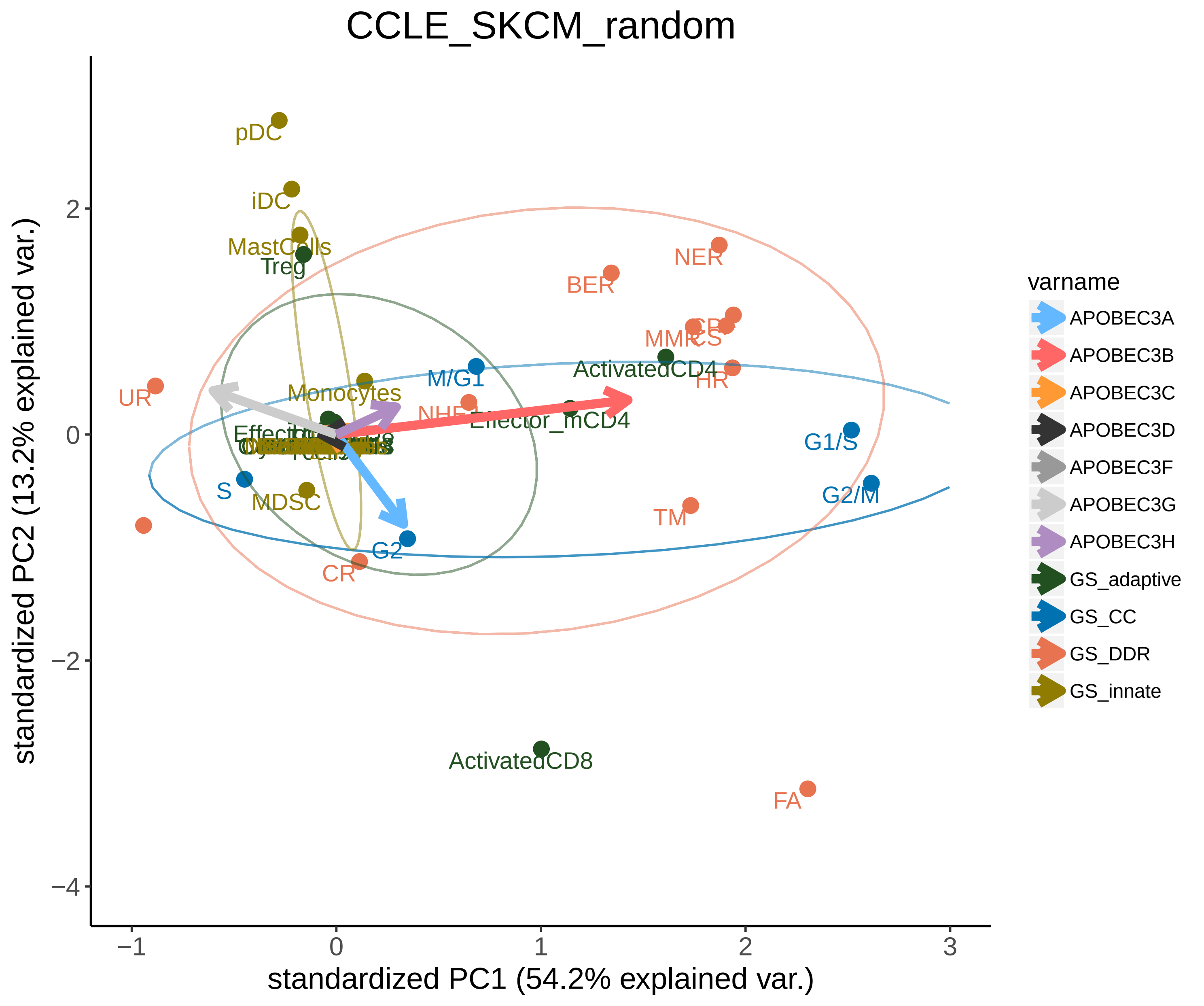
CCLE_SKCM_random
standardized PC1 (54.2% explained var.)
standardized PC2 (13.2% explained var.)
−1
0
1
2
3
2
0
−2
−4
pDC
iDC
MastCells
Treg
NER
BER
MMR
CS
ActivatedCD4
HR
M/G1
Monocytes
UR
Effector_mCD4
G1/S
S
MDSC
G2/M
TM
G2
CR
ActivatedCD8
FA
varname
APOBEC3A
APOBEC3B
APOBEC3C
APOBEC3D
APOBEC3F
APOBEC3G
APOBEC3H
GS_adaptive
GS_CC
GS_DDR
GS_innate

CCLE_STAD_random

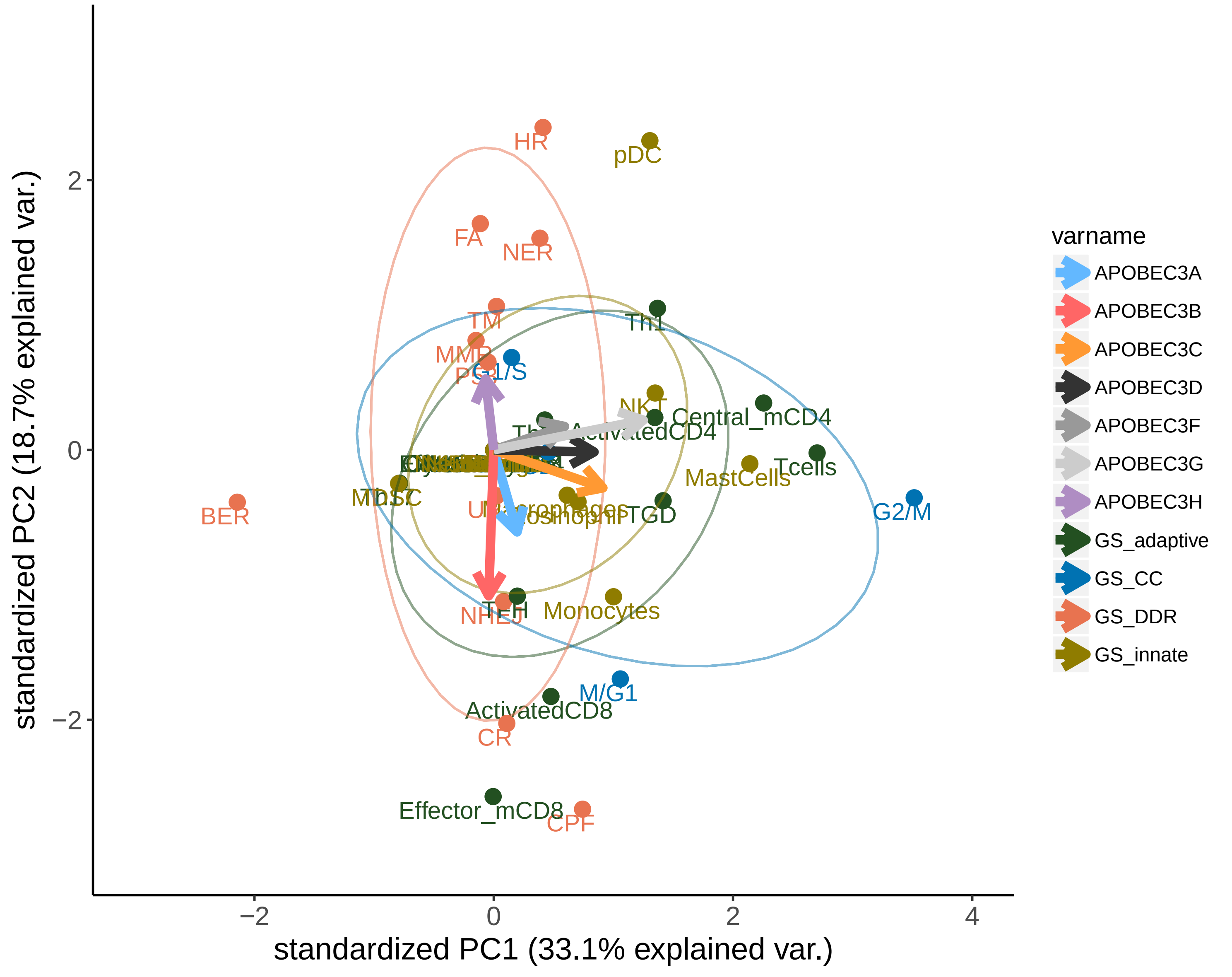

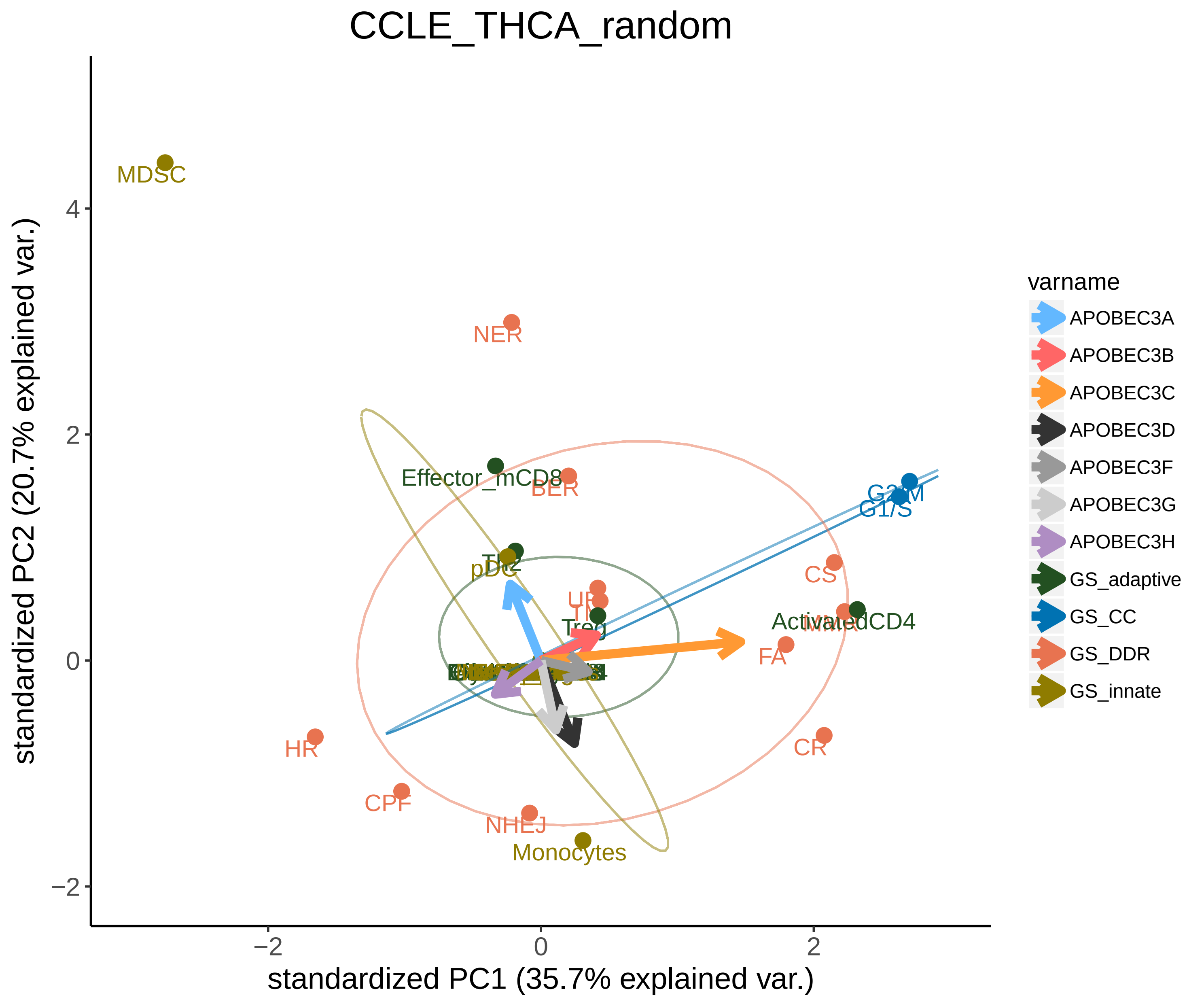

CCLE_THCA_random
standardized PC1 (35.7% explained var.)
standardized PC2 (20.7% explained var.)
varname
APOBEC3A
APOBEC3B
APOBEC3C
APOBEC3D
APOBEC3F
APOBEC3G
APOBEC3H
GS_adaptive
GS_CC
GS_DDR
GS_innate
MDSC
NER
Effector_mCD8
BER
G2/M
G1/S
pDC
CS
Treg
ActivatedCD4
FA
HR
CR
CPF
NHEJ
Monocytes

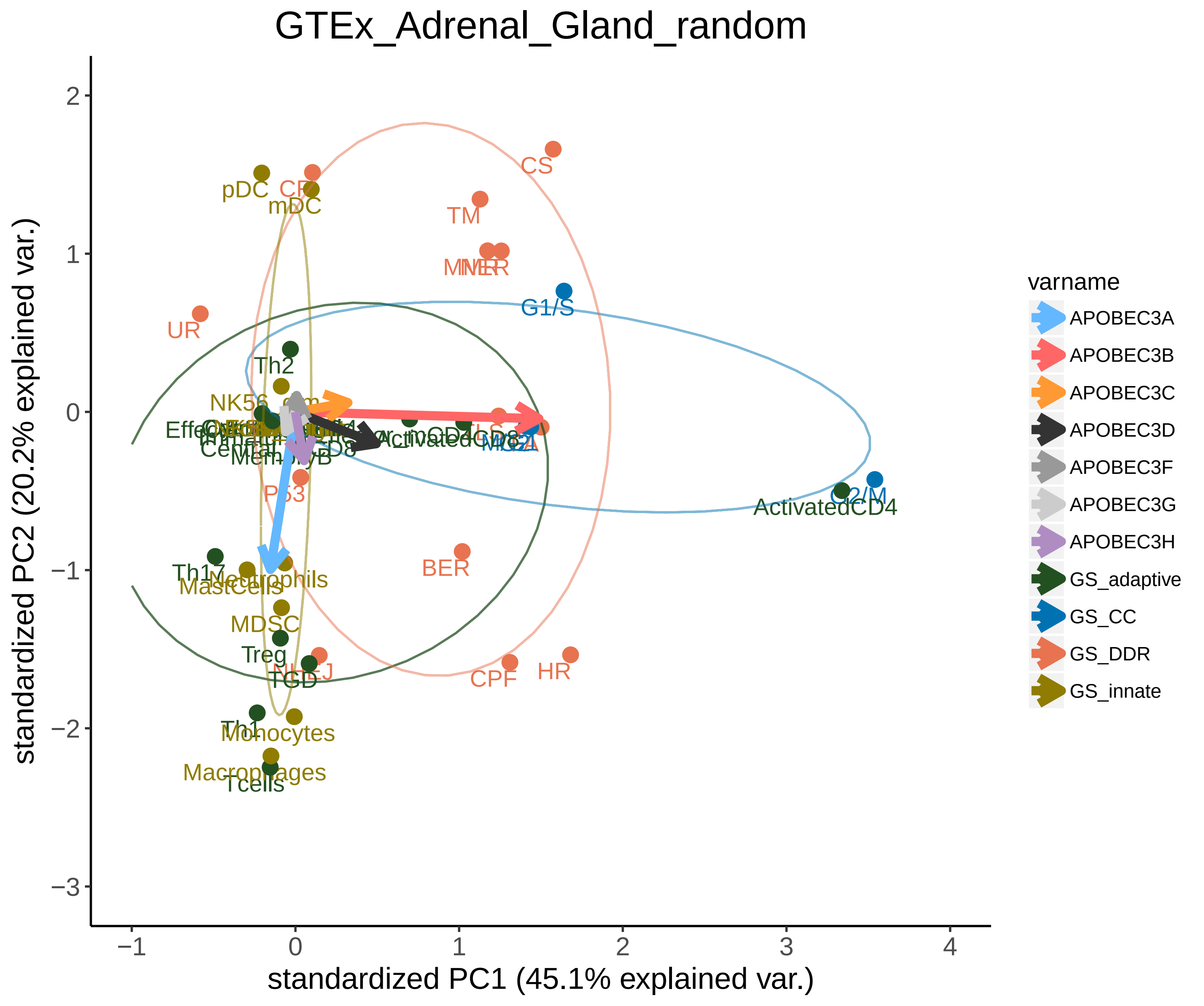
GTEx_Adrenal_Gland_random
standardized PC2 (20.2% explained var.)
standardized PC1 (45.1% explained var.)
2
1
0
−1
−2
−3
−1
0
1
2
3
4
CS
pDC
mDC
TM
MMR
UR
G1/S
Th2
NK56
P53
G2/M
ActivatedCD4
BER
Th17
Neutrophils
MastCells
MDSC
Treg
TGD
CPF
HR
Th1
Monocytes
Macrophages
Tcells
varname
APOBEC3A
APOBEC3B
APOBEC3C
APOBEC3D
APOBEC3F
APOBEC3G
APOBEC3H
GS_adaptive
GS_CC
GS_DDR
GS_innate

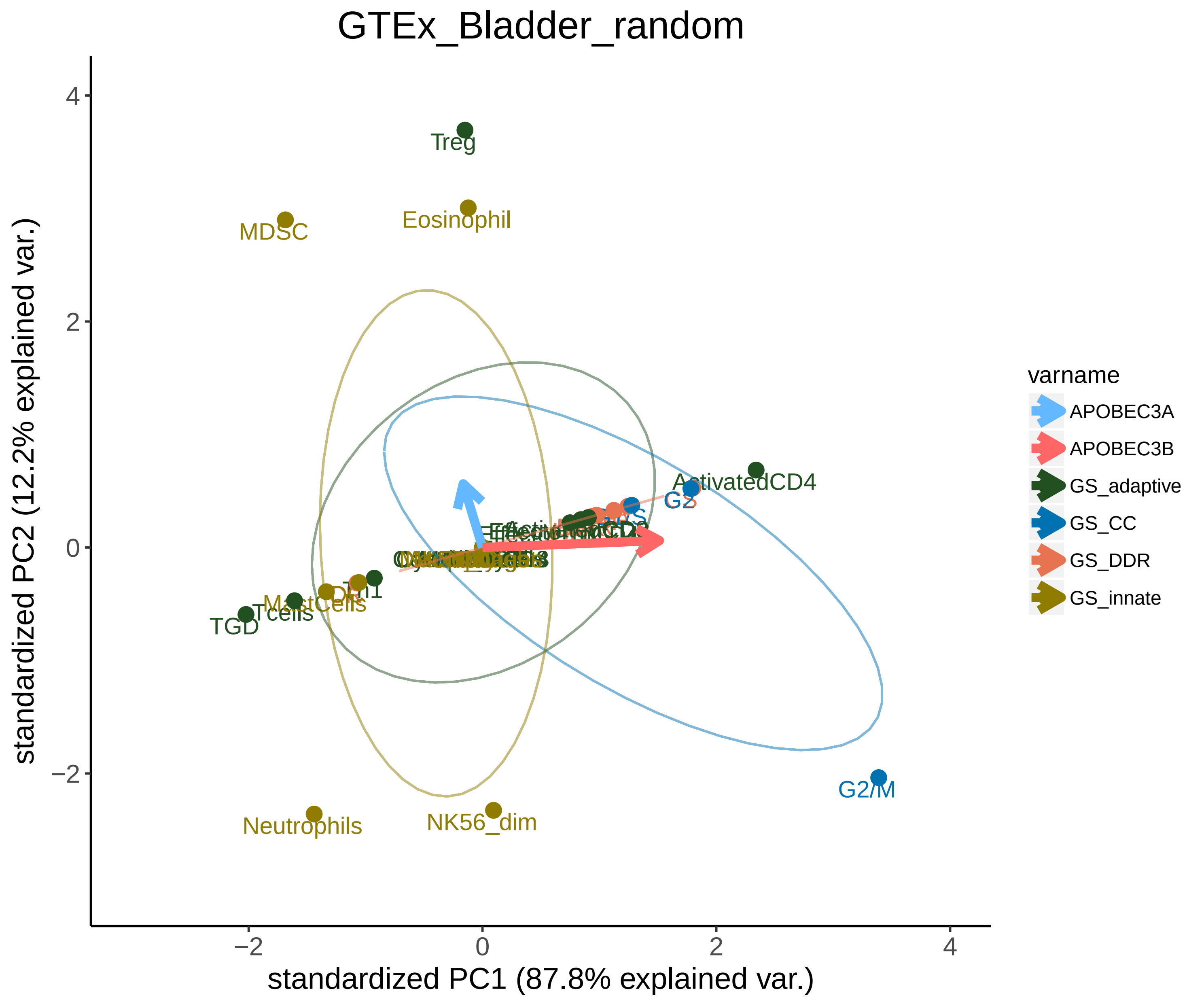

GTEx_Bladder_random
standardized PC1 (87.8% explained var.)
standardized PC2 (12.2% explained var.)
Treg
Eosinophil
MDSC
ActivatedCD4
G2
G2/M
TGD
Tcells
MastCells
Neutrophils
NK56_dim
varname
APOBEC3A
APOBEC3B
GS_adaptive
GS_CC
GS_DDR
GS_innate

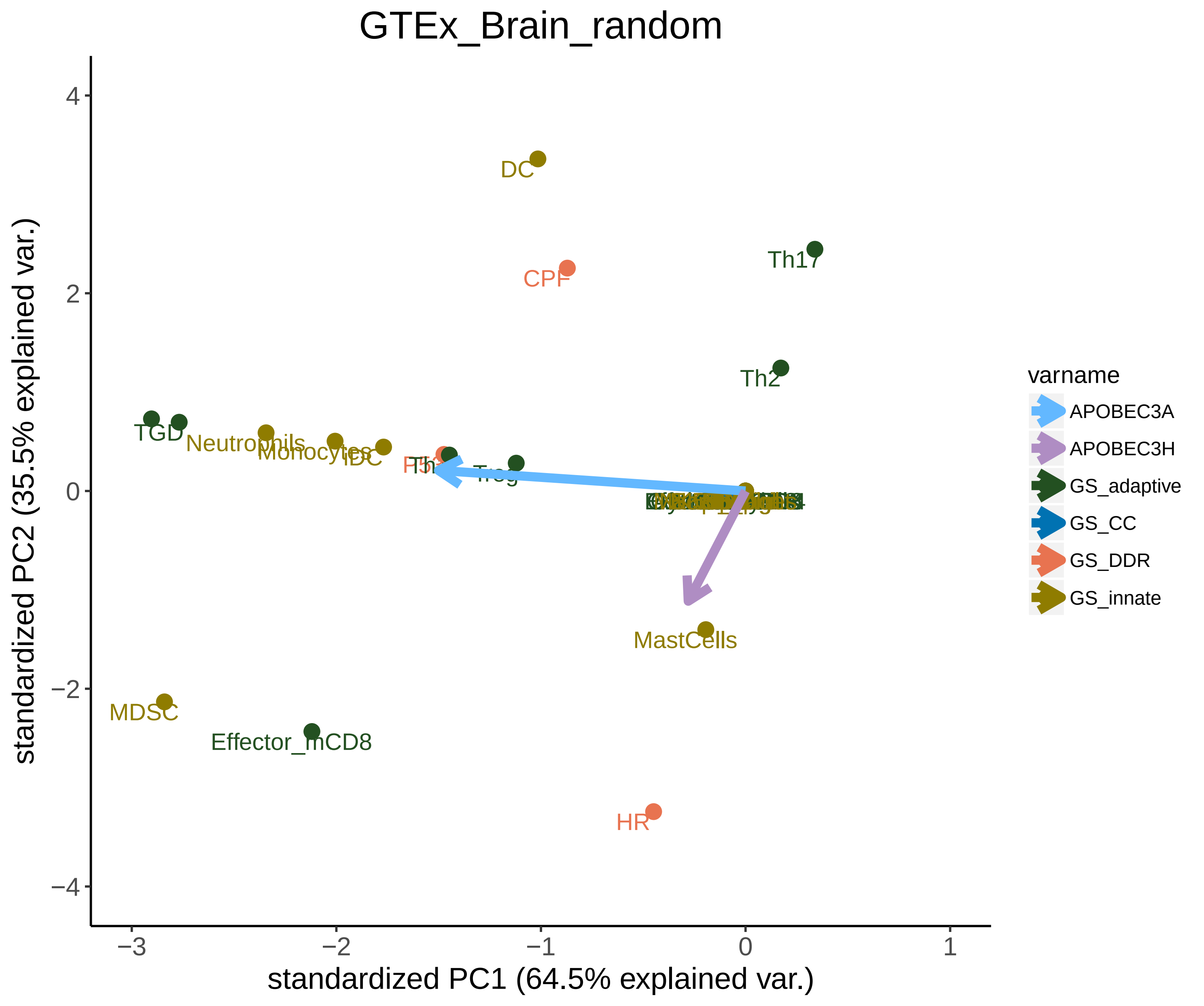
GTEx_Brain_random
DC
Th17
CPF
Th2
TGD
Neutrophils
Monocytes
IDC
Treg
MastCells
MDSC
Effector_mCD8
HR
standardized PC1 (64.5% explained var.)
standardized PC2 (35.5% explained var.)
−3
−2
−1
0
1
4
2
0
−2
−4
varname
APOBEC3A
APOBEC3H
GS_adaptive
GS_CC
GS_DDR
GS_innate

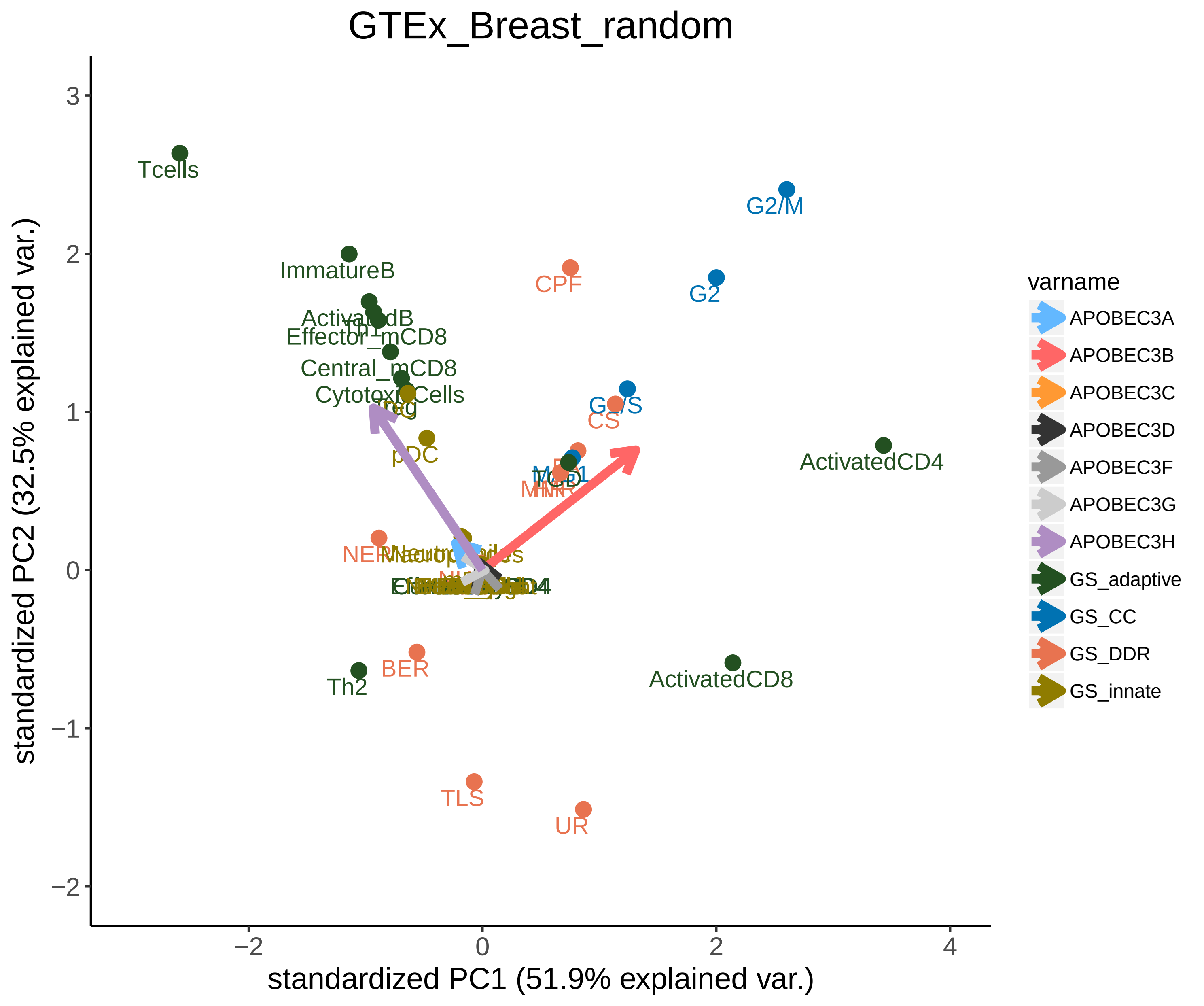

GTEx_Breast_random
Tcells
G2/M
ImmatureB
CPF
G2
ActivatedB
Th1
Effector_mCD8
Central_mCD8
Cytotoxic Cells
Treg
G1/S
CS
pDC
ActivatedCD4
MMR
Neutrophils
Macrophages
BER
Th2
ActivatedCD8
TLS
UR
standardized PC2 (32.5% explained var.)
standardized PC1 (51.9% explained var.)
3
2
1
0
−1
−2
−2
0
2
4
varname
APOBEC3A
APOBEC3B
APOBEC3C
APOBEC3D
APOBEC3F
APOBEC3G
APOBEC3H
GS_adaptive
GS_CC
GS_DDR
GS_innate

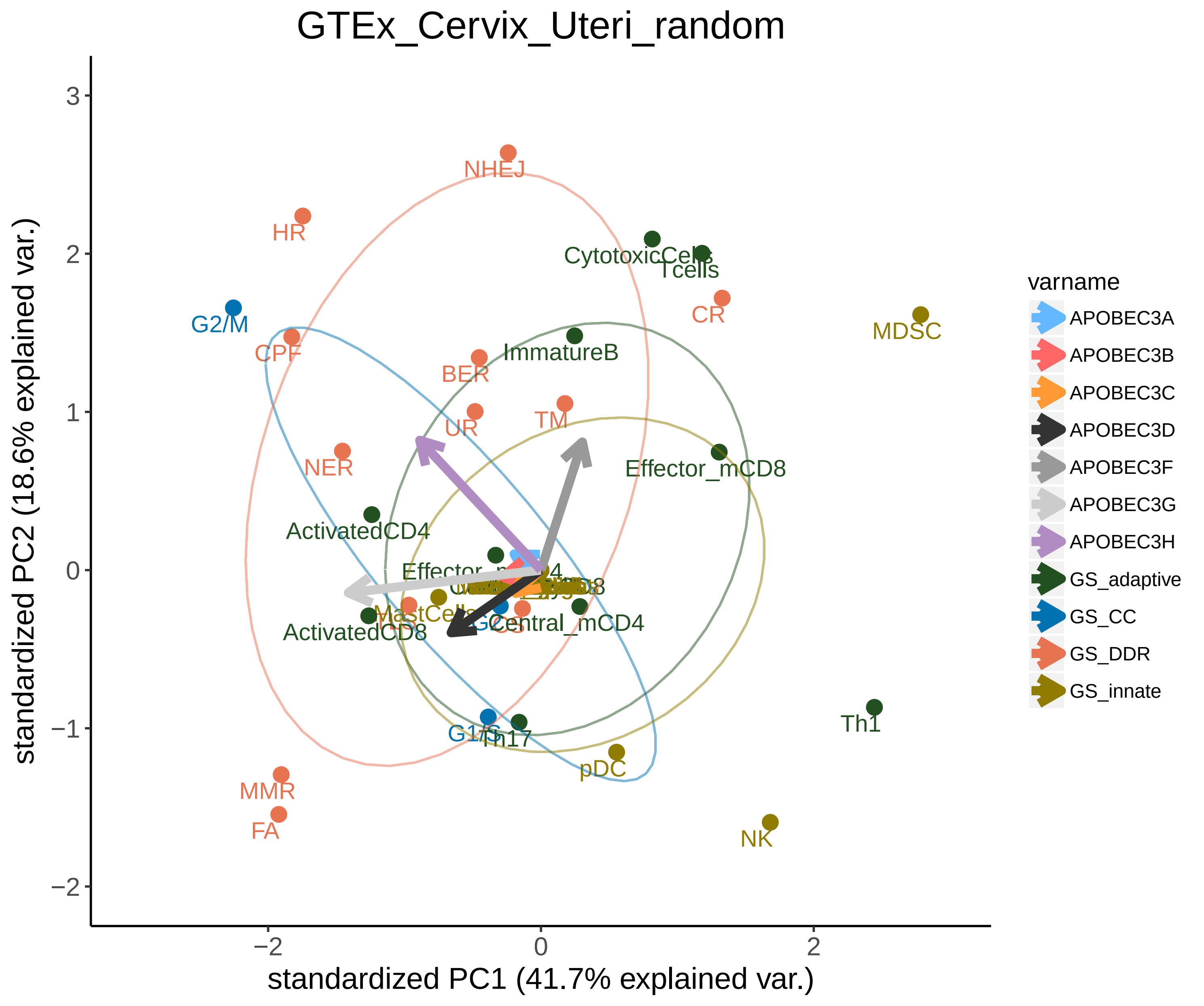
GTEx_Cervix_Uteri_random
standardized PC2 (18.6% explained var.)
standardized PC1 (41.7% explained var.)
3
2
1
0
−1
−2
−2
0
2
NHEJ
HR
G2/M
CPF
BER
UR
TM
NER
CytotoxicCells
Tcells
CR
MDSC
ImmatureB
Effector_mCD8
ActivatedCD4
ActivatedCD8
Central_mCD4
MastCells
G1/S
Th17
Th1
pDC
MMR
FA
NK
varname
APOBEC3A
APOBEC3B
APOBEC3C
APOBEC3D
APOBEC3F
APOBEC3G
APOBEC3H
GS_adaptive
GS_CC
GS_DDR
GS_innate

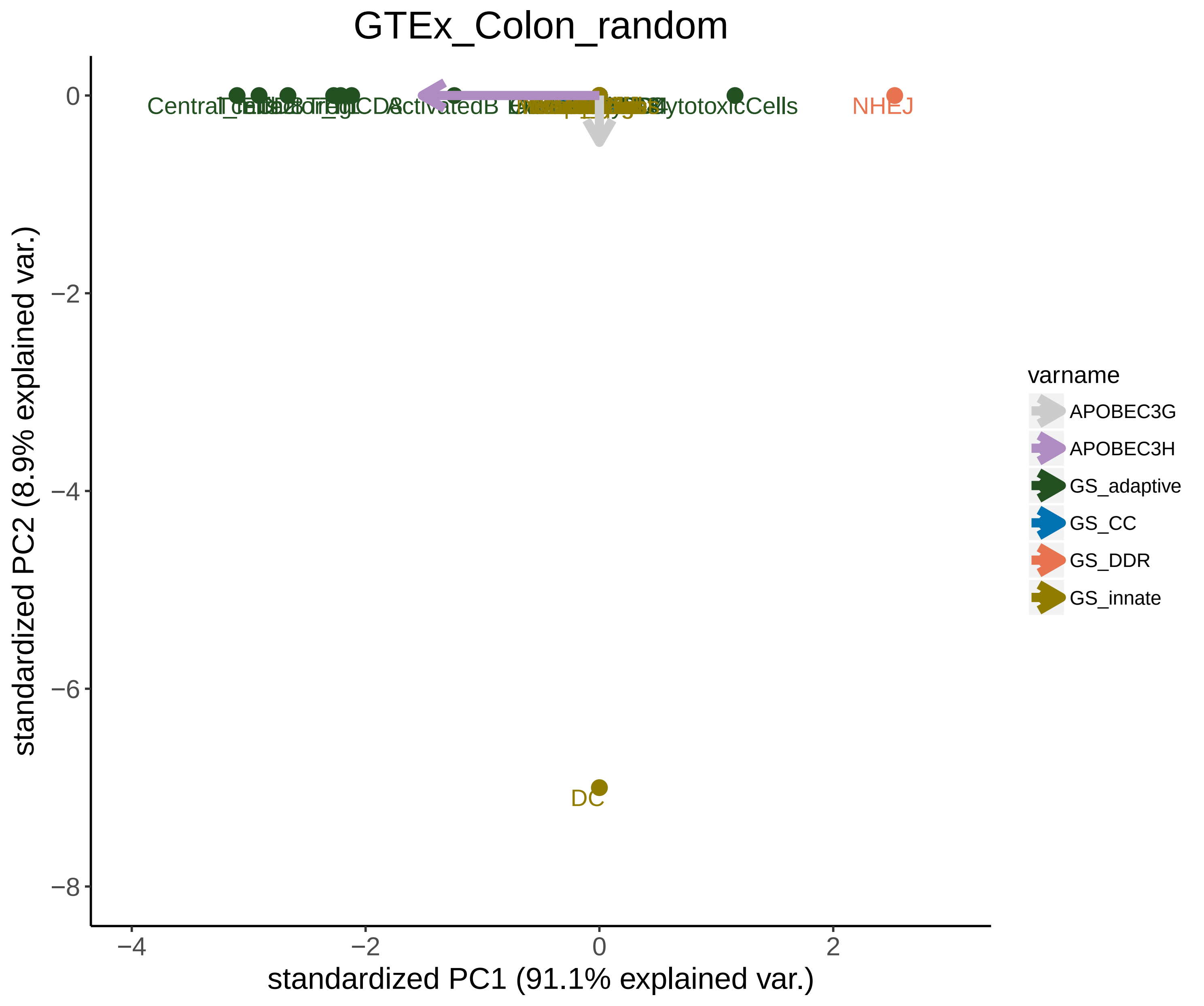
GTEx_Colon_random
standardized PC2 (8.9% explained var.)
standardized PC1 (91.1% explained var.)
0
−2
−4
−6
−8
−4
−2
0
2
CytotoxicCells
NHEJ
DC
varname
APOBEC3G
APOBEC3H
GS_adaptive
GS_CC
GS_DDR
GS_innate

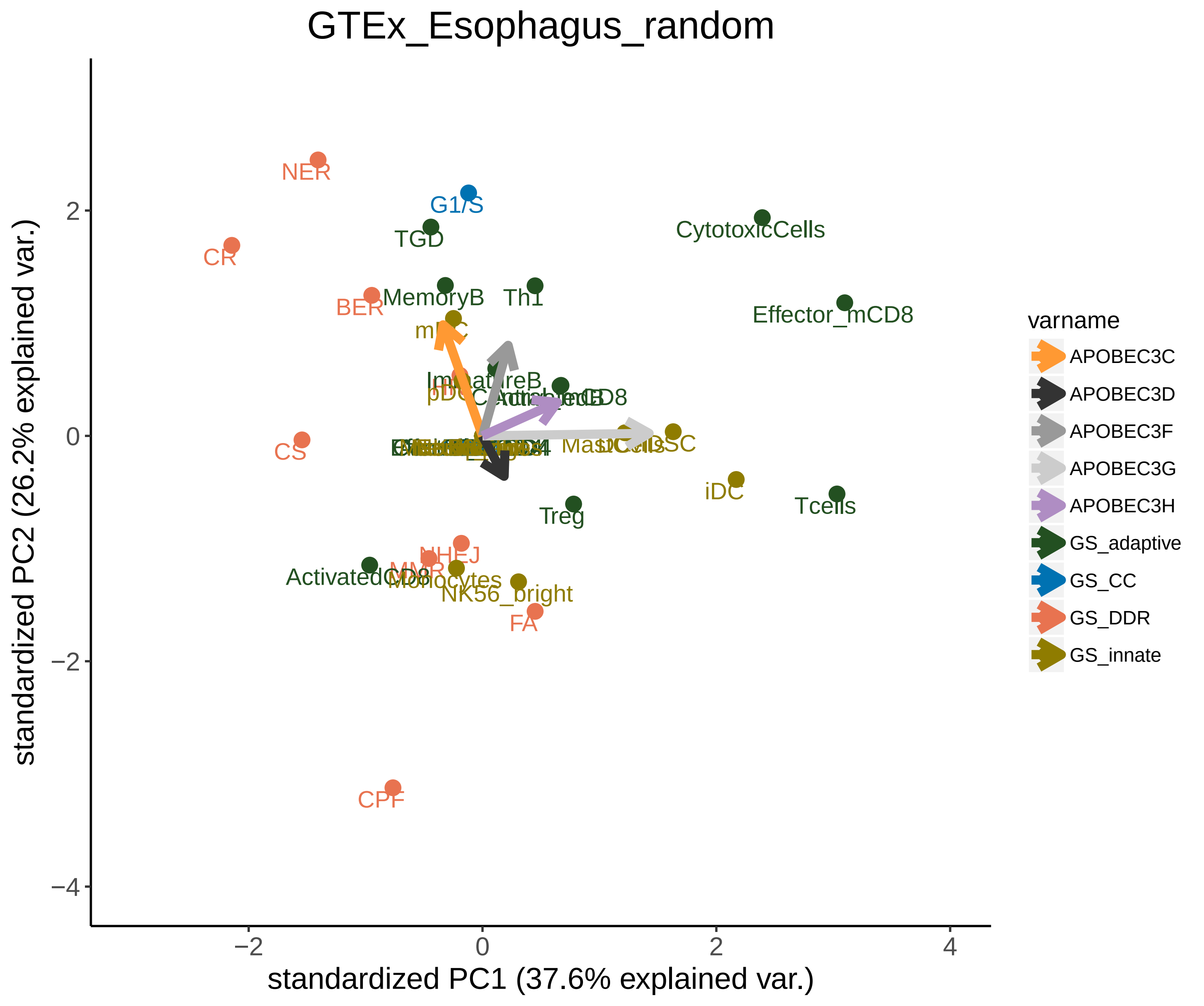

GTEx_Esophagus_random
standardized PC1 (37.6% explained var.)
standardized PC2 (26.2% explained var.)
−2
0
2
4
−4
varname
APOBEC3C
APOBEC3D
APOBEC3F
APOBEC3G
APOBEC3H
GS_adaptive
GS_CC
GS_DDR
GS_innate
NER
G1/S
TGD
CR
CytotoxicCells
MemoryB
Th1
BER
Effector_mCD8
CS
iDC
Tcells
Treg
NHEJ
MMR
ActivatedCD8
Monocytes
NK56_bright
FA
CPF

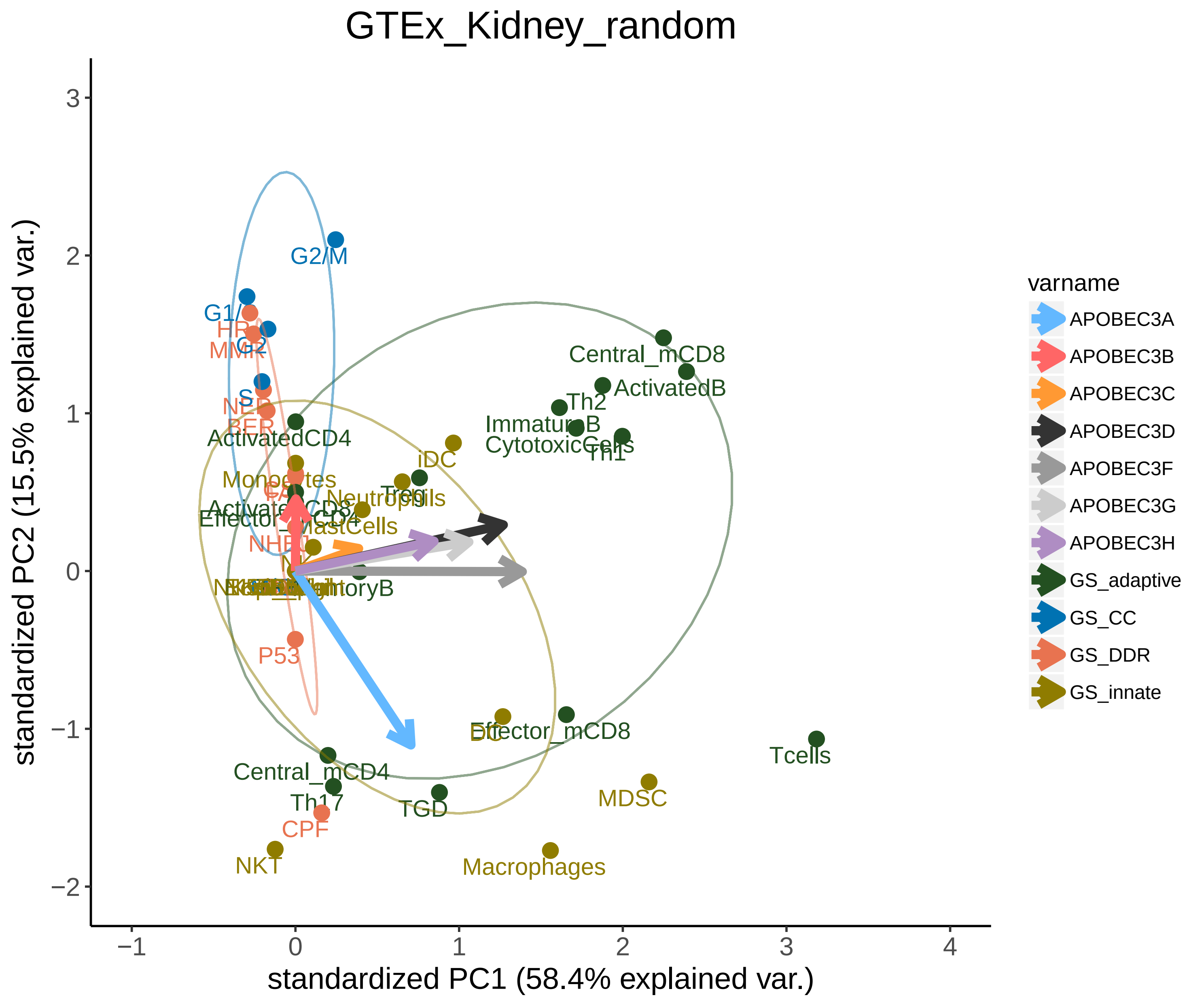
GTEx_Kidney_random
standardized PC2 (15.5% explained var.)
standardized PC1 (58.4% explained var.)
3
2
1
0
−1
−2
−1
0
1
2
3
4
G2/M
G1/S
HR
G2
MMR
S
NER
BER
ActivatedCD4
Monocytes
Central_mCD8
ActivatedB
Th2
ImmatureB
CytotoxicCells
Th1
iDC
Treg
Neutrophils
MastCells
NHEJ
P53
Effector_mCD8
DC
Tcells
Central_mCD4
Th17
CPF
TGD
MDSC
NKT
Macrophages
varname
APOBEC3A
APOBEC3B
APOBEC3C
APOBEC3D
APOBEC3F
APOBEC3G
APOBEC3H
GS_adaptive
GS_CC
GS_DDR
GS_innate

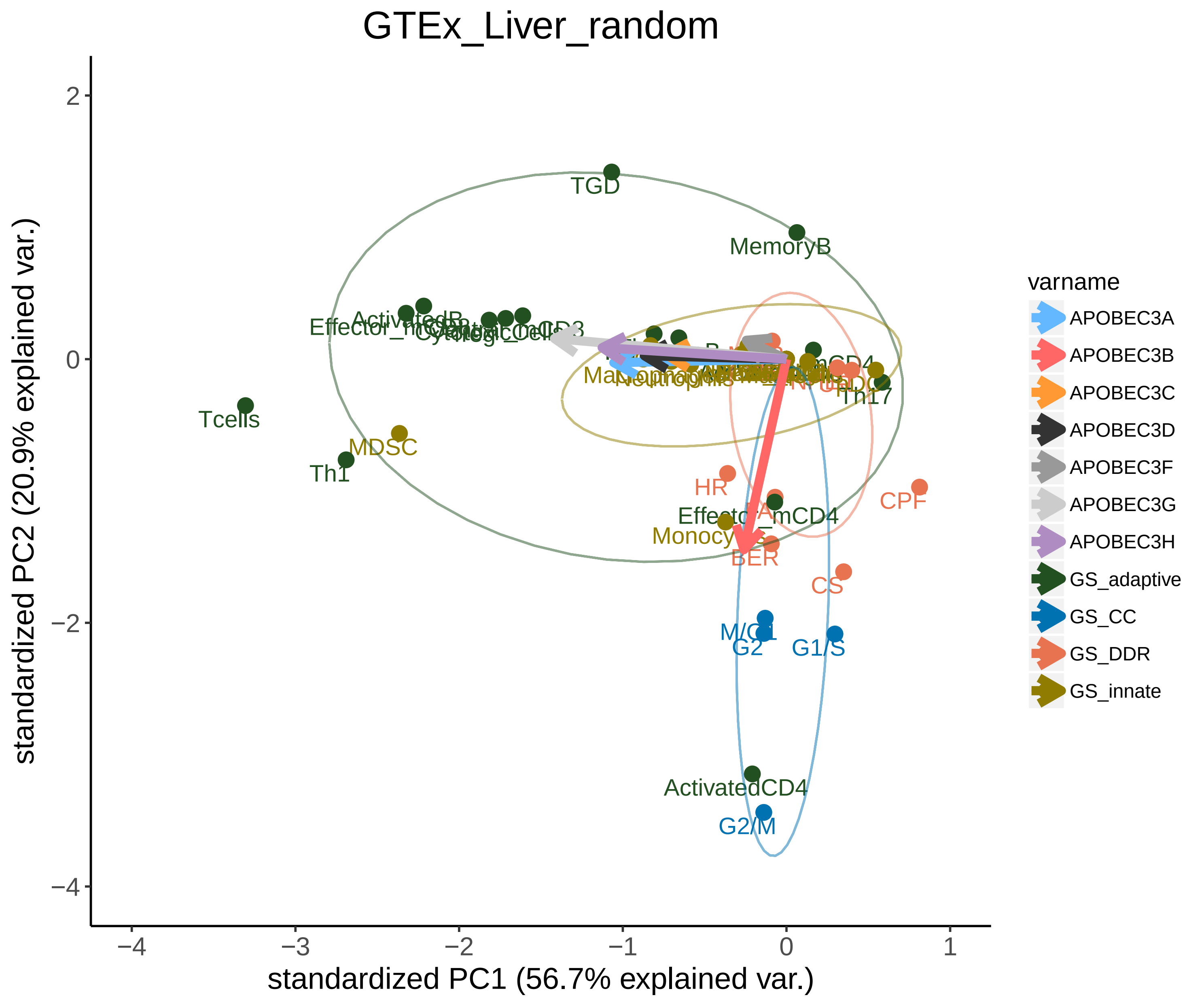

GTEx_Liver_random
standardized PC1 (56.7% explained var.)
standardized PC2 (20.9% explained var.)
TGD
MemoryB
Tcells
MDSC
Th1
HR
CPF
BER
CS
Th17
ActivatedCD4
M/G1
G2
G1/S
G2/M
varname
APOBEC3A
APOBEC3B
APOBEC3C
APOBEC3D
APOBEC3F
APOBEC3G
APOBEC3H
GS_adaptive
GS_CC
GS_DDR
GS_innate

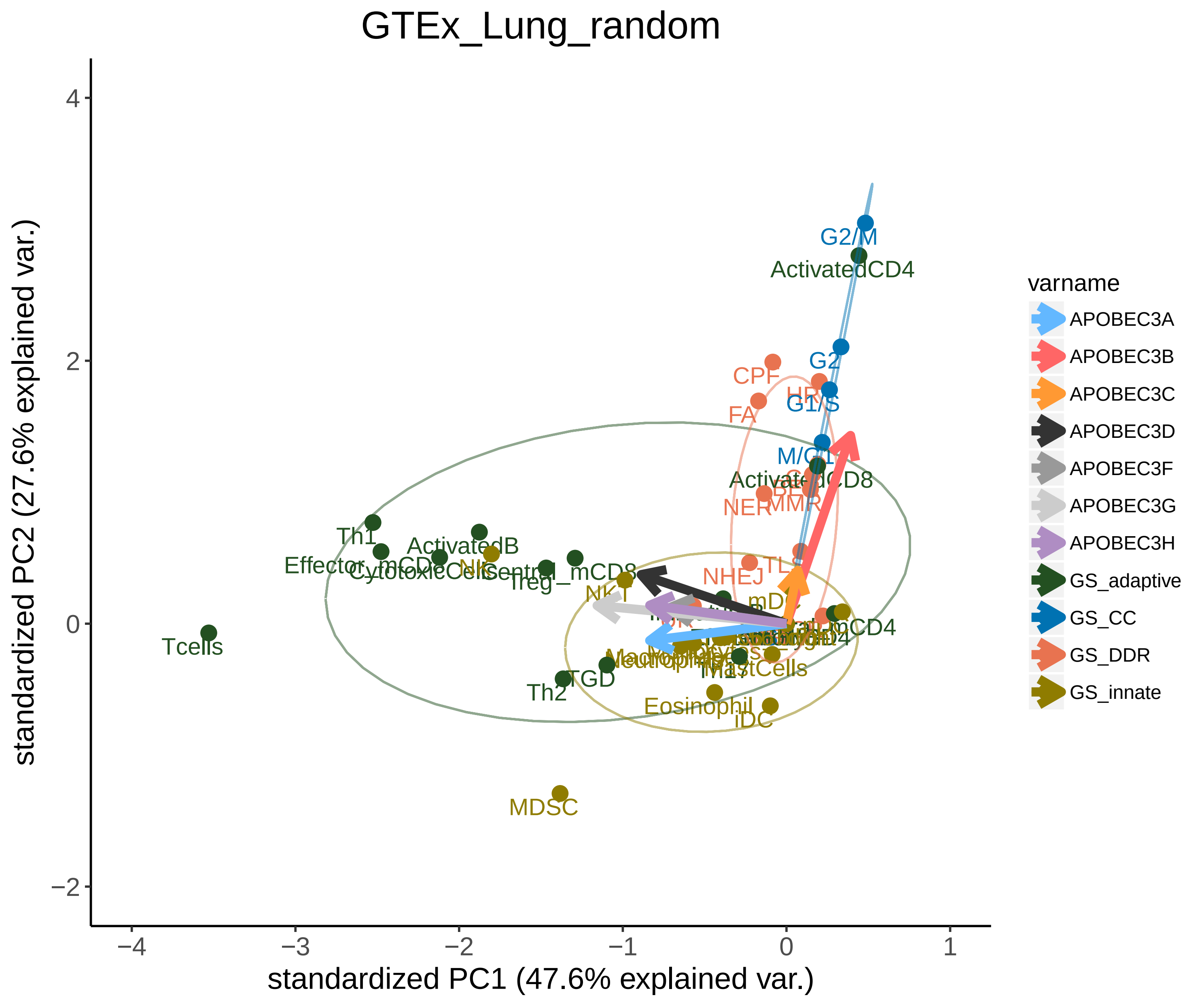
GTEx_Lung_random
standardized PC2 (27.6% explained var.)
standardized PC1 (47.6% explained var.)
4
2
0
−2
−4
−3
−2
−1
0
1
varname
APOBEC3A
APOBEC3B
APOBEC3C
APOBEC3D
APOBEC3F
APOBEC3G
APOBEC3H
GS_adaptive
GS_CC
GS_DDR
GS_innate
G2/M
ActivatedCD4
G2
CPF
G1/S
FA
M/G1
NER
MMR
Th1
ActivatedB
Tcells
NHEJ
NKT
TGD
Th2
Eosinophil
iDC
MastCells
MDSC

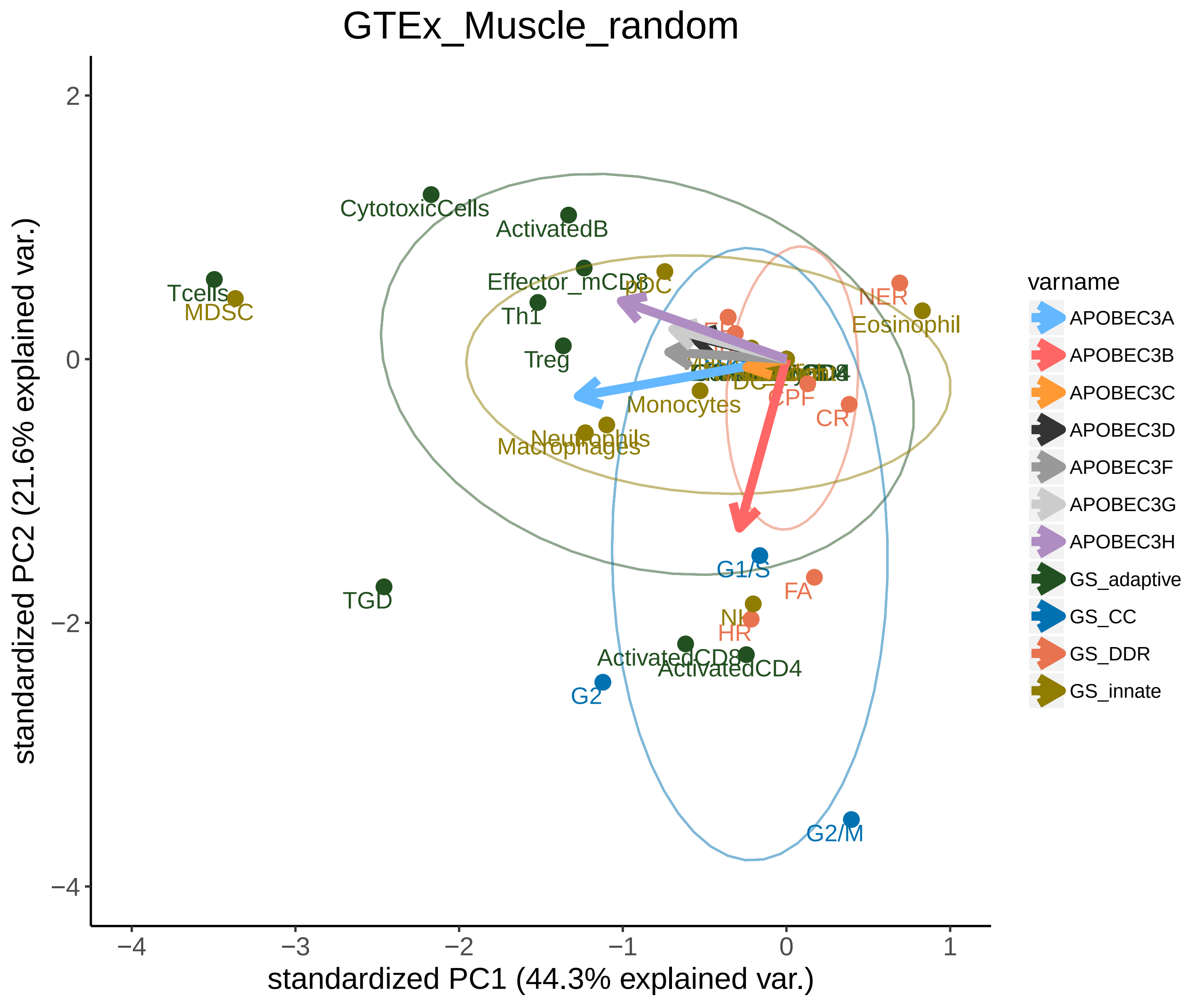

GTEx_Muscle_random
standardized PC1 (44.3% explained var.)
standardized PC2 (21.6% explained var.)
−4
−3
−2
−1
0
1
2
0
−2
−4
CytotoxicCells
ActivatedB
Effector_mCD8
pDC
Tcells
MDSC
Th1
Treg
NER
Eosinophil
Monocytes
CPF
CR
Neutrophils
Macrophages
G1/S
FA
TGD
HR
ActivatedCD8
ActivatedCD4
G2
G2/M
varname
APOBEC3A
APOBEC3B
APOBEC3C
APOBEC3D
APOBEC3F
APOBEC3G
APOBEC3H
GS_adaptive
GS_CC
GS_DDR
GS_innate

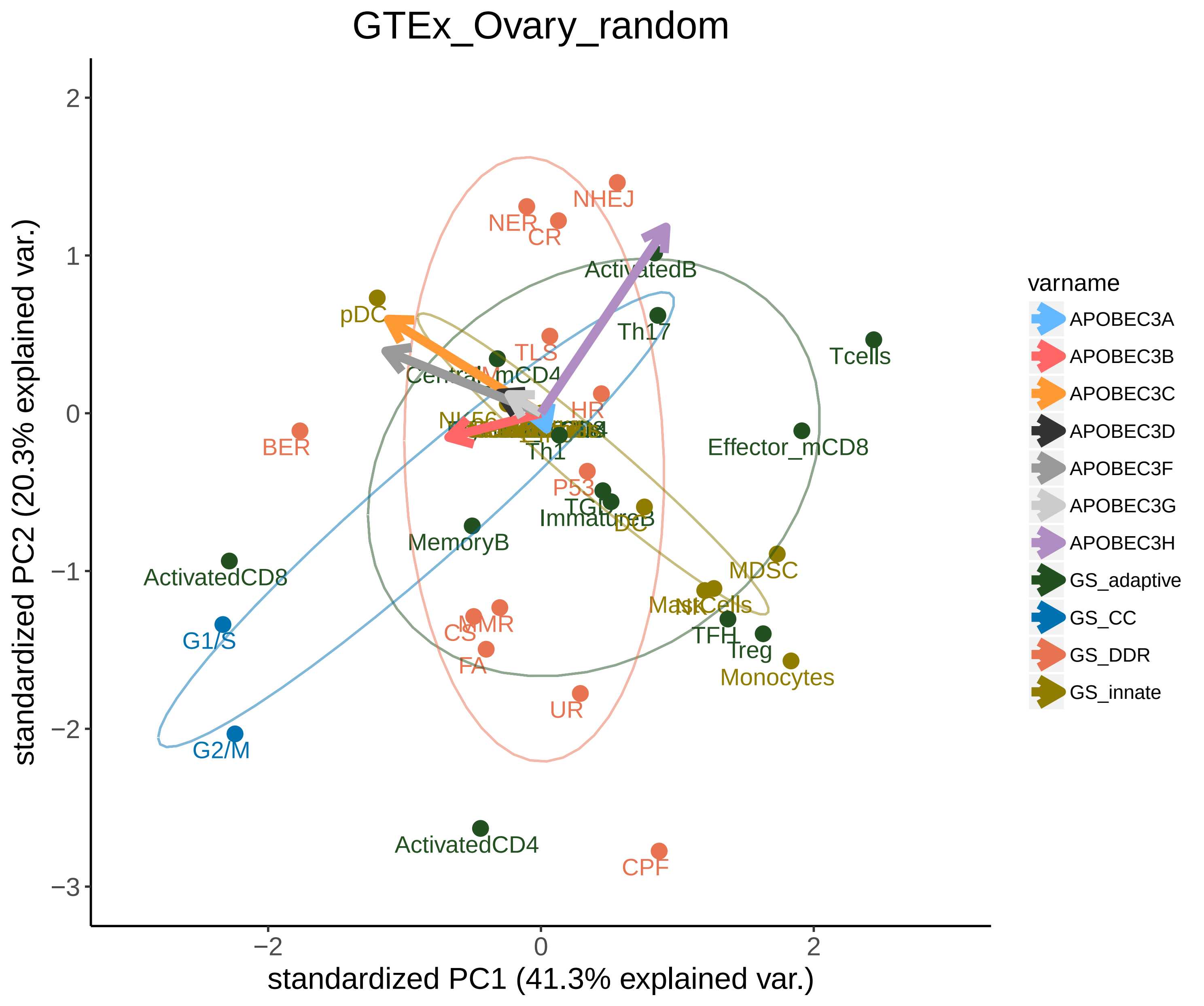
GTEx_Ovary_random
standardized PC2 (20.3% explained var.)
standardized PC1 (41.3% explained var.)
2
1
0
−1
−2
−3
−2
0
2
NHEJ
NER
CR
ActivatedB
pDC
Th17
Tcells
TLS
HR
BER
Th1
Effector_mCD8
P53
MemoryB
ActivatedCD8
MDSC
G1/S
MMR
CS
TFH
Treg
FA
Monocytes
UR
G2/M
ActivatedCD4
CPF
varname
APOBEC3A
APOBEC3B
APOBEC3C
APOBEC3D
APOBEC3F
APOBEC3G
APOBEC3H
GS_adaptive
GS_CC
GS_DDR
GS_innate

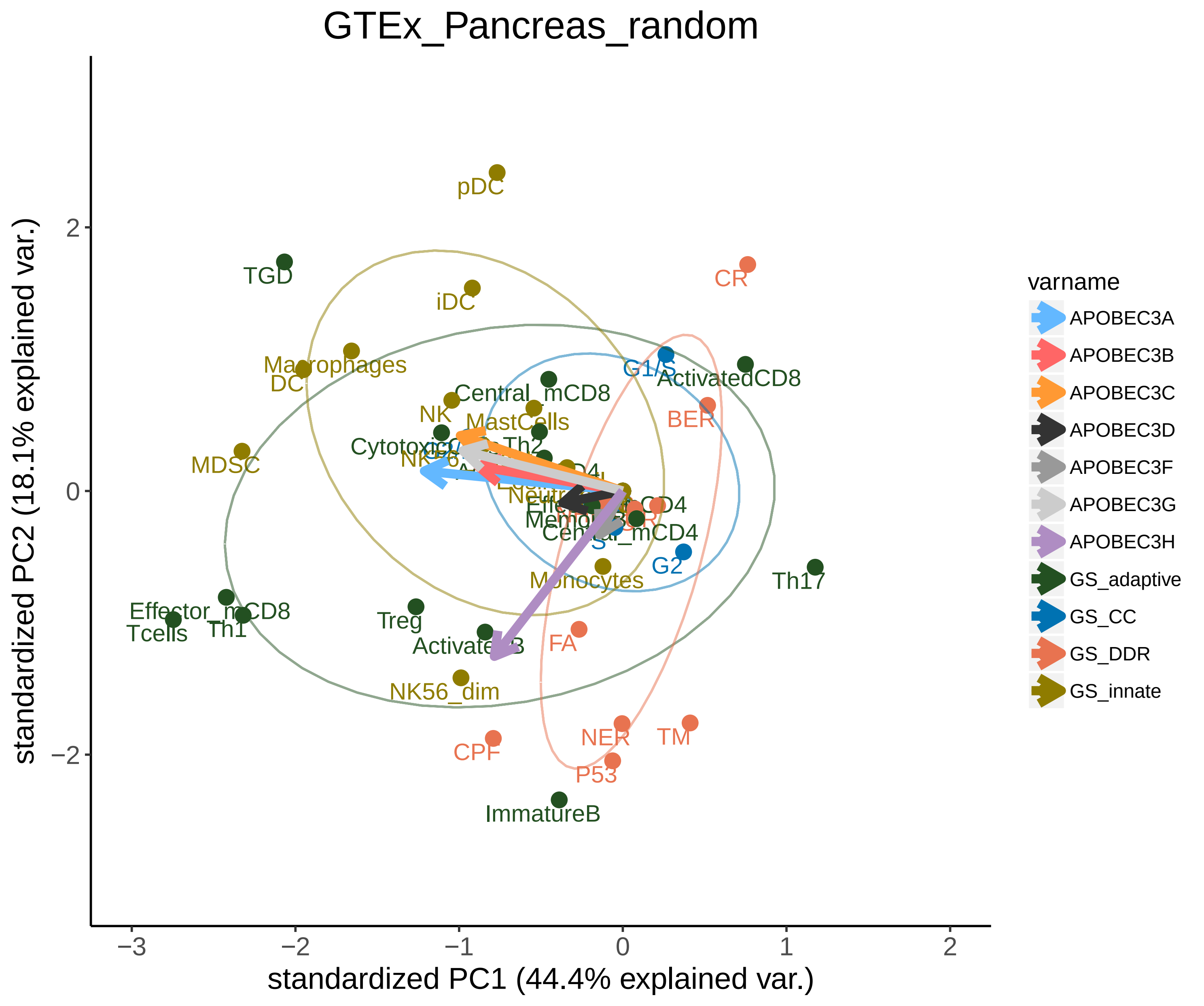
GTEx_Pancreas_random
standardized PC1 (44.4% explained var.)
standardized PC2 (18.1% explained var.)
−3
−2
−1
0
1
2
−2
0
2
varname
APOBEC3A
APOBEC3B
APOBEC3C
APOBEC3D
APOBEC3F
APOBEC3G
APOBEC3H
GS_adaptive
GS_CC
GS_DDR
GS_innate
pDC
TGD
CR
iDC
Macrophages
DC
G1/S
ActivatedCD8
Central_mCD8
NK
MastCells
BER
MDSC
Th2
Central_mCD4
G2
Monocytes
Th17
Effector_mCD8
Tcells
Th1
Treg
FA
NK56_dim
CPF
NER
TM
P53
ImmatureB

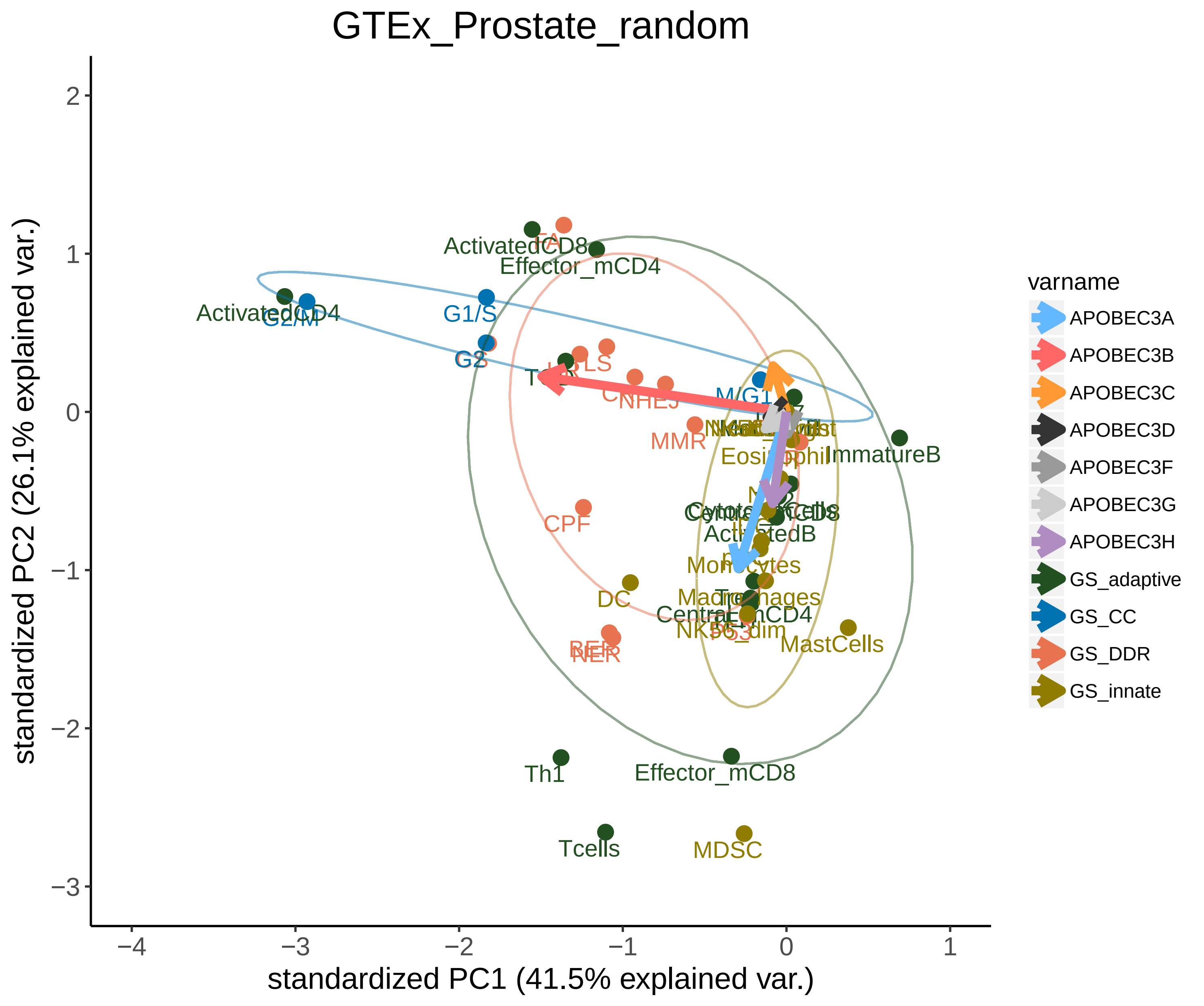
GTEx_Prostate_random
standardized PC2 (26.1% explained var.)
standardized PC1 (41.5% explained var.)
2
1
0
−1
−2
−3
−4
−3
−2
−1
0
1
ActivatedCD8
Effector_mCD4
ActivatedCD4
G2/M
G1/S
G2
MMR
CPF
ImmatureB
ActivatedB
Monocytes
DC
Macrophages
CentralmCD4
MastCells
Th1
Effector_mCD8
Tcells
MDSC
varname
APOBEC3A
APOBEC3B
APOBEC3C
APOBEC3D
APOBEC3F
APOBEC3G
APOBEC3H
GS_adaptive
GS_CC
GS_DDR
GS_innate

GTEx_Stomach_random

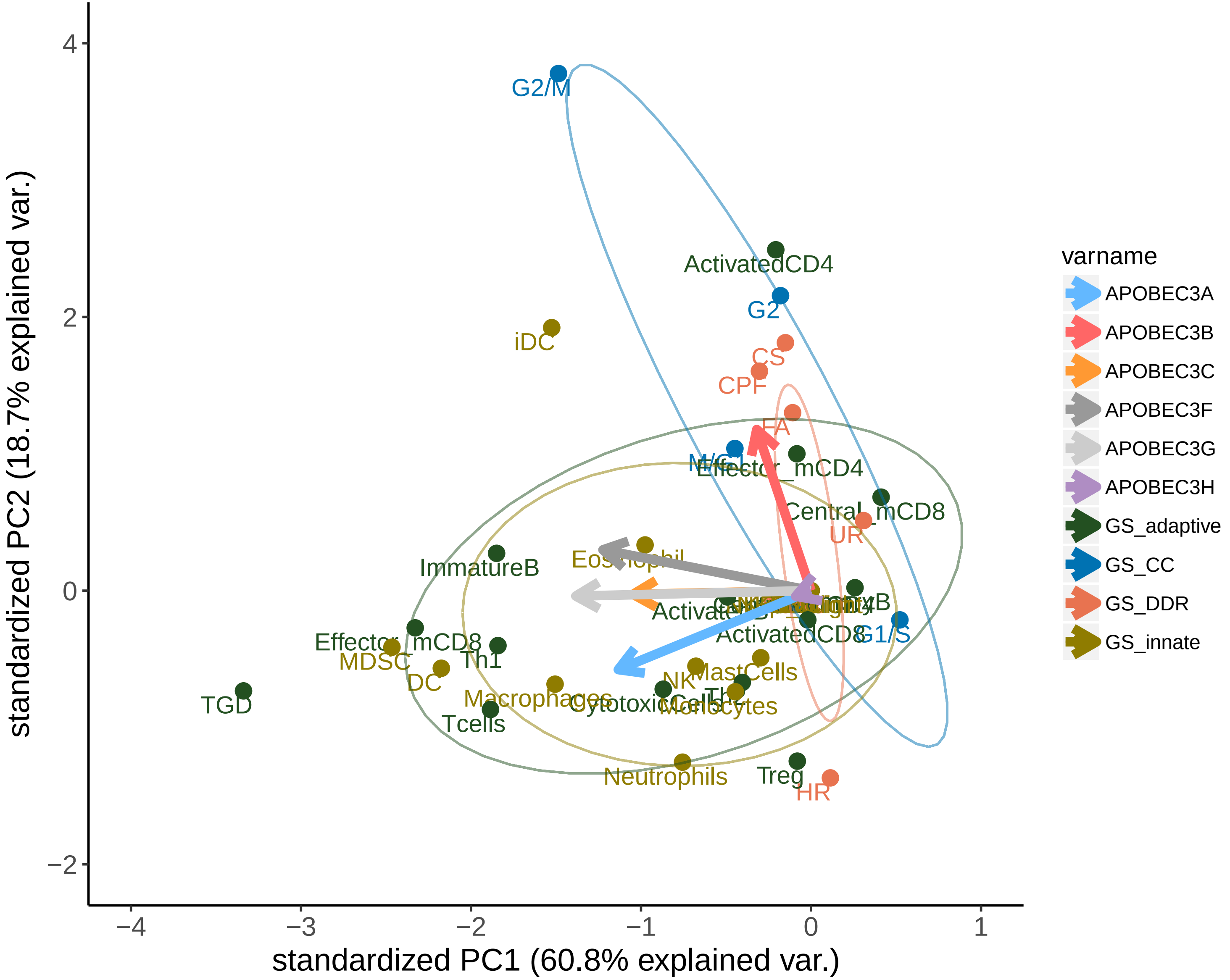

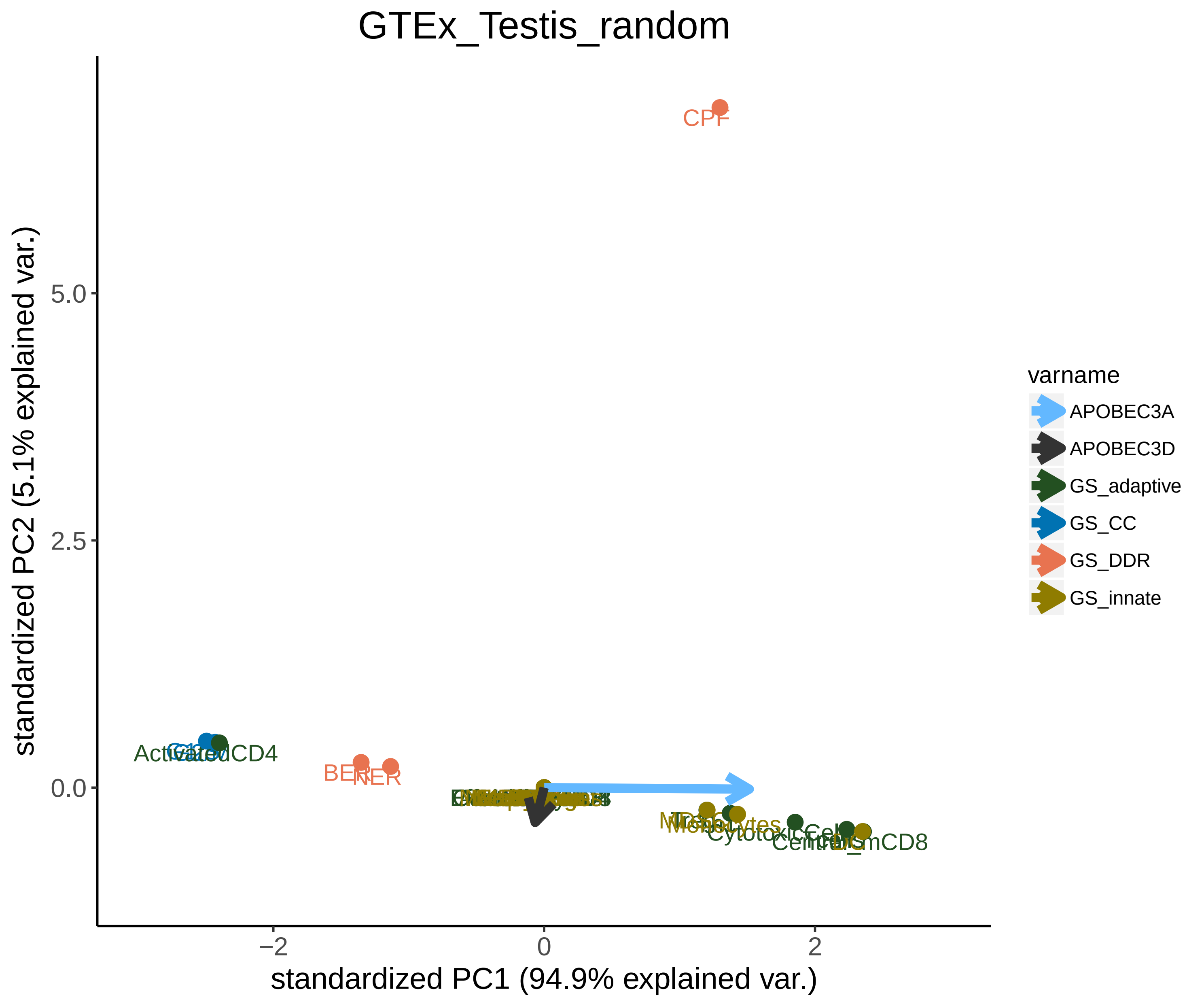
GTEx_Testis_random
CPF
BER
NER
standardized PC2 (5.1% explained var.)
5.0
2.5
0.0
−2
0
2
standardized PC1 (94.9% explained var.)
varname
APOBEC3A
APOBEC3D
GS_adaptive
GS_CC
GS_DDR
GS_innate

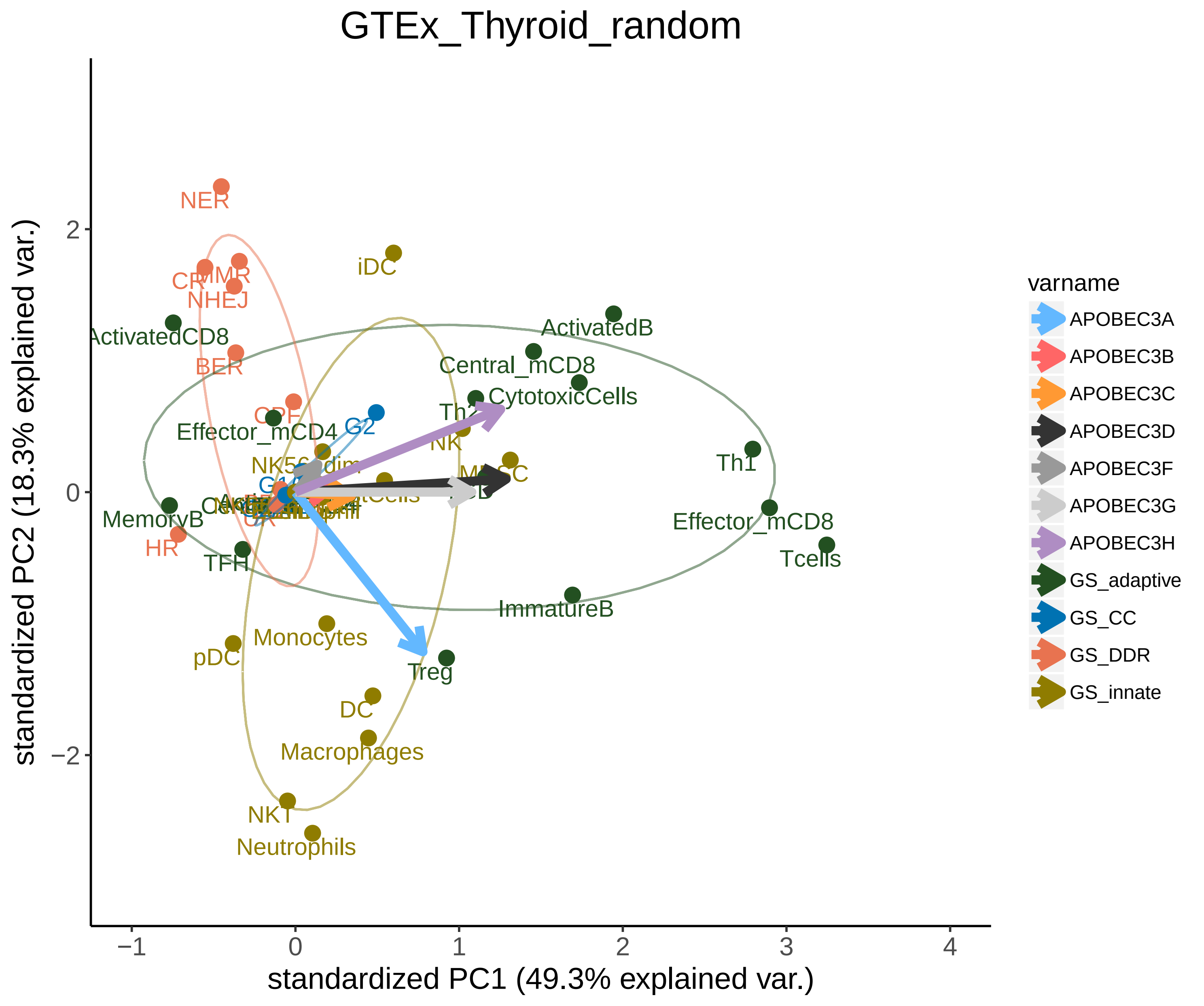
GTEx_Thyroid_random
standardized PC2 (18.3% explained var.)
standardized PC1 (49.3% explained var.)
2
0
−2
−1
0
1
2
3
4
NER
iDC
CR
MMR
NHEJ
ActivatedCD8
ActivatedB
Central_mCD8
BER
CytotoxicCells
Effector_mCD4
G2
NK
Th1
MemoryB
Effector_mCD8
HR
TFH
Tcells
ImmatureB
Monocytes
pDC
Treg
DC
Macrophages
NKT
Neutrophils
varname
APOBEC3A
APOBEC3B
APOBEC3C
APOBEC3D
APOBEC3F
APOBEC3G
APOBEC3H
GS_adaptive
GS_CC
GS_DDR
GS_innate

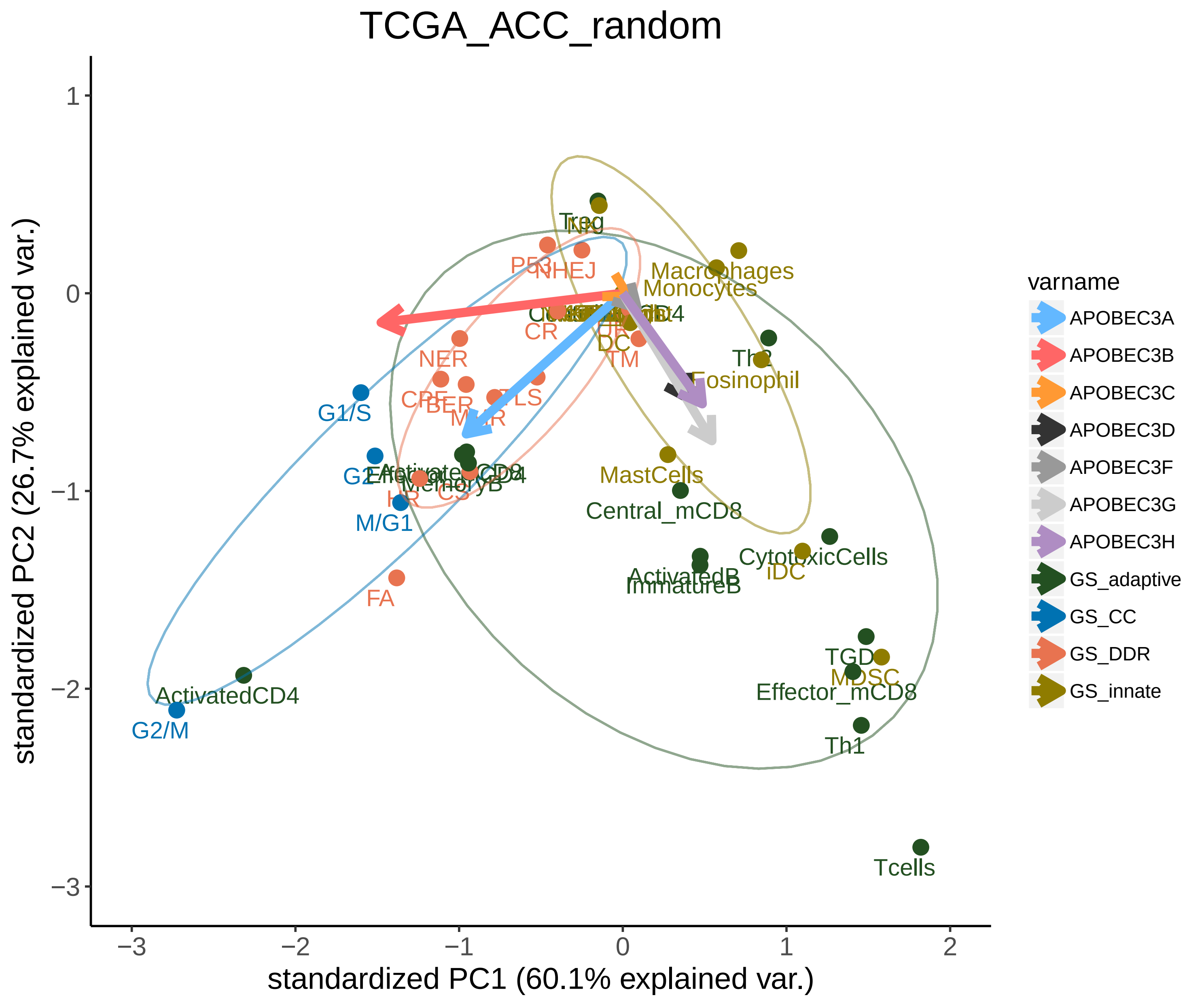
TCGA_ACC_random
standardized PC2 (26.7% explained var.)
standardized PC1 (60.1% explained var.)
varname
APOBEC3A
APOBEC3B
APOBEC3C
APOBEC3D
APOBEC3F
APOBEC3G
APOBEC3H
GS_adaptive
GS_CC
GS_DDR
GS_innate
Macrophages
Monocytes
NHEJ
CR
NER
Th2
Eosinophil
G1/S
MastCells
Central_mCD8
M/G1
CytotoxicCells
iDC
ImmatureB
FA
ActivatedCD4
G2/M
TGD
MDSC
Effector_mCD8
Th1
Tcells
TM
DC

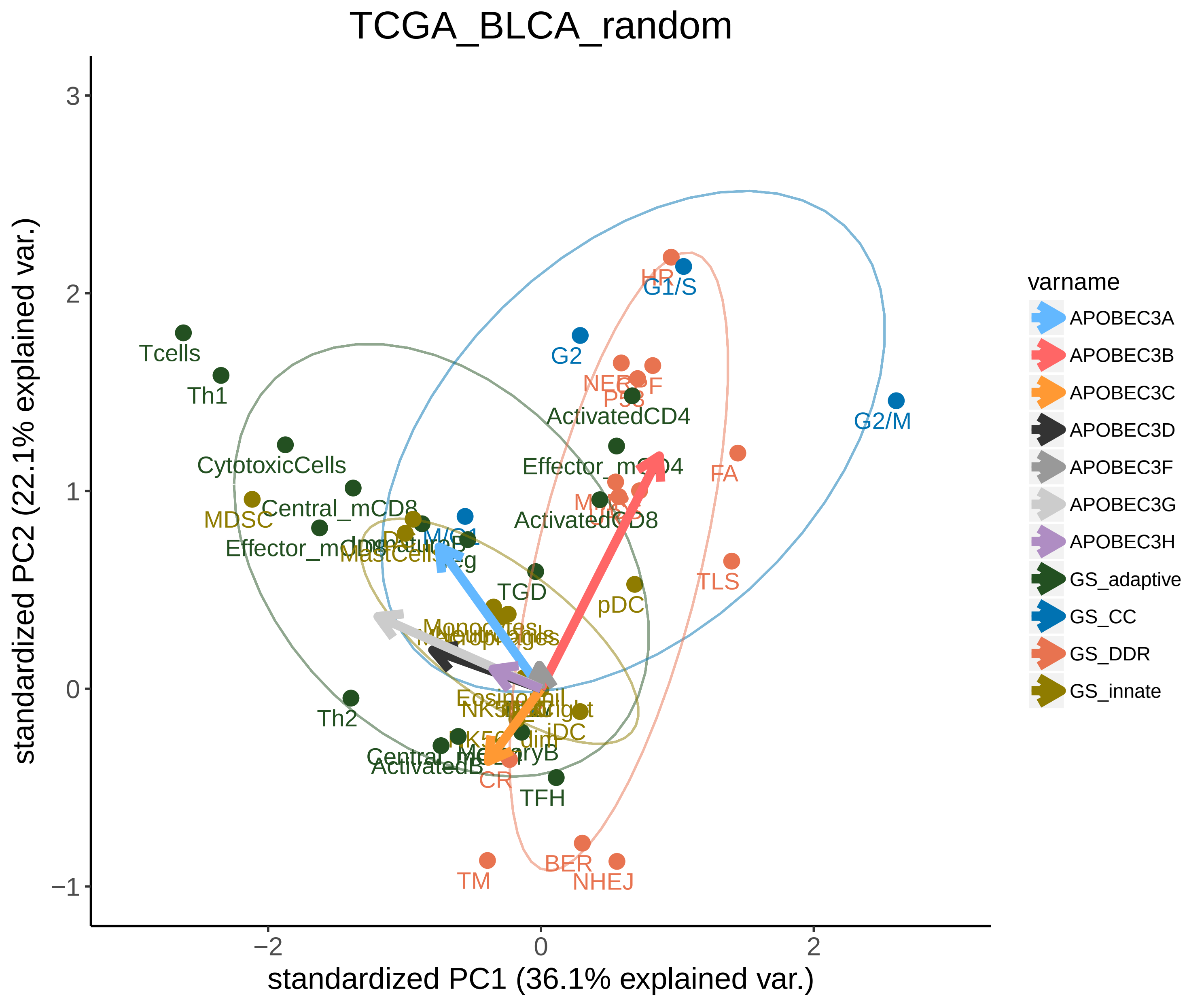

TCGA_BLCA_random
standardized PC2 (22.1% explained var.)
standardized PC1 (36.1% explained var.)
3
2
1
0
−1
−2
0
2
Tcells
Th1
CytotoxicCells
Central_mCD8
MDSC
Effector_mCD8
MastCells
TGD
pDC
Th2
ActivatedB
CR
TFH
TM
BER
NHEJ
TLS
FA
ActivatedCD8
Effector_mCD4
ActivatedCD4
G2
G1/S
HR
G2/M
iDC
Eosinophil
varname
APOBEC3A
APOBEC3B
APOBEC3C
APOBEC3D
APOBEC3F
APOBEC3G
APOBEC3H
GS_adaptive
GS_CC
GS_DDR
GS_innate

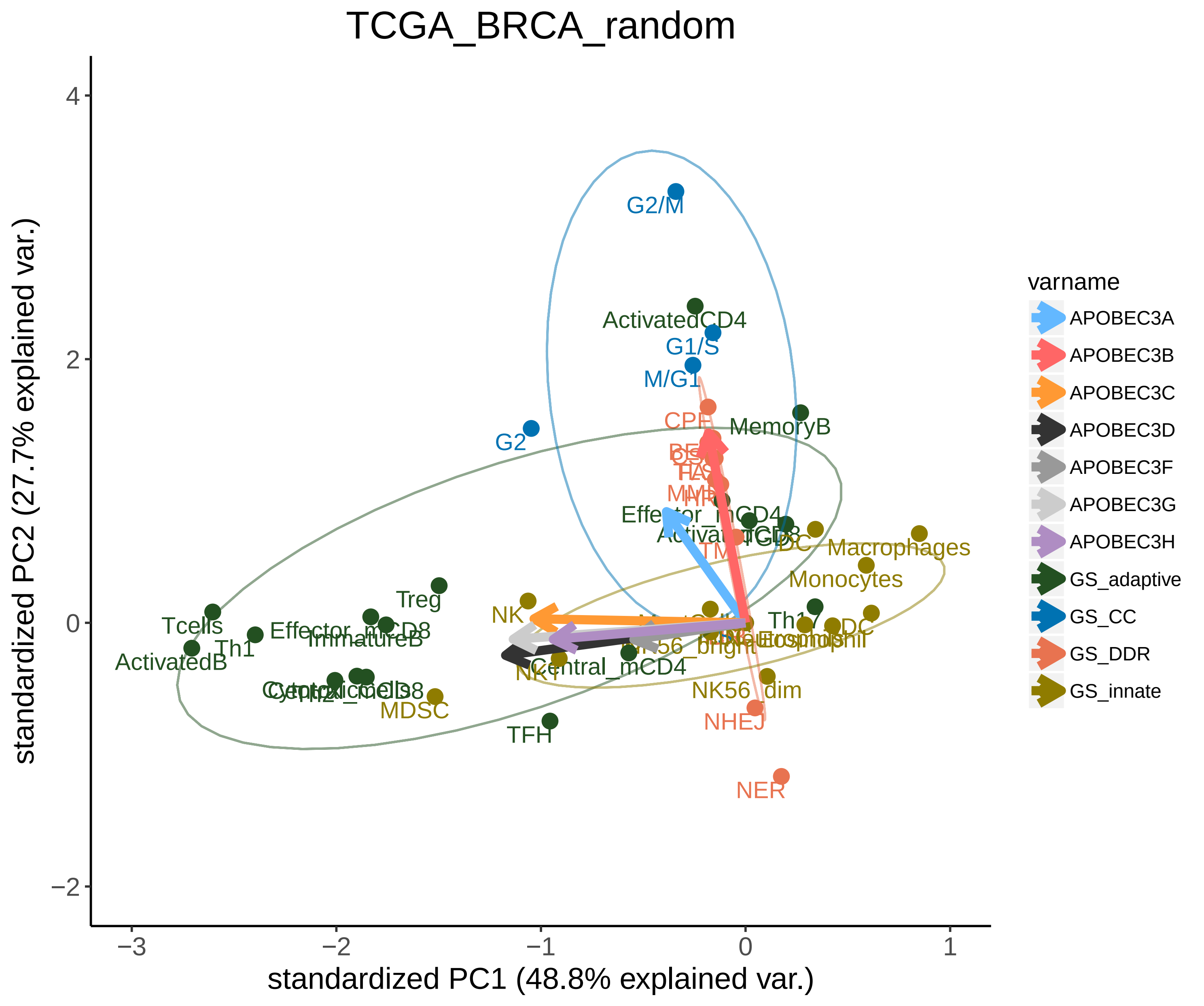

TCGA_BRCA_random
standardized PC2 (27.7% explained var.)
standardized PC1 (48.8% explained var.)
4
2
0
−2
−3
−2
−1
0
1
G2/M
ActivatedCD4
G1/S
M/G1
G2
CPF
MemoryB
MMR
Effector_mCD4
TM
Macrophages
Monocytes
Treg
NK
Tcells
Th1
Effector_mCD8
ImmatureB
ActivatedB
Th17
Central_mCD4
NKT
NK56_dim
MDSC
NHEJ
TFH
NER
varname
APOBEC3A
APOBEC3B
APOBEC3C
APOBEC3D
APOBEC3F
APOBEC3G
APOBEC3H
GS_adaptive
GS_CC
GS_DDR
GS_innate

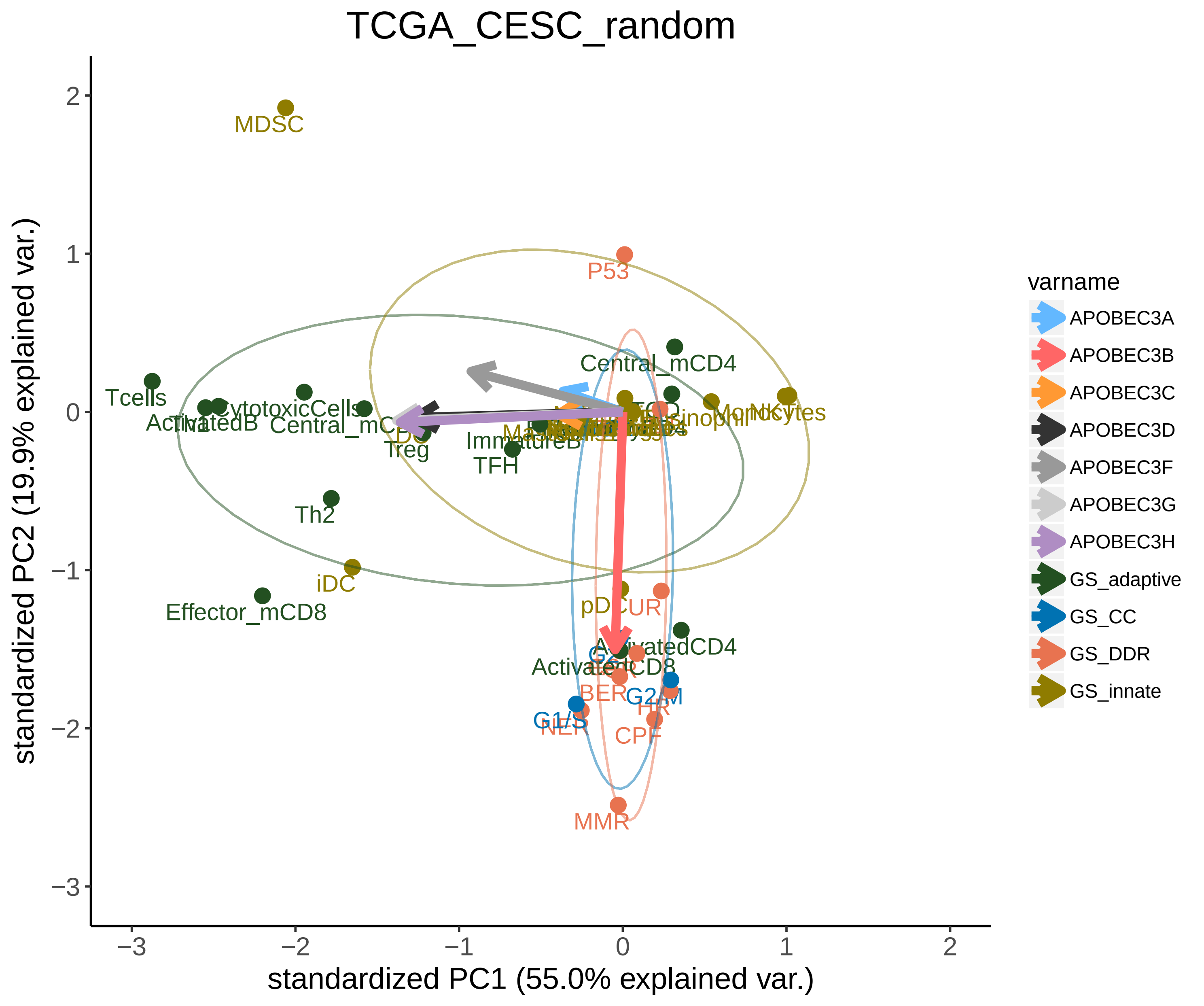

TCGA_CESC_random
standardized PC2 (19.9% explained var.)
standardized PC1 (55.0% explained var.)
2
1
0
−1
−2
−3
−3
−2
−1
0
1
2
MDSC
P53
Central_mCD4
Tcells
CytotoxicCells
ActivatedB
Central_mCD8
Treg
ImmatureB
TFH
Th2
iDC
Effector_mCD8
NKT
Monocytes
pDC
UR
ActivatedCD4
ActivatedCD8
BER
G2/M
HR
G1/S
NER
CPF
MMR
varname
APOBEC3A
APOBEC3B
APOBEC3C
APOBEC3D
APOBEC3F
APOBEC3G
APOBEC3H
GS_adaptive
GS_CC
GS_DDR
GS_innate

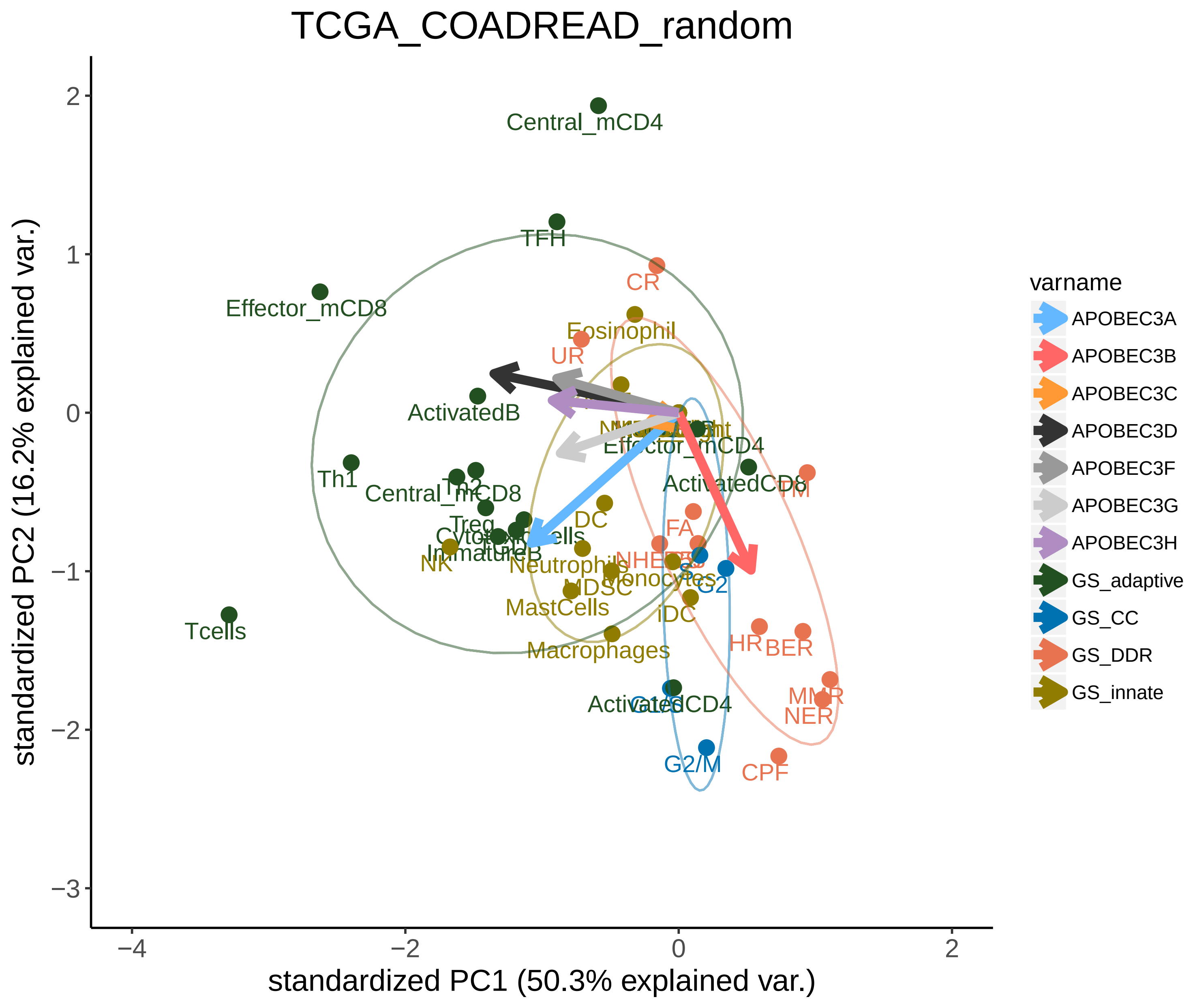
TCGA_COADREAD_random
standardized PC2 (16.2% explained var.)
standardized PC1 (50.3% explained var.)
2
1
0
−1
−2
−3
−4
−2
0
2
Central_mCD4
TFH
CR
Effector_mCD8
Eosinophil
UR
ActivatedB
Effector_mCD4
Th1
Central_mCD8
ActivatedCD8
TM
DC
FA
Treg
NK
Neutrophils
MDSC
MastCells
Monocytes
G2
iDC
Tcells
Macrophages
HR
BER
MMR
NER
G2/M
CPF
varname
APOBEC3A
APOBEC3B
APOBEC3C
APOBEC3D
APOBEC3F
APOBEC3G
APOBEC3H
GS_adaptive
GS_CC
GS_DDR
GS_innate

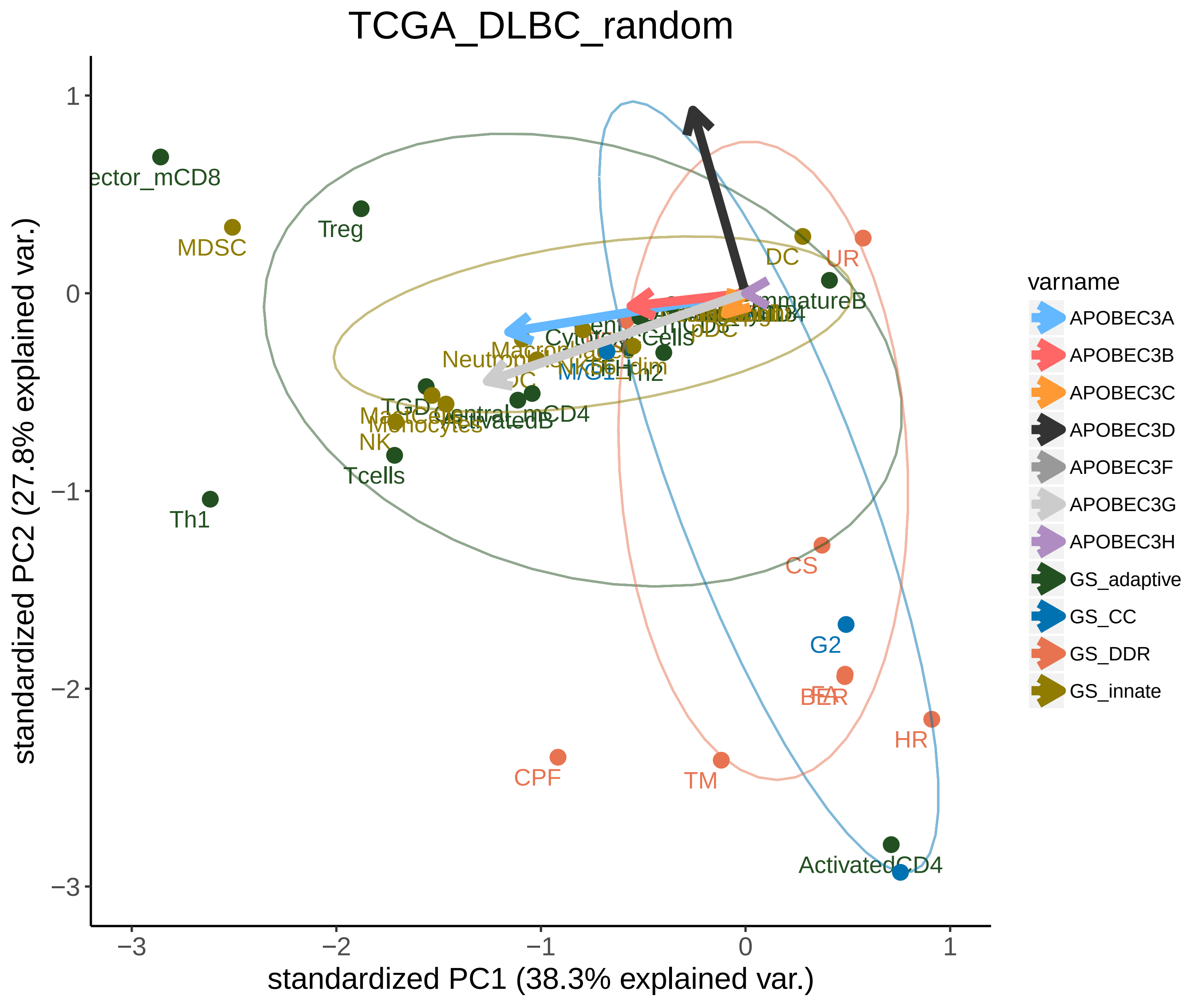

TCGA_DLBC_random
standardized PC1 (38.3% explained var.)
standardized PC2 (27.8% explained var.)
varname
APOBEC3A
APOBEC3B
APOBEC3C
APOBEC3D
APOBEC3F
APOBEC3G
APOBEC3H
GS_adaptive
GS_CC
GS_DDR
GS_innate
ector_mCD8
MDSC
Treg
DC
UR
Th1
Tcells
NK
CS
G2
HR
CPF
TM
ActivatedCD4

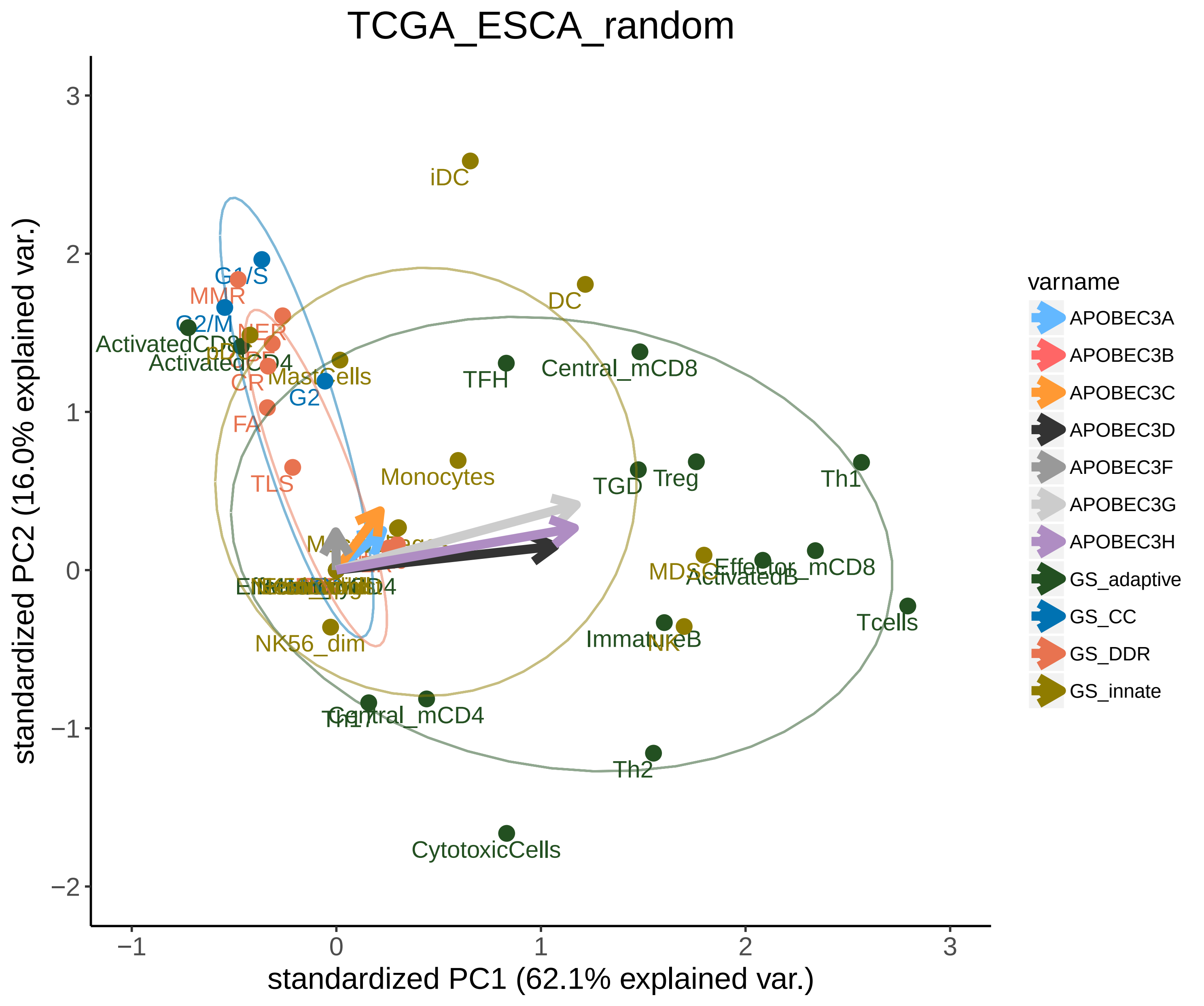
TCGA_ESCA_random
standardized PC2 (16.0% explained var.)
standardized PC1 (62.1% explained var.)
3
2
1
0
−1
−2
−1
0
1
2
3
varname
APOBEC3A
APOBEC3B
APOBEC3C
APOBEC3D
APOBEC3F
APOBEC3G
APOBEC3H
GS_adaptive
GS_CC
GS_DDR
GS_innate
iDC
DC
G1/S
MMR
G2/M
NER
ActivatedCD4
MastCells
CR
G2
FA
TFH
Central_mCD8
Monocytes
TLS
TGD
Treg
Th1
Effector_mCD8
ActivatedB
Tcells
ImmatureB
NK
NK56_dim
Central_mCD4
Th2
CytotoxicCells

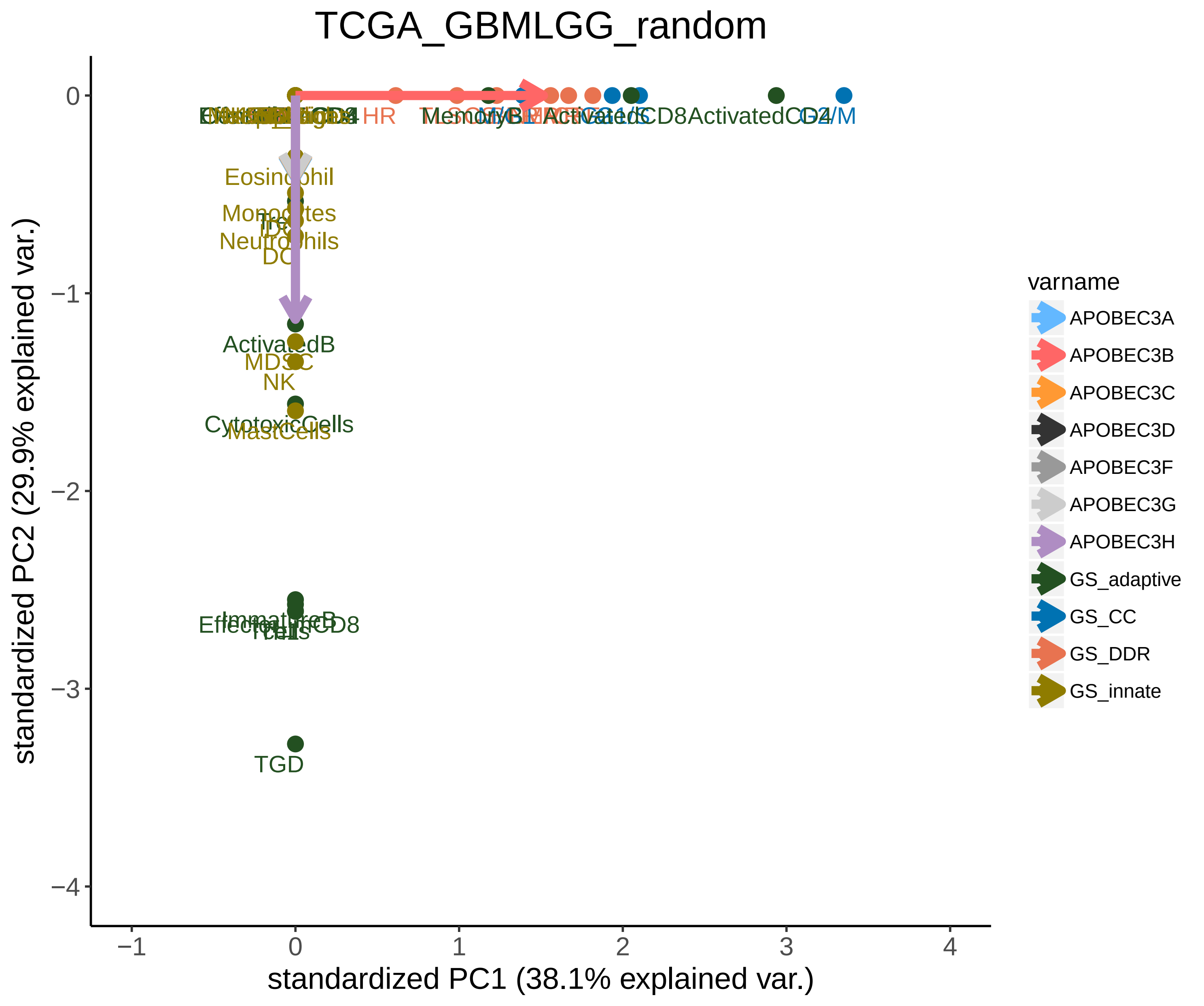

TCGA_GBMLGG_random
standardized PC1 (38.1% explained var.)
standardized PC2 (29.9% explained var.)
Eosinophil
Monocytes
Neutrophils
DC
ActivatedB
MDSC
NK
MastCells
TGD
G2/M
varname
APOBEC3A
APOBEC3B
APOBEC3C
APOBEC3D
APOBEC3F
APOBEC3G
APOBEC3H
GS_adaptive
GS_CC
GS_DDR
GS_innate

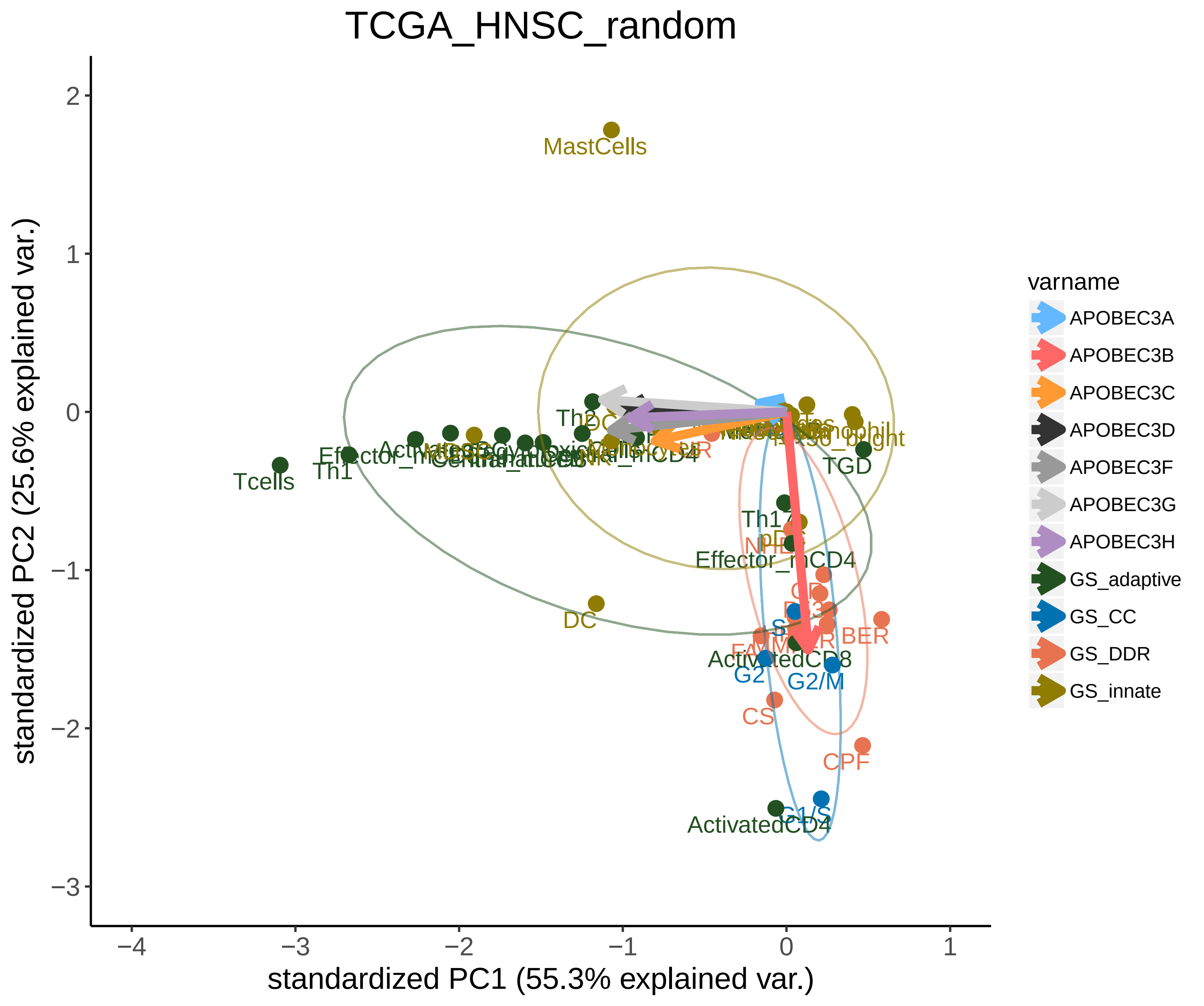
TCGA_HNSC_random
standardized PC2 (25.6% explained var.)
standardized PC1 (55.3% explained var.)
2
1
0
−1
−2
−3
−4
−3
−2
−1
0
1
MastCells
Tcells
Th1
TGD
Th17
Effector_mCD4
DC
BER
CPF
CS
G2
G2/M
ActivatedCD4
varname
APOBEC3A
APOBEC3B
APOBEC3C
APOBEC3D
APOBEC3F
APOBEC3G
APOBEC3H
GS_adaptive
GS_CC
GS_DDR
GS_innate

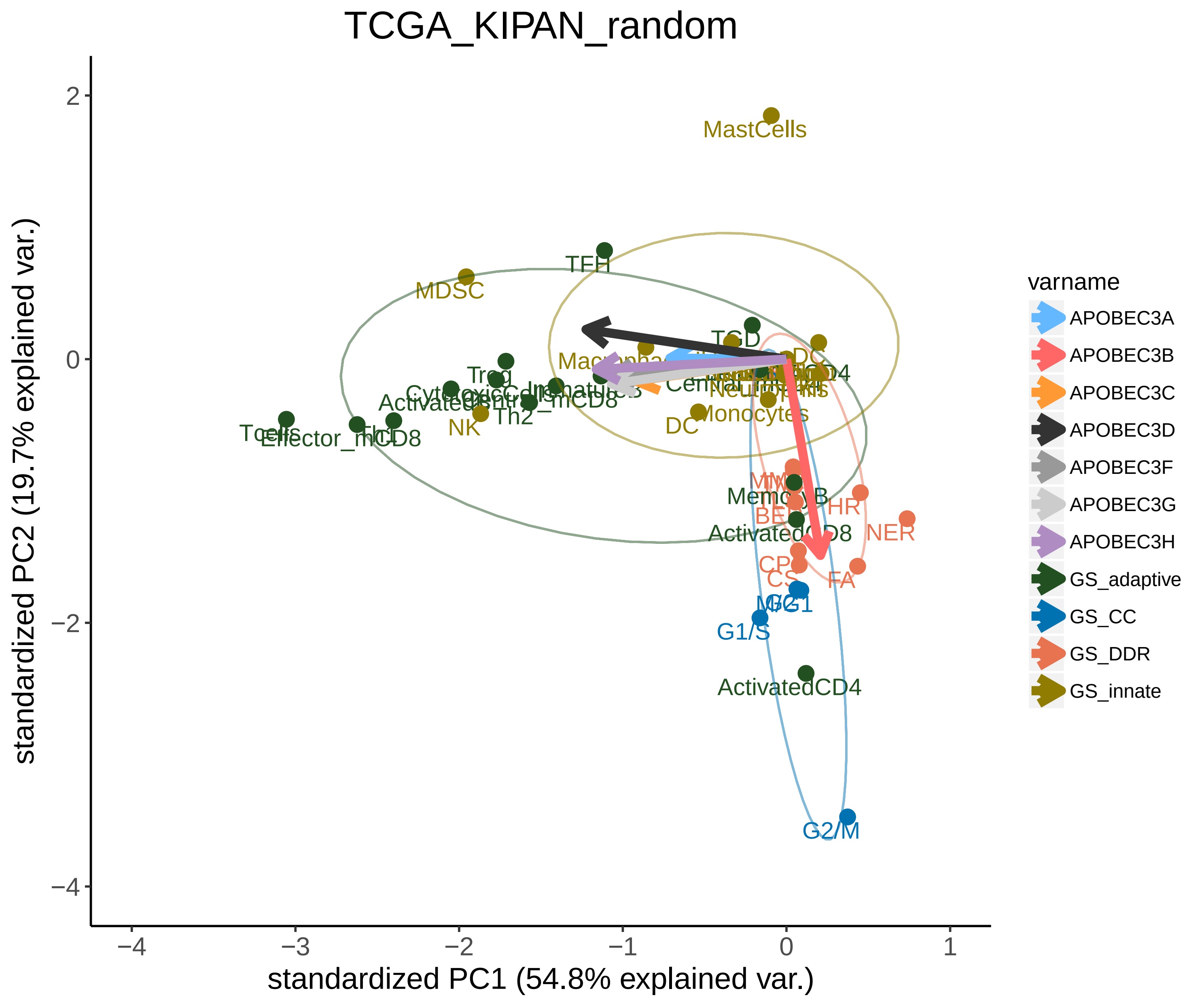

TCGA_KIPAN_random
standardized PC1 (54.8% explained var.)
standardized PC2 (19.7% explained var.)
−4
−3
−2
−1
0
1
2
0
−2
−4
varname
APOBEC3A
APOBEC3B
APOBEC3C
APOBEC3D
APOBEC3F
APOBEC3G
APOBEC3H
GS_adaptive
GS_CC
GS_DDR
GS_innate
MastCells
TFH
MDSC
TGD
Treg
NK
Th2
DC
Monocytes
Memory B
ActivatedCD8
ActivatedCD4
HR
NER
FA
G1/S
G2/M

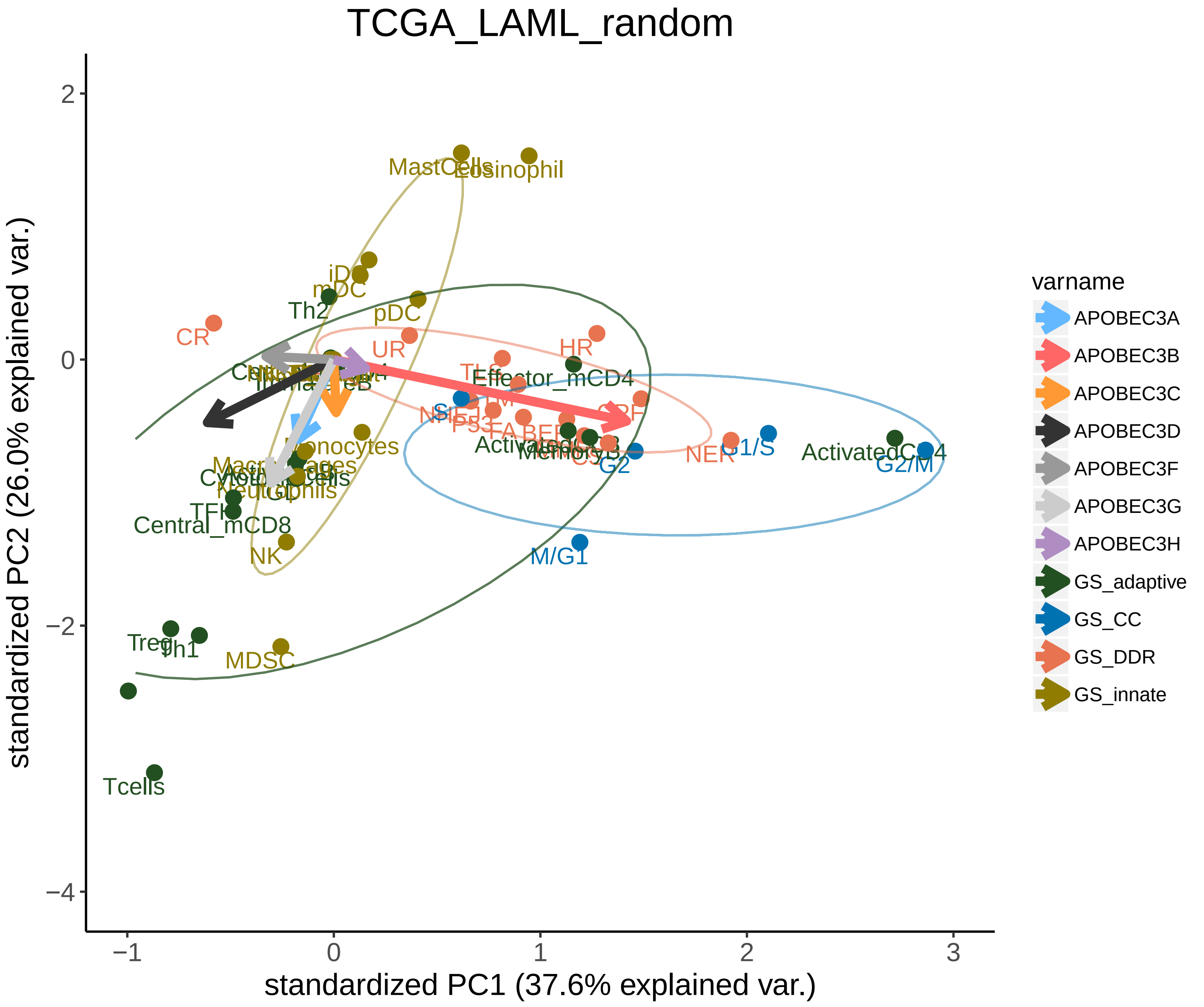

TCGA_LAML_random
standardized PC2 (26.0% explained var.)
standardized PC1 (37.6% explained var.)
2
0
−2
−4
−1
0
1
2
3
varname
APOBEC3A
APOBEC3B
APOBEC3C
APOBEC3D
APOBEC3F
APOBEC3G
APOBEC3H
GS_adaptive
GS_CC
GS_DDR
GS_innate
MastCells
Eosinophil
iDC
mDC
Th2
pDC
CR
UR
HR
Effector_mCD4
NER
G1/S
ActivatedCD4
G2/M
G2
M/G1
Central_mCD8
NK
MDSC
Treg
Th1
Tcells

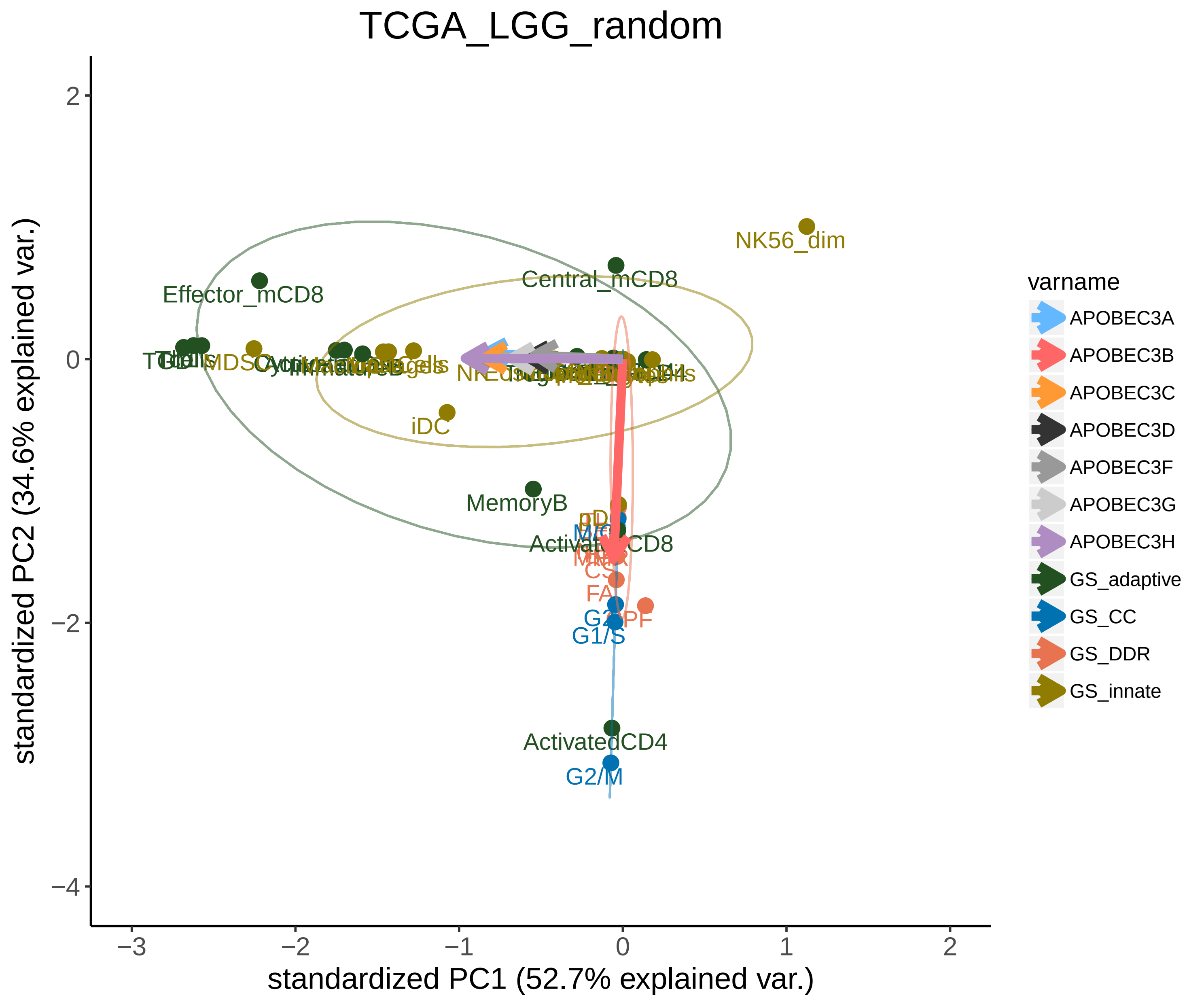

TCGA_LGG_random
standardized PC1 (52.7% explained var.)
standardized PC2 (34.6% explained var.)
varname
APOBEC3A
APOBEC3B
APOBEC3C
APOBEC3D
APOBEC3F
APOBEC3G
APOBEC3H
GS_adaptive
GS_CC
GS_DDR
GS_innate
NK56_dim
Central_mCD8
Effector_mCD8
iDC
MemoryB
ActivatedCD4
G2/M
G1/S
FA

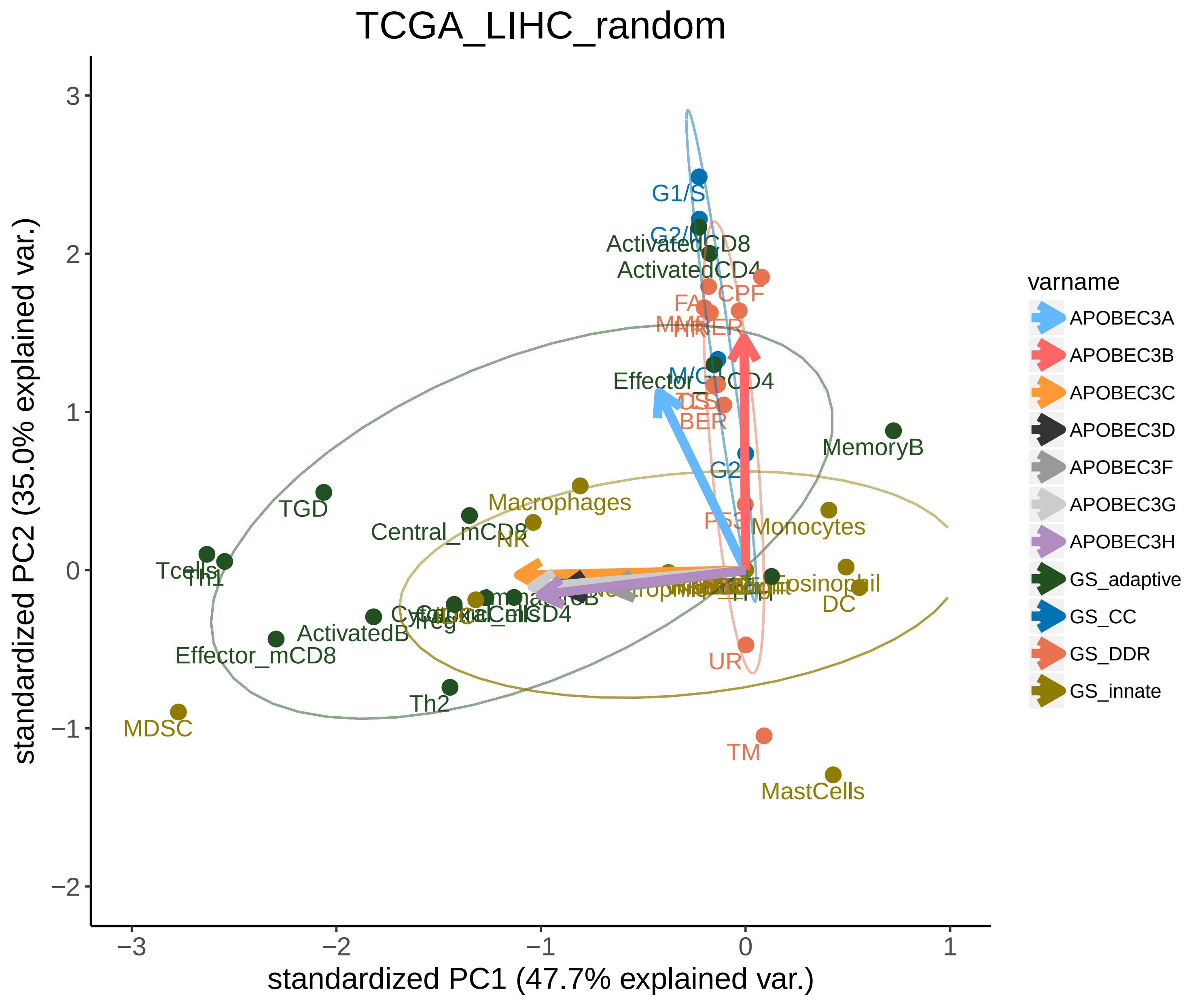
TCGA_LIHC_random
standardized PC1 (47.7% explained var.)
standardized PC2 (35.0% explained var.)
varname
APOBEC3A
APOBEC3B
APOBEC3C
APOBEC3D
APOBEC3F
APOBEC3G
APOBEC3H
GS_adaptive
GS_CC
GS_DDR
GS_innate
G1/S
ActivatedCD8
ActivatedCD4
CPF
MemoryB
G2
TGD
Macrophages
Central_mCD8
NK
Monocytes
Tcells
Th1
Eosinophil
DC
ActivatedB
Effector_mCD8
UR
Th2
MDSC
TM
MastCells

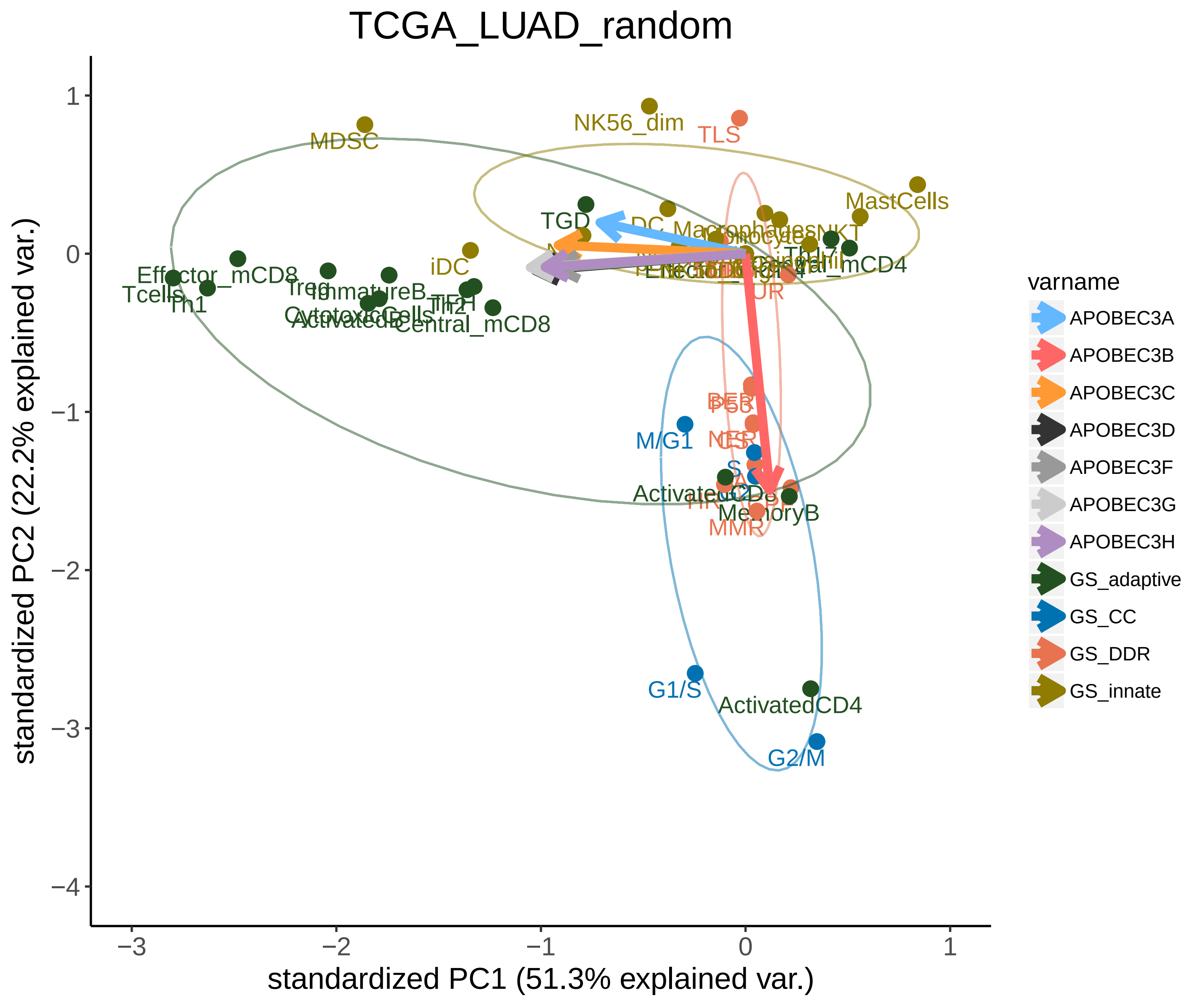
TCGA_LUAD_random
standardized PC2 (22.2% explained var.)
standardized PC1 (51.3% explained var.)
1
0
−1
−2
−3
−4
−3
−2
−1
0
1
NK56_dim
TLS
MDSC
MastCells
TGD
NKT
iDC
Effector_mCD8
Tcells
Th1
Treg
ImmatureB
Th2
TFH
Central_mCD8
M/G1
S
MemoryB
MMR
G1/S
ActivatedCD4
G2/M
varname
APOBEC3A
APOBEC3B
APOBEC3C
APOBEC3D
APOBEC3F
APOBEC3G
APOBEC3H
GS_adaptive
GS_CC
GS_DDR
GS_innate

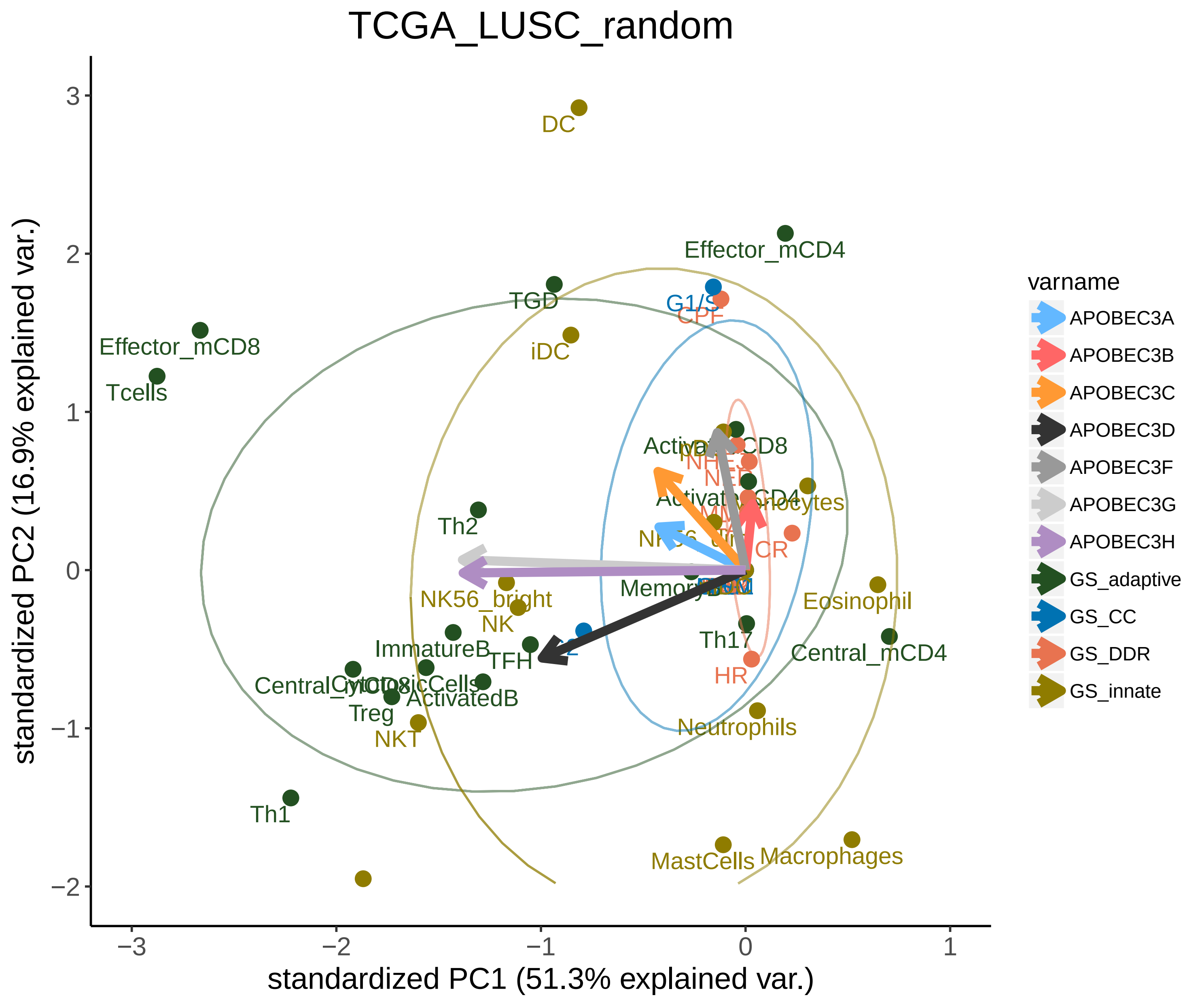

TCGA_LUSC_random
standardized PC2 (16.9% explained var.)
standardized PC1 (51.3% explained var.)
3
2
1
0
−1
−2
−3
−2
−1
0
1
DC
Effector_mCD4
TGD
G1/S
CPF
Effector_mCD8
iDC
Tcells
Th2
NK56_bright
NK
ImmatureB
TFH
Central_mCD8
CytotoxicCells
ActivatedB
Treg
NKT
Th1
Th17
Central_mCD4
HR
Eosinophil
Neutrophils
MastCells
Macrophages
varname
APOBEC3A
APOBEC3B
APOBEC3C
APOBEC3D
APOBEC3F
APOBEC3G
APOBEC3H
GS_adaptive
GS_CC
GS_DDR
GS_innate

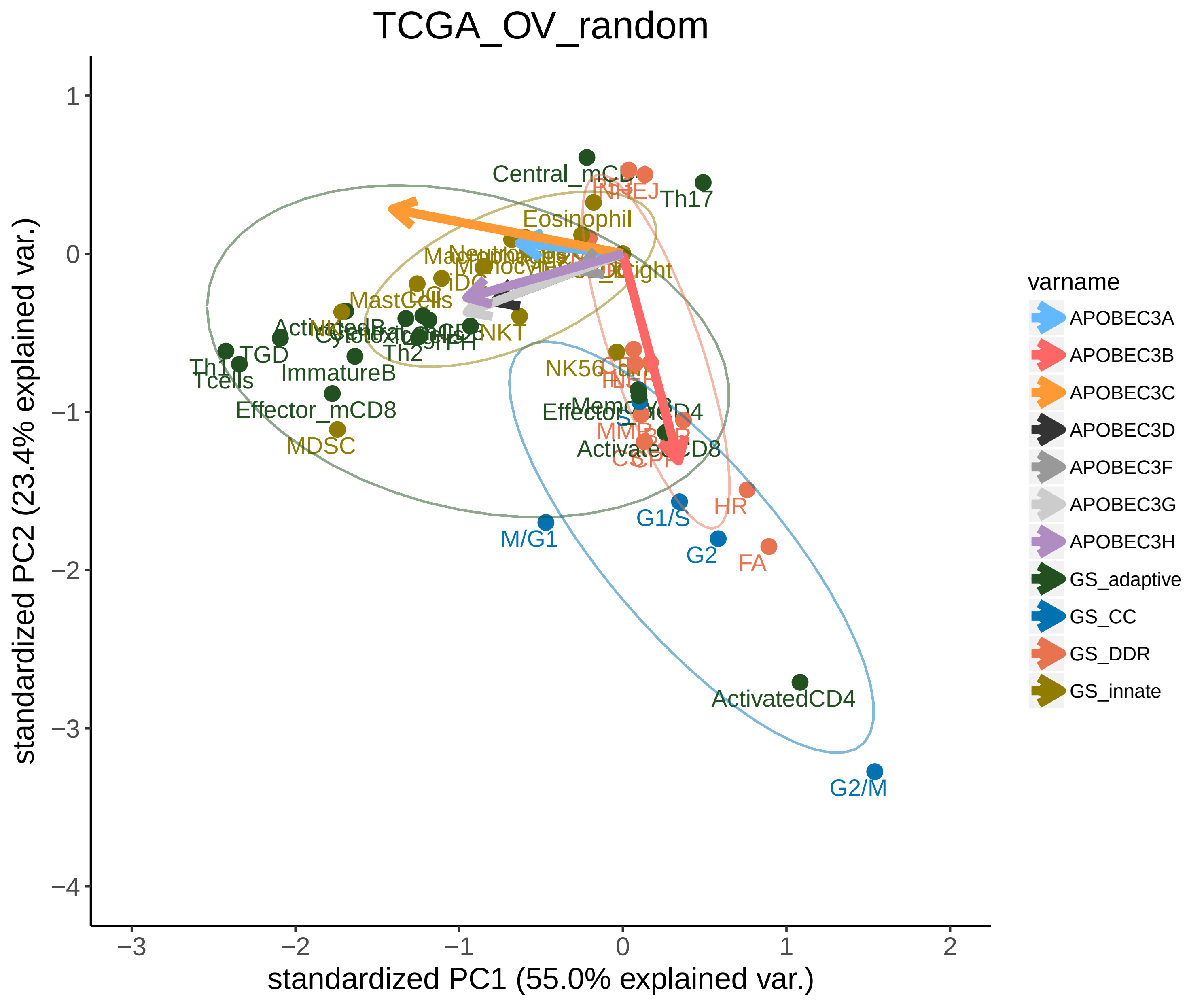
TCGA_OV_random
standardized PC2 (23.4% explained var.)
standardized PC1 (55.0% explained var.)
1
0
−1
−2
−3
−4
−3
−2
−1
0
1
2
Central_mCD4
NHEJ
Th17
Eosinophil
Th1
TGD
Tcells
ImmatureB
Th2
NKT
MastCells
iDC
Effector_mCD8
MDSC
NK56
Activated CD8
HR
G1/S
M/G1
G2
FA
ActivatedCD4
G2/M
varname
APOBEC3A
APOBEC3B
APOBEC3C
APOBEC3D
APOBEC3F
APOBEC3G
APOBEC3H
GS_adaptive
GS_CC
GS_DDR
GS_innate

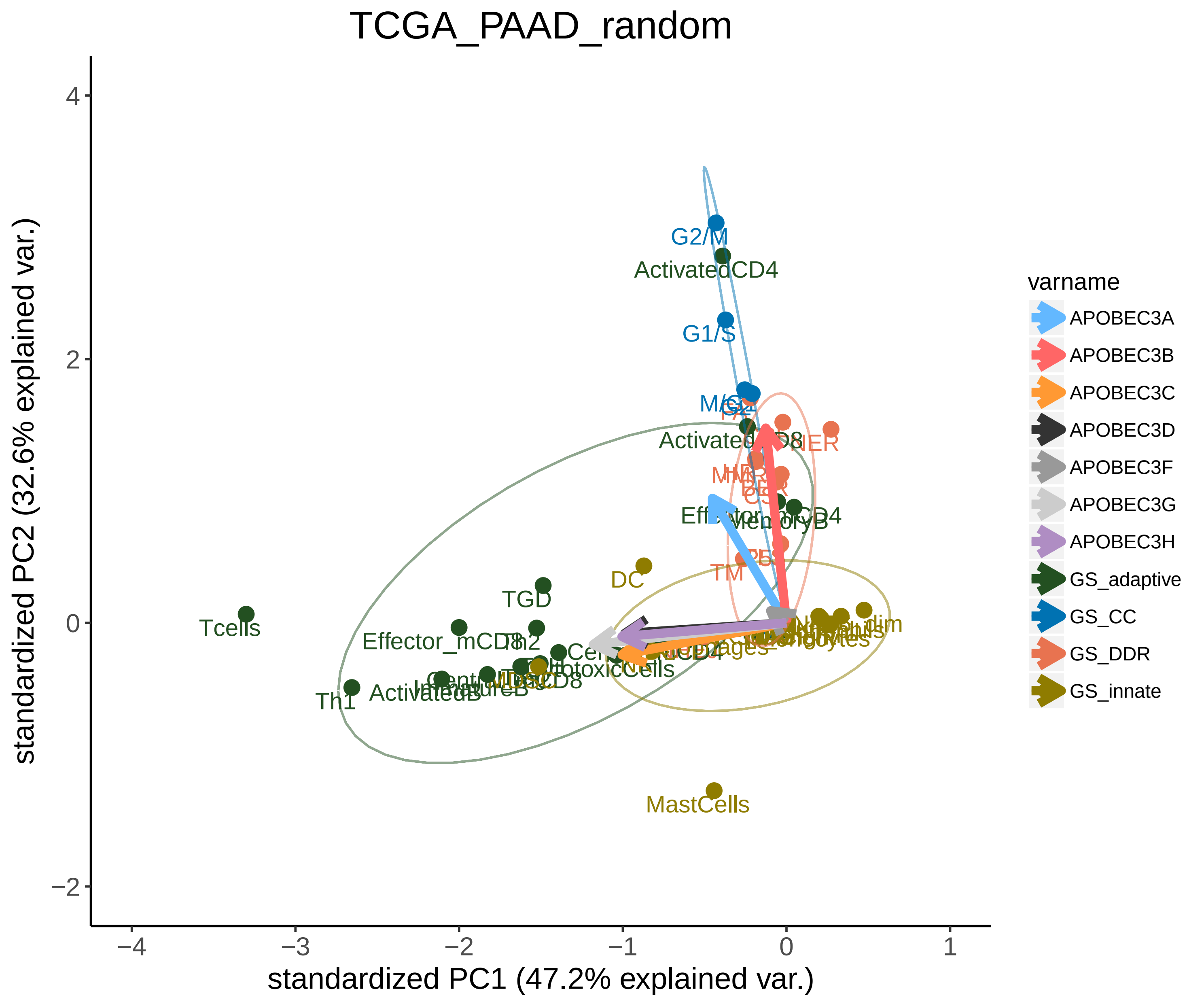
TCGA_PAAD_random
standardized PC1 (47.2% explained var.)
standardized PC2 (32.6% explained var.)
−4
−3
−2
−1
0
1
4
2
0
−2
varname
APOBEC3A
APOBEC3B
APOBEC3C
APOBEC3D
APOBEC3F
APOBEC3G
APOBEC3H
GS_adaptive
GS_CC
GS_DDR
GS_innate
G2/M
ActivatedCD4
G1/S
Tcells
TGD
DC
Th1
MastCells
HER
TM
Effector_mCD8

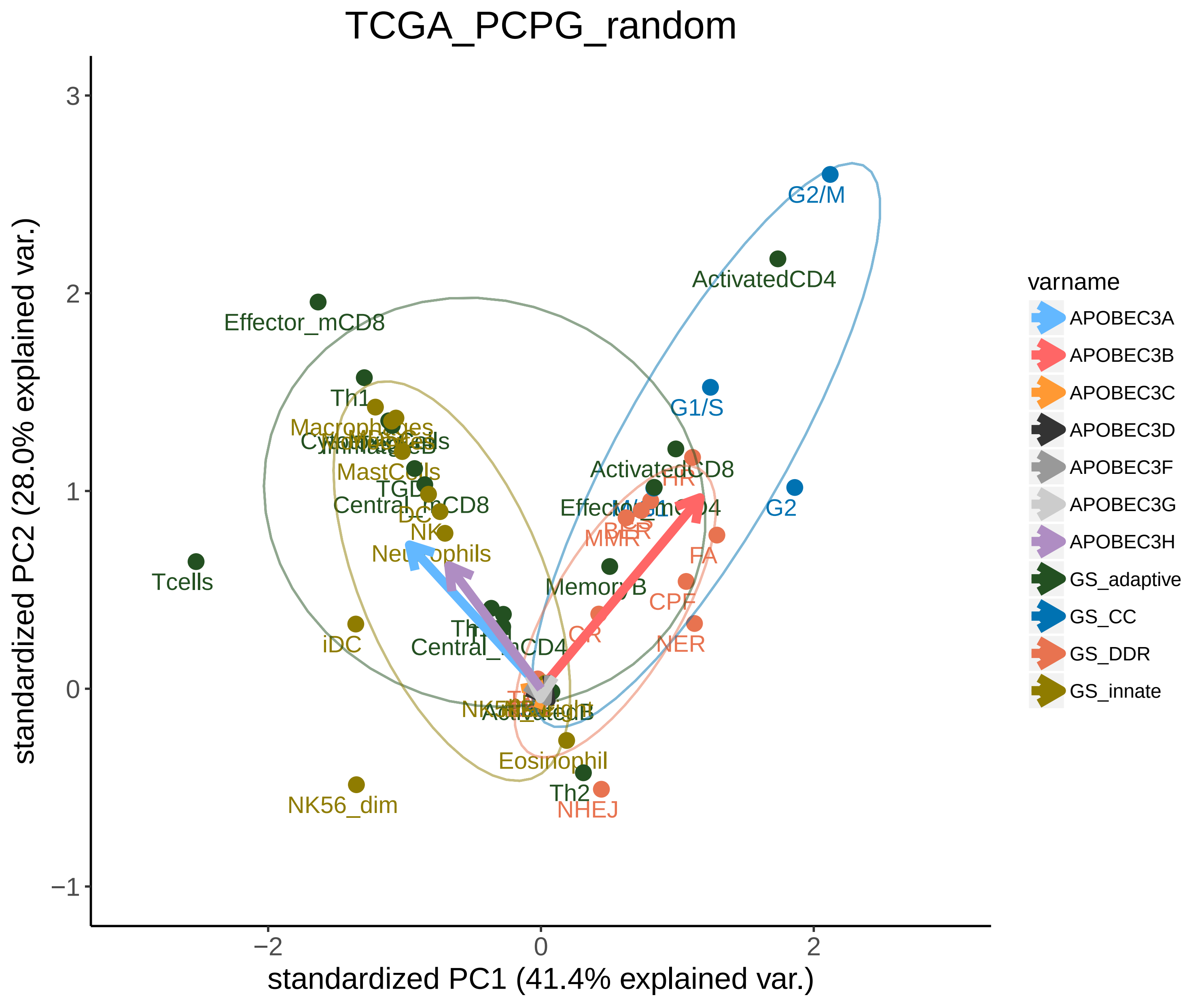
TCGA_PCPG_random
standardized PC1 (41.4% explained var.)
standardized PC2 (28.0% explained var.)
−2
0
2
−1
0
1
2
3
varname
APOBEC3A
APOBEC3B
APOBEC3C
APOBEC3D
APOBEC3F
APOBEC3G
APOBEC3H
GS_adaptive
GS_CC
GS_DDR
GS_innate
G2/M
ActivatedCD4
Effector_mCD8
G1/S
Th1
ActivatedCD8
MastCells
Central_mCD8
G2
FA
Memory B
CPF
NER
Tcells
iDC
Eosinophil
Th2
NHEJ
NK56_dim

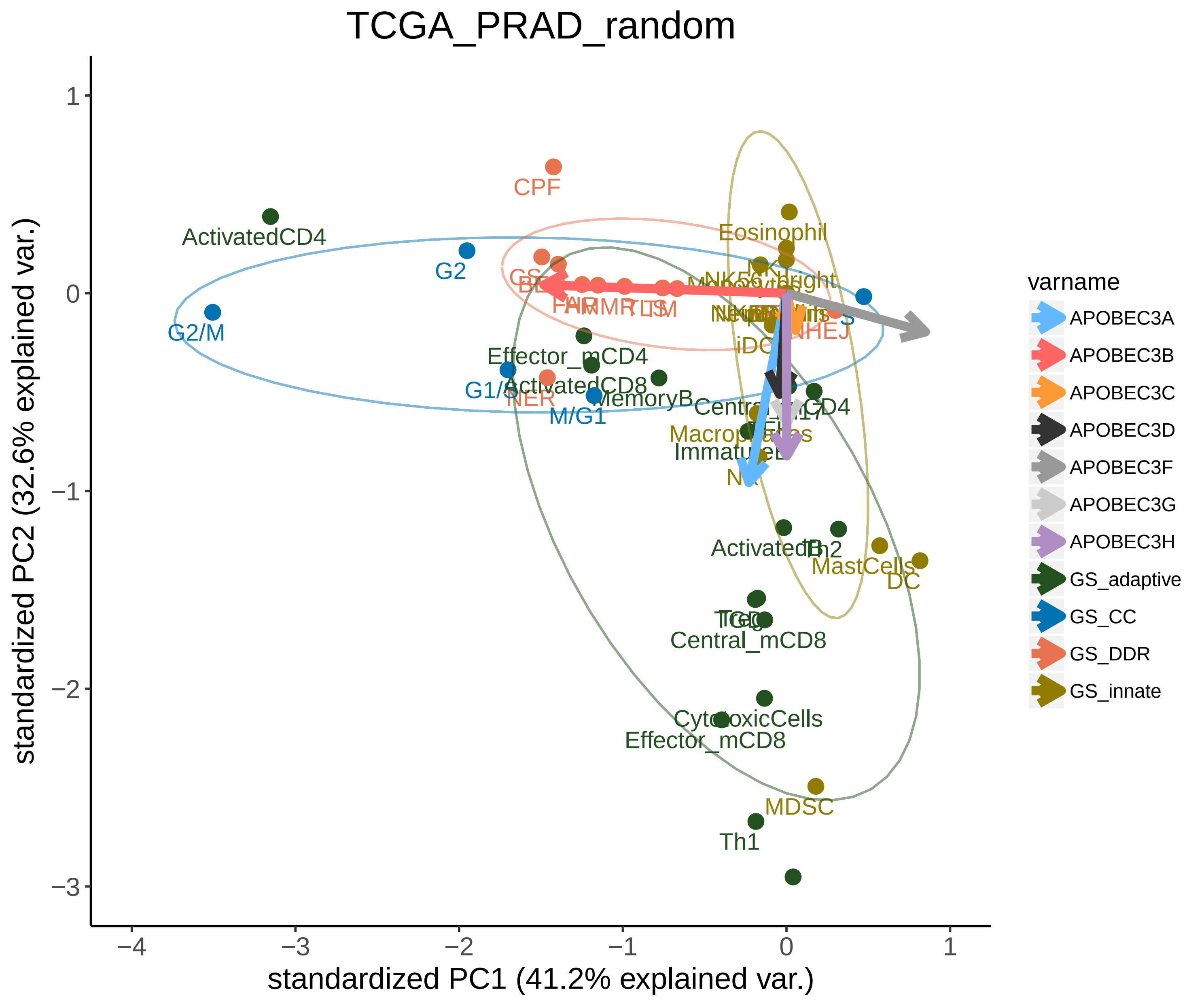
TCGA_PRAD_random
standardized PC2 (32.6% explained var.)
standardized PC1 (41.2% explained var.)
1
0
−1
−2
−3
−4
−3
−2
−1
0
1
CPF
ActivatedCD4
Eosinophil
G2
G2/M
Effector_mCD4
ActivatedCD8
MemoryB
M/G1
NER
Macrophages
NK
iDC
NHEJ
Th2
MastCells
DC
Central_mCD8
CytotoxicCells
Effector_mCD8
MDSC
Th1
varname
APOBEC3A
APOBEC3B
APOBEC3C
APOBEC3D
APOBEC3F
APOBEC3G
APOBEC3H
GS_adaptive
GS_CC
GS_DDR
GS_innate

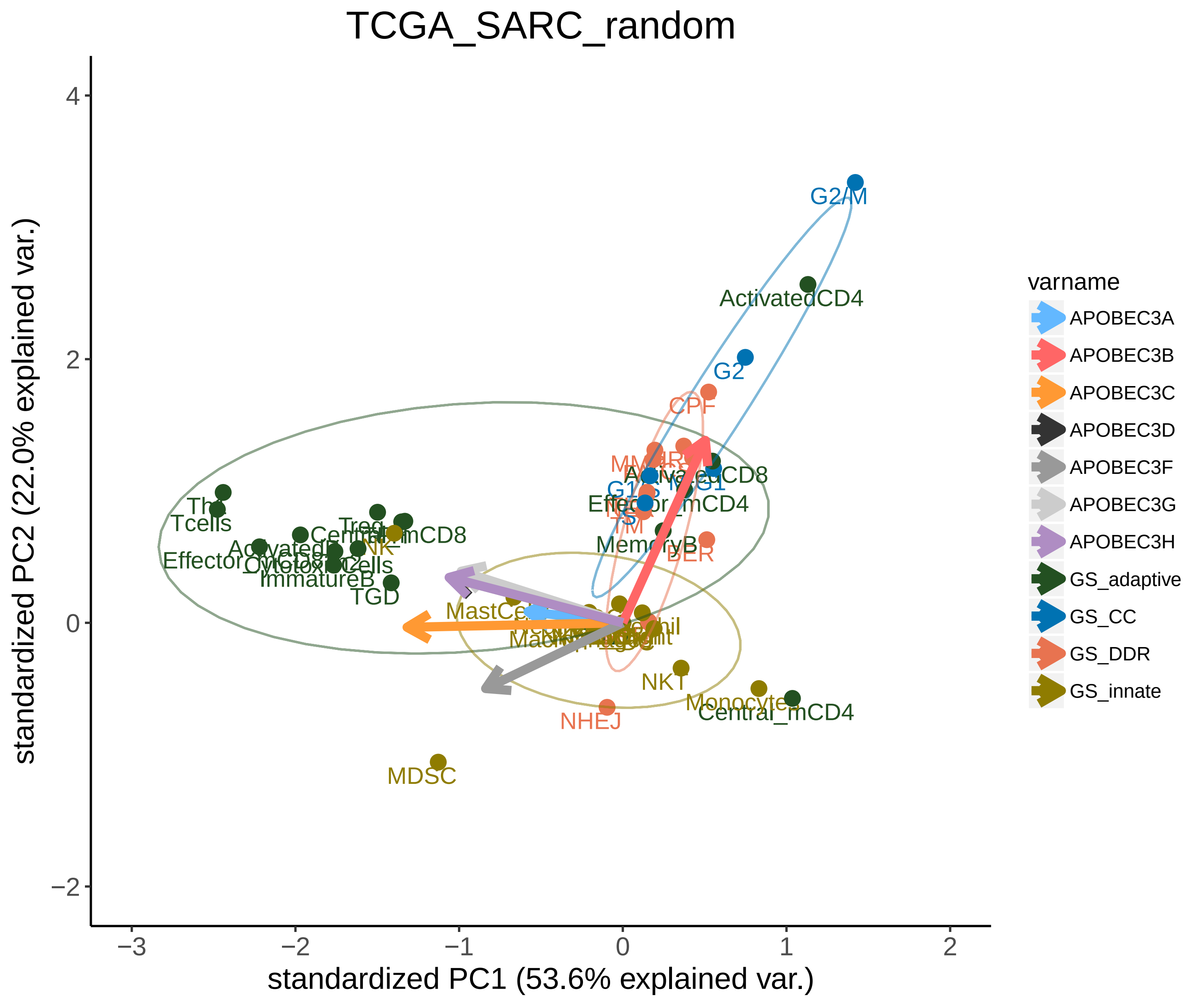
TCGA_SARC_random
standardized PC1 (53.6% explained var.)
standardized PC2 (22.0% explained var.)
varname
APOBEC3A
APOBEC3B
APOBEC3C
APOBEC3D
APOBEC3F
APOBEC3G
APOBEC3H
GS_adaptive
GS_CC
GS_DDR
GS_innate
G2/M
ActivatedCD4
G2
CPF
ActivatedCD8
Effector_mCD4
MemoryB
BER
Th1
Tcells
Treg
Central_mCD8
NK
ImmatureB
TGD
MastCell
NKT
Monocyte
Central_mCD4
NHEJ
MDSC
4
2
0
−2
−3
−2
−1
0
1
2

TCGA_SKCM_random

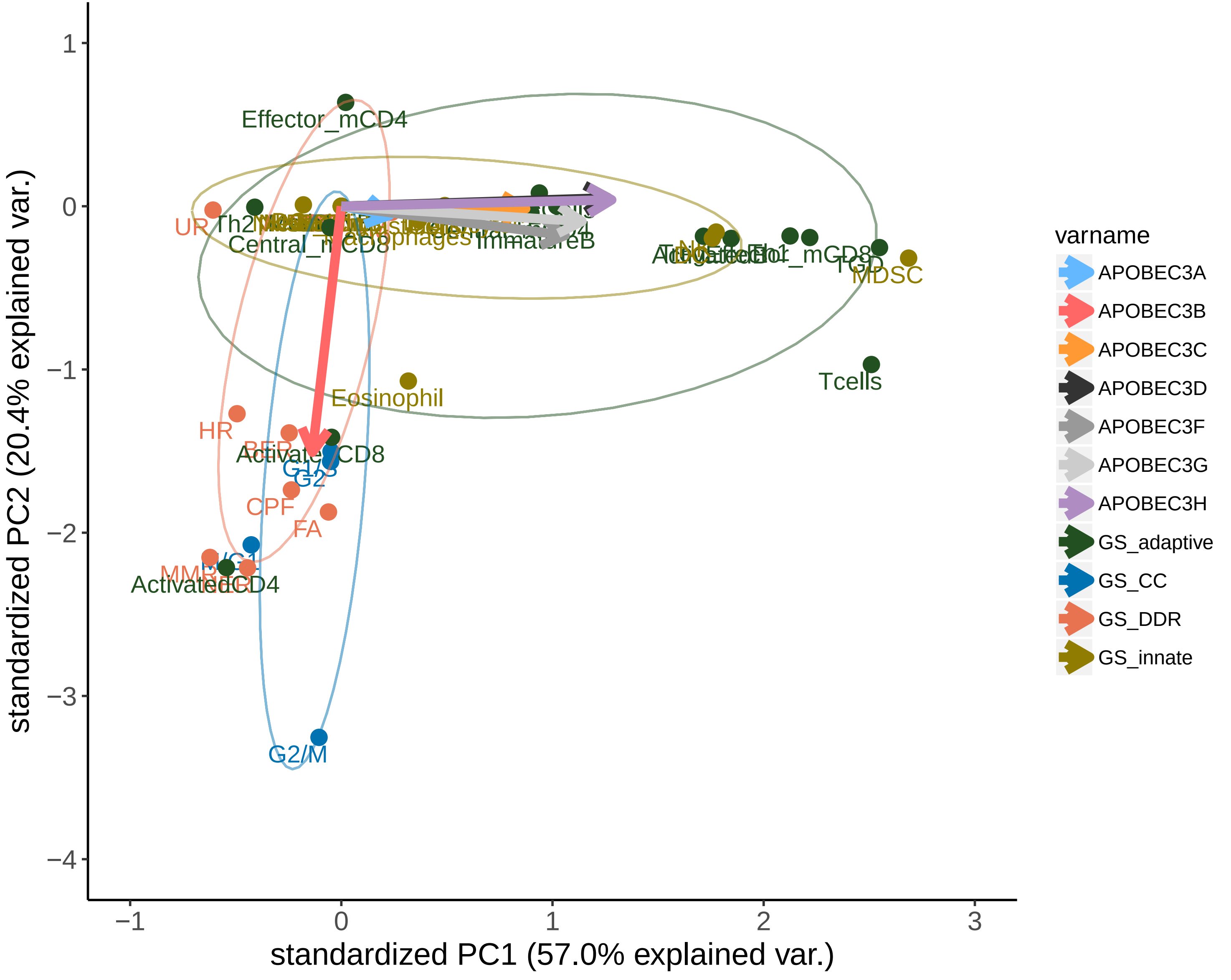

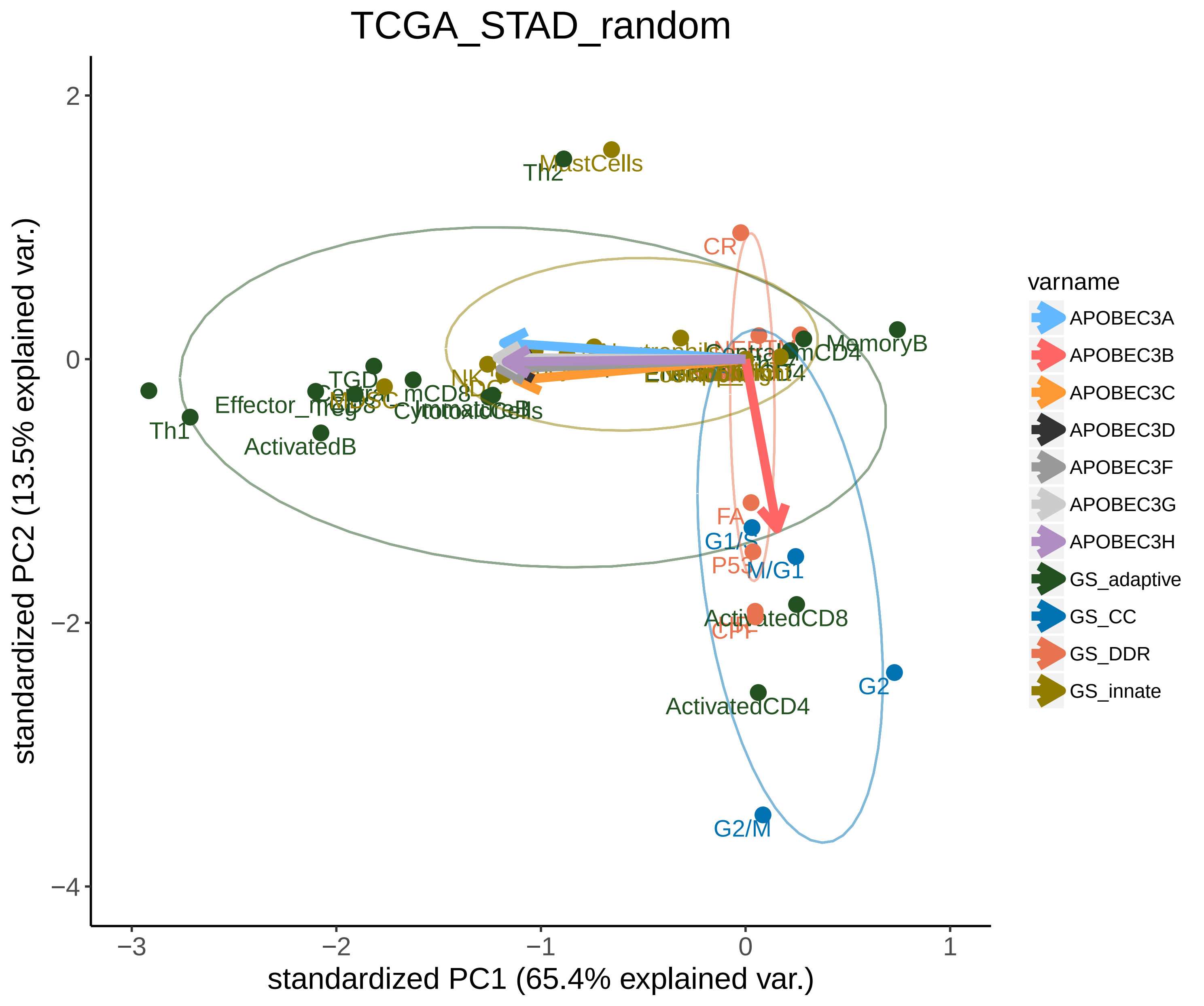

TCGA_STAD_random
standardized PC2 (13.5% explained var.)
standardized PC1 (65.4% explained var.)
2
0
−2
−4
−3
−2
−1
0
1
varname
APOBEC3A
APOBEC3B
APOBEC3C
APOBEC3D
APOBEC3F
APOBEC3G
APOBEC3H
GS_adaptive
GS_CC
GS_DDR
GS_innate
MastCells
Th2
CR
MemoryB
TGD
Effector_mCD8
Th1
ActivatedB
FA
G1/S
P53
M/G1
ActivatedCD8
G2
ActivatedCD4
G2/M

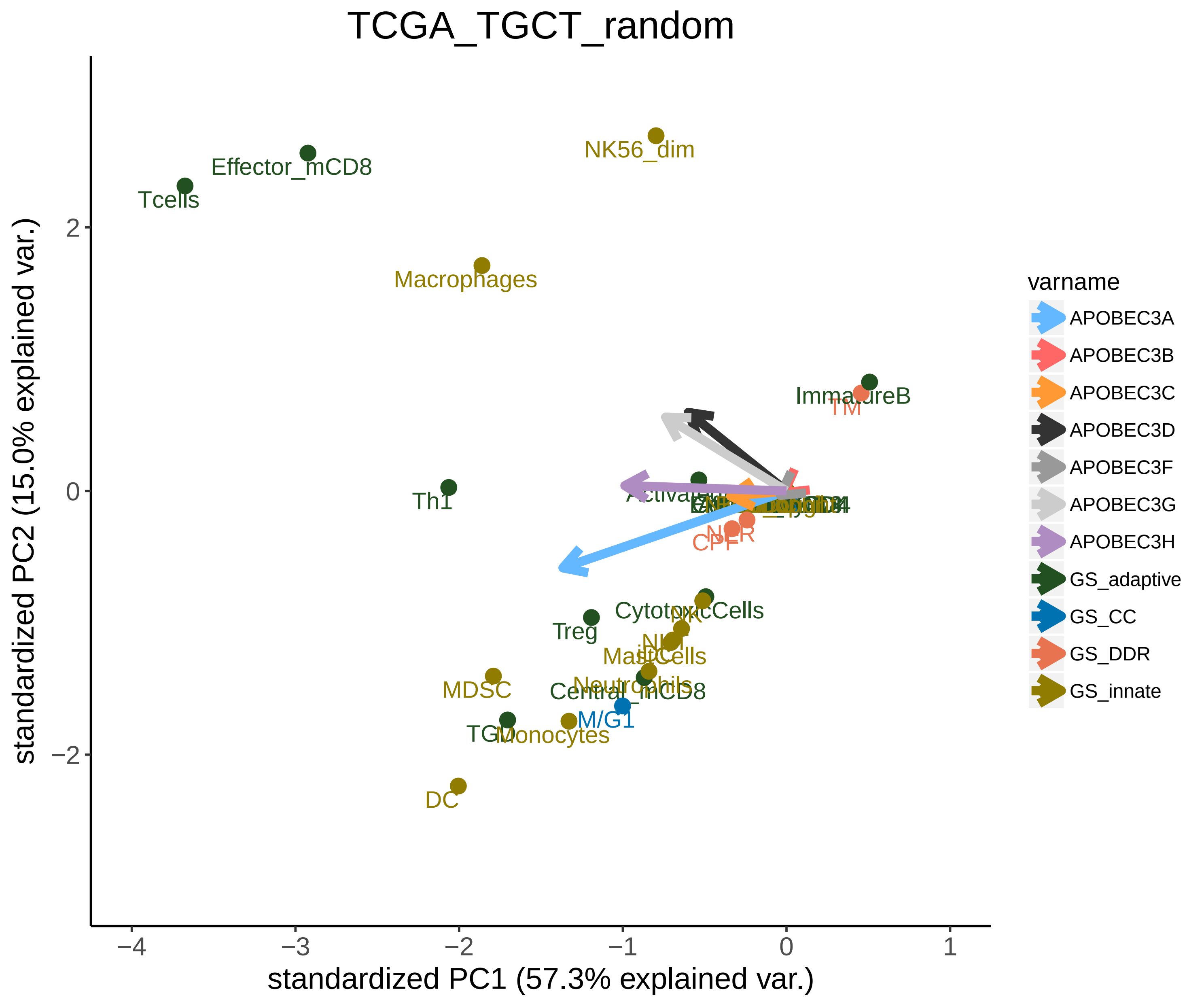
TCGA_TGCT_random
standardized PC2 (15.0% explained var.)
standardized PC1 (57.3% explained var.)
2
0
−2
−4
−3
−2
−1
0
1
Effector_mCD8
Tcells
NK56_dim
Macrophages
ImmatureB
TM
Th1
NER
CPF
CytotoxicCells
NK
Treg
MastCells
MDSC
Neutrophils
Central_mCD8
M/G1
Monocytes
DC
varname
APOBEC3A
APOBEC3B
APOBEC3C
APOBEC3D
APOBEC3F
APOBEC3G
APOBEC3H
GS_adaptive
GS_CC
GS_DDR
GS_innate

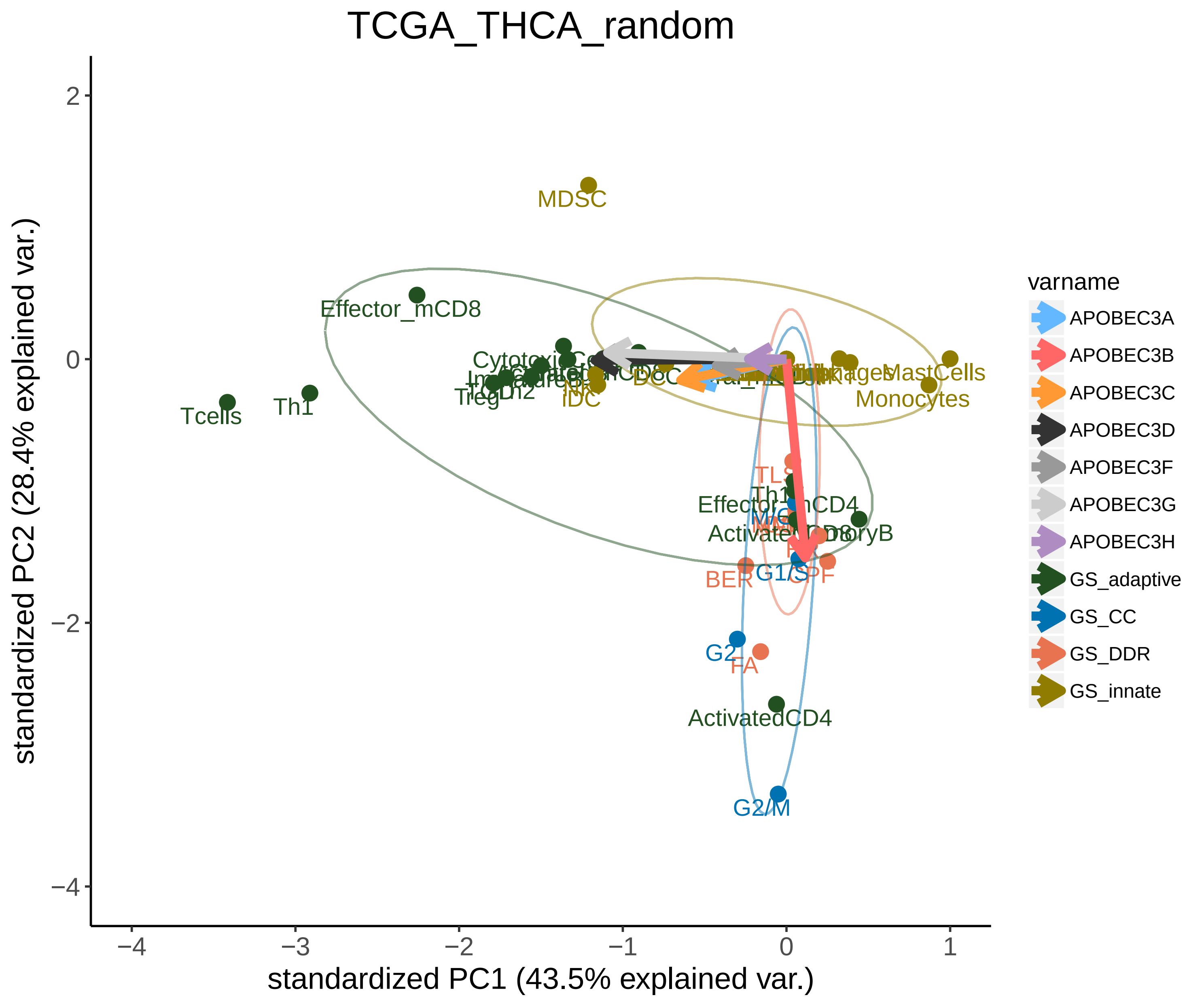
TCGA_THCA_random
standardized PC2 (28.4% explained var.)
standardized PC1 (43.5% explained var.)
2
0
−2
−4
−4
−3
−2
−1
0
1
MDSC
Effector_mCD8
Tcells
Th1
Treg
Th2
NK
iDC
DC
MastCells
Monocytes
TLS
Th17
Effector_mCD4
BER
G1/S
G2
FA
ActivatedCD4
G2/M
varname
APOBEC3A
APOBEC3B
APOBEC3C
APOBEC3D
APOBEC3F
APOBEC3G
APOBEC3H
GS_adaptive
GS_CC
GS_DDR
GS_innate

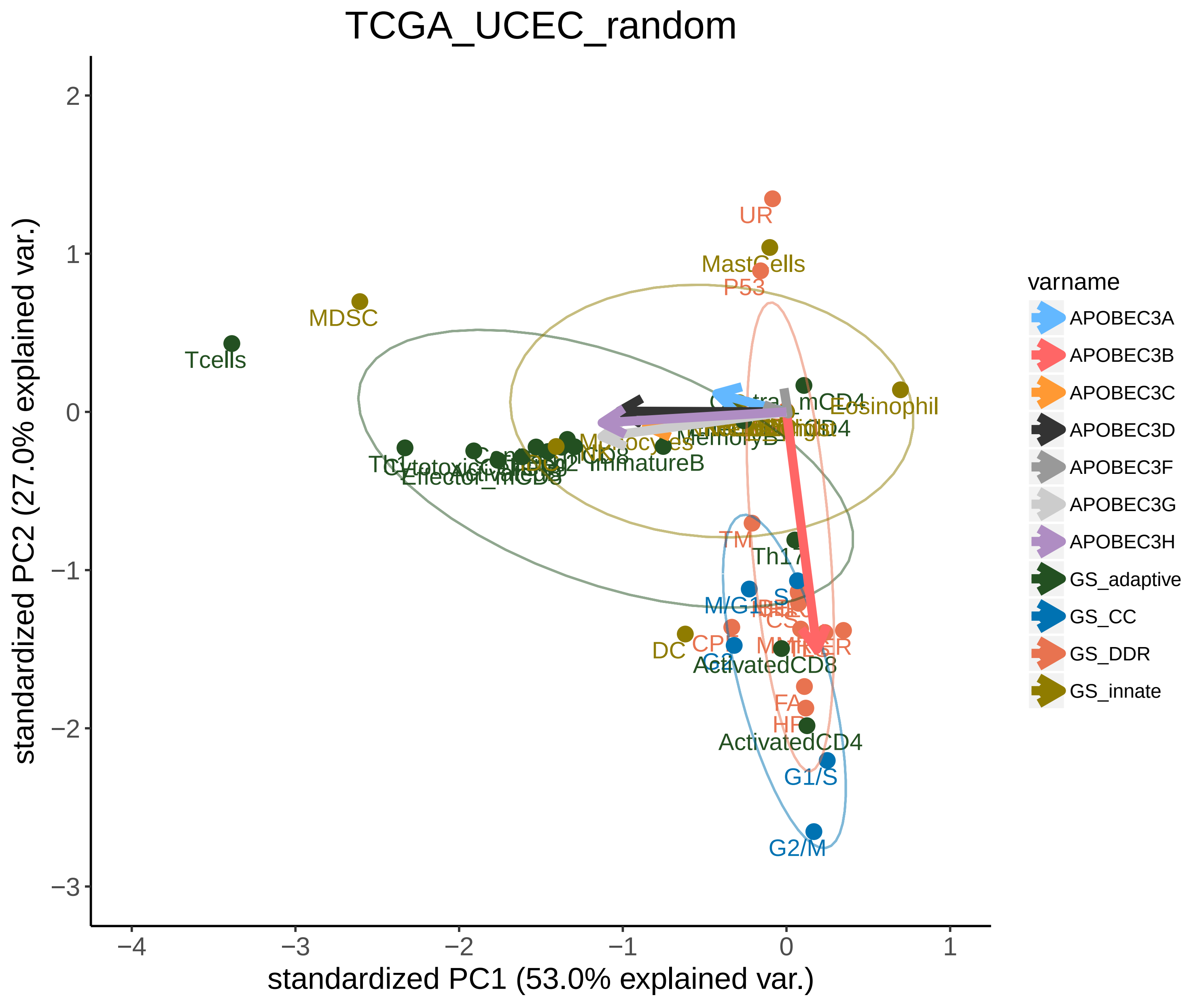

TCGA_UCEC_random
standardized PC1 (53.0% explained var.)
standardized PC2 (27.0% explained var.)
varname
APOBEC3A
APOBEC3B
APOBEC3C
APOBEC3D
APOBEC3F
APOBEC3G
APOBEC3H
GS_adaptive
GS_CC
GS_DDR
GS_innate
UR
MastCells
P53
MDSC
Tcells
Eosinophil
ImmatureB
MemoryB
TM
Th17
M/G1
DC
ActivatedCD8
ActivatedCD4
G1/S
G2/M

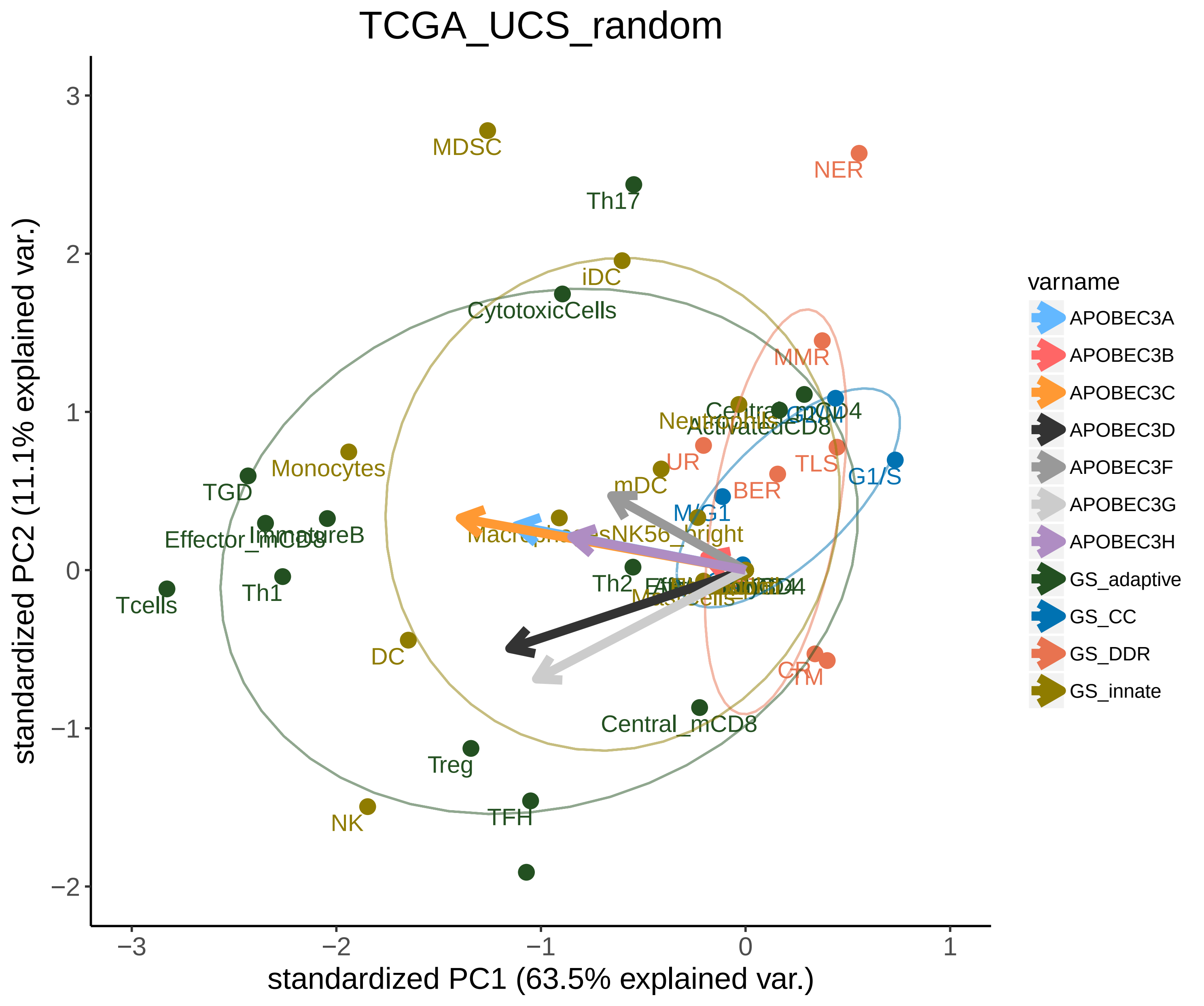
TCGA_UCS_random
standardized PC2 (11.1% explained var.)
standardized PC1 (63.5% explained var.)
3
2
1
0
−1
−2
−3
varname
APOBEC3A
APOBEC3B
APOBEC3C
APOBEC3D
APOBEC3F
APOBEC3G
APOBEC3H
GS_adaptive
GS_CC
GS_DDR
GS_innate
MDSC
NER
Th17
iDC
CytotoxicCells
MMR
ActivatedCD8
Monocytes
TGD
UR
TLS
G1/S
mDC
BER
M/G1
Effector_mCD8
ImmatureB
Tcells
Th1
Th2
DC
CR
TM
Central_mCD8
Treg
NK
TFH
