# Supplementary material for: Pan-cancer transcriptomic analysis dissects immune and proliferative functions of APOBEC3 cytidine deaminases
Source: Nucleic Acids Res. 2019 Jan 9;47(3):1178–94. doi: 10.1093/nar/gky1316 (PMC6379723; doi:10.1093/nar/gky1316)
Supplement: Supplementary Data [file gky1316_supplemental_files.zip › supplementary_v8_combined-with-numbers.pdf]

Supplementary Information

Pan-cancer transcriptomic analysis dissects immune and proliferative functions of APOBEC3 cytidine deaminases.

Joseph CF Ng[1], Jelmar Quist[2], Anita Grigoriadis[2], Michael H Malim[3] & Franca Fraternali[1]

Affiliation

[1]Randall Centre for Cell and Molecular Biophysics, King's College London, United Kingdom
[2]Cancer Bioinformatics, School of Cancer and Pharmaceutical Sciences, CRUK King's Health Partners Centre, Breast Cancer Now Research Unit, King's College London, United Kingdom
[3]Department of Infectious Diseases, School of Immunology and Microbial Sciences, King's College London, United Kingdom

Correspondence to: Prof. Franca Fraternali (franca.fraternali@kcl.ac.uk)

Table of Contents

Supplementary Table S1. Summary statistics of all RNA-seq cohorts utilised in this analysis.

Supplementary Table S2. Purity-APOBEC3 expression correlation data.

Supplementary Table S3. Pairwise comparison p-values for Purity-APOBEC3 expression correlation analysis.

Supplementary Table S4. Statistics from tests of differences in correlations between APOBEC3 gene expression and inferred immune cell type proportions.

Supplementary Table S5. Immune cell level-APOBEC3 correlation data.

Supplementary Table S6. Pairwise comparison p-values for Immune cell type proportion-APOBEC3 expression correlation analysis.

Supplementary Table S7. Tumour/tissue-specific ratio.

Supplementary Table S8. Pairwise comparison p-values for the tumour/tissue-specific ratio analysis.

Supplementary Table S9. Cell-type proportion estimates and RESPECTEx analysis of the breast cancer cohort GSE79688

Supplementary Table S10. APOBEC3 gene co-expression partners across cohorts.

Supplementary Table S11. Statistics of APOBEC3 gene co-expression partners across cohorts and overlaps with bootstrapping samples.

Supplementary Table S12. Gene set enrichment analysis of APOBEC3 gene co-expression partners

Supplementary Table S13. Distinguishing genes for APOBEC3s

Supplementary Figure S1. Summary flowchart for this study.

Supplementary Figure S2. Association between various tumour purity measurements/estimates and APOBEC3 gene expression.

Supplementary Figure S3. Correlation boxplots of various tumour purity measurements/estimates with APOBEC3 gene expression.

Supplementary Figure S4. Correlation boxplots of immune cell types proportions with APOBEC3 gene expression.

Supplementary Figure S5. Association of the estimated T cells and Macrophage M1 levels and APOBEC3 gene expression.

Supplementary Figure S6. t-Distributed Stochastic Neighbour Embedding (t-SNE) analysis of the immune cell type-APOBEC3 expression correlation data.

Supplementary Figure S7. Correlation boxplots of tumour/nonimmune specific ratio for TCGA and GTEx cohorts.

Supplementary Figure S8. Case-by-case expression heatmap for single-cell RNA sequencing data analysed in this study.

Supplementary Figure S9. Correlation between RESPECTEx-deconvolved cell-type specific expression with expression in marker gene-positive single cells.

Supplementary Figure S10. Statistics of co-expressing genes extracted.

Supplementary Figure S11. Overlap of gene sets.

Supplementary Figure S12. Functional barcodes of APOBEC3 co-expressing genes.

Supplementary Figure S13. APOBEC3 gene co-expression networks of tumours, cancer cell lines and normal tissues.

Supplementary Figure S14. APOBEC3 gene co-expression networks extracted from the median of bootstrapped samples.

Supplementary Figure S15. Gene Set Enrichment Analysis (GSEA) of co-expressing genes of APOBEC3 genes.

Supplementary Figure S16. GSEA of co-expressing genes of APOBEC3 genes extracted from the median of bootstrapped samples.

Supplementary Figure S17. Quantifying the overlap of co-expressing genes shared between pairs of APOBEC3 genes.

Supplementary Figure S18. Relationship between expression of selected genes with APOBEC3 gene expression.

Supplementary Figure S19. Distinguishing genes barcodes for APOBEC3 genes in the TCGA cohorts.

Supplementary Figure S20. Distinguishing genes barcodes for APOBEC3 genes in the GTEx cohorts.

Supplementary Figure S21. Fluctuations in transcriptome and its relation to functional groups of genes.

Supplementary Figure S22. Bar-plots of APOBEC3 gene expression values across all examined cohorts

Supplementary Figure S23. Bar-plots of APOBEC3 gene expression values reconstituted using RESPECTEx for immune and non-immune components of TCGA and GTEx samples

Supplementary Figure S24. Bar-plots of Normalised Enrichment Score (NES) of GO Cell Cycle and Immune Responses gene sets for APOBEC3 co-expressing genes in all examined cohorts

SUPPLEMENTARY TABLES

Supplementary Table S1. Summary statistics of all RNA-seq cohorts utilised in this analysis.

Case counts represent the number of cases for which the respective data are available. See Methods for the data sources utilised.

Normal Tissues (GTEx)

-	No. of cases analysed for		
**GTEx Site**	**expression & co-expression**	**purity**	**immune cell deconvolution**
Adrenal Gland	**145**	**145**	**1**
Bladder	**11**	**11**	**2**
Blood	**511**	**511**	**511**
Brain	**1259**	**1259**	**0**
Breast	**214**	**214**	**49**
Cervix Uteri	**11**	**11**	**2**
Colon	**345**	**345**	**153**
Esophagus	**686**	**686**	**171**
Head & Neck	NA	NA	NA
Kidney	**32**	**32**	**4**
Liver	**119**	**119**	**1**
Lung	**320**	**320**	**308**
Muscle	**430**	**430**	**1**
Ovary	**97**	**97**	**0**
Pancreas	**171**	**171**	**1**
Prostate	**106**	**106**	**15**
Skin	**891**	**891**	**0**
Stomach	**193**	**193**	**109**
Testis	**172**	**172**	**3**
Thyroid	**323**	**323**	**20**
Uterus	**83**	**83**	**6**

Tumours (TCGA)

-	-	No. of cases analysed for		
**TCGA code**	Description	**expression & co-expression**	**purity**	**immune cell deconvolution**
ACC	Adrenocortical carcinoma	**79**	**79**	**6**
PCPG	Pheochromocytoma and Paraganglioma	**179**	NA	**5**
BLCA	Bladder urothelial carcinoma	**408**	**408**	**127**
DLBC	Diffuse Large B-cell Lymphoma	**48**	NA	**48**
LAML	Acute myeloid leukaemia	**173**	NA	**118**
GBMLGG	Glioma	**669**	**666**	**466**
BRCA	Breast invasive carcinoma	**1093**	**1090**	**504**
CESC	Cervical and endocervical cancers	**304**	**304**	**149**
COADREAD	Colorectal adenocarcinoma	**379**	**378**	**86**
ESCA	Esophageal carcinoma	**184**	NA	**36**
HNSC	Head and Neck squamous cell carcinoma	**520**	**520**	**354**
KIPAN	Pan-kidney cohort	**889**	**887**	**243**
LIHC	Liver hepatocellular carcinoma	**371**	**371**	**32**
LUAD	Lung adenocarcinoma	**515**	**510**	**323**
LUSC	Lung squamous cell carcinoma	**501**	**496**	**342**
SARC	Sarcoma	**259**	NA	**128**
OV	Ovarian serous cystadenocarcinoma	**303**	**303**	**78**
PAAD	Pancreatic adenocarcinoma	**178**	NA	**65**
PRAD	Prostate adenocarcinoma	**497**	**490**	**11**
SKCM	Skin cutaneous melanoma	**103**	**103**	**11**
STAD	Stomach adenocarcinoma	**415**	NA	**137**
TGCT	Testicular Germ Cell Tumors	**150**	NA	**105**
THCA	Thyroid carcinoma	**501**	**500**	**60**
UCEC	Uterine corpus endometrial carcinoma	**176**	**176**	**57**
UCS	Uterine carcinosarcoma	**57**	**57**	**18**

Cancer cell-lines (CCLE)

-	No. of cases analysed for
**CCLE code**	**expression & co-expression**
NA	NA
BLCA	**26**
DLBC	**57**
LAML	**31**
LGG	**65**
BRCA	**56**
CESC	**25**
COAD	**58**
ESCA	**26**
HNSC	**33**
KIPAN	**25**
LIHC	**32**
LUAD	**61**
LUSC	**50**
SARC	**40**
OV	**45**
PAAD	**41**
PRAD	**7**
SKCM	**52**
STAD	**41**
NA	NA
THCA	**12**
UCEC/UCS	**3**

Supplementary Table S2. Purity-APOBEC3 expression correlation data.

Uploaded separately.

Supplementary Table S3. Pairwise comparison p-values for Purity-APOBEC3 expression correlation analysis.

P-values were obtained from the Dunn's test by pairwise comparison of the distributions of Spearman correlations for each APOBEC3 gene, and adjusted by the Benjamini-Hochberg method.

TCGA CPE	A3A	A3B	A3C	A3D	A3F	A3G
A3B	0.0335					
A3C	0.0045	3.41E-06				
A3D	0.0052	4.43E-06	0.4793			
A3F	0.4604	0.0394	0.0036	0.0042		
A3G	7.27E-06	3.25E-10	0.0531	0.0446	5.38E-06	
A3H	0.0036	2.56E-06	0.4936	0.4711	2.98E-03	0.0662

GTEx ESTIMATE	A3A	A3B	A3C	A3D	A3F	A3G
A3B	0.4503					
A3C	0.0446	0.0795				
A3D	0.2768	0.2451	0.1511			
A3F	0.1760	0.1387	0.2544	0.3681		
A3G	0.0365	0.0436	0.4876	0.1409	0.2452	
A3H	0.3723	0.3598	0.0807	0.3897	0.2532	0.0726

TCGA CPE randomised	A3A	A3B	A3C	A3D	A3F	A3G
A3B	0.4545					
A3C	0.3701	0.3386				
A3D	0.5427	0.4416	0.4893			
A3F	0.7469	0.5351	0.4185	0.4606		
A3G	0.3841	0.3801	0.4723	0.4677	0.4231	
A3H	0.4759	0.4711	0.3989	0.3650	0.4303	0.3960

GTEx randomised	A3A	A3B	A3C	A3D	A3F	A3G
A3B	0.4459					
A3C	0.4779	0.5561				
A3D	0.2058	0.3115	0.2593			
A3F	0.5227	0.5611	0.5277	0.2289		
A3G	0.2698	0.3686	0.2923	0.4969	0.2509	
A3H	0.5013	0.4470	0.4825	0.3637	0.5333	0.7149

TCGA IHC	A3A	A3B	A3C	A3D	A3F	A3G
A3B	0.1960					
A3C	0.1365	0.0182				
A3D	0.3260	0.1258	0.2241			
A3F	0.3776	0.1560	0.1820	0.4309		
A3G	0.1017	0.0176	0.4022	0.1571	0.1359	
A3H	0.2148	0.4463	0.0241	0.1251	0.1439	0.0165

TCGA ESTIMATE	A3A	A3B	A3C	A3D	A3F	A3G
A3B	0.0958					
A3C	0.0010	4.32E-06				
A3D	6.48E-04	2.71E-06	0.4628			
A3F	0.1881	0.0152	0.0139	0.0120		
A3G	6.39E-08	1.14E-11	0.0150	0.0193	4.38E-06	
A3H	0.0011	5.00E-06	0.4703	0.4548	0.0147	0.0143

TCGA IHC randomised	A3A	A3B	A3C	A3D	A3F	A3G
A3B	0.4505					
A3C	0.6010	0.8296				
A3D	1.0000	0.8212	1.0000			
A3F	0.5098	0.5042	0.9322	0.6779		
A3G	0.7493	0.6467	1.0000	0.4869	0.5438	
A3H	0.6137	0.6091	1.0000	0.5798	0.4621	0.4921

TCGA ESTIMATE randomised	A3A	A3B	A3C	A3D	A3F	A3G
A3B	0.5855					
A3C	0.4772	0.4806				
A3D	0.4044	0.4015	0.3489			
A3F	0.5615	0.3516	0.3886	0.4801		
A3G	0.7171	0.3763	0.4353	0.5274	0.4901	
A3H	1.0000	0.4609	0.8606	0.5057	0.5545	0.5012

Supplementary Table S4. Statistics from tests of differences in correlations between APOBEC3 gene expression and inferred immune cell type proportions.

Only immune cell types included in the CIBERSORT LM22 matrix and inferred to be present in all examined TCGA and GTEx cohorts were tested by a Kruskal-Wallis (KW) test, its results, p-values and q-values (Benjamini-Hochberg) were presented here.

Normal tissue (GTEx)			
**cell type**	**Kruskal-Wallis H statistic**	**p-value**	**q-value**
Monocytes	18.0179	0.0062	0.0457
T cells CD8	17.4977	0.0076	0.0457
NK cells activated	15.6527	0.0157	0.0630
T cells follicular helper	13.4276	0.0367	0.1102
T cells CD4 memory resting	12.5435	0.0509	0.1221
Macrophages M1	10.5127	0.1047	0.2093
NK cells resting	9.8276	0.1321	0.2265
T cells regulatory (Tregs)	7.3461	0.2900	0.4215
Mast cells resting	6.8120	0.3386	0.4215
Macrophages M2	6.6821	0.3513	0.4215
Plasma cells	3.1512	0.7896	0.8614
B cells naive	2.3747	0.8822	0.8822

Normal tissue (GTEx) - randomised control			
**cell type**	**Kruskal-Wallis H statistic**	**p-value**	**q-value**
T cells regulatory (Tregs)	9.9580	0.1264	0.9050
Mast cells resting	7.9489	0.2419	0.9050
B cells naive	6.7233	0.3472	0.9050
Macrophages M2	5.9854	0.4248	0.9050
NK cells activated	5.1054	0.5304	0.9050
T cells follicular helper	4.9263	0.5533	0.9050
NK cells resting	4.5492	0.6028	0.9050
Plasma cells	4.5450	0.6033	0.9050
Monocytes	3.9382	0.6850	0.9134
Macrophages M1	3.0858	0.7980	0.9338
T cells CD8	2.6096	0.8560	0.9338
T cells CD4 memory resting	1.3737	0.9674	0.9674

Tumour (TCGA)			
**cell type**	**Kruskal-Wallis H statistic**	**p-value**	**q-value**
T cells CD8	63.3229	9.49E-12	1.14E-10
Macrophages M1	41.9146	1.91E-07	1.15E-06
B cells naive	30.2286	3.56E-05	1.42E-04
Monocytes	21.4546	0.0015	0.0046
T cells follicular helper	20.0210	0.0027	0.0066
NK cells activated	13.4627	0.0362	0.0725
T cells regulatory (Tregs)	9.9993	0.1247	0.2137
Mast cells resting	9.0086	0.1731	0.2506
T cells CD4 memory resting	8.7536	0.1879	0.2506
NK cells resting	7.3763	0.2874	0.3428
Macrophages M2	7.0718	0.3143	0.3428
Plasma cells	3.8243	0.7004	0.7004

Tumour (TCGA) - randomised control			
**cell type**	**Kruskal-Wallis H statistic**	**p-value**	**q-value**
Macrophages M1	9.3819	0.1532	0.9183
NK cells activated	5.8813	0.4366	0.9183
B cells naive	5.0502	0.5374	0.9183
T cells CD4 memory resting	4.7025	0.5825	0.9183
Plasma cells	3.9773	0.6797	0.9183
Monocytes	3.8543	0.6964	0.9183
Mast cells resting	3.0511	0.8024	0.9183
T cells follicular helper	2.9845	0.8108	0.9183
T cells CD8	2.7349	0.8413	0.9183
Macrophages M2	2.7144	0.8437	0.9183
NK cells resting	2.6418	0.8523	0.9183
T cells regulatory (Tregs)	2.0150	0.9183	0.9183

Supplementary Table S5. Immune cell level-APOBEC3 correlation data.

Uploaded separately.

Supplementary Table S6. Pairwise comparison p-values for Immune cell type proportion-APOBEC3 expression correlation analysis.

P-values were obtained in identical procedure as described in Supplementary Table S2 (and the Methods section). Here the adjusted p-values (Benjamini-Hochberg) are shown.

B-cell naïve TCGA	A3A	A3B	A3C	A3D	A3F	A3G
A3B	0.2156					
A3C	0.0765	0.0095				
A3D	0.0033	1.68E-04	0.1338			
A3F	0.0419	0.0042	0.4205	0.1955		
A3G	0.0037	2.62E-04	0.1313	0.4777	0.1920	
A3H	0.0322	0.0030	0.3786	0.2165	0.4621	0.2121

T cell CD8+ TCGA	A3A	A3B	A3C	A3D	A3F	A3G
A3B	0.4672					
A3C	0.0166	0.0169				
A3D	5.79E-06	4.65E-06	0.0159			
A3F	0.0030	0.0029	0.2885	0.0671		
A3G	6.48E-07	5.55E-07	0.0043	0.3373	0.0224	
A3H	5.82E-07	7.39E-07	0.0033	0.2980	0.0177	0.4633

Bcell naïve TCGA randomised	A3A	A3B	A3C	A3D	A3F	A3G
A3B	0.3706					
A3C	0.7494	0.4989				
A3D	0.4539	0.5607	0.4774			
A3F	0.4643	0.4834	0.4572	0.5115		
A3G	0.5436	0.5187	0.5063	0.4649	0.4235	
A3H	0.4124	0.5711	0.4845	0.4888	0.4961	0.4452

T cell CD8+ TCGA randomised	A3A	A3B	A3C	A3D	A3F	A3G
A3B	0.5651					
A3C	0.4442	0.4995				
A3D	0.5651	0.4683	0.4779			
A3F	0.5646	1.0000	0.6129	0.9350		
A3G	0.7634	0.5049	0.6682	0.5320	1.0000	
A3H	0.4676	0.6756	0.5401	0.6467	0.4685	1.0000

Macrophage M1 TCGA	A3A	A3B	A3C	A3D	A3F	A3G
A3B	0.0592					
A3C	0.2626	0.0175				
A3D	0.0633	0.0011	0.1821			
A3F	0.2414	0.0148	0.4589	0.2012		
A3G	1.72E-04	6.27E-08	0.0015	0.0225	0.0018	
A3H	0.0159	8.46E-05	0.0572	0.2487	0.0660	0.0927

T cell CD8+ GTEx	A3A	A3B	A3C	A3D	A3F	A3G
A3B	0.4025					
A3C	0.1853	0.2710				
A3D	0.0121	0.0155	0.0930			
A3F	0.1359	0.1814	0.4230	0.1325		
A3G	0.1126	0.1449	0.3127	0.1651	0.4168	
A3H	0.0232	0.0200	0.1088	0.4956	0.1458	0.1755

Macrophage M1 TCGA randomised	A3A	A3B	A3C	A3D	A3F	A3G
A3B	0.1690					
A3C	0.3675	0.1354				
A3D	0.3986	0.2534	0.2796			
A3F	0.2952	0.3771	0.1784	0.4009		
A3G	0.3831	0.2611	0.4944	0.2986	0.2024	
A3H	0.2664	0.3509	0.2081	0.3615	0.4436	0.2693

T cell CD8+ GTEx randomised	A3A	A3B	A3C	A3D	A3F	A3G
A3B	0.9793					
A3C	0.6872	0.4669				
A3D	1.0000	0.4839	0.5841			
A3F	0.7898	0.4922	0.4540	0.5568		
A3G	1.0000	0.5267	0.7224	0.4767	0.6490	
A3H	0.5252	0.5851	0.4943	0.8405	0.4988	1.0000

**Monocyte GTEx**	A3A	A3B	A3C	A3D	A3F	A3G
A3B	0.0286					
A3C	0.0050	0.4124				
A3D	0.0017	0.2569	0.4526			
A3F	0.0116	0.4307	0.4278	0.4153		
A3G	0.0058	0.4078	0.4693	0.4293	0.4177	
A3H	0.0190	0.4382	0.4230	0.3233	0.4494	0.4491

**Monocyte GTEx randomised**	A3A	A3B	A3C	A3D	A3F	A3G
A3B	0.4509					
A3C	0.4968	0.4181				
A3D	0.7105	0.7289	0.5146			
A3F	0.5015	0.5635	0.4754	0.4461		
A3G	0.8442	0.9463	0.8522	0.5127	0.5494	
A3H	0.5363	0.4735	0.4824	0.4464	0.4698	0.6068

Supplementary Table S7. Tumour/tissue-specific ratio.

Uploaded separately.

Supplementary Table S8. Pairwise comparison p-values for the tumour/tissue-specific ratio analysis.

P-values were obtained in identical procedure as described in Supplementary Tables S2 and S4 (and the Methods section). Adjusted p-values (Benjamini-Hochberg) are shown.

TCGA	A3A	A3B	A3C	A3D	A3F	A3G
A3B	3.13E-04					
A3C	0.1214	0.0155				
A3D	0.4625	0.0003	0.1260			
A3F	0.0161	0.1203	0.1539	0.0163		
A3G	0.3538	0.0012	0.2051	0.3722	0.0390	
A3H	0.0969	4.67E-07	0.0055	0.0868	2.11E-04	0.0412

GTEx	A3A	A3B	A3C	A3D	A3F	A3G
A3B	0.0733					
A3C	2.07E-04	0.0240				
A3D	0.0978	0.4181	0.0172			
A3F	0.0022	0.0829	0.3025	0.0624		
A3G	0.0848	0.4588	0.0215	0.4399	0.0706	
A3H	0.2072	0.3084	0.0052	0.3760	0.0255	0.3337

TCGA case-randomised	A3A	A3B	A3C	A3D	A3F	A3G
A3B	0.1849					
A3C	0.0039	0.0277				
A3D	0.0397	0.2345	0.1435			
A3F	0.0064	0.0647	0.3481	0.2399		
A3G	0.0079	0.0673	0.3396	0.2458	0.4899	
A3H	0.4932	0.1948	0.0026	0.0426	0.0055	0.0061

GTEx case-randomised	A3A	A3B	A3C	A3D	A3F	A3G
A3B	0.2471					
A3C	4.37E-04	0.0030				
A3D	0.0533	0.2133	0.0302			
A3F	0.0019	0.0111	0.3310	0.0847		
A3G	0.0031	0.0173	0.2548	0.1284	0.4207	
A3H	0.2410	0.4760	0.0029	0.2186	0.0113	0.0181

TCGA case-randomised & gene-randomised	A3A	A3B	A3C	A3D	A3F	A3G
A3B	0.5952					
A3C	0.6439	0.4463				
A3D	0.4697	0.4785	0.4977			
A3F	0.4931	0.4407	0.4866	0.4579		
A3G	0.4704	0.4875	0.5075	0.4213	0.4570	
A3H	0.4763	0.5579	0.9014	0.4682	0.4792	0.5350

GTEx case-randomised and gene-randomised	A3A	A3B	A3C	A3D	A3F	A3G
A3B	1.0000					
A3C	1.0000	0.6934				
A3D	0.5522	0.8346	1.0000			
A3F	0.7359	0.5903	0.9984	0.6255		
A3G	1.0000	0.5592	0.5264	1.0000	0.7697	
A3H	1.0000	0.6570	0.4911	1.0000	0.9074	0.5094

Supplementary Table S9. Cell-type proportion estimates and RESPECTEx analysis of the breast cancer cohort GSE79688

Uploaded separately.

Supplementary Table S10. APOBEC3 gene co-expression partners across cohorts.

Uploaded separately.

Supplementary Table S11. Statistics of APOBEC3 gene co-expression partners across cohorts and overlaps with bootstrapping samples.

For each bootstrapping sample the fraction of co-expression partners were taken; the median and lower/upper quartiles of this quantity are given here. See methods for more details.

Supplementary Table S12. Gene set enrichment analysis of APOBEC3 gene co-expression partners

These tables include GSEA results of both GO gene sets (subfolder GSEA_GO) and curated gene sets.
Uploaded separately.

Supplementary Table S13. Distinguishing genes for APOBEC3s

Uploaded separately.

Supplementary Table S11

		APOBEC3A			APOBEC3B			APOBEC3C			APOBEC3D			APOBEC3F			APOBEC3G			APOBEC3H		
cohort type	cohort	No. of coexpression partners	median	lower/upper quartiles	No. of coexpression partners	median	lower/upper quartiles	No. of coexpression partners	median	lower/upper quartiles	No. of coexpression partners	median	lower/upper quartiles	No. of coexpression partners	median	lower/upper quartiles	No. of coexpression partners	median	lower/upper quartiles	No. of coexpression partners	median	lower/upper quartiles
Cancer cell line	CCLE.BLCA	462	0.198	(0.142 - 0.230)	501	0.186	(0.140 - 0.219)	494	0.230	(0.175 - 0.273)	435	0.214	(0.180 - 0.262)	503	0.206	(0.164 - 0.236)	467	0.199	(0.152 - 0.259)	446	0.244	(0.208 - 0.297)
Cancer cell line	CCLE.BRCA	360	0.122	(0.073 - 0.165)	507	0.131	(0.099 - 0.164)	380	0.264	(0.170 - 0.320)	383	0.146	(0.109 - 0.186)	419	0.175	(0.125 - 0.242)	323	0.133	(0.081 - 0.202)	502	0.120	(0.086 - 0.148)
Cancer cell line	CCLE.CESC	478	0.183	(0.147 - 0.219)	506	0.204	(0.170 - 0.249)	496	0.235	(0.188 - 0.274)	479	0.221	(0.176 - 0.281)	485	0.229	(0.182 - 0.276)	469	0.205	(0.166 - 0.240)	475	0.206	(0.172 - 0.245)
Cancer cell line	CCLE.COAD	469	0.124	(0.091 - 0.150)	456	0.120	(0.083 - 0.159)	459	0.233	(0.164 - 0.281)	483	0.175	(0.122 - 0.253)	446	0.187	(0.136 - 0.247)	419	0.148	(0.106 - 0.211)	465	0.107	(0.076 - 0.132)
Cancer cell line	CCLE.DLBC	315	0.102	(0.076 - 0.137)	334	0.181	(0.127 - 0.239)	360	0.124	(0.077 - 0.166)	423	0.136	(0.089 - 0.165)	390	0.136	(0.084 - 0.183)	474	0.150	(0.113 - 0.189)	344	0.150	(0.105 - 0.208)
Cancer cell line	CCLE.ESCA	504	0.207	(0.163 - 0.248)	489	0.193	(0.157 - 0.237)	516	0.195	(0.161 - 0.240)	468	0.186	(0.149 - 0.222)	434	0.225	(0.172 - 0.259)	491	0.206	(0.164 - 0.248)	462	0.208	(0.155 - 0.270)
Cancer cell line	CCLE.HNSC	409	0.186	(0.152 - 0.226)	490	0.193	(0.166 - 0.240)	523	0.158	(0.128 - 0.202)	502	0.153	(0.127 - 0.189)	505	0.196	(0.144 - 0.238)	513	0.230	(0.171 - 0.289)	499	0.174	(0.135 - 0.219)
Cancer cell line	CCLE.KIPAN	427	0.175	(0.145 - 0.221)	424	0.202	(0.167 - 0.245)	513	0.267	(0.225 - 0.308)	419	0.224	(0.178 - 0.262)	464	0.220	(0.190 - 0.268)	407	0.268	(0.227 - 0.319)	534	0.243	(0.212 - 0.281)
Cancer cell line	CCLE.LAML	438	0.170	(0.139 - 0.221)	440	0.183	(0.154 - 0.229)	424	0.253	(0.171 - 0.319)	386	0.330	(0.277 - 0.373)	412	0.301	(0.251 - 0.356)	449	0.221	(0.165 - 0.277)	494	0.188	(0.164 - 0.231)
Cancer cell line	CCLE.LGG	510	0.124	(0.092 - 0.159)	239	0.135	(0.086 - 0.188)	186	0.123	(0.072 - 0.178)	331	0.089	(0.058 - 0.123)	212	0.123	(0.074 - 0.175)	197	0.121	(0.085 - 0.167)	507	0.106	(0.086 - 0.140)
Cancer cell line	CCLE.LIHC	415	0.200	(0.147 - 0.242)	463	0.204	(0.169 - 0.237)	436	0.203	(0.166 - 0.259)	497	0.164	(0.145 - 0.199)	437	0.165	(0.132 - 0.205)	454	0.188	(0.146 - 0.214)	474	0.169	(0.121 - 0.215)
Cancer cell line	CCLE.LUAD	508	0.105	(0.087 - 0.127)	502	0.119	(0.095 - 0.143)	525	0.148	(0.103 - 0.202)	545	0.165	(0.117 - 0.225)	504	0.140	(0.111 - 0.173)	495	0.134	(0.099 - 0.193)	491	0.102	(0.082 - 0.129)
Cancer cell line	CCLE.LUSC	492	0.122	(0.094 - 0.147)	462	0.118	(0.081 - 0.152)	486	0.172	(0.125 - 0.220)	469	0.119	(0.088 - 0.162)	465	0.139	(0.099 - 0.179)	496	0.166	(0.130 - 0.230)	487	0.110	(0.087 - 0.139)
Cancer cell line	CCLE.OV	471	0.144	(0.102 - 0.195)	477	0.167	(0.133 - 0.202)	455	0.165	(0.128 - 0.194)	464	0.154	(0.114 - 0.188)	515	0.166	(0.126 - 0.206)	499	0.153	(0.114 - 0.192)	494	0.133	(0.105 - 0.177)
Cancer cell line	CCLE.PAAD	530	0.149	(0.123 - 0.176)	511	0.125	(0.099 - 0.165)	518	0.148	(0.122 - 0.182)	514	0.147	(0.123 - 0.181)	502	0.126	(0.107 - 0.159)	502	0.130	(0.105 - 0.178)	513	0.140	(0.112 - 0.173)
Cancer cell line	CCLE.SARC	197	0.114	(0.076 - 0.175)	520	0.230	(0.165 - 0.274)	393	0.154	(0.121 - 0.195)	379	0.150	(0.093 - 0.193)	516	0.147	(0.109 - 0.190)	552	0.152	(0.118 - 0.184)	401	0.142	(0.101 - 0.175)
Cancer cell line	CCLE.SKCM	476	0.130	(0.094 - 0.162)	233	0.100	(0.069 - 0.135)	462	0.176	(0.113 - 0.208)	492	0.126	(0.088 - 0.148)	479	0.147	(0.121 - 0.195)	493	0.149	(0.117 - 0.176)	397	0.121	(0.093 - 0.159)
Cancer cell line	CCLE.STAD	489	0.152	(0.125 - 0.187)	522	0.151	(0.120 - 0.173)	460	0.153	(0.118 - 0.198)	434	0.154	(0.113 - 0.183)	439	0.164	(0.125 - 0.209)	432	0.173	(0.129 - 0.228)	462	0.120	(0.092 - 0.157)
Cancer cell line	CCLE.THCA	423	0.586	(0.483 - 0.628)	354	0.537	(0.485 - 0.576)	479	0.506	(0.433 - 0.619)	408	0.556	(0.511 - 0.633)	430	0.540	(0.480 - 0.626)	378	0.557	(0.481 - 0.640)	214	0.549	(0.479 - 0.720)
Normal tissue	GTEx.Adrenal_Gland	553	0.110	(0.076 - 0.138)	575	0.221	(0.175 - 0.301)	498	0.204	(0.152 - 0.266)	729	0.145	(0.097 - 0.181)	480	0.141	(0.096 - 0.184)	682	0.148	(0.104 - 0.203)	634	0.143	(0.111 - 0.182)
Normal tissue	GTEx.Bladder	562	0.739	(0.599 - 0.776)	709	0.668	(0.589 - 0.786)	0	NA		0	NA		0	NA		0	NA		0	NA	
Normal tissue	GTEx.Blood	0	NA		225	0.258	(0.152 - 0.369)	0	NA		0	NA		0	NA		0	NA		0	NA	
Normal tissue	GTEx.Brain	345	0.483	(0.426 - 0.539)	165	0.320	(0.234 - 0.377)	8	0.429	(0.246 - 0.690)	115	0.425	(0.359 - 0.535)	44	0.404	(0.312 - 0.544)	15	0.312	(0.205 - 0.500)	395	0.590	(0.542 - 0.650)
Normal tissue	GTEx.Breast	184	0.121	(0.092 - 0.176)	562	0.158	(0.122 - 0.204)	172	0.173	(0.130 - 0.237)	155	0.161	(0.114 - 0.228)	75	0.089	(0.060 - 0.138)	163	0.150	(0.110 - 0.206)	459	0.134	(0.077 - 0.185)
Normal tissue	GTEx.Cervix_Uteri	379	0.685	(0.610 - 0.823)	213	0.821	(0.606 - 0.881)	657	0.740	(0.590 - 0.840)	480	0.651	(0.548 - 0.693)	528	0.643	(0.630 - 0.829)	737	0.811	(0.626 - 0.827)	497	0.683	(0.585 - 0.800)
Normal tissue	GTEx.Colon	9	0.065	(0.024 - 0.126)	0	NA		1	0.002	(0.000 - 0.011)	2	0.009	(0.000 - 0.027)	49	0.070	(0.042 - 0.112)	142	0.141	(0.111 - 0.182)	218	0.187	(0.118 - 0.305)
Normal tissue	GTEx.Esophagus	0	NA		0	NA		402	0.446	(0.350 - 0.520)	476	0.485	(0.412 - 0.572)	663	0.357	(0.286 - 0.407)	624	0.454	(0.403 - 0.527)	188	0.149	(0.095 - 0.224)
Normal tissue	GTEx.Kidney	570	0.244	(0.181 - 0.318)	501	0.202	(0.134 - 0.262)	421	0.270	(0.191 - 0.350)	604	0.282	(0.214 - 0.359)	624	0.189	(0.141 - 0.271)	411	0.274	(0.177 - 0.351)	555	0.205	(0.136 - 0.278)
Normal tissue	GTEx.Liver	267	0.082	(0.038 - 0.130)	309	0.088	(0.042 - 0.151)	763	0.311	(0.238 - 0.381)	321	0.114	(0.077 - 0.162)	577	0.099	(0.067 - 0.127)	631	0.166	(0.104 - 0.229)	469	0.084	(0.050 - 0.122)
Normal tissue	GTEx.Lung	673	0.394	(0.364 - 0.441)	483	0.277	(0.201 - 0.332)	632	0.357	(0.300 - 0.435)	592	0.439	(0.398 - 0.507)	702	0.337	(0.282 - 0.403)	664	0.456	(0.382 - 0.506)	387	0.281	(0.227 - 0.337)
Normal tissue	GTEx.Muscle	448	0.194	(0.139 - 0.253)	447	0.090	(0.055 - 0.138)	397	0.459	(0.395 - 0.508)	596	0.272	(0.214 - 0.320)	577	0.212	(0.129 - 0.300)	446	0.274	(0.204 - 0.356)	576	0.093	(0.065 - 0.125)
Normal tissue	GTEx.Ovary	606	0.071	(0.045 - 0.122)	635	0.120	(0.088 - 0.159)	706	0.196	(0.141 - 0.252)	409	0.146	(0.095 - 0.193)	645	0.172	(0.122 - 0.242)	490	0.125	(0.077 - 0.168)	684	0.104	(0.076 - 0.135)
Normal tissue	GTEx.Pancreas	672	0.178	(0.080 - 0.243)	767	0.202	(0.142 - 0.268)	650	0.227	(0.161 - 0.286)	493	0.139	(0.087 - 0.195)	612	0.131	(0.076 - 0.200)	642	0.206	(0.128 - 0.292)	756	0.103	(0.066 - 0.132)
Normal tissue	GTEx.Prostate	623	0.096	(0.059 - 0.146)	688	0.101	(0.056 - 0.138)	689	0.174	(0.133 - 0.217)	528	0.192	(0.143 - 0.261)	516	0.168	(0.121 - 0.202)	561	0.139	(0.101 - 0.203)	436	0.101	(0.071 - 0.135)
Normal tissue	GTEx.Skin	270	0.222	(0.158 - 0.335)	8	0.162	(0.084 - 0.333)	0	NA	(NA - NA)	0	NA	(NA - NA)	0	NA	(NA - NA)	0	NA	(NA - NA)	0	NA	(NA - NA)
Normal tissue	GTEx.Stomach	453	0.262	(0.179 - 0.360)	246	0.379	(0.263 - 0.569)	543	0.217	(0.167 - 0.280)	6	0.023	(0.013 - 0.043)	703	0.245	(0.201 - 0.290)	495	0.285	(0.233 - 0.330)	28	0.020	(0.007 - 0.046)
Normal tissue	GTEx.Testis	384	0.071	(0.049 - 0.114)	1	0.000	(0.000 - 0.000)	13	0.026	(0.005 - 0.037)	49	0.041	(0.022 - 0.062)	79	0.073	(0.050 - 0.134)	168	0.183	(0.096 - 0.259)	105	0.064	(0.022 - 0.110)
Normal tissue	GTEx.Thyroid	619	0.245	(0.181 - 0.319)	740	0.179	(0.135 - 0.233)	628	0.354	(0.284 - 0.404)	541	0.345	(0.291 - 0.393)	687	0.235	(0.176 - 0.291)	595	0.339	(0.293 - 0.388)	524	0.239	(0.171 - 0.329)
Normal tissue	GTEx.Uterus	739	0.138	(0.087 - 0.182)	675	0.129	(0.093 - 0.175)	597	0.179	(0.126 - 0.233)	169	0.098	(0.066 - 0.136)	627	0.136	(0.099 - 0.197)	389	0.106	(0.053 - 0.146)	287	0.049	(0.026 - 0.093)
Tumour	TCGA.ACC	546	0.220	(0.100 - 0.312)	518	0.267	(0.144 - 0.345)	521	0.131	(0.092 - 0.175)	484	0.153	(0.113 - 0.196)	519	0.142	(0.100 - 0.195)	610	0.226	(0.133 - 0.403)	511	0.113	(0.073 - 0.163)
Tumour	TCGA.BLCA	654	0.244	(0.161 - 0.297)	682	0.255	(0.188 - 0.307)	621	0.262	(0.221 - 0.310)	579	0.217	(0.172 - 0.263)	548	0.255	(0.213 - 0.326)	675	0.237	(0.194 - 0.295)	476	0.208	(0.175 - 0.239)
Tumour	TCGA.BRCA	549	0.616	(0.564 - 0.667)	591	0.641	(0.605 - 0.677)	763	0.790	(0.759 - 0.812)	825	0.797	(0.769 - 0.833)	611	0.500	(0.412 - 0.553)	821	0.873	(0.856 - 0.891)	742	0.847	(0.824 - 0.867)
Tumour	TCGA.CESC	655	0.418	(0.375 - 0.468)	671	0.205	(0.160 - 0.250)	524	0.286	(0.234 - 0.333)	731	0.435	(0.311 - 0.512)	581	0.227	(0.184 - 0.261)	795	0.594	(0.485 - 0.659)	703	0.540	(0.402 - 0.596)
Tumour	TCGA.COADREAD	650	0.273	(0.234 - 0.349)	582	0.177	(0.127 - 0.225)	451	0.174	(0.151 - 0.208)	756	0.370	(0.284 - 0.449)	707	0.299	(0.239 - 0.361)	694	0.680	(0.621 - 0.724)	728	0.556	(0.453 - 0.634)
Tumour	TCGA.DLBC	736	0.194	(0.120 - 0.280)	513	0.143	(0.108 - 0.179)	173	0.073	(0.039 - 0.113)	411	0.142	(0.101 - 0.188)	455	0.152	(0.111 - 0.201)	628	0.217	(0.150 - 0.288)	203	0.070	(0.035 - 0.090)
Tumour	TCGA.ESCA	683	0.391	(0.317 - 0.435)	490	0.106	(0.067 - 0.145)	633	0.346	(0.275 - 0.402)	706	0.130	(0.086 - 0.190)	633	0.142	(0.104 - 0.228)	590	0.200	(0.148 - 0.268)	677	0.221	(0.123 - 0.315)
Tumour	TCGA.GBMLGG	206	0.193	(0.145 - 0.249)	260	0.458	(0.378 - 0.542)	221	0.570	(0.479 - 0.650)	119	0.410	(0.329 - 0.501)	160	0.510	(0.410 - 0.634)	254	0.687	(0.584 - 0.785)	421	0.533	(0.458 - 0.618)
Tumour	TCGA.HNSC	721	0.483	(0.416 - 0.527)	678	0.332	(0.245 - 0.398)	508	0.342	(0.267 - 0.380)	774	0.627	(0.543 - 0.673)	719	0.463	(0.392 - 0.527)	821	0.715	(0.676 - 0.743)	693	0.622	(0.573 - 0.660)
Tumour	TCGA.KIPAN	433	0.301	(0.271 - 0.349)	475	0.473	(0.424 - 0.528)	432	0.653	(0.570 - 0.707)	680	0.626	(0.563 - 0.688)	530	0.493	(0.428 - 0.553)	459	0.795	(0.742 - 0.833)	569	0.706	(0.661 - 0.748)
Tumour	TCGA.LAML	561	0.446	(0.361 - 0.563)	551	0.305	(0.228 - 0.392)	448	0.194	(0.145 - 0.243)	549	0.207	(0.156 - 0.277)	556	0.244	(0.184 - 0.312)	679	0.286	(0.220 - 0.349)	481	0.181	(0.135 - 0.227)
Tumour	TCGA.LIHC	567	0.161	(0.109 - 0.246)	621	0.232	(0.155 - 0.288)	661	0.623	(0.572 - 0.668)	677	0.533	(0.413 - 0.596)	451	0.173	(0.128 - 0.212)	701	0.652	(0.576 - 0.710)	630	0.463	(0.374 - 0.539)
Tumour	TCGA.LUAD	575	0.278	(0.245 - 0.335)	649	0.421	(0.348 - 0.485)	680	0.461	(0.370 - 0.525)	808	0.549	(0.447 - 0.625)	758	0.352	(0.256 - 0.434)	826	0.695	(0.647 - 0.745)	782	0.553	(0.473 - 0.614)
Tumour	TCGA.LUSC	652	0.343	(0.300 - 0.385)	531	0.241	(0.195 - 0.287)	639	0.234	(0.177 - 0.271)	837	0.452	(0.317 - 0.537)	674	0.211	(0.179 - 0.242)	802	0.580	(0.459 - 0.653)	841	0.423	(0.337 - 0.491)
Tumour	TCGA.OV	651	0.293	(0.211 - 0.376)	727	0.183	(0.128 - 0.242)	812	0.243	(0.144 - 0.362)	847	0.350	(0.264 - 0.484)	695	0.180	(0.129 - 0.238)	828	0.476	(0.351 - 0.592)	694	0.527	(0.369 - 0.602)
Tumour	TCGA.PAAD	709	0.140	(0.091 - 0.192)	531	0.169	(0.107 - 0.257)	507	0.160	(0.112 - 0.221)	747	0.188	(0.143 - 0.292)	538	0.133	(0.089 - 0.179)	779	0.237	(0.140 - 0.419)	785	0.263	(0.166 - 0.388)
Tumour	TCGA.PCPG	674	0.164	(0.096 - 0.253)	545	0.209	(0.173 - 0.248)	565	0.160	(0.104 - 0.266)	394	0.082	(0.059 - 0.128)	452	0.174	(0.134 - 0.228)	501	0.113	(0.074 - 0.152)	485	0.172	(0.131 - 0.267)
Tumour	TCGA.PRAD	682	0.311	(0.251 - 0.383)	724	0.513	(0.435 - 0.582)	525	0.657	(0.601 - 0.725)	713	0.620	(0.559 - 0.677)	712	0.362	(0.287 - 0.403)	702	0.647	(0.608 - 0.709)	620	0.475	(0.399 - 0.555)
Tumour	TCGA.SARC	492	0.183	(0.115 - 0.285)	549	0.317	(0.239 - 0.390)	670	0.302	(0.212 - 0.395)	692	0.438	(0.325 - 0.534)	570	0.210	(0.158 - 0.256)	728	0.507	(0.425 - 0.584)	729	0.678	(0.590 - 0.740)
Tumour	TCGA.SKCM	338	0.064	(0.044 - 0.103)	568	0.096	(0.072 - 0.127)	511	0.106	(0.068 - 0.142)	645	0.169	(0.102 - 0.284)	536	0.123	(0.076 - 0.214)	771	0.310	(0.171 - 0.430)	600	0.162	(0.099 - 0.269)
Tumour	TCGA.STAD	556	0.275	(0.227 - 0.314)	431	0.171	(0.112 - 0.246)	597	0.454	(0.324 - 0.550)	843	0.609	(0.557 - 0.666)	817	0.417	(0.324 - 0.502)	771	0.716	(0.682 - 0.754)	763	0.640	(0.593 - 0.682)
Tumour	TCGA.TGCT	421	0.217	(0.133 - 0.302)	71	0.039	(0.023 - 0.061)	355	0.205	(0.168 - 0.259)	448	0.299	(0.251 - 0.392)	206	0.203	(0.161 - 0.318)	452	0.293	(0.250 - 0.357)	320	0.220	(0.153 - 0.294)
Tumour	TCGA.THCA	540	0.348	(0.270 - 0.402)	558	0.375	(0.300 - 0.415)	337	0.326	(0.257 - 0.374)	738	0.727	(0.665 - 0.777)	538	0.497	(0.396 - 0.575)	686	0.767	(0.717 - 0.806)	325	0.368	(0.297 - 0.432)
Tumour	TCGA.UCEC	549	0.154	(0.115 - 0.195)	613	0.154	(0.107 - 0.203)	659	0.163	(0.104 - 0.243)	586	0.270	(0.186 - 0.356)	367	0.113	(0.076 - 0.155)	727	0.381	(0.251 - 0.494)	564	0.223	(0.112 - 0.344)
Tumour	TCGA.UCS	592	0.130	(0.105 - 0.171)	548	0.120	(0.084 - 0.151)	696	0.147	(0.112 - 0.175)	645	0.154	(0.091 - 0.215)	549	0.121	(0.079 - 0.162)	671	0.148	(0.099 - 0.209)	596	0.134	(0.085 - 0.200)

SUPPLEMENTARY FIGURES

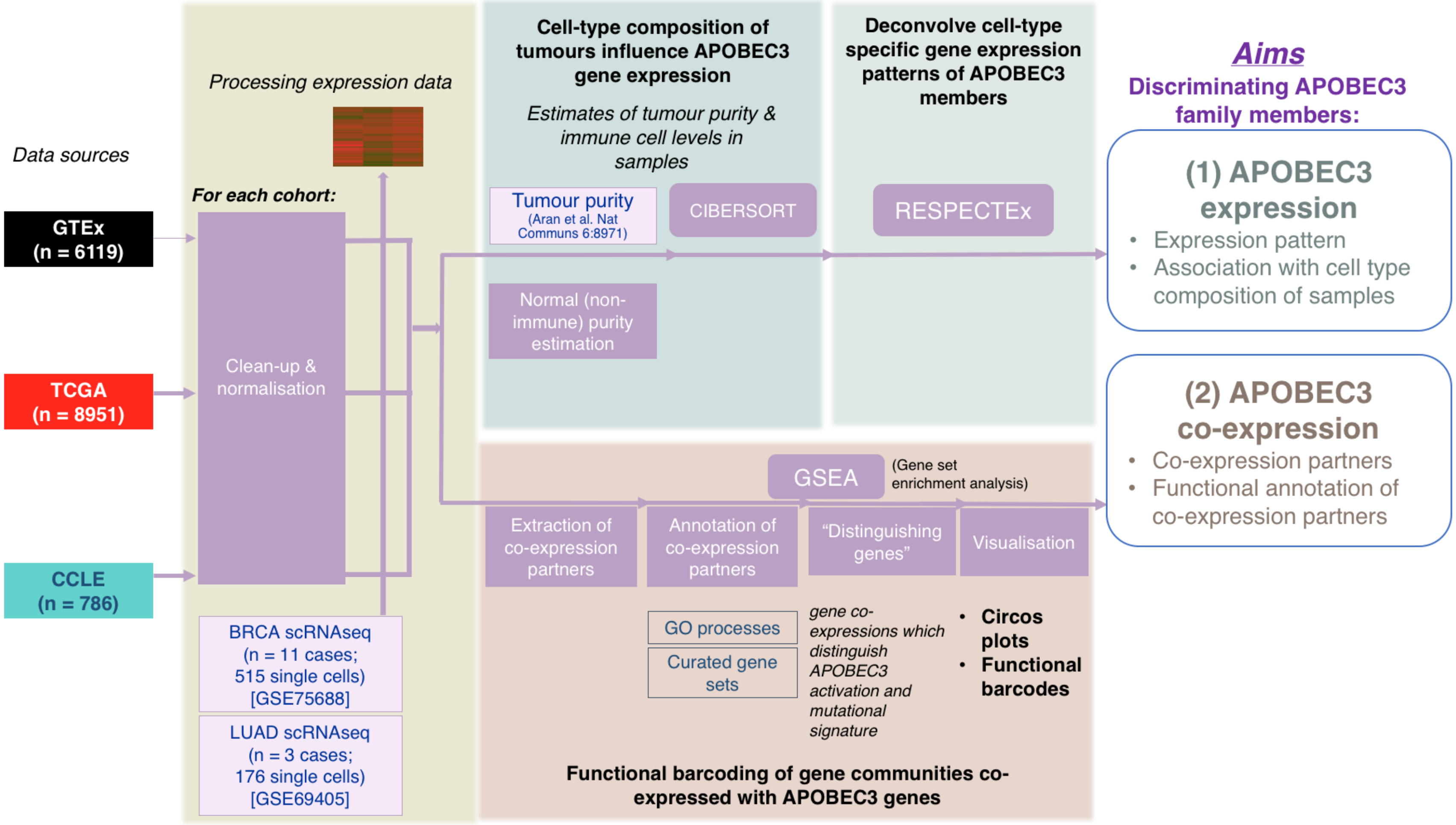

Supplementary Figure S1. Summary flowchart for this study.

Supplementary Figure S2

ESTIMATE purity

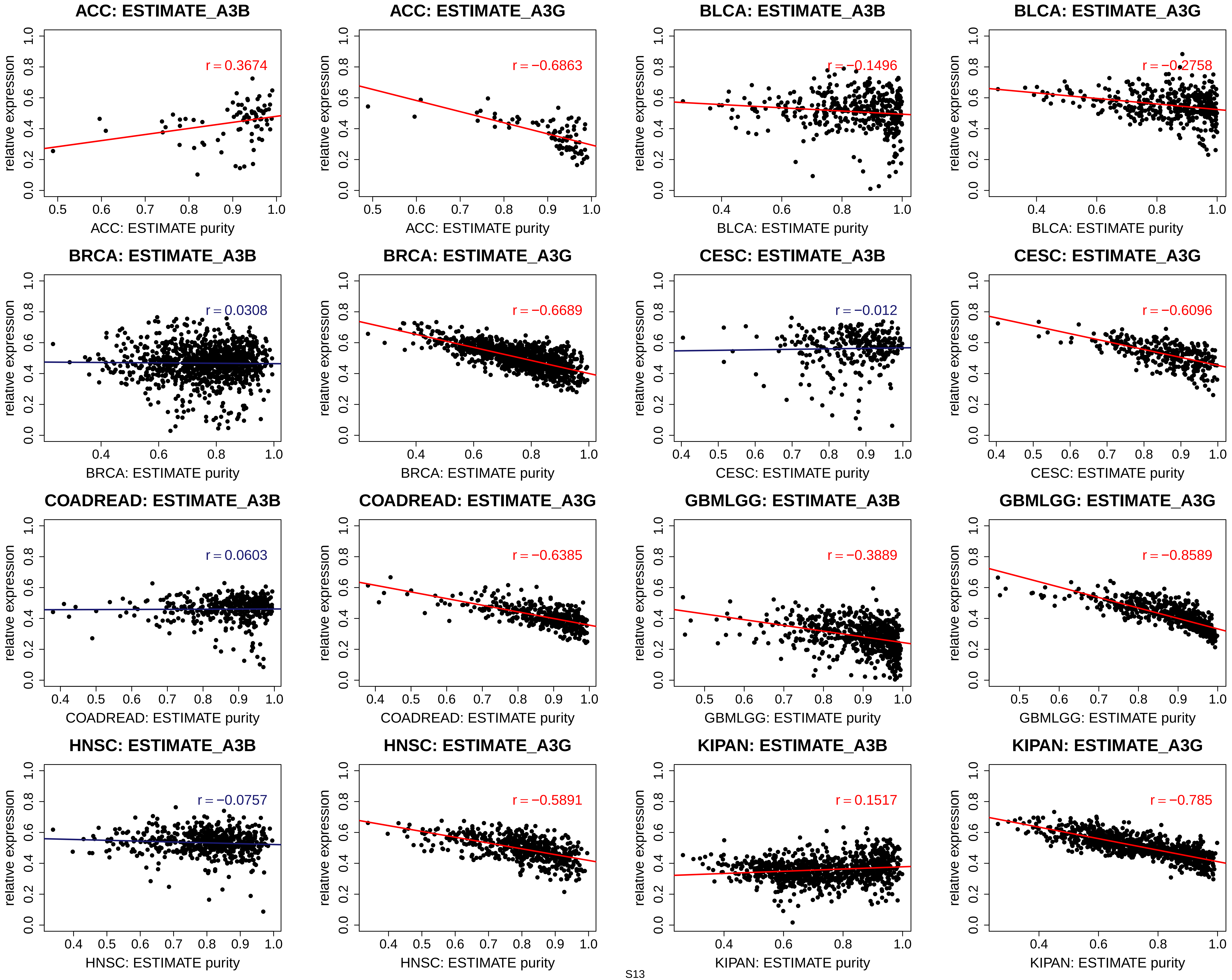

Supplementary Figure S2

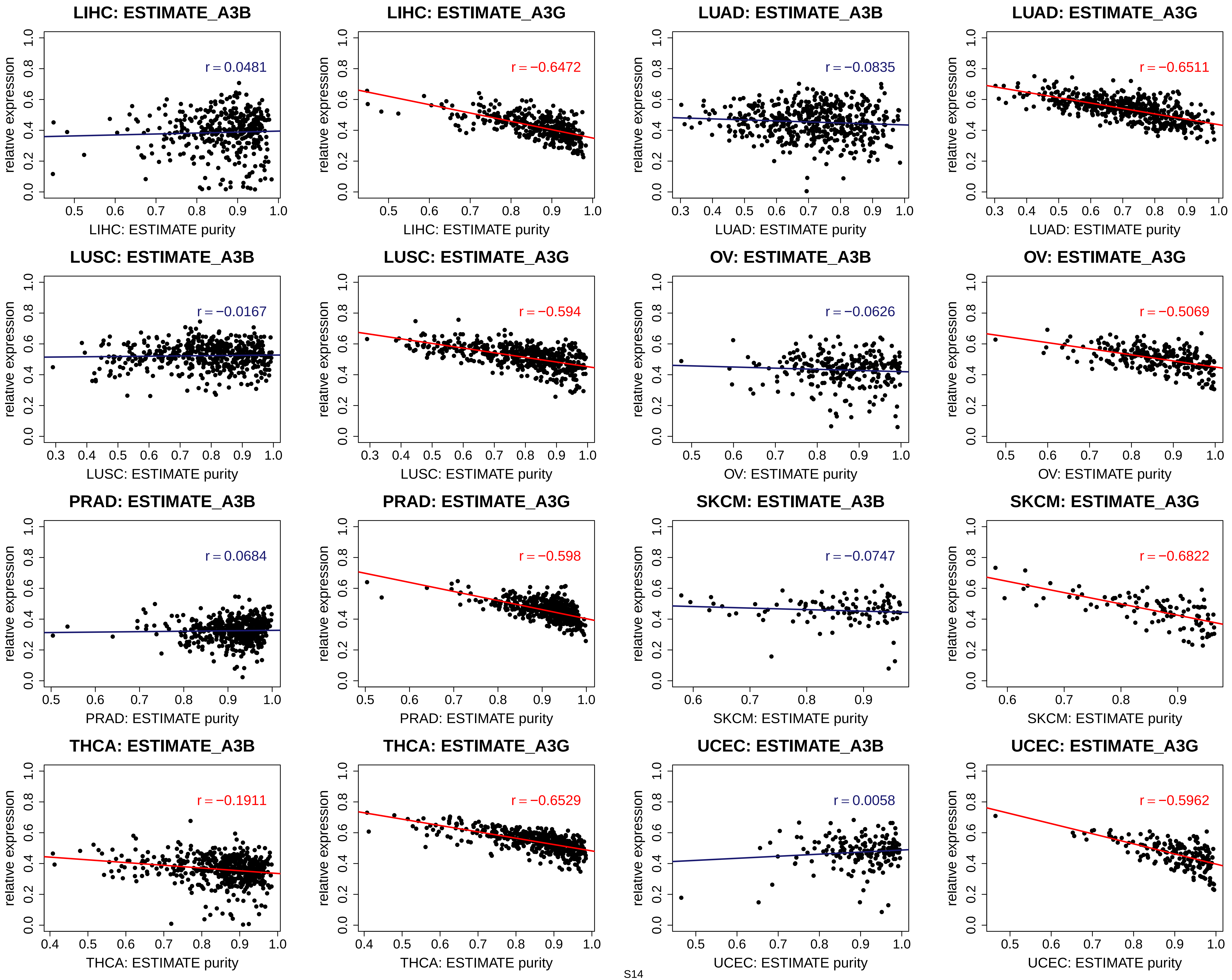

Supplementary Figure S2

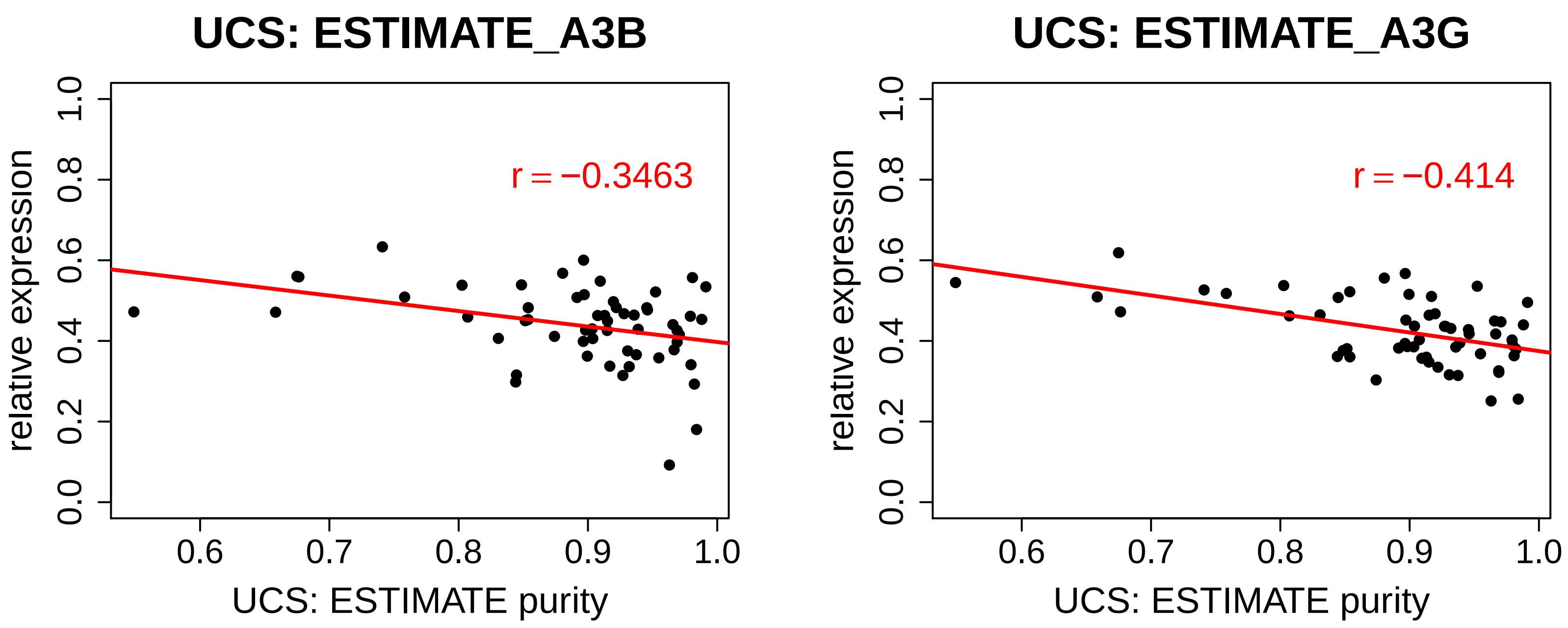

Supplementary Figure S2

CPE (Composite purity estimate)

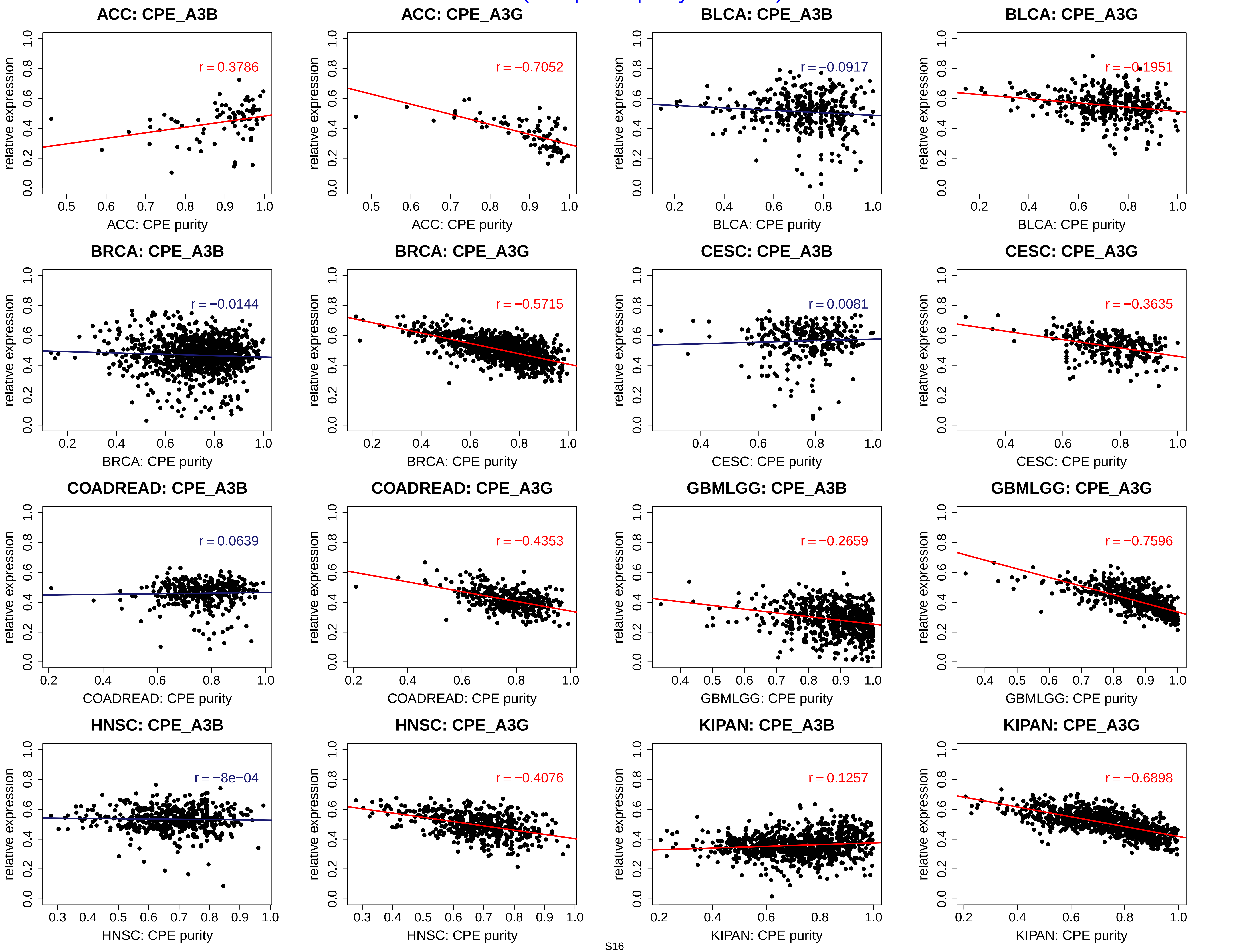

Supplementary Figure S2

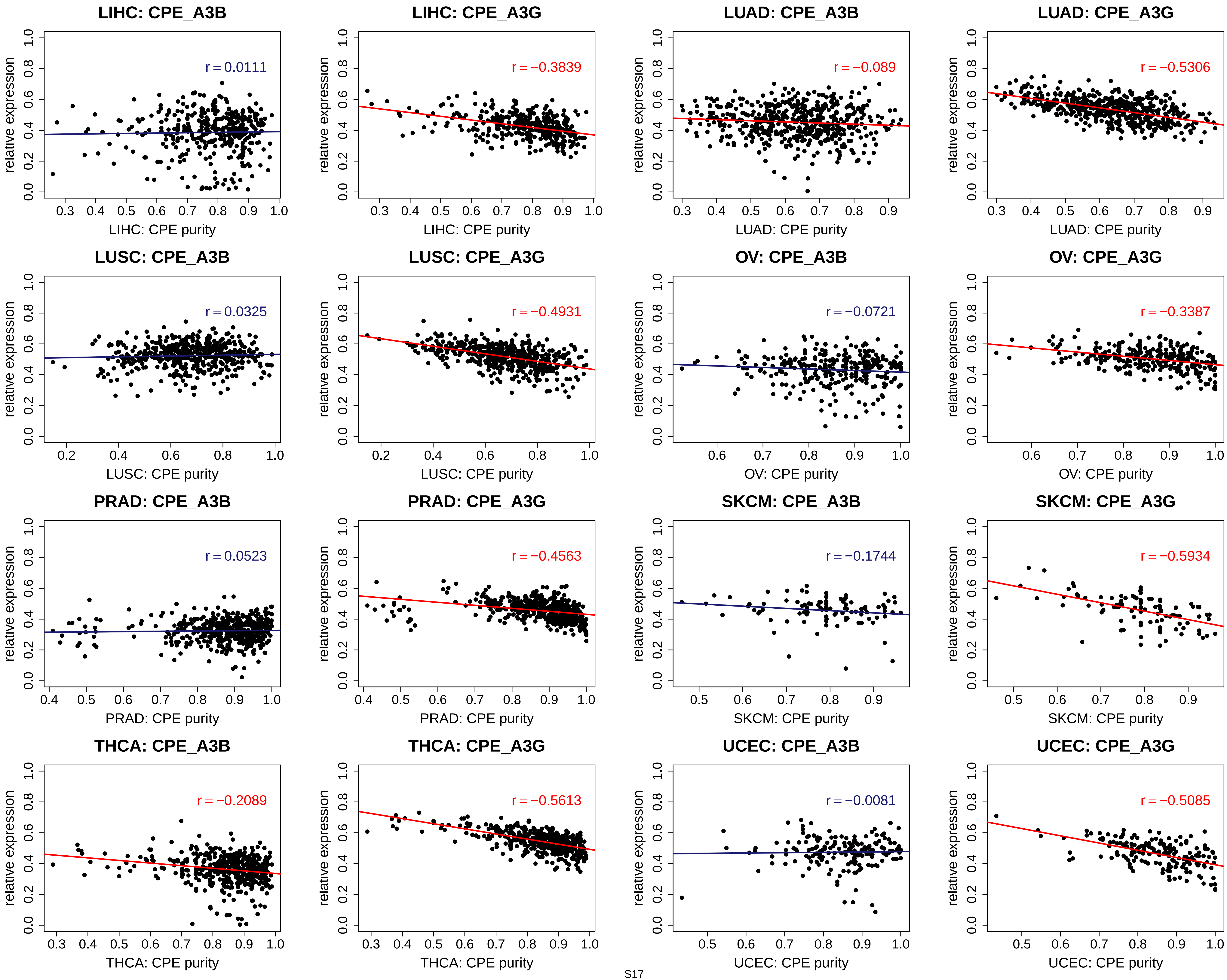

Supplementary Figure S2

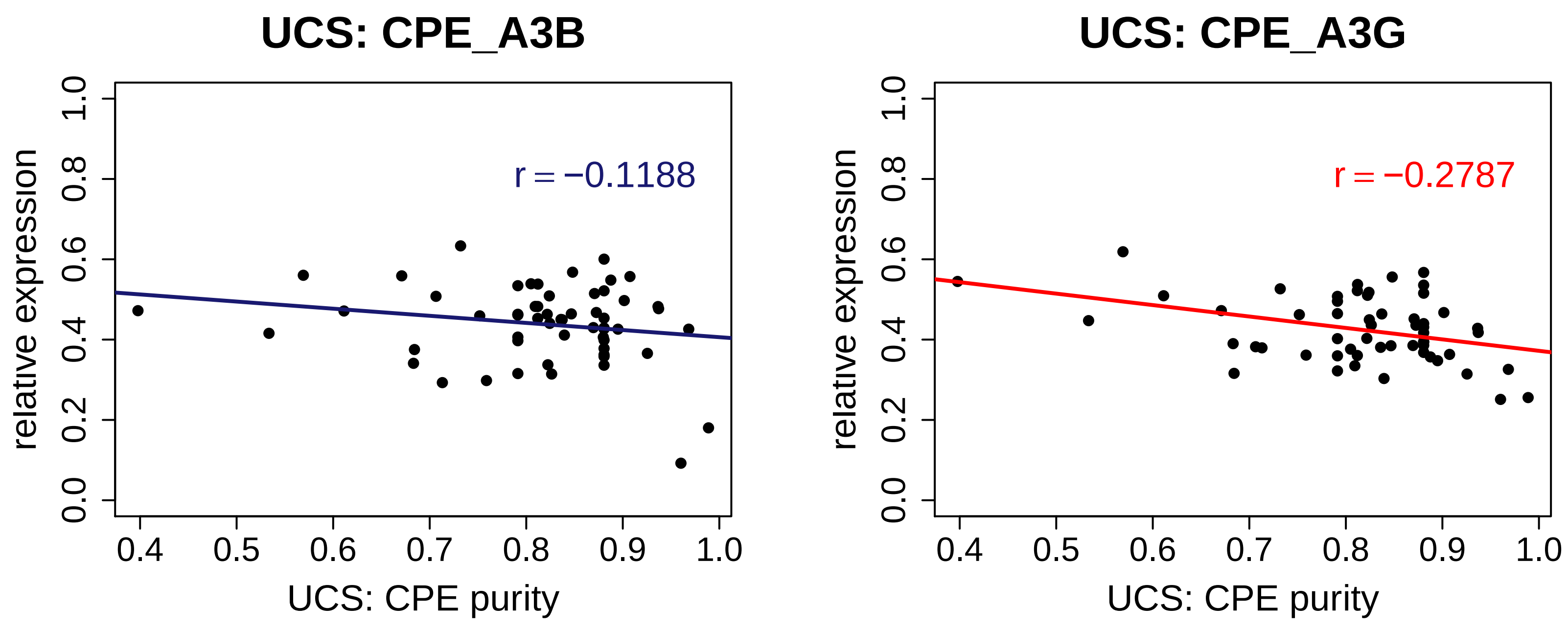

Supplementary Figure S2

IHC-based purity

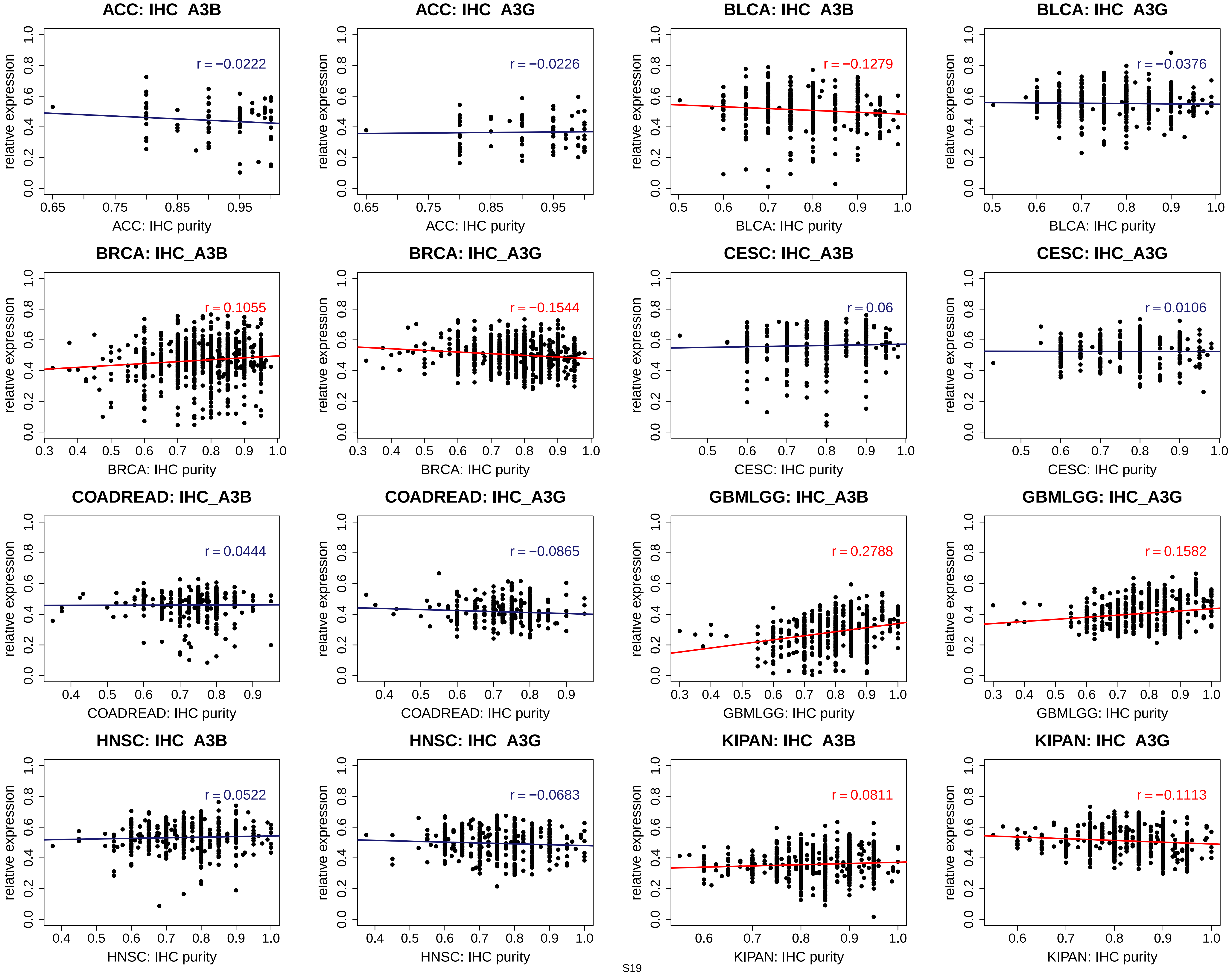

Supplementary Figure S2

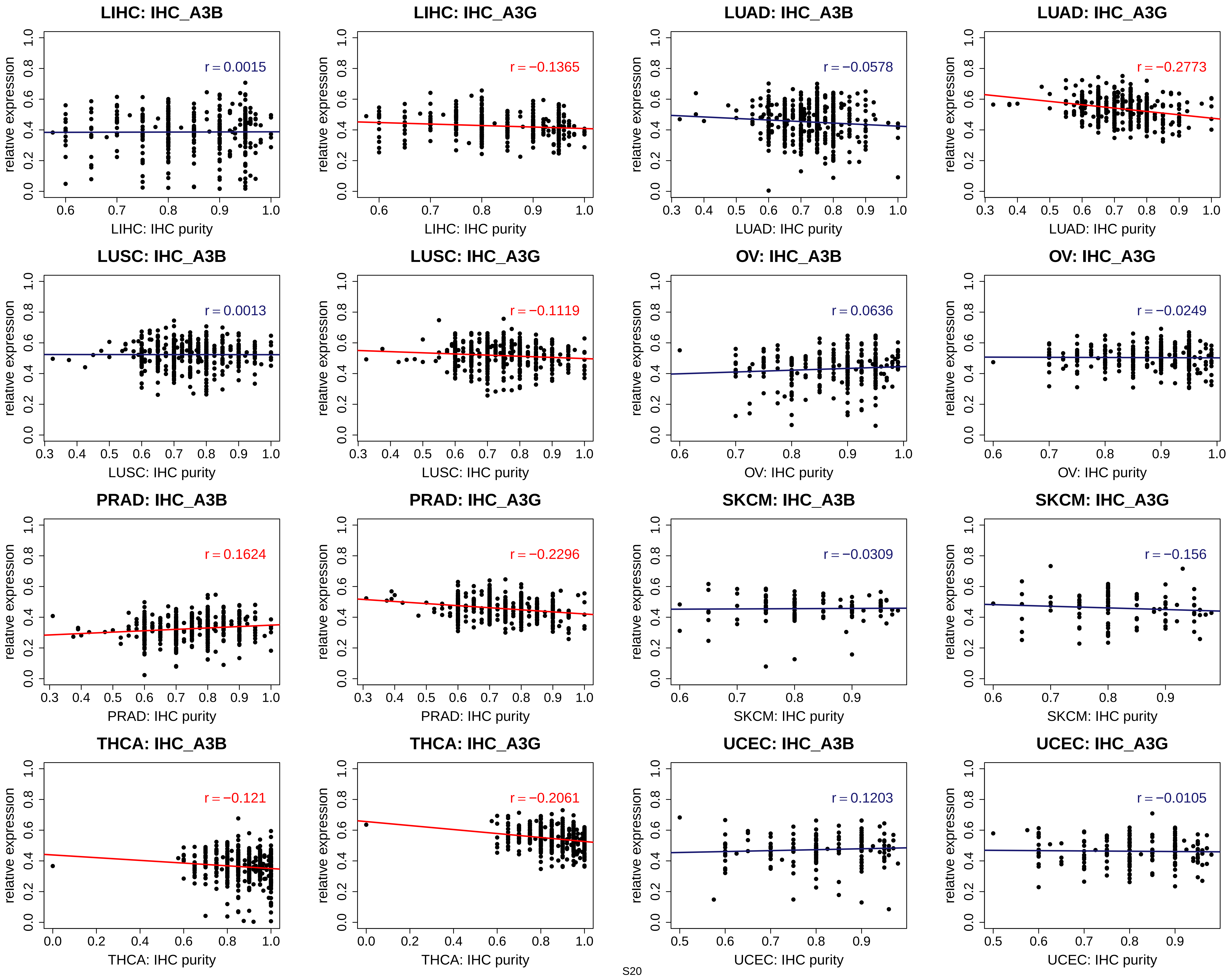

Supplementary Figure S2

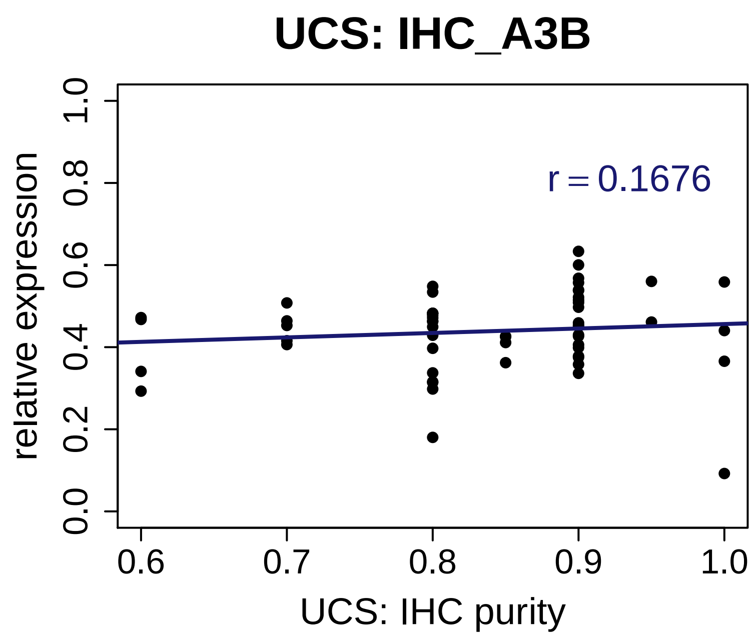

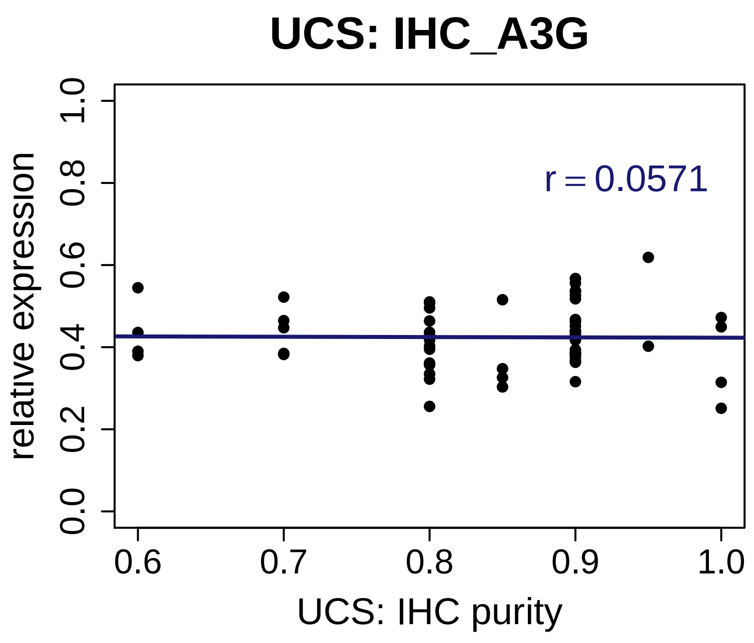

Supplementary Figure S2

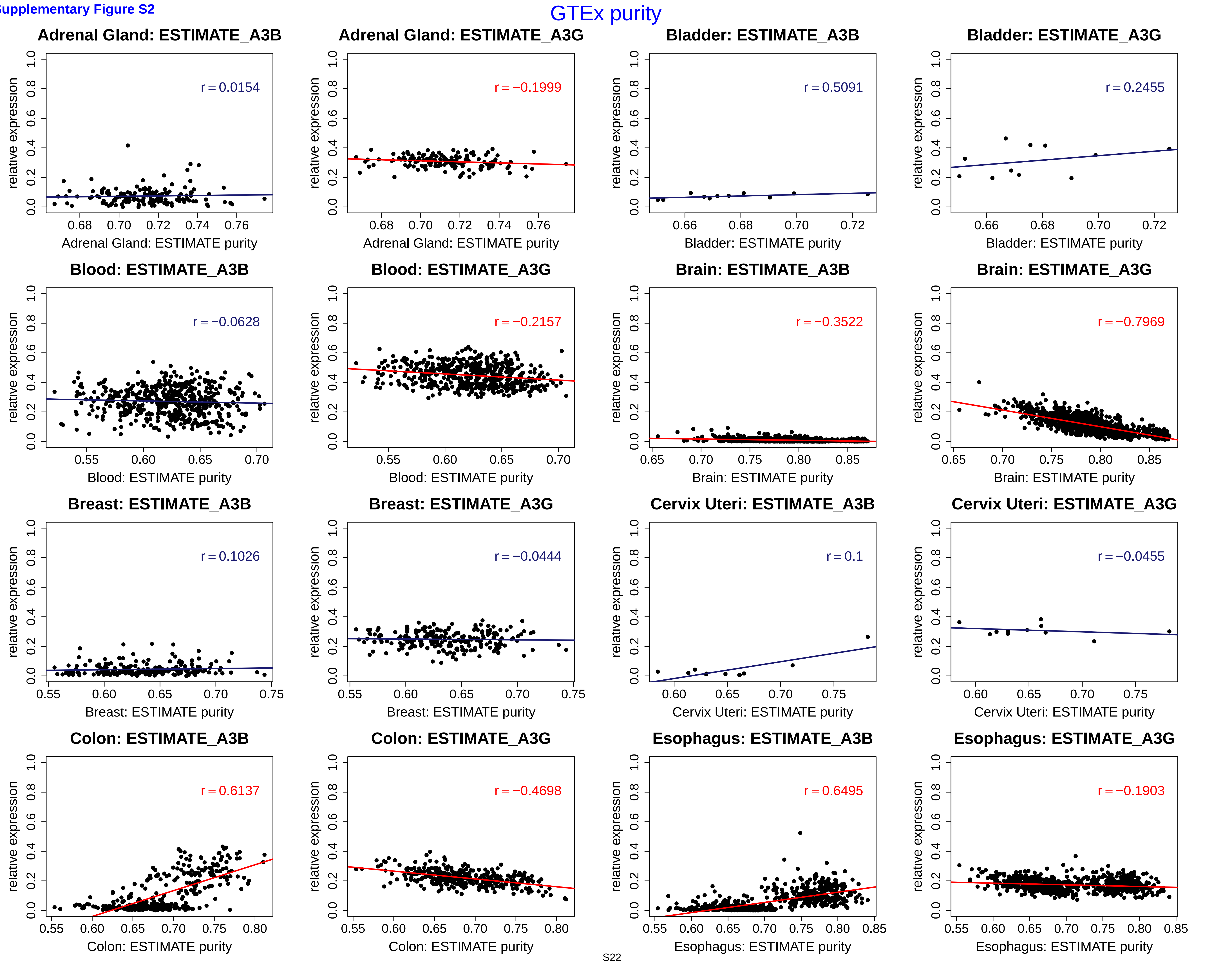

Supplementary Figure S2

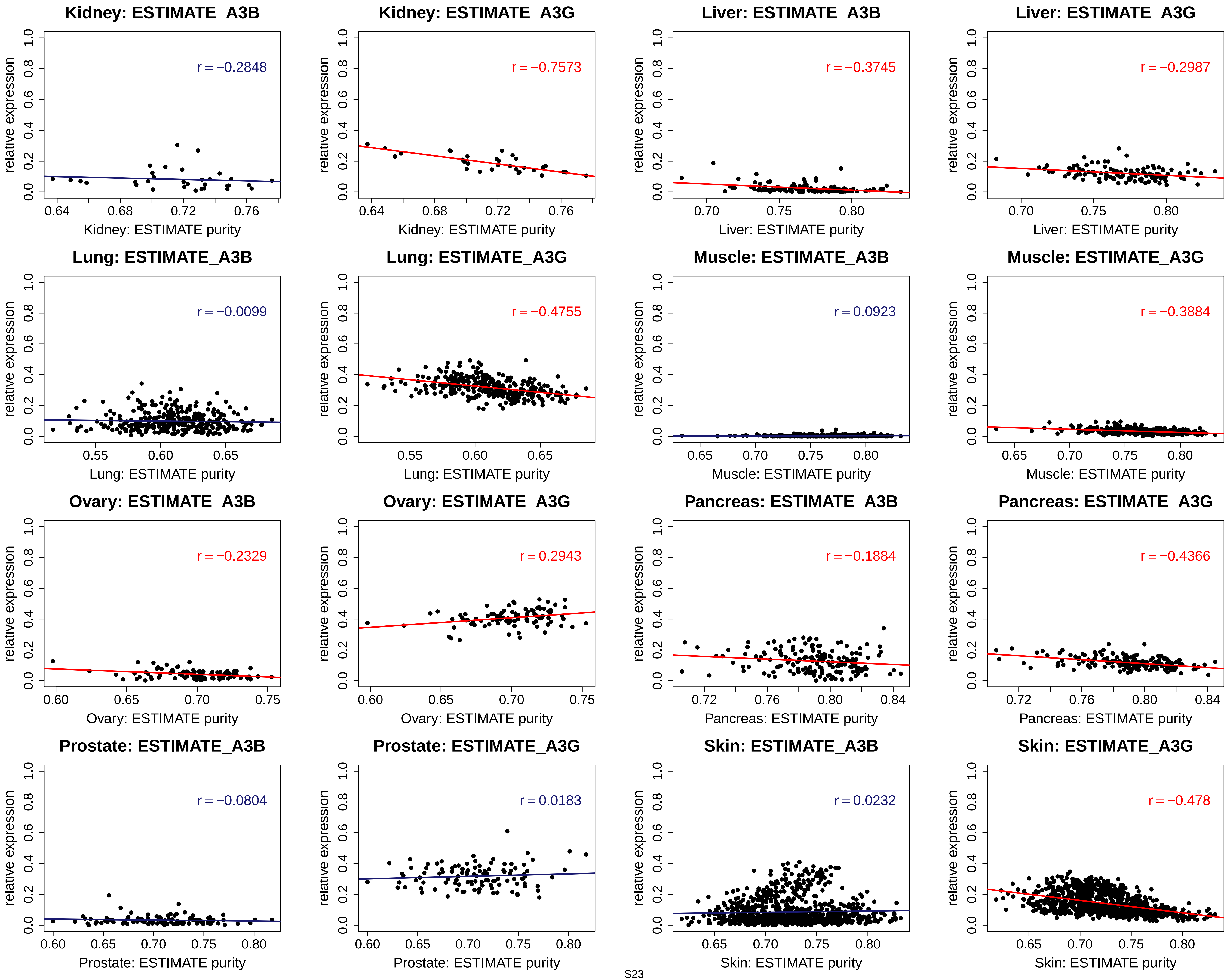

Supplementary Figure S2

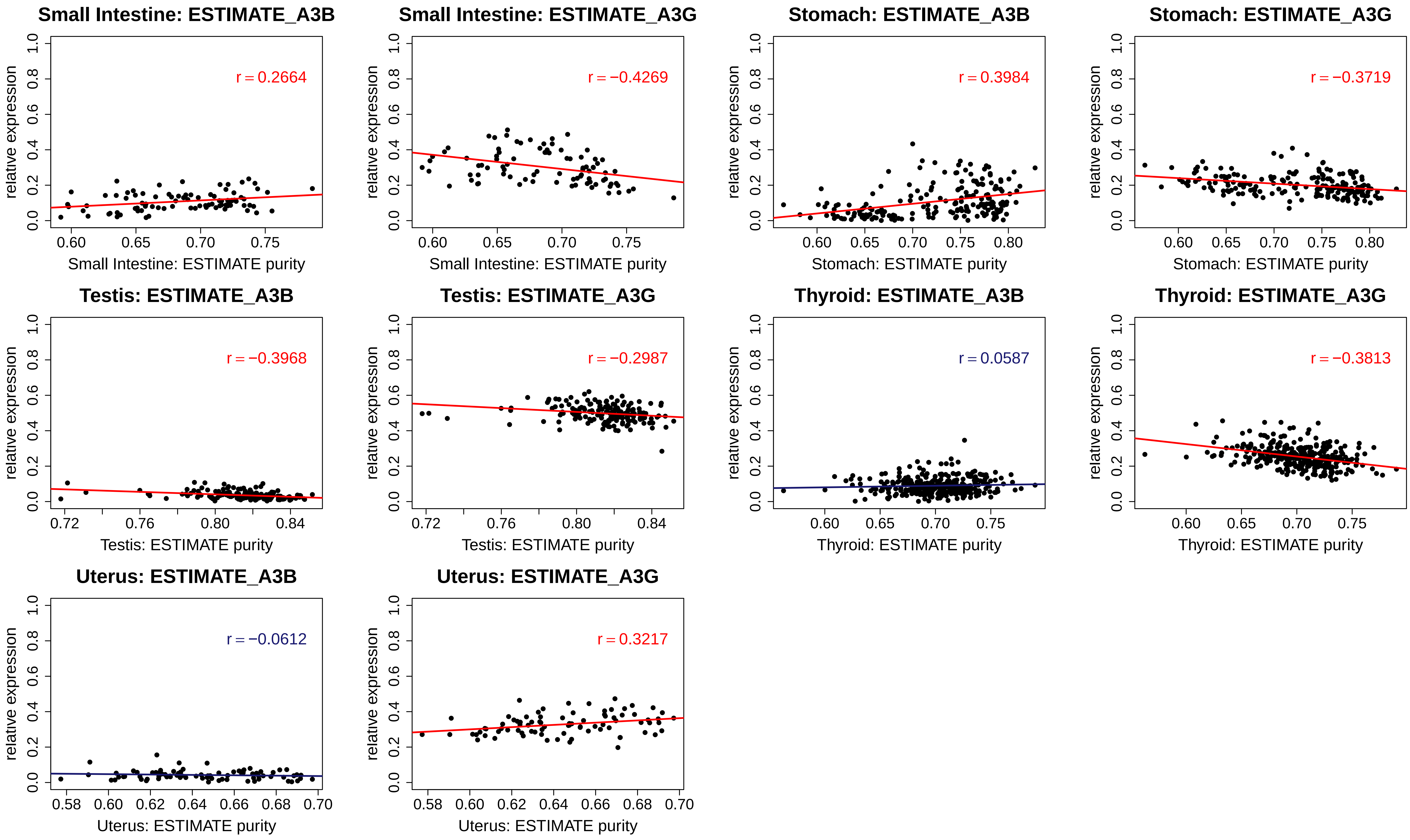

Supplementary Figure S2. Association between various tumour purity measurements/estimates and APOBEC3 gene expression.

The same plots as Figure 2B are shown here, but for all TCGA cancer types, and with purity data based on immunohistochemistry (IHC) and Composite Purity Estimate (CPE) provided by Aran, Sirota and Butte (2015). The analogous plots produced for GTEx tissue types are also included. Same colour code as in Figure 2B is adopted. See the corresponding figure caption for details.

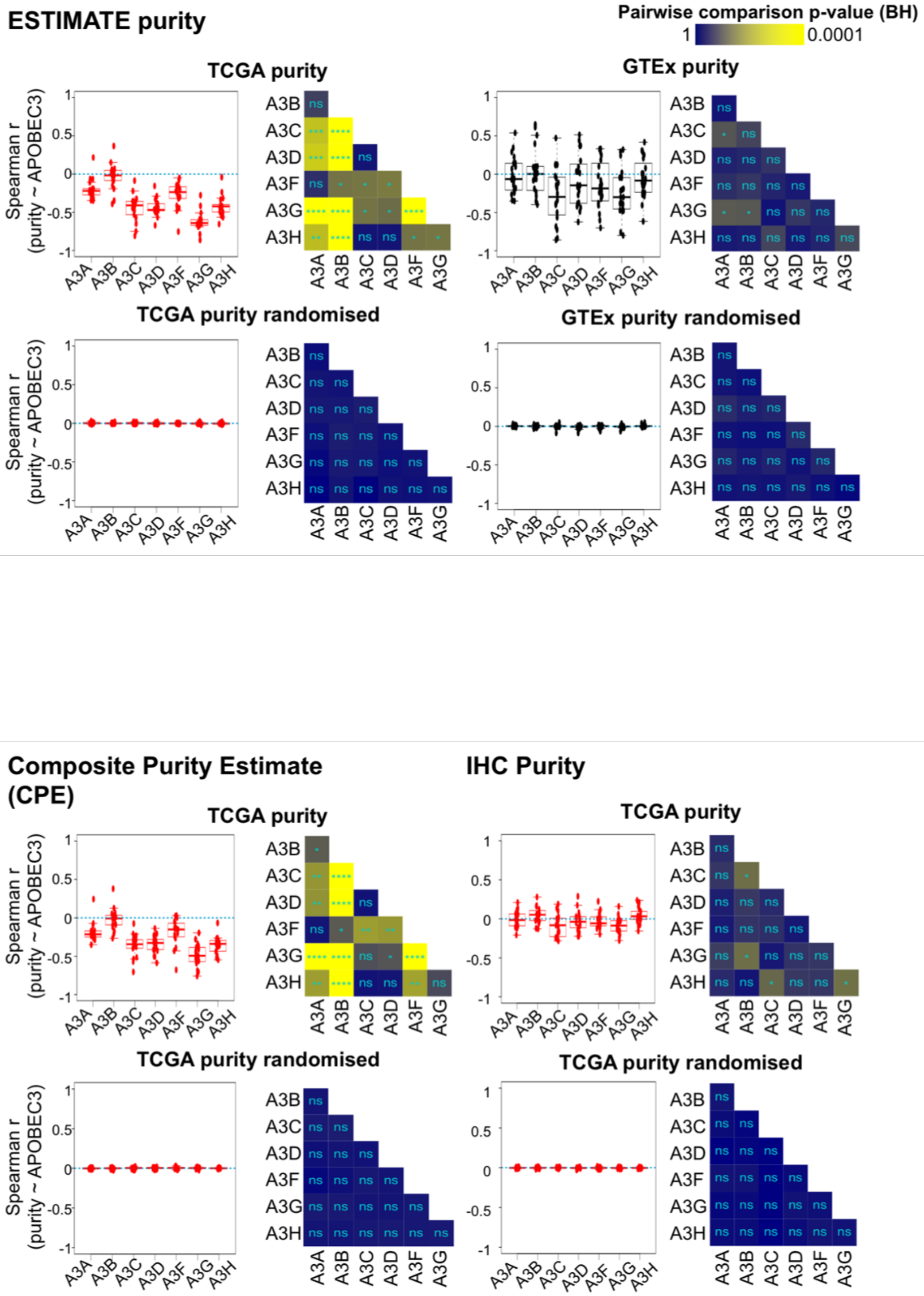

Supplementary Figure S3. Correlation boxplots of various tumour purity measurements/estimates with APOBEC3 gene expression.

Correlation analysis with GTEx estimated non-immune content (see Methods) are also shown here, together with randomised controls for all these statistics. Identical treatment as Figure 2C is adopted here. Figure 2C is taken from the TCGA ESTIMATE plot shown in this Figure.

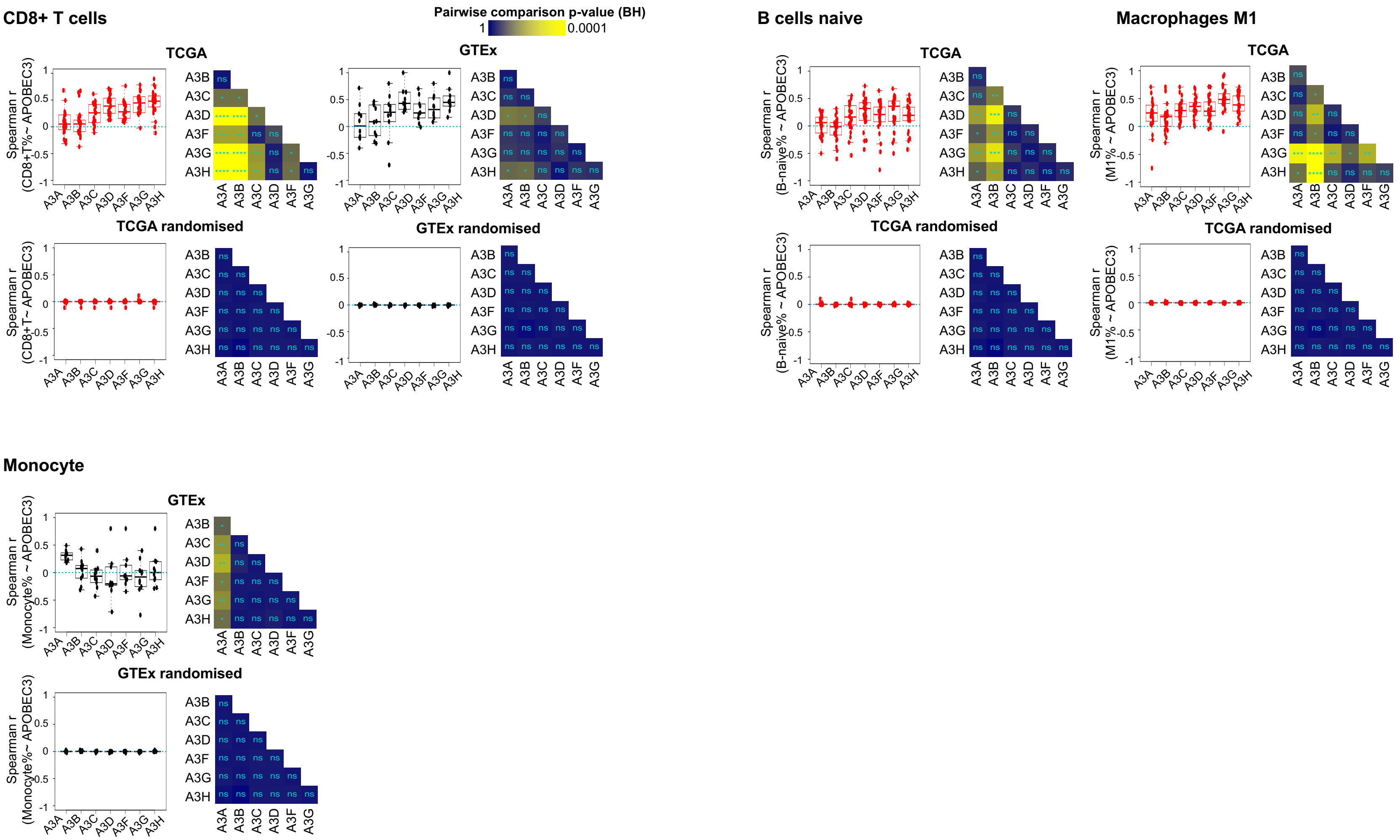

Supplementary Figure S4. Correlation boxplots of immune cell types proportions with APOBEC3 gene expression.

Only immune cell types with significant differences in correlation values for each APOBEC3 gene are shown here. Randomised controls for these statistics are included as well. Identical treatment as Figure 3C is adopted here. Figure 3C is taken from the TCGA and GTEx CD8+ T cell plots shown in this Figure.

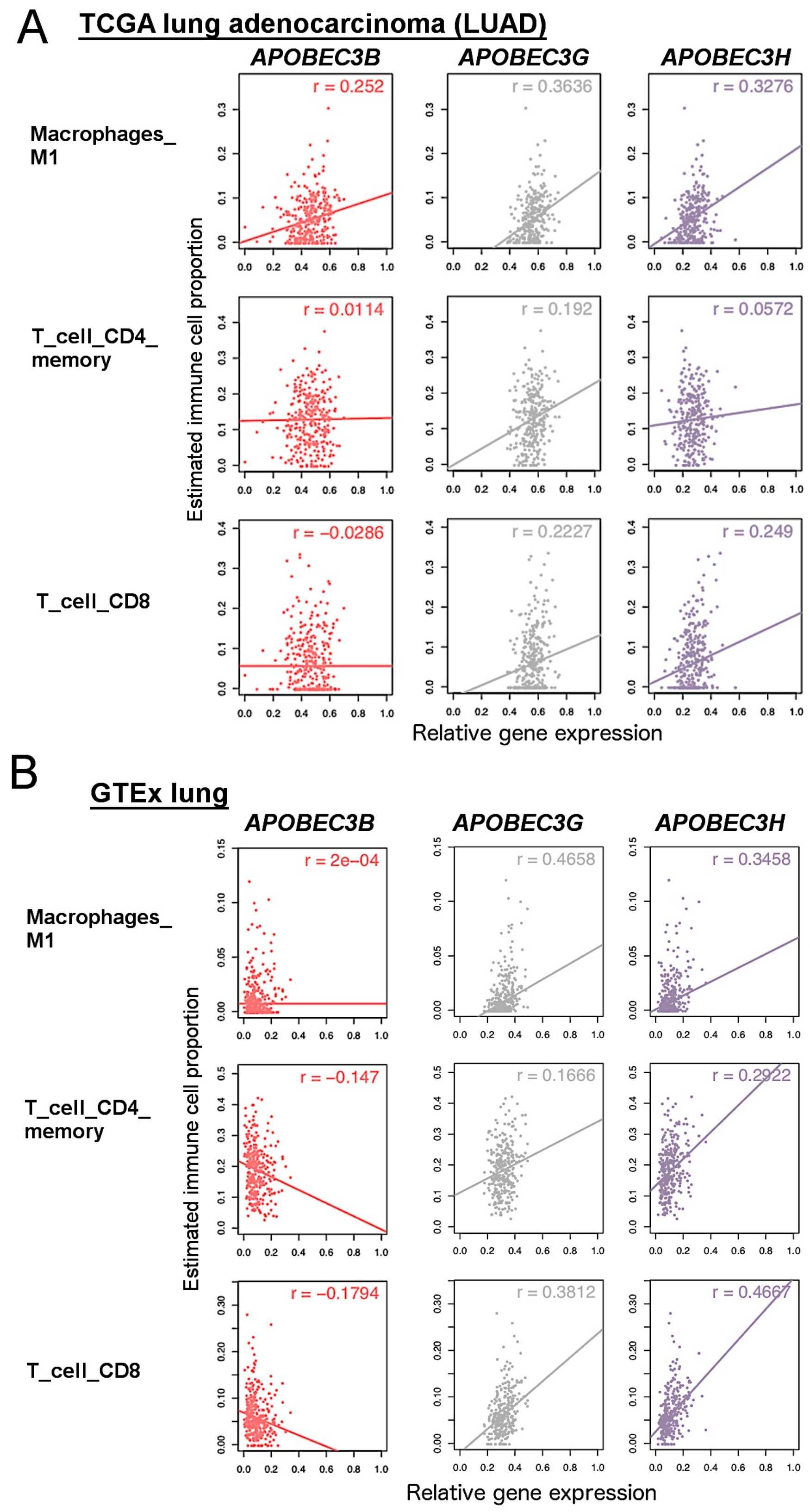

Supplementary Figure S5. Association of the estimated T cells and Macrophage M1 levels and APOBEC3 gene expression.

Here the expression of *APOBEC3B*, *APOBEC3G* and *APOBEC3H* (normalised against *GAPDH* expression) are considered, and data of TCGA lung adenocarcinoma (A) and GTEx lung samples (B) are shown. Spearman correlation coefficients are given.

A **TCGA Immune**

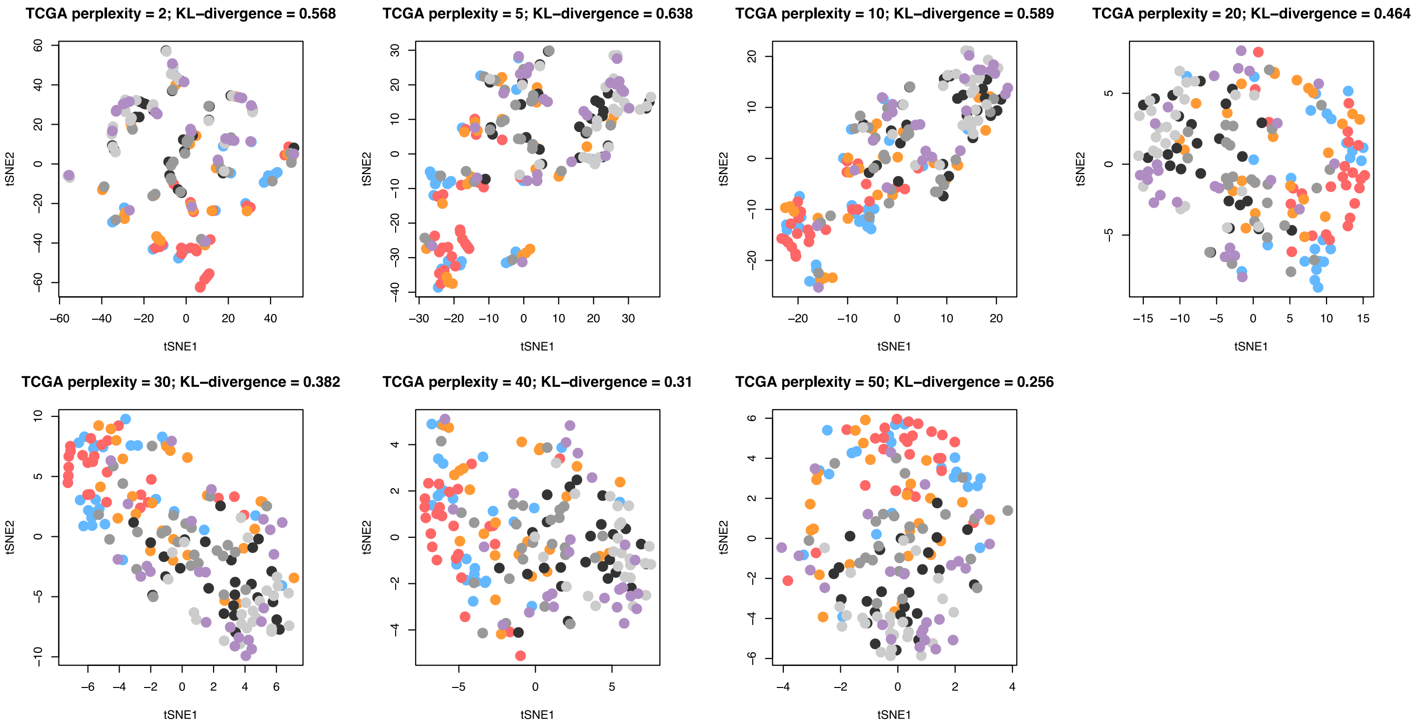

B **GTEx Immune**

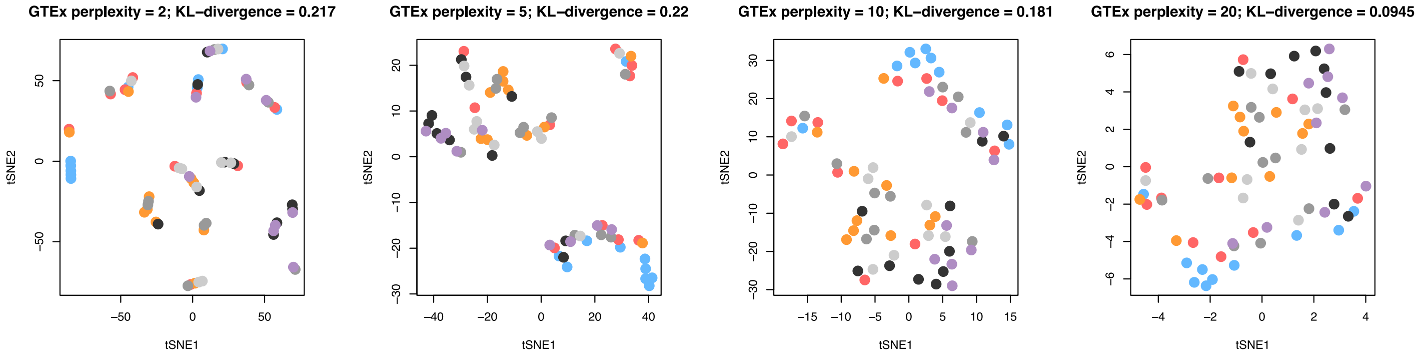

Supplementary Figure S6. t-Distributed Stochastic Neighbour Embedding (t-SNE) analysis of the immune cell type-APOBEC3 expression correlation data.

t-SNE was performed over a range of perplexity parameter values (see Methods) and iterated to find the optimal solution (lowest Kullback-Leibler divergence). Plots were shown for the optimal solution in the TCGA Immune component analysis (A) and the GTEx immune component analysis (B). Results were shown from the lowest perplexity value to the largest value in the list (see Methods) with which the algorithm is still able to find a solution. These values were shown above each plot. Colour codes and descriptions follow that of Figure 3D.

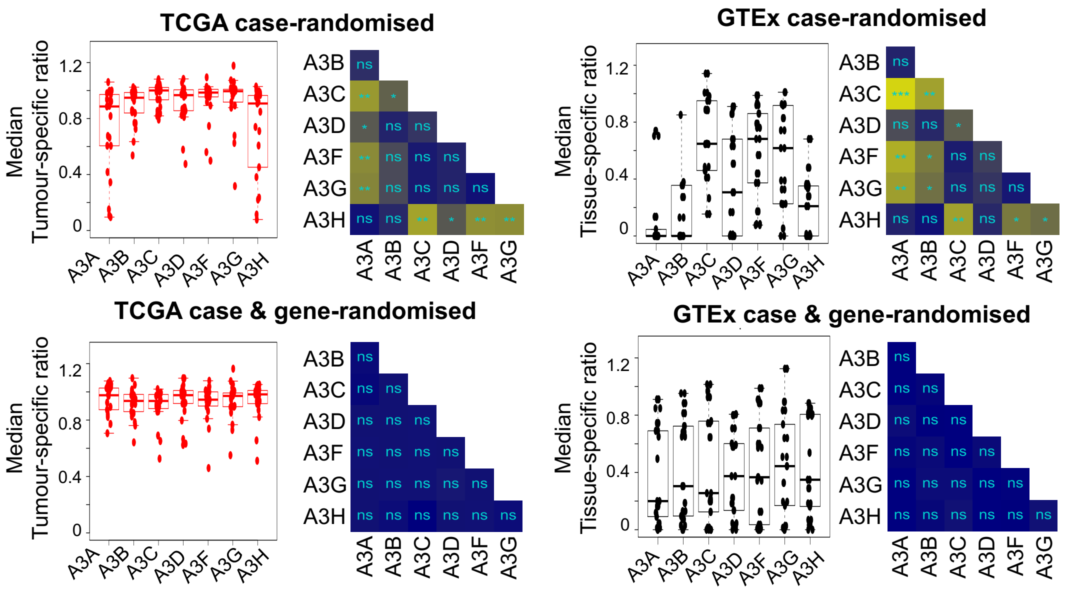

Supplementary Figure S7. Correlation boxplots of tumour/nonimmune specific ratio for TCGA and GTEx cohorts.

Randomised case and gene controls are also shown here (see Methods). Each data point corresponds to one cancer/tissue type. Identical treatment as Figure 4B is adopted here. Notice that significant differences still exist in the case-randomised controls. This could be due to very similar expression levels across cases for particular APOBEC3 gene(s) that leads to little differences when case labels are randomised. The case-and-gene randomised controls, in which gene labels are also randomised, support this hypothesis.

Supplementary Figure S8

Lung adenocarcinoma dataset (GSE69405)

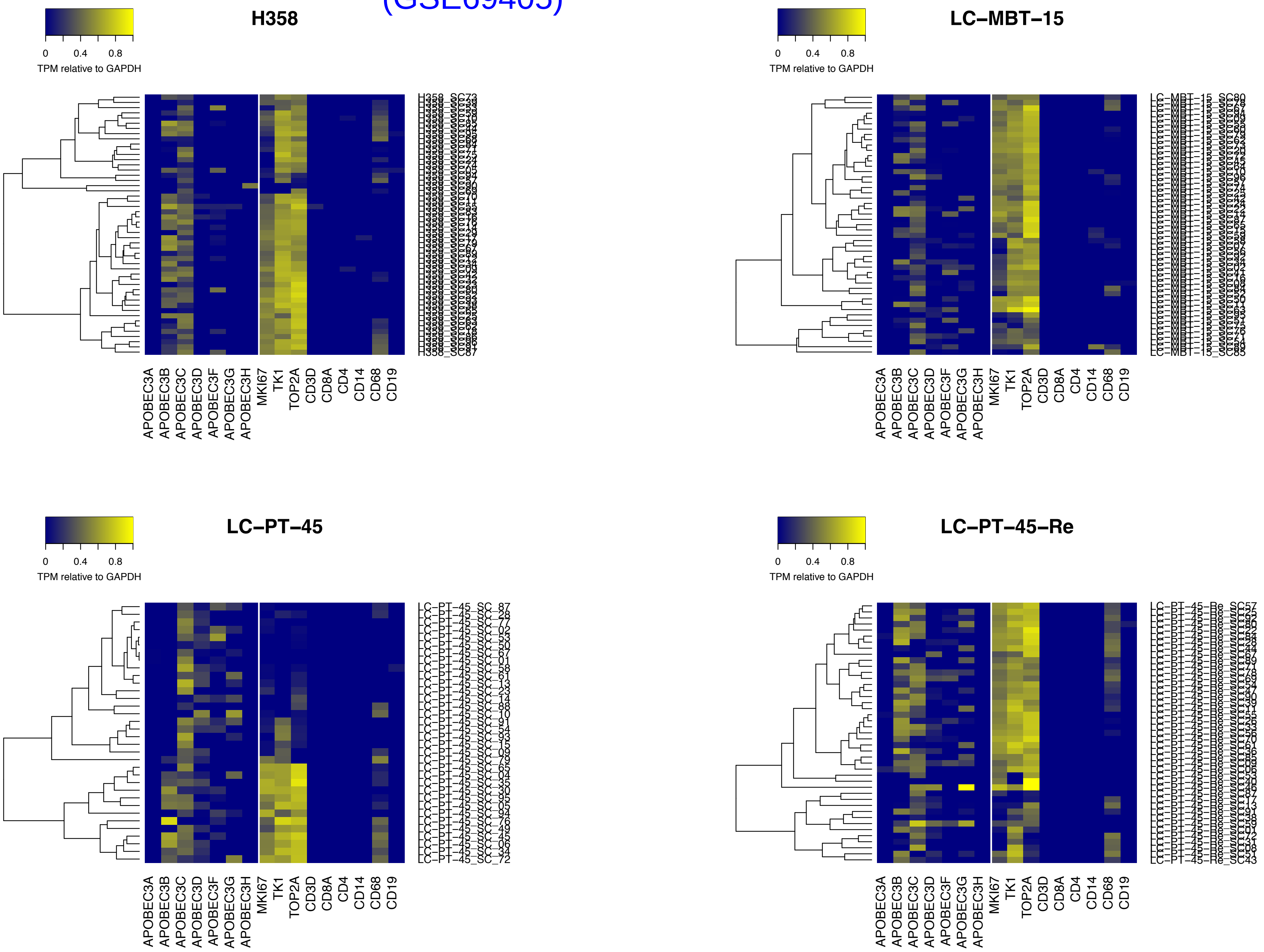

Supplementary Figure S8

Breast tumour dataset (GSE75688)

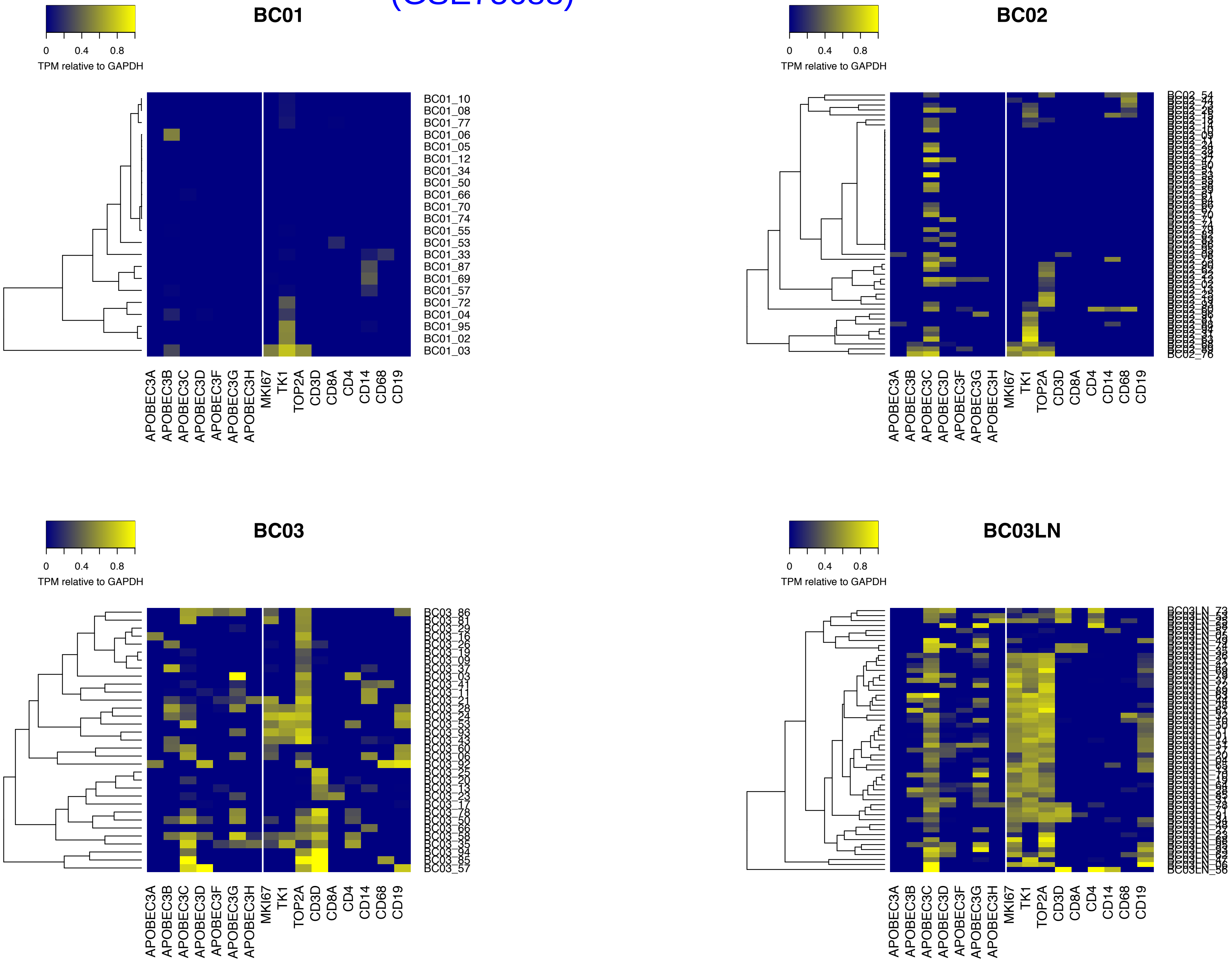

Supplementary Figure S8

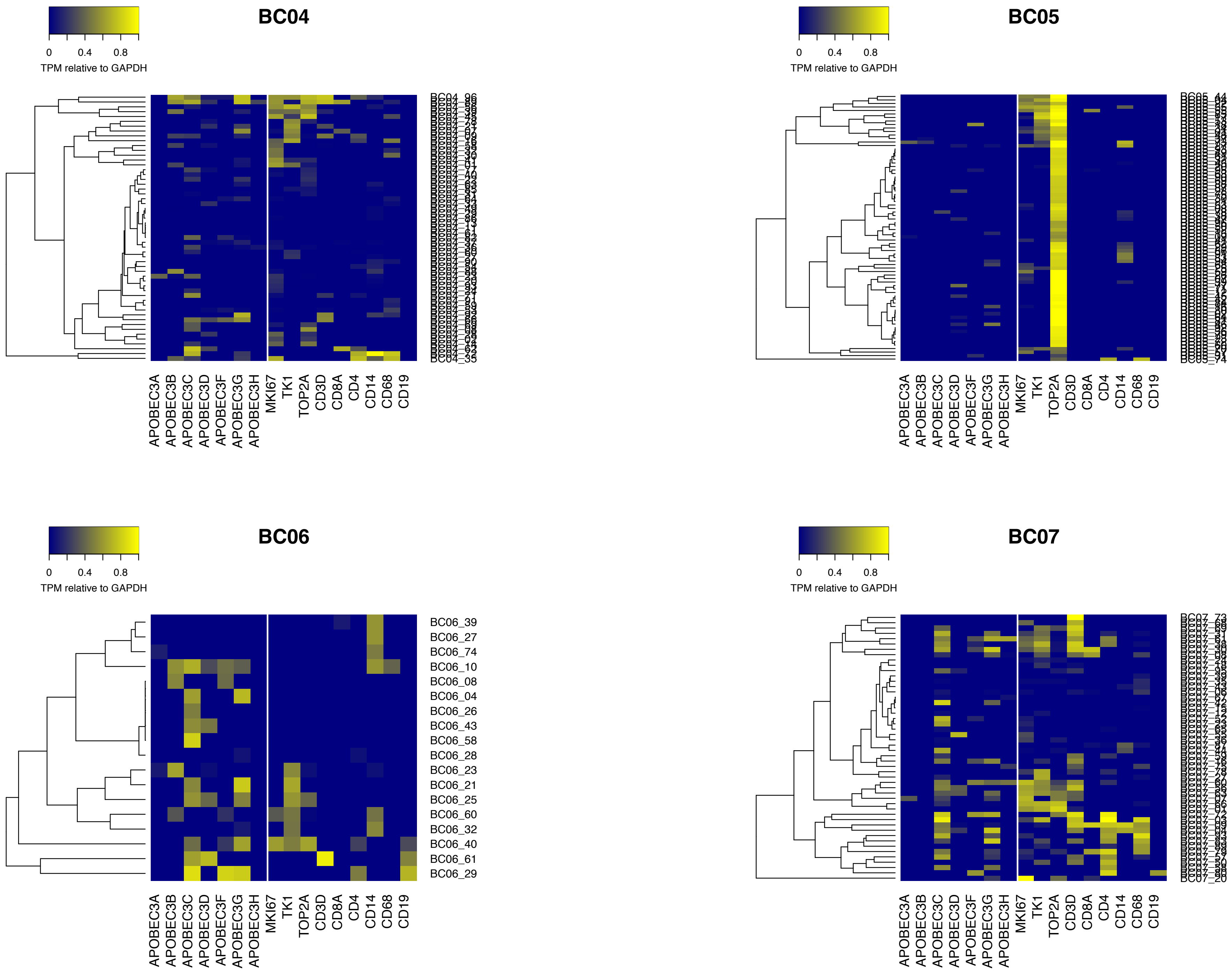

Supplementary Figure S8

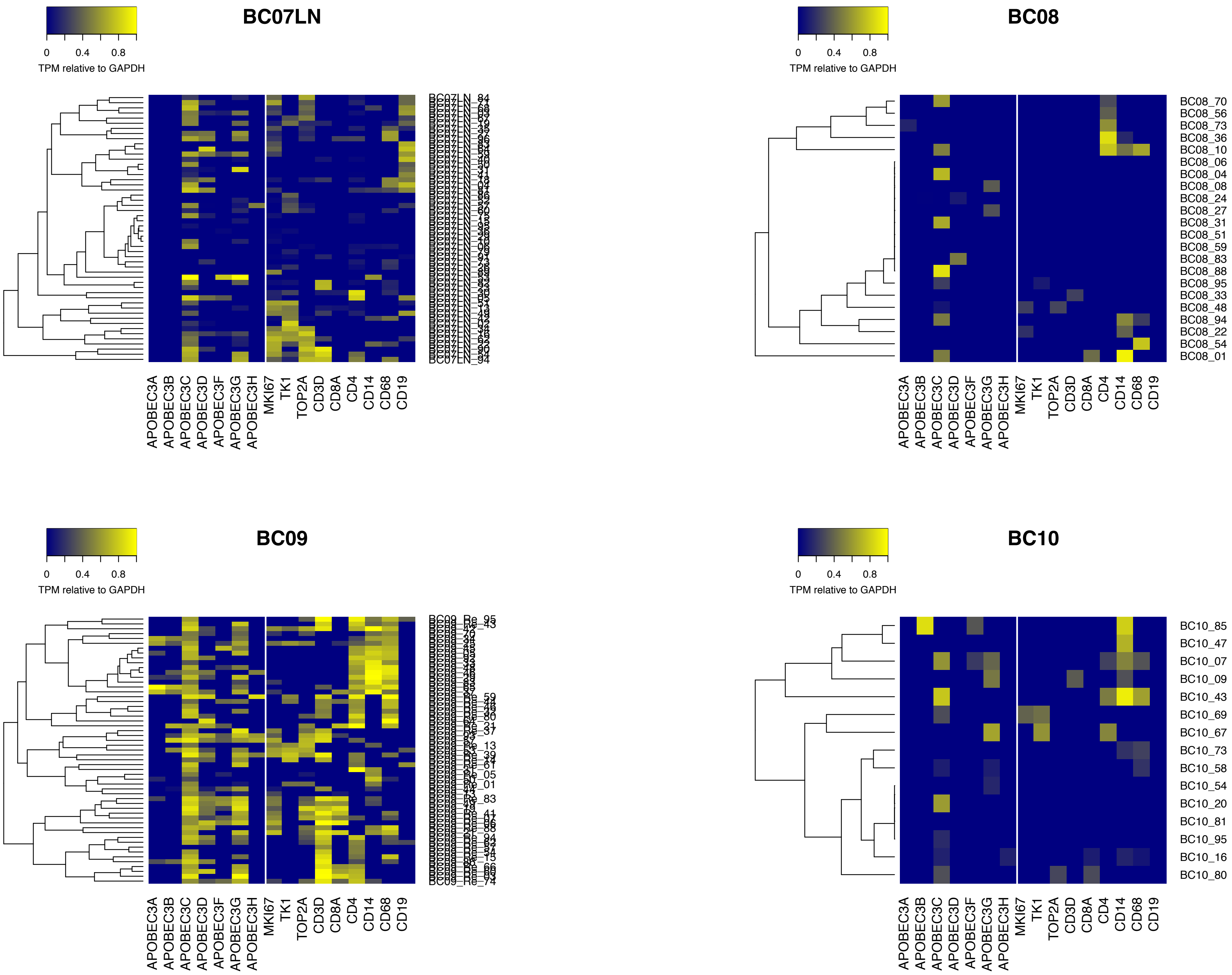

Supplementary Figure S8

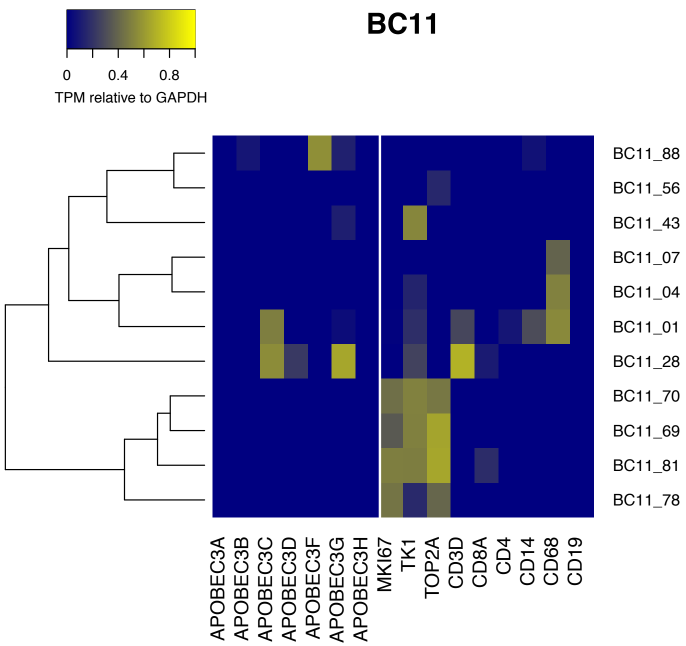

Supplementary Figure S8. Case-by-case expression heatmap for single-cell RNA sequencing data analysed in this study.

Colour codes and description follow that of Figure 4C.

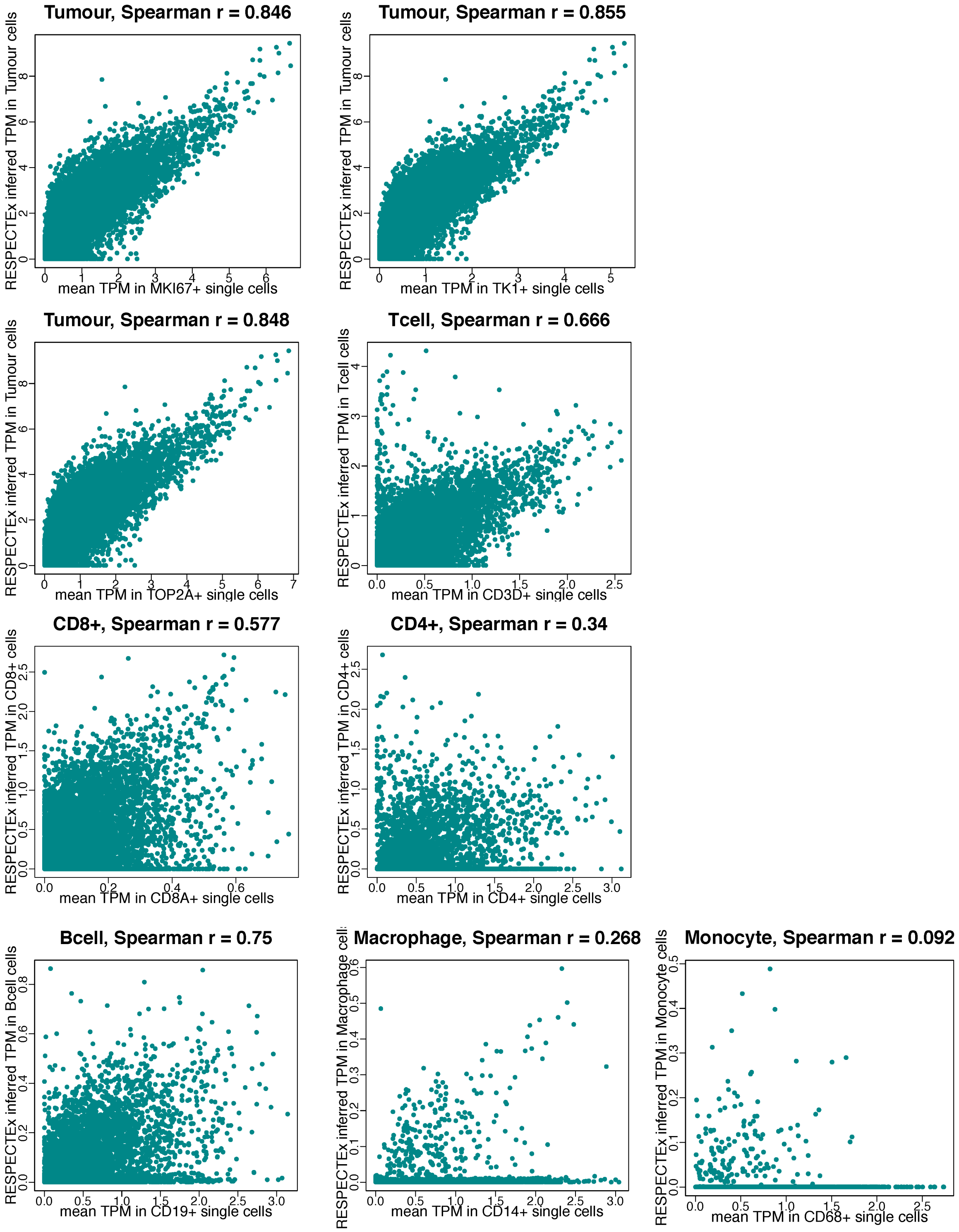

Supplementary Figure S9. Correlation between RESPECTEx-deconvolved cell-type specific expression with expression in marker gene-positive single cells.

Description follows that of Figure 4D. Here all marker genes displayed in Figure 4C were considered with their corresponding cell types. The figures (Figure 4D) for Tumours (*MKI67+*) and CD8+ T cells (*CD8A+*) were taken from here.

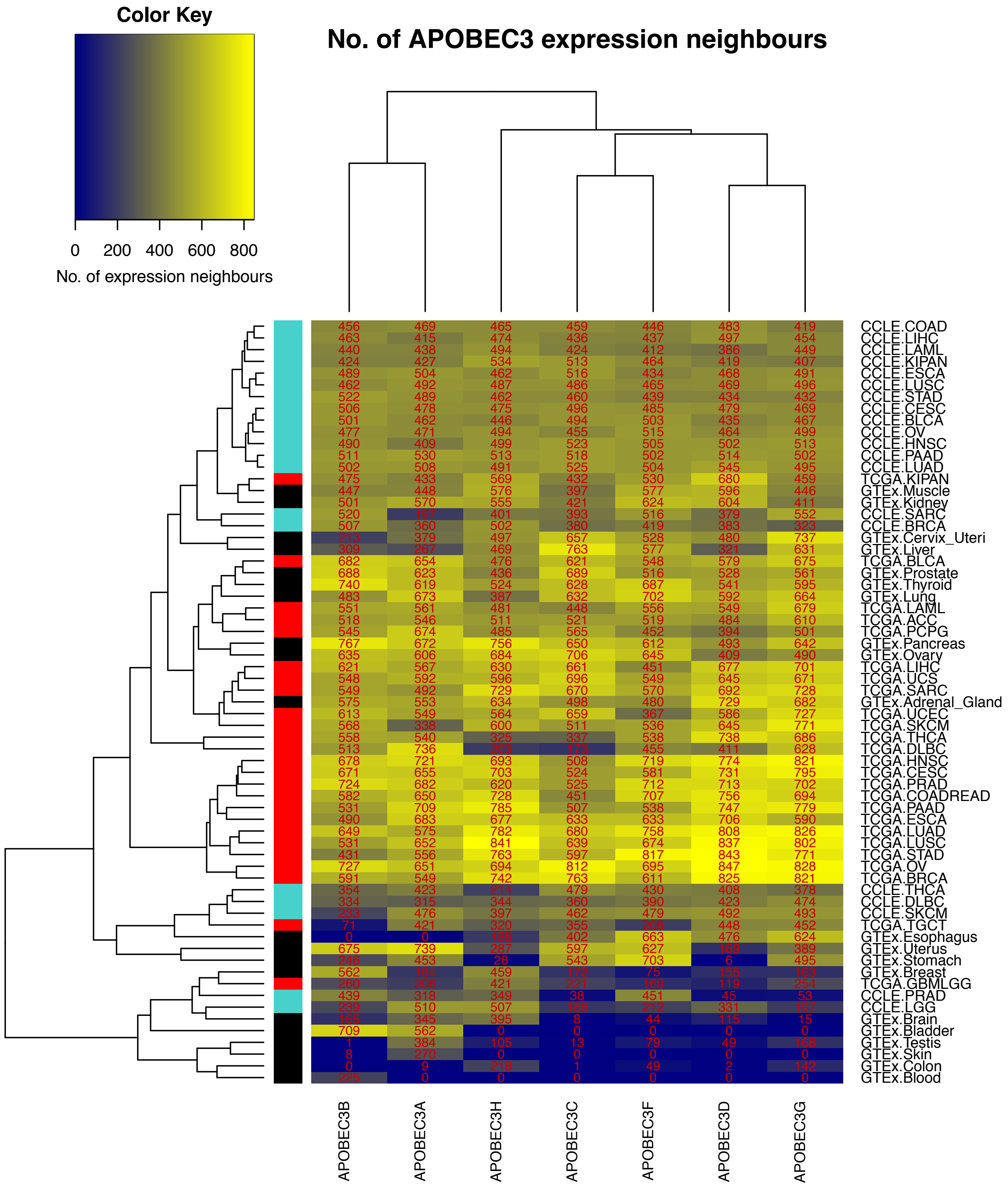

Supplementary Figure S10. Statistics of co-expressing genes extracted.

A heatmap of the numbers of co-expressing genes extracted for each APOBEC3 gene in each cohort examined. The exact numbers are indicated on the heatmap, and are identical to that included in Supplementary Table S11.

A Gene ontology (GO) Biological processes

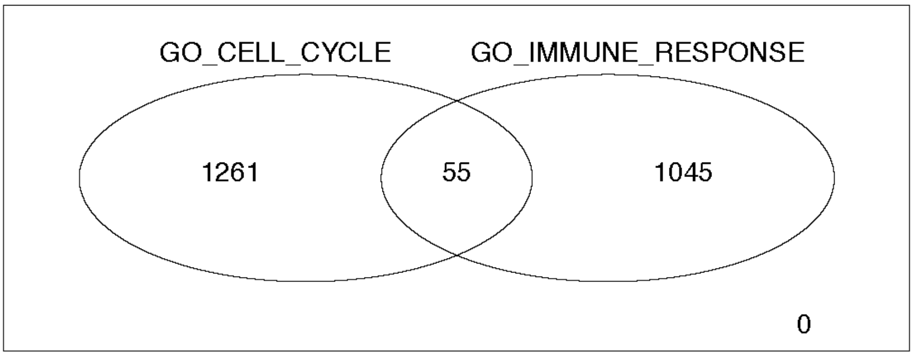

B Curated gene sets

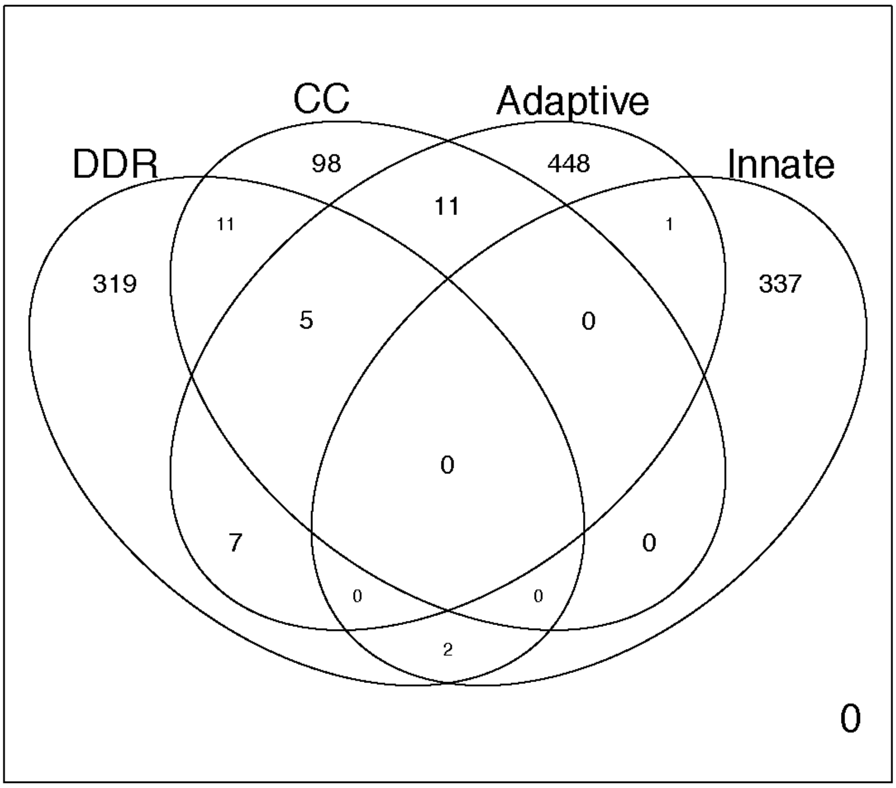

Supplementary Figure S11. Overlap of gene sets.

Venn diagrams depict the overlap of gene sets utilised in this study: the GO cell cycle and immune response gene sets (A); the four categories of curated gene sets (B) (see Result and Methods sections).

Supplementary Figure S12. Functional barcodes of APOBEC3 co-expressing genes.

The functional barcodes for the two GO gene sets (cell cycle/immune process) and the four categories of curated gene sets are shown. Data include all considered tumour, cancer cell lines and normal cohorts in this study.
Uploaded separately.

Supplementary Figure S13. APOBEC3 gene co-expression networks of tumours, cancer cell lines and normal tissues.

See Figure 5 for description of the Circos plot.
Uploaded separately.

Supplementary Figure S14. APOBEC3 gene co-expression networks extracted from the median of bootstrapped samples.

Bootstrapped samples were taken (see Methods) for the correlation analysis, and the median of these samples were subjected to co-expression partner extraction and annotation. Identical colour codes, layout and explanations as detailed in Figure 5.
Uploaded separately.

Supplementary Figure S15. Gene Set Enrichment Analysis (GSEA) of co-expressing genes of APOBEC3 genes.

Principal Component Analysis (PCA) plots for all considered cohorts in this study. The data correspond to those in Supplementary Figure S13. Colour scheme corresponds to that in Figure 6C.
Uploaded separately.

Supplementary Figure S16. GSEA of co-expressing genes of APOBEC3 genes extracted from the median of bootstrapped samples.

These plots are analogous to Supplementary Figure S15, but the data correspond to the Circos plot shown in Supplementary Figure S14.
Uploaded separately.

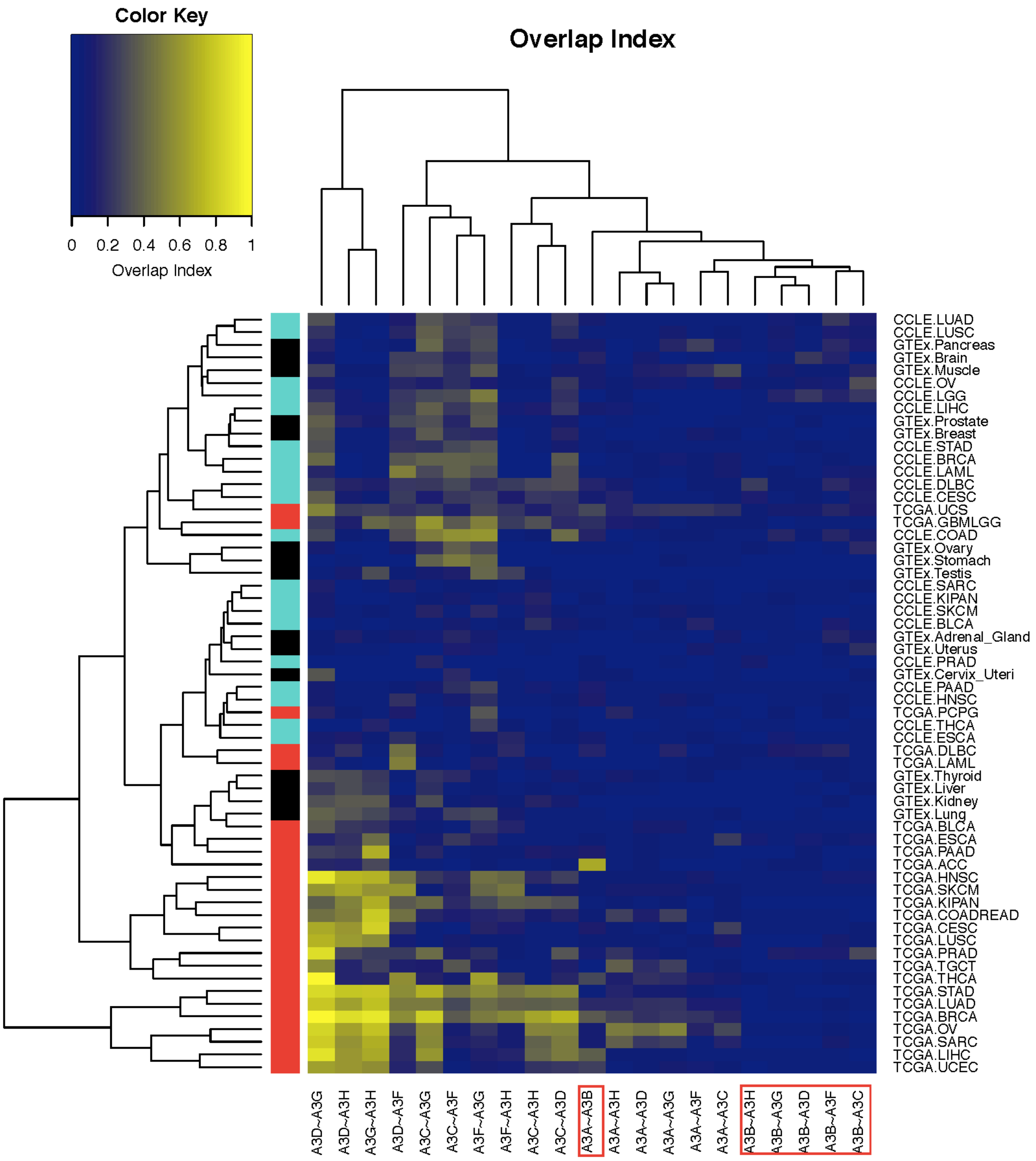

Supplementary Figure S17. Quantifying the overlap of co-expressing genes shared between pairs of APOBEC3 genes.

The extent of overlap of gene co-expression between all pairs of APOBEC3 genes was quantified with the Jaccard score. An overlap index was derived by normalising the Jaccard scores to be in the range of [0, 1] by dividing against the maximum value in the matrix. Such quantification is shown as a heatmap here. Pairs involving APOBEC3B are indicated with a red rectangle around column labels (see bottom of plot).

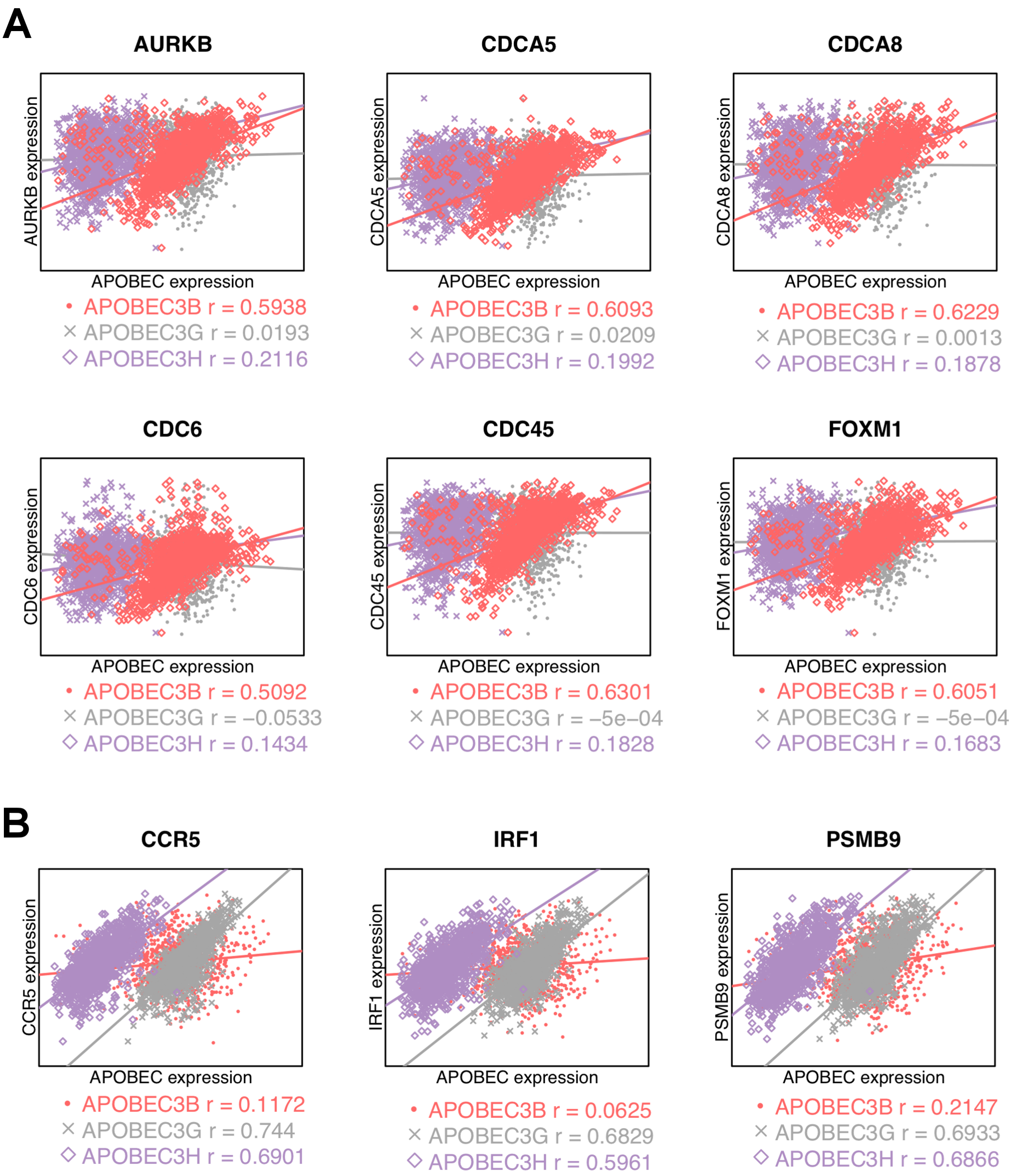

Supplementary Figure S18. Relationship between expression of selected genes with APOBEC3 gene expression.

Here *APOBEC3B*, *APOBEC3G* and *APOBEC3H* expression in TCGA Breast invasive carcinoma (BRCA) are considered. Respective Spearman correlation coefficient are shown under each plot. Data points are colour coded per APOBEC3 gene involved. Two categories of marker genes were considered: genes known to involve in cell cycle transition and control (A), and immune marker genes (B). Notice opposite trends to panel (A) depicted in these plots.

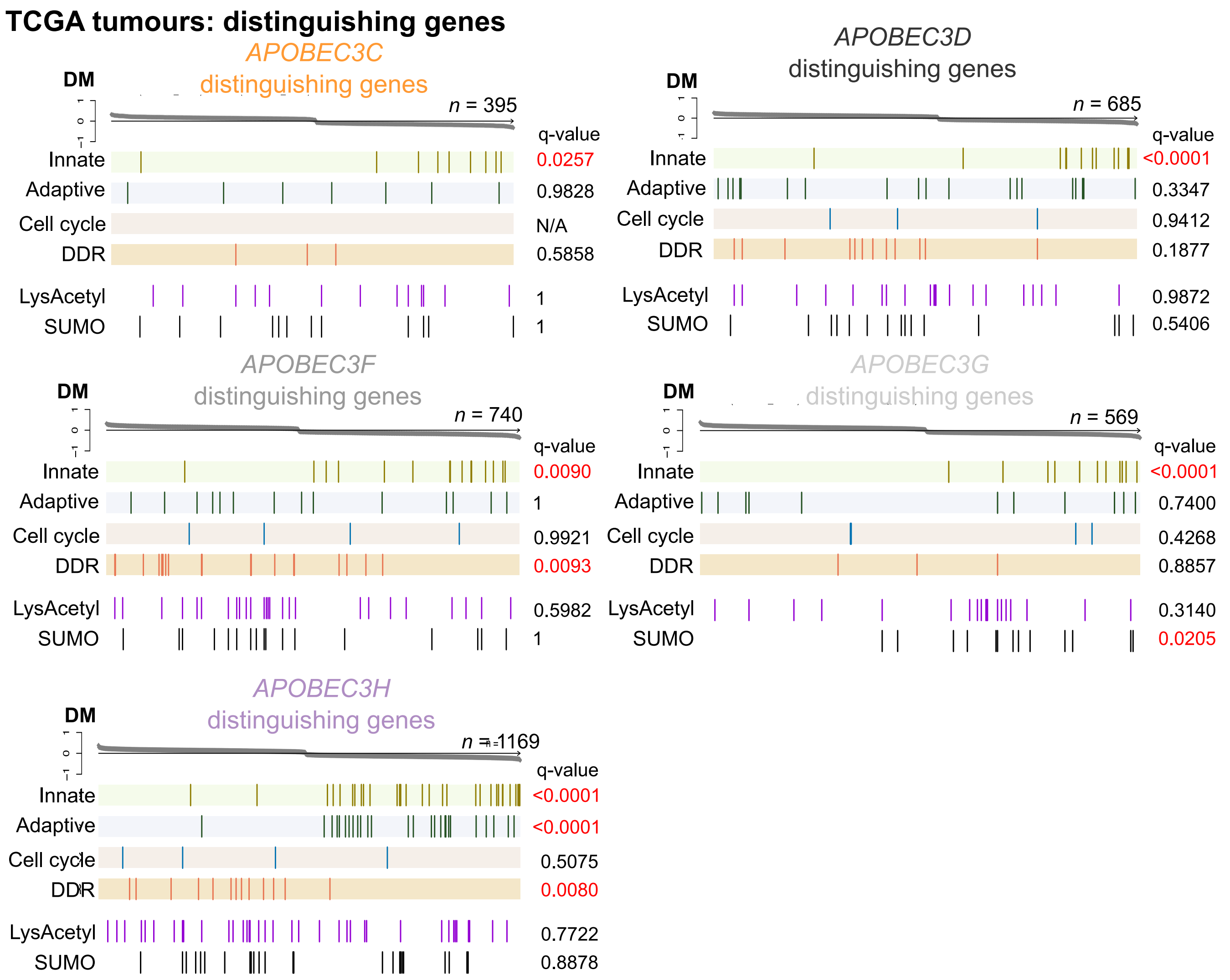

Supplementary Figure S19. Distinguishing genes barcodes for APOBEC3 genes in the TCGA cohorts.
APOBEC3A and APOBEC3B distinguishing genes were included in Figure 7B. See Figure 7B and its caption for details.

GTEx normal tissues: distinguishing genes

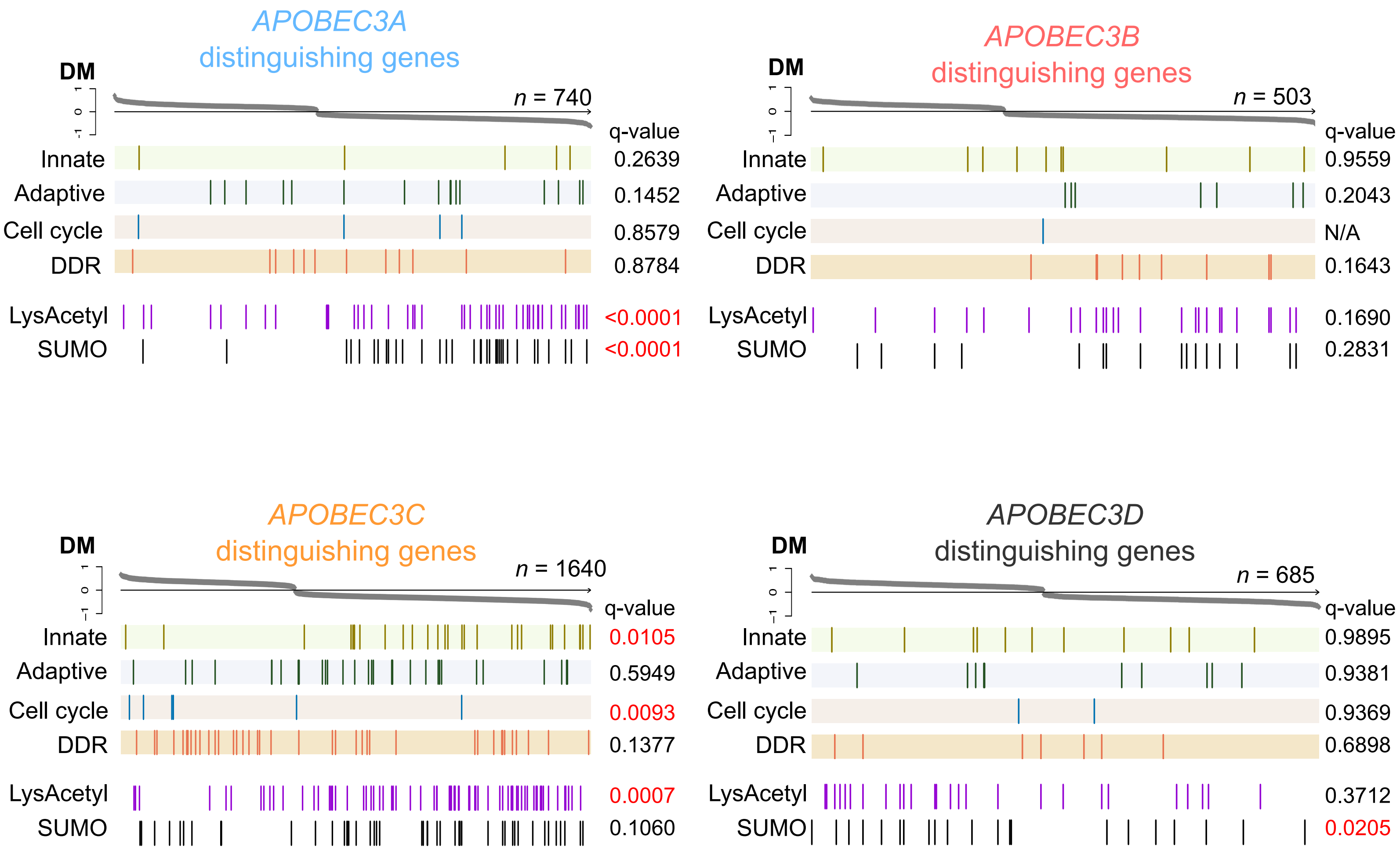

GTEx normal tissues: distinguishing genes

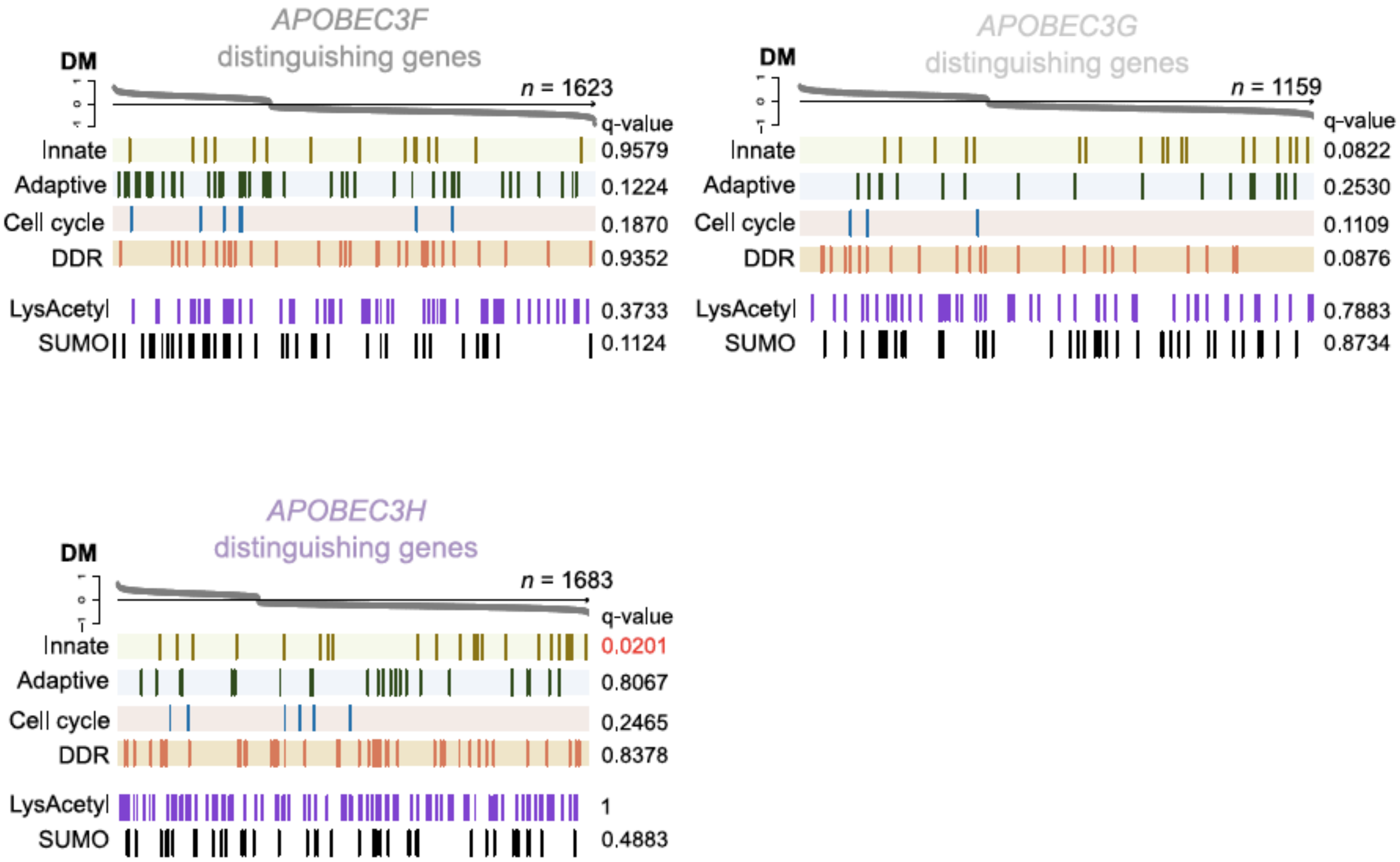

Supplementary Figure S20. Distinguishing genes barcodes for APOBEC3 genes in the GTEx cohorts.
See Figure 7B and its caption for details.

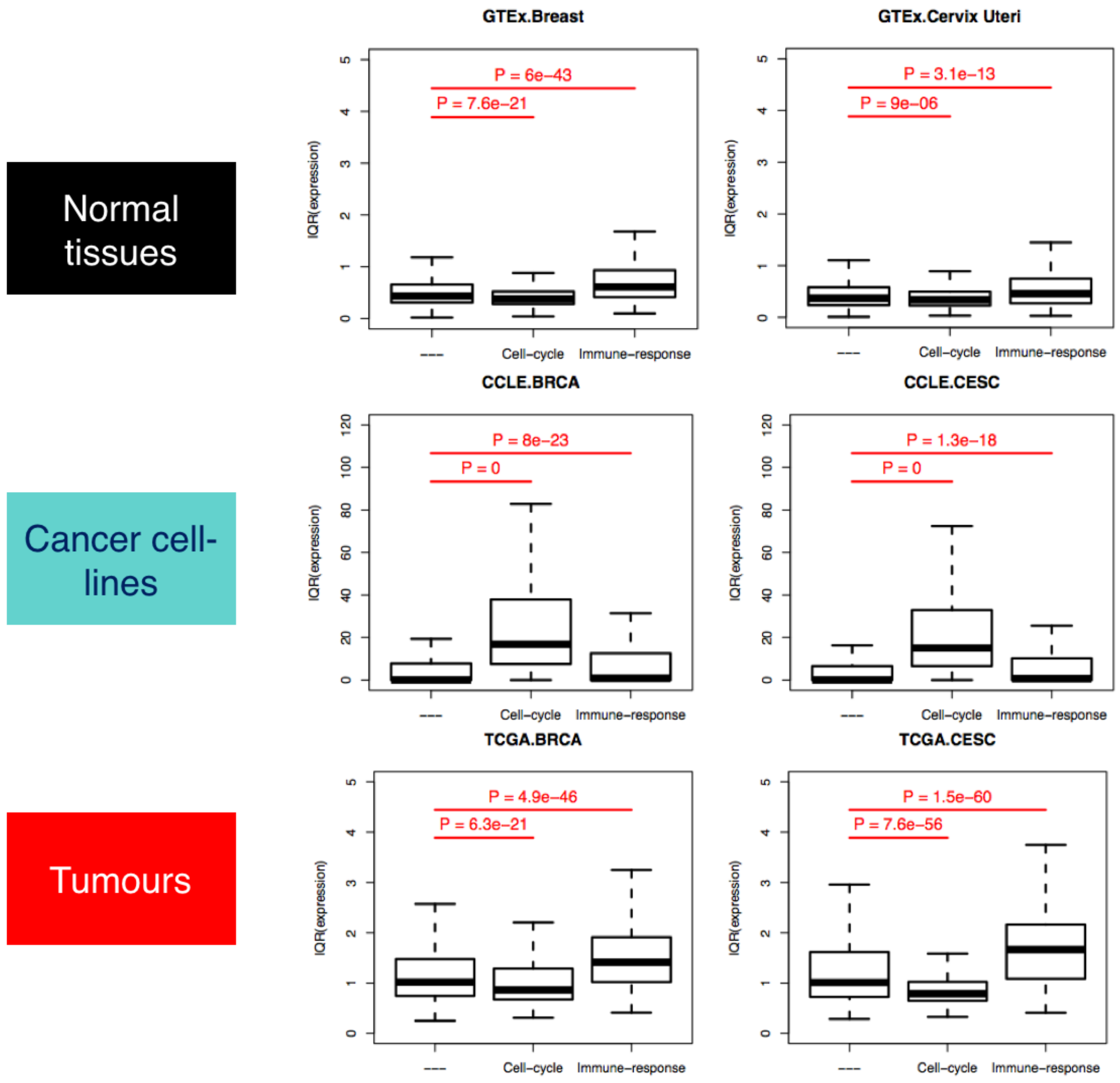

Supplementary Figure S21. Fluctuations in transcriptome and its relation to functional groups of genes.

Genes in the RNA-seq data in tumours, normal tissues and cancer cell-lines were divided into three groups: cell-cycle genes (those in the GO_CELL_CYCLE gene set), immune-response genes (those in the GO_IMMUNE_RESPONSE gene set), and those that overlap with neither sets. Inter-quartile ranges from each cohort for each gene are plotted here as a distribution across the three groups of genes defined. Note the large range in expression signals for the cell-cycle genes in cancer cell-lines and the immune-response genes in tumours, both reflective of the composition and nature of the samples. P-values were obtained by pairwise Wilcoxon tests.

Supplementary Figure S22

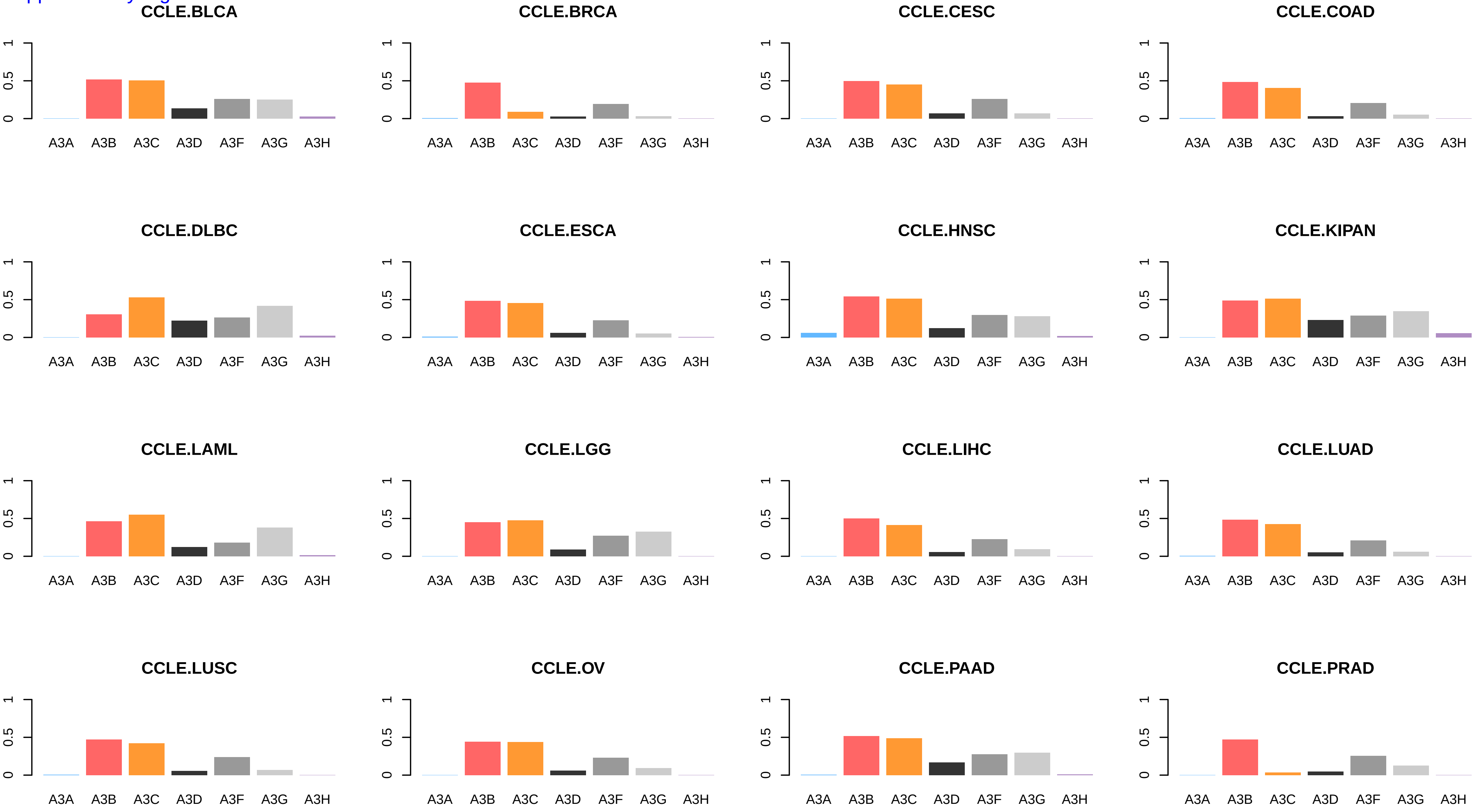

y-axis: median expression value (log2, relative to GAPDH)

Supplementary Figure S22 (cont.)

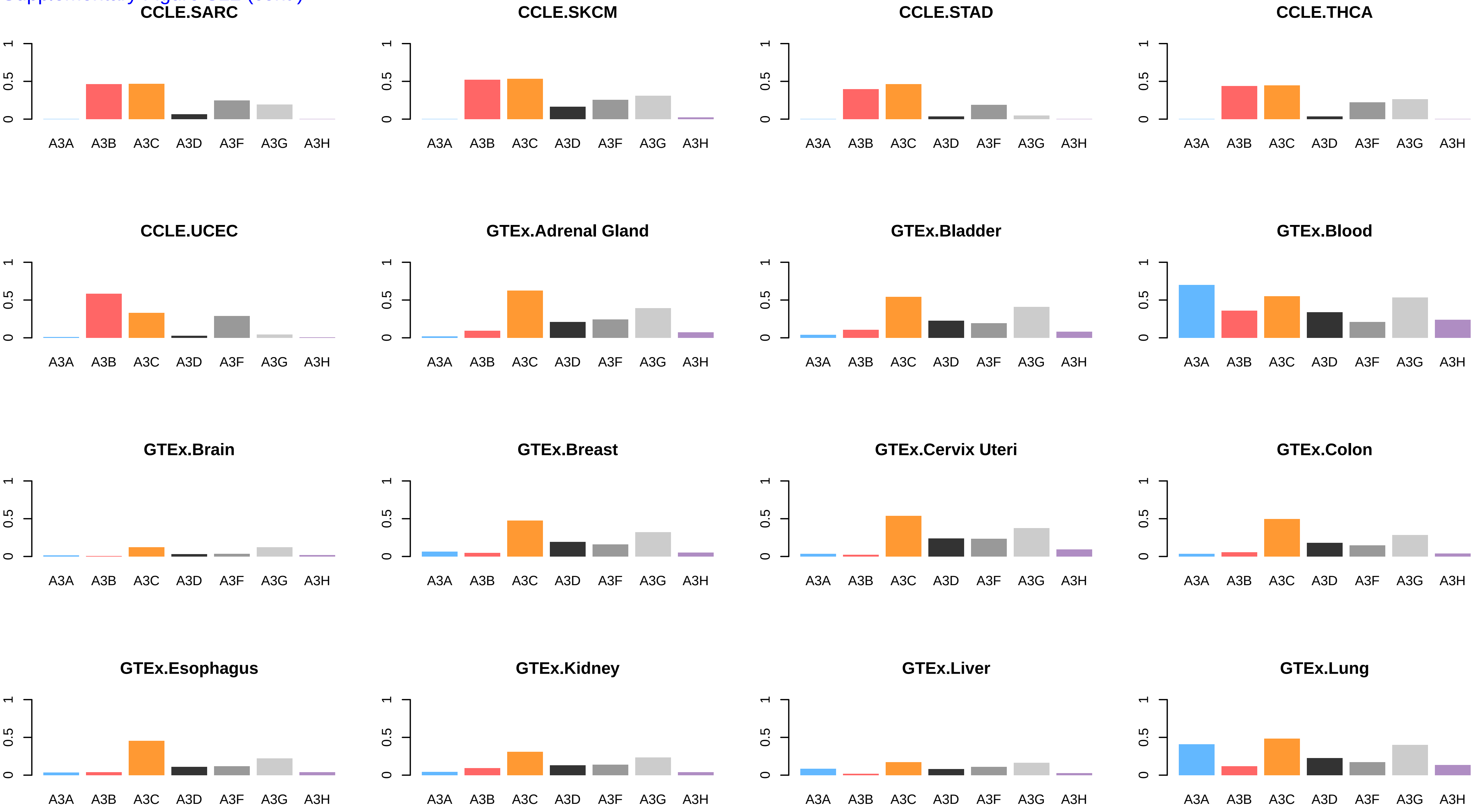

y-axis: median expression value (log2, relative to GAPDH)

Supplementary Figure S22 (cont.)

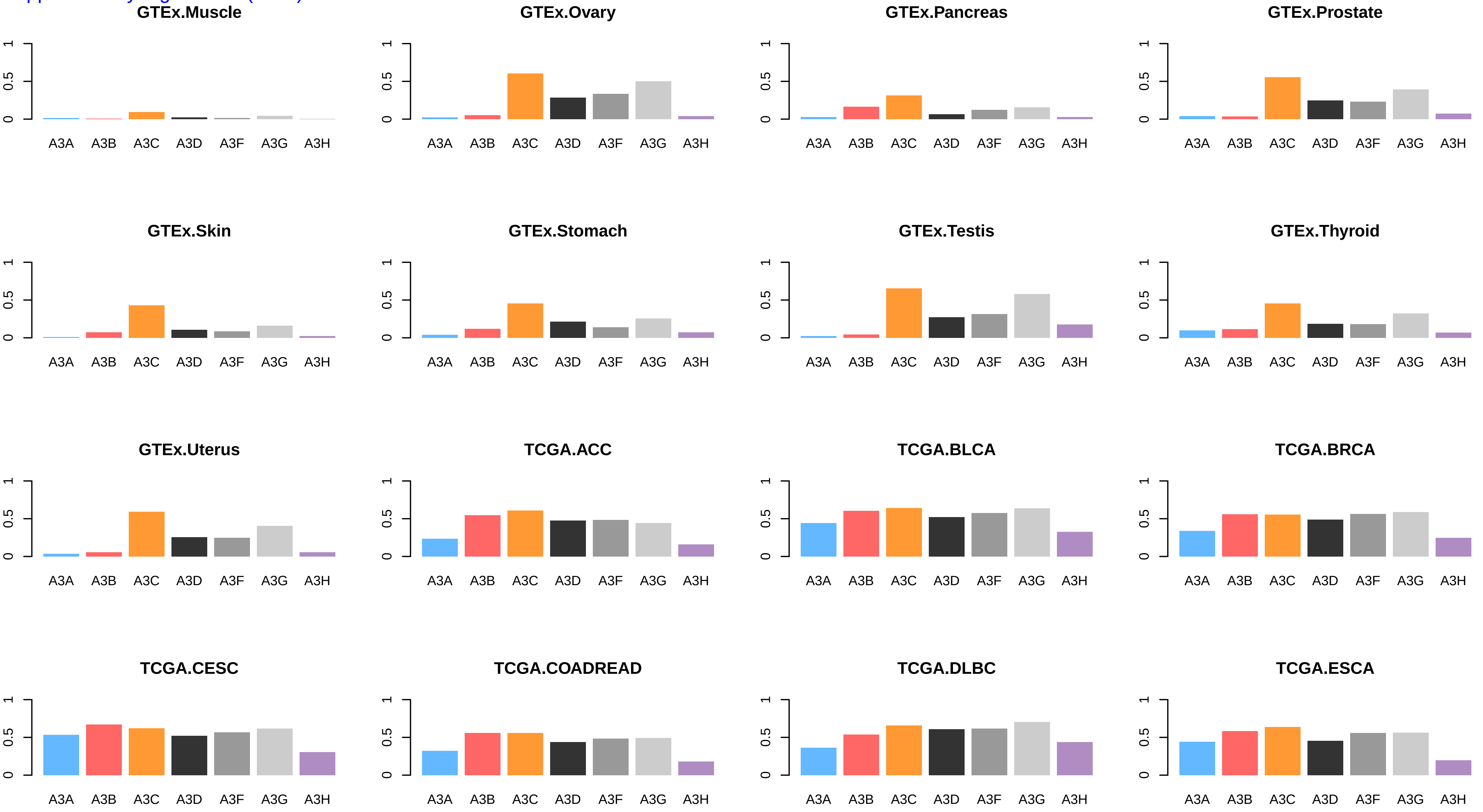

y-axis: median expression value (log2, relative to GAPDH)

Supplementary Figure S22 (cont.)

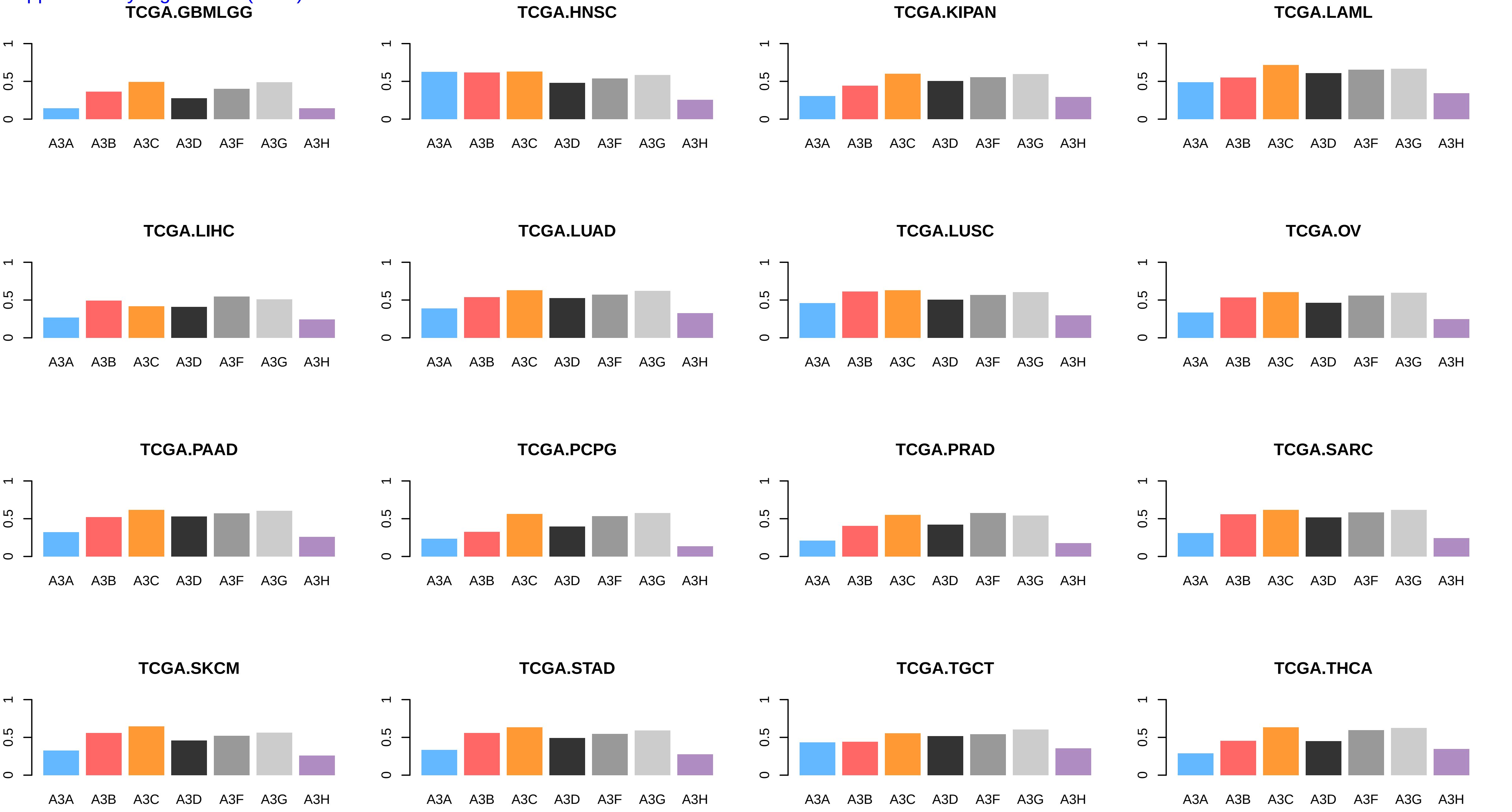

y-axis: median expression value (log2, relative to GAPDH)

Supplementary Figure S22 (cont.)

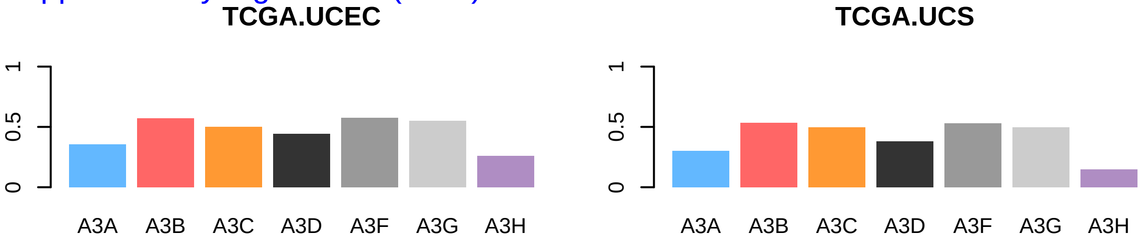

y-axis: median expression value (log2, relative to GAPDH)

Supplementary Figure S22. Bar-plots of APOBEC3 gene expression values across all examined cohorts

These plots correspond to Figure 8B (labels “Normal tissues” and “Cancer cell lines”). Data were identical to those displayed in the heatmap in main text Figure 1. All gene expression values were median of the respective cohort, log2-transformed and relative to the *GAPDH* gene. For the sake of completion median expression profiles in all TCGA cohorts are also included in this document, but they are not illustrated in main text Figure 8: the tumour expression there profiles are the RESPECTEx-reconstituted one corresponding to Figure 4B and Supplementary Figure S23.

Supplementary Figure S23

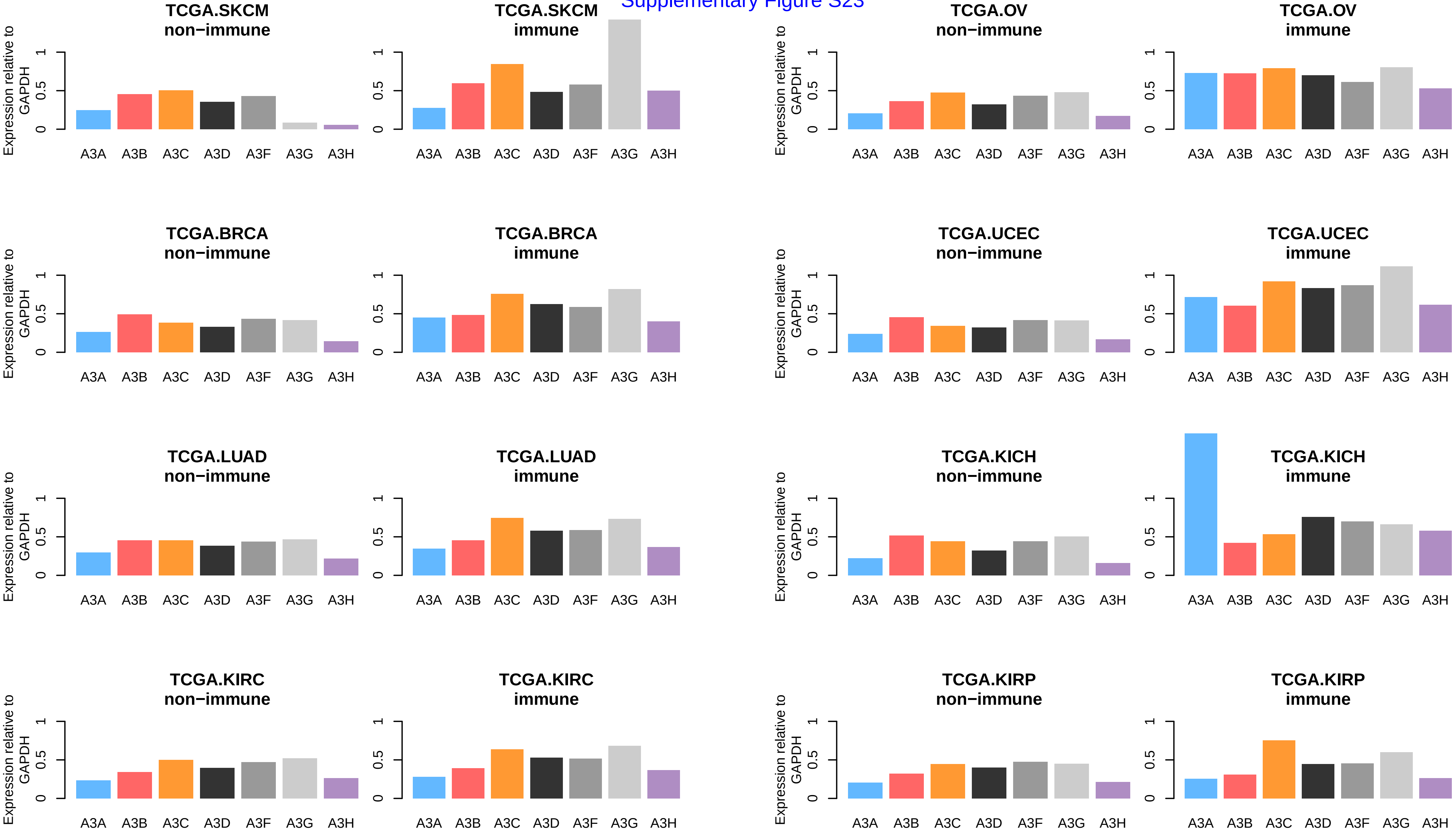

Supplementary Figure S23 (cont.)

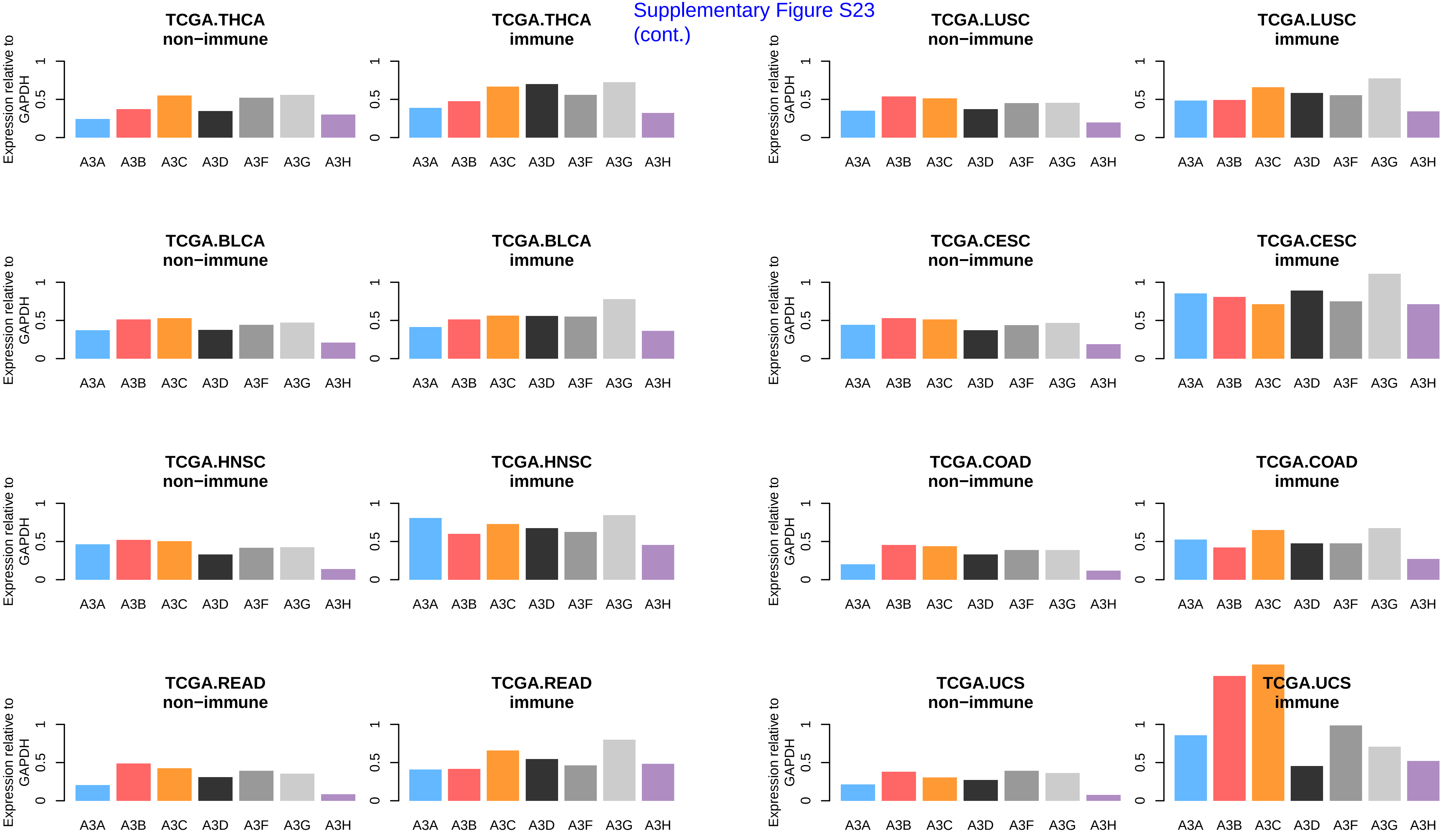

Supplementary Figure S23 (cont.)

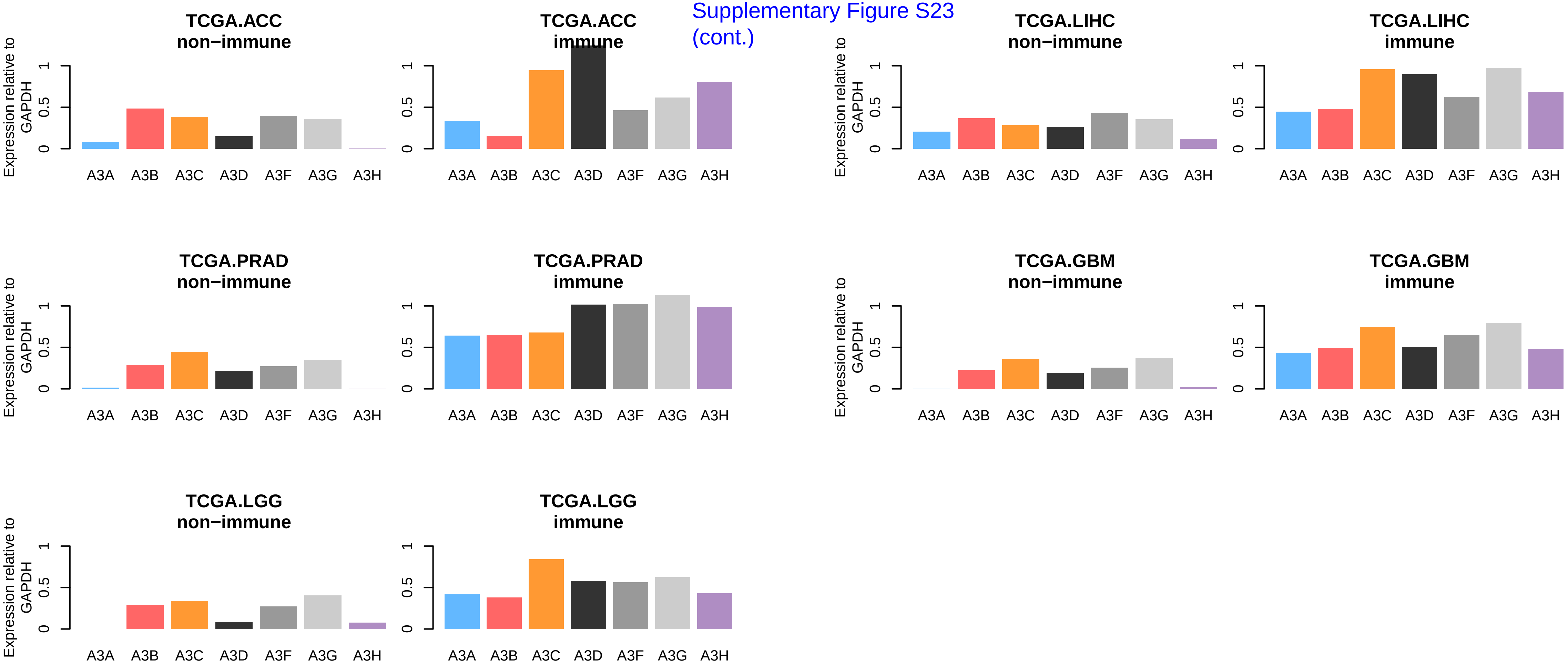

Supplementary Figure S23 (cont.)

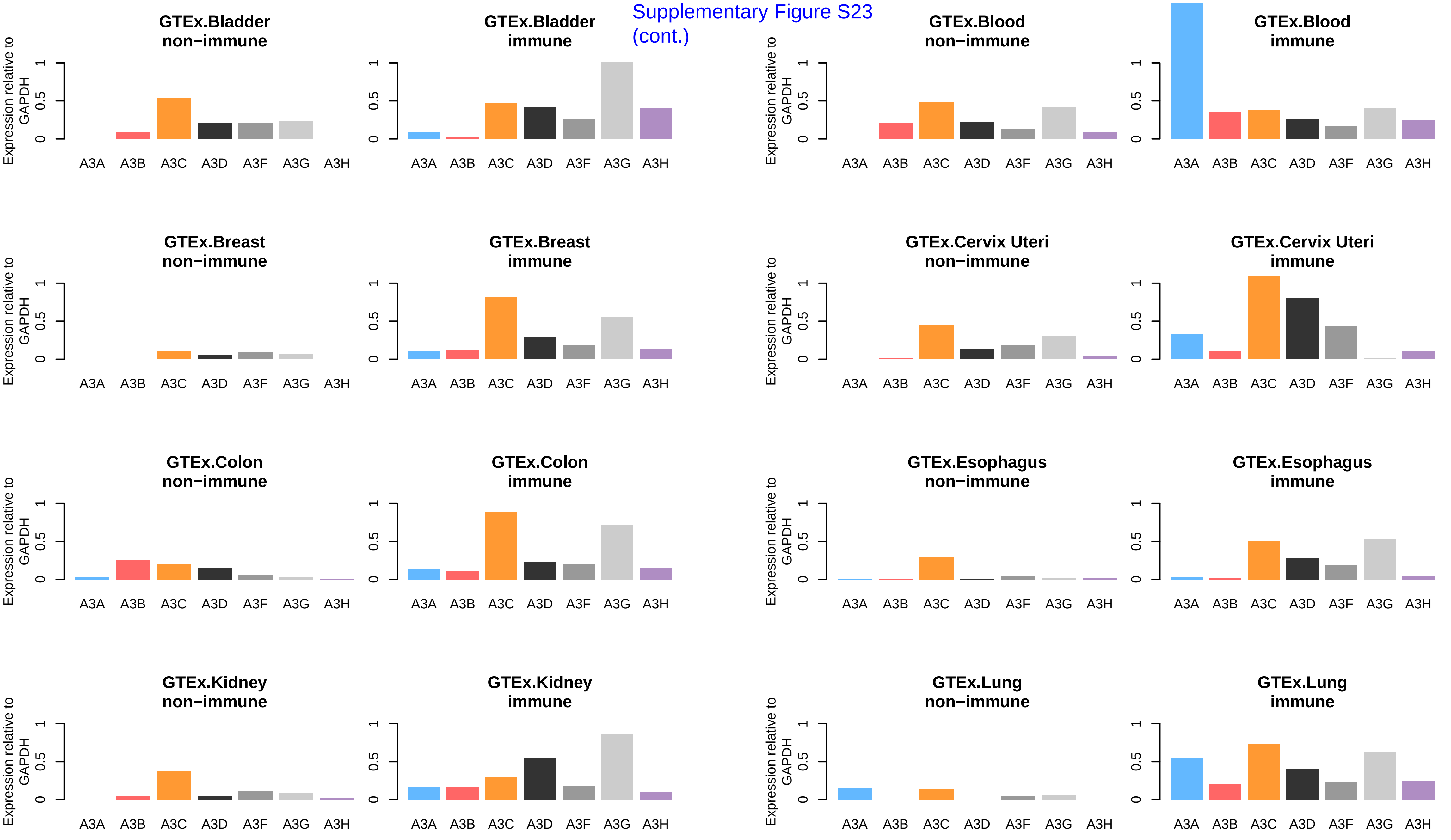

Supplementary Figure S23 (cont.)

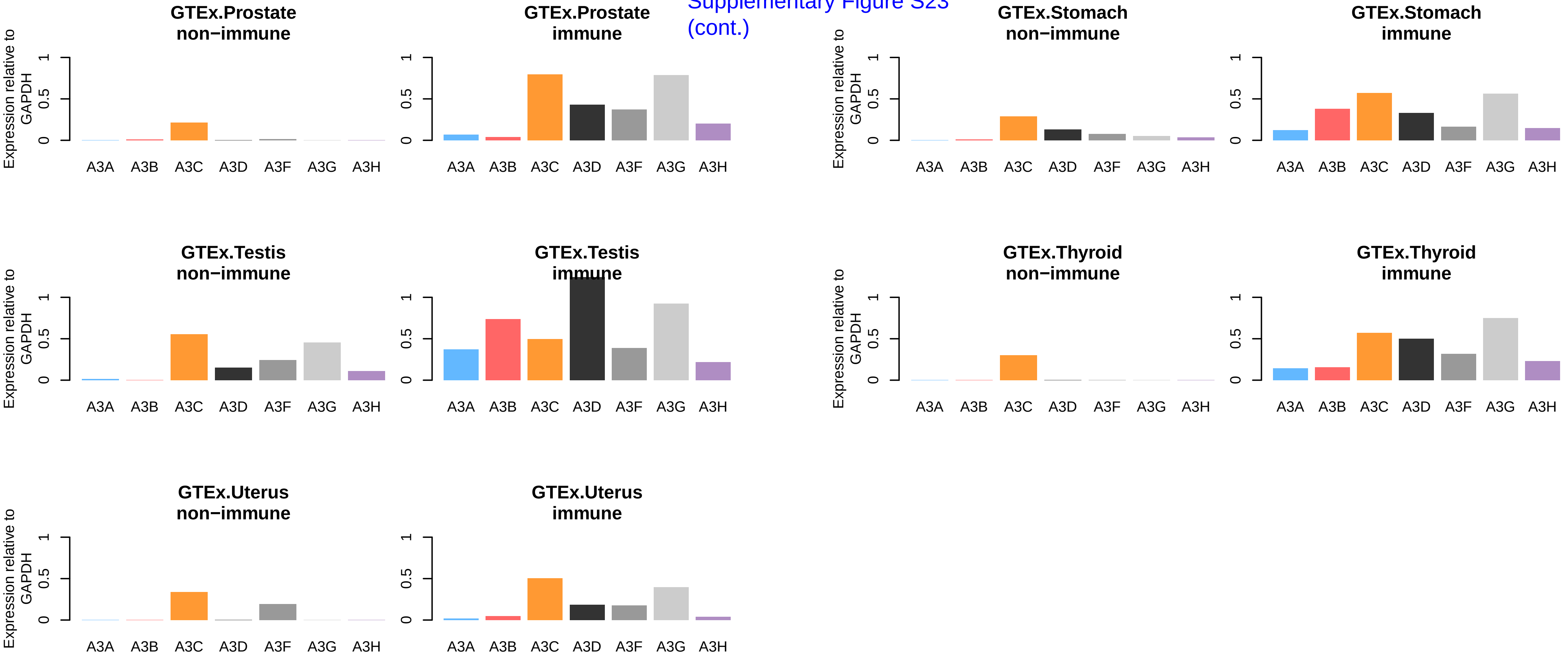

Supplementary Figure S23. Bar-plots of APOBEC3 gene expression values reconstituted using RESPECTEx for immune and non-immune components of TCGA and GTEx samples

These plots correspond to Figure 8B (labels "Tumours"). The same data were used to derive the non-immune/immune ratio illustrated in Figures 4A-B. All gene expression values were normalised to the *GAPDH* gene.

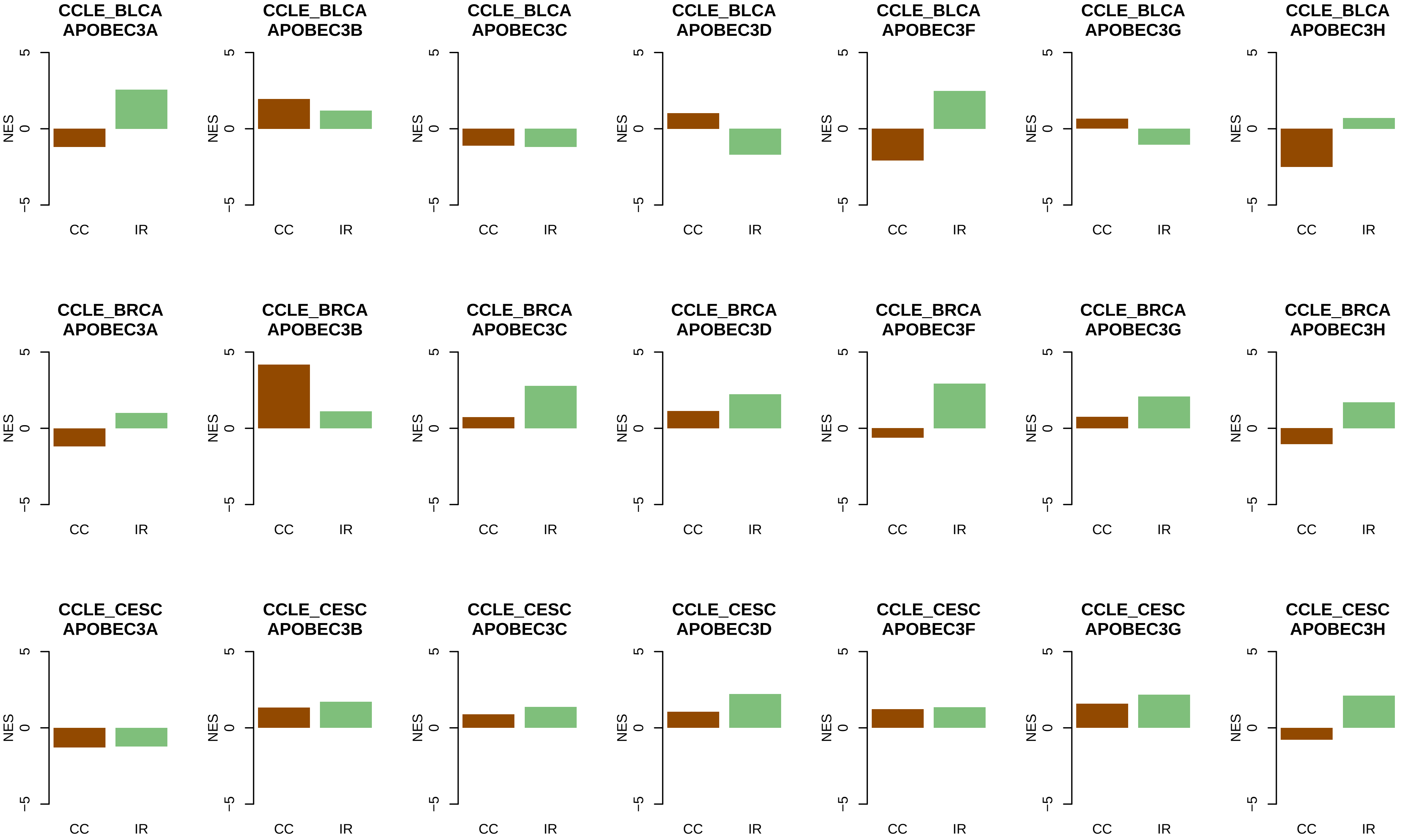

Supplementary Figure S24

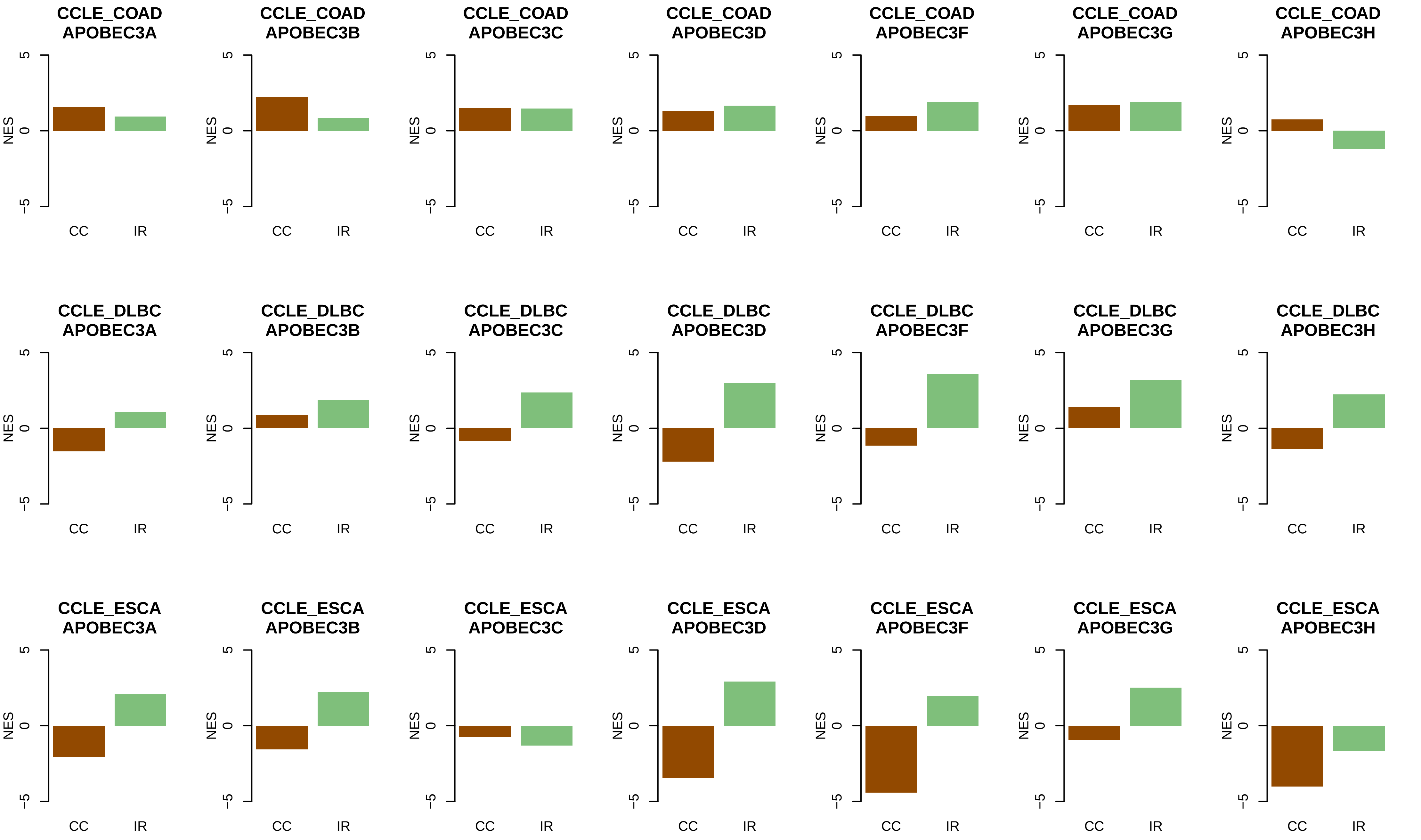

Supplementary Figure S24 (cont.)

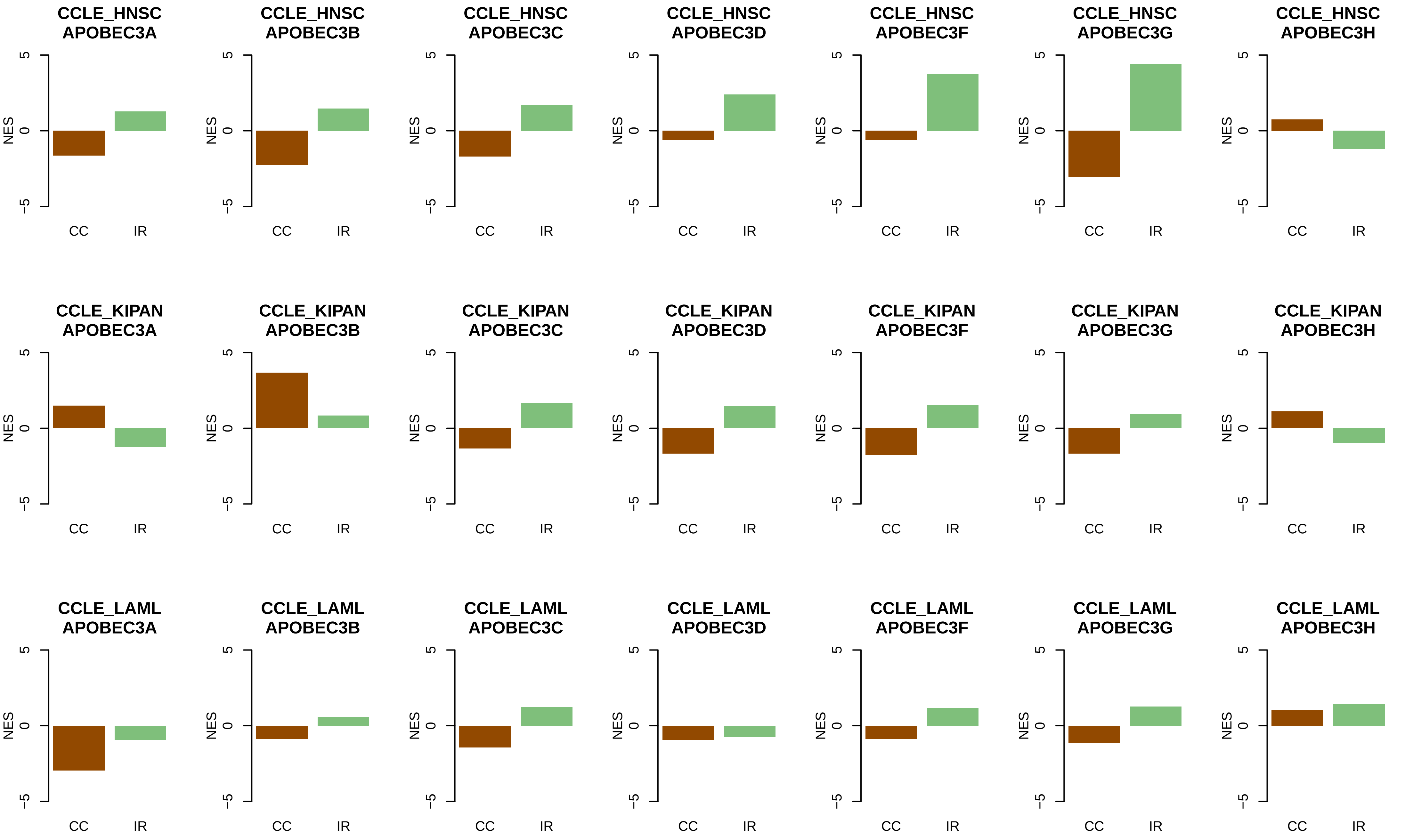

Supplementary Figure S24 (cont.)

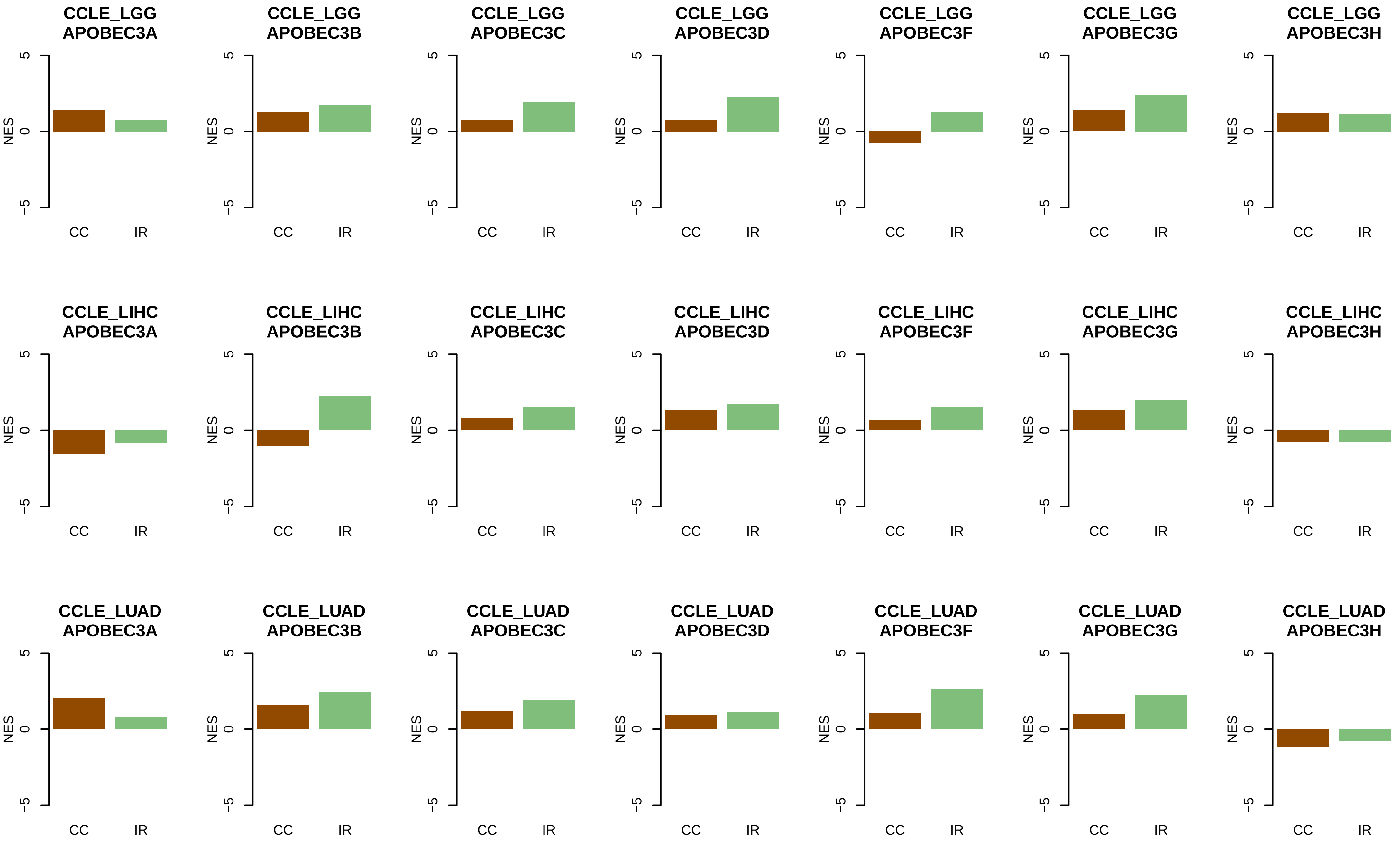

Supplementary Figure S24 (cont.)

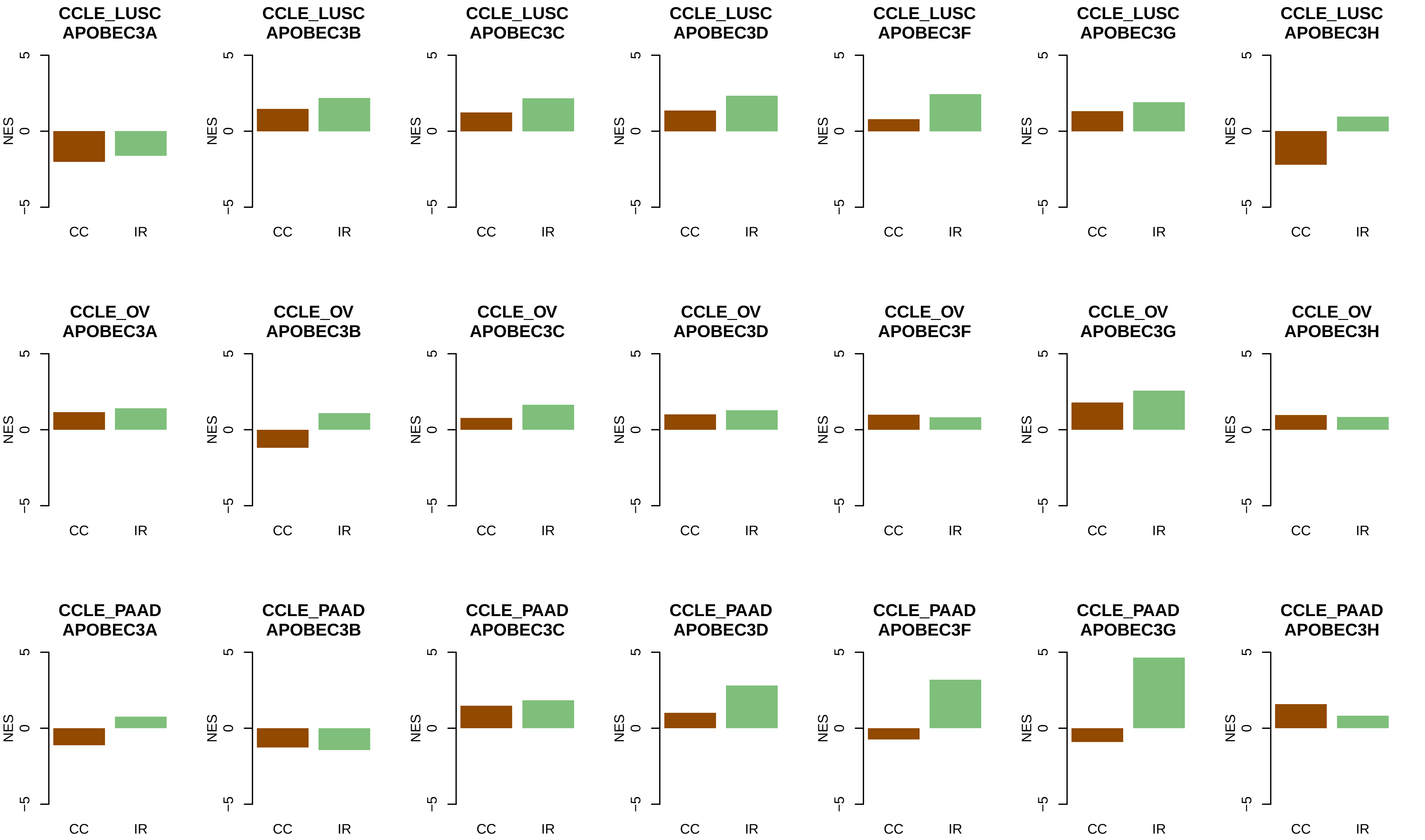

Supplementary Figure S24 (cont.)

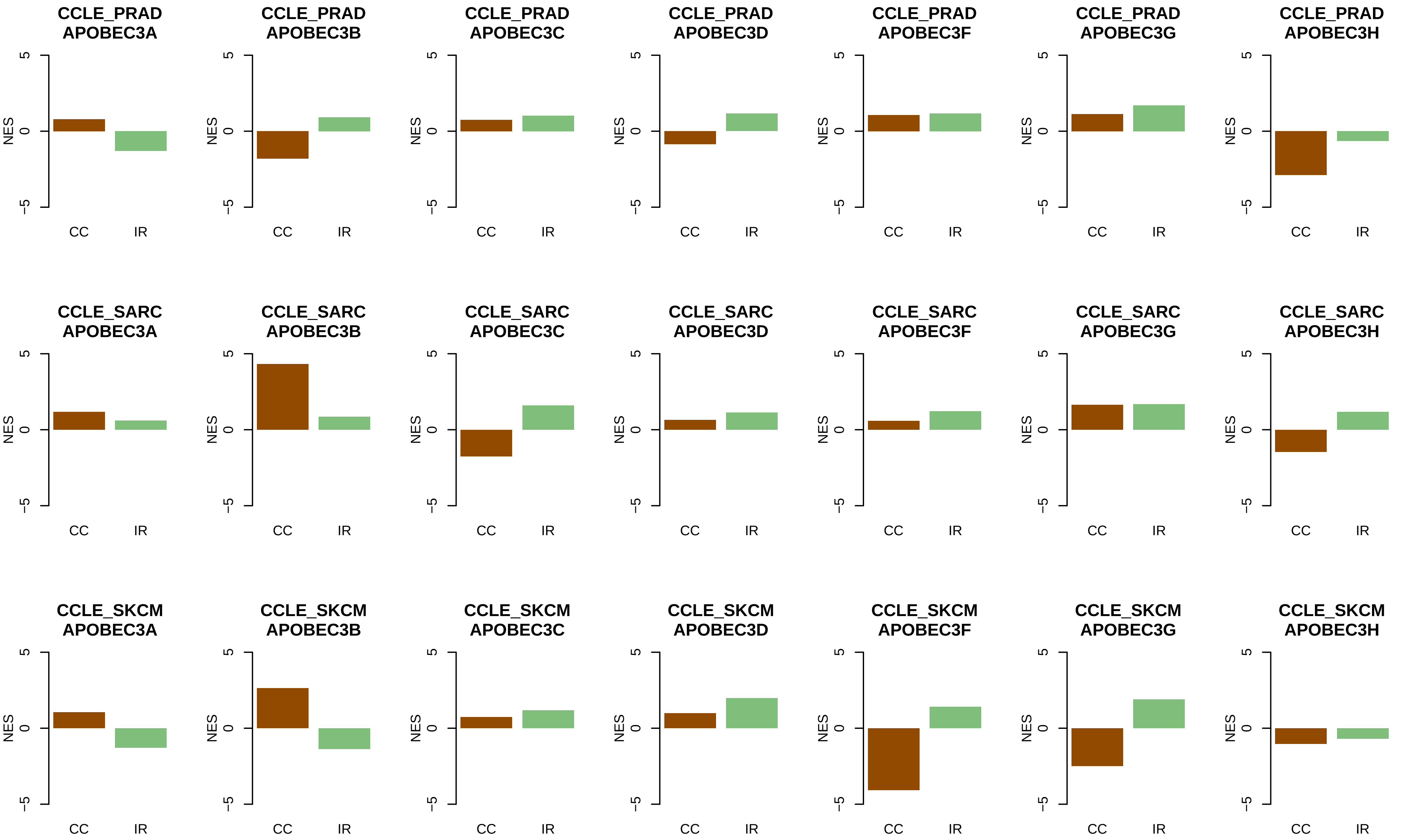

Supplementary Figure S24 (cont.)

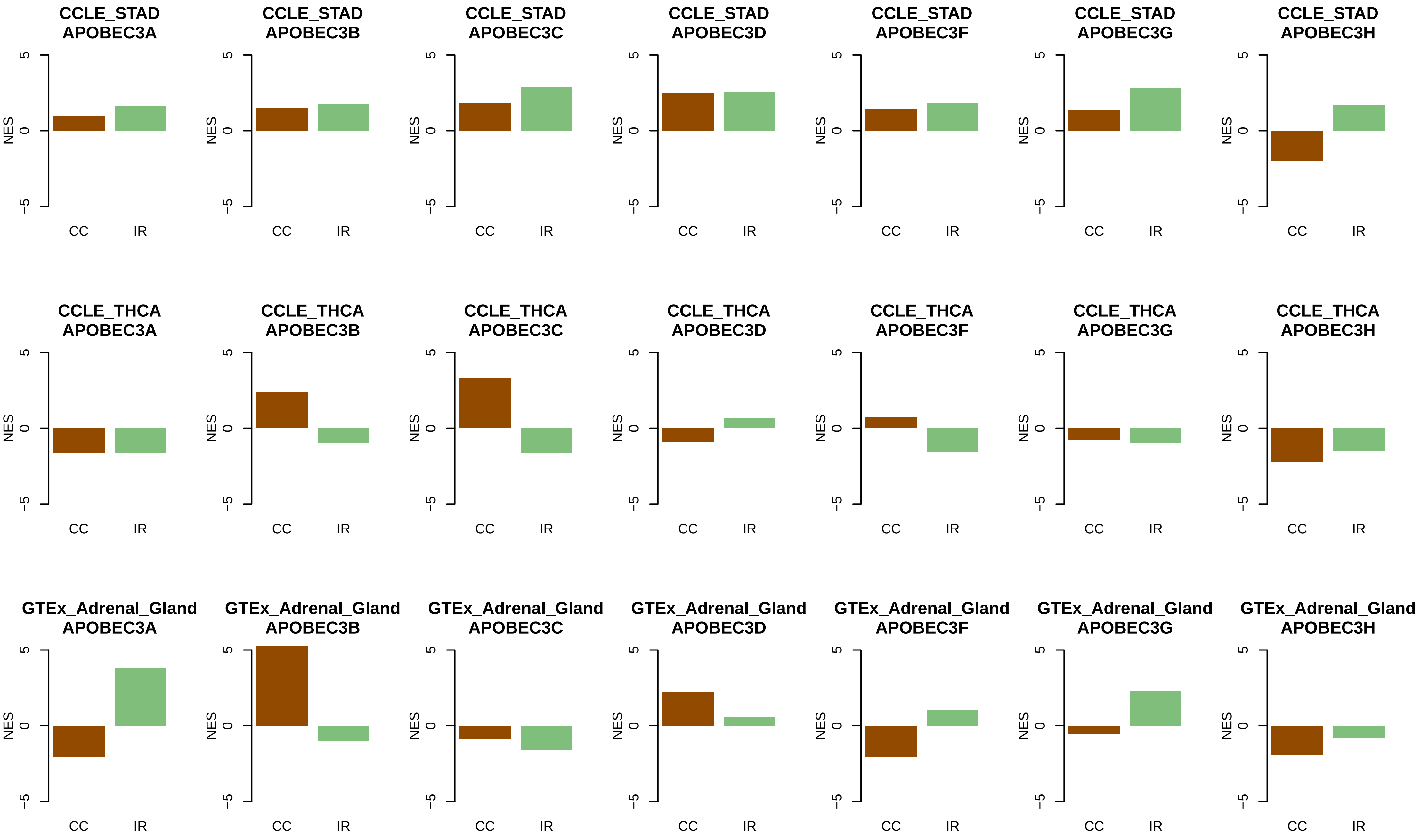

Supplementary Figure S24 (cont.)

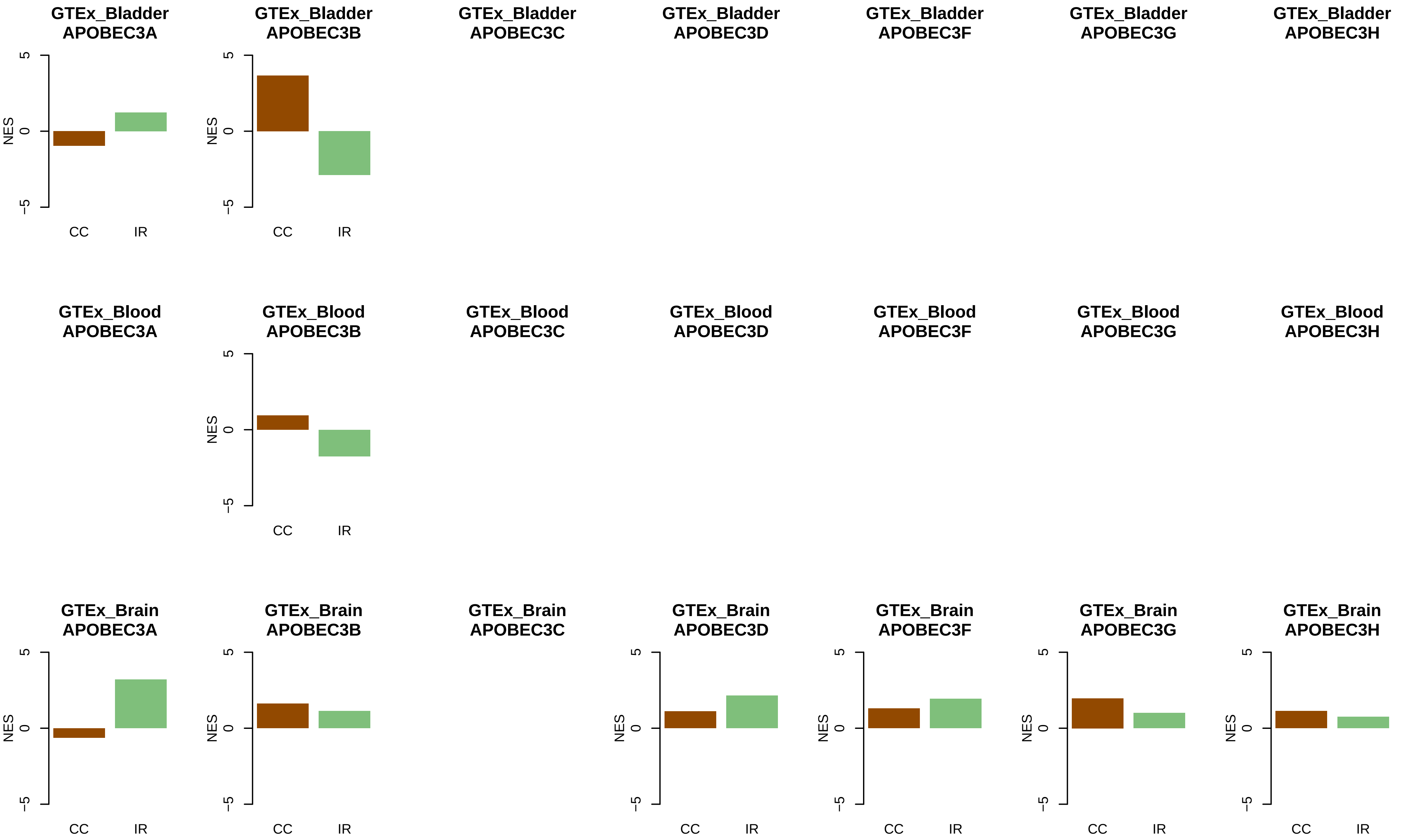

Supplementary Figure S24 (cont.)

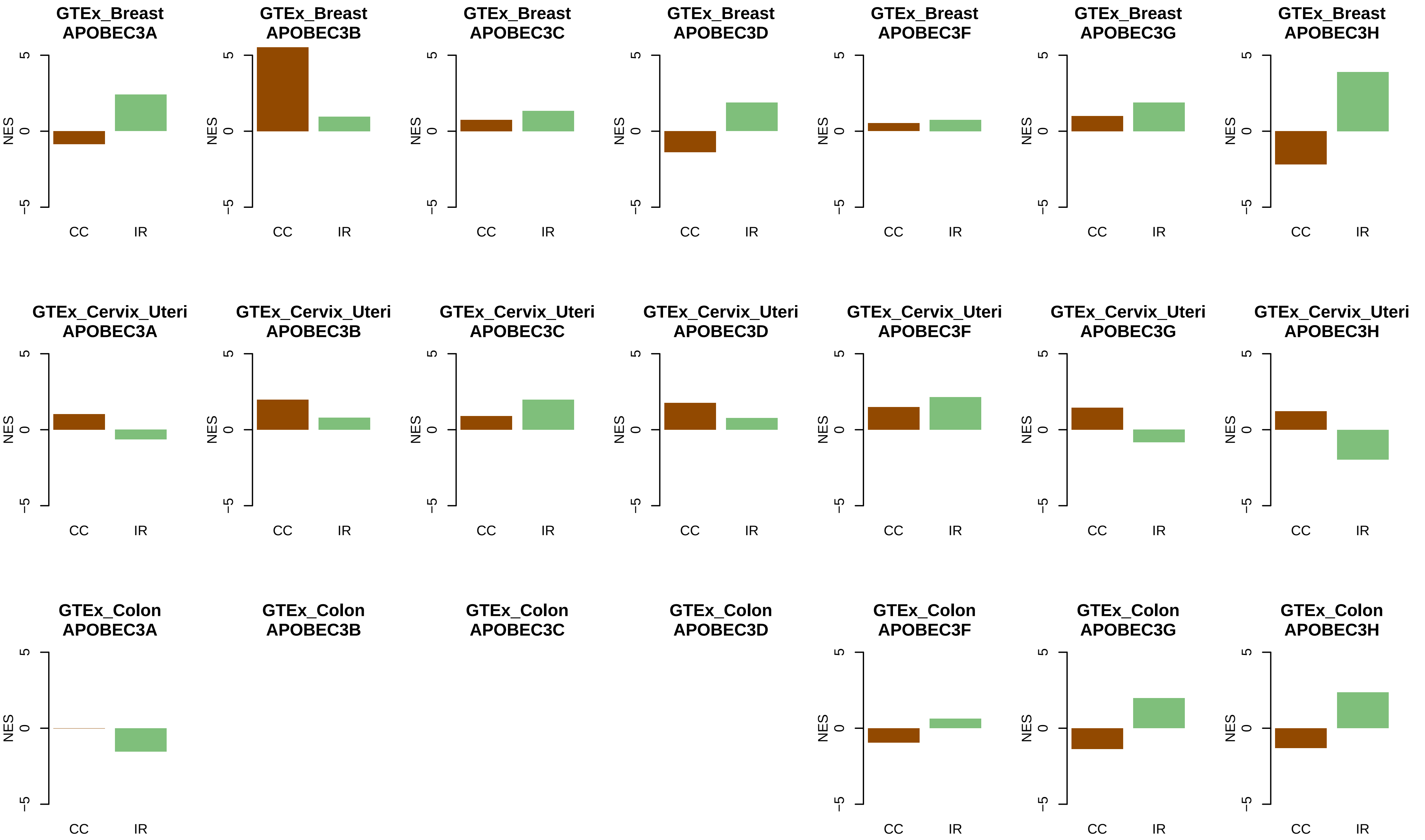

Supplementary Figure S24 (cont.)

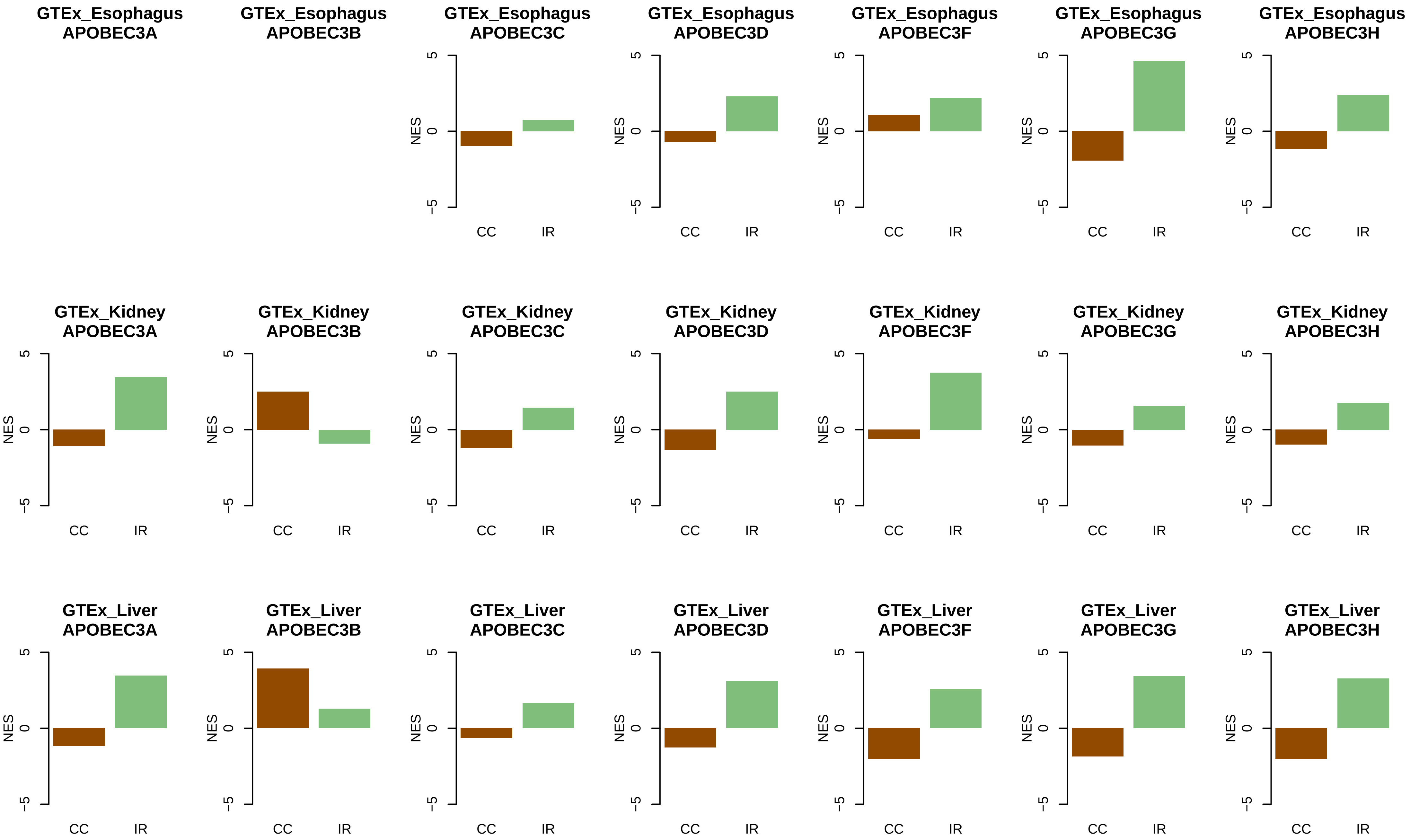

Supplementary Figure S24 (cont.)

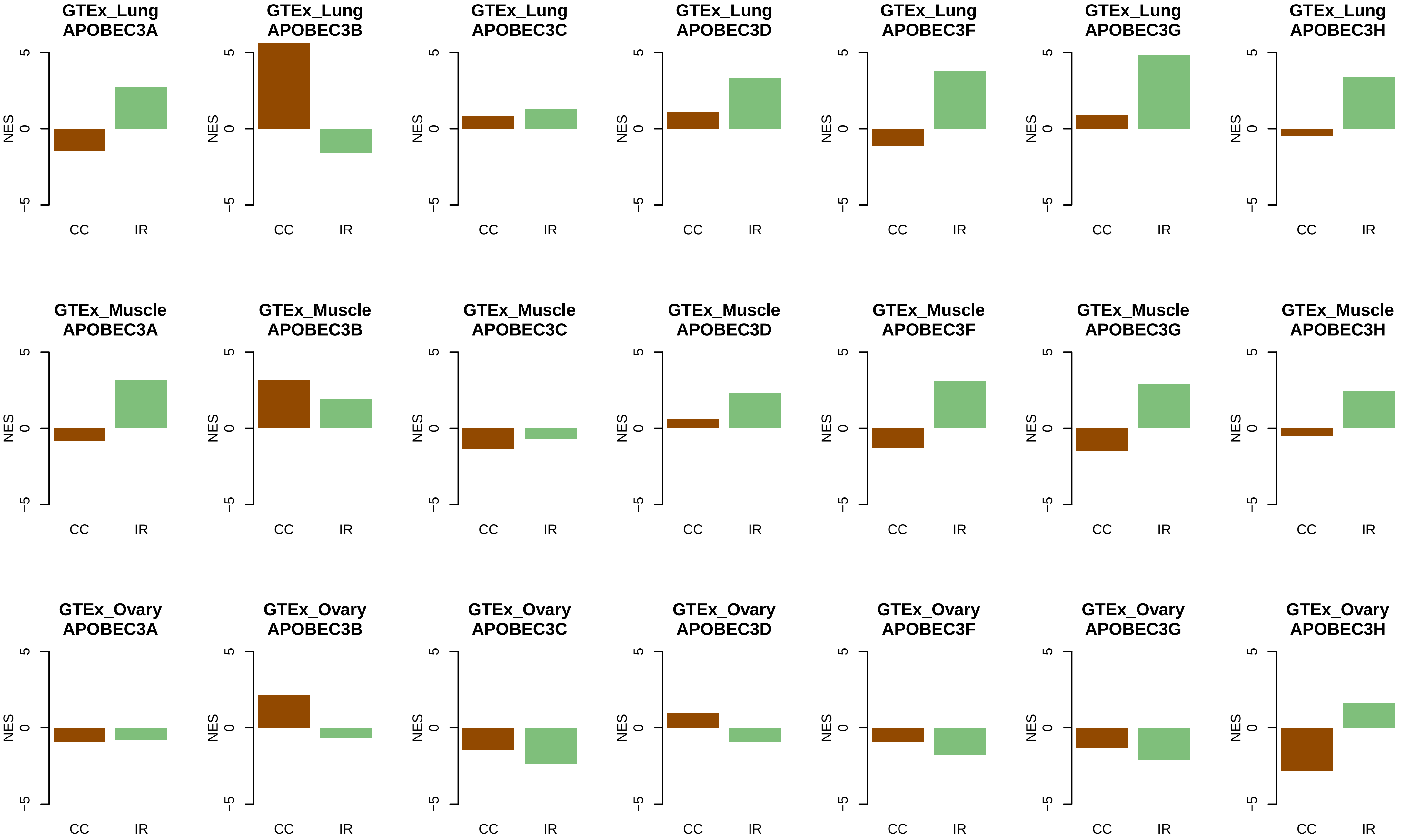

Supplementary Figure S24 (cont.)

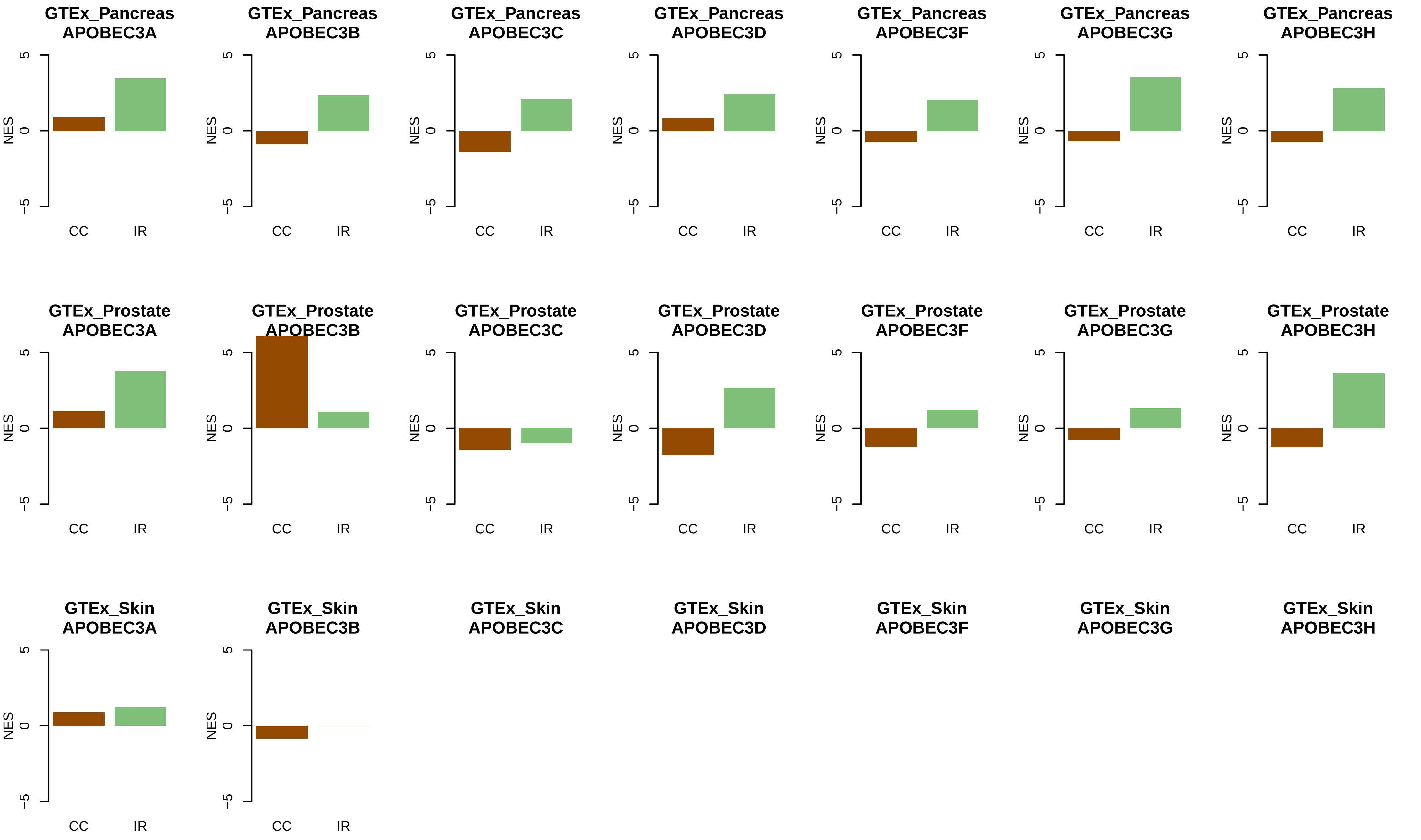

Supplementary Figure S24 (cont.)

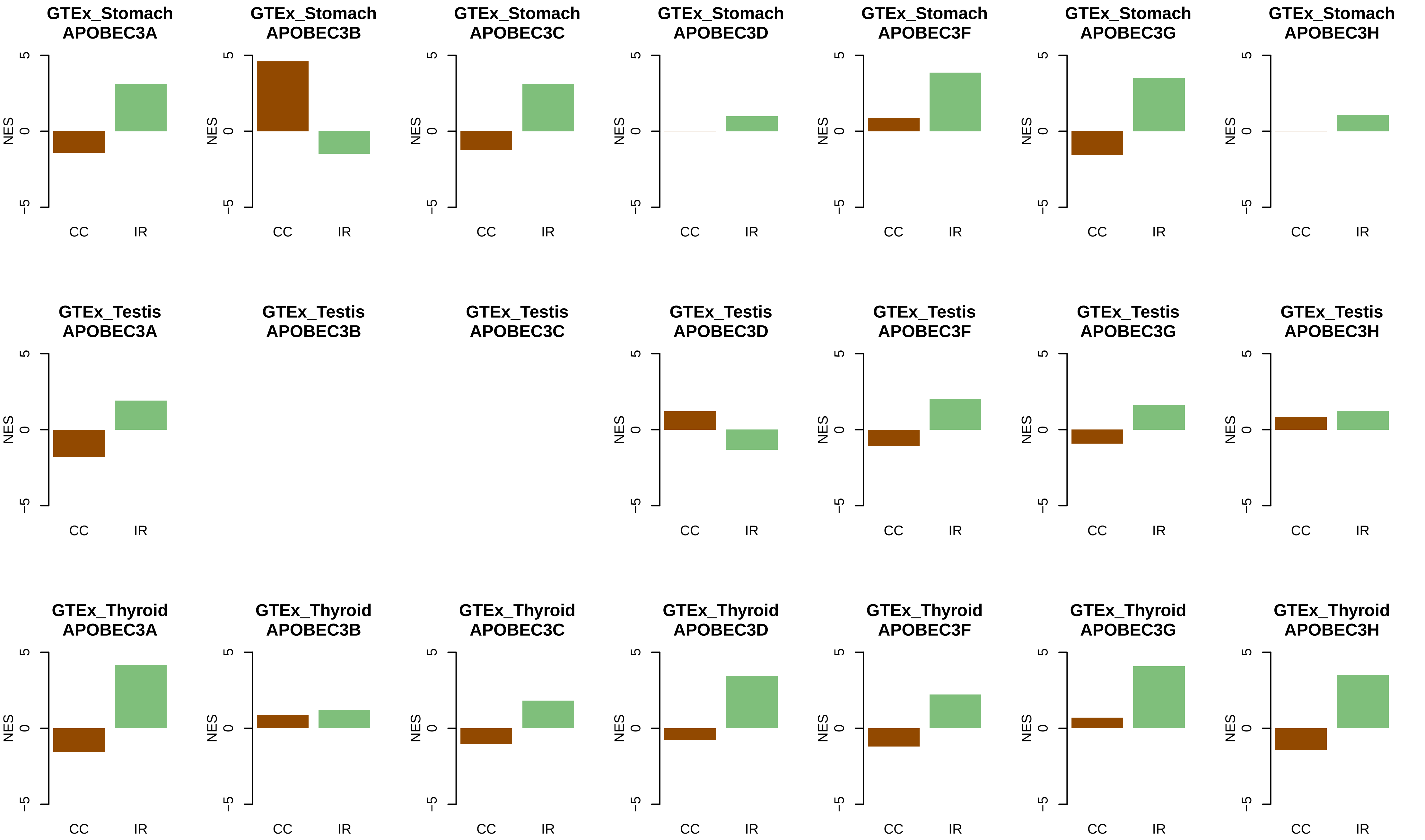

Supplementary Figure S24 (cont.)

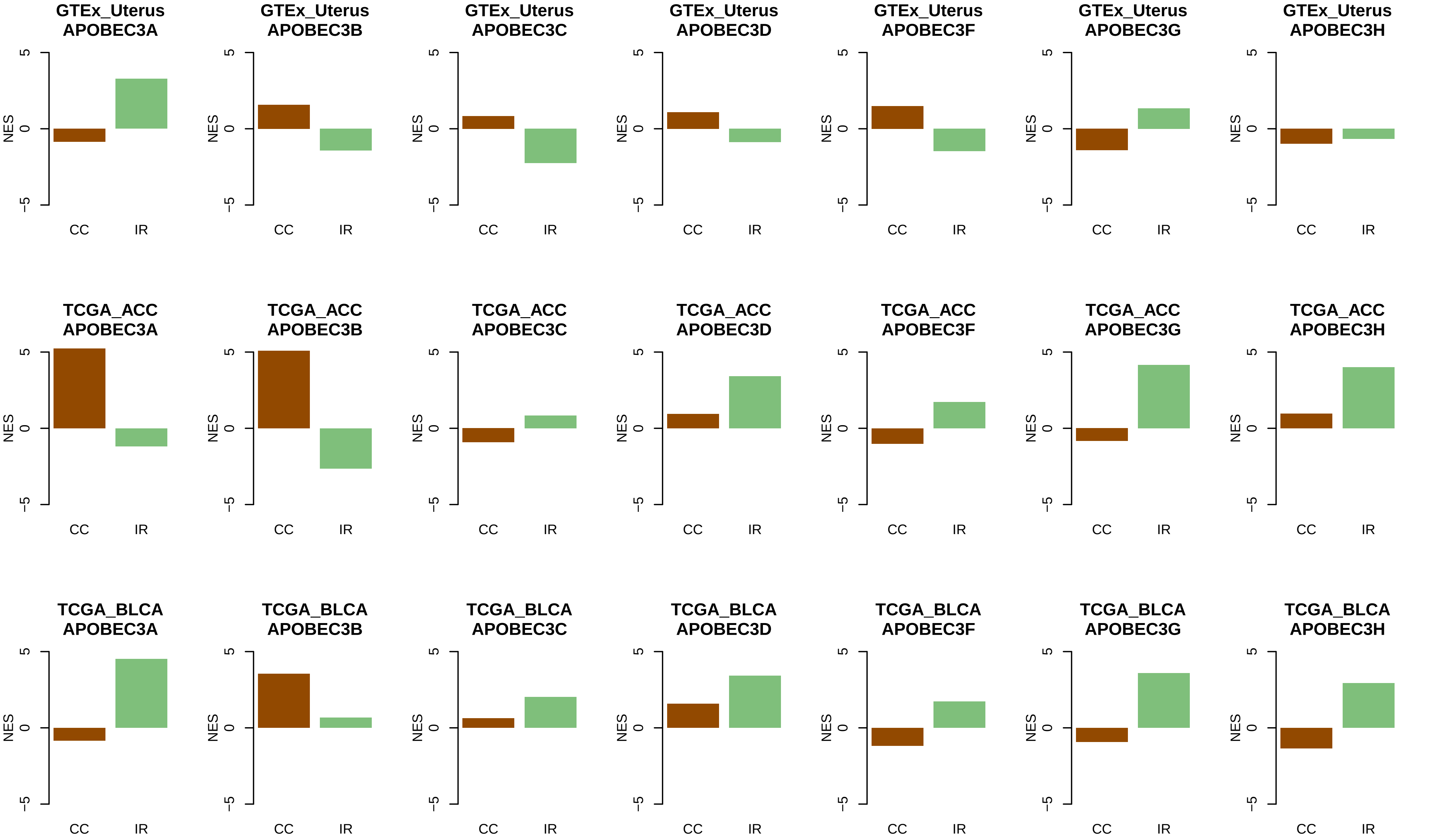

Supplementary Figure S24 (cont.)

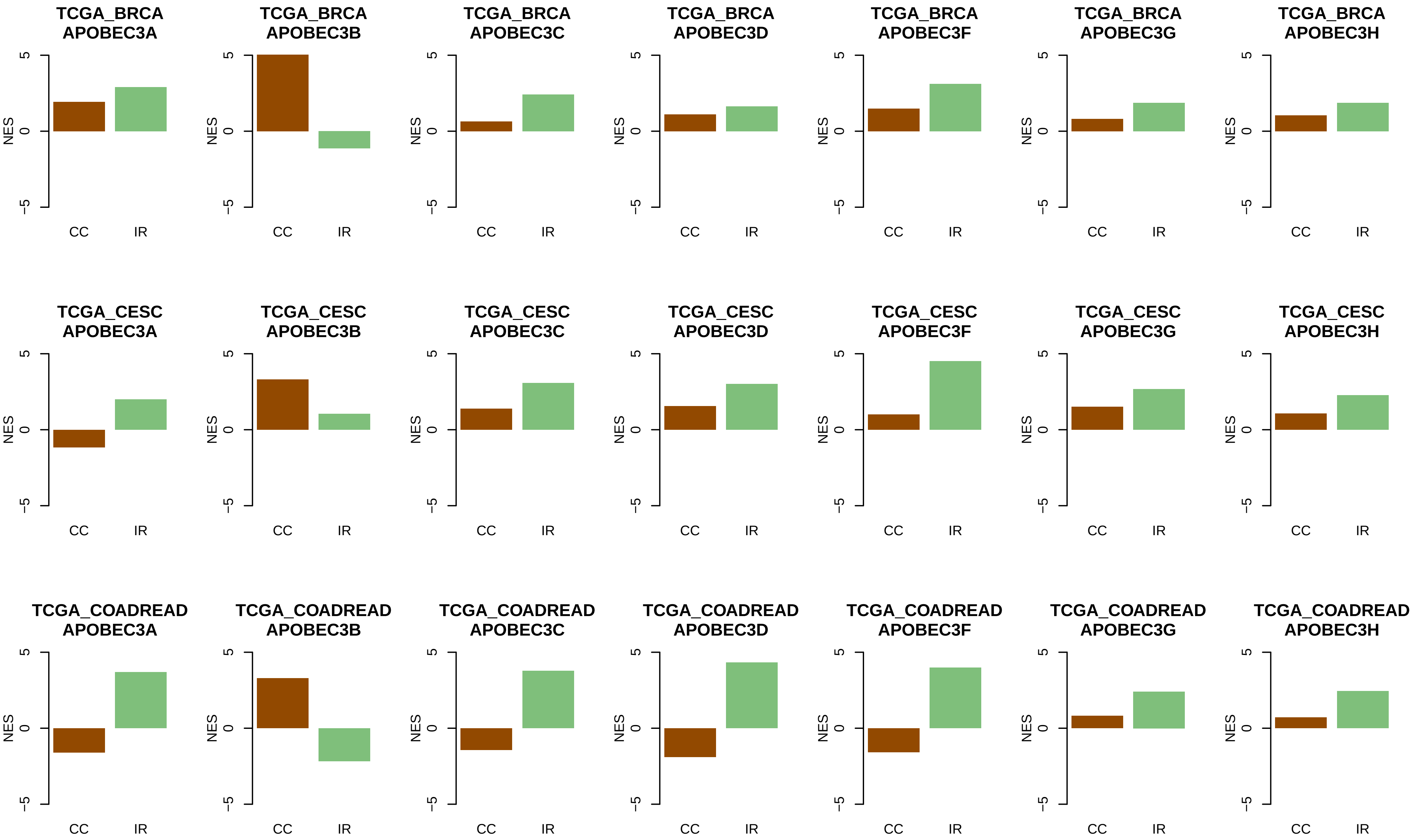

Supplementary Figure S24 (cont.)

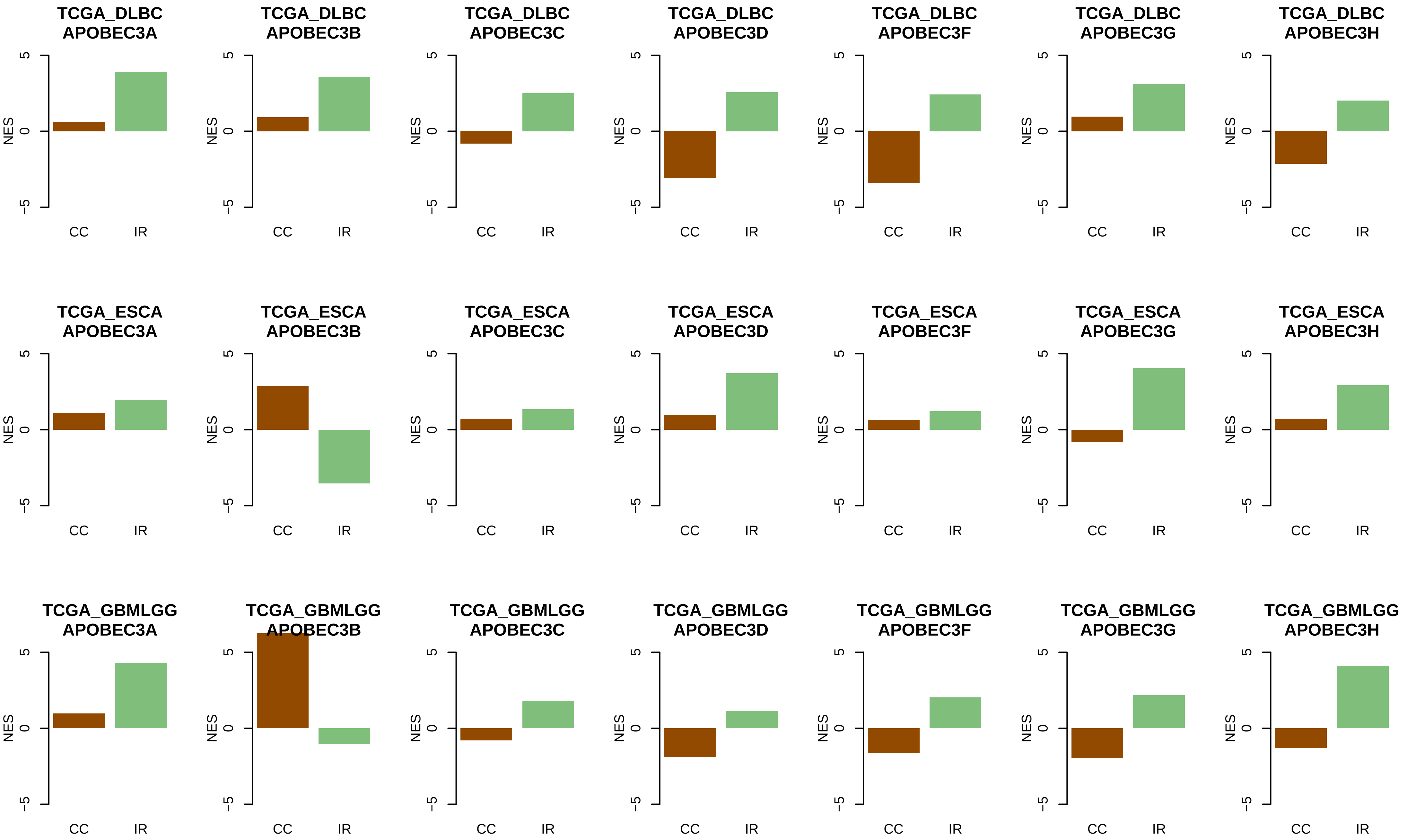

Supplementary Figure S24 (cont.)

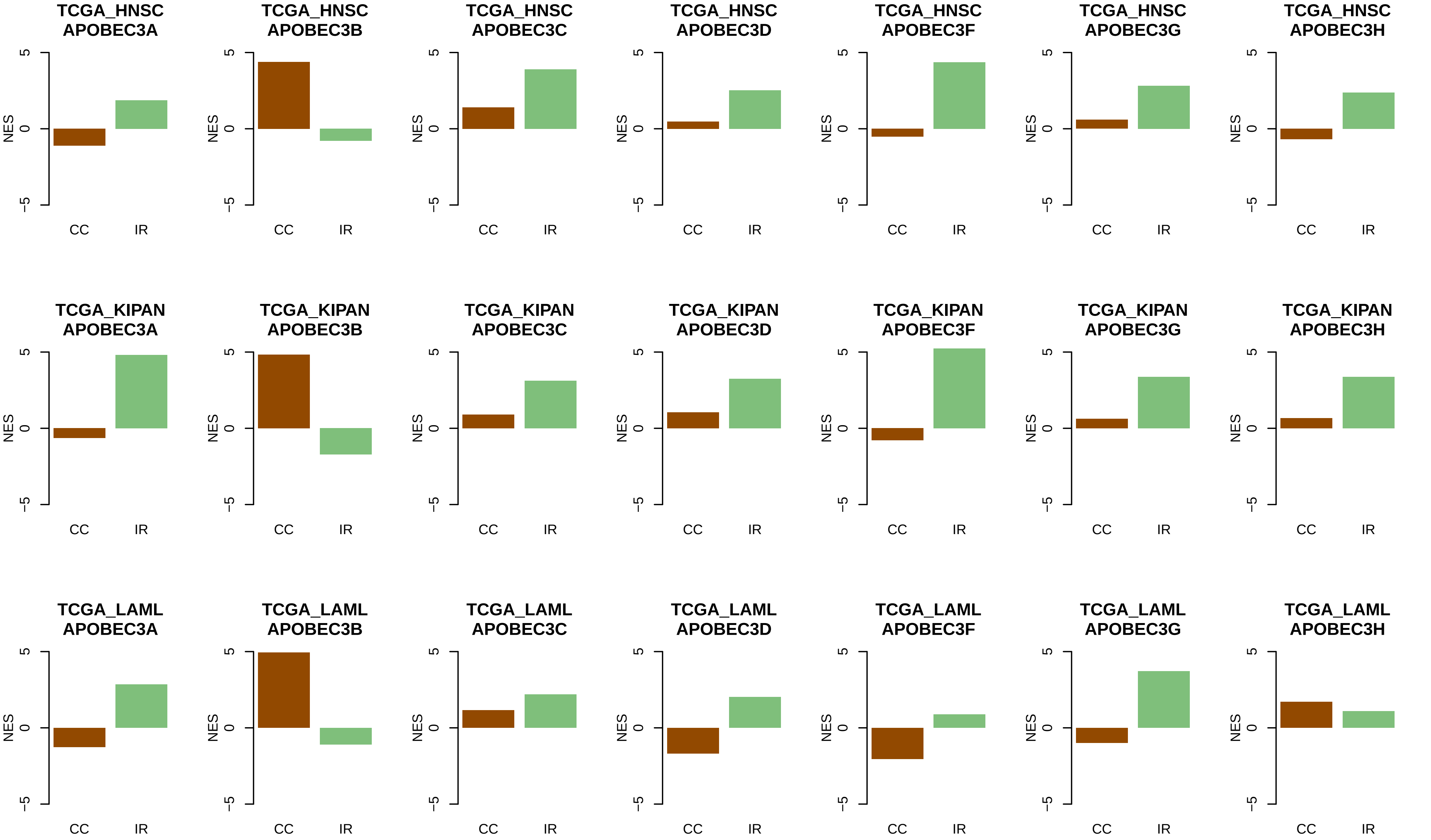

Supplementary Figure S24 (cont.)

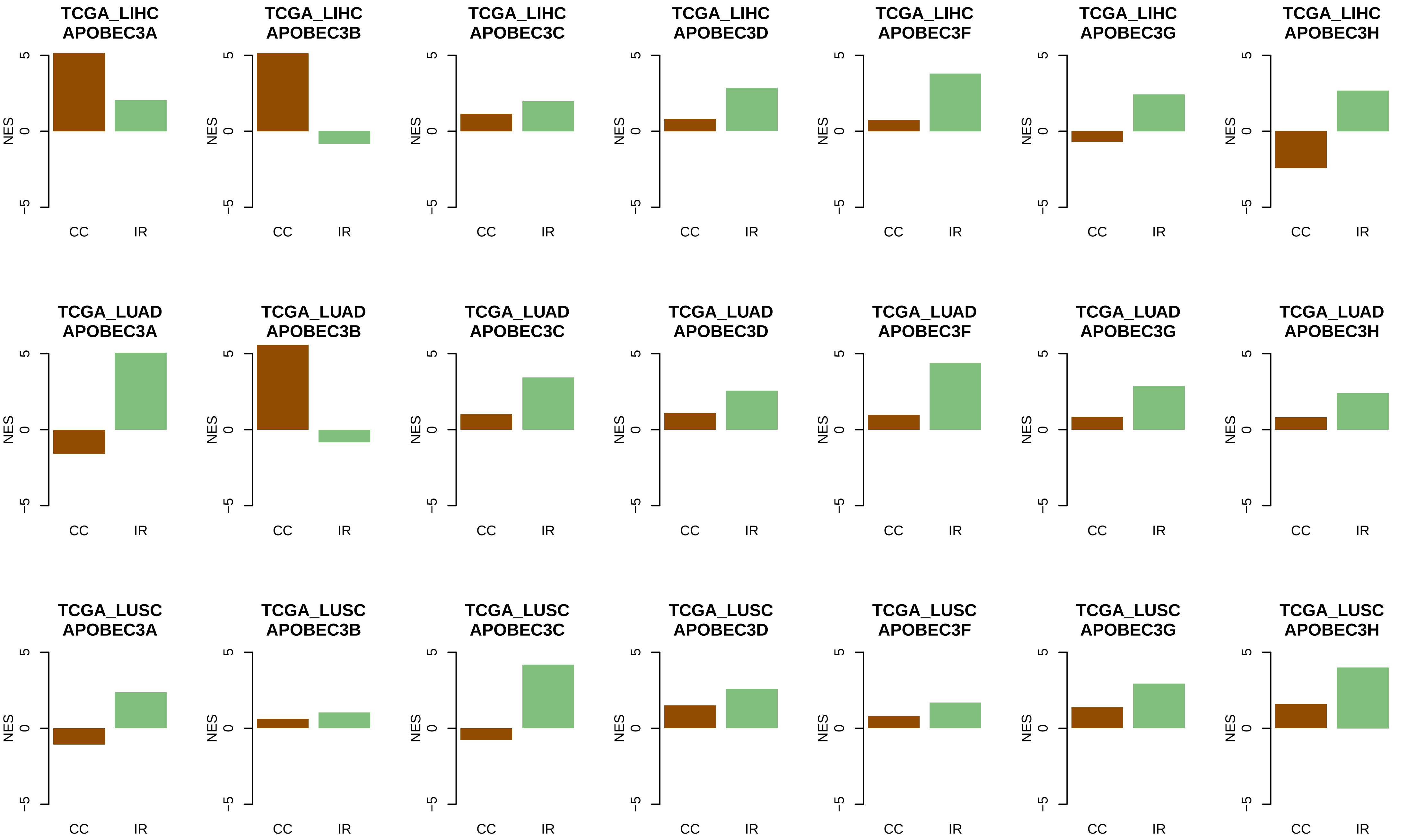

Supplementary Figure S24 (cont.)

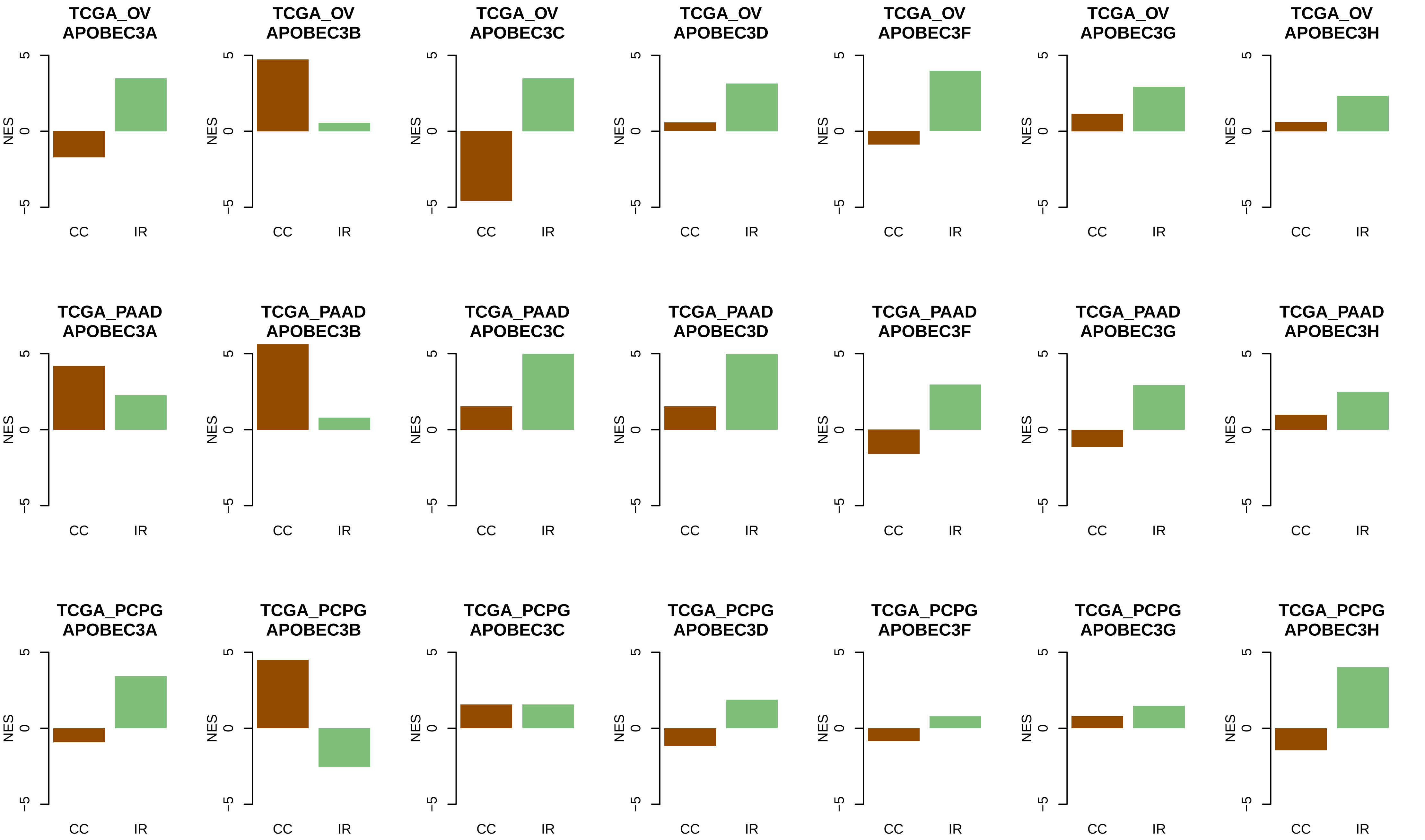

Supplementary Figure S24 (cont.)

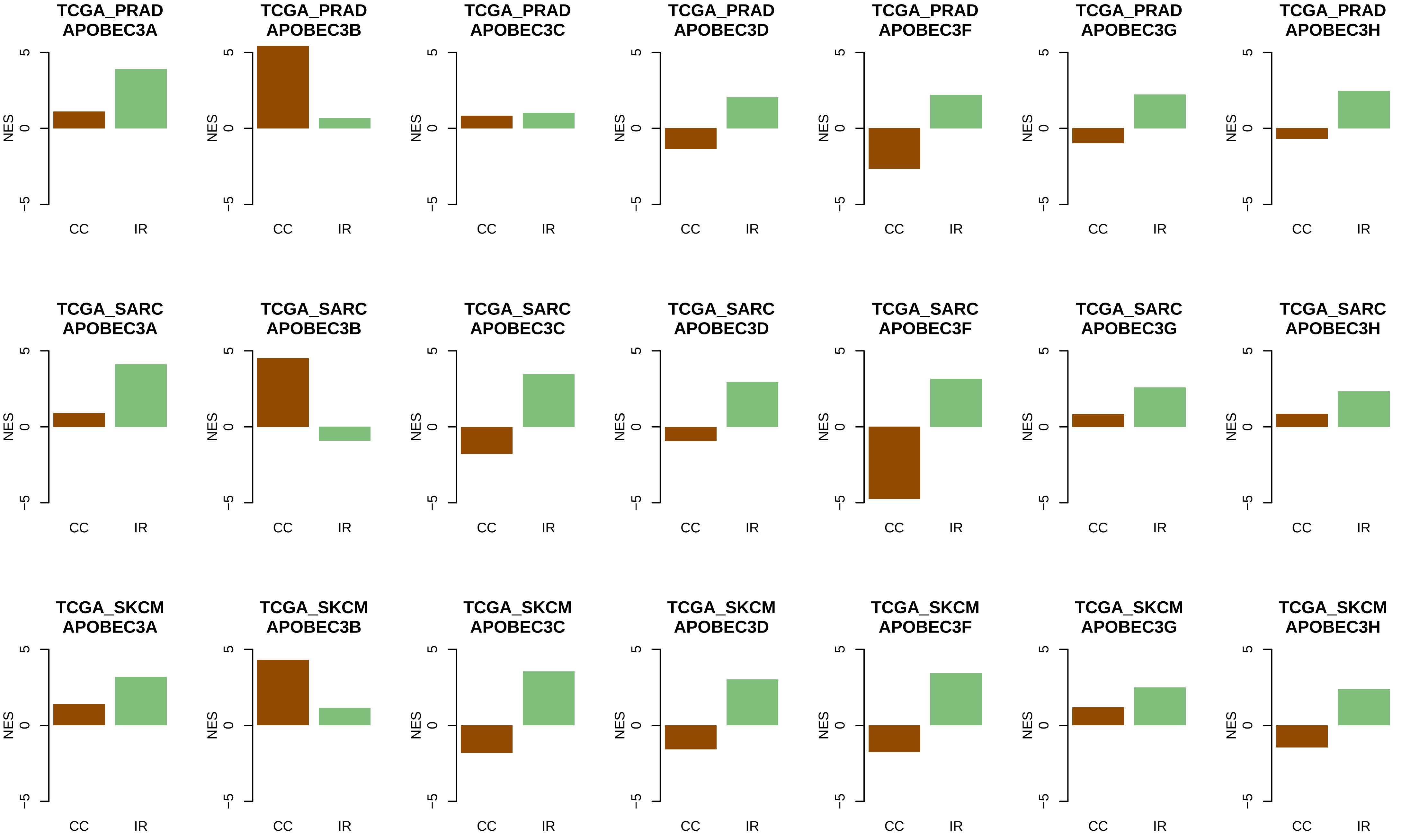

Supplementary Figure S24 (cont.)

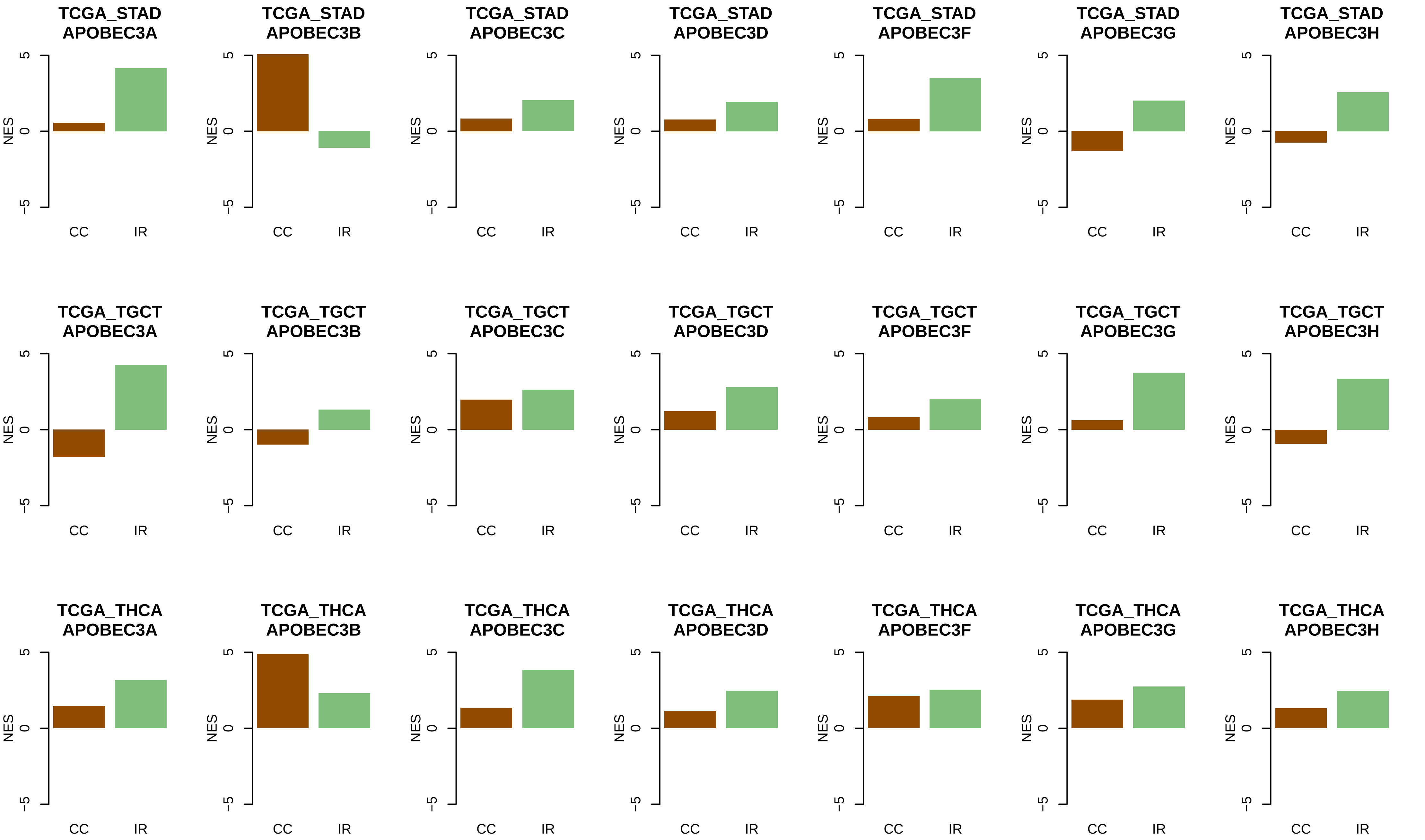

Supplementary Figure S24 (cont.)

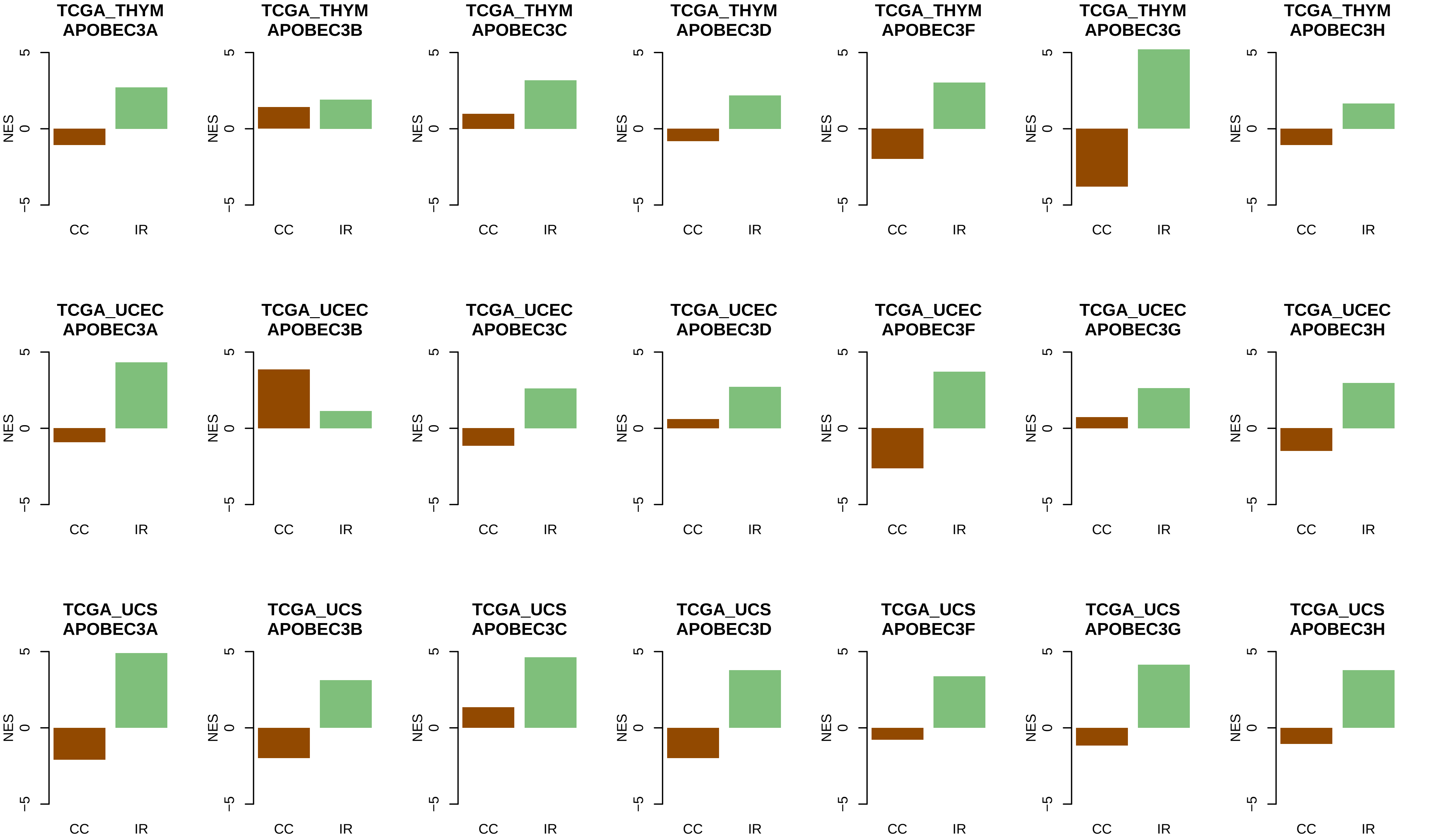

Supplementary Figure S24 (cont.)

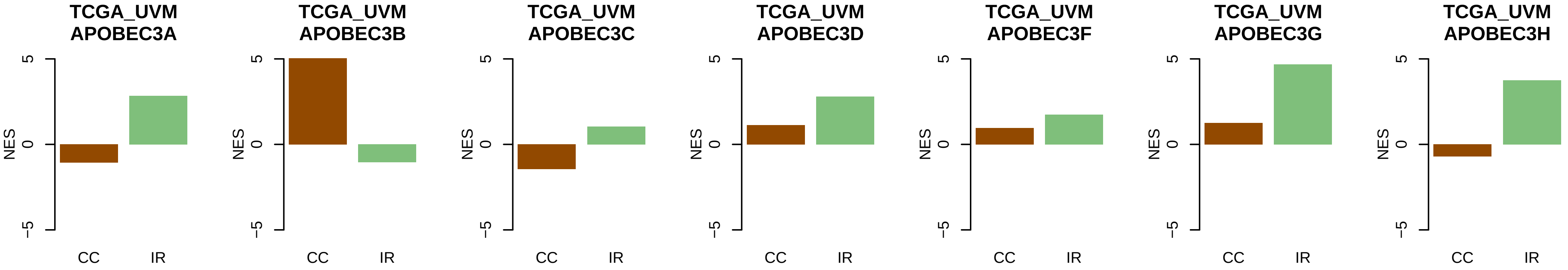

Supplementary Figure S24 (cont.)

Supplementary Figure S24. Bar-plots of Normalised Enrichment Score (NES) of GO Cell Cycle and Immune Responses gene sets for APOBEC3 co-expressing genes in all examined cohorts

These plots correspond to Figure 8C. The same GSEA procedure as in Figures 5-7 was used here. NES values from GSEA were used to generate the PCA plot in Figure 6C. Here NESs were extracted for the GO gene sets but displayed as a bar-plot. For some cohorts and specific APOBEC3 genes, no co-expressions satisfied the criteria (see Methods) for our extraction of gene co-expression, hence the empty panels in those cases. CC, Cell Cycle; IR, Immune Response.
