# Supplementary figures and images for: Pan-cancer transcriptomic analysis dissects immune and proliferative functions of APOBEC3 cytidine deaminases
Source: Nucleic Acids Res. 2019 Jan 9;47(3):1178–94. doi: 10.1093/nar/gky1316 (PMC6379723; doi:10.1093/nar/gky1316)

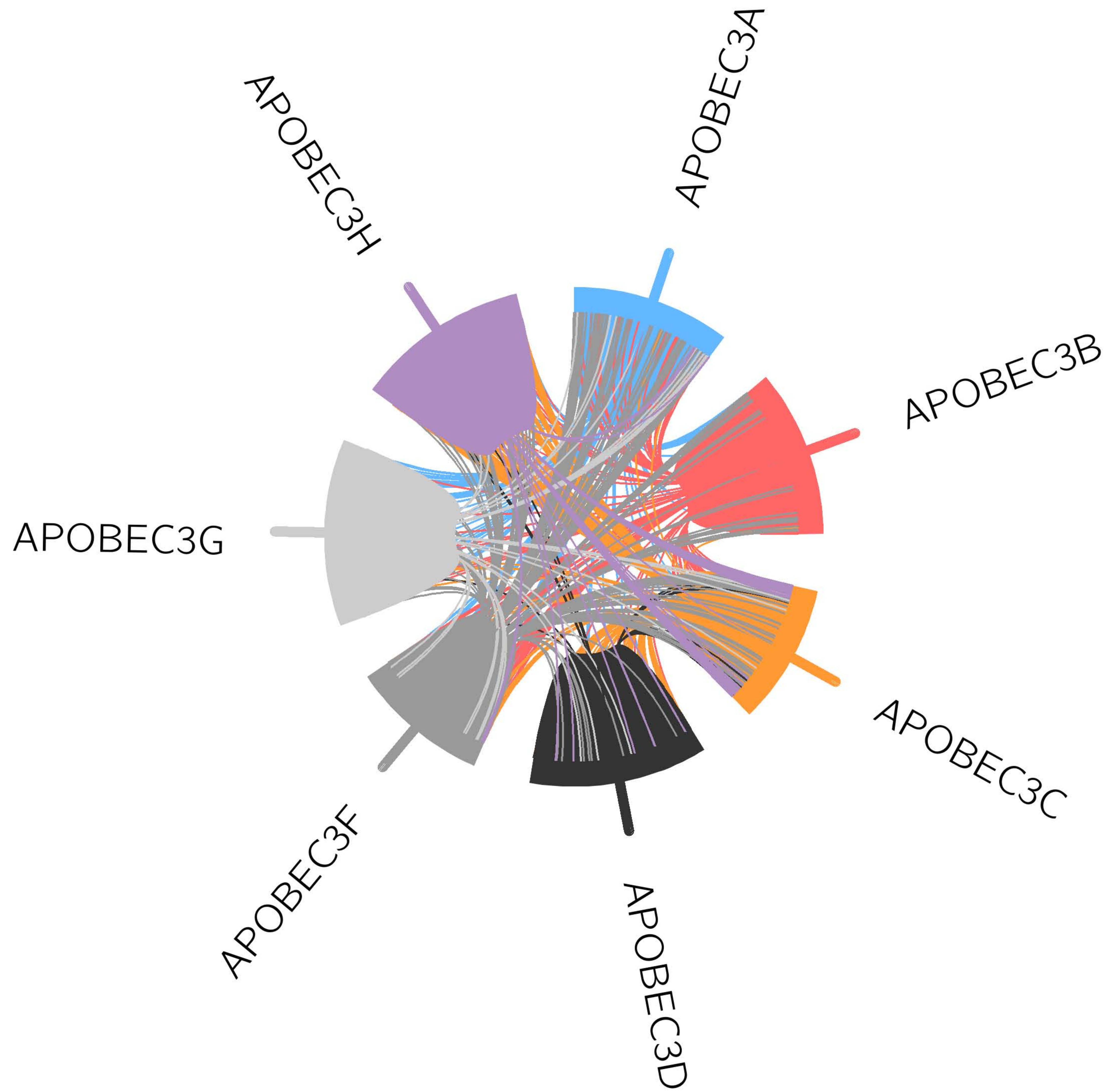

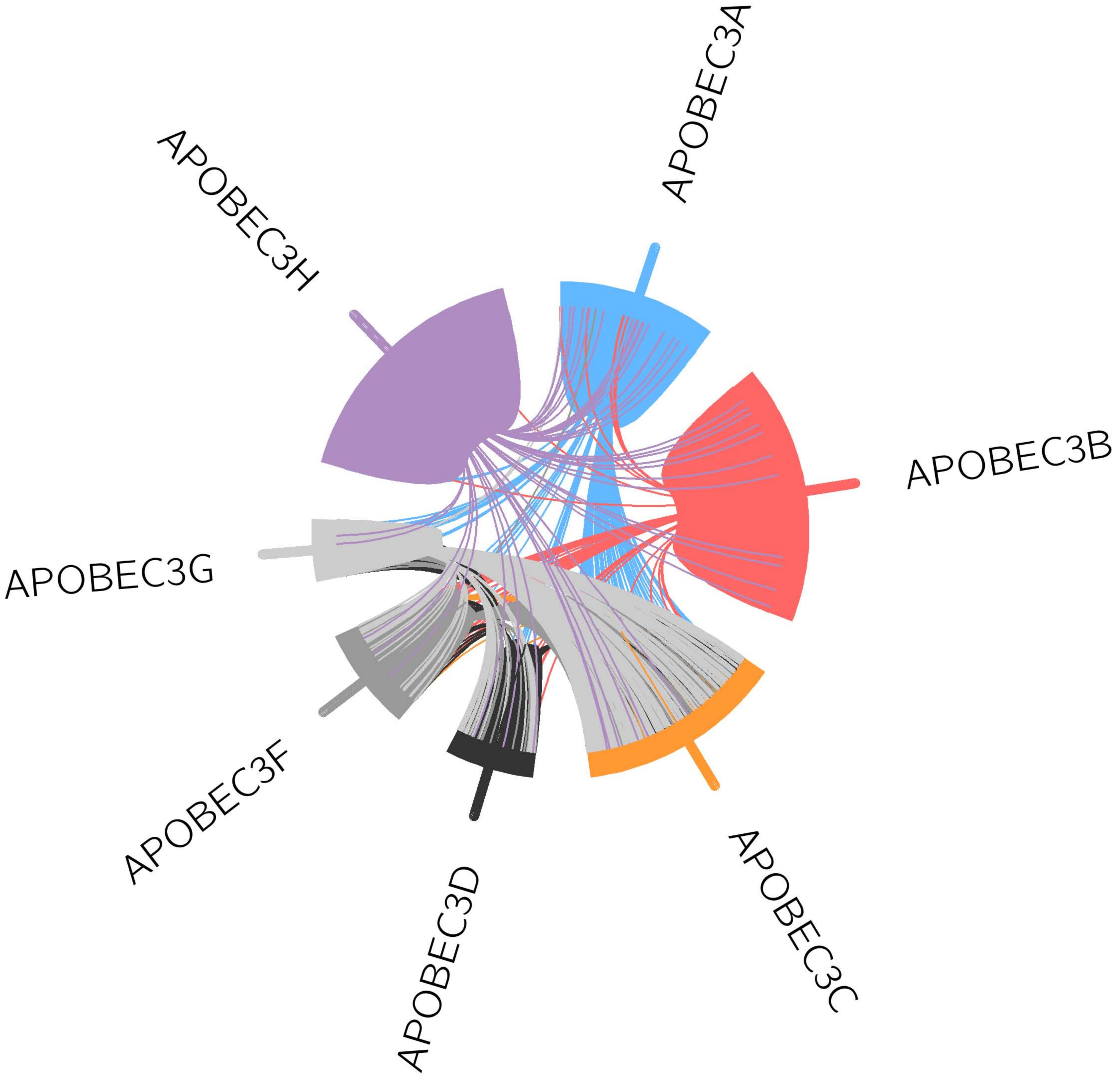

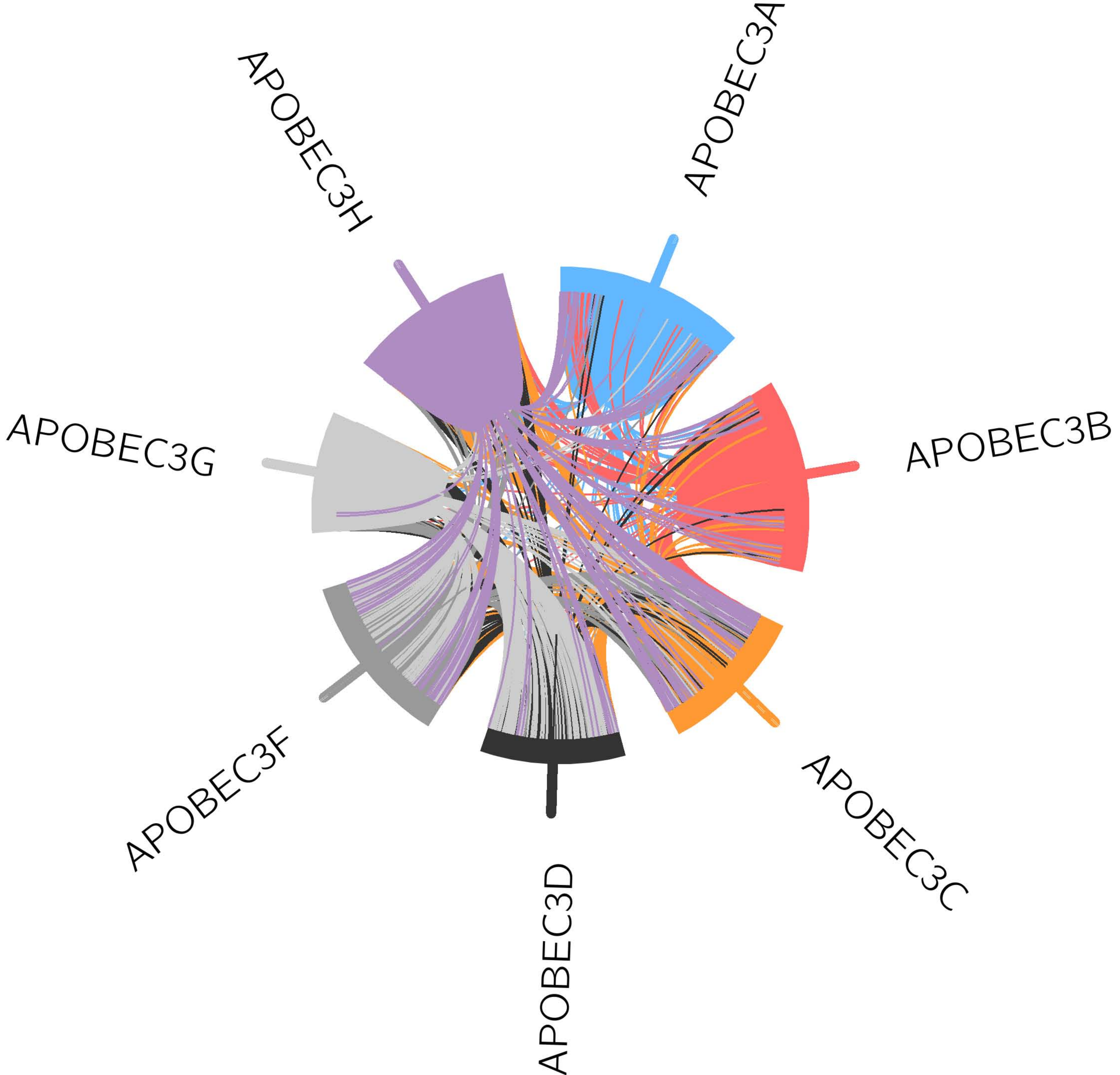

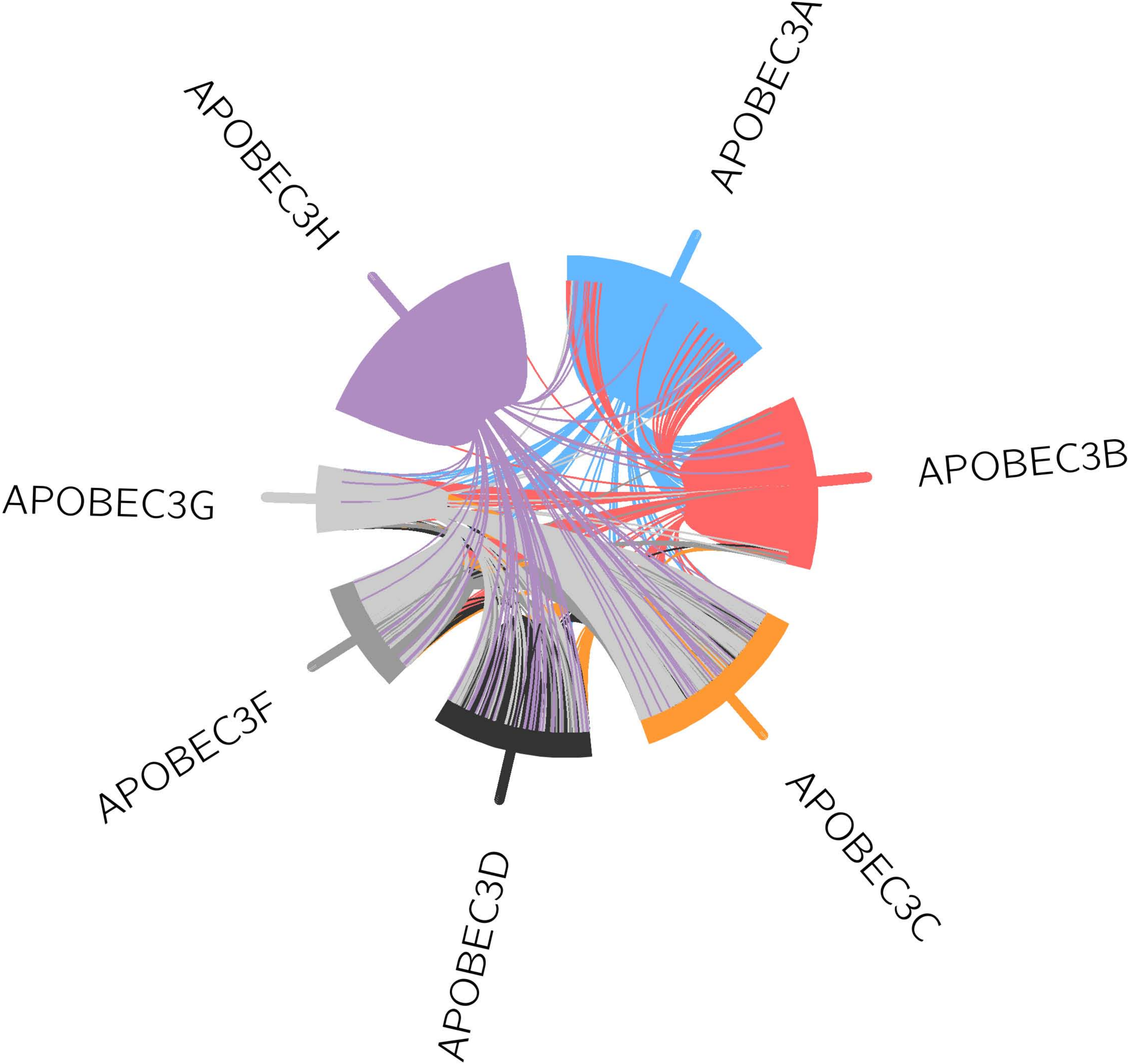

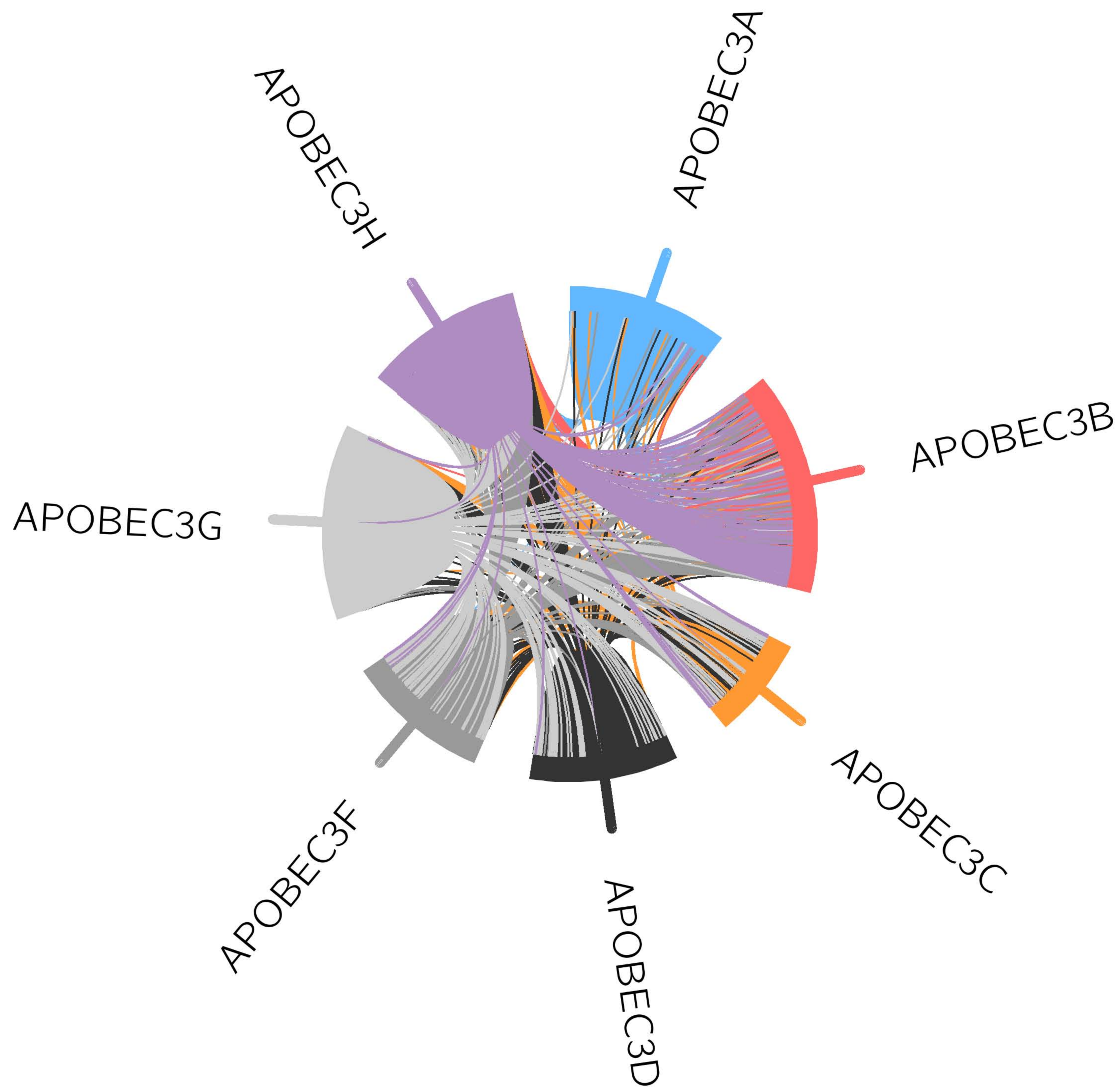

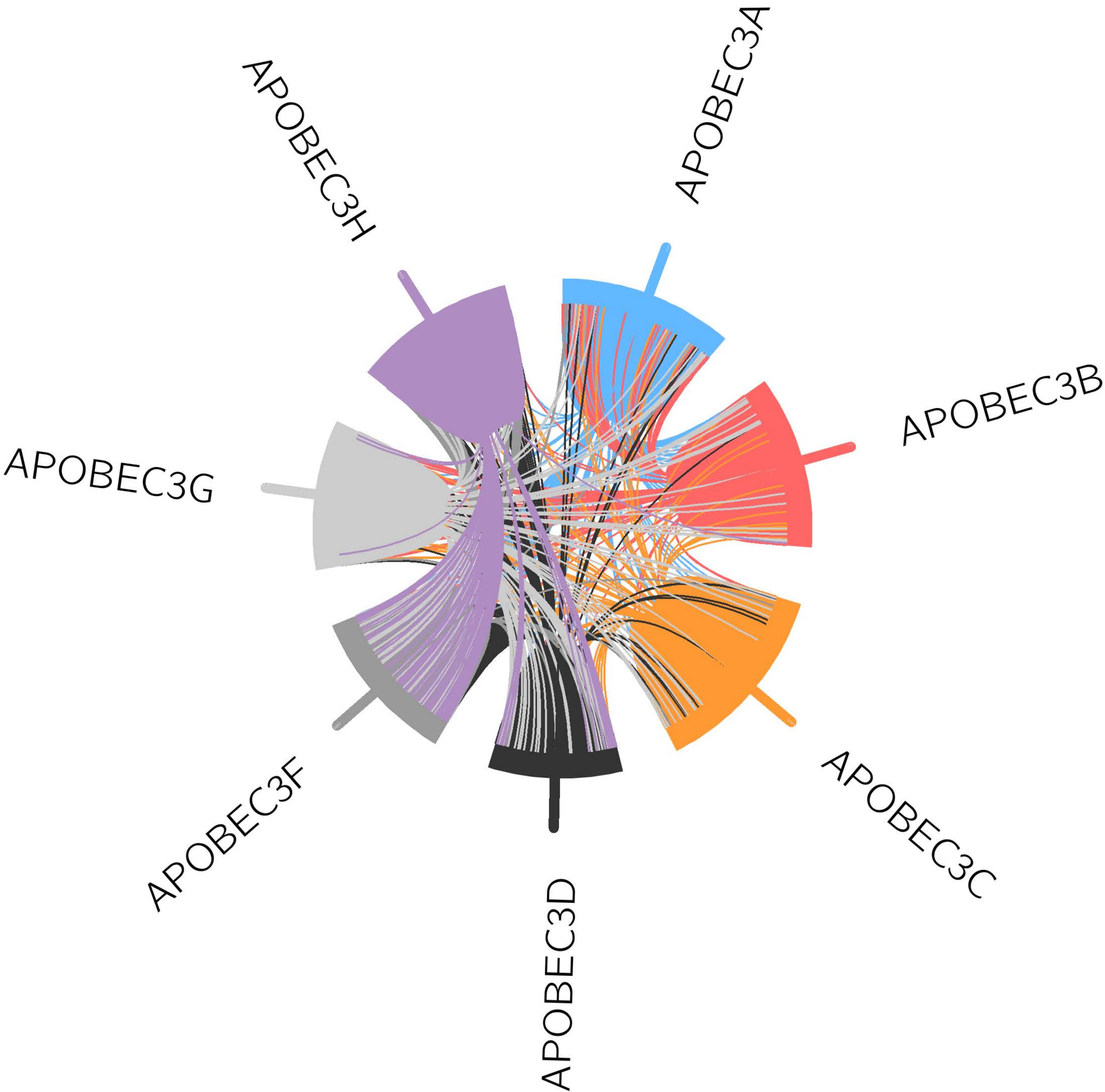

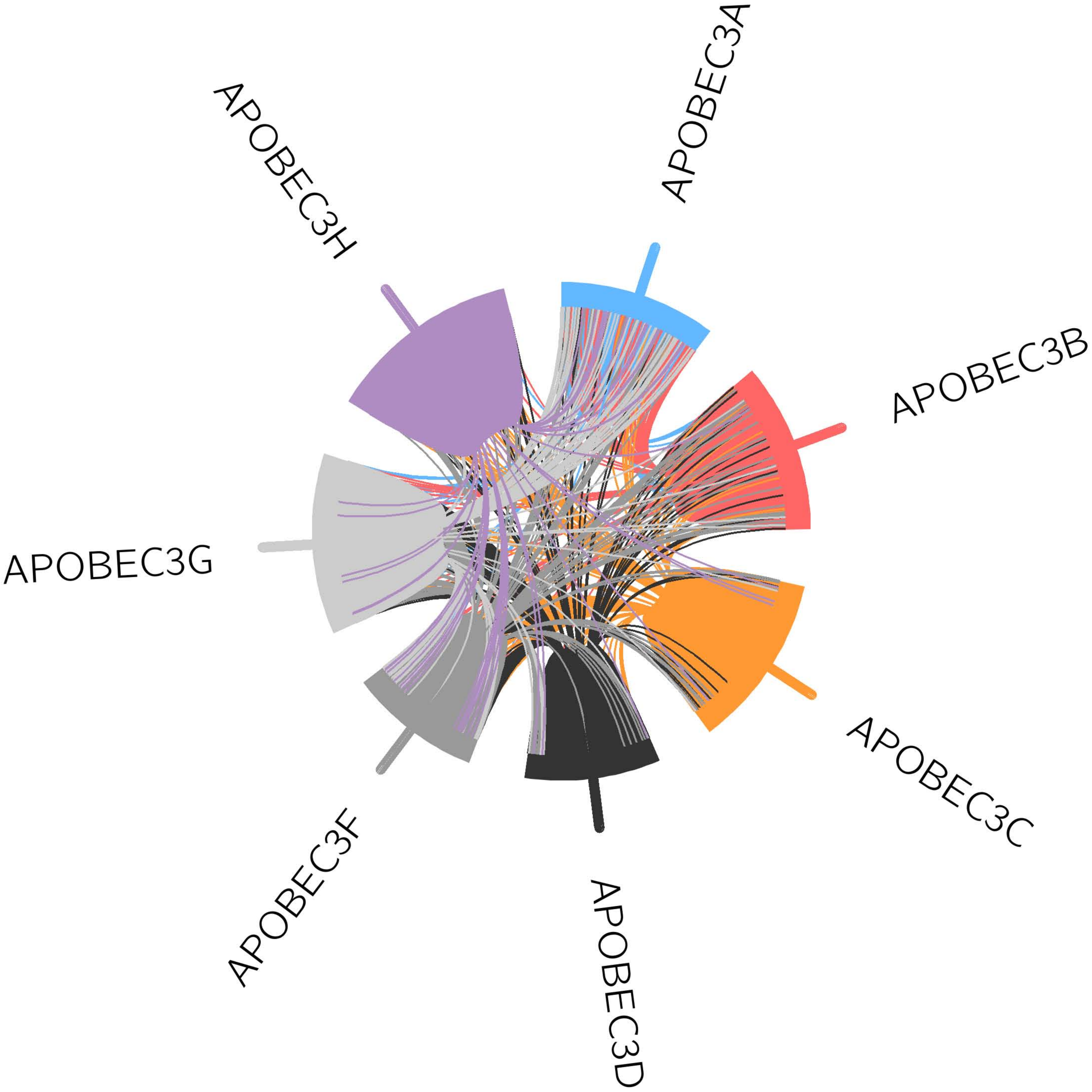

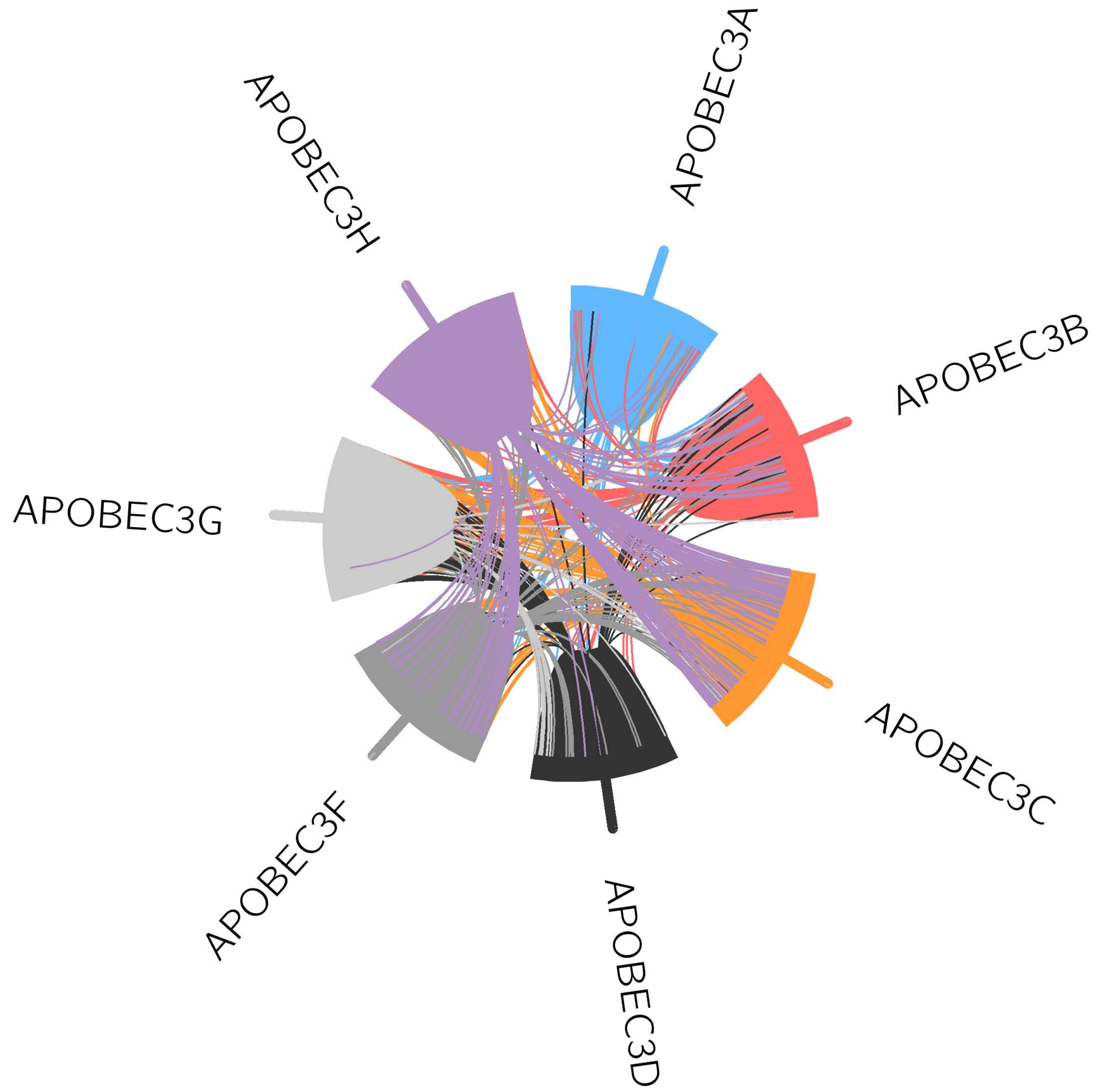

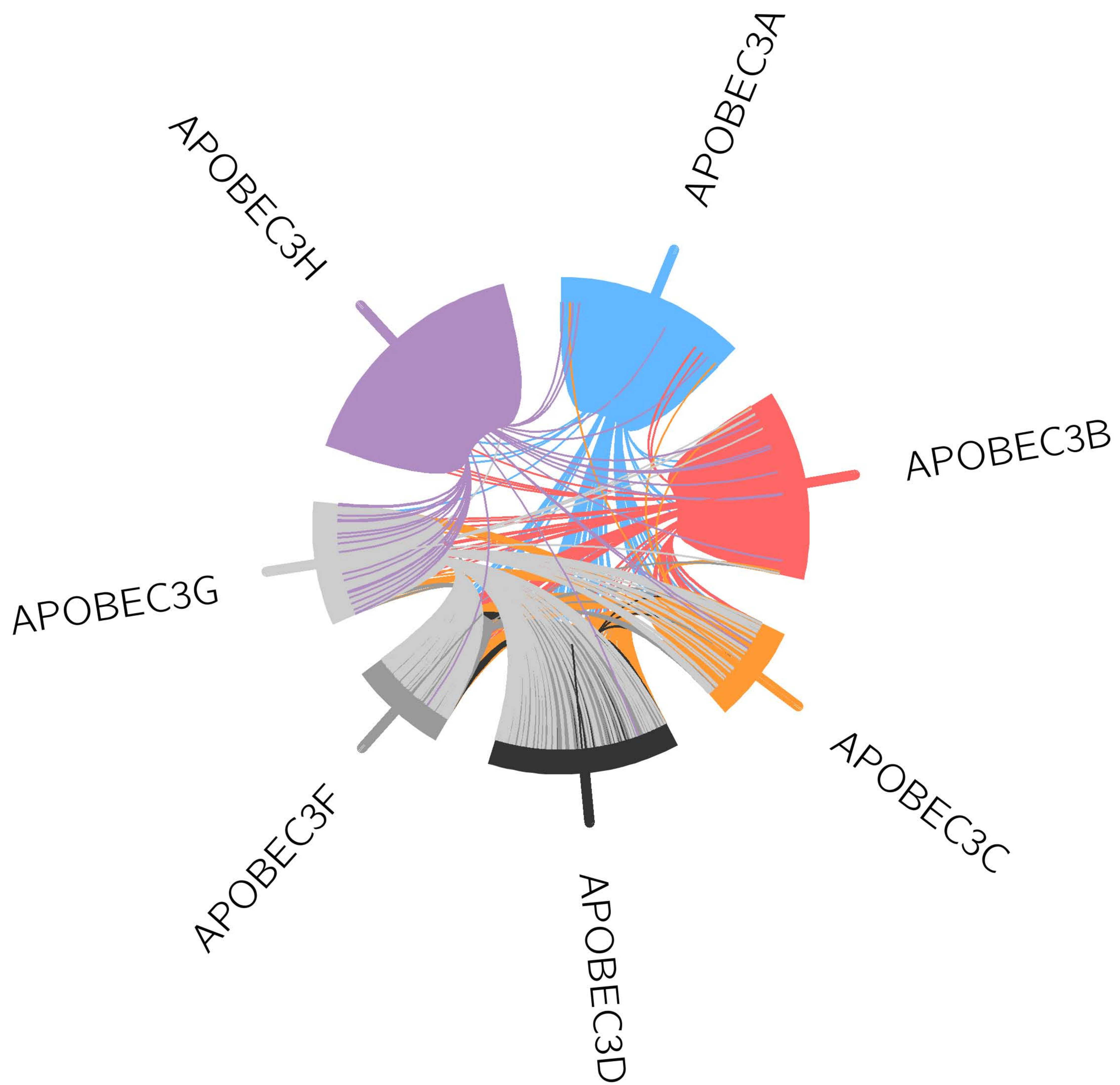

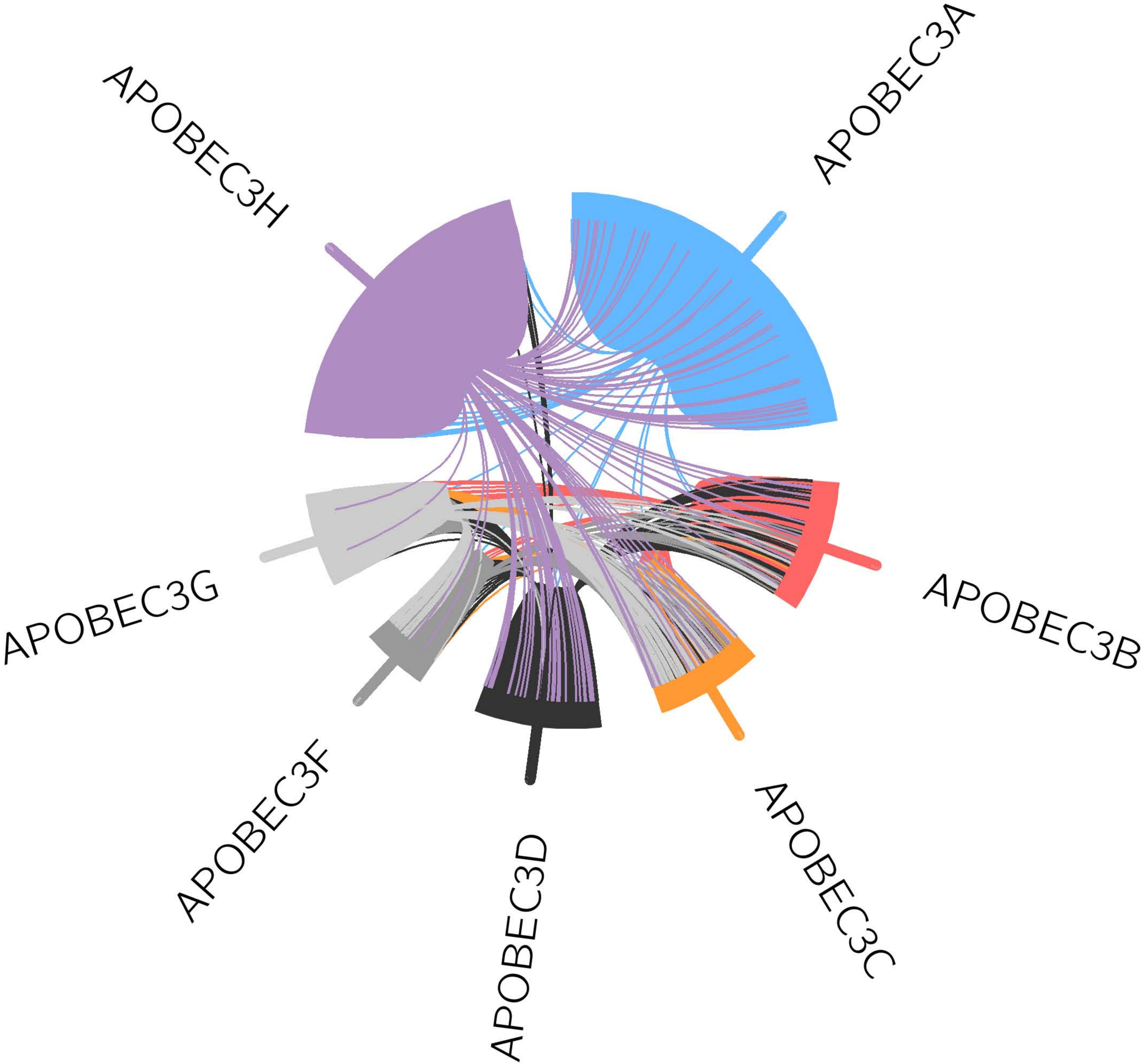

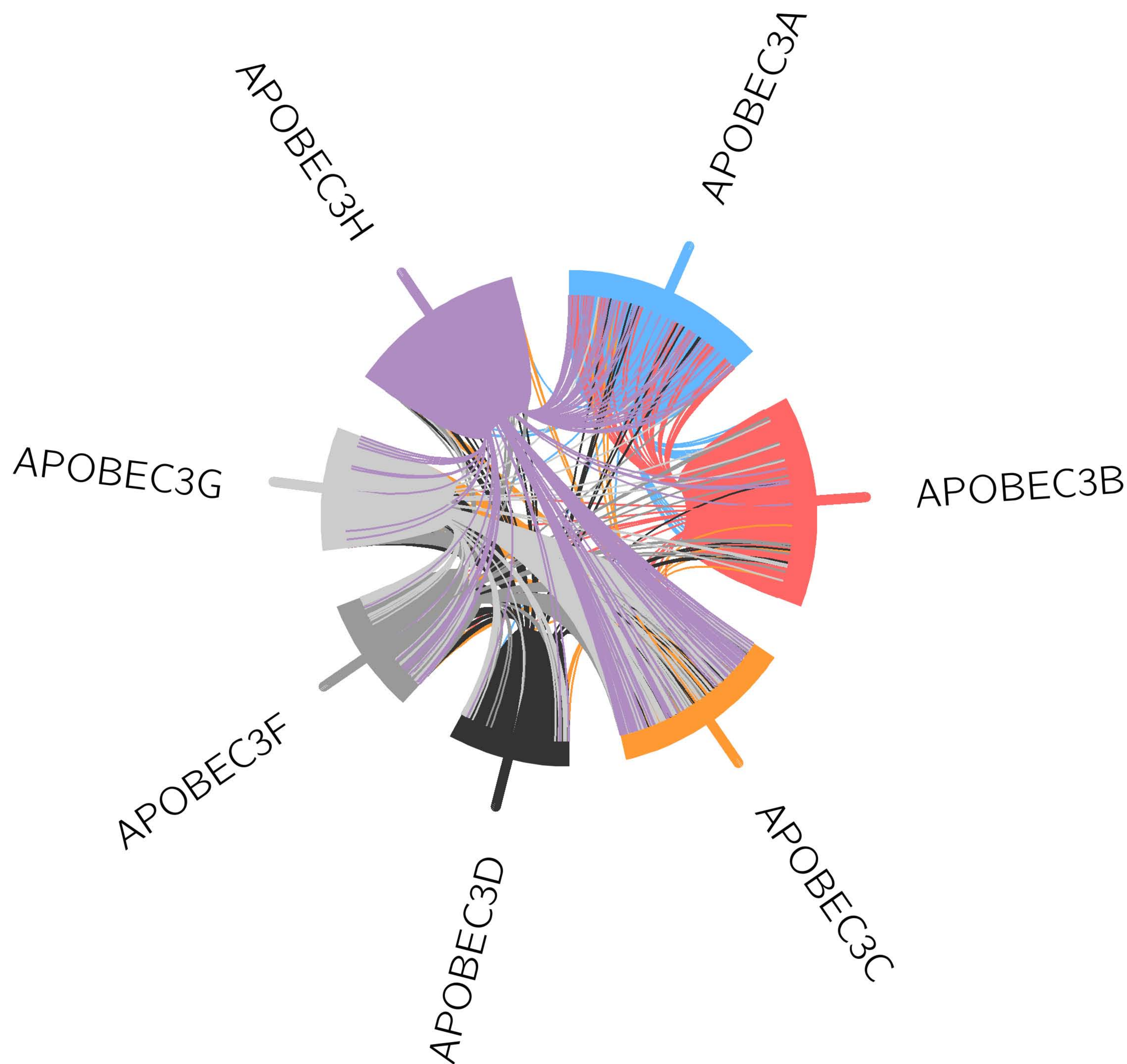

CCLE.LUAD

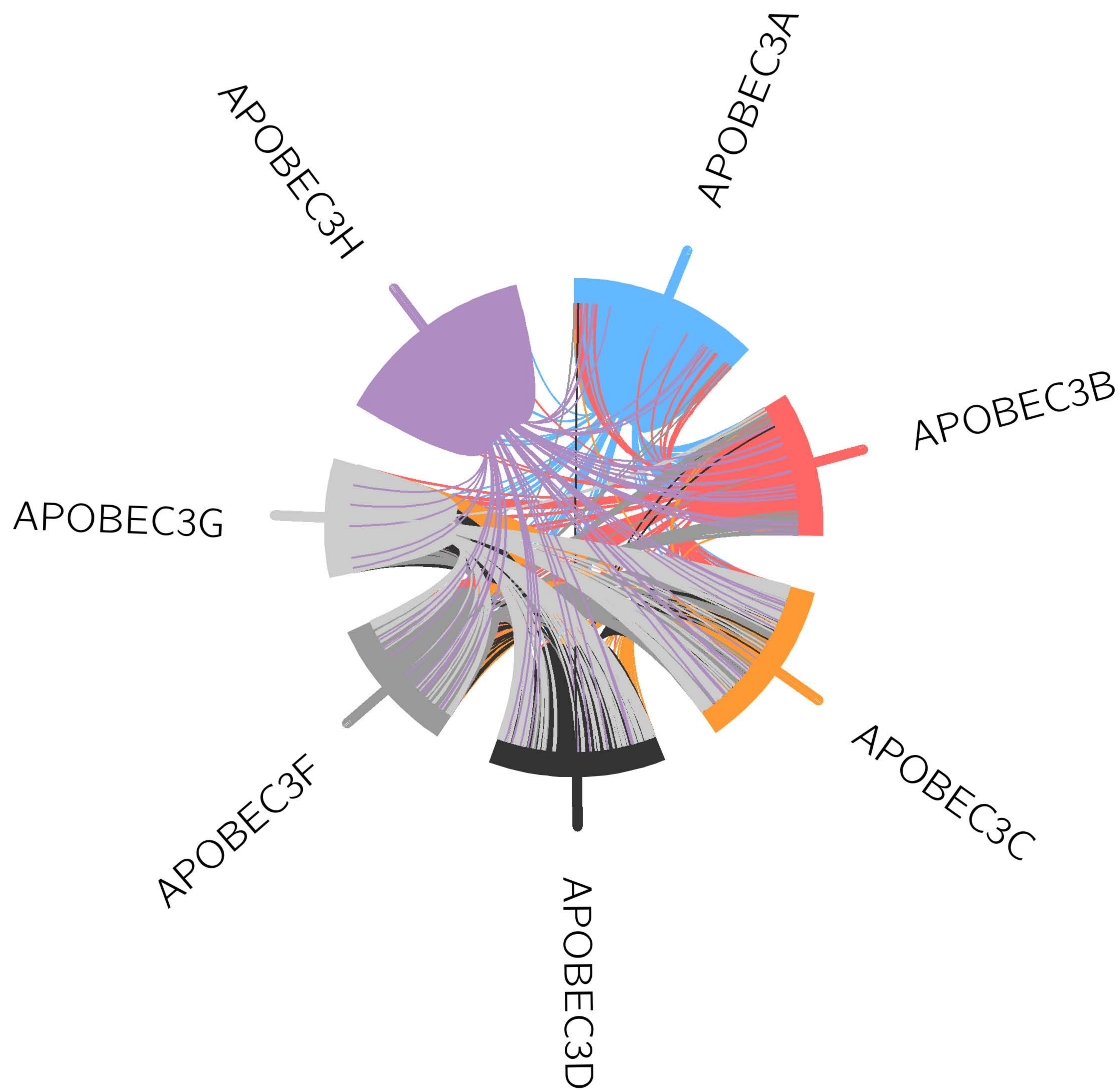

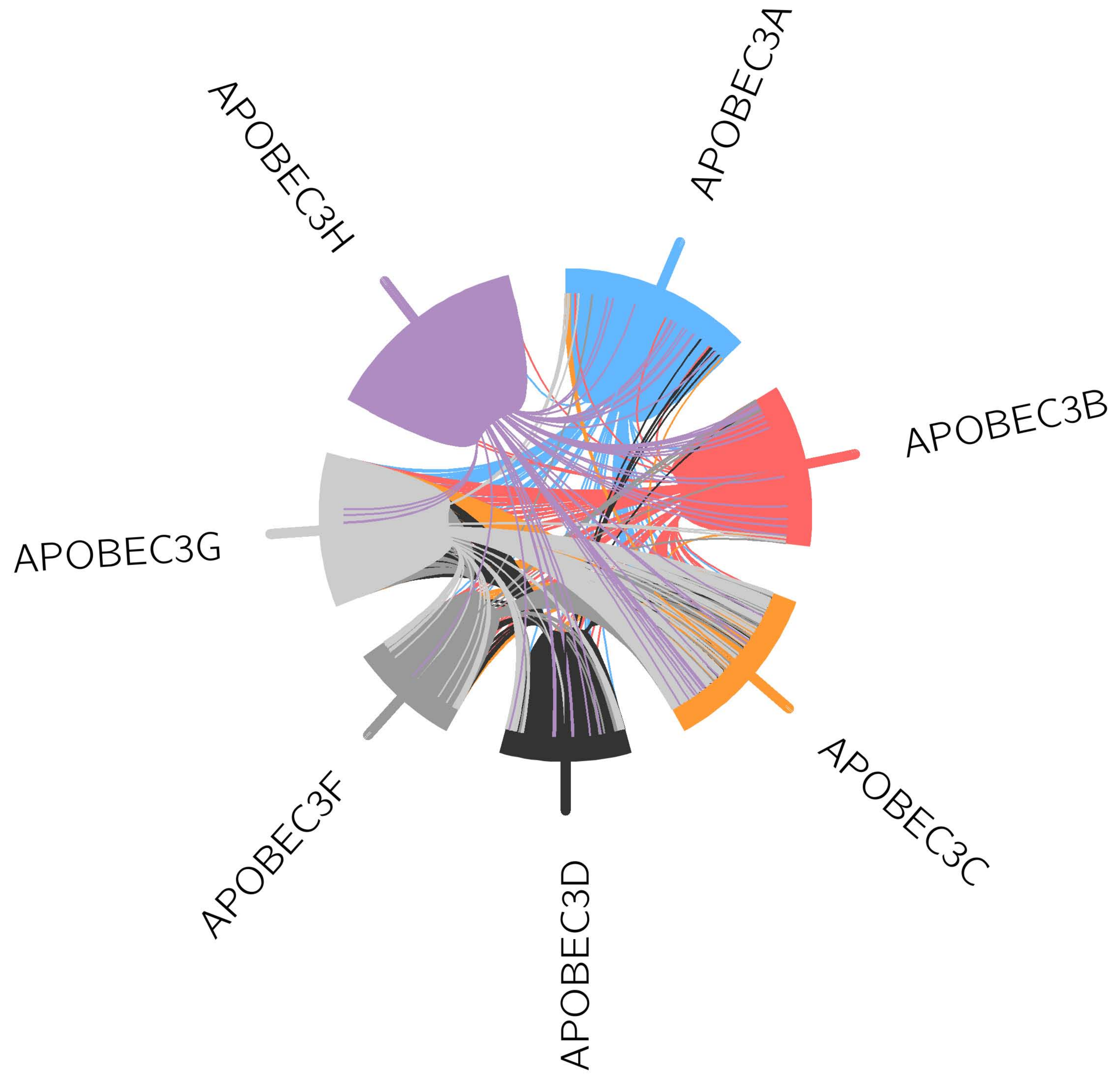

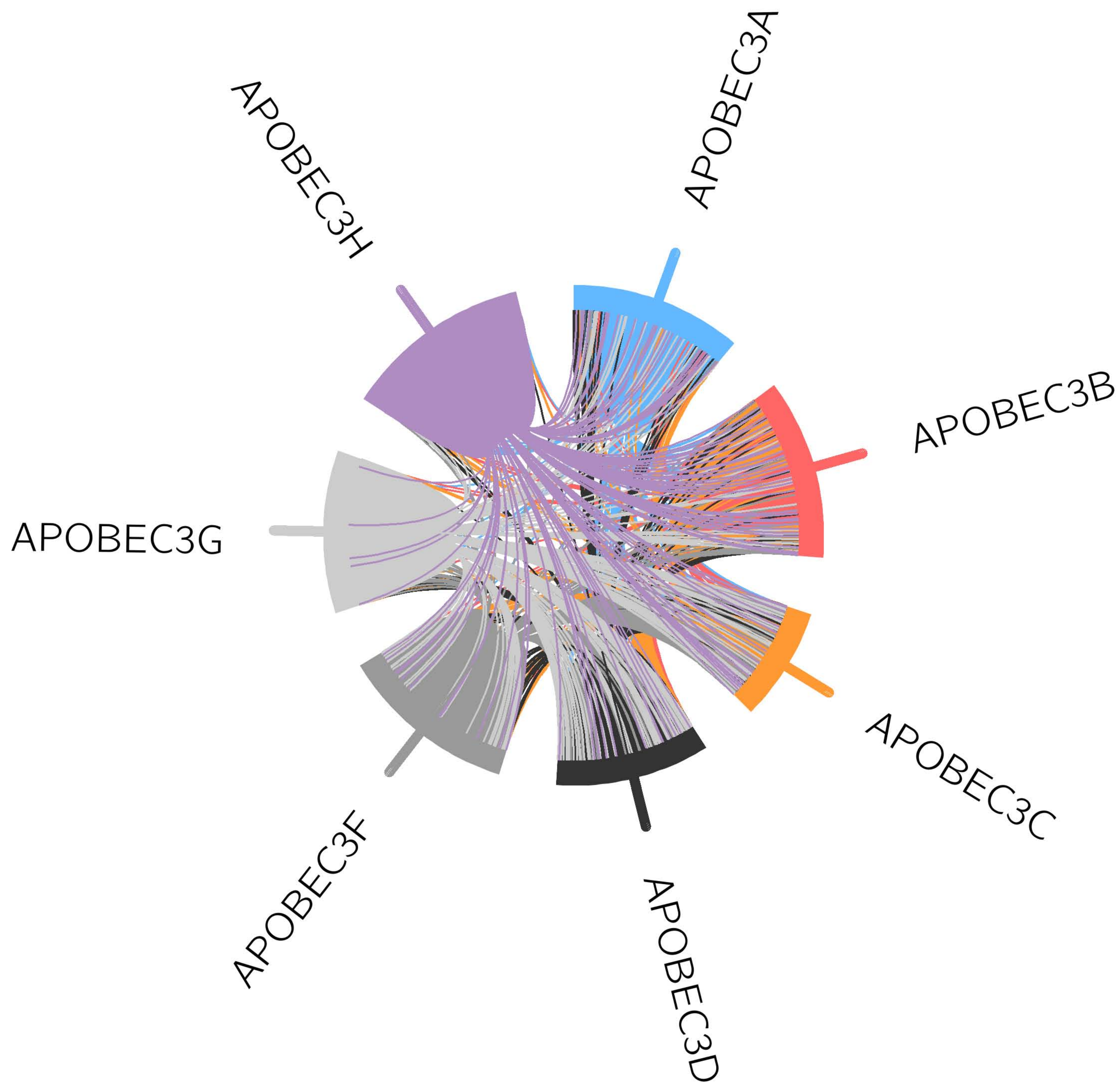

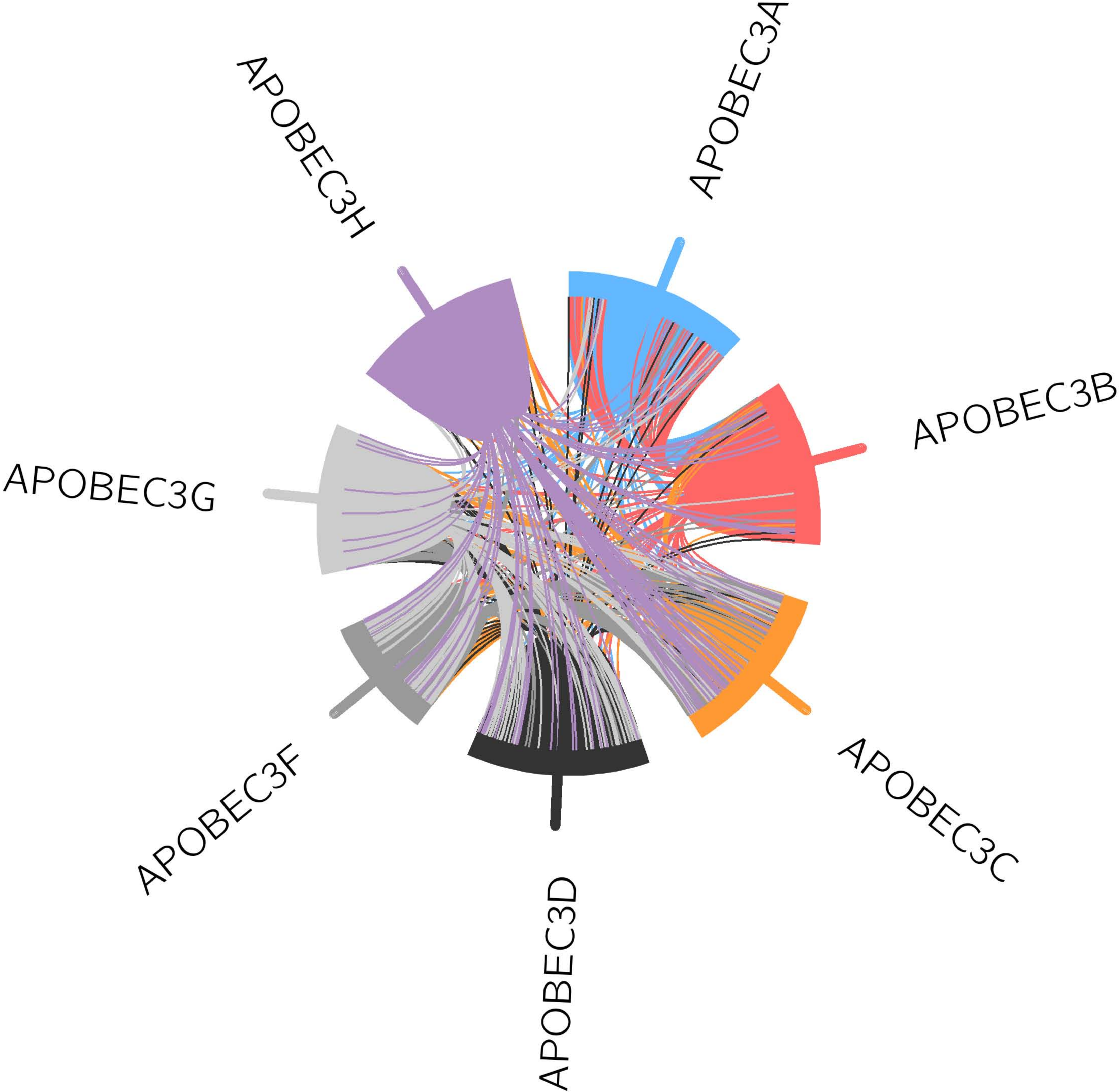

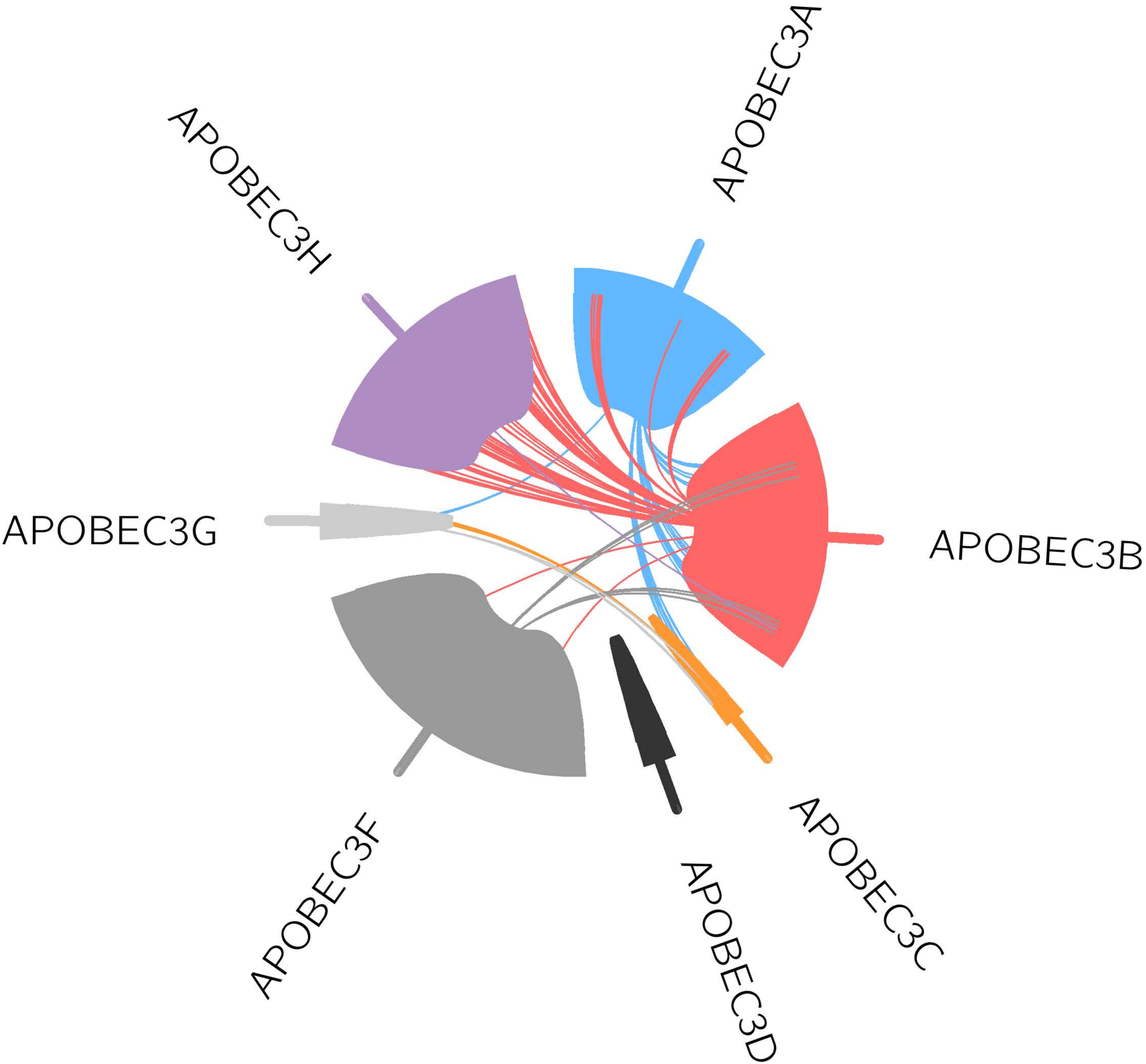

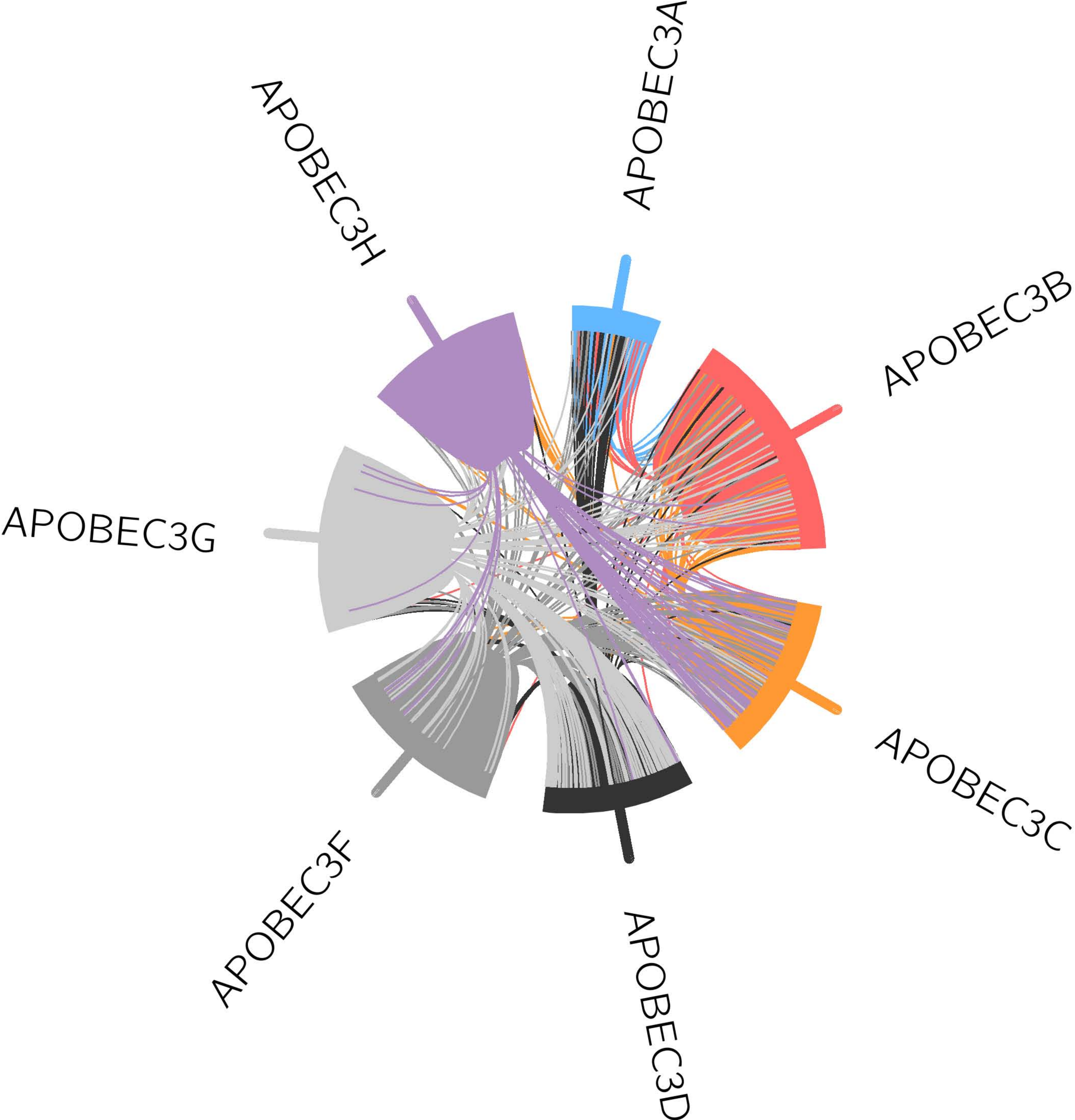

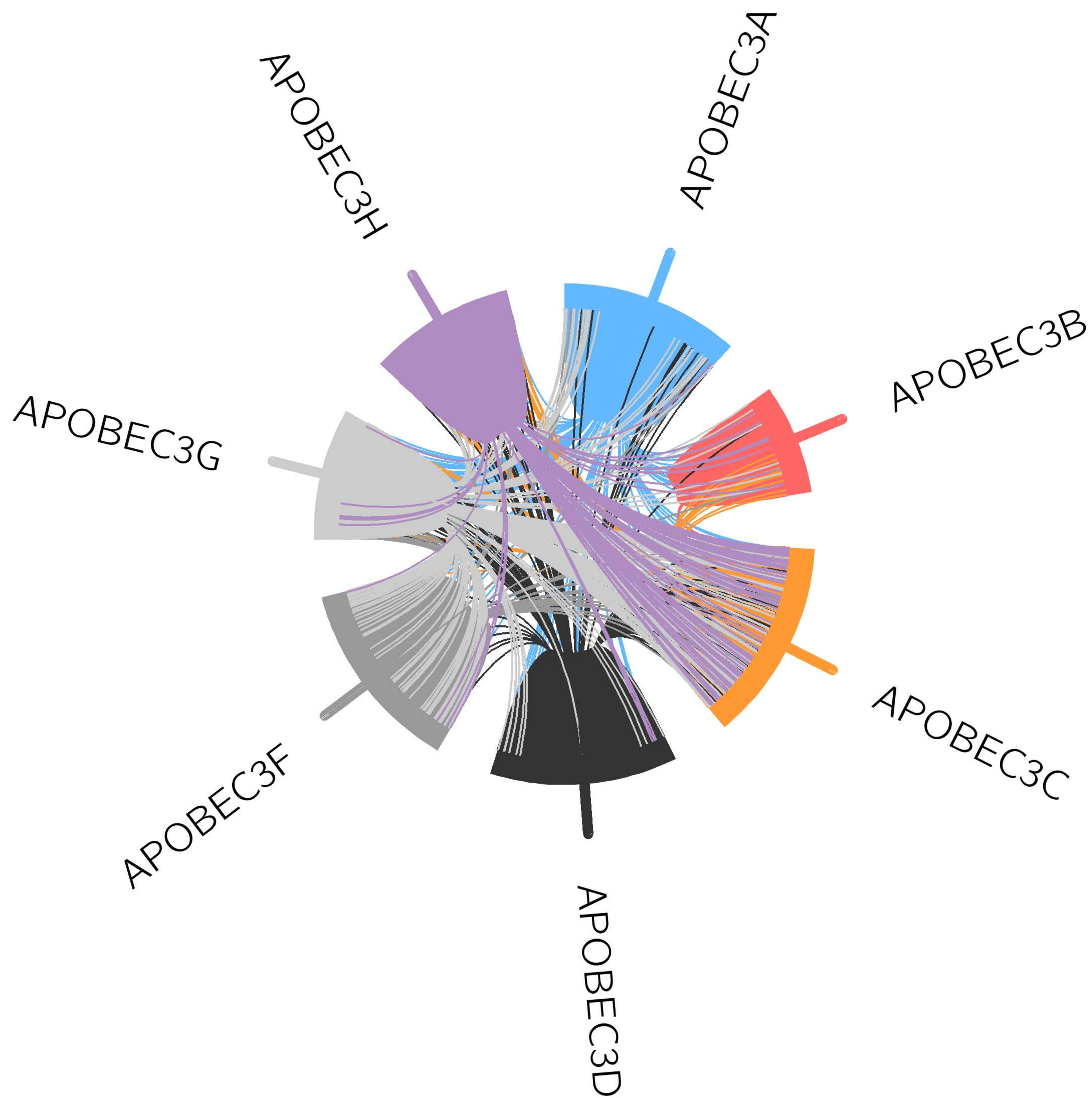

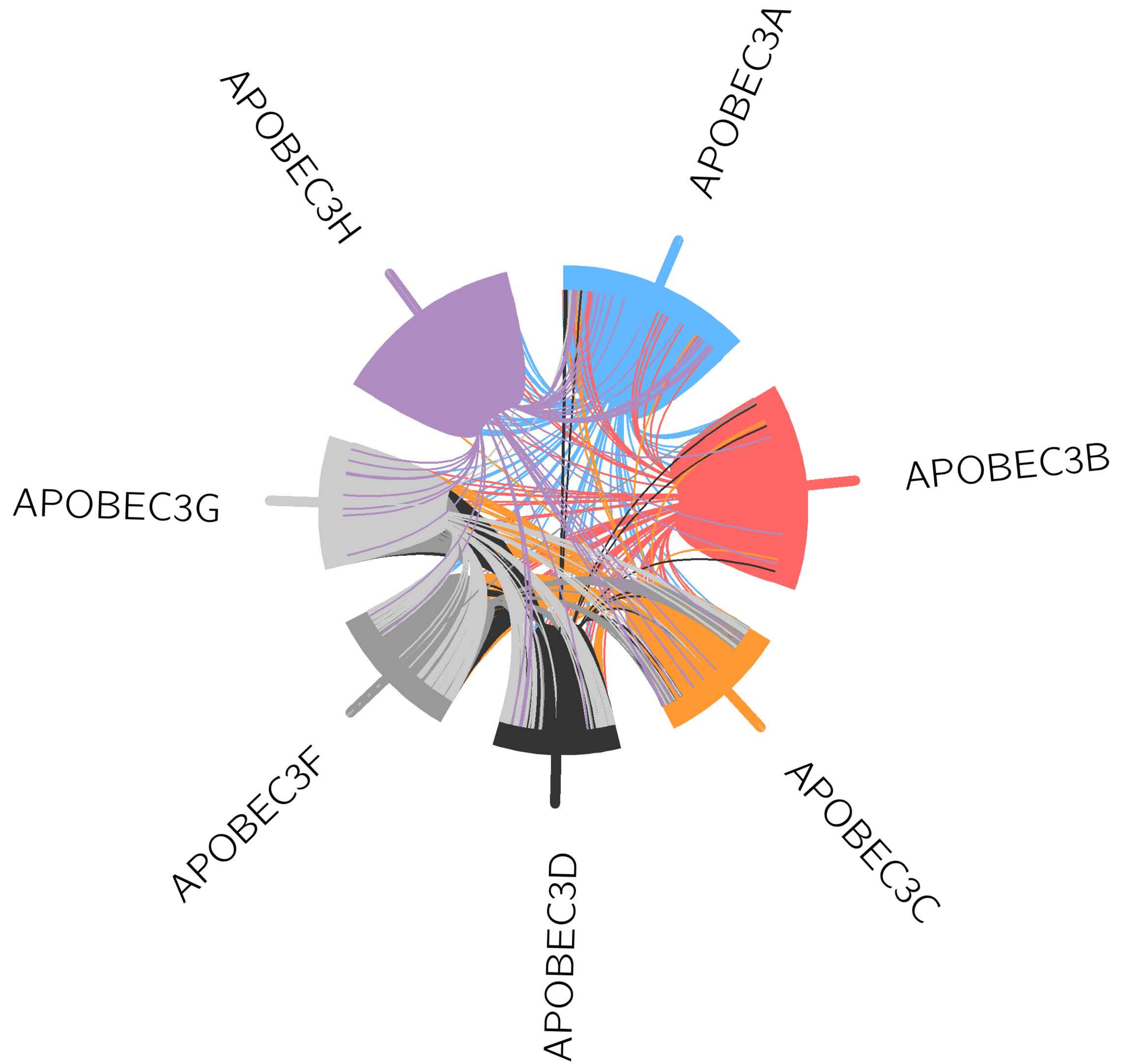

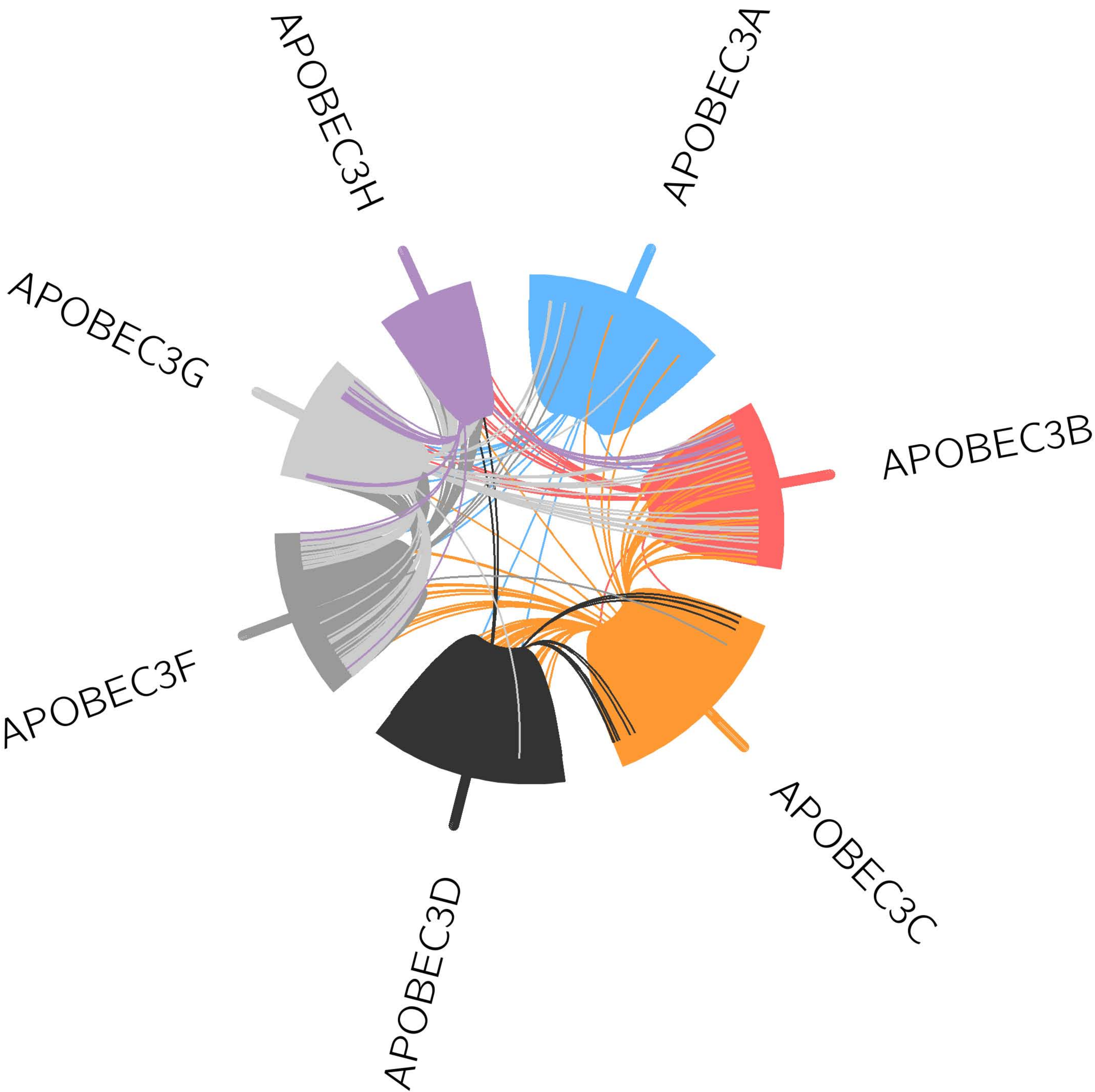

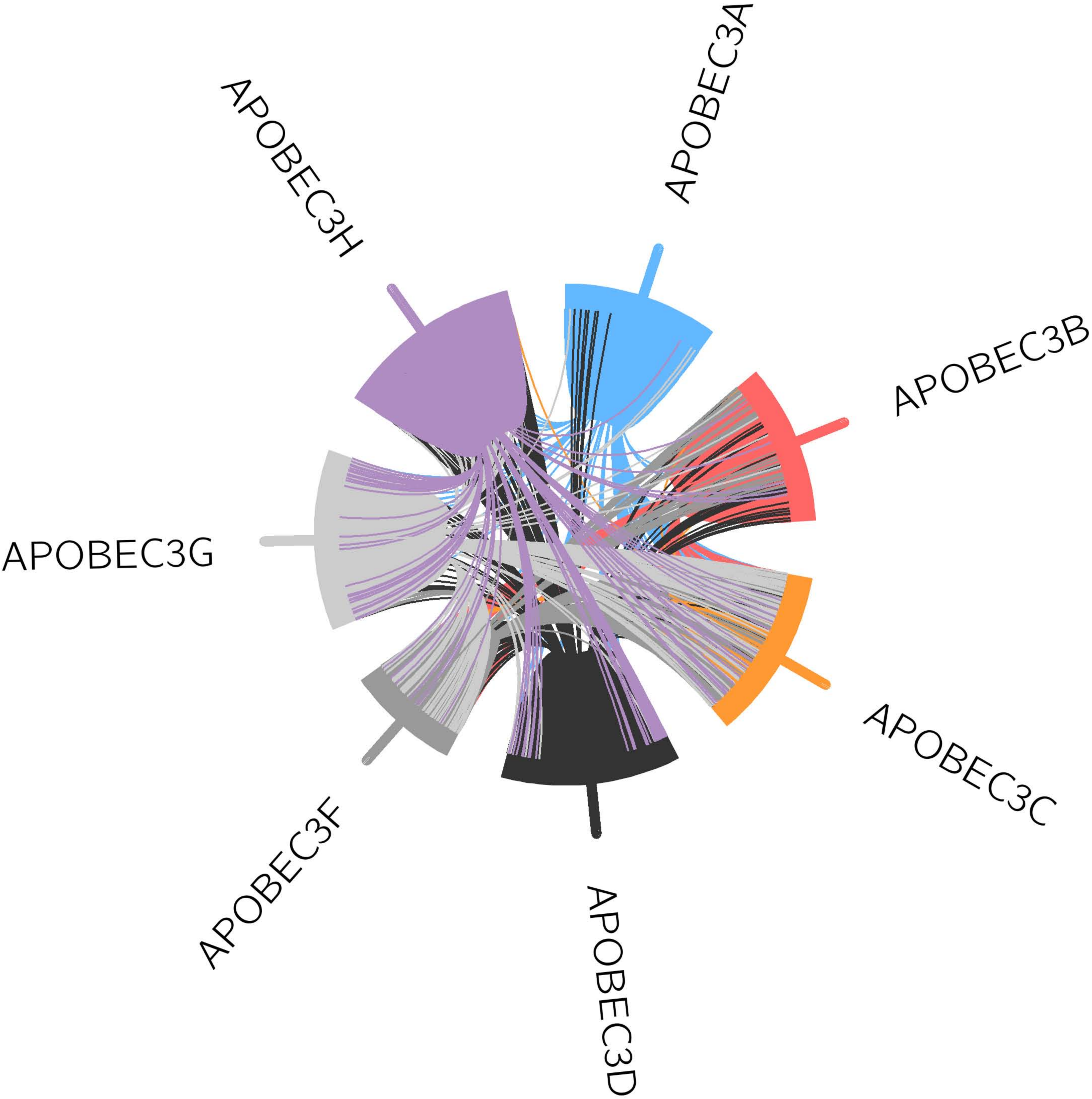

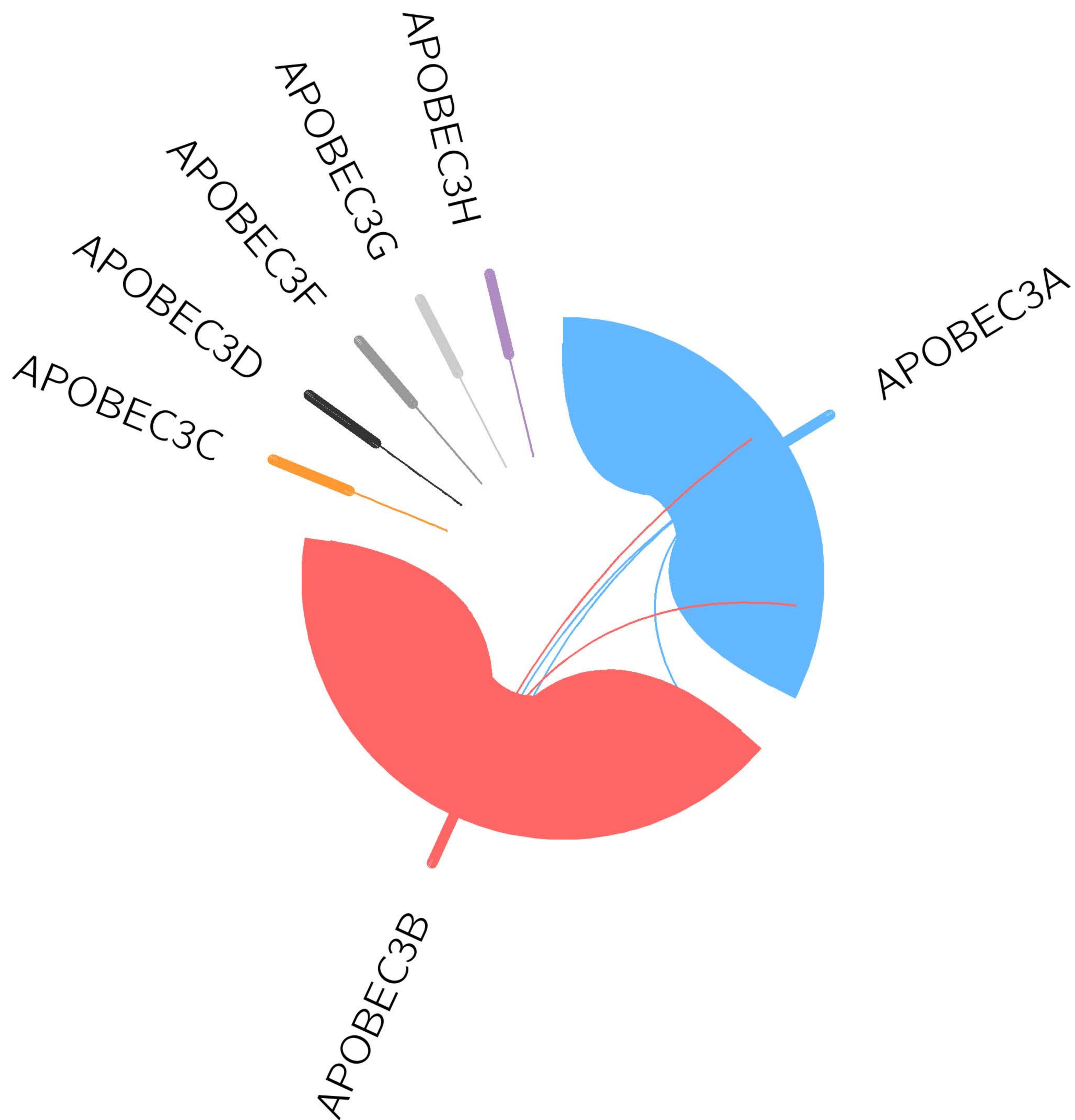

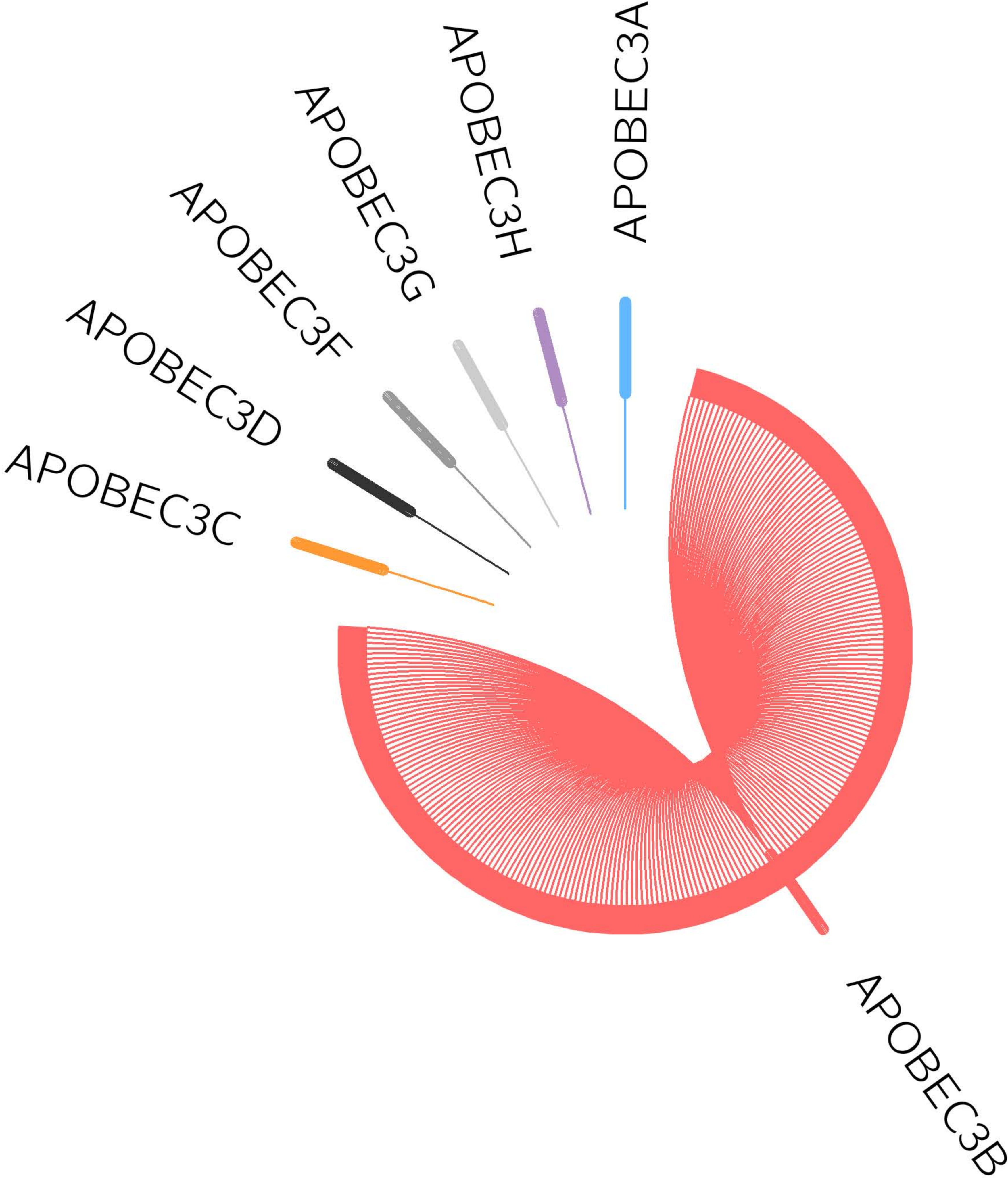

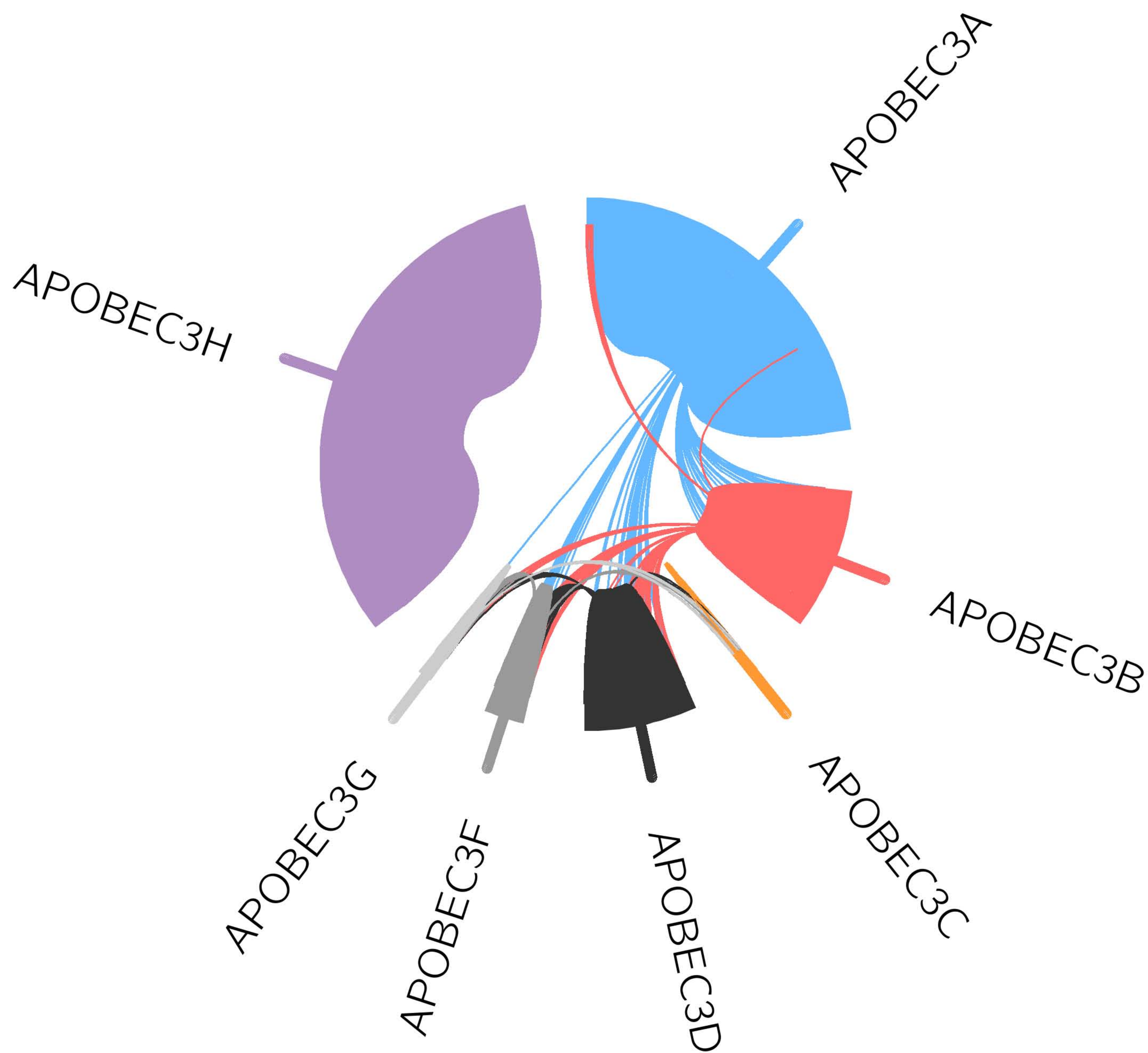

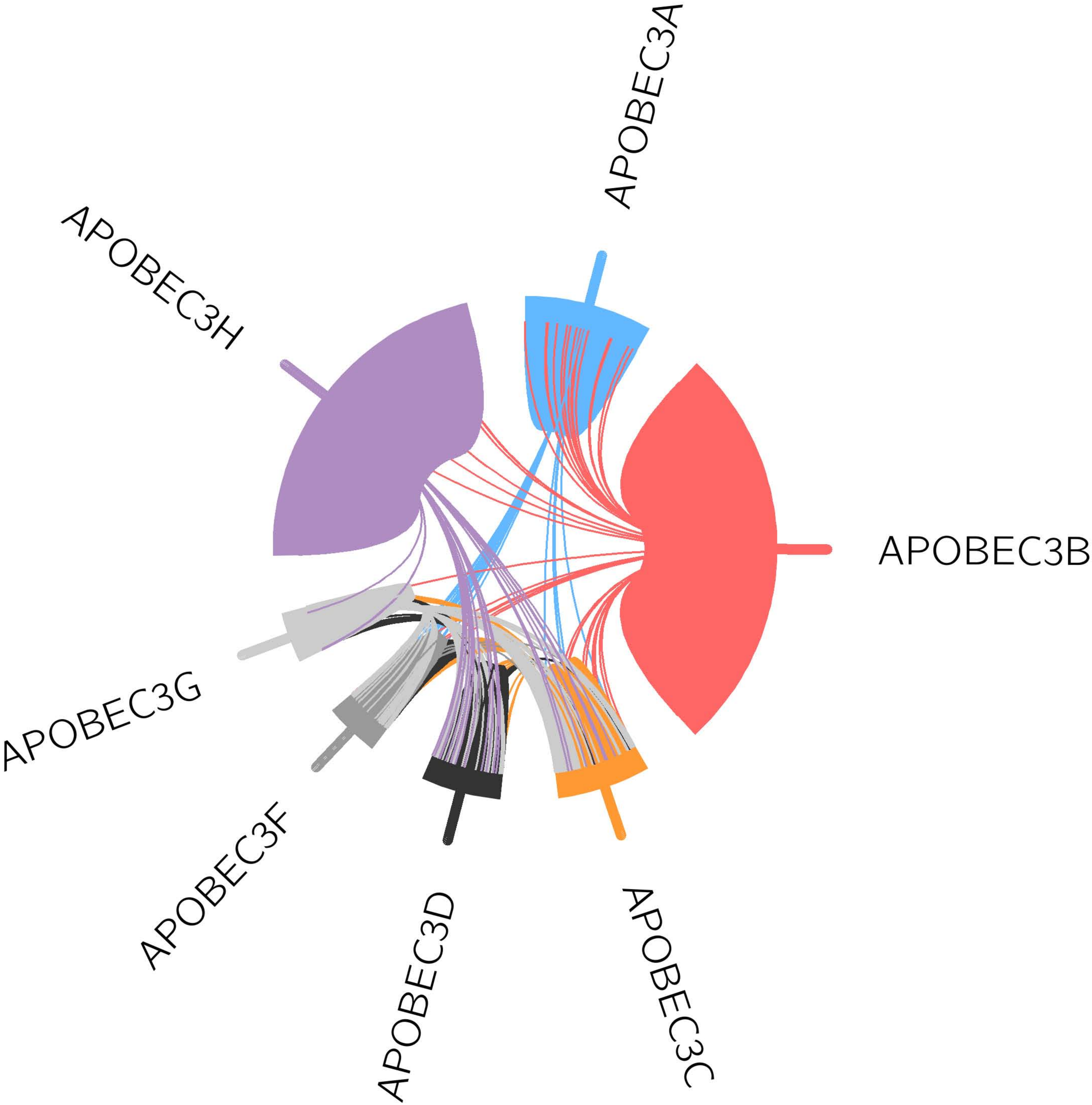

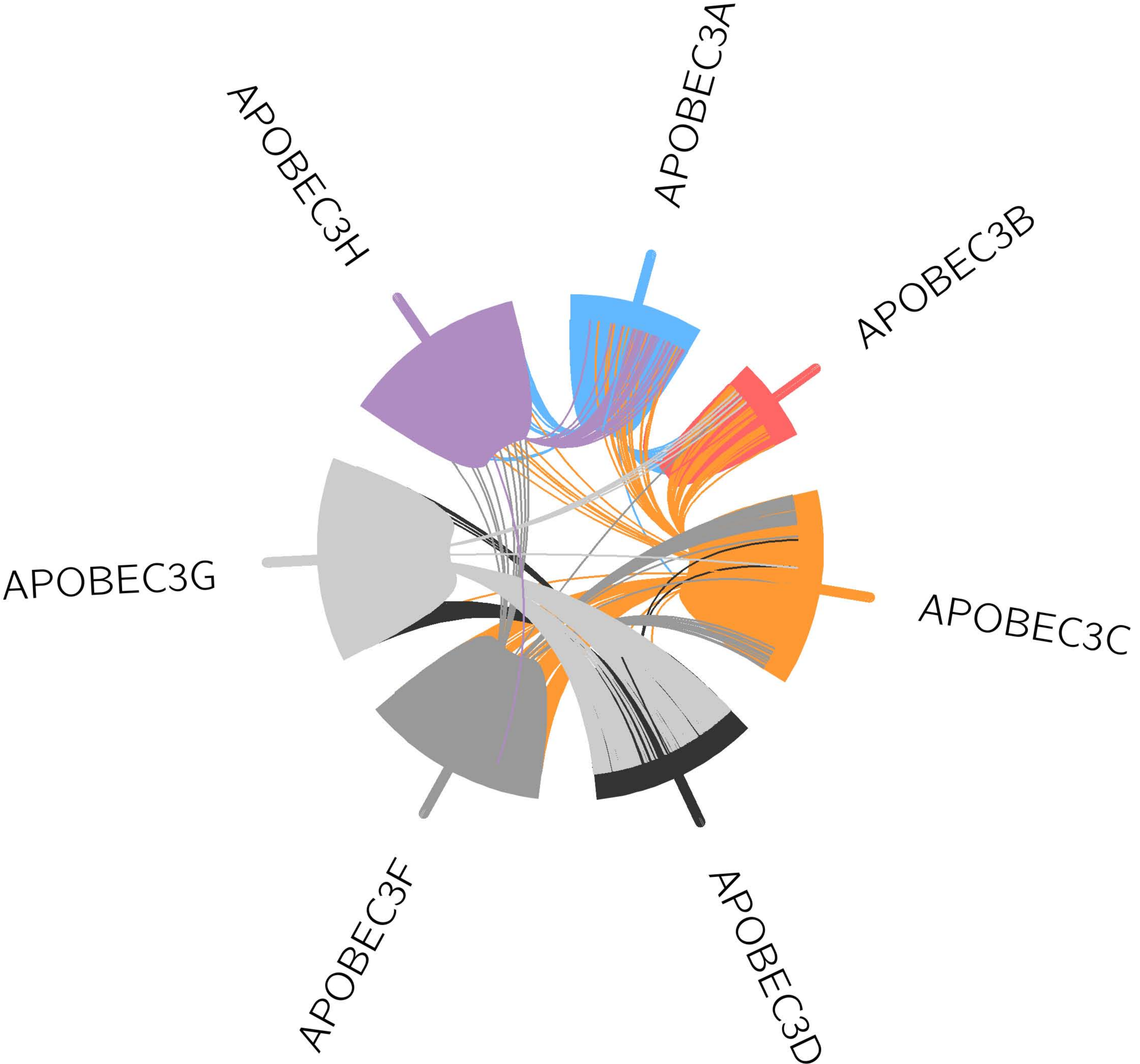

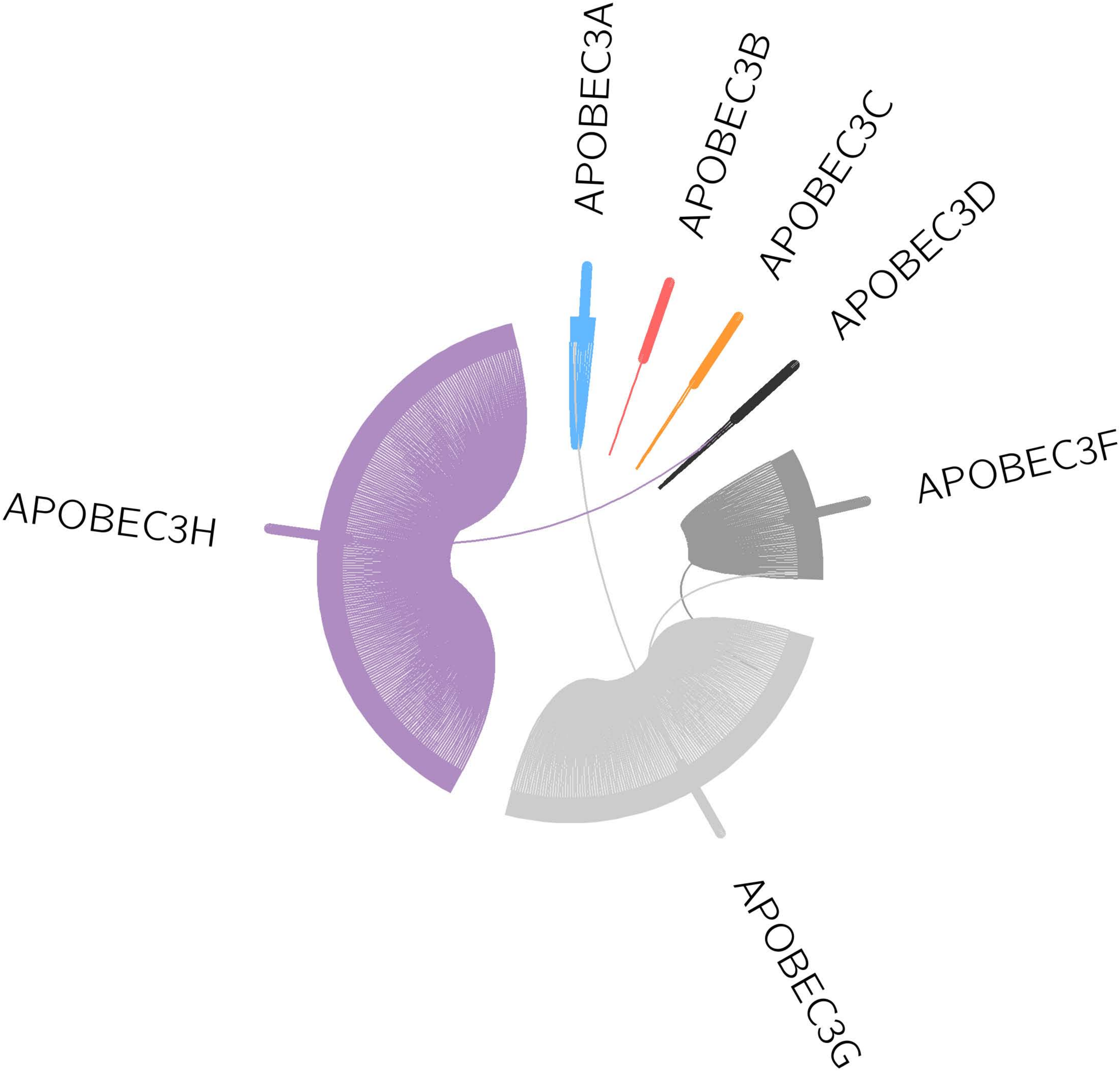

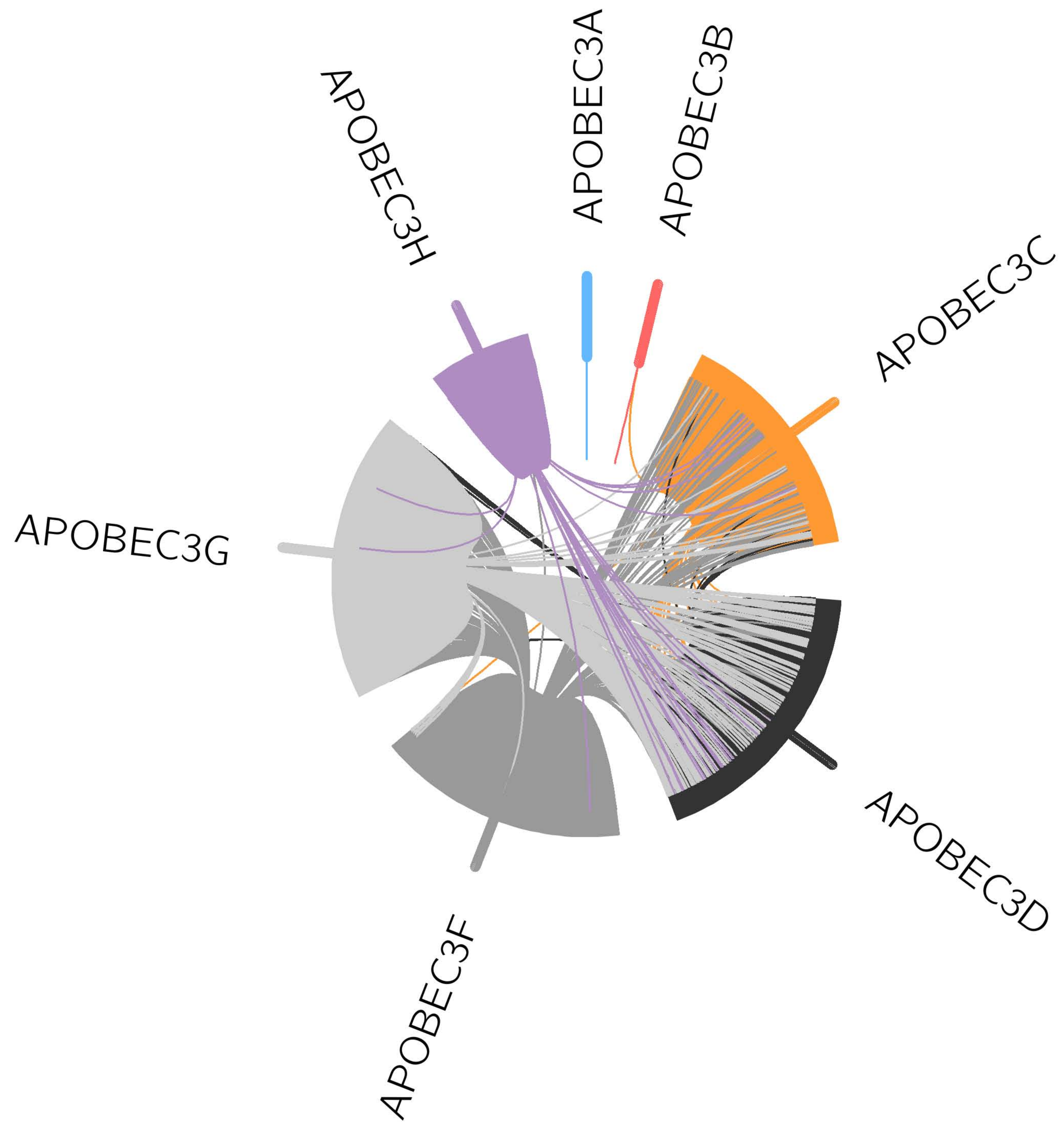

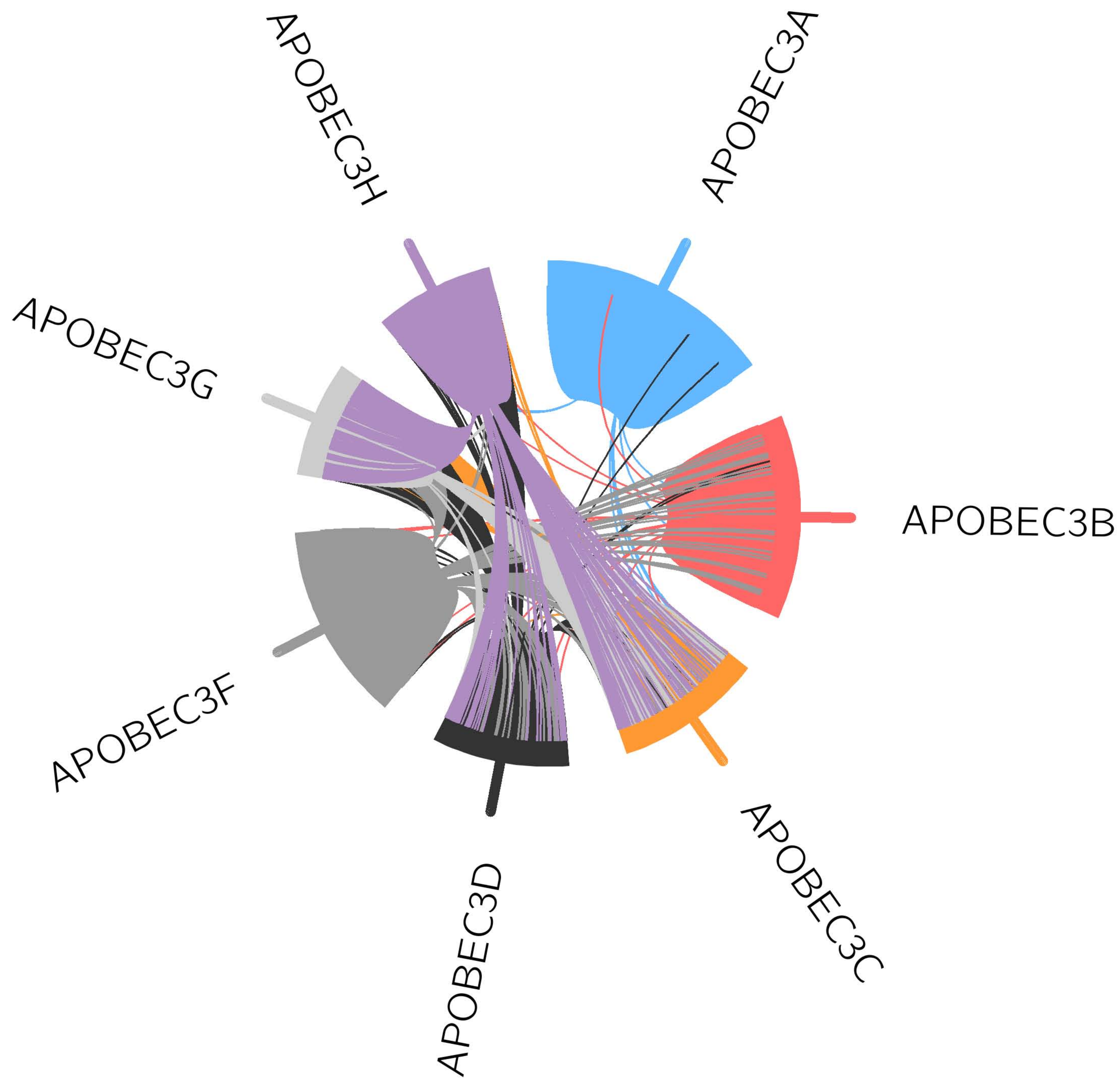

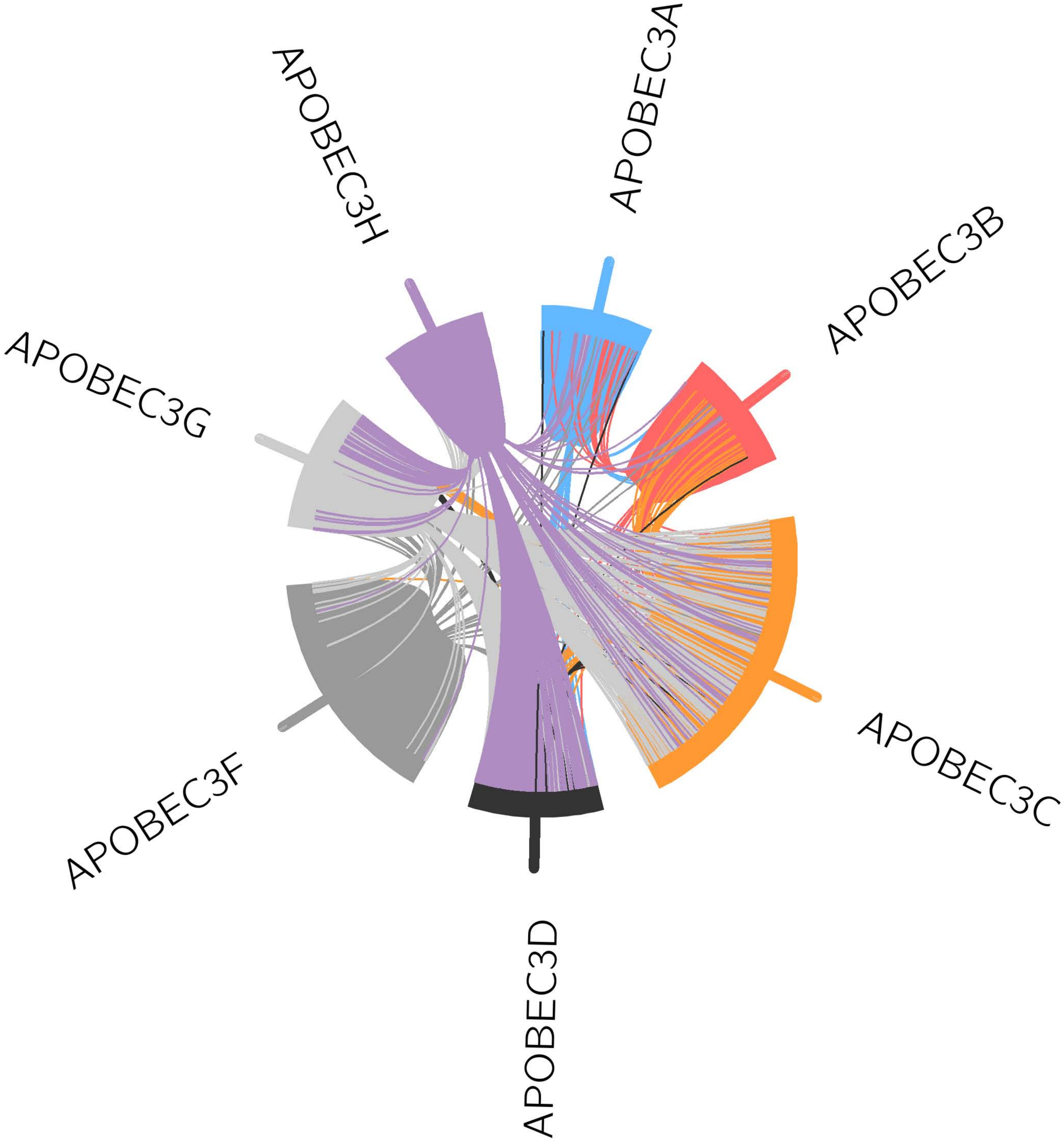

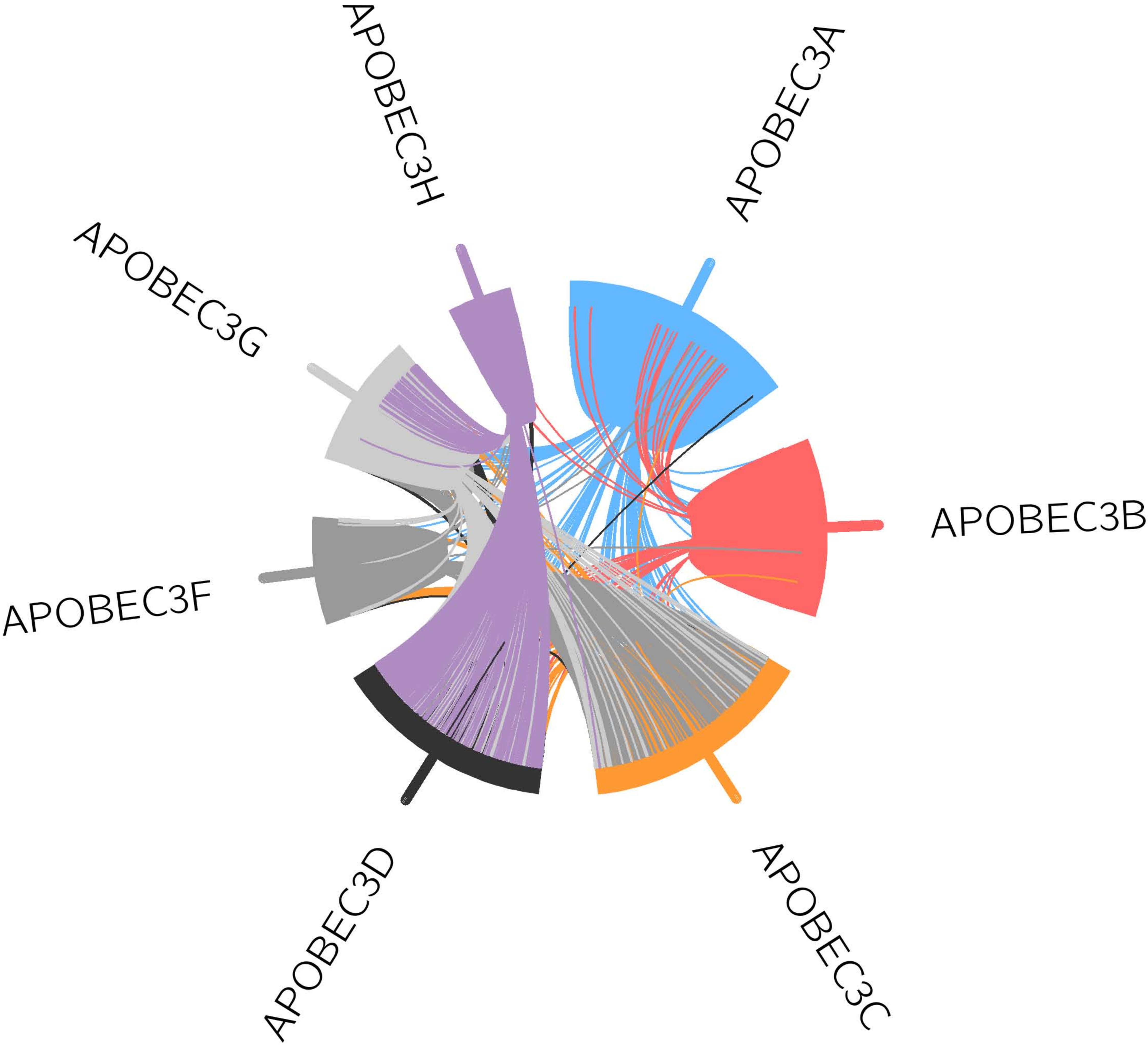

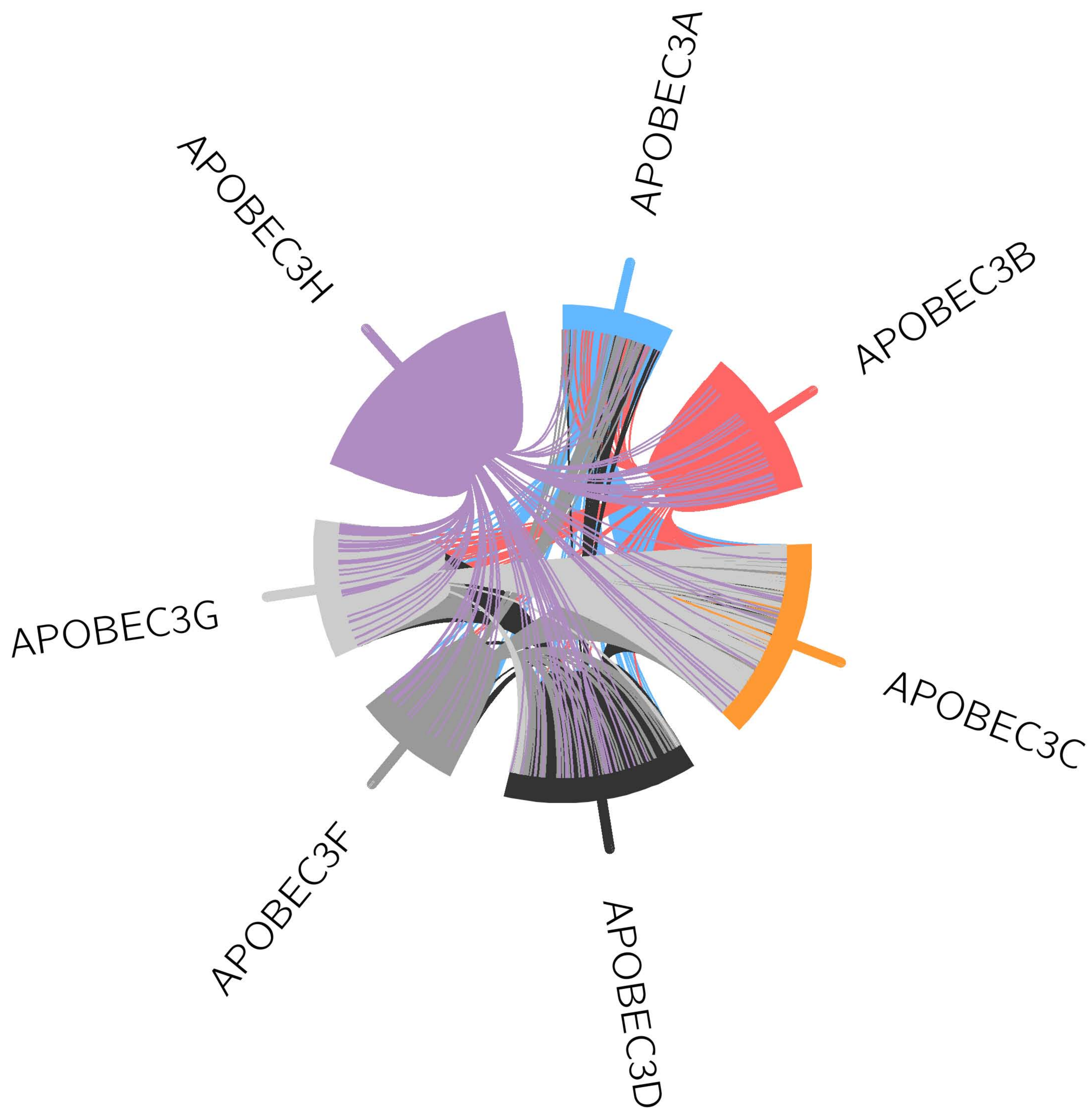

## GTEx.Ovary

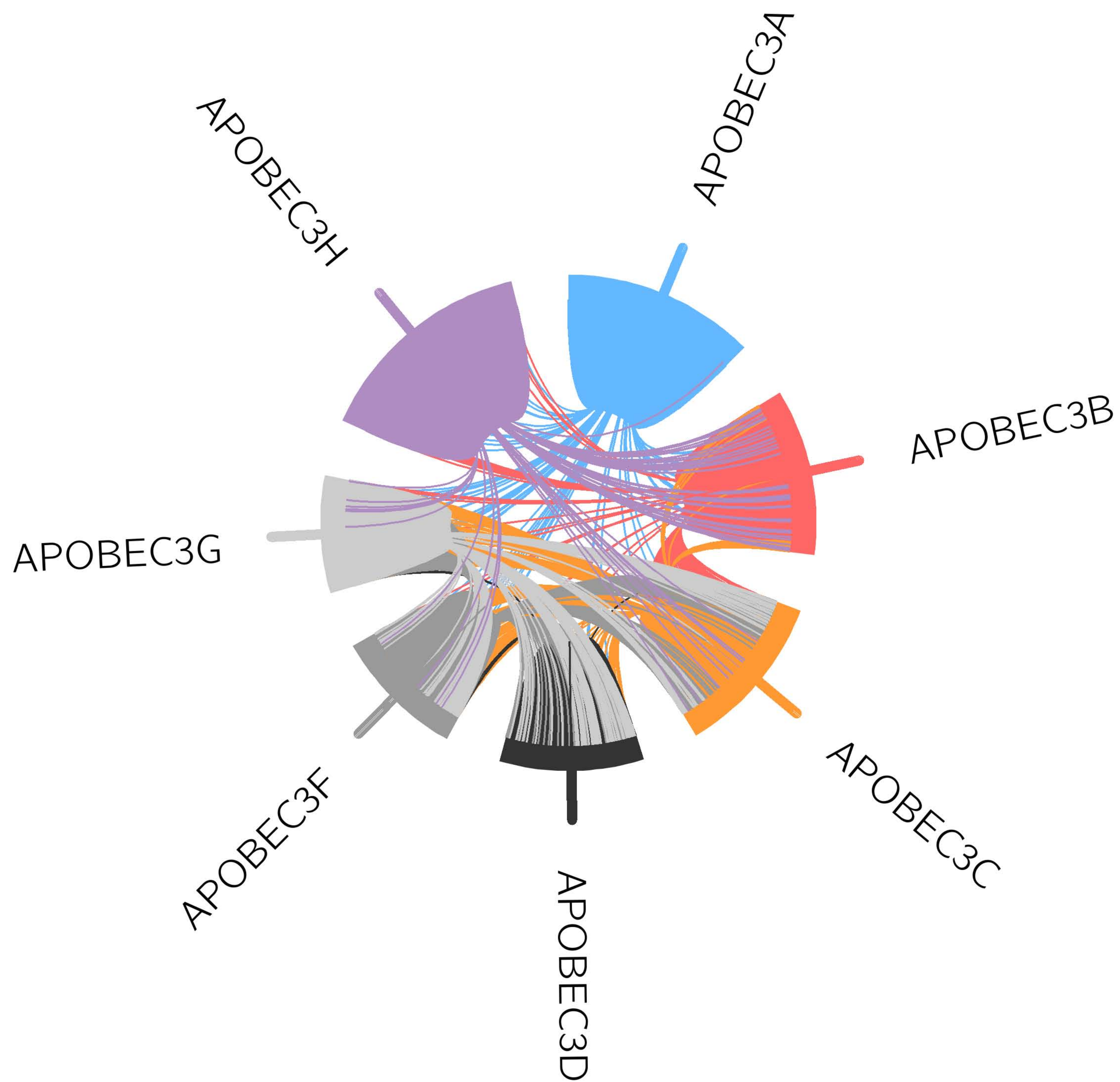

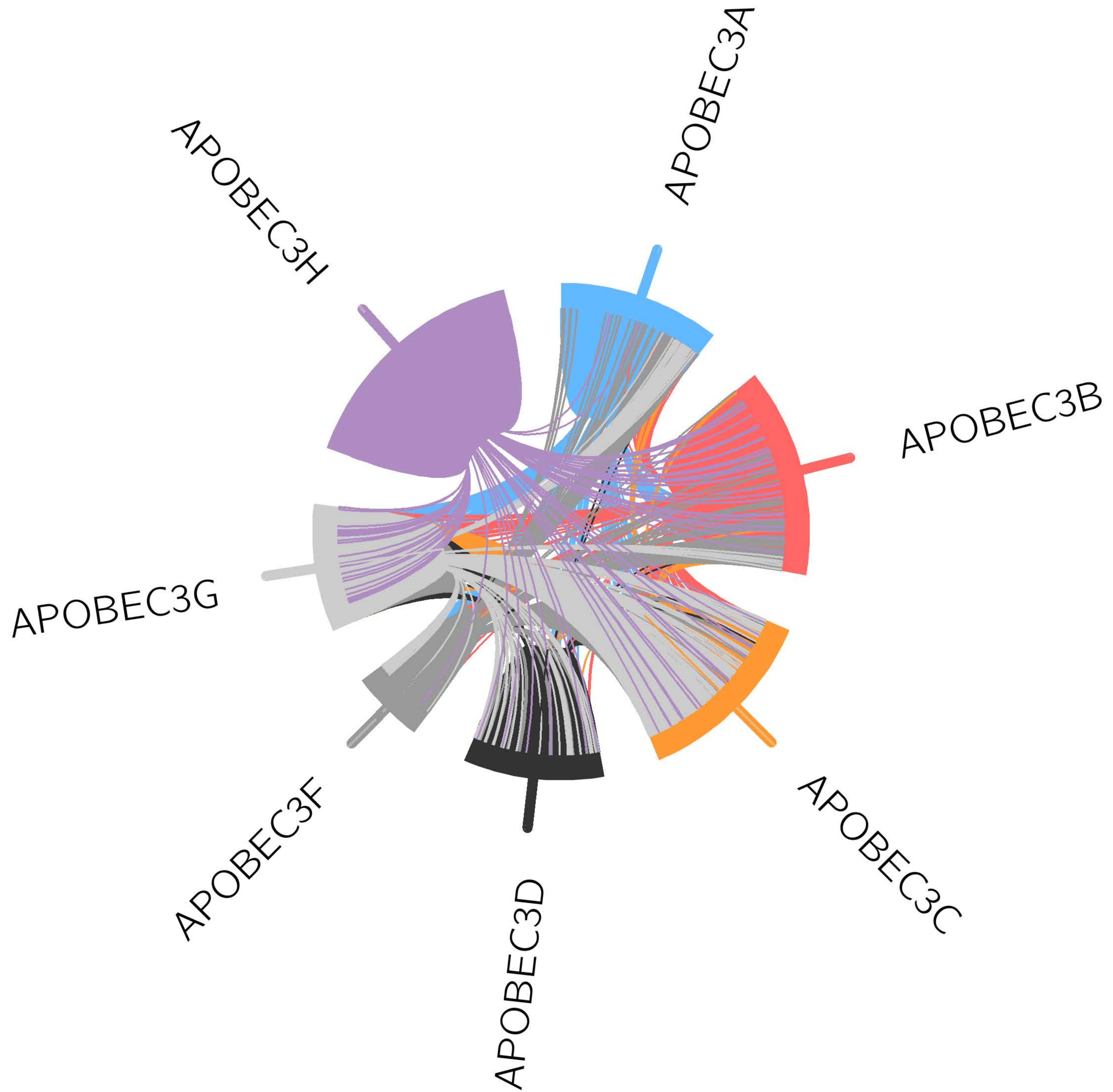

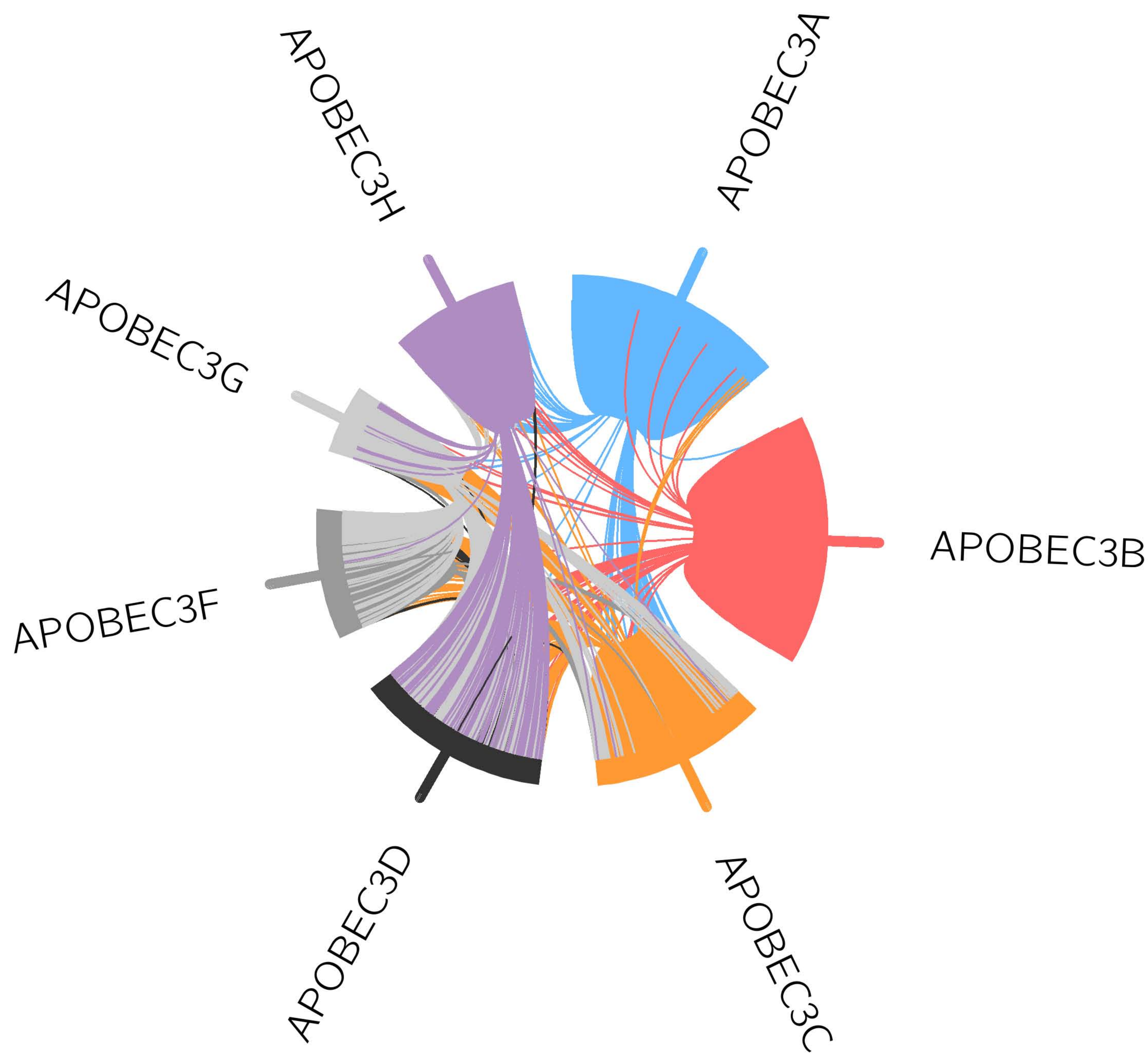

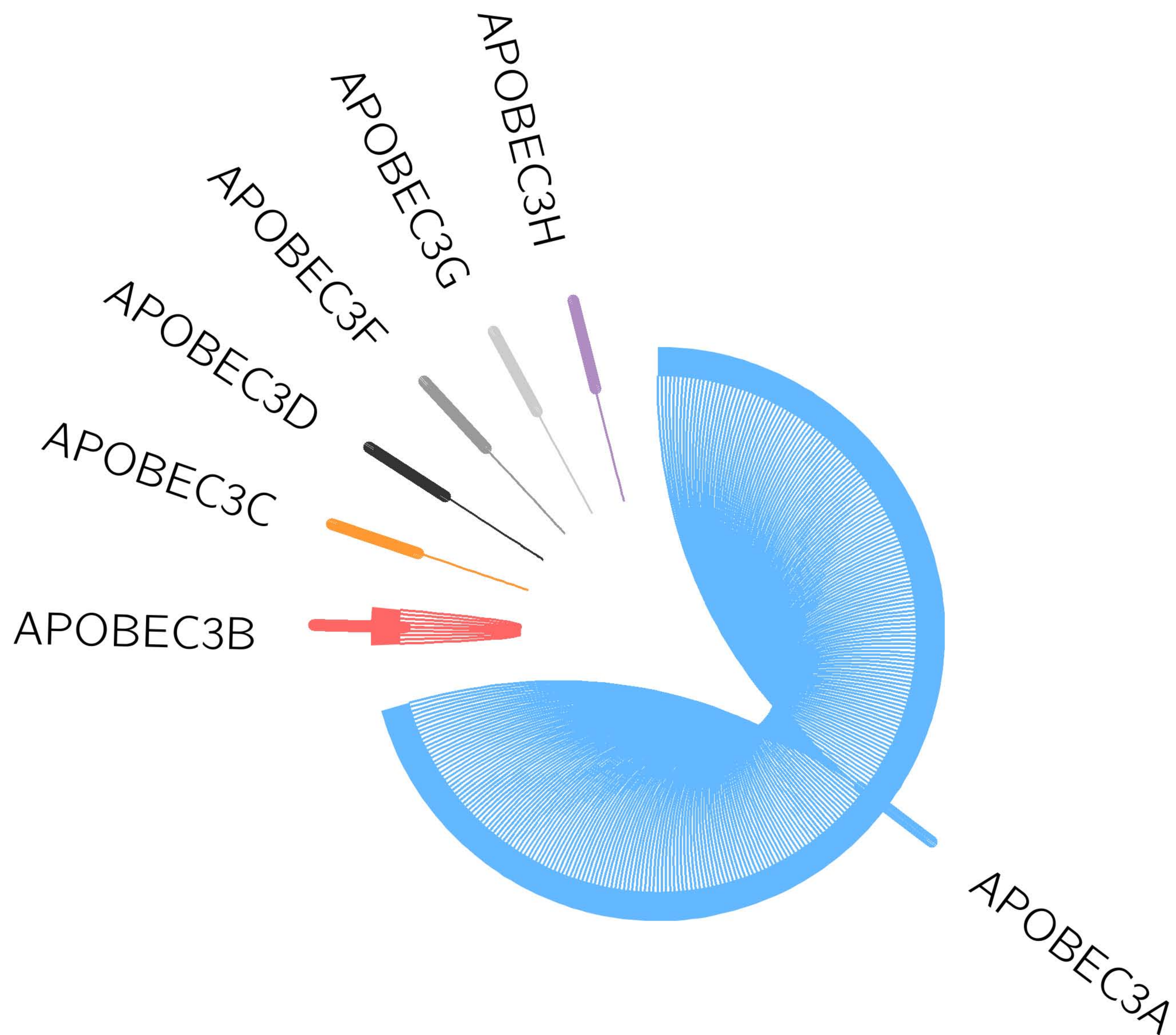

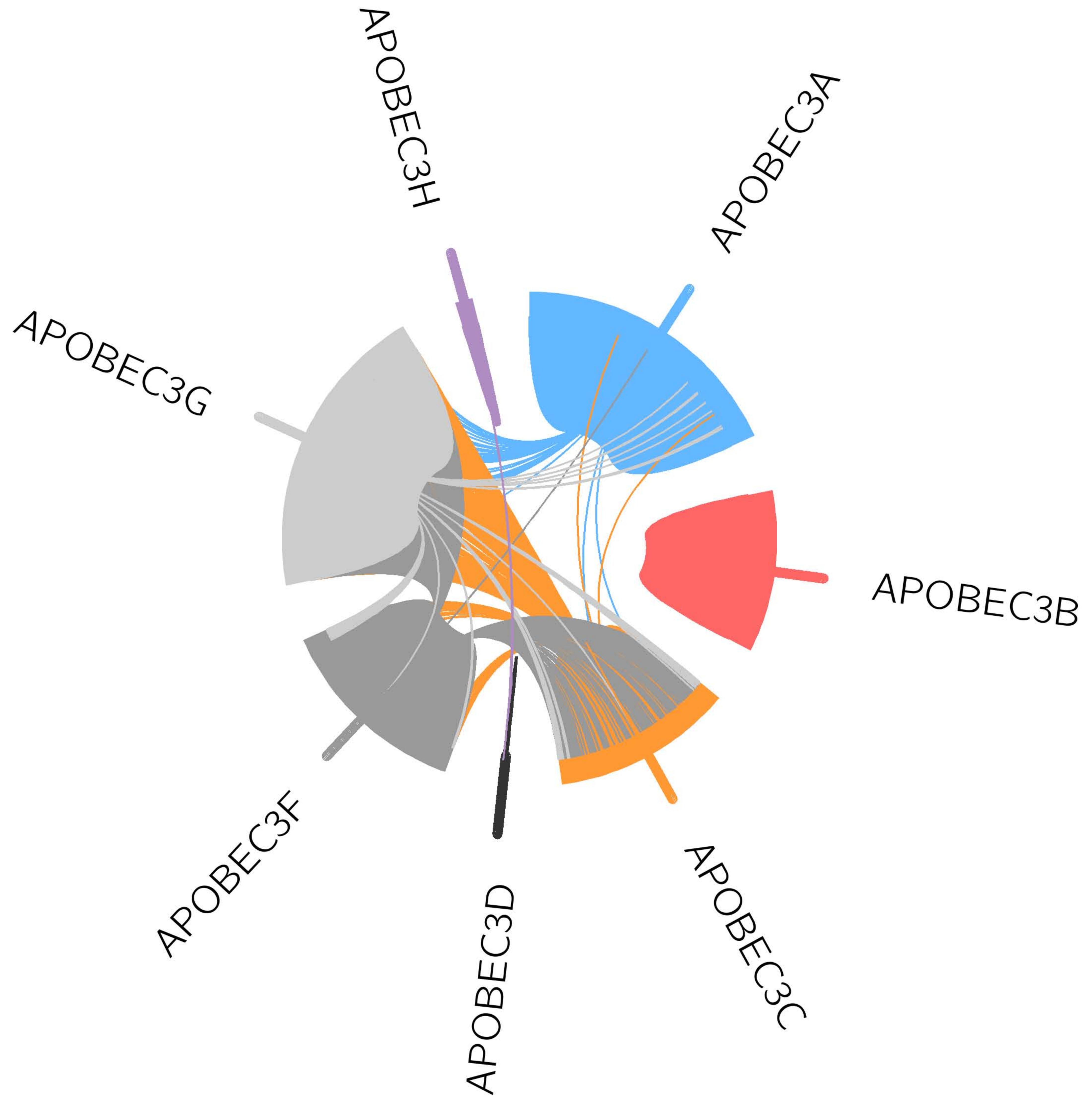

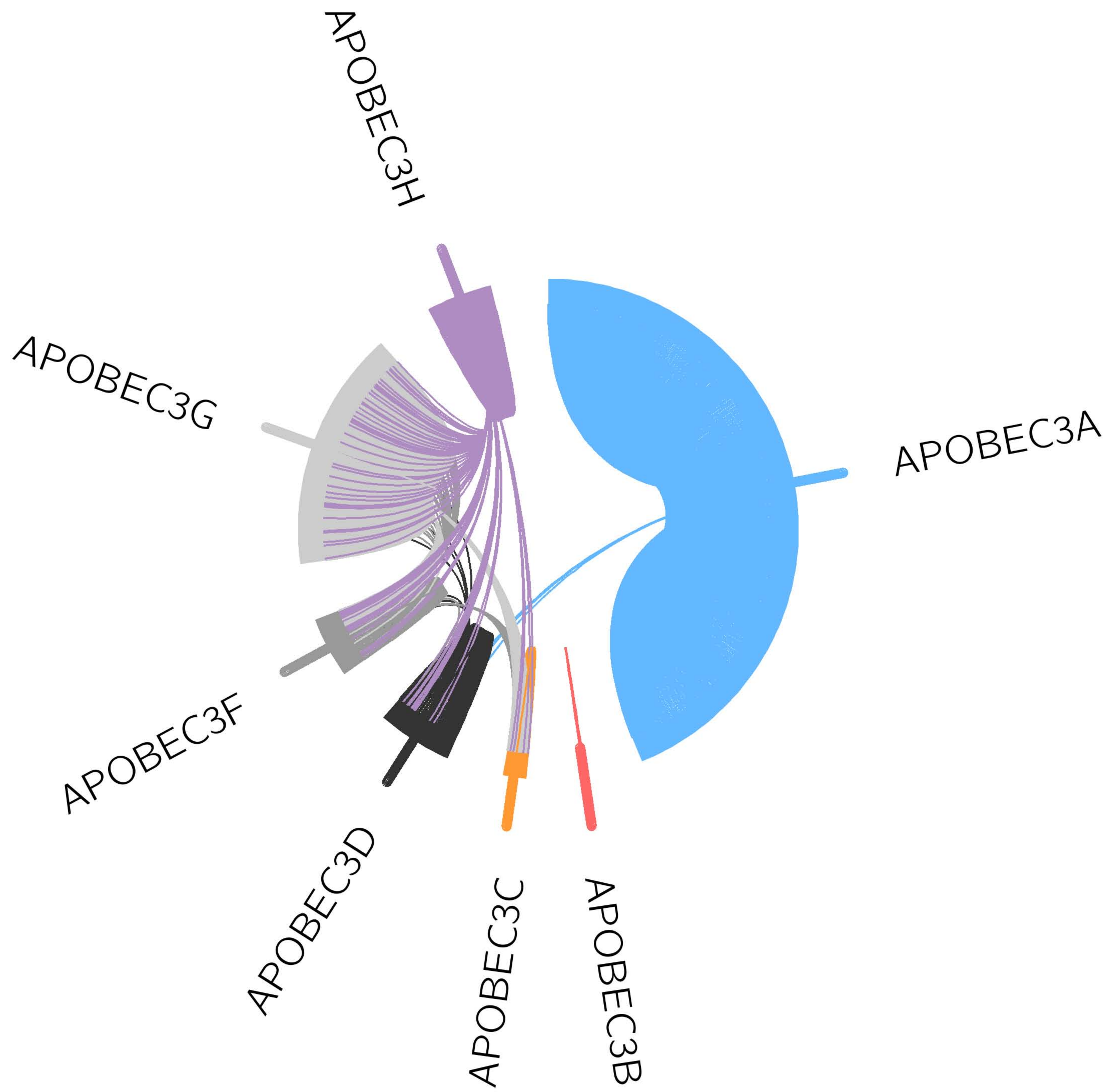

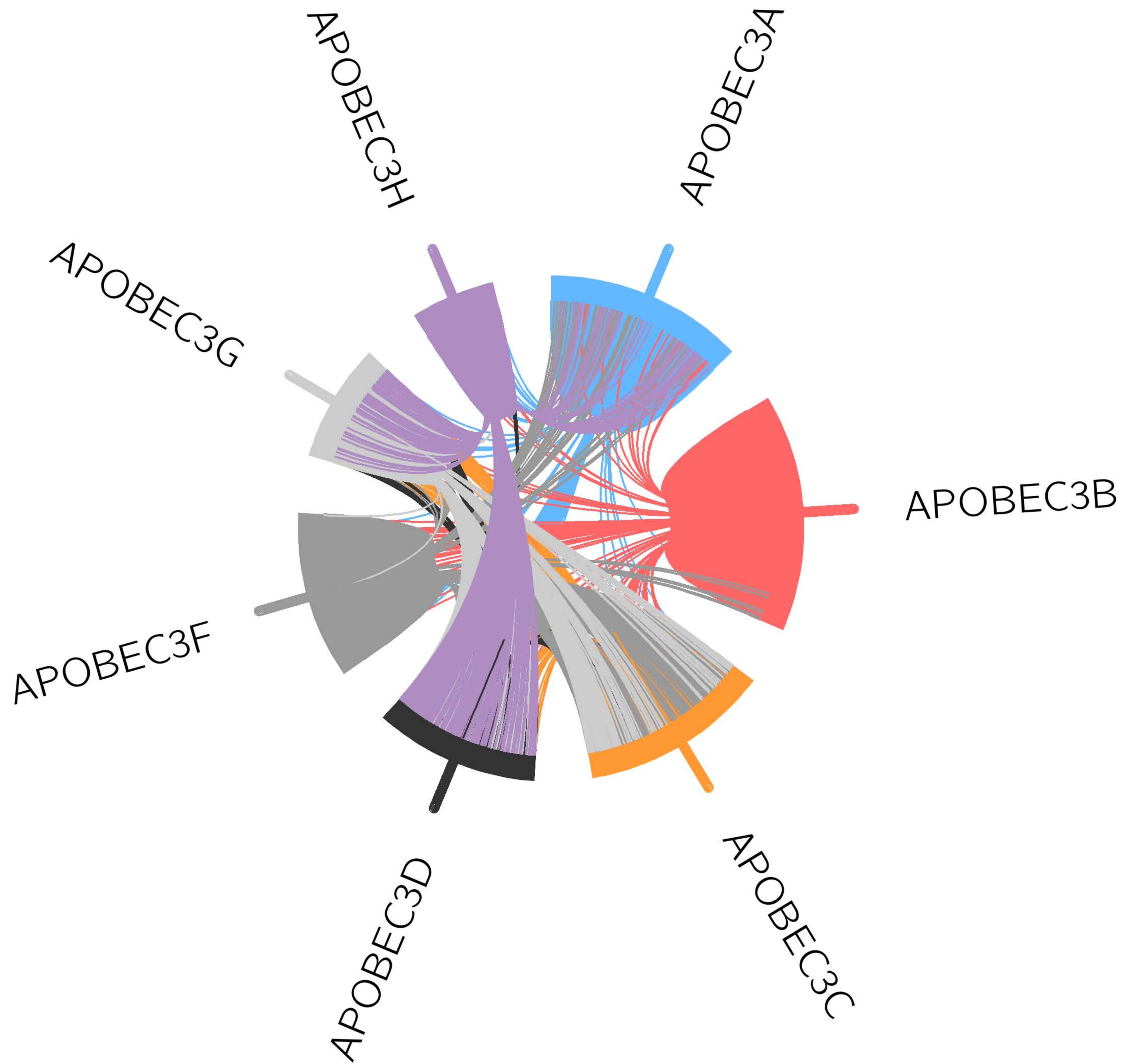

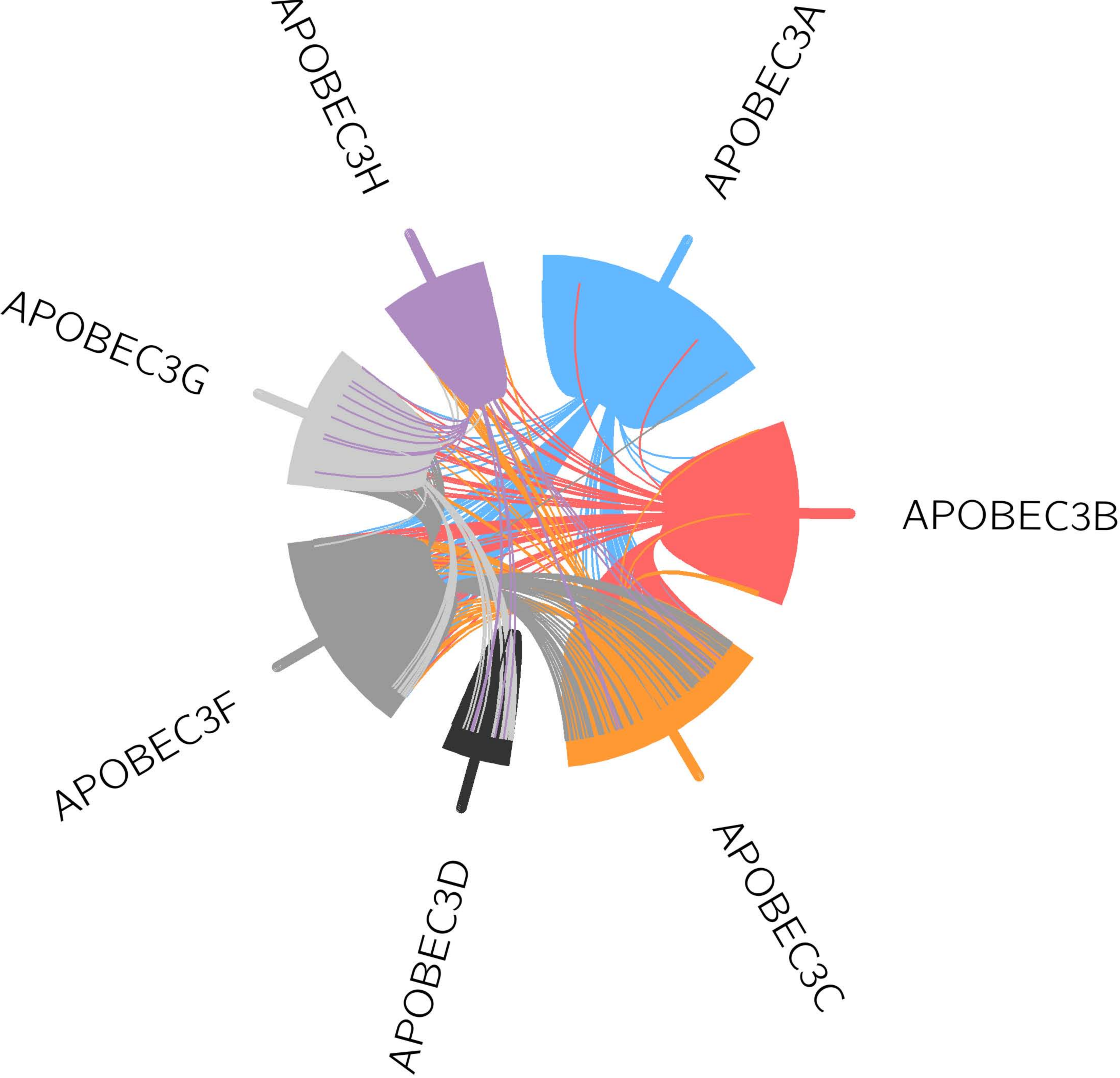

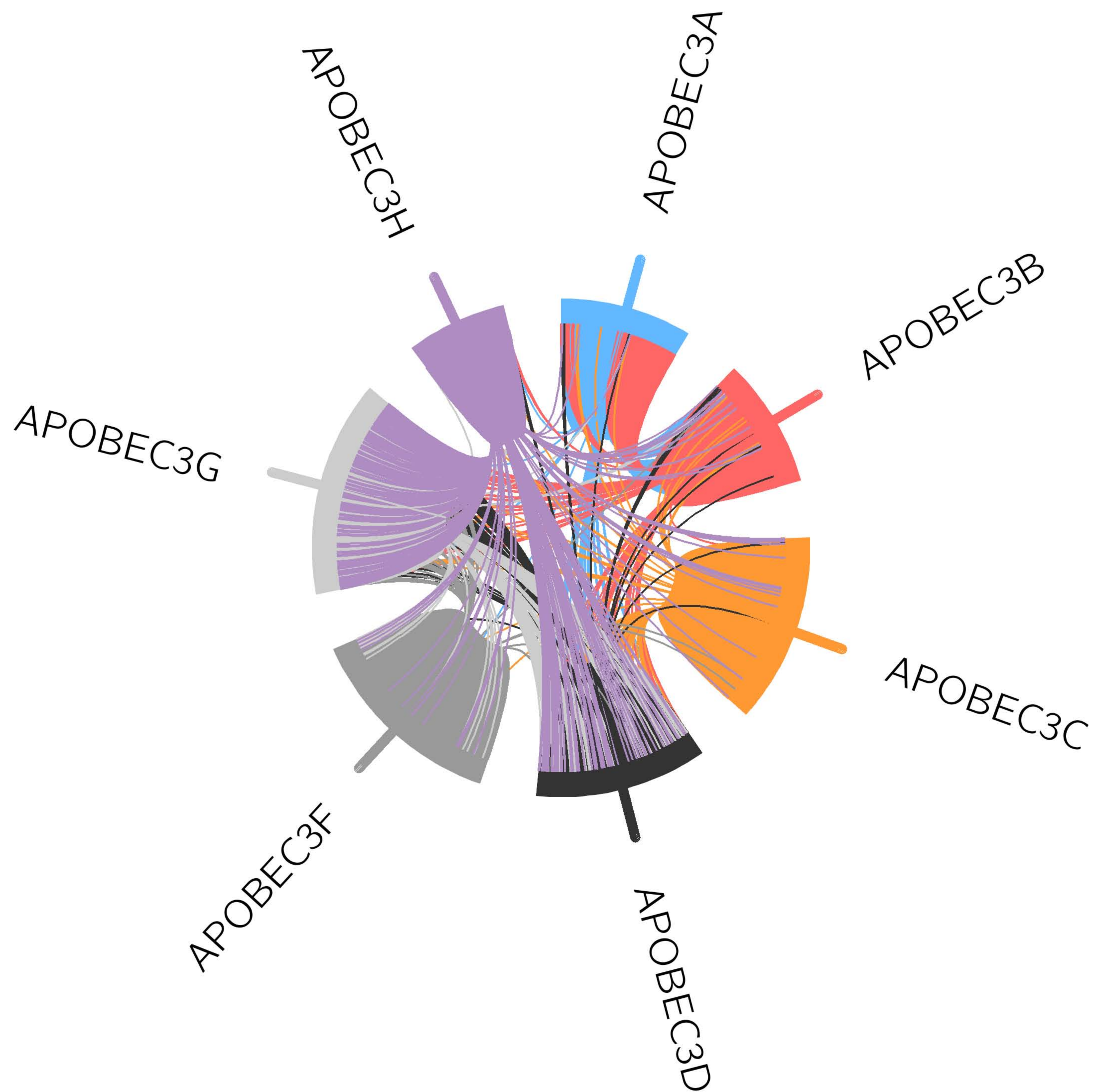

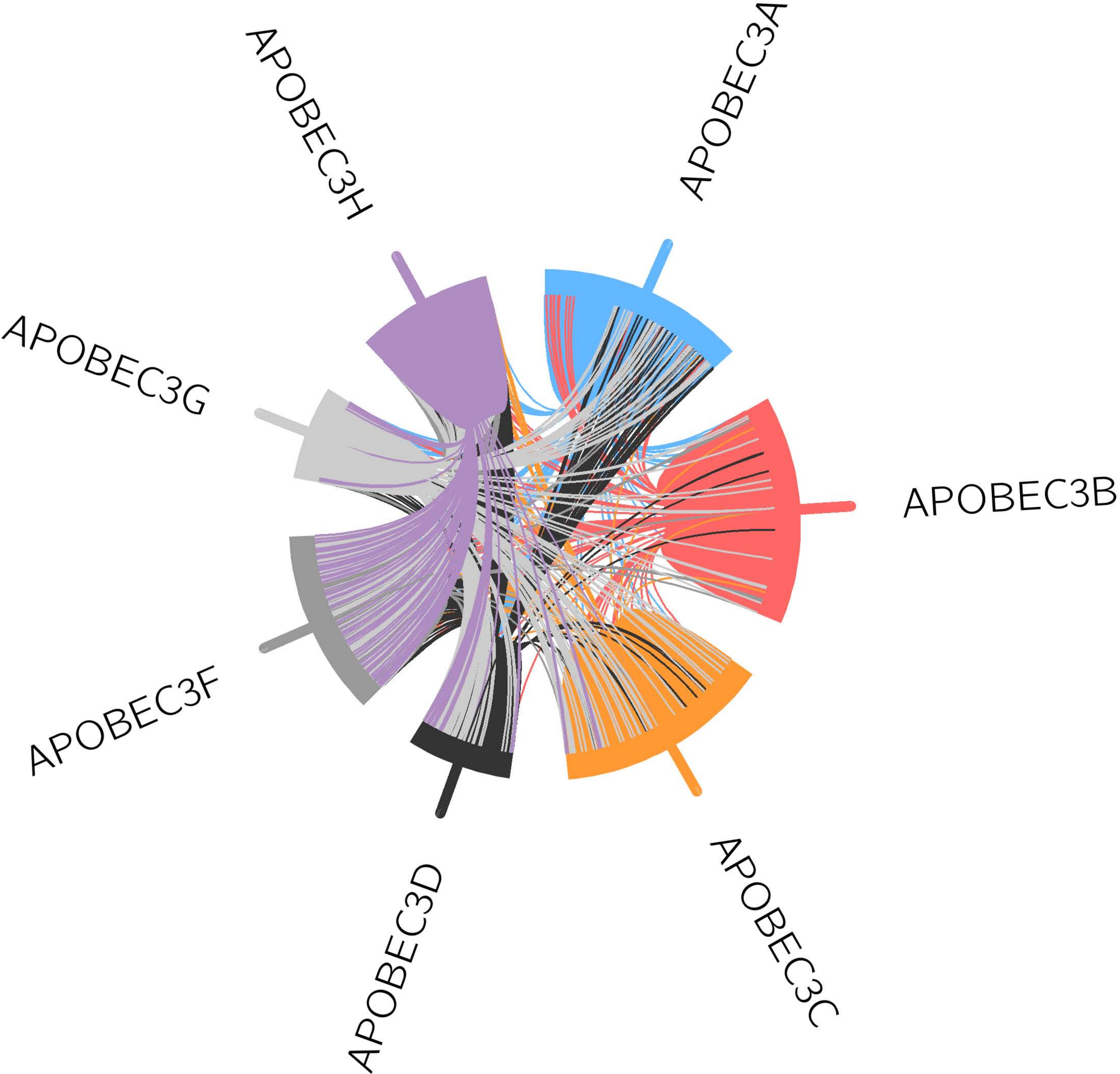

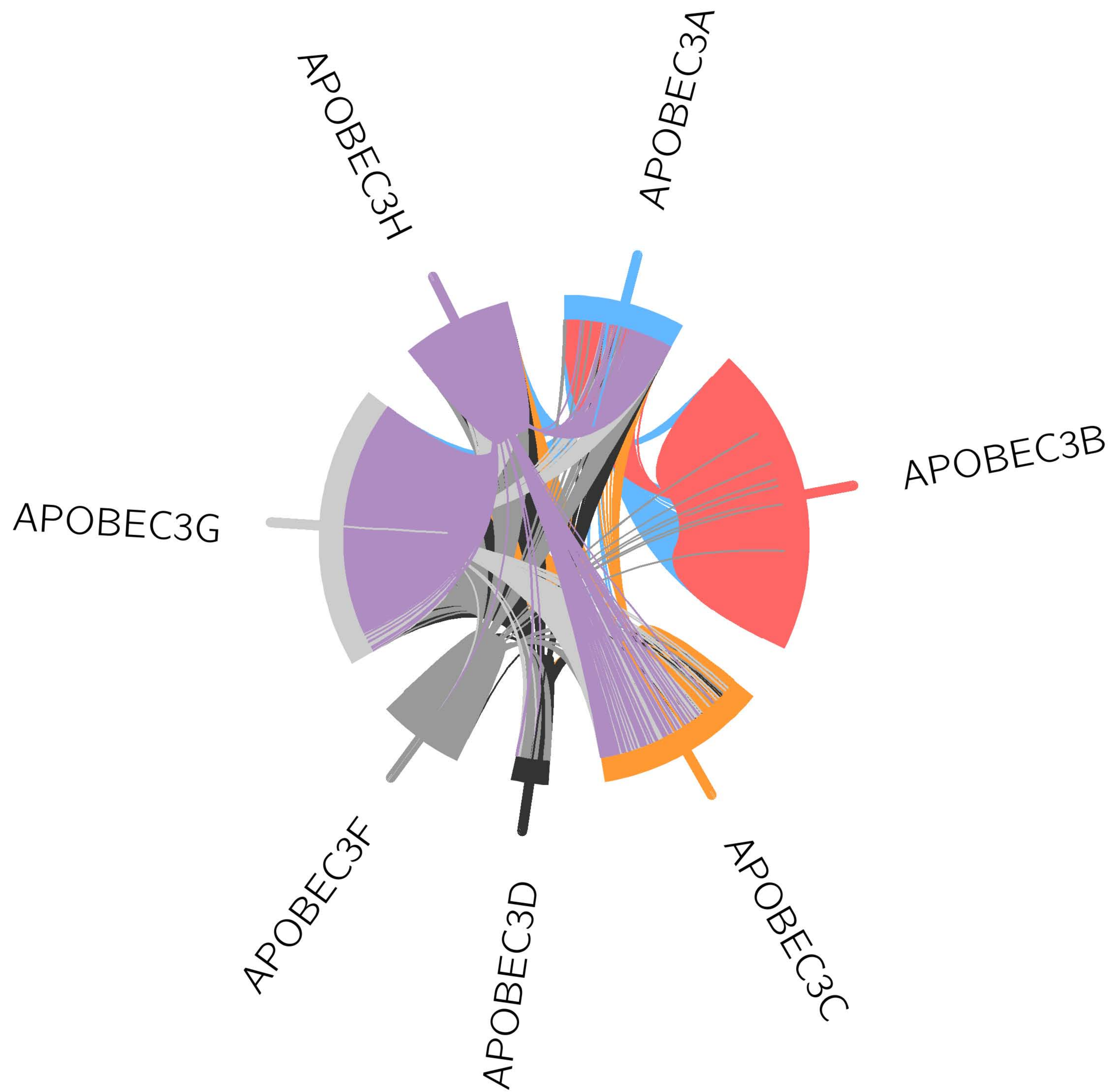

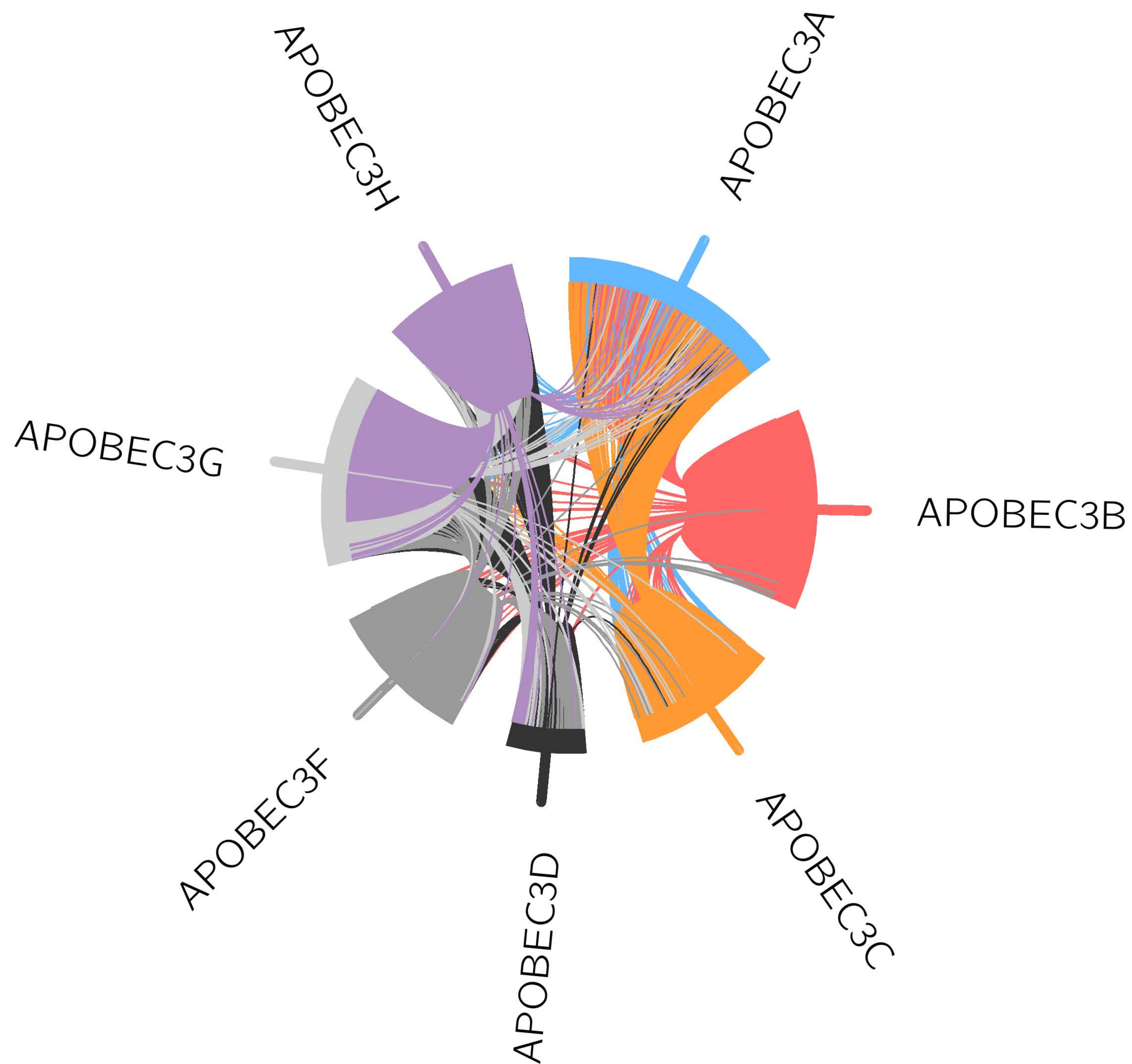

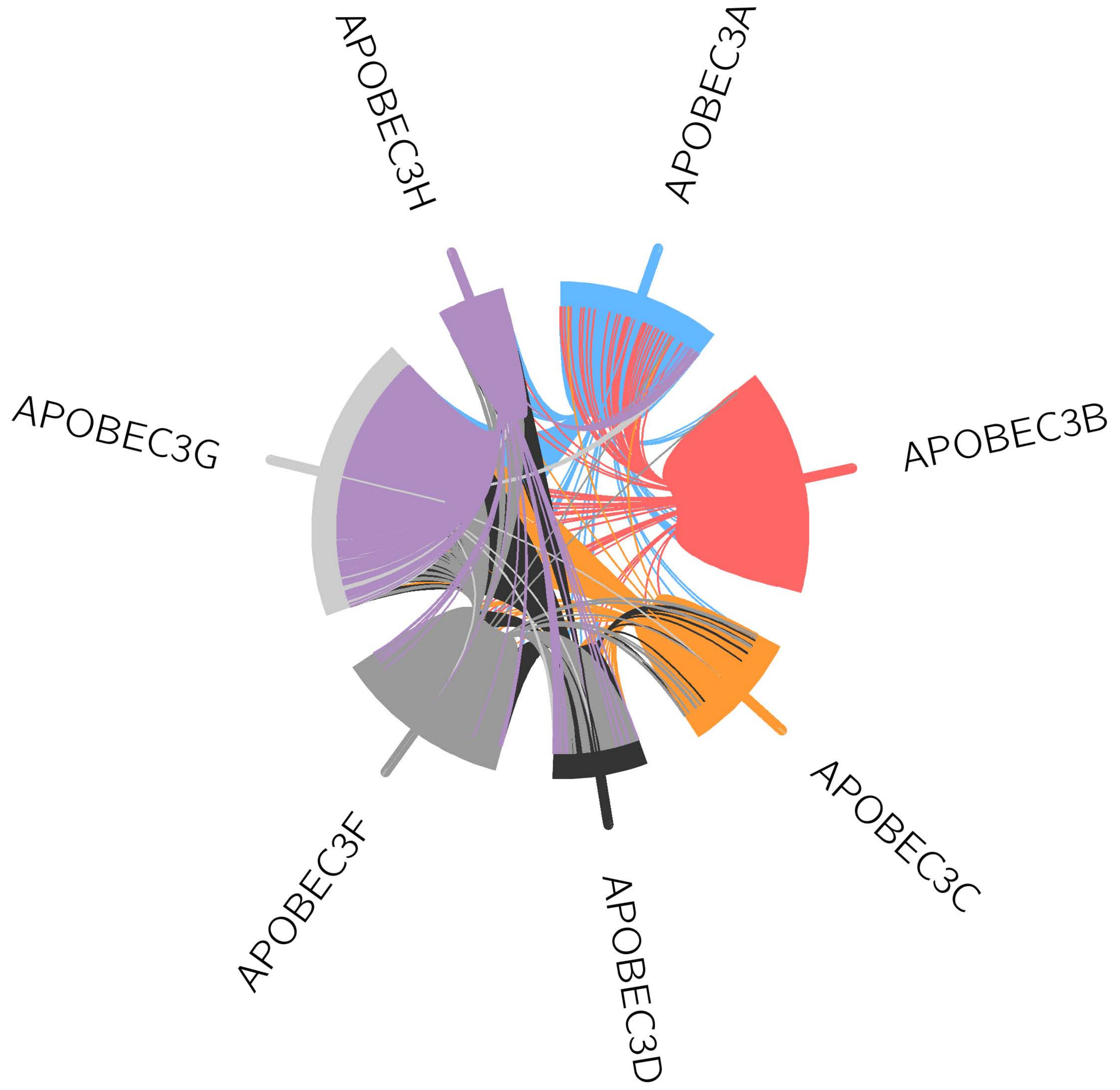

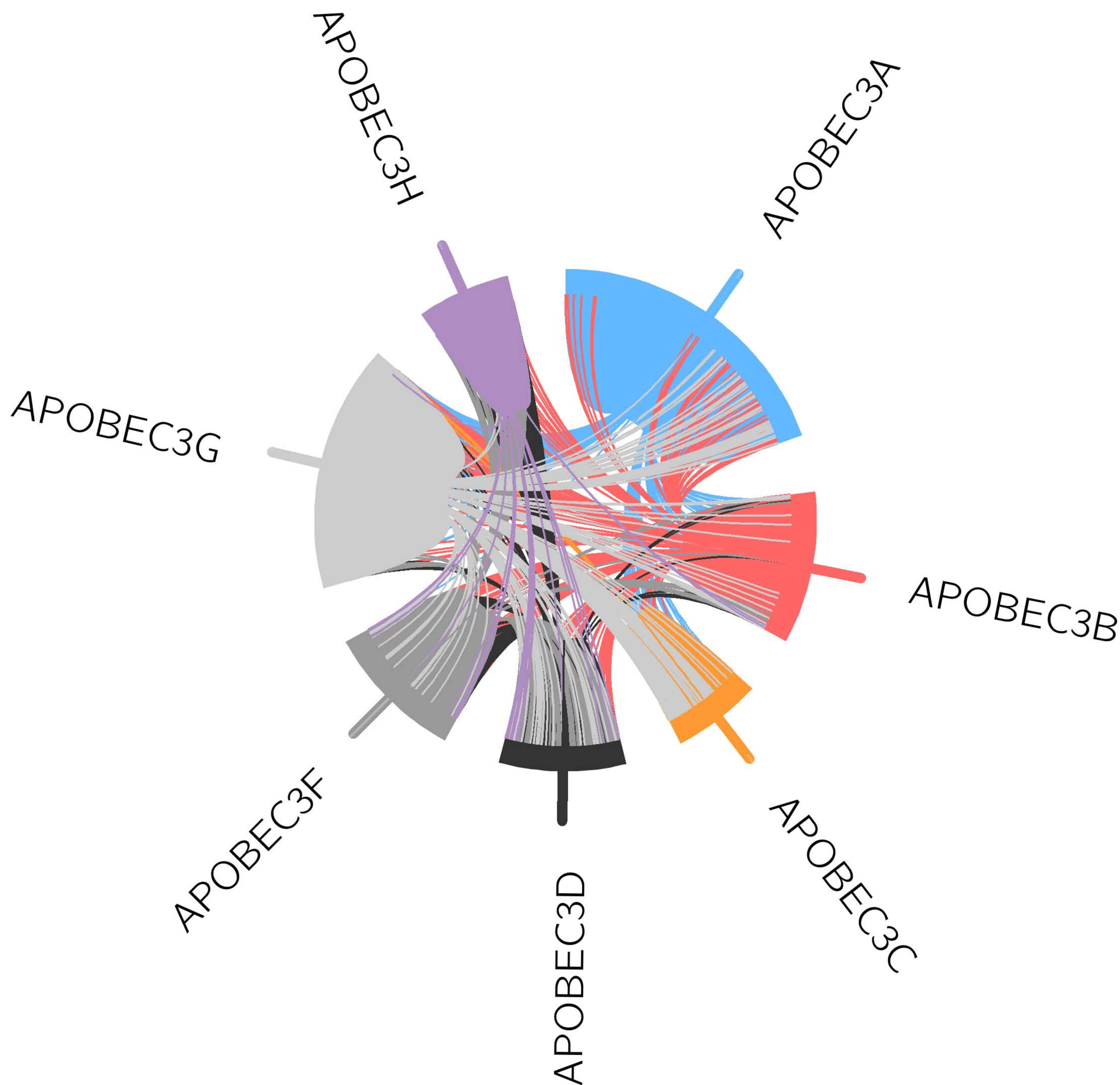

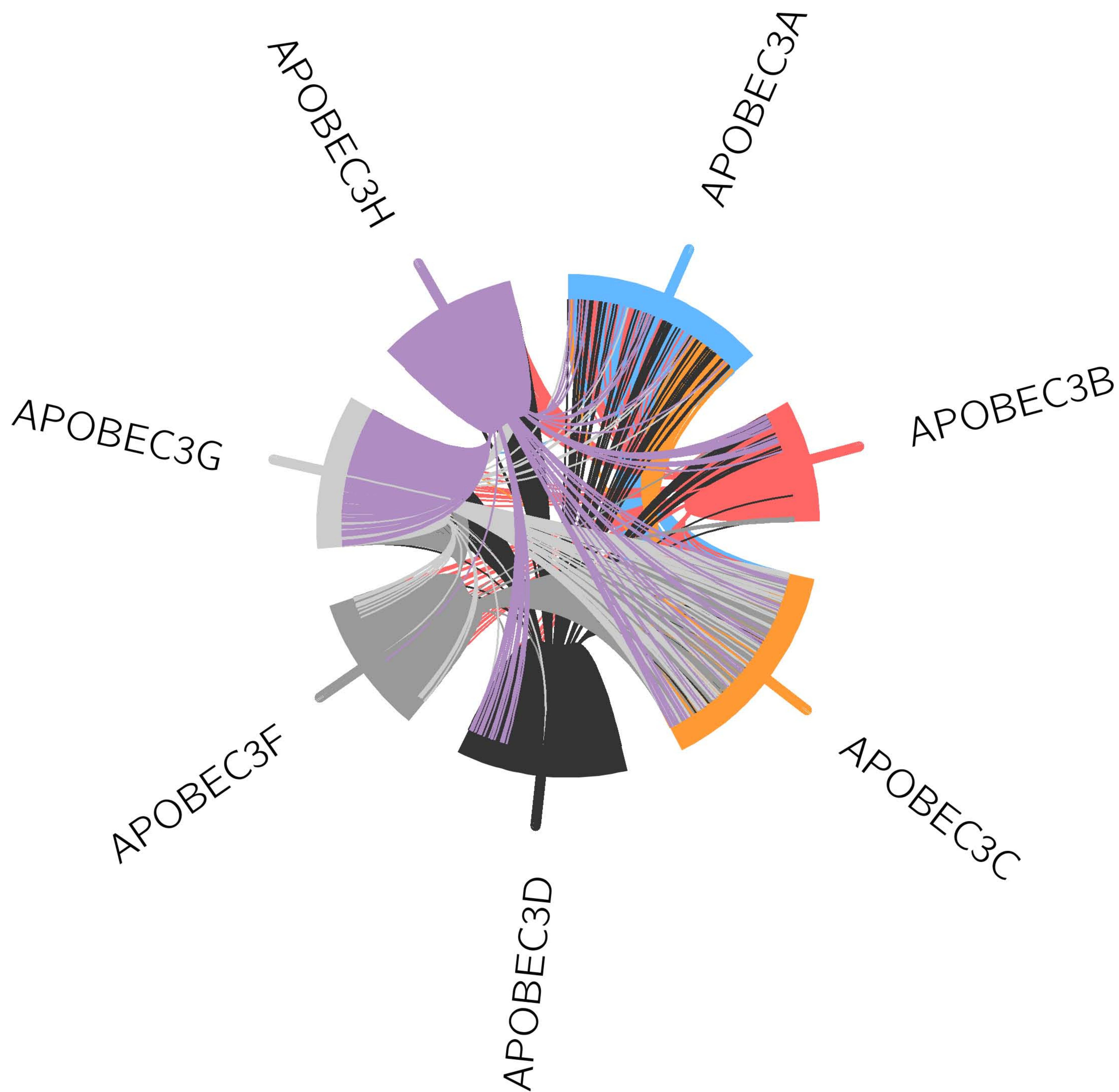

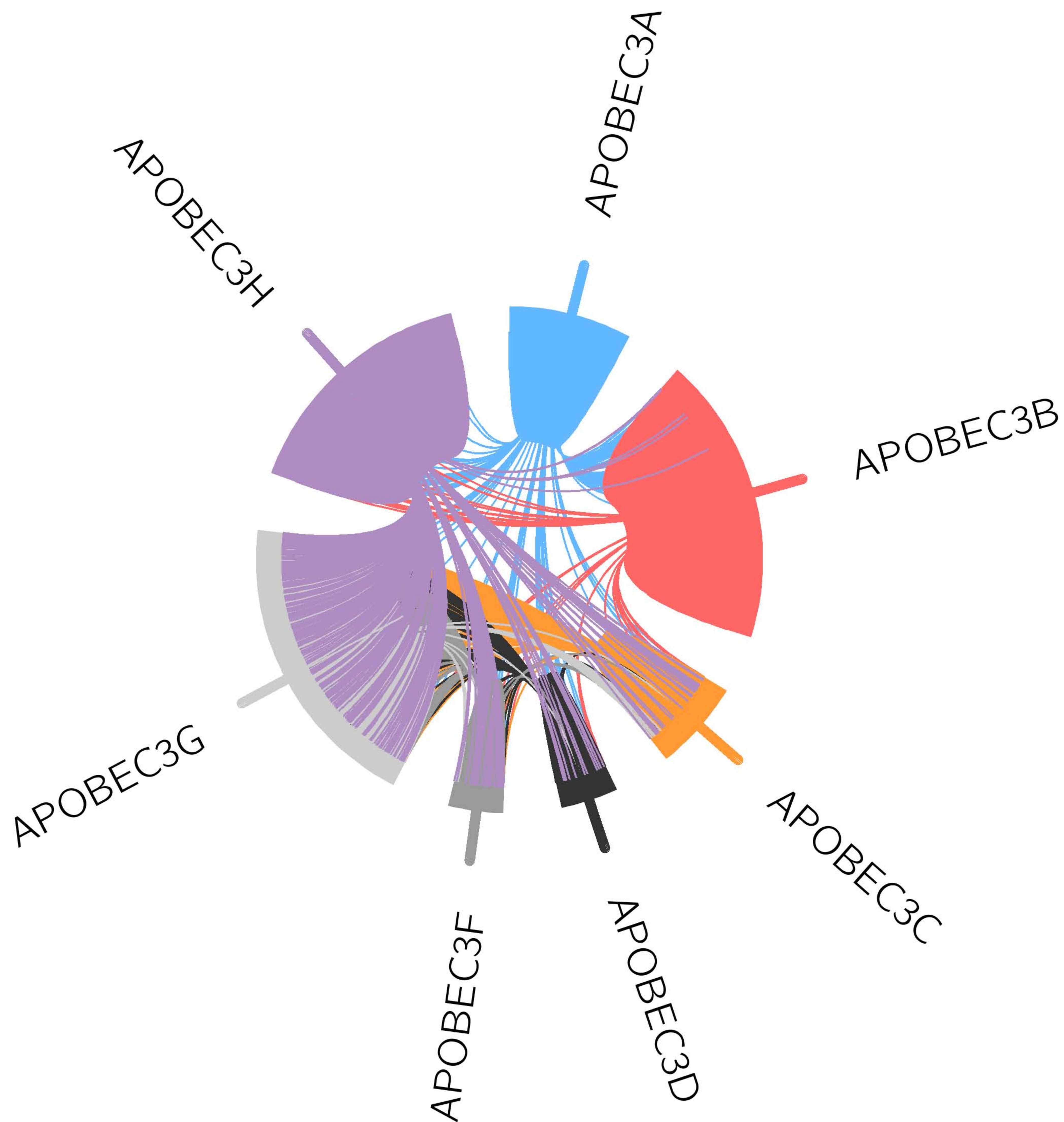

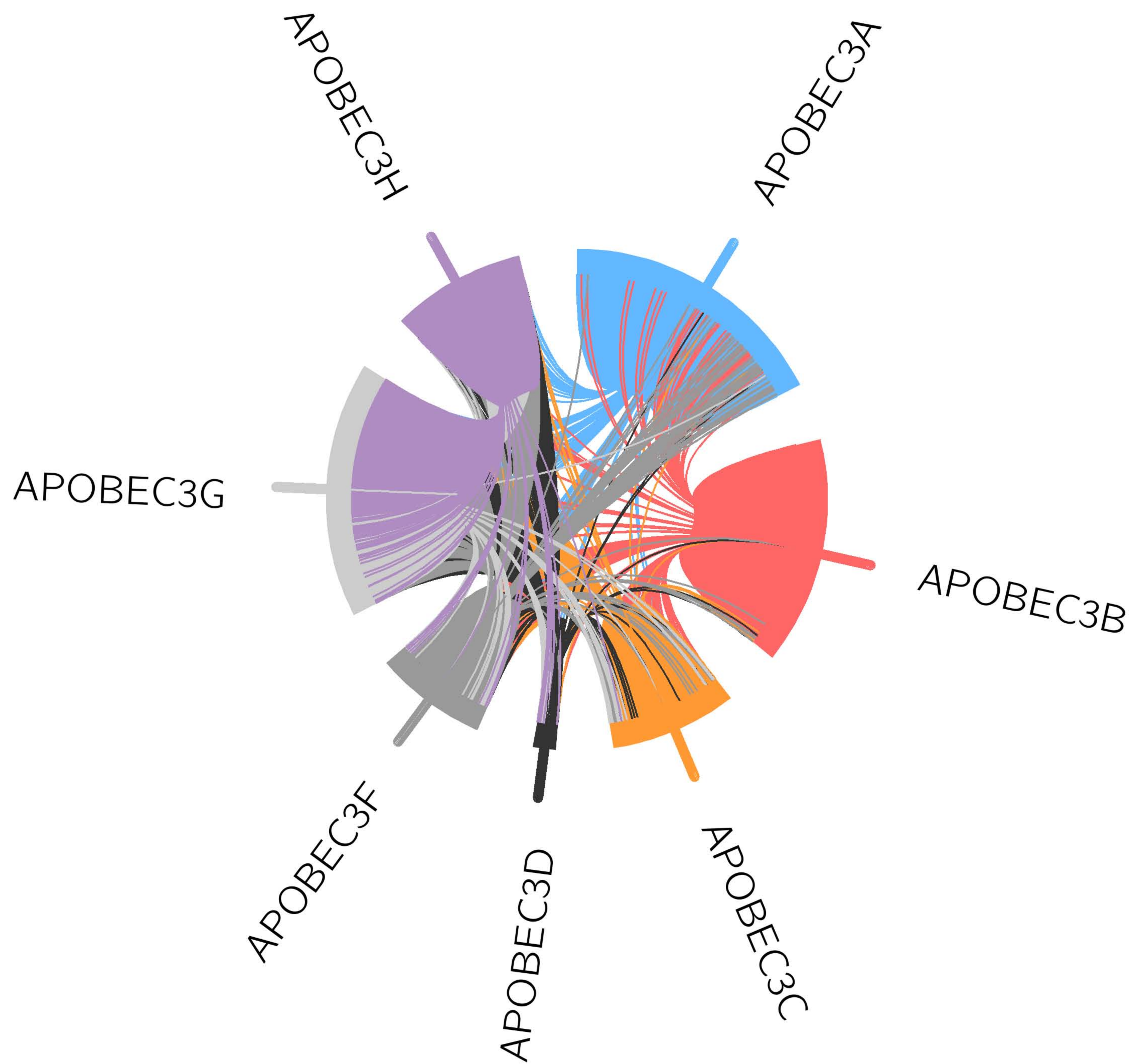

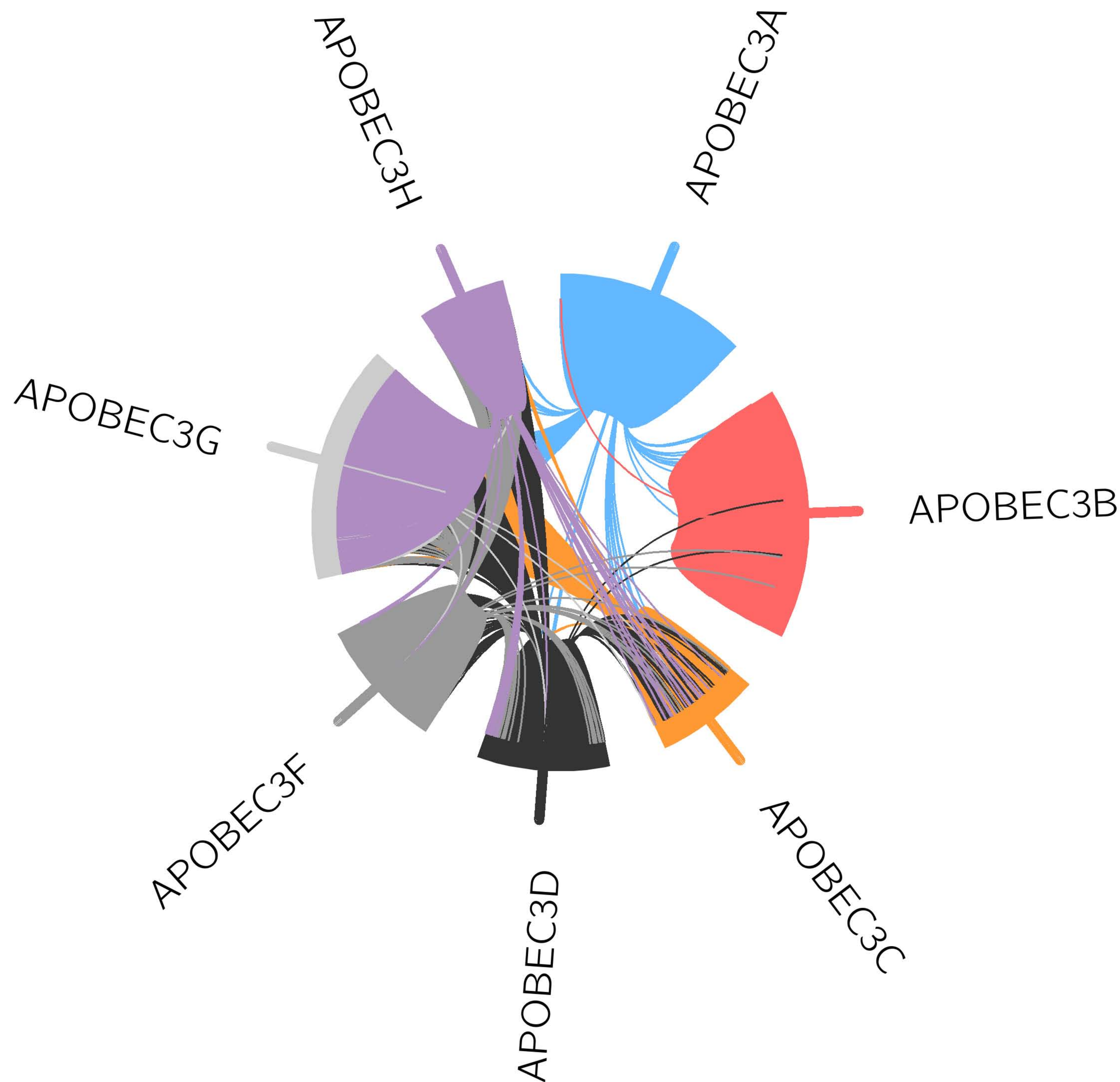

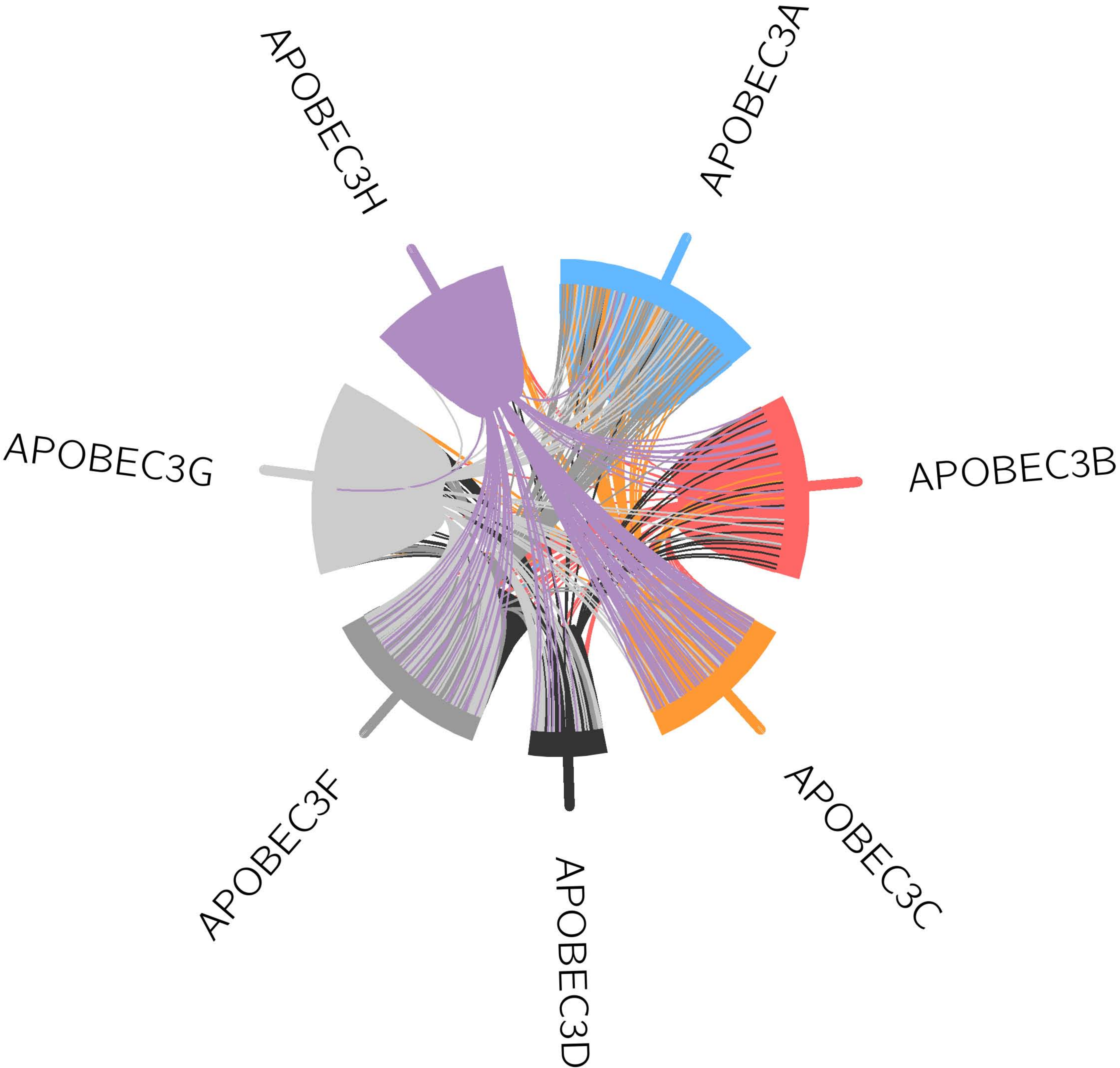

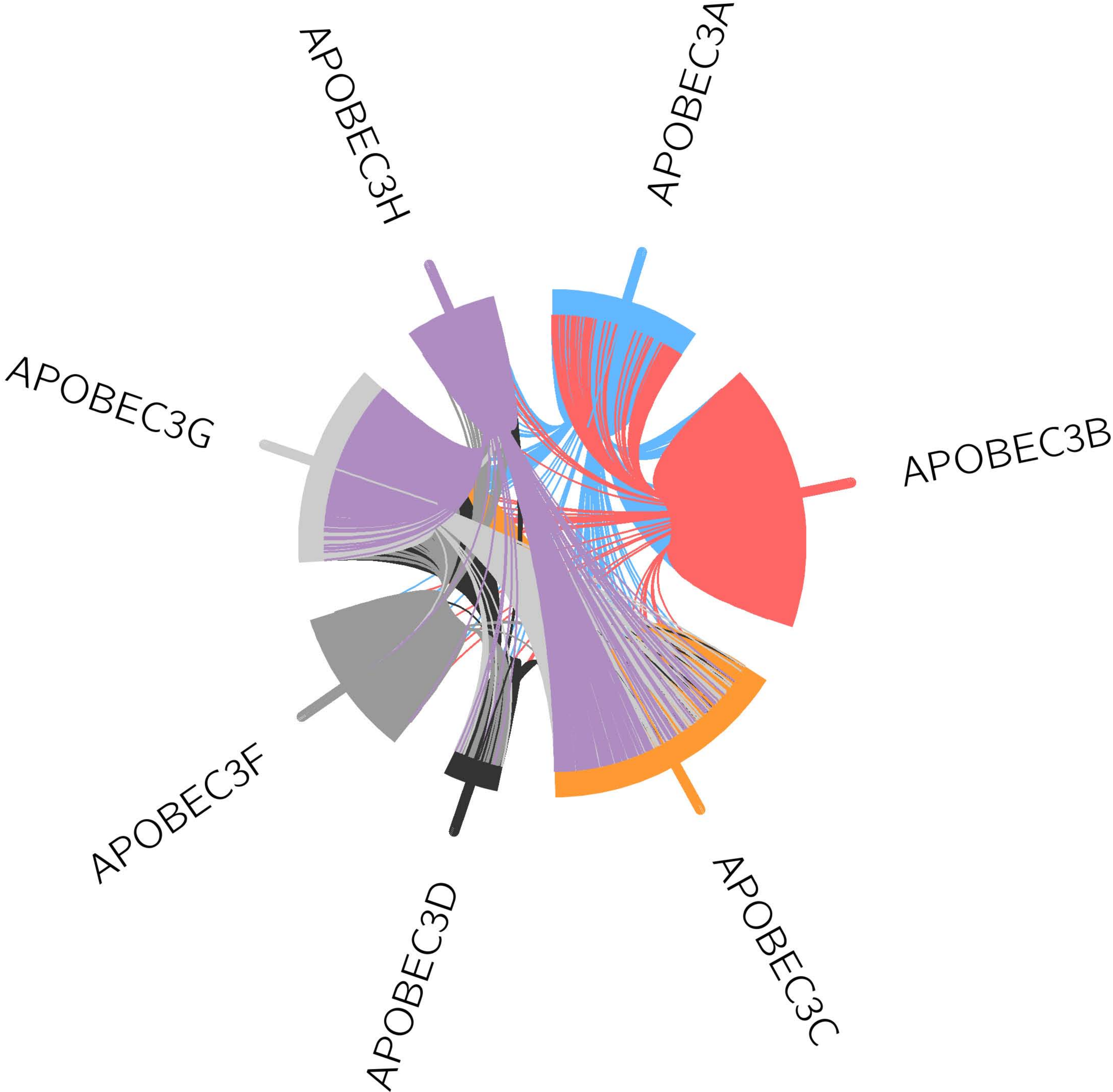

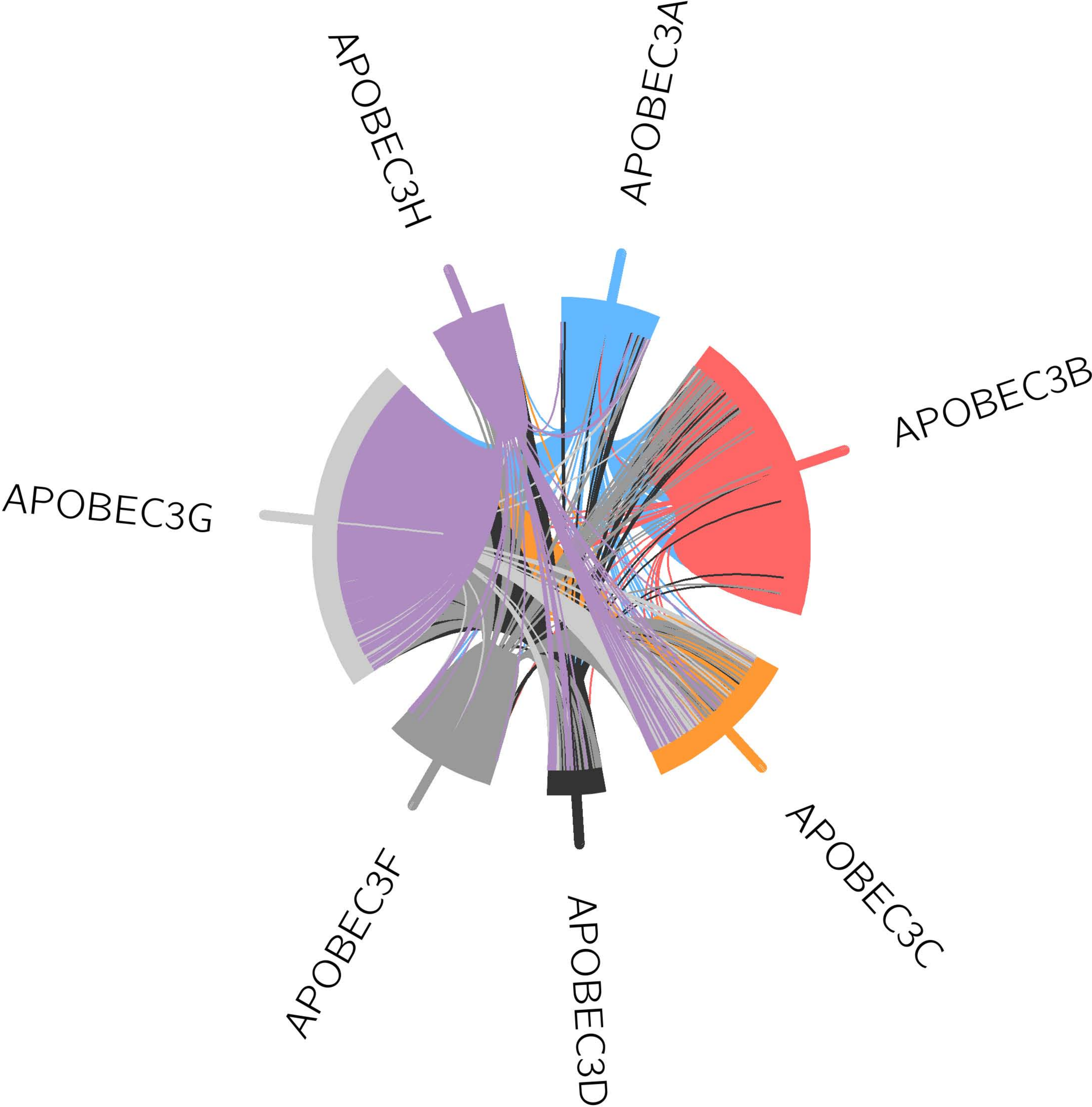

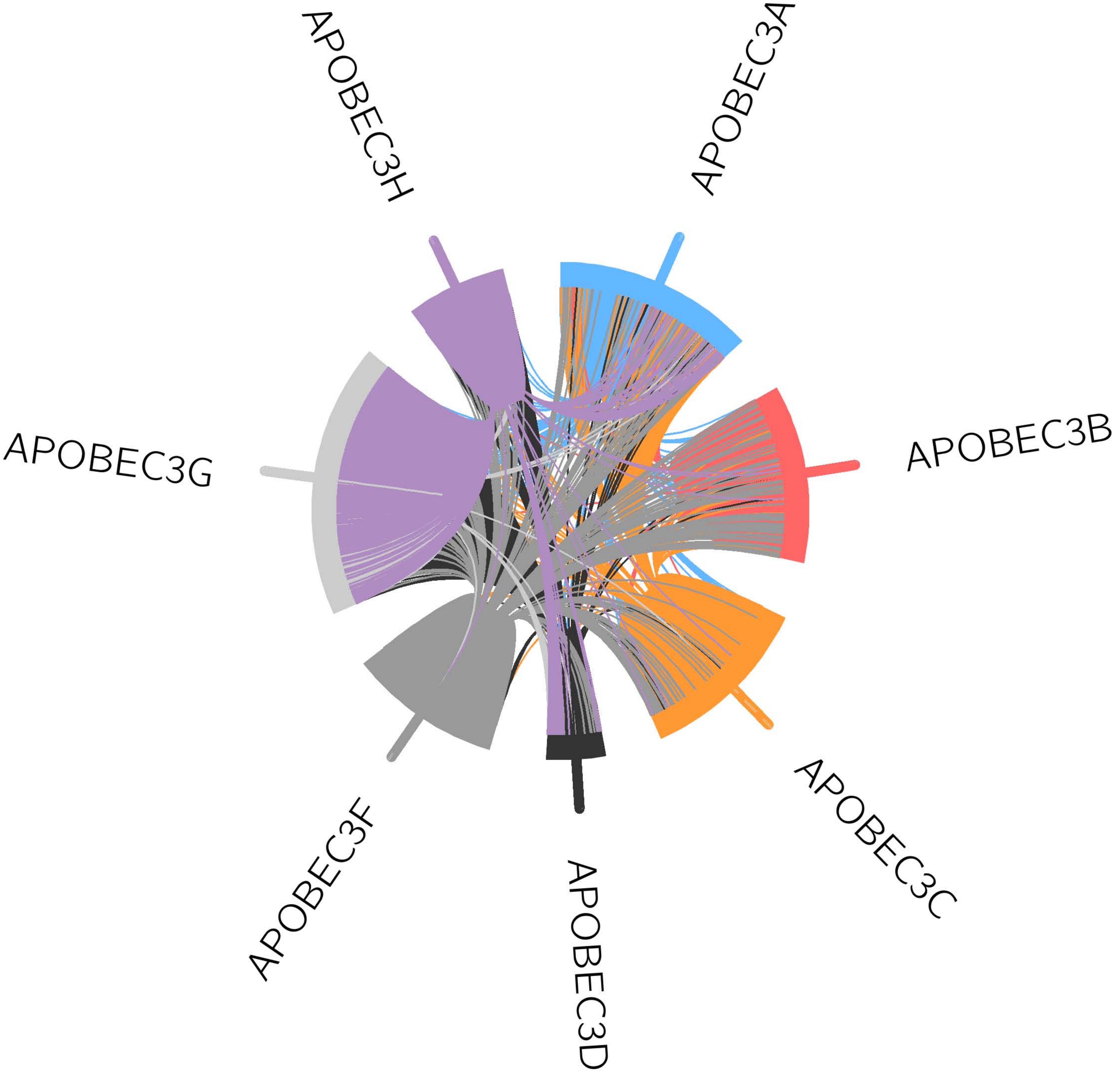

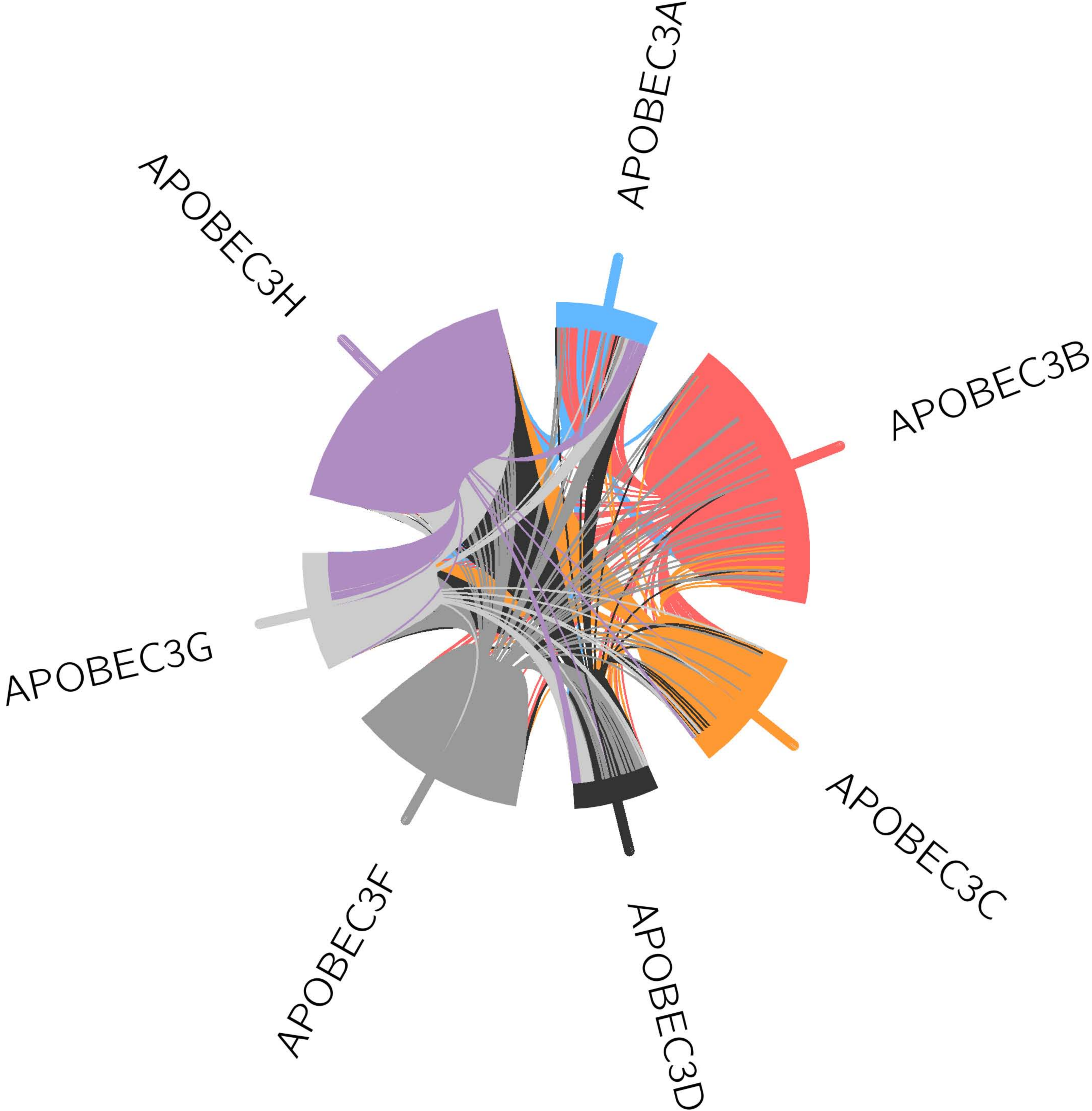

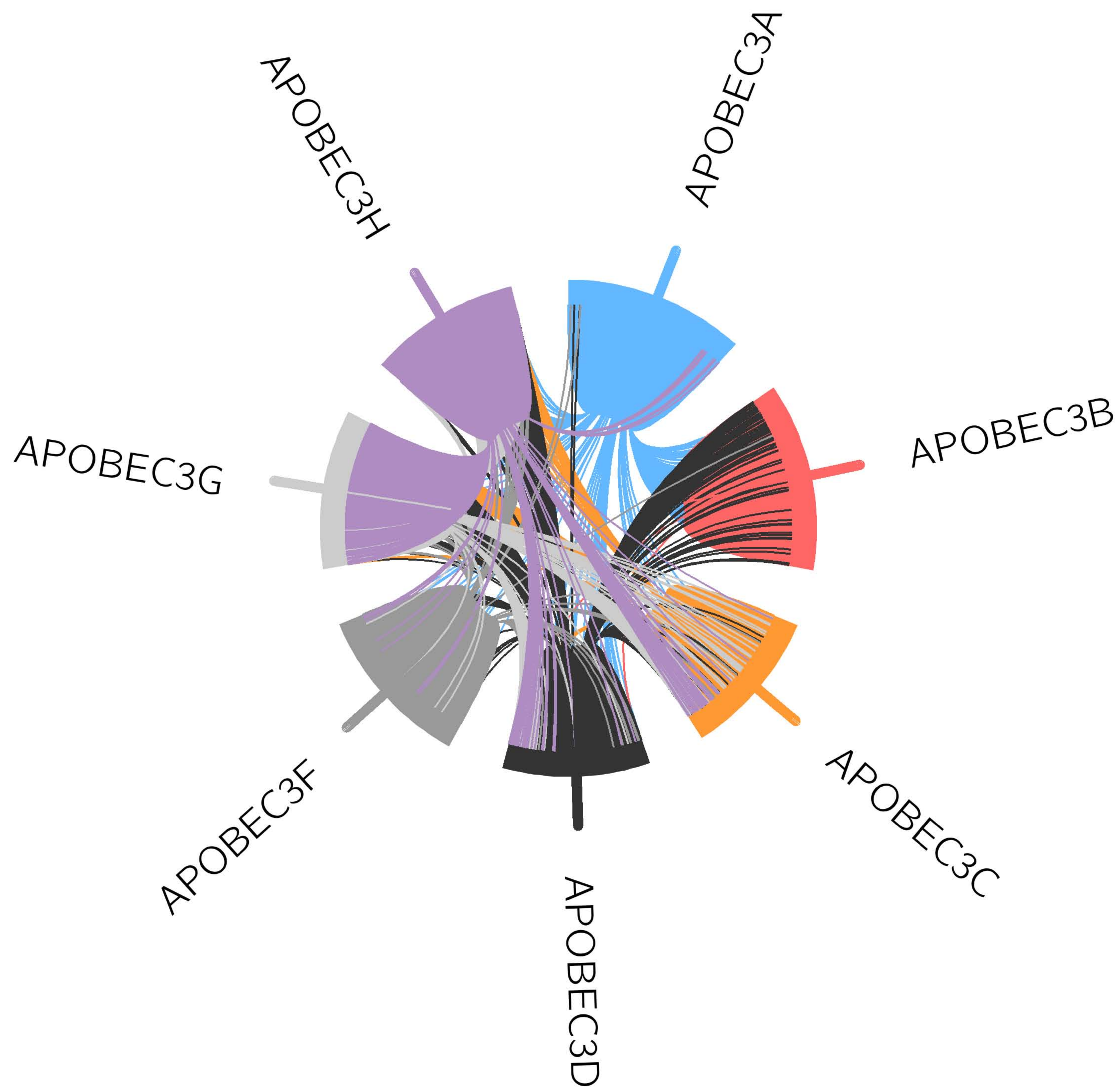

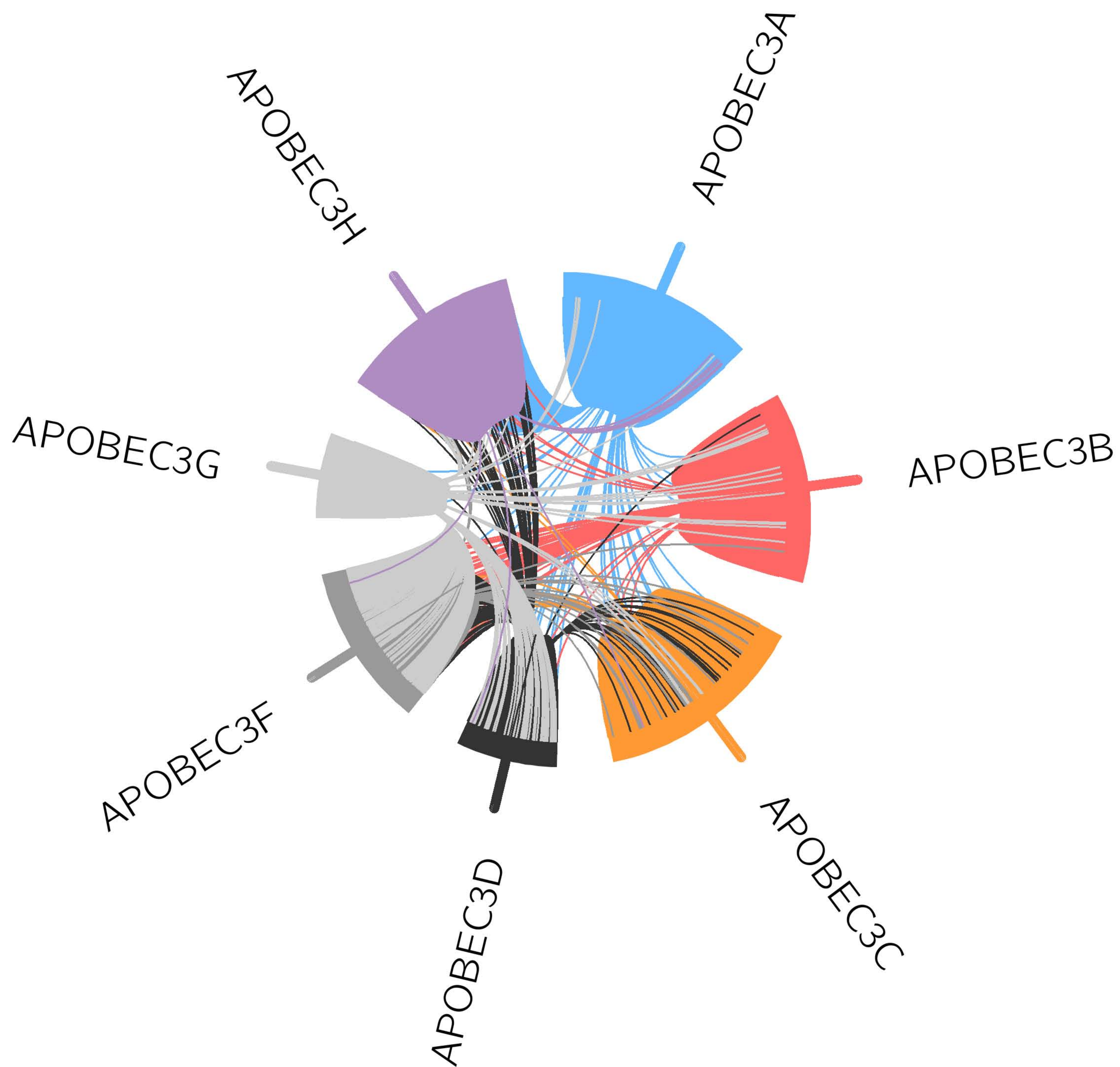

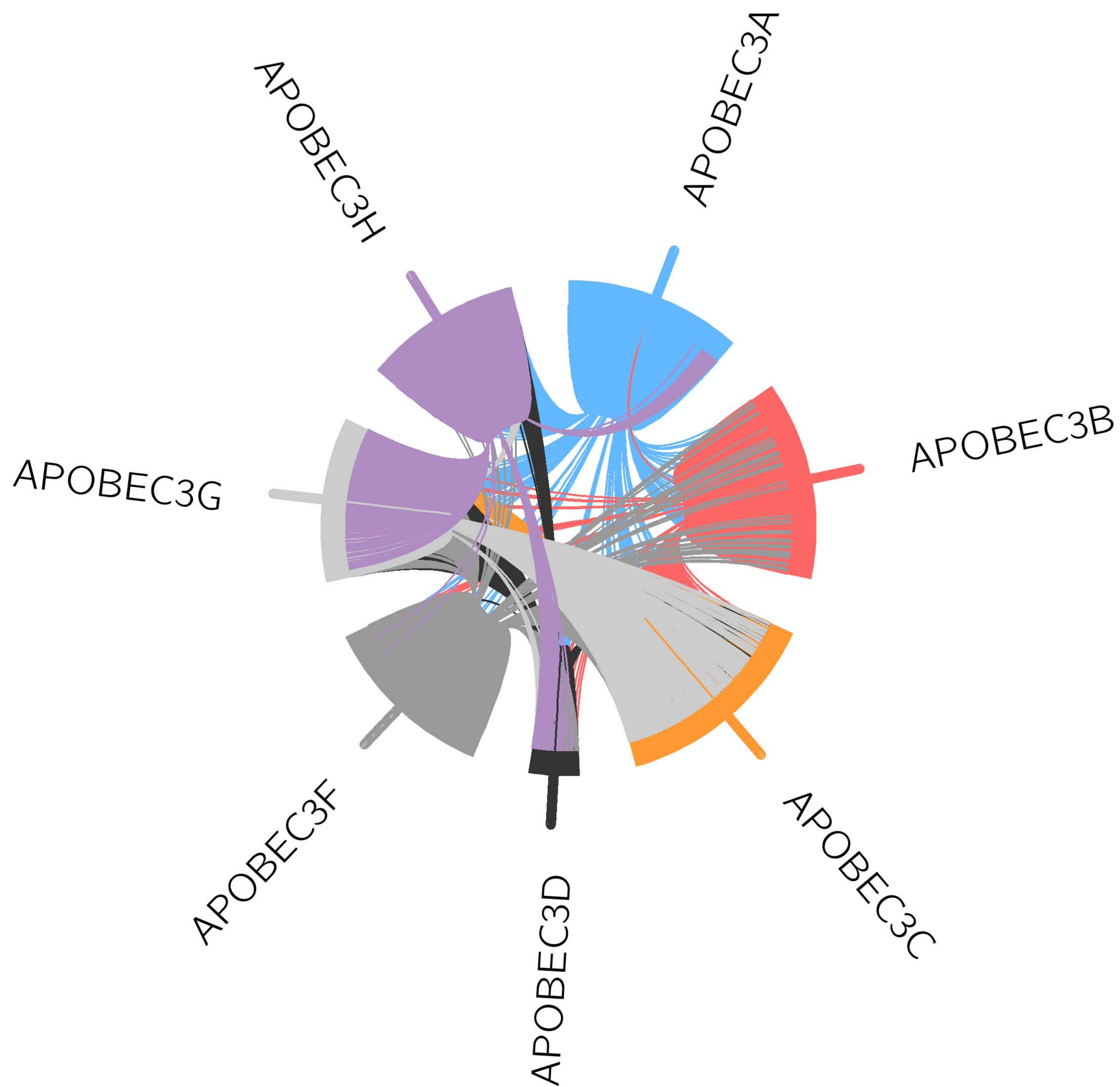

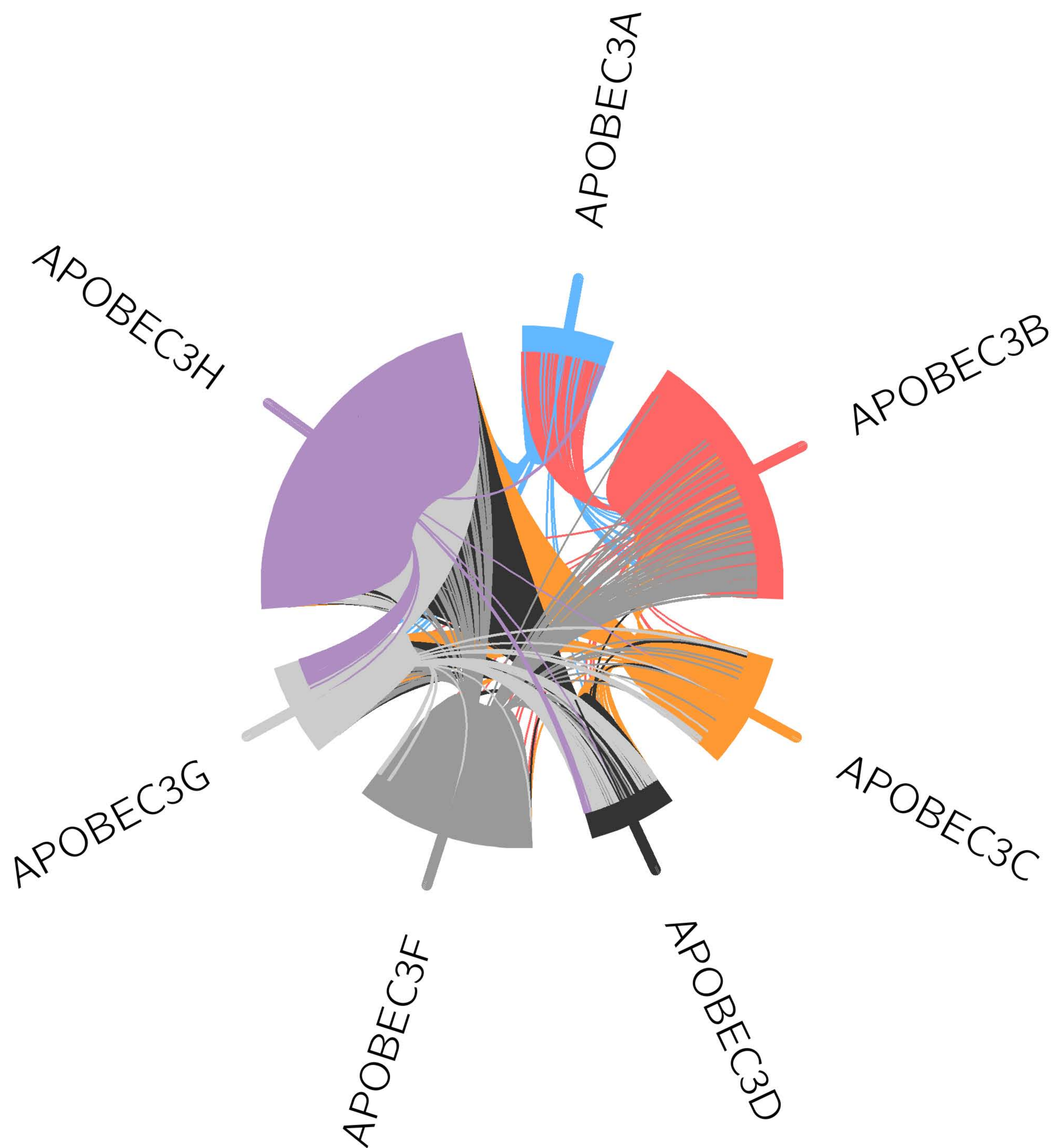

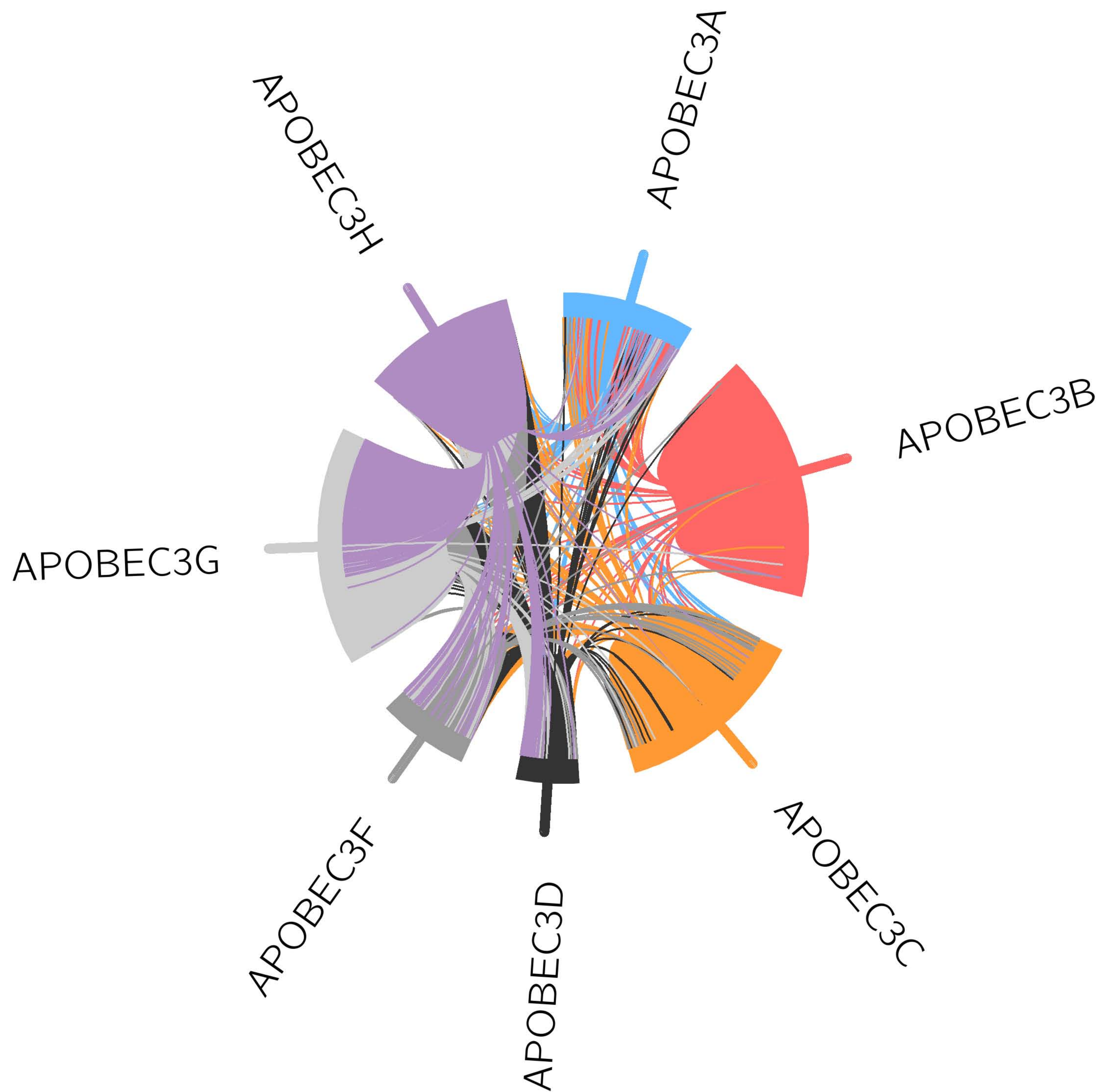

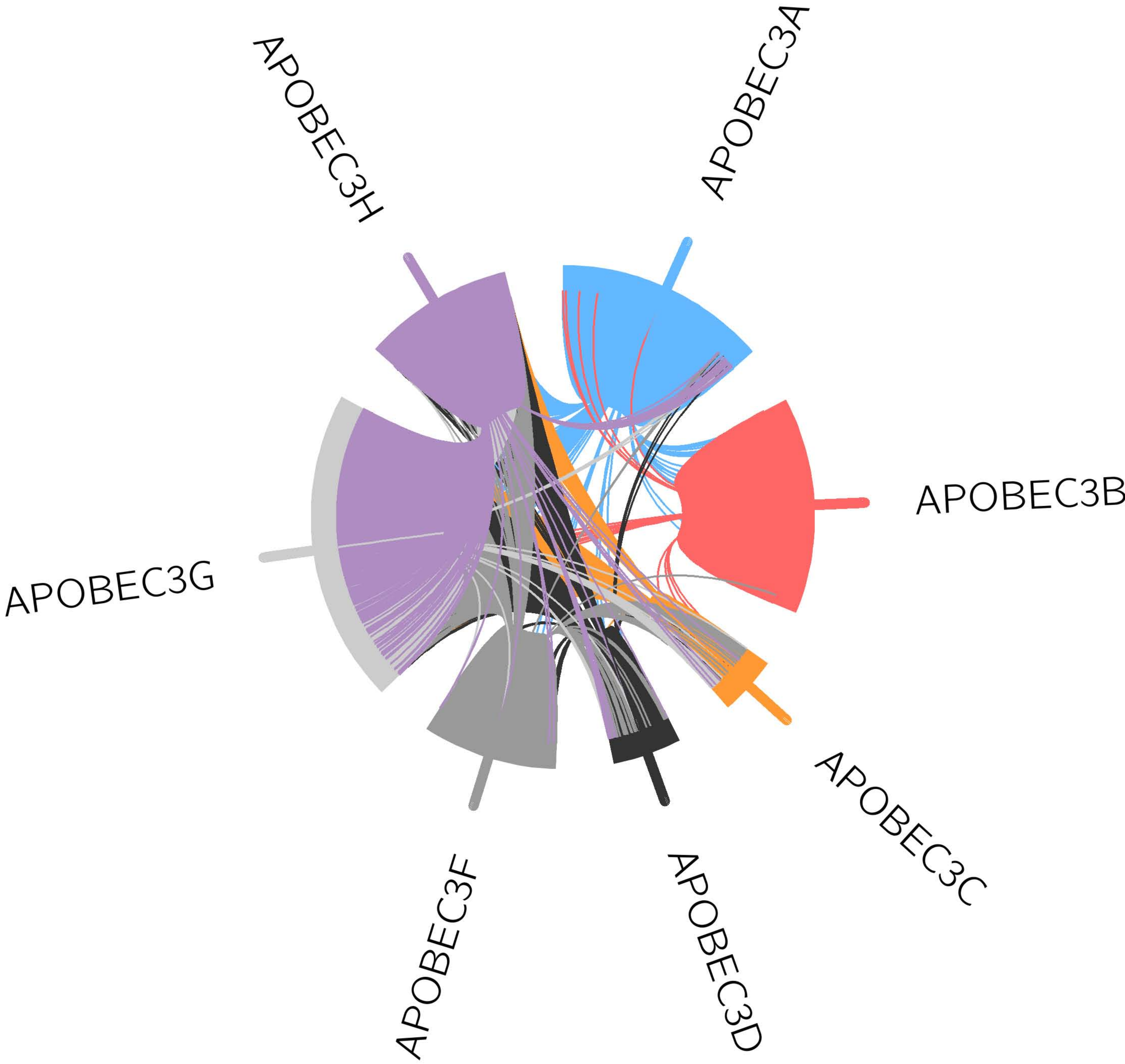

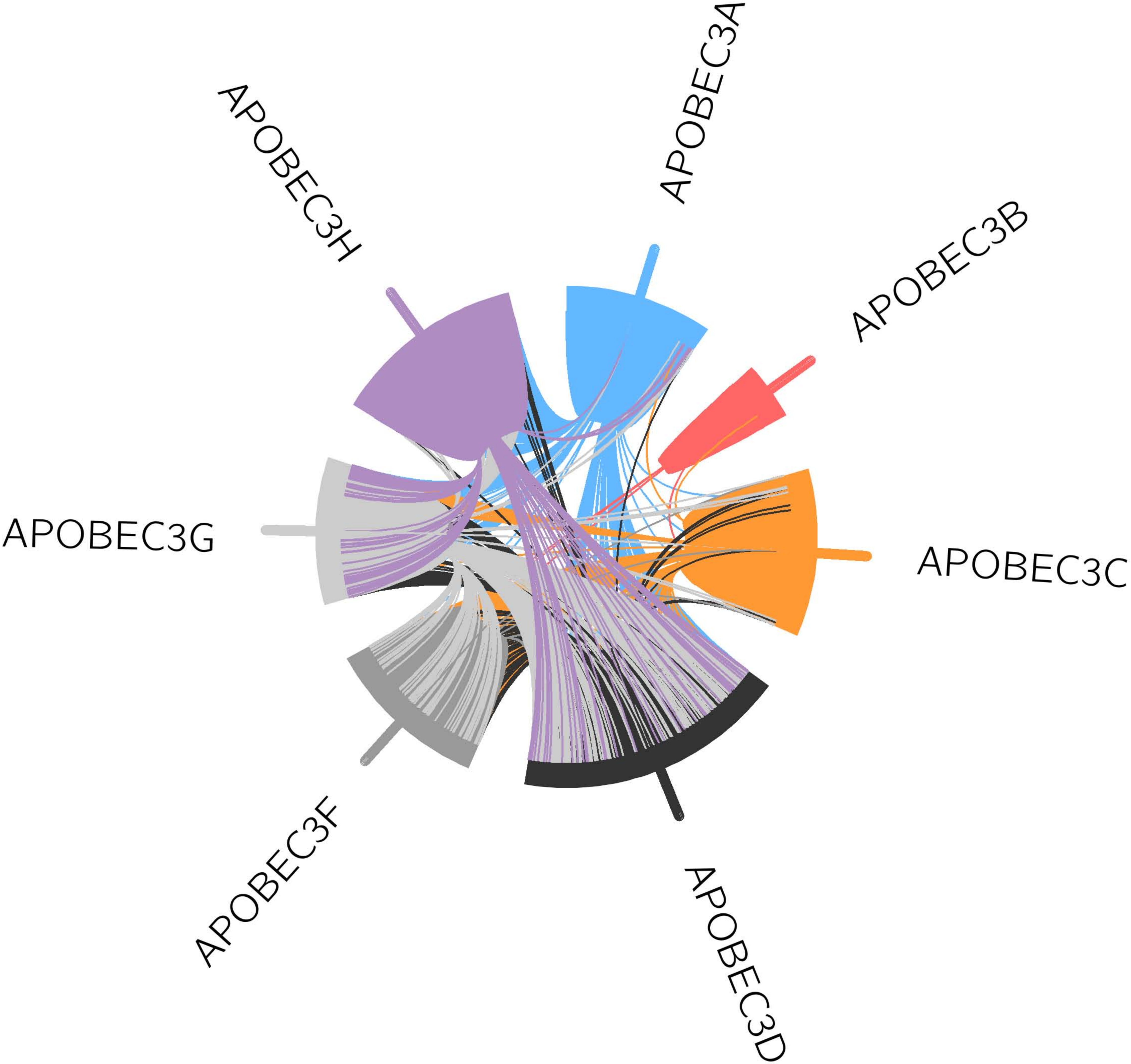

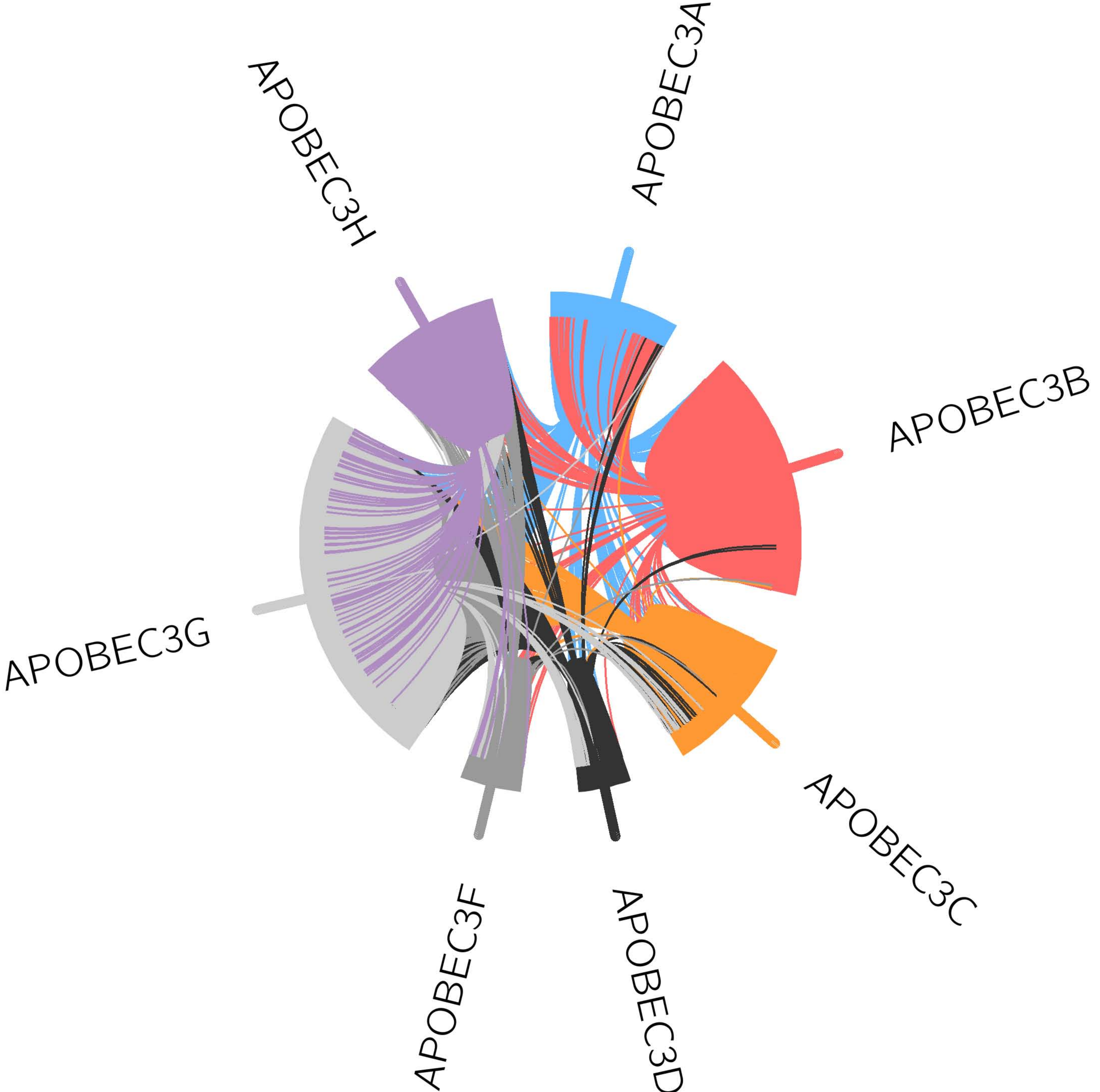

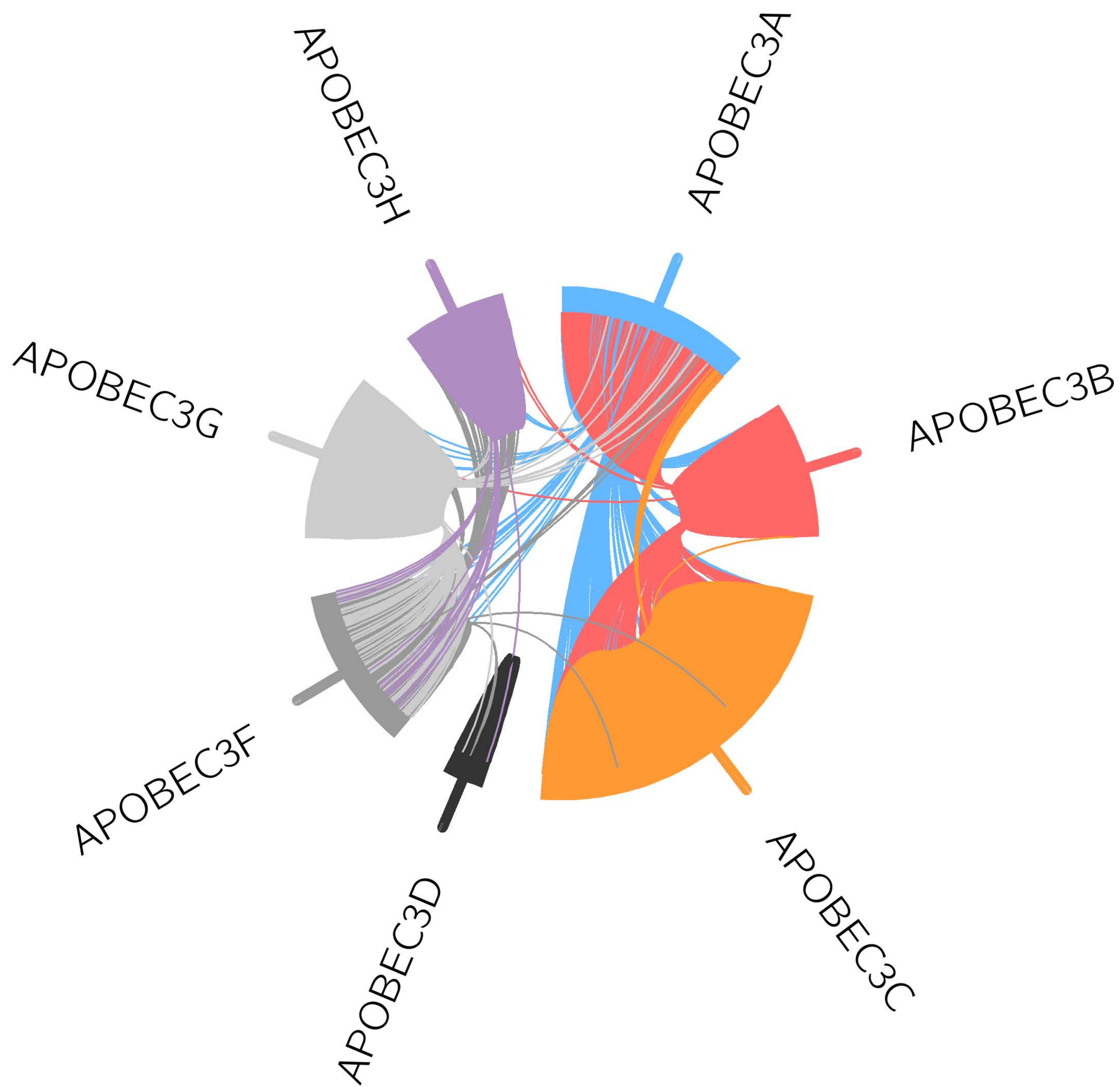

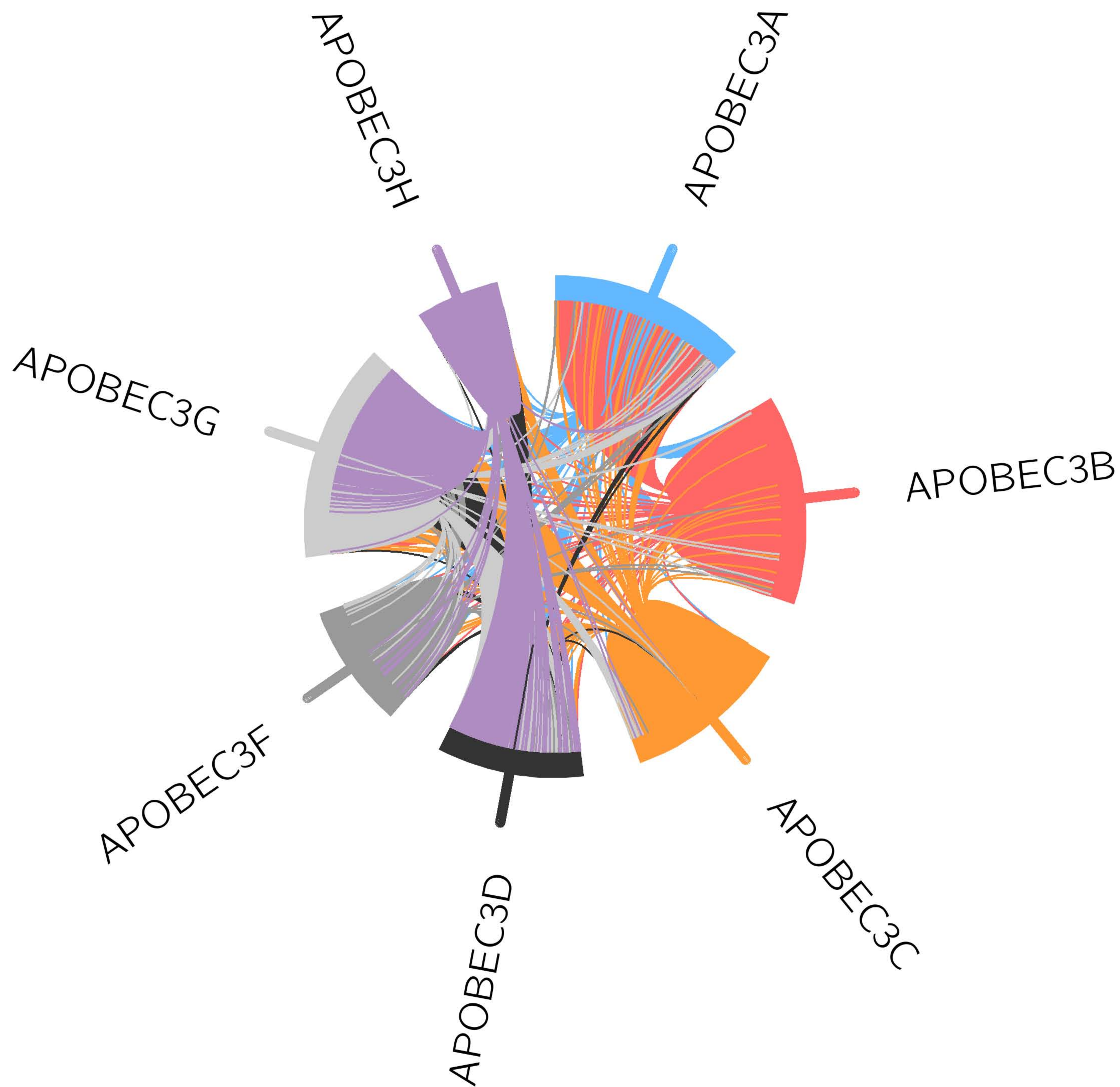

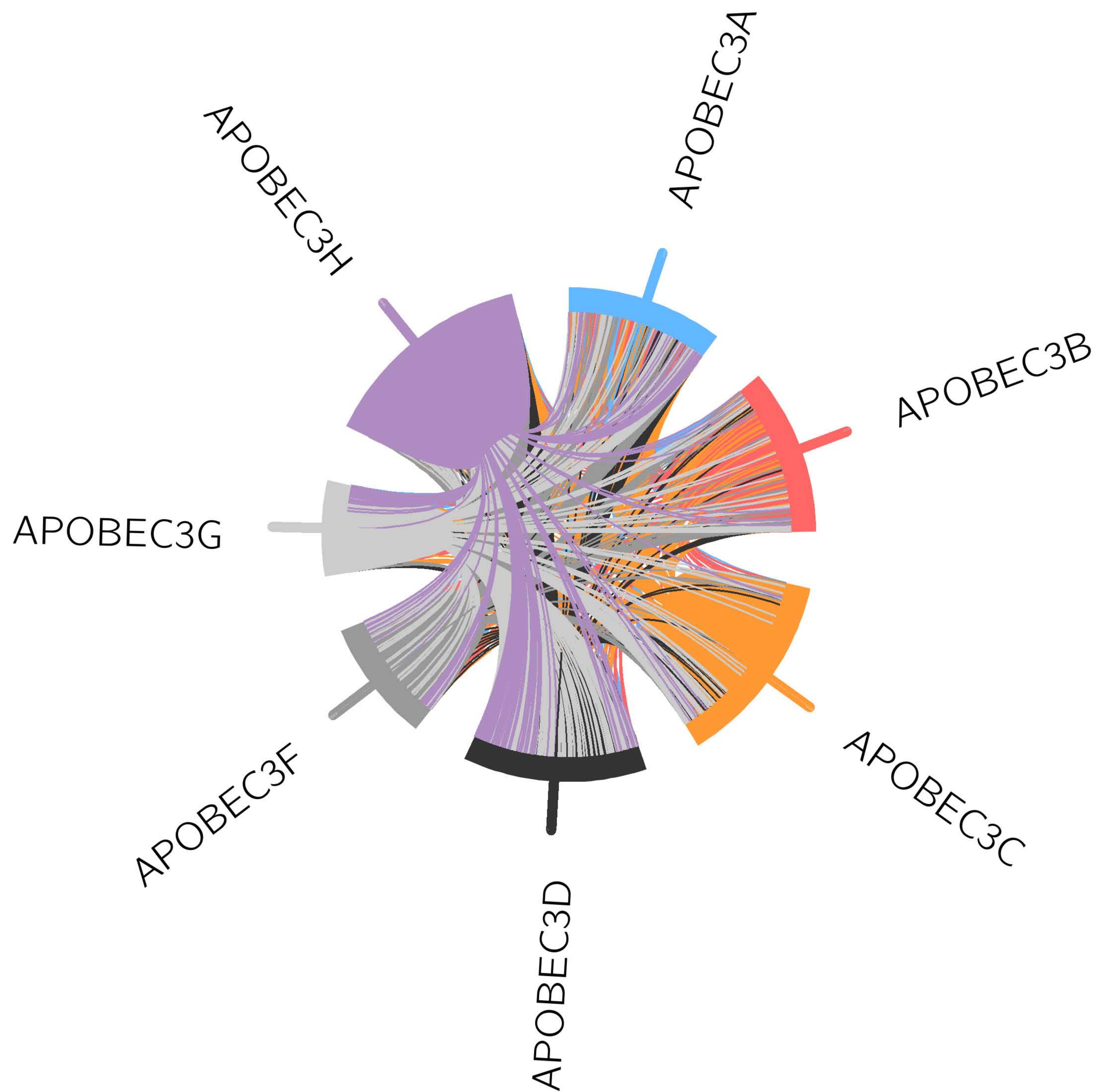

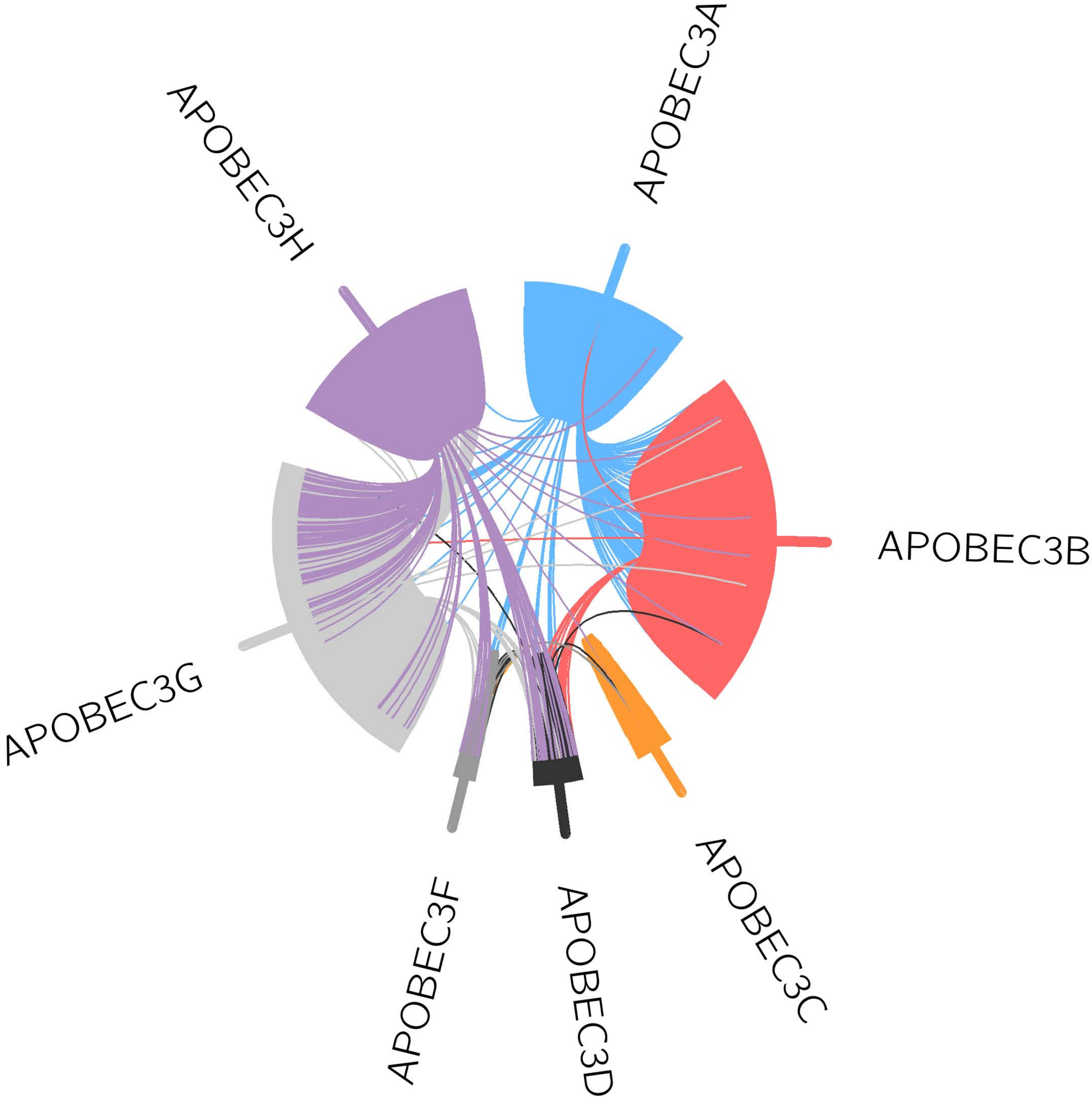

Supplement: Supplementary Data [file gky1316_supplemental_files.zip › FigureS13_CircosPlots.pdf]

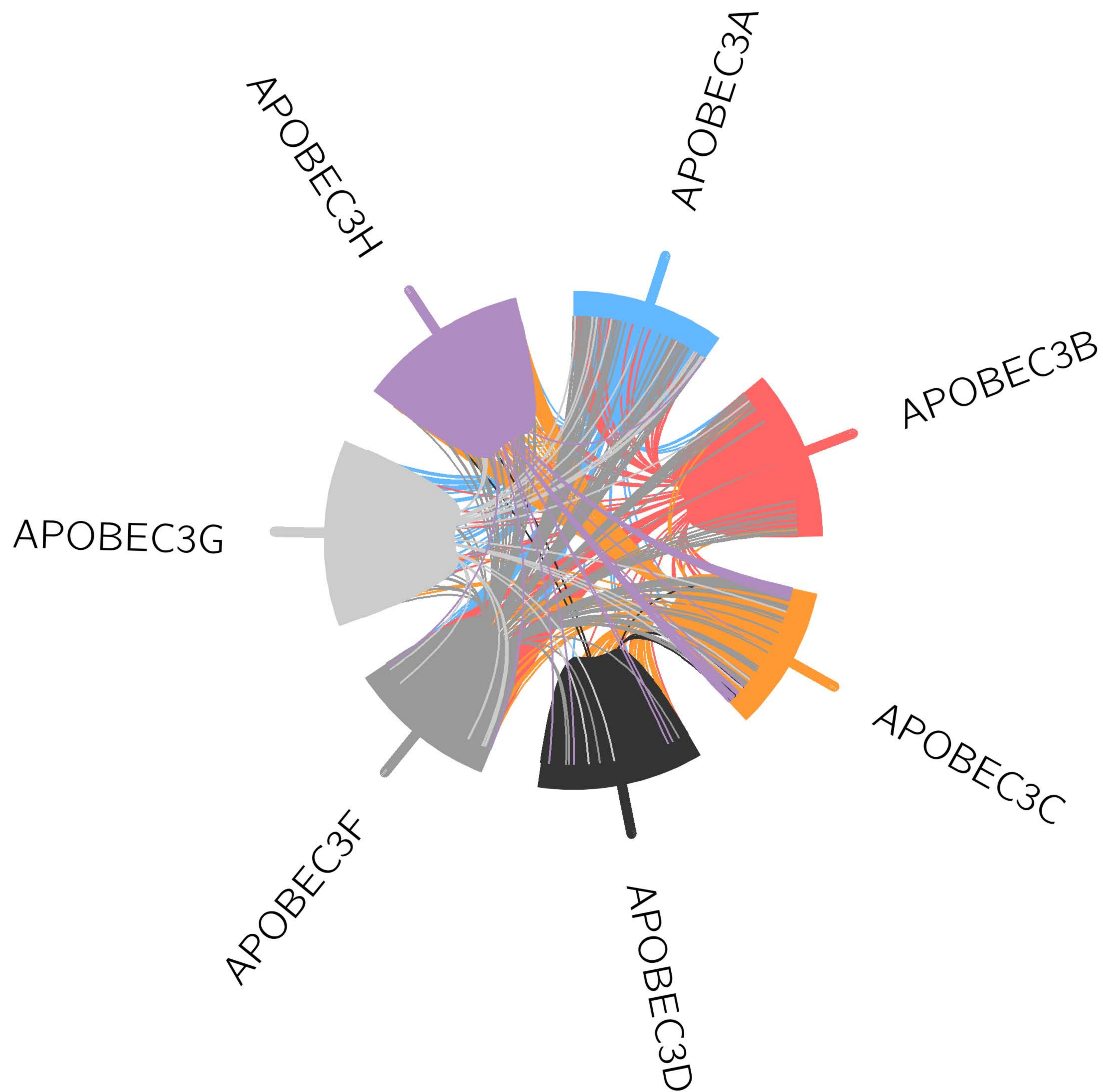

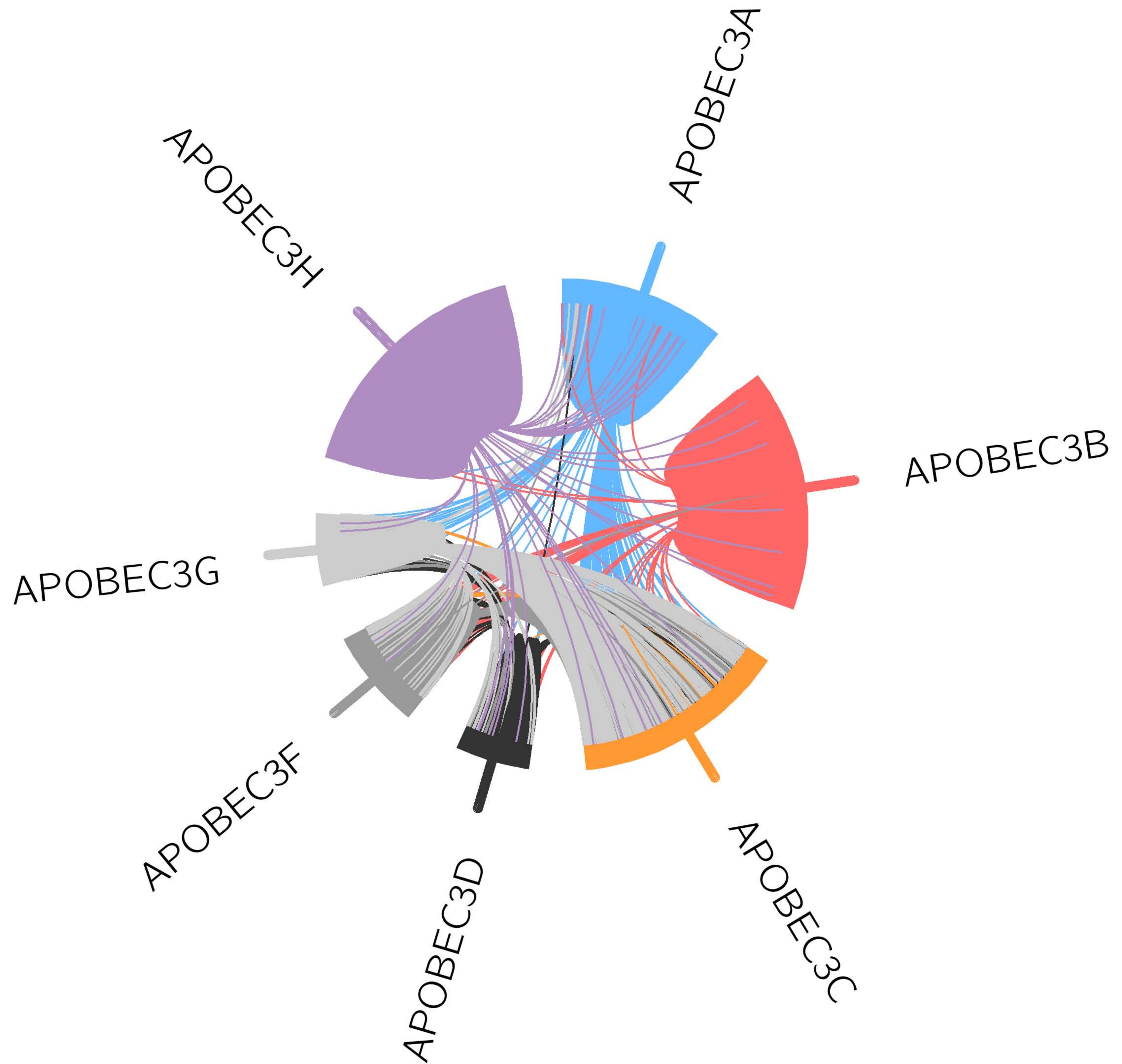

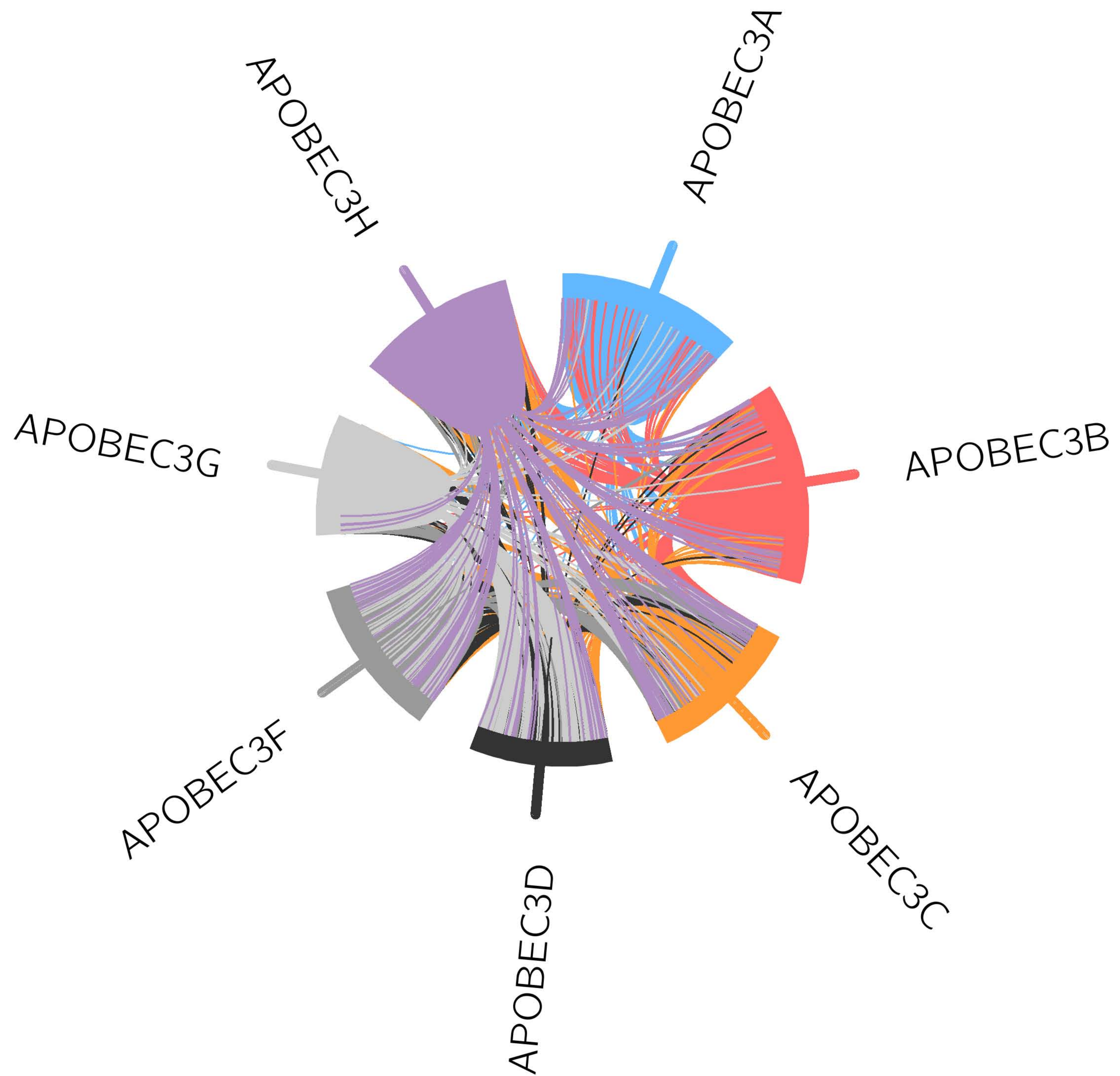

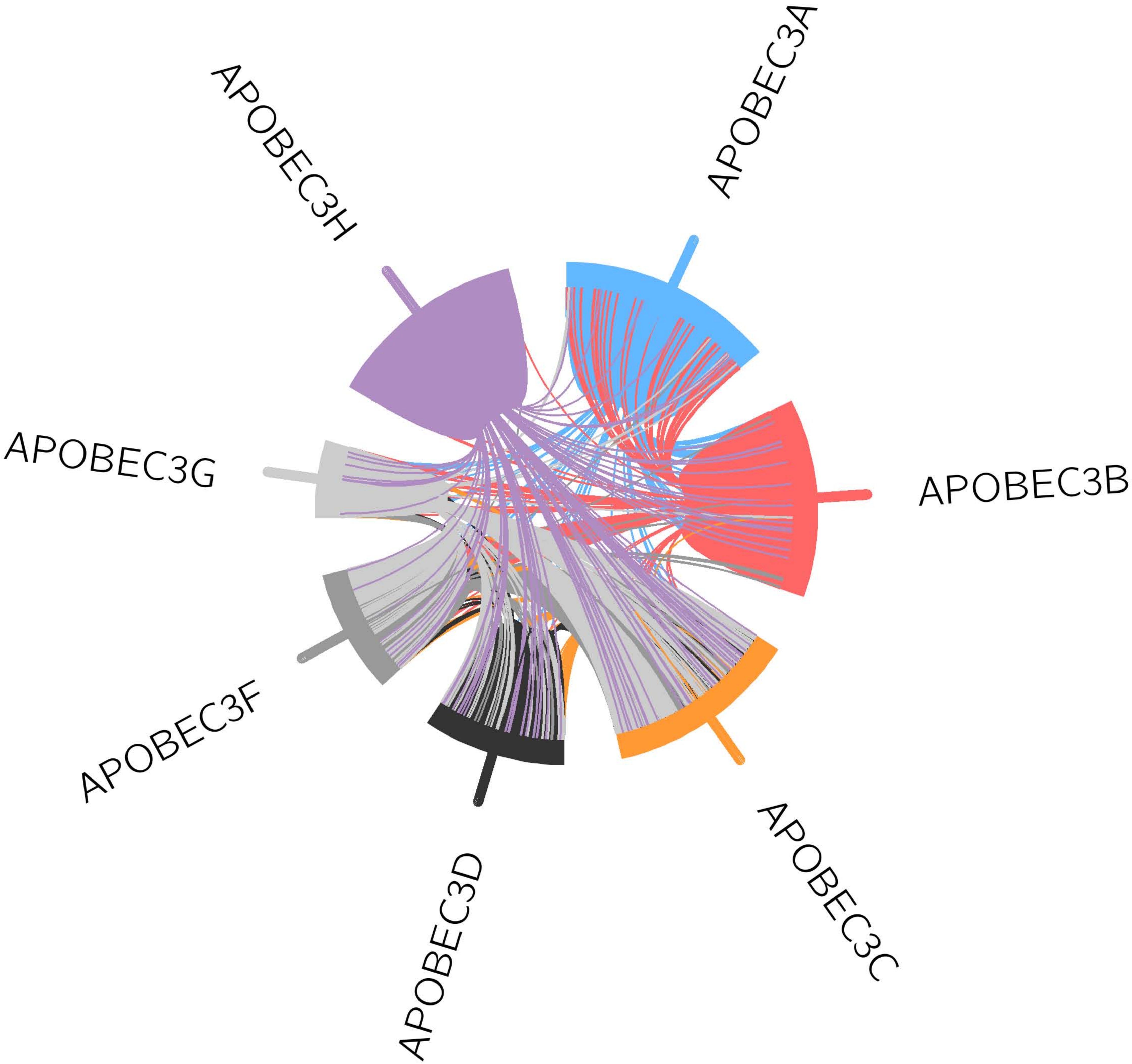

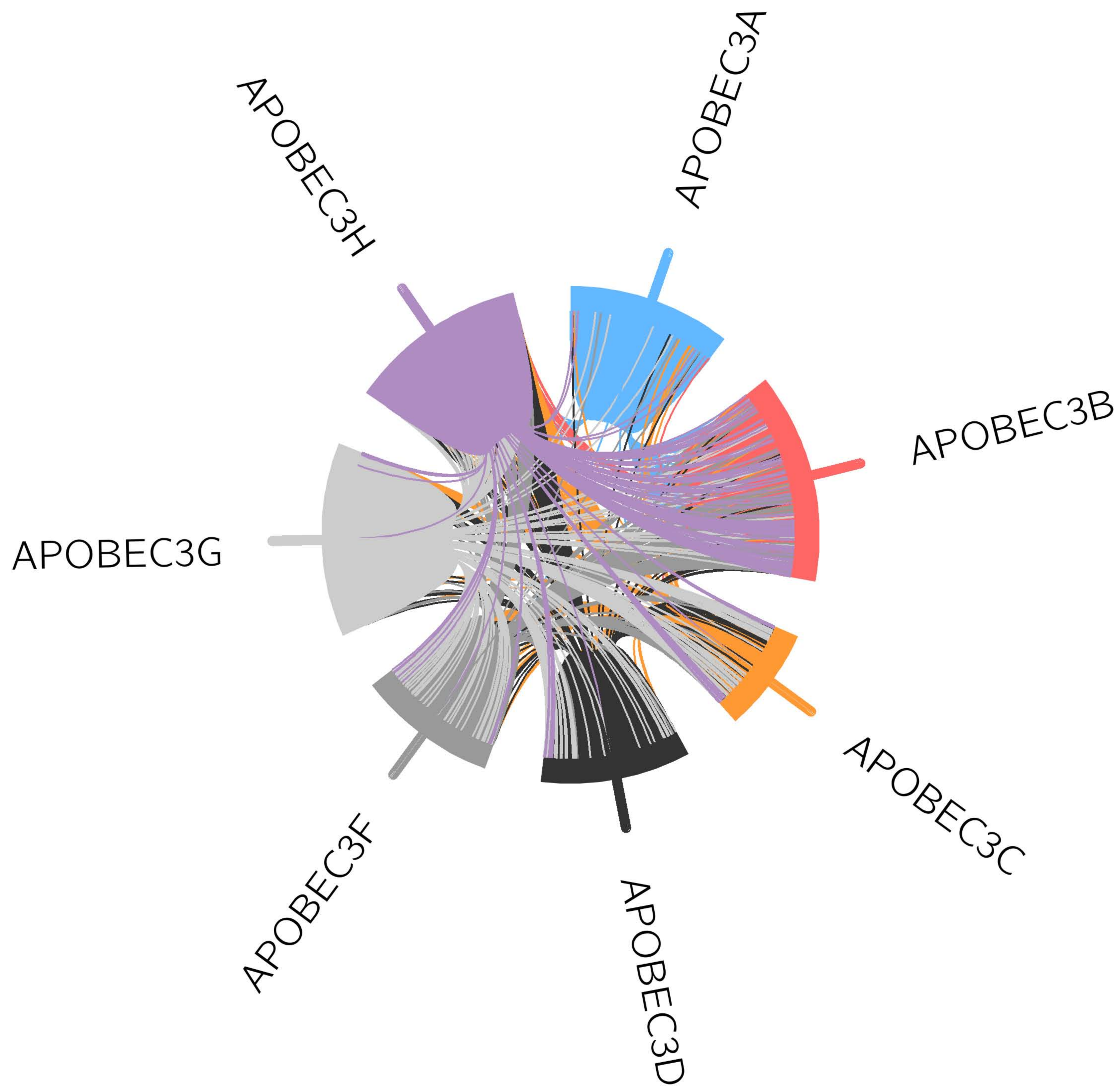

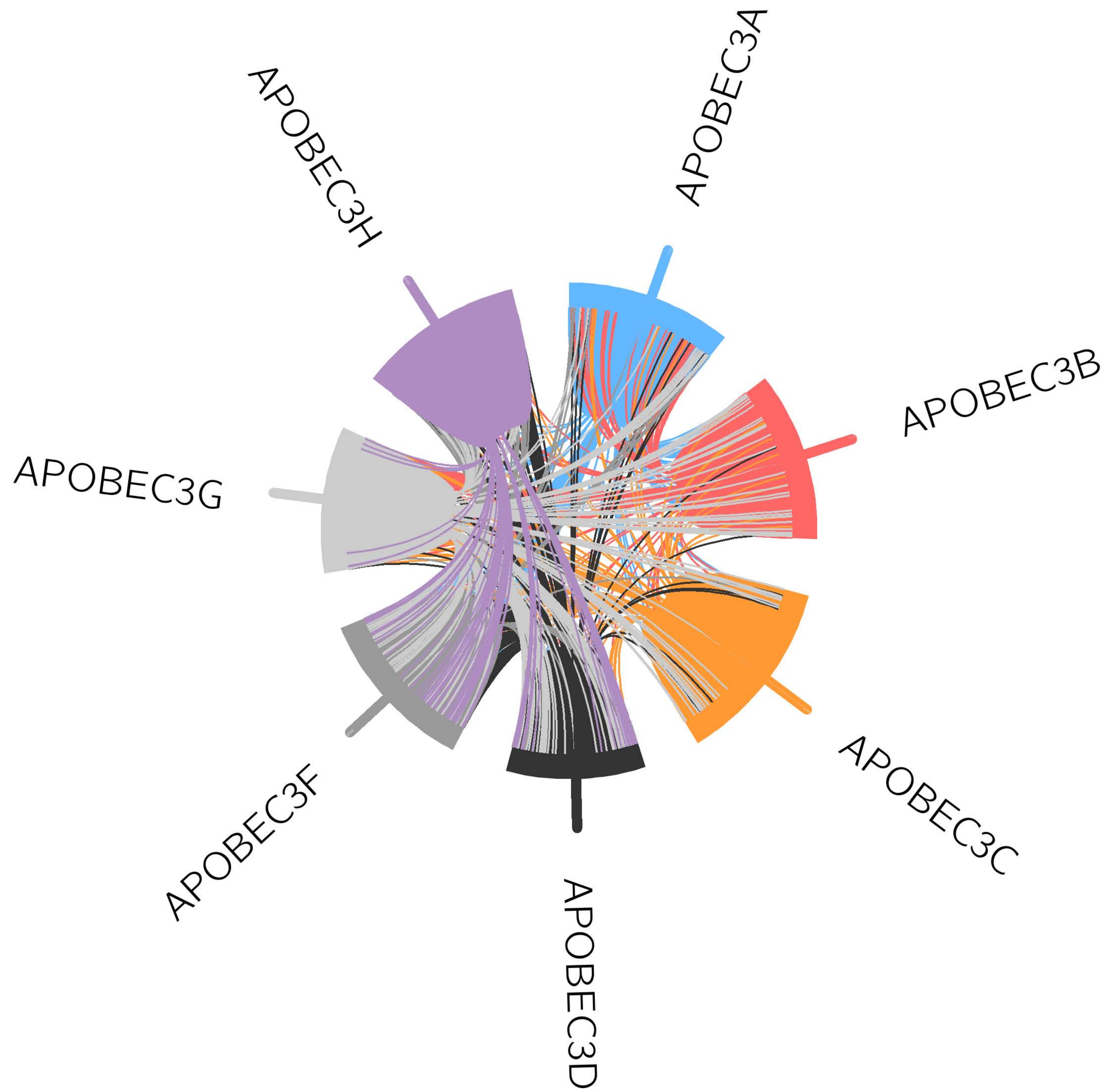

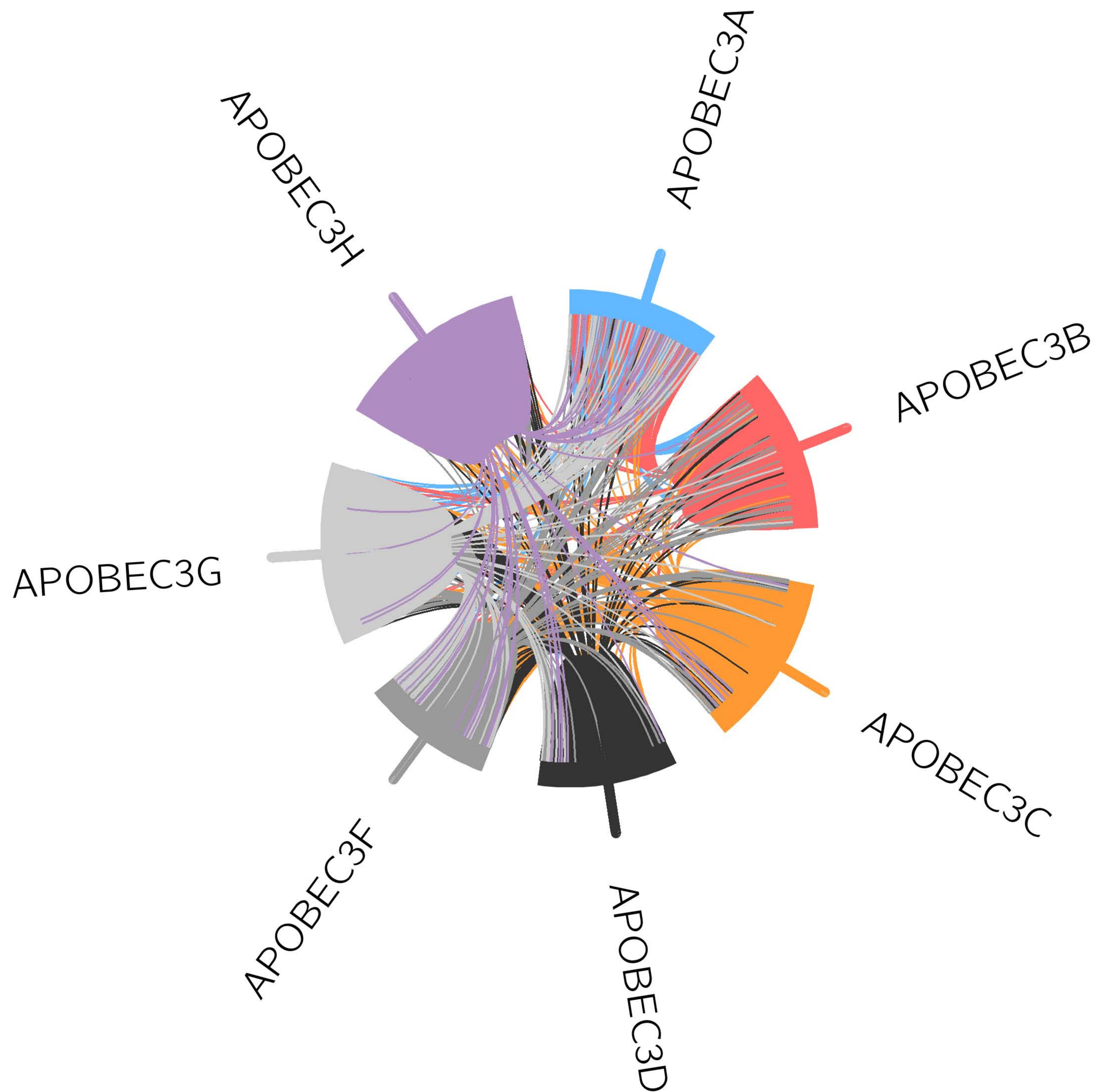

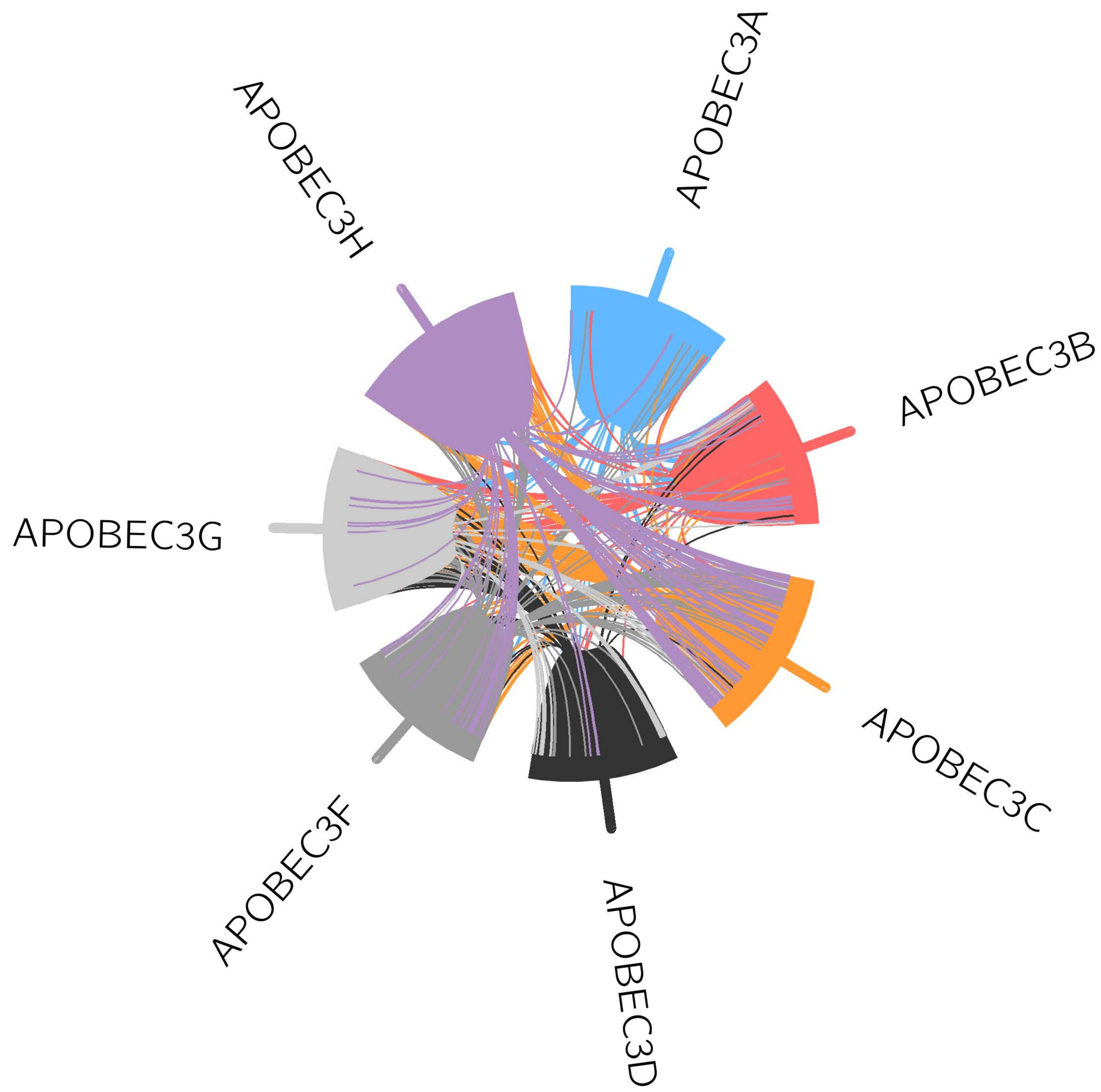

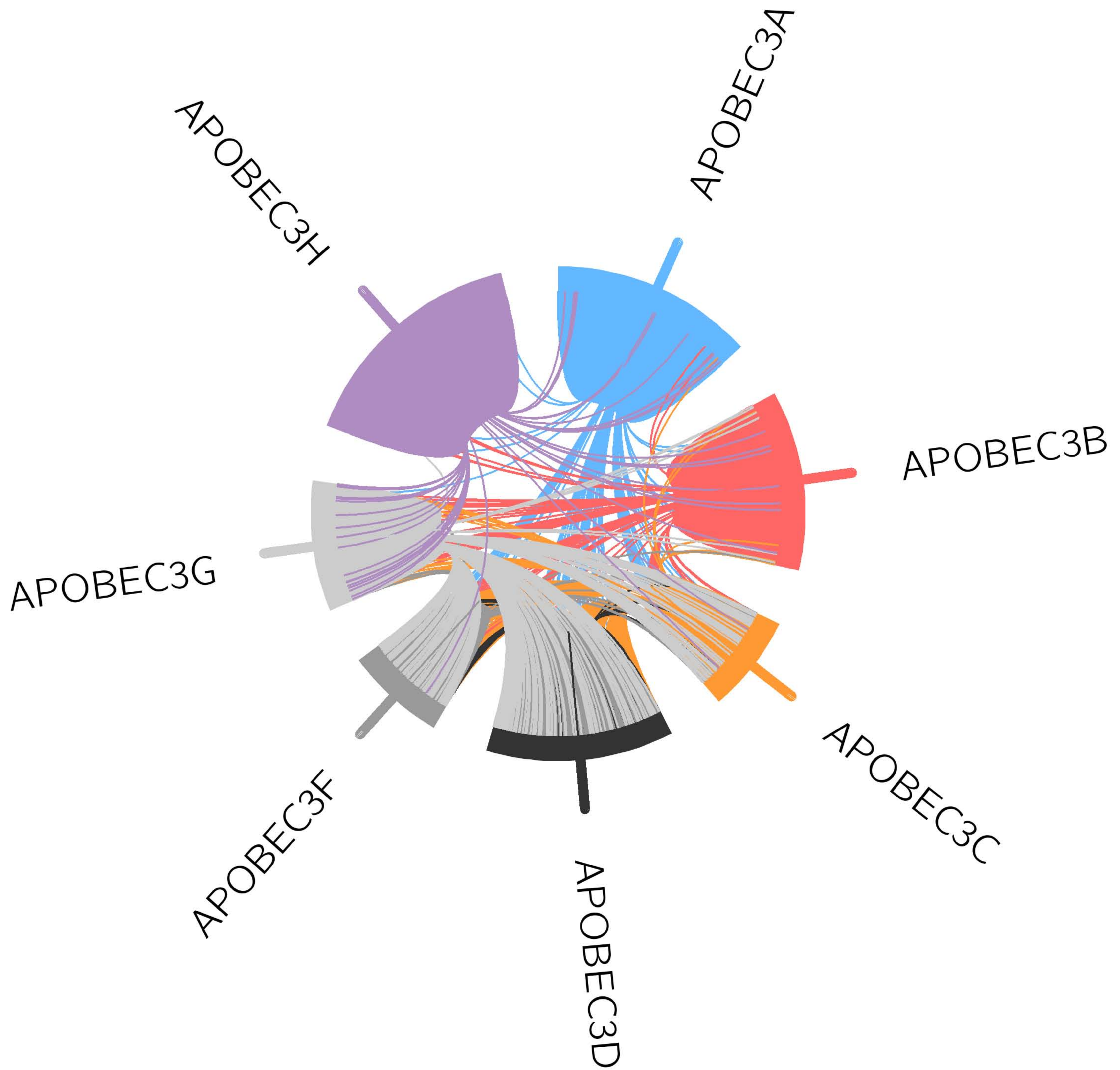

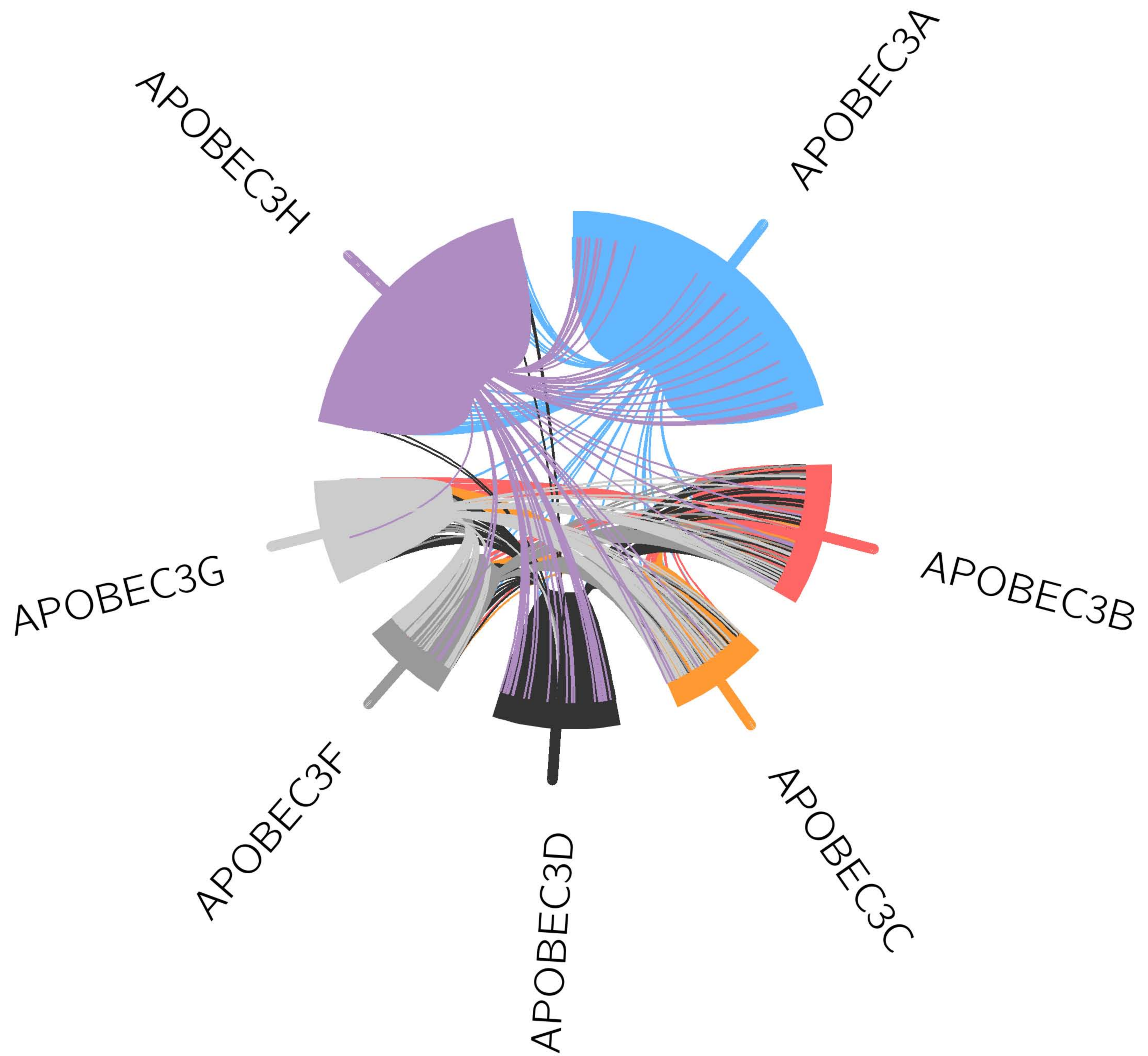

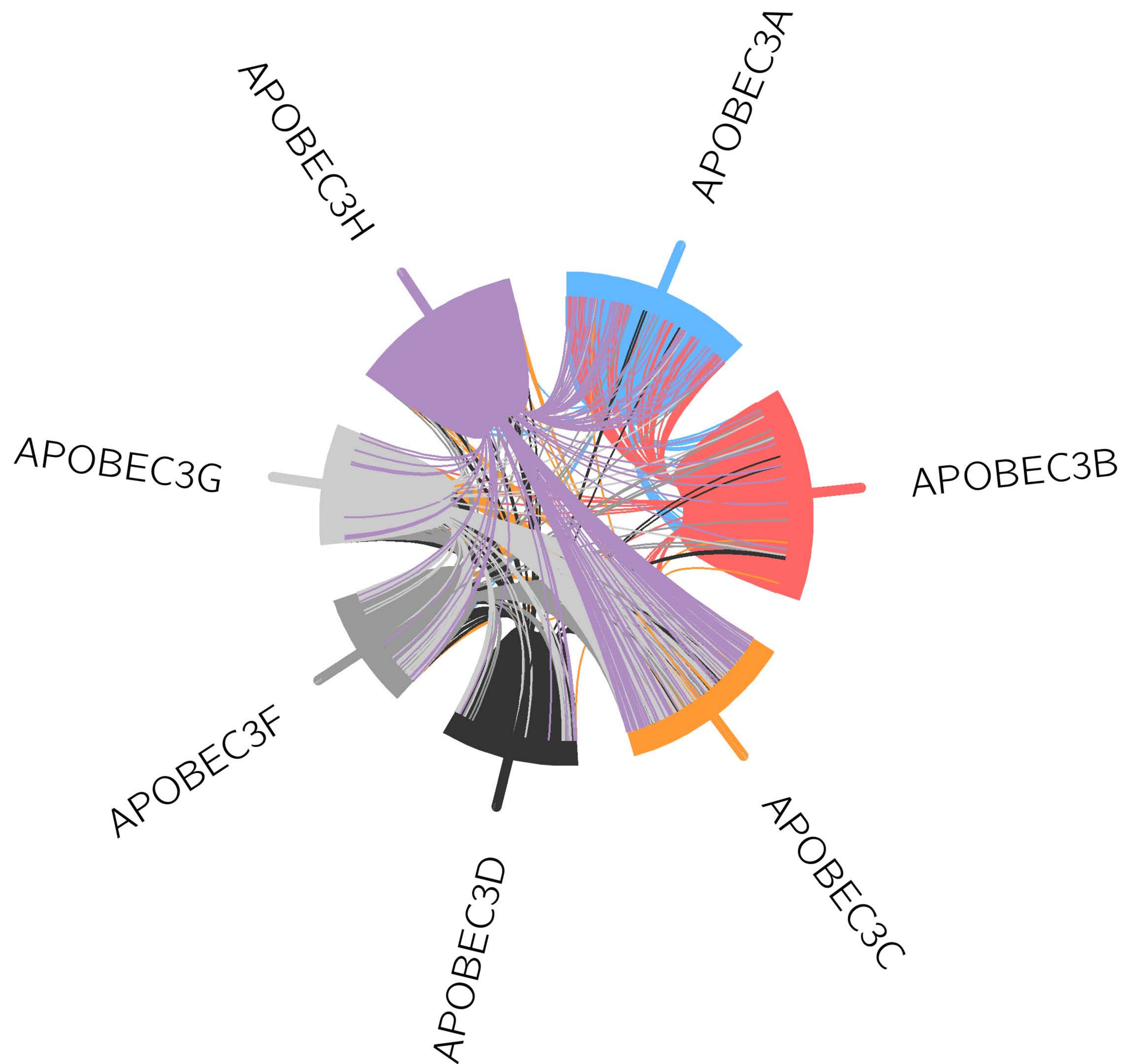

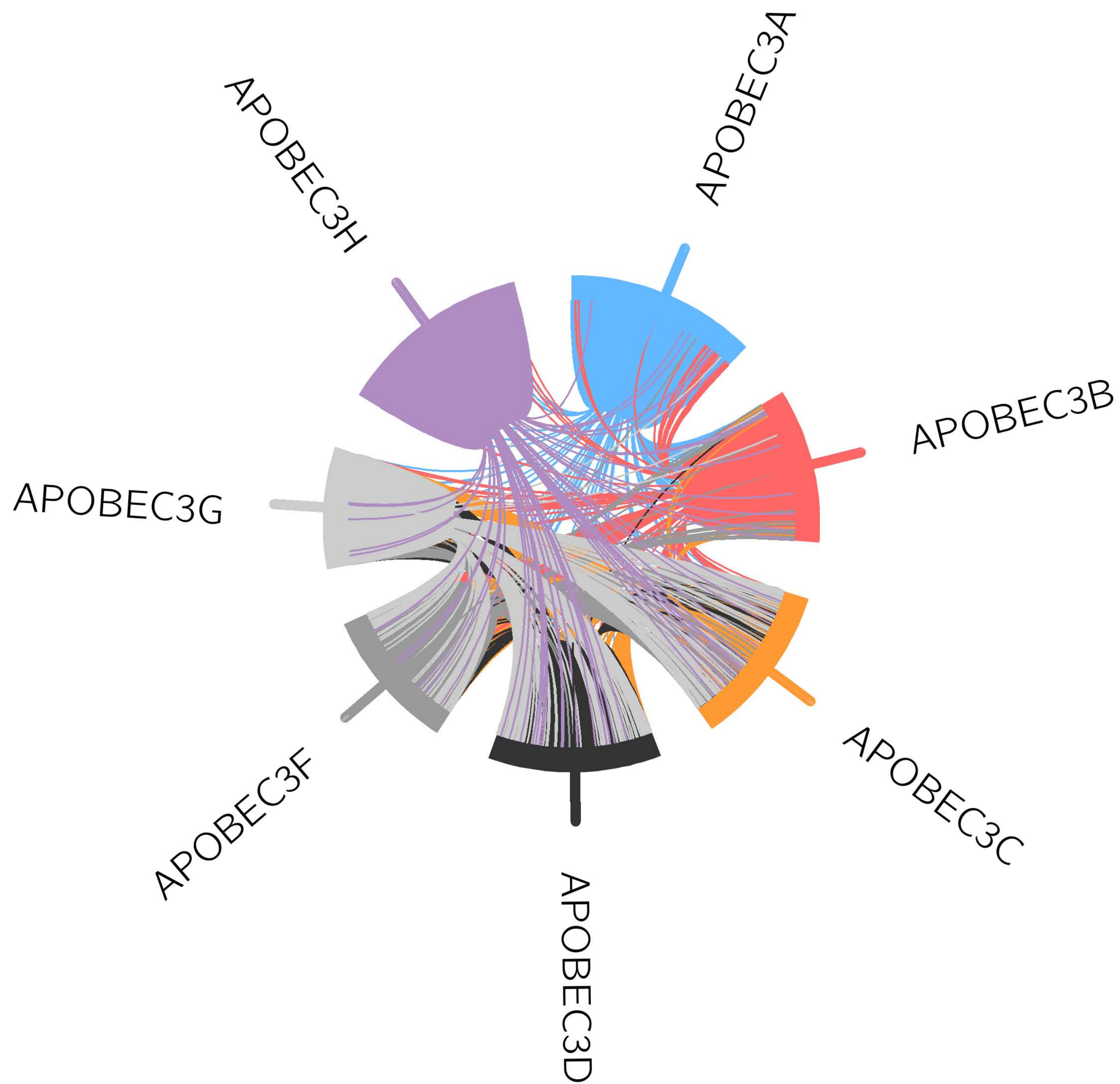

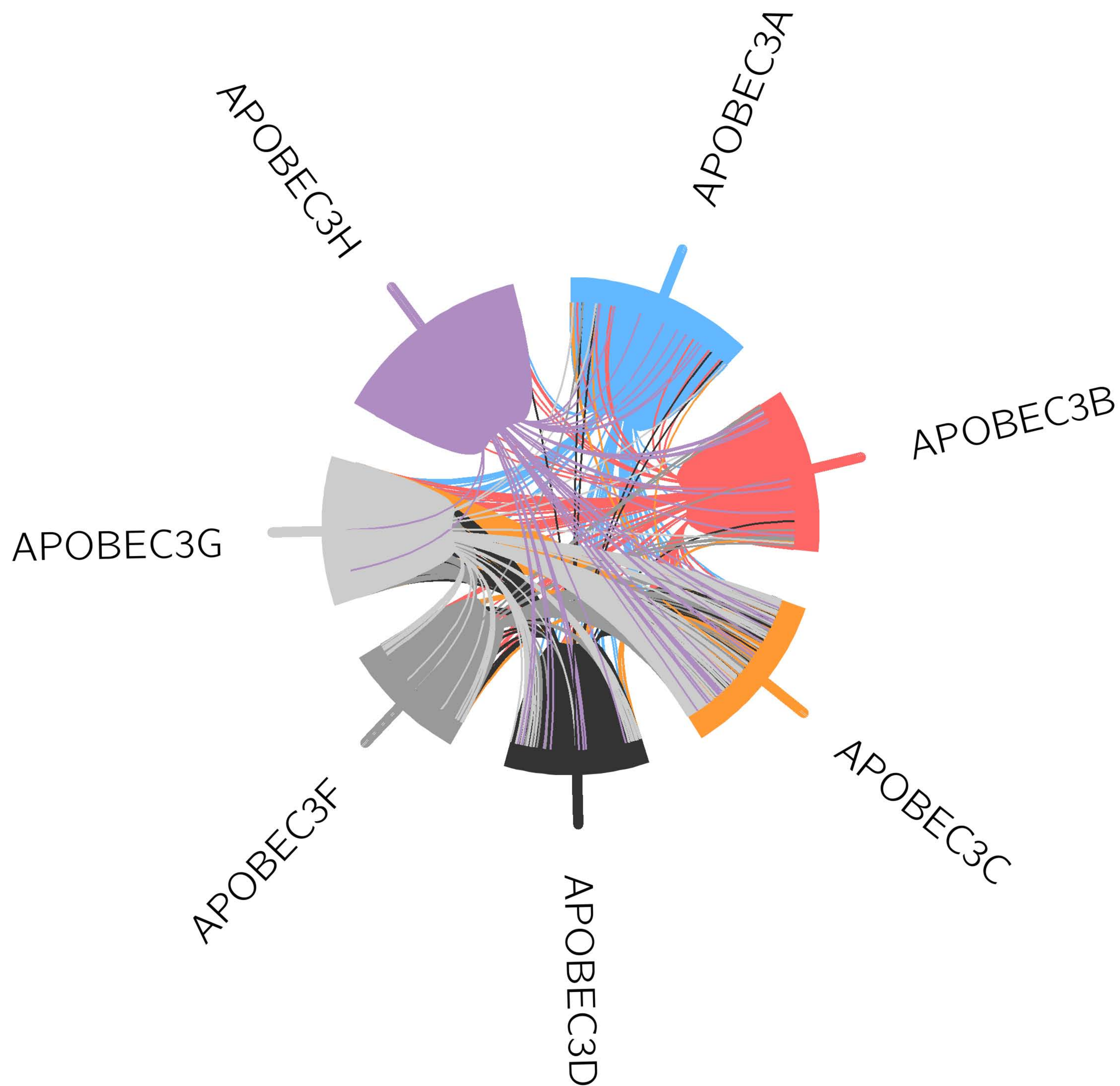

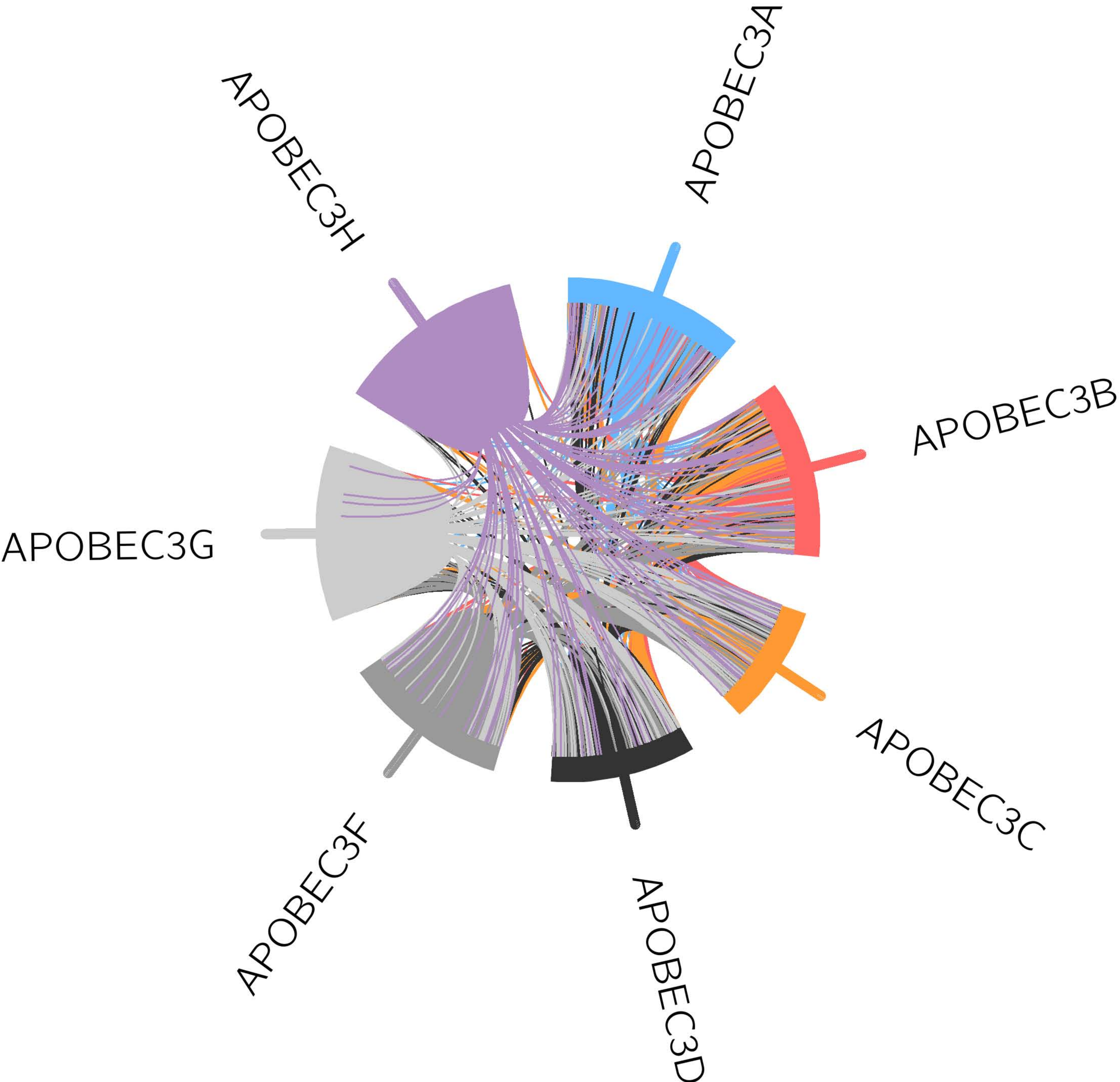

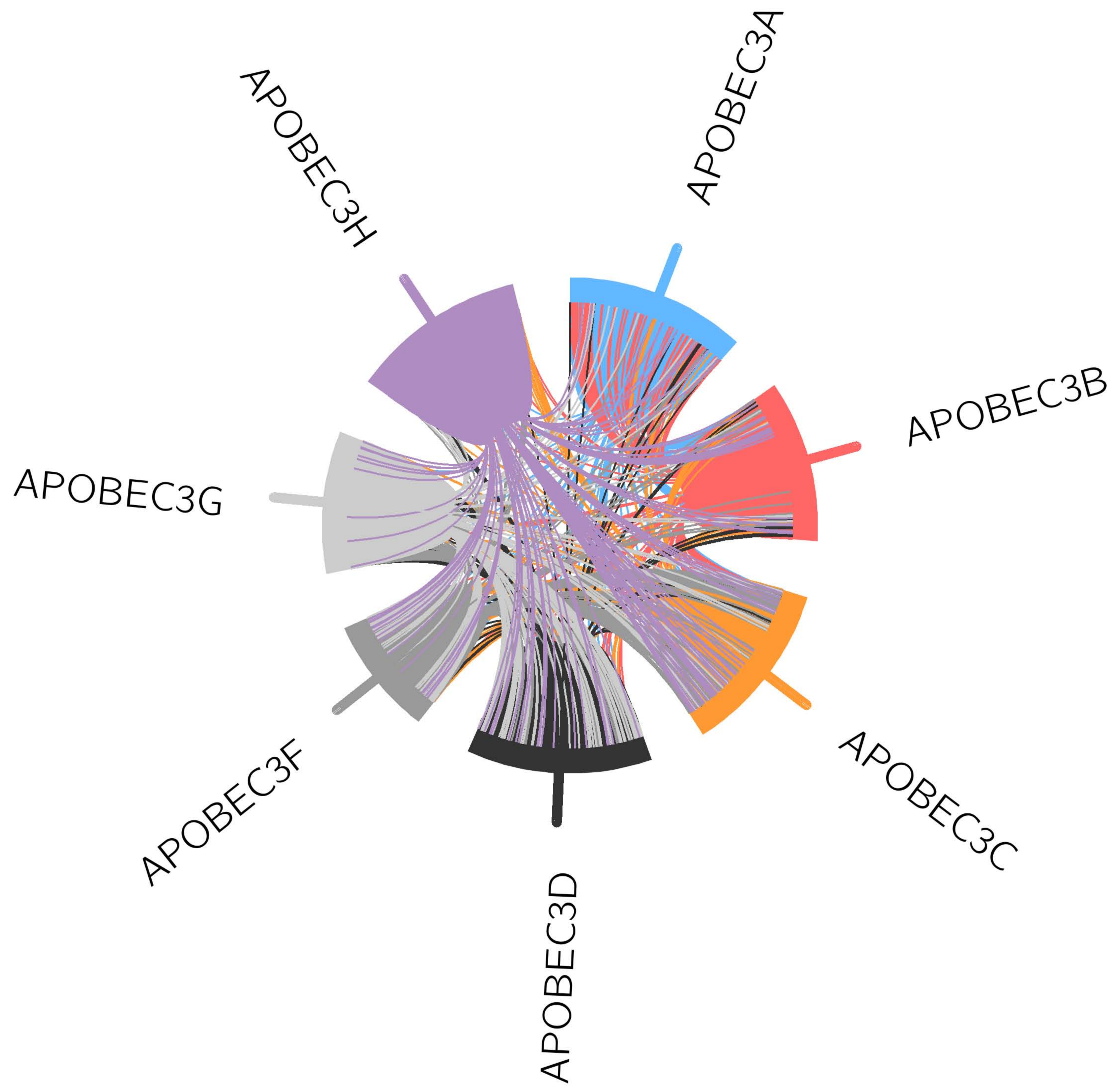

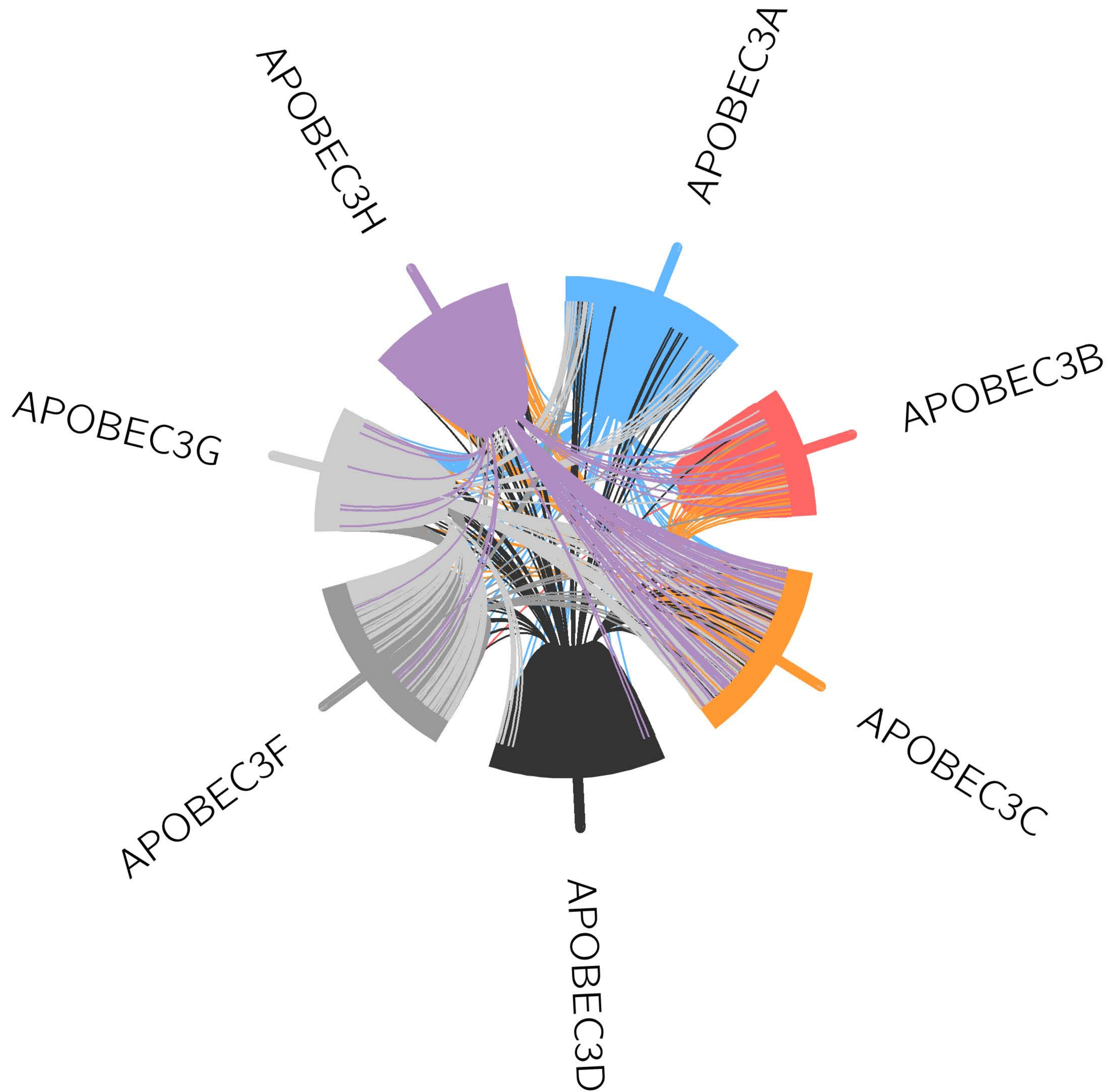

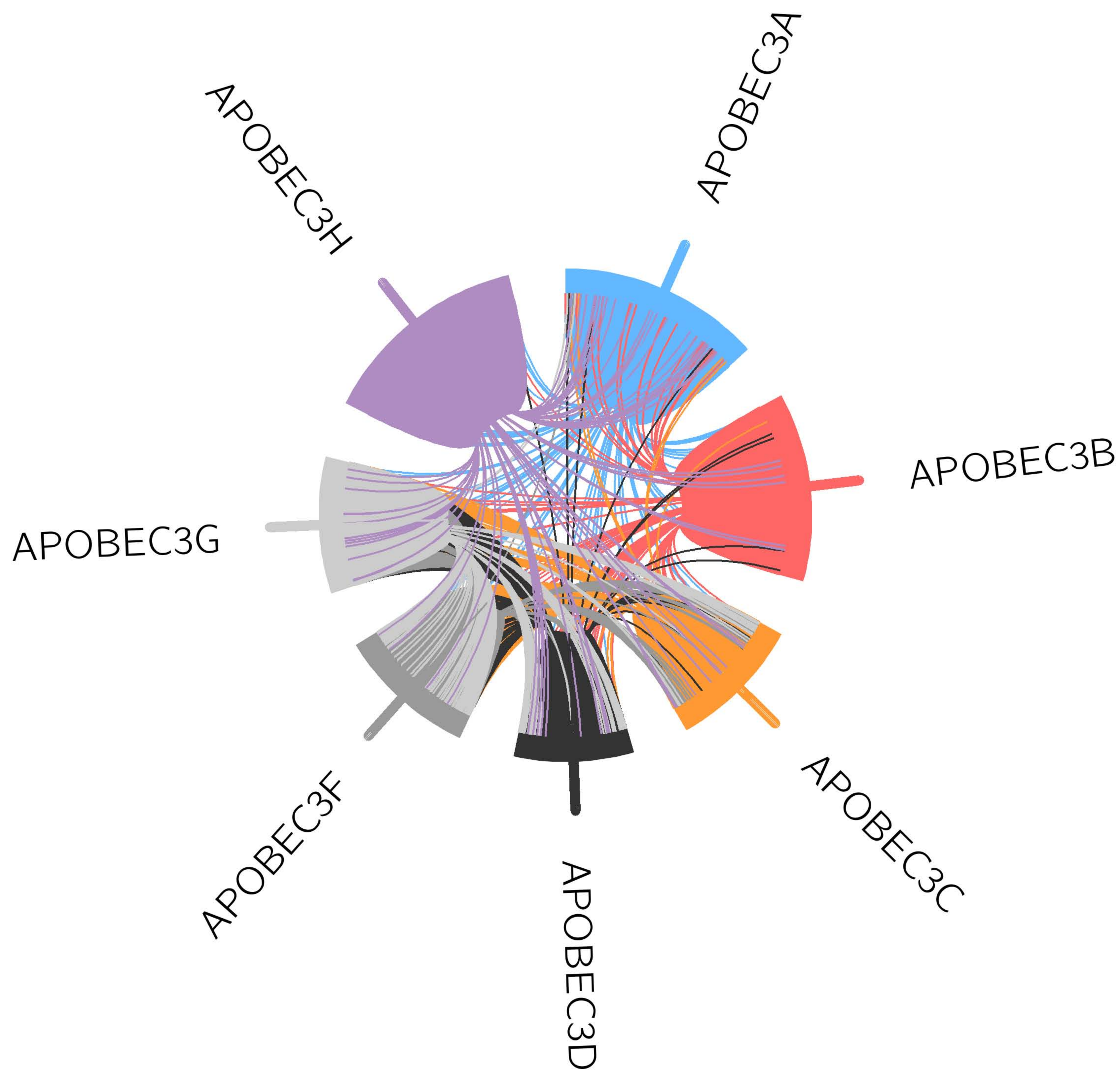

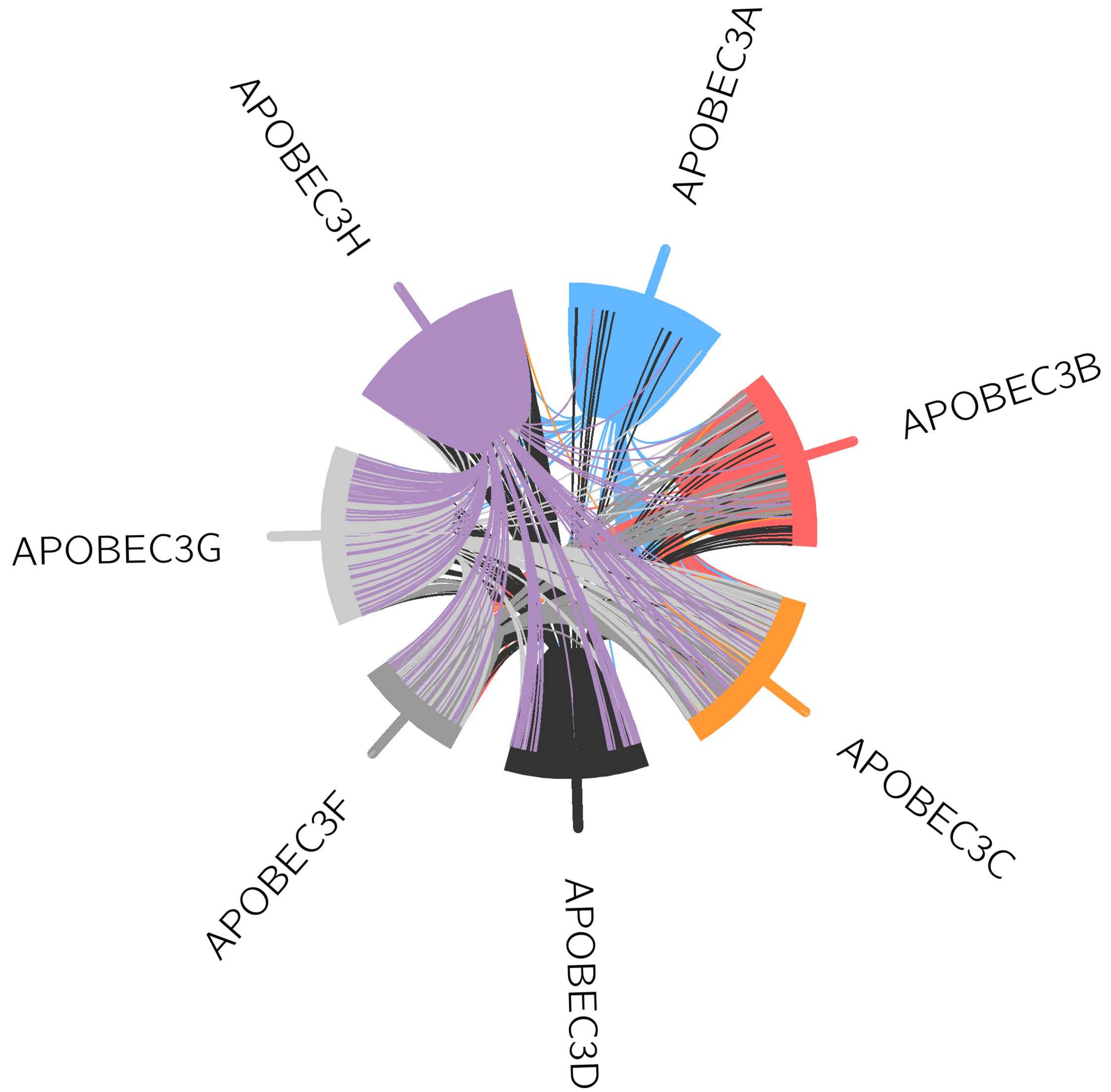

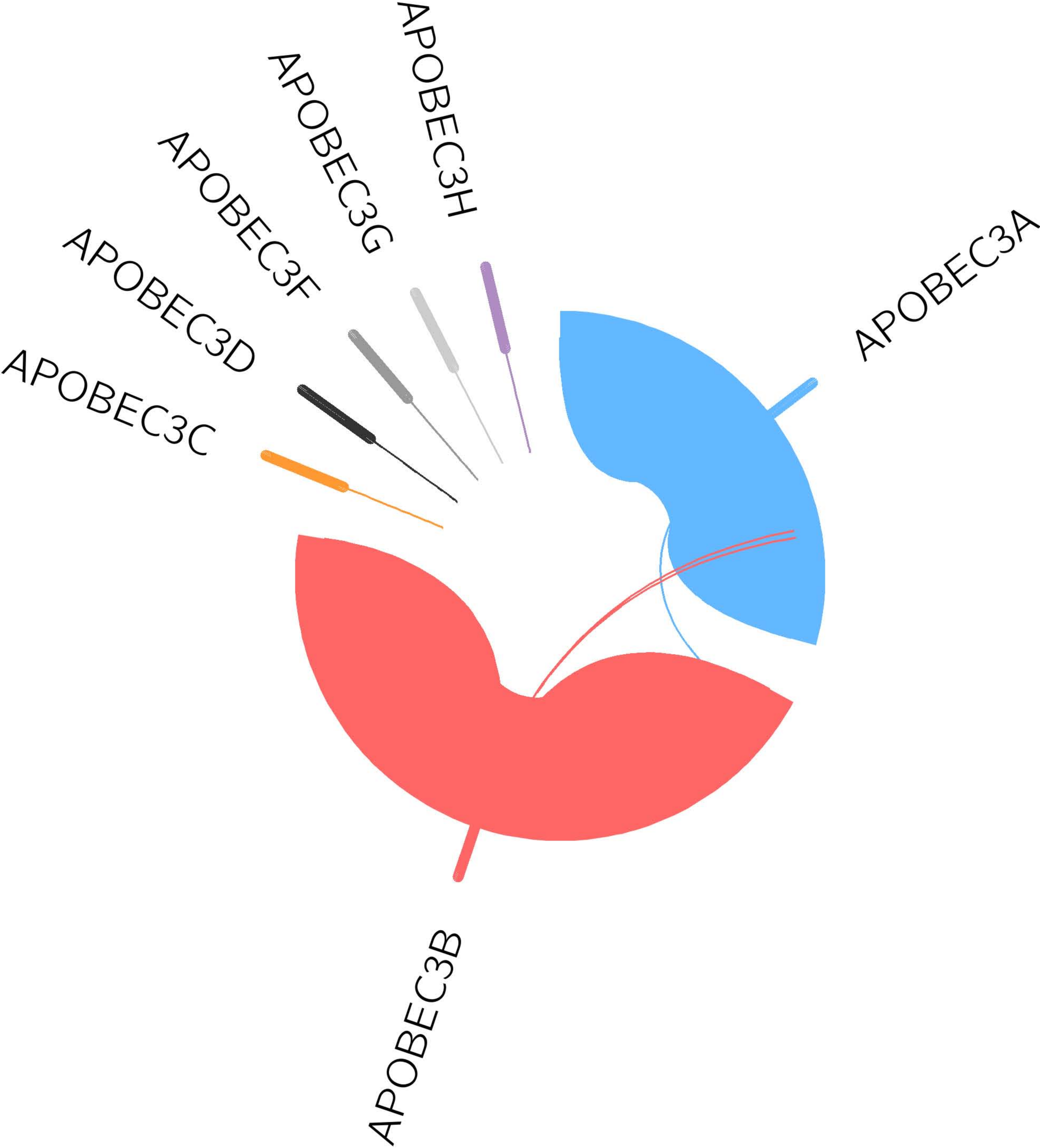

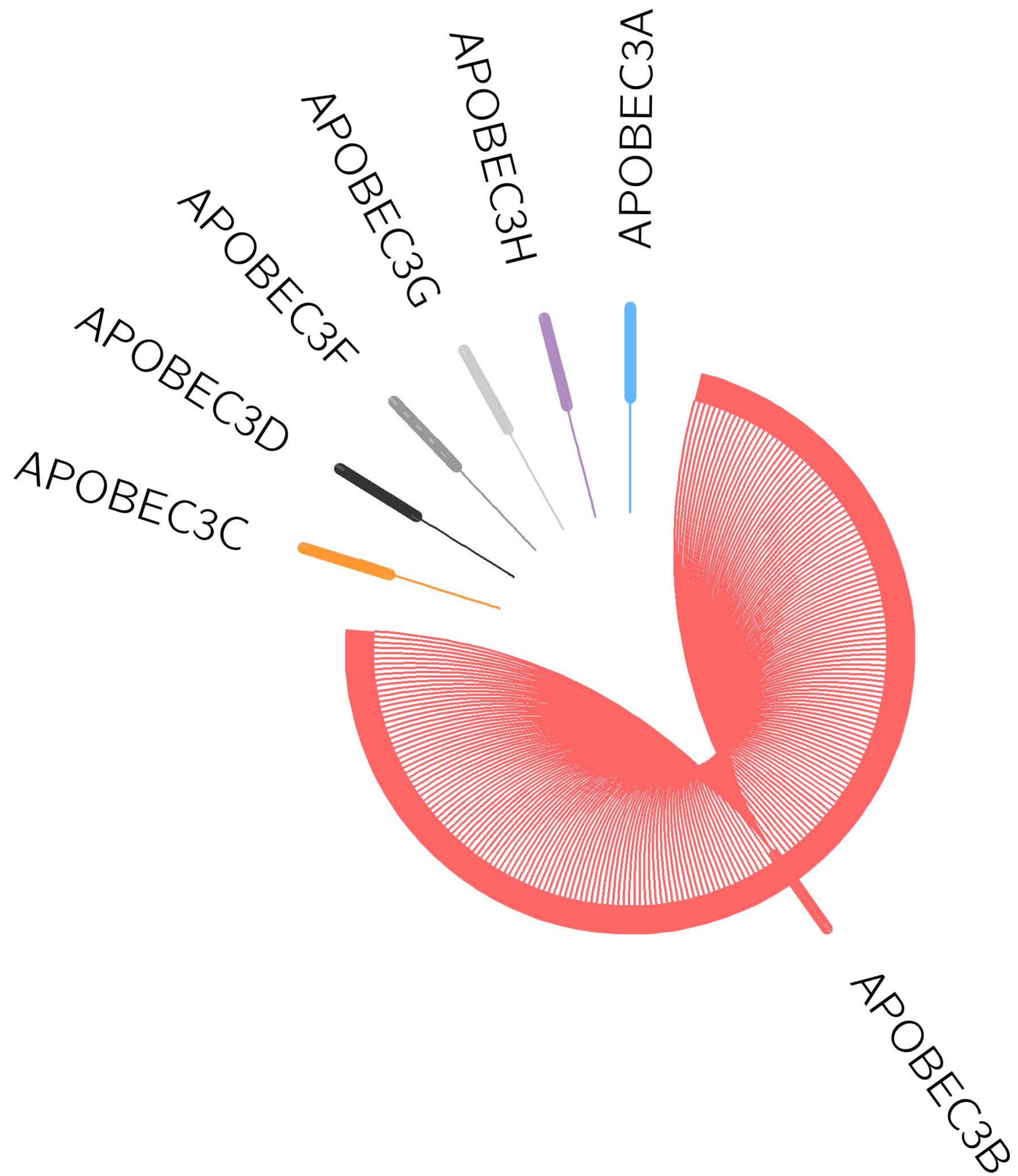

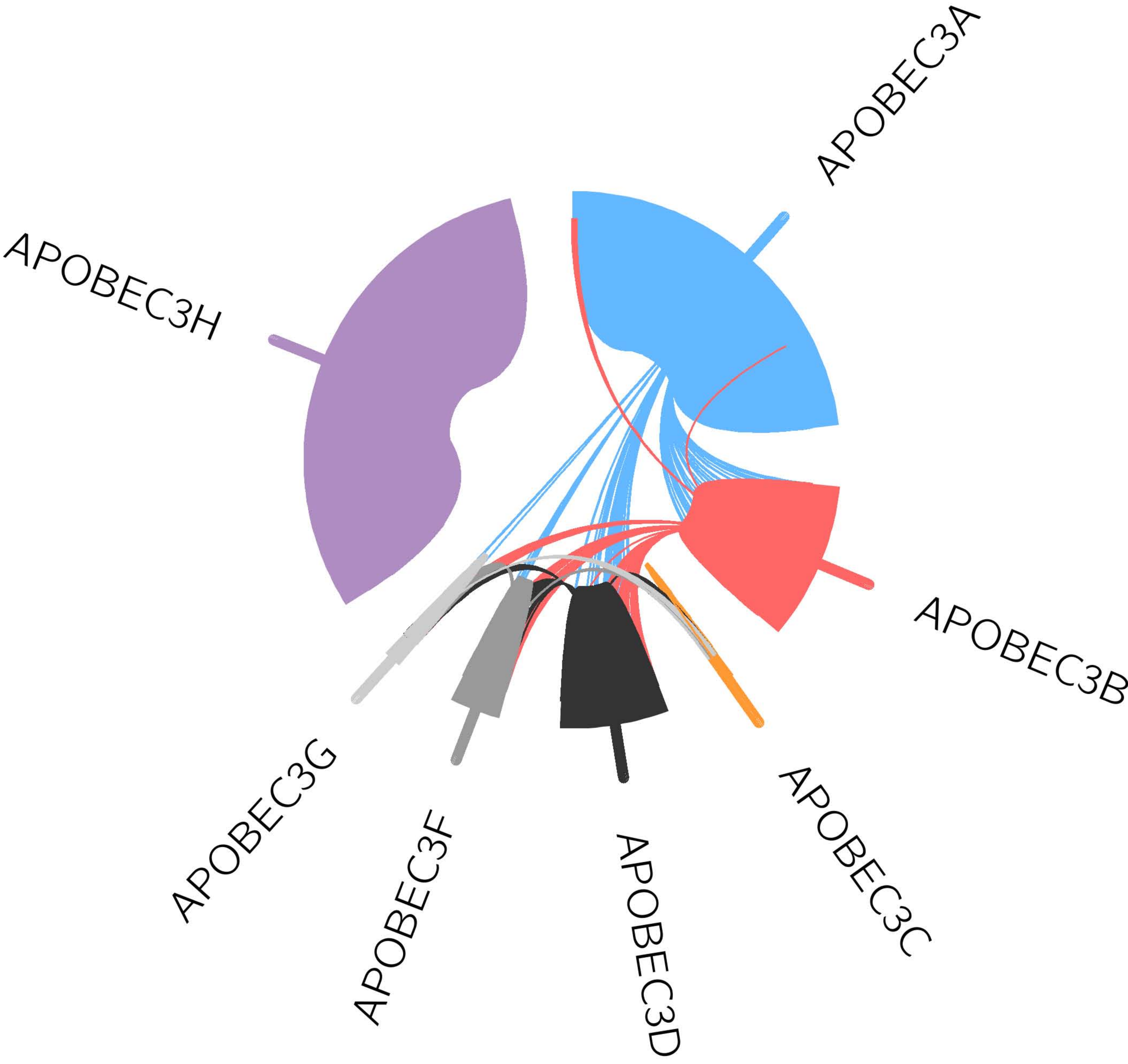

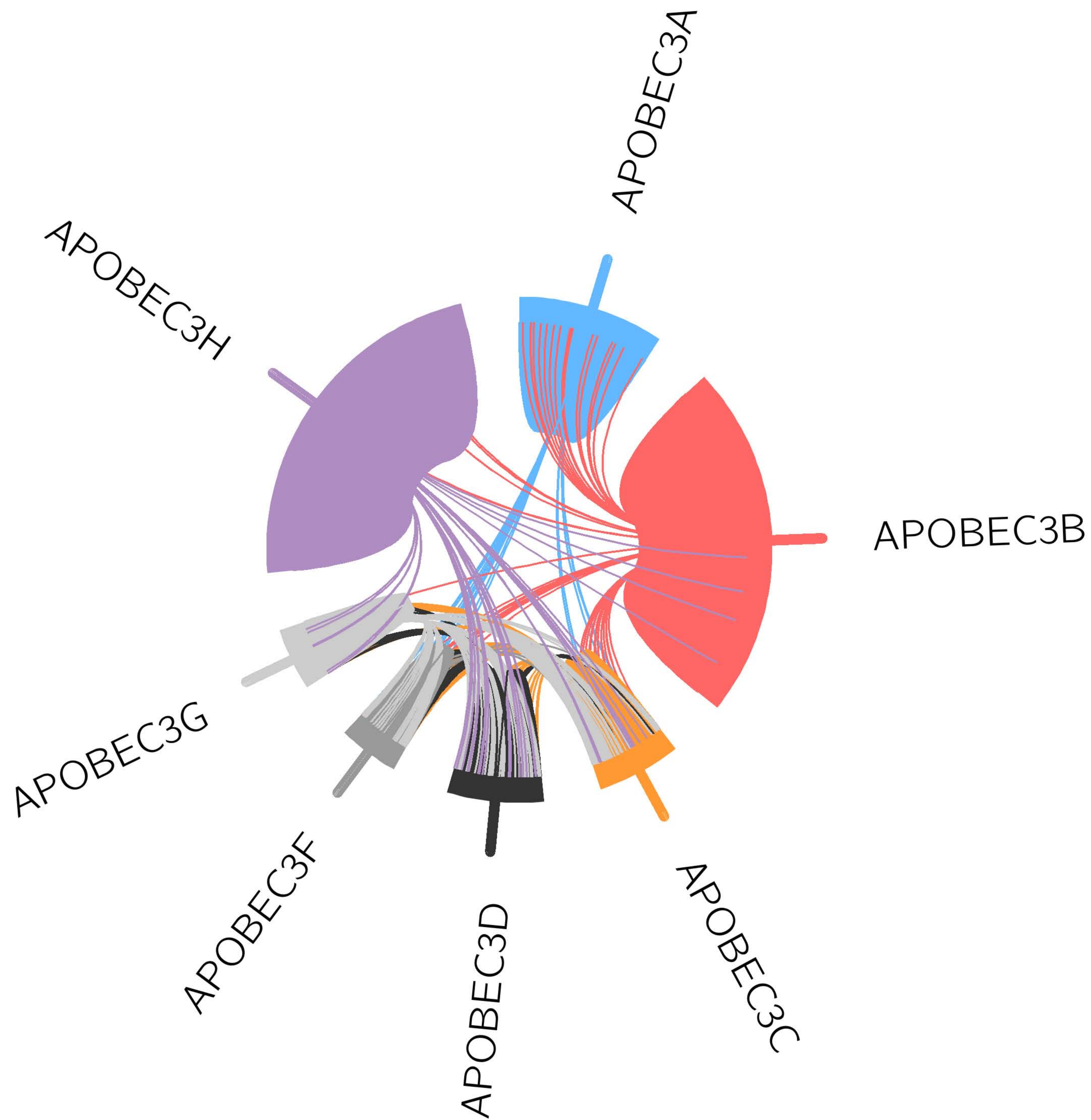

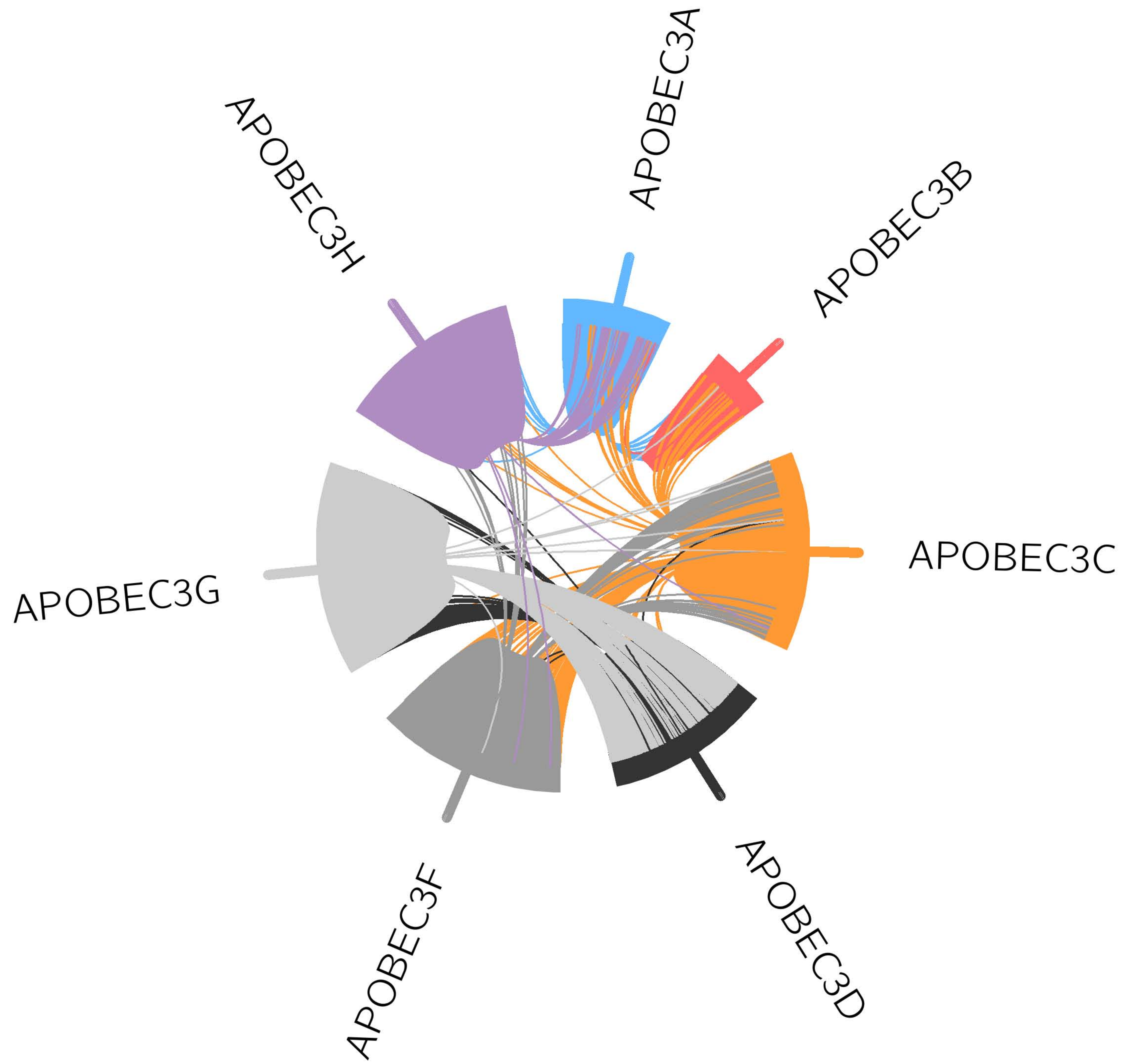

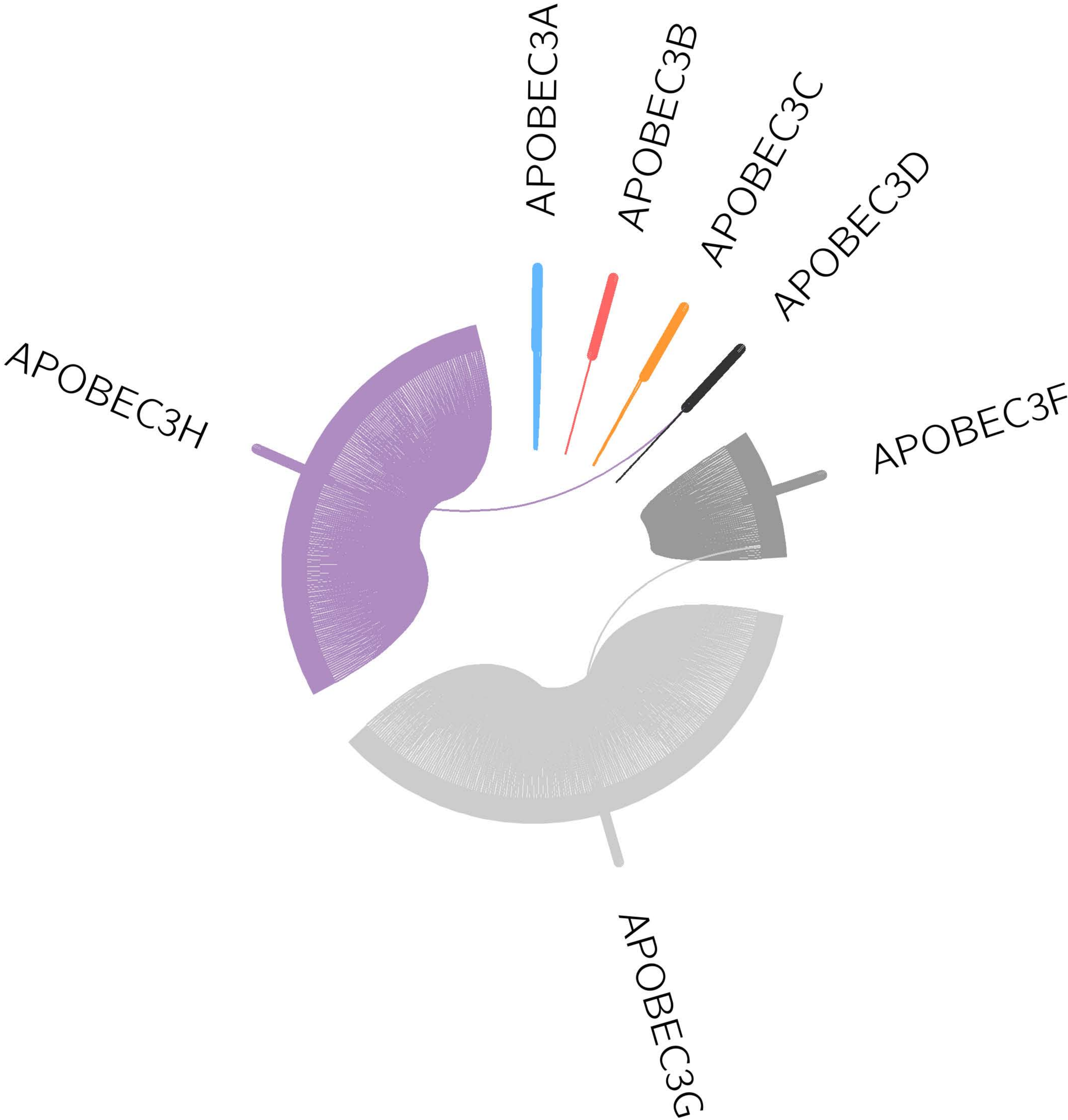

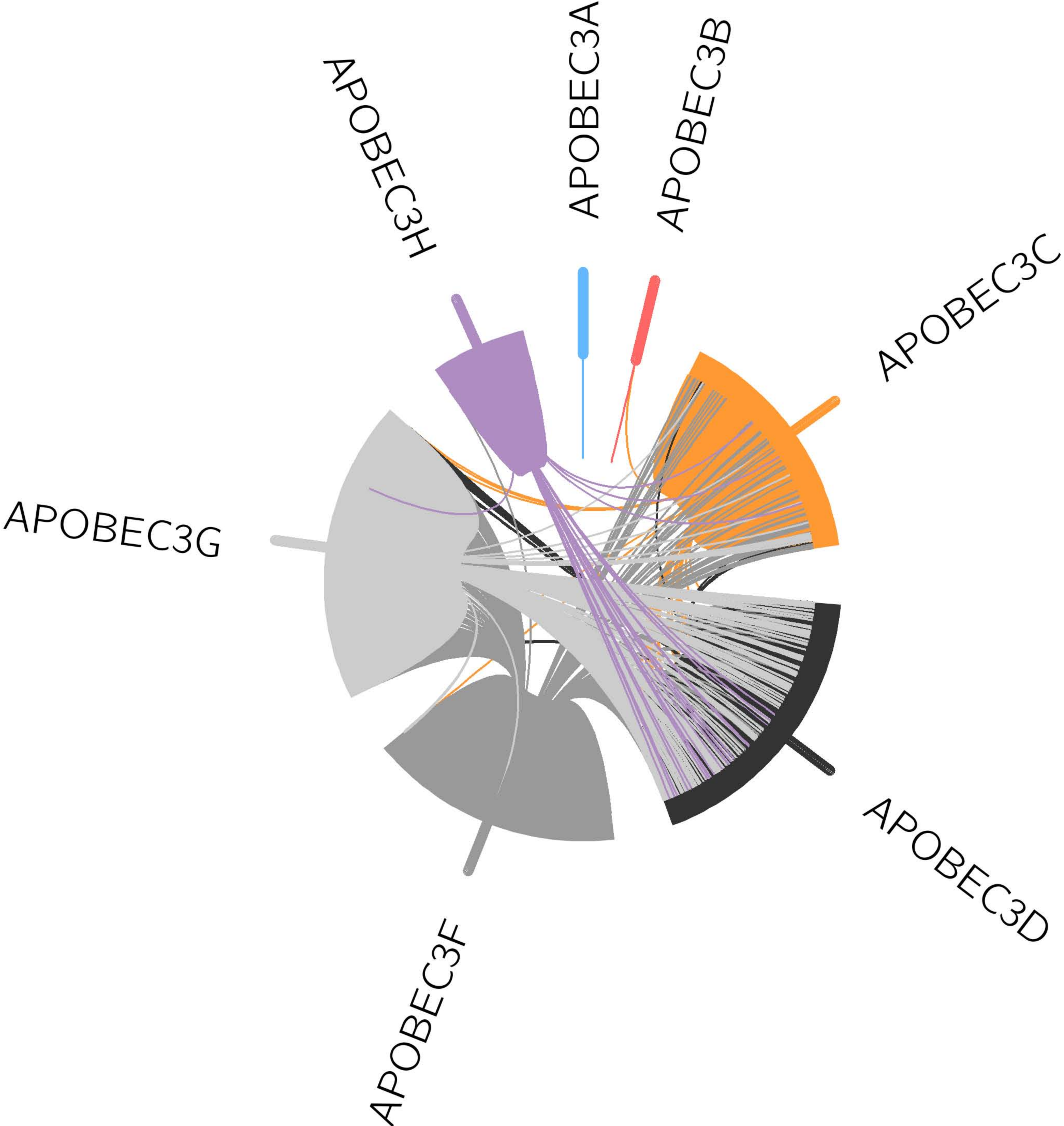

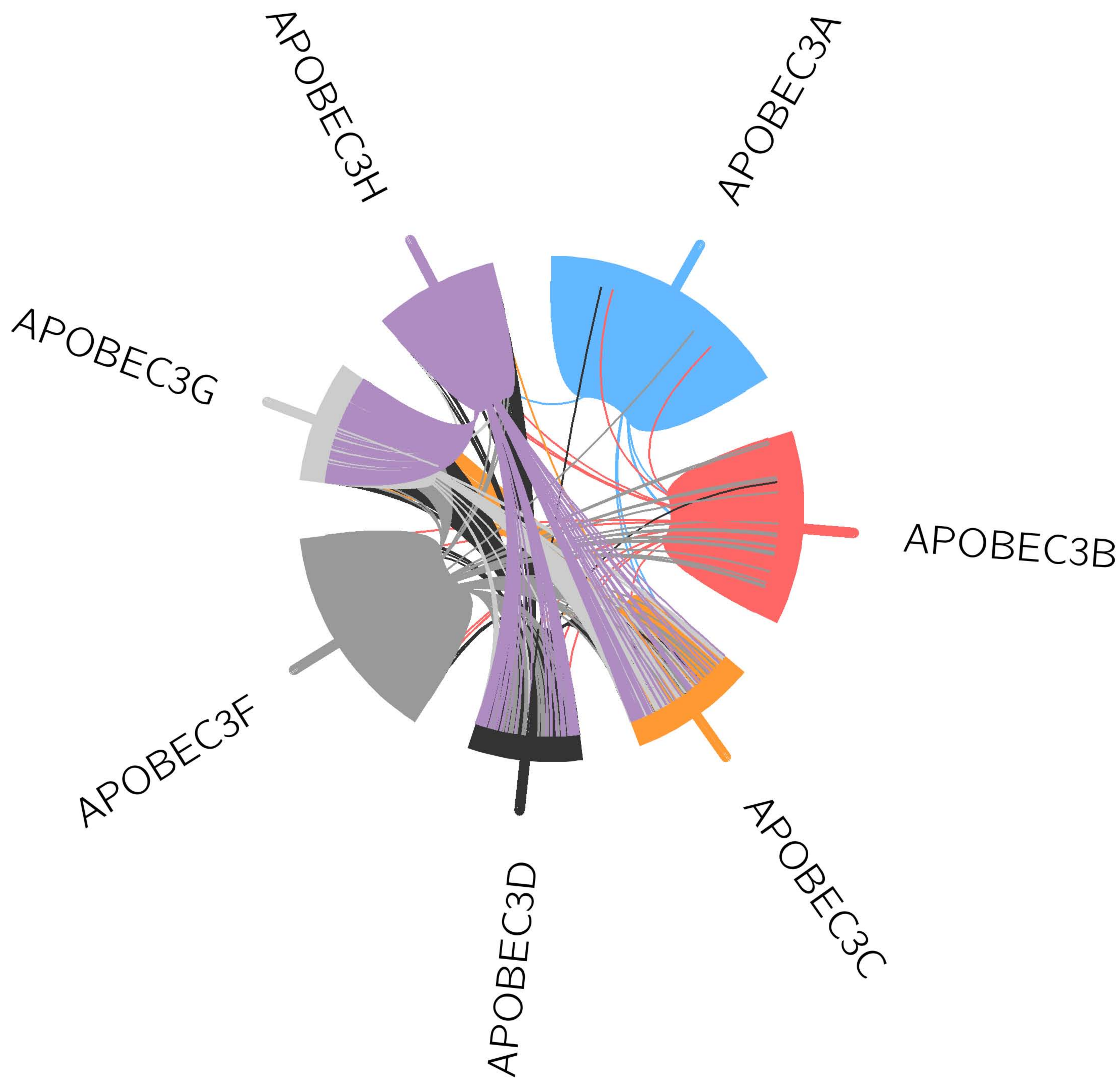

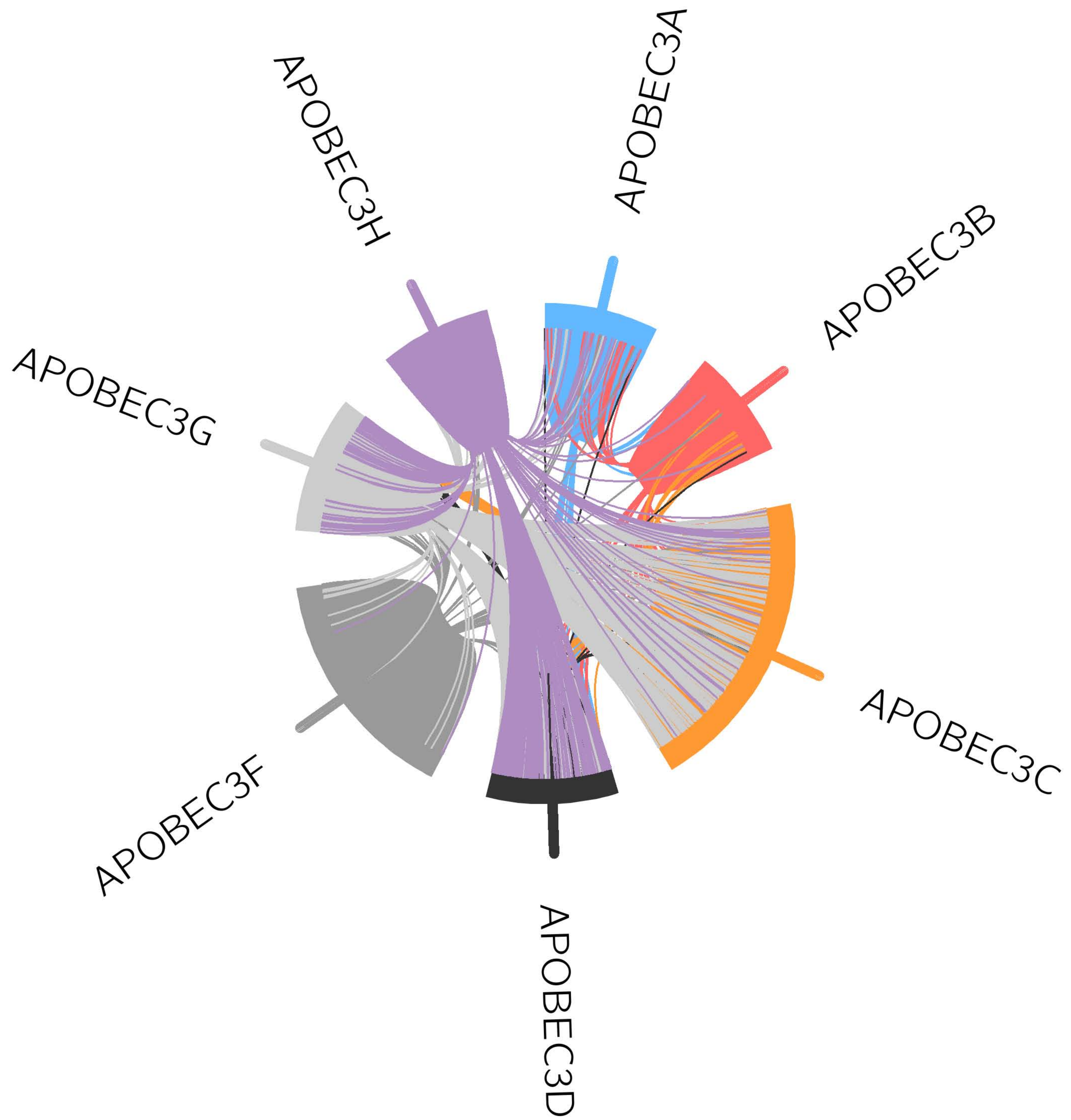

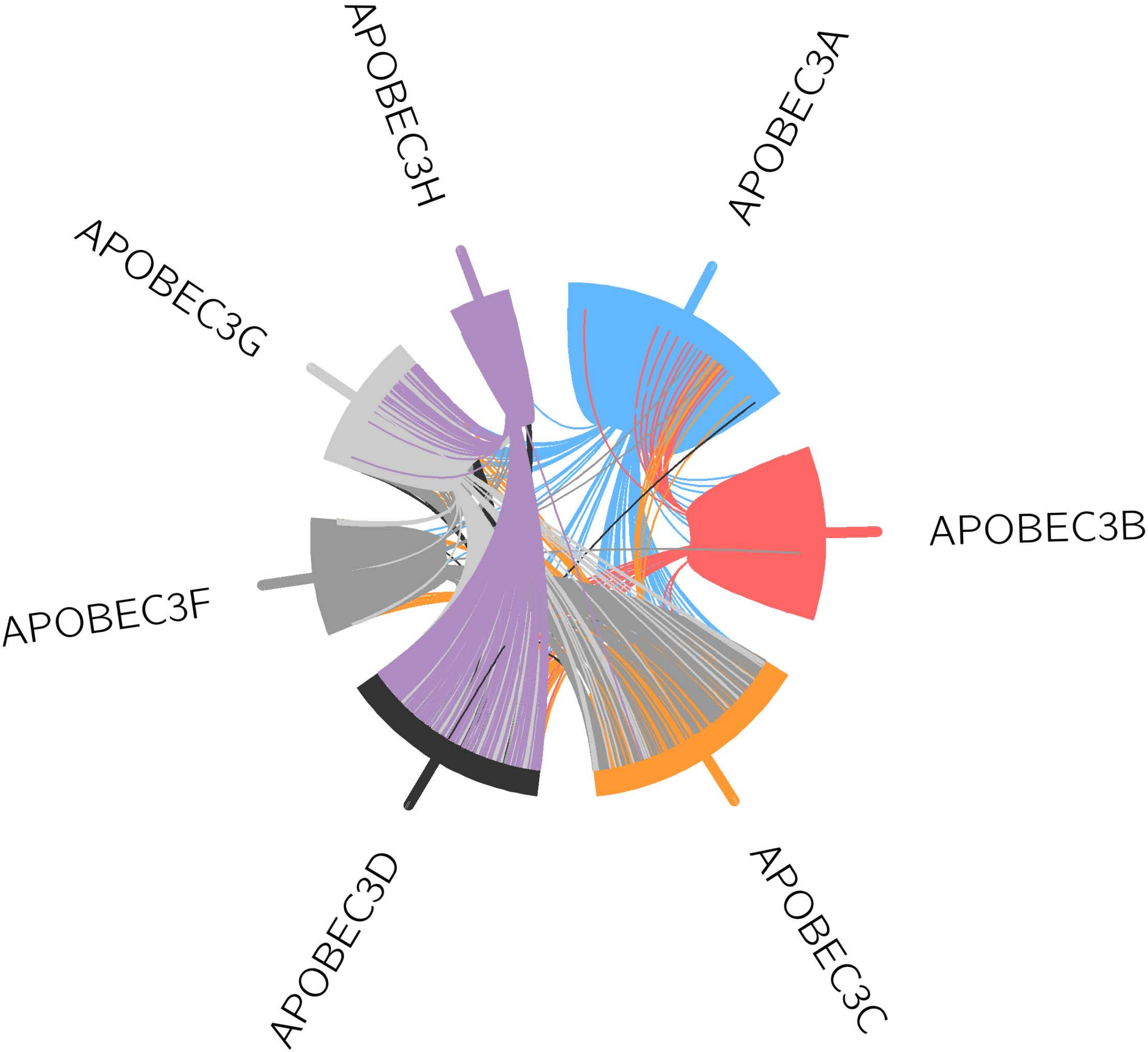

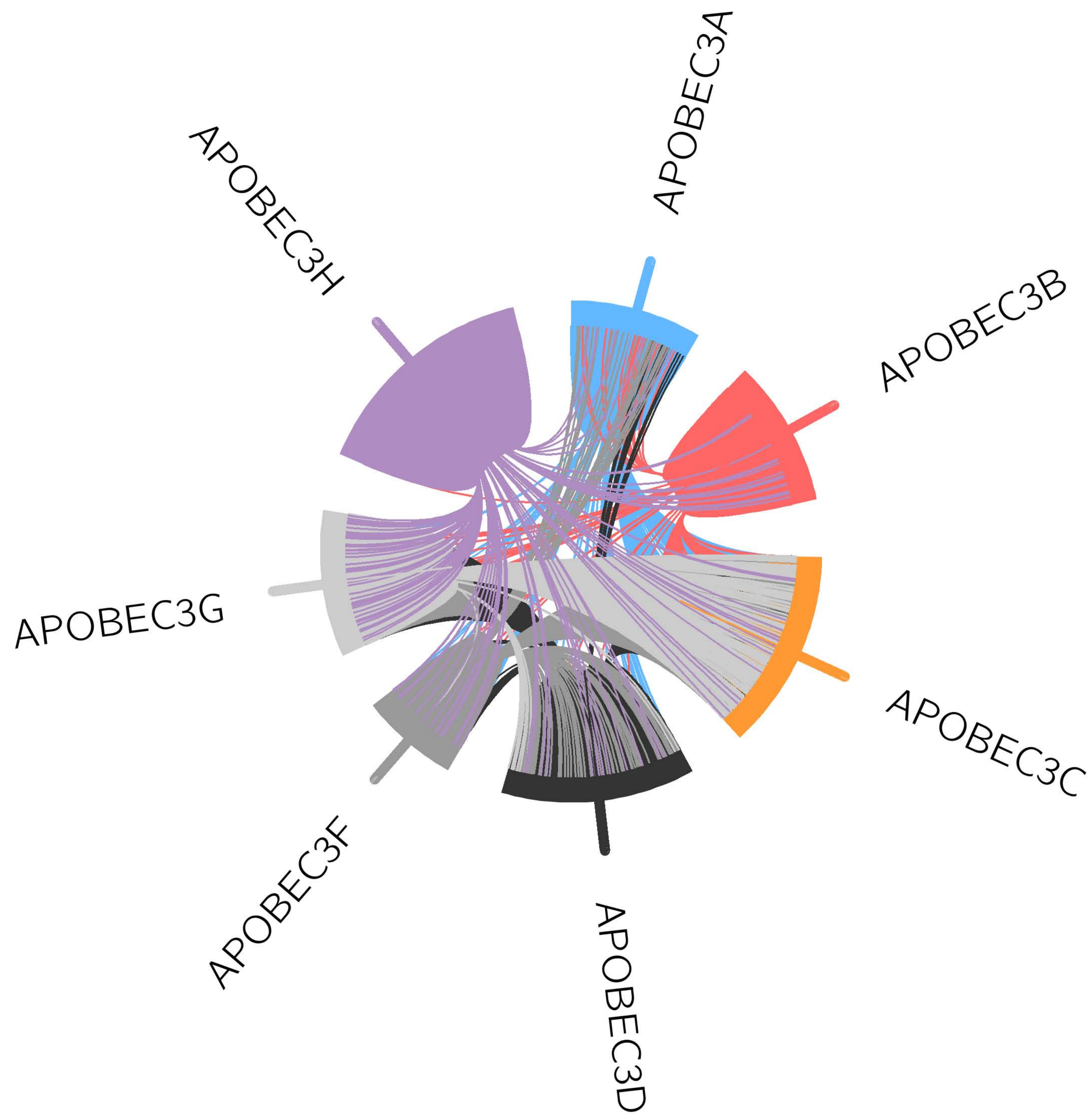

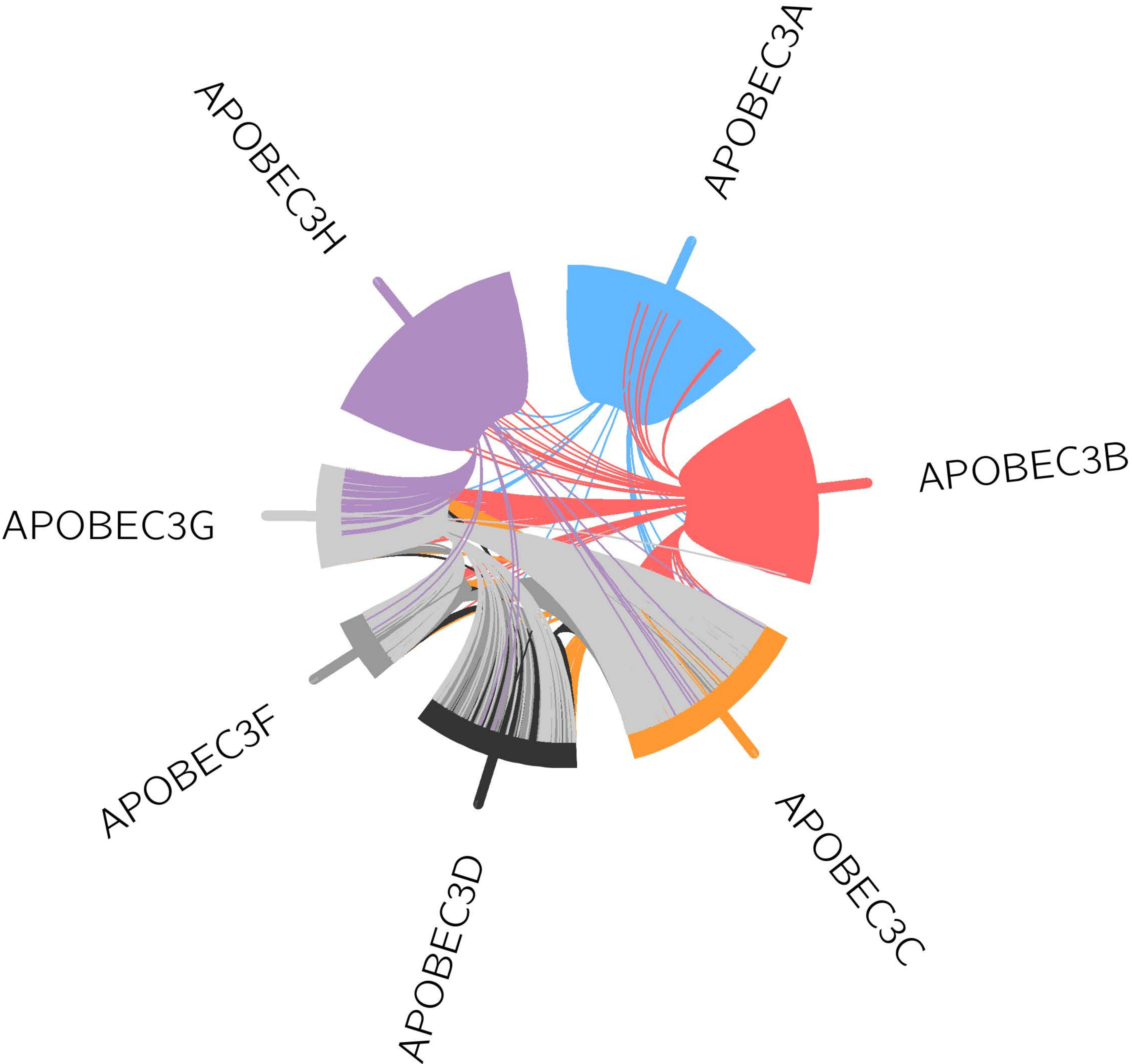

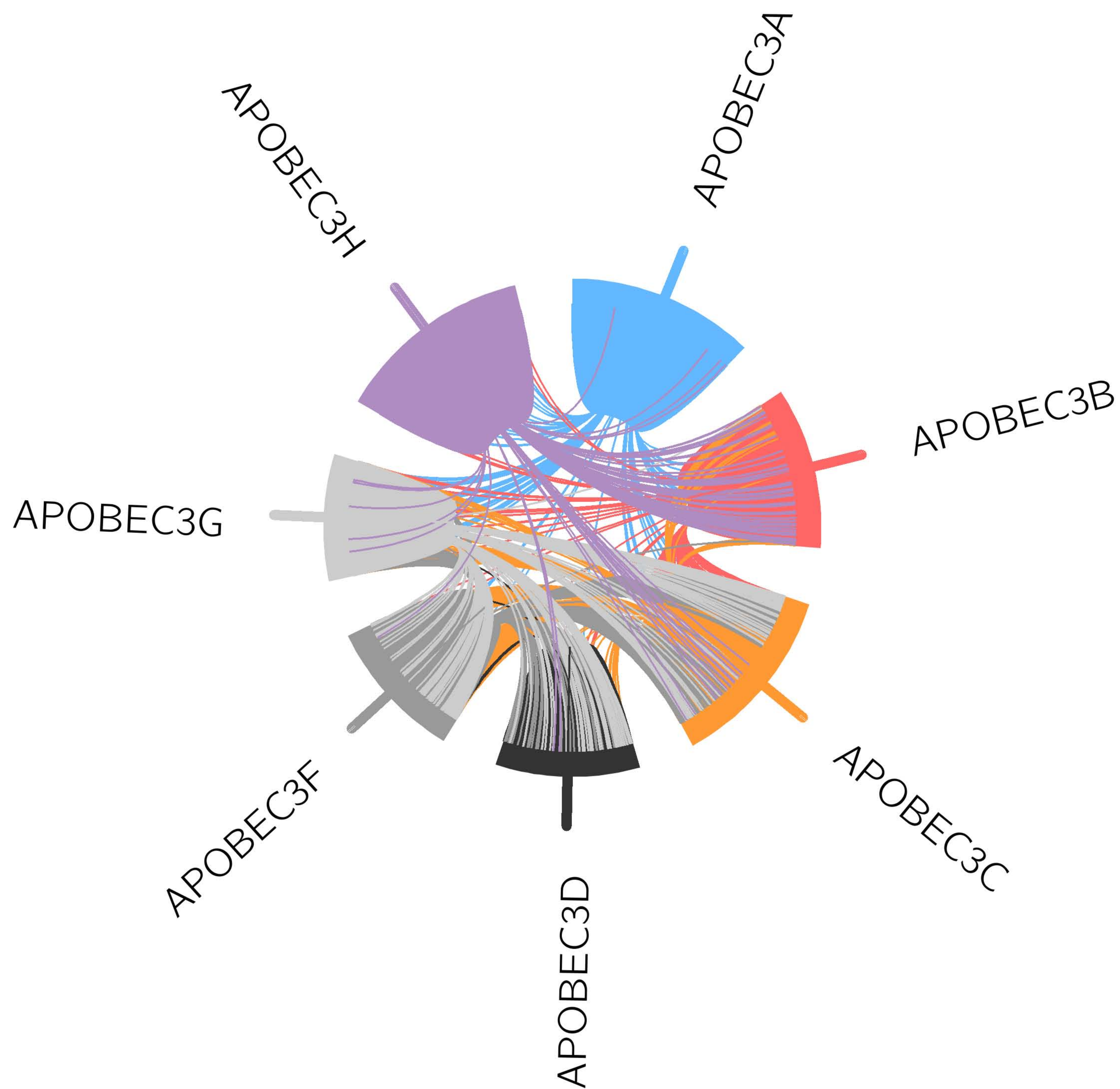

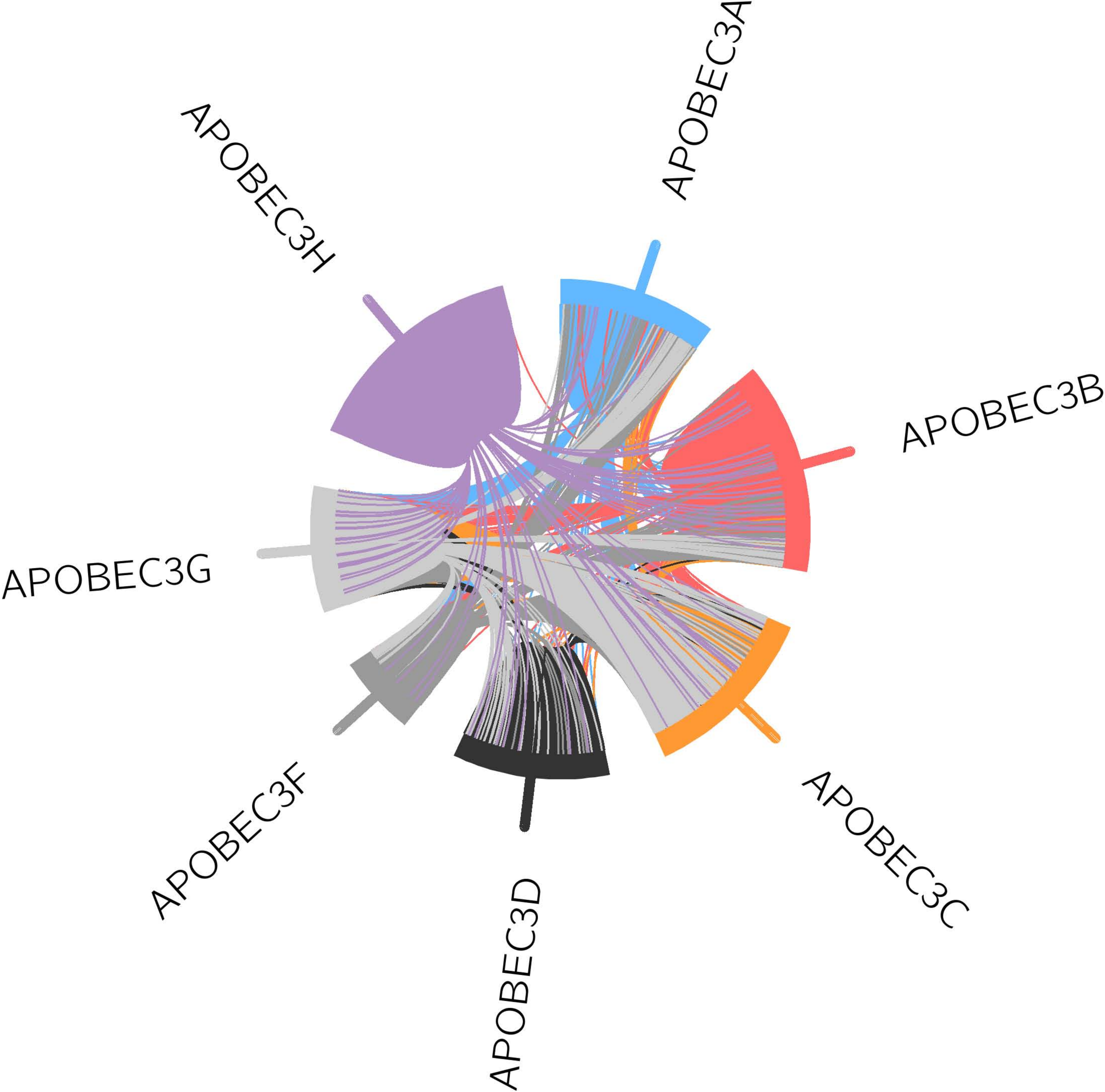

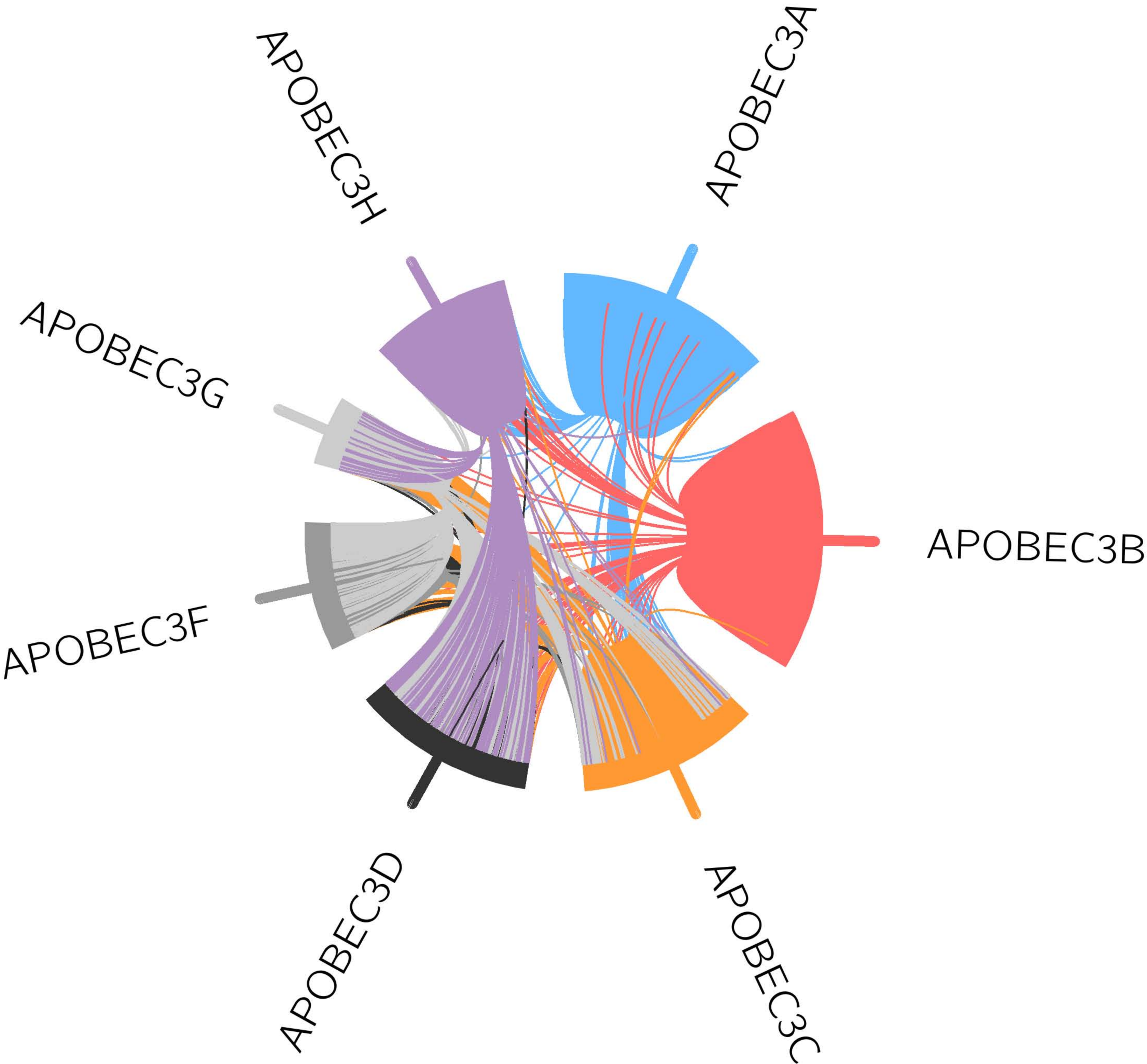

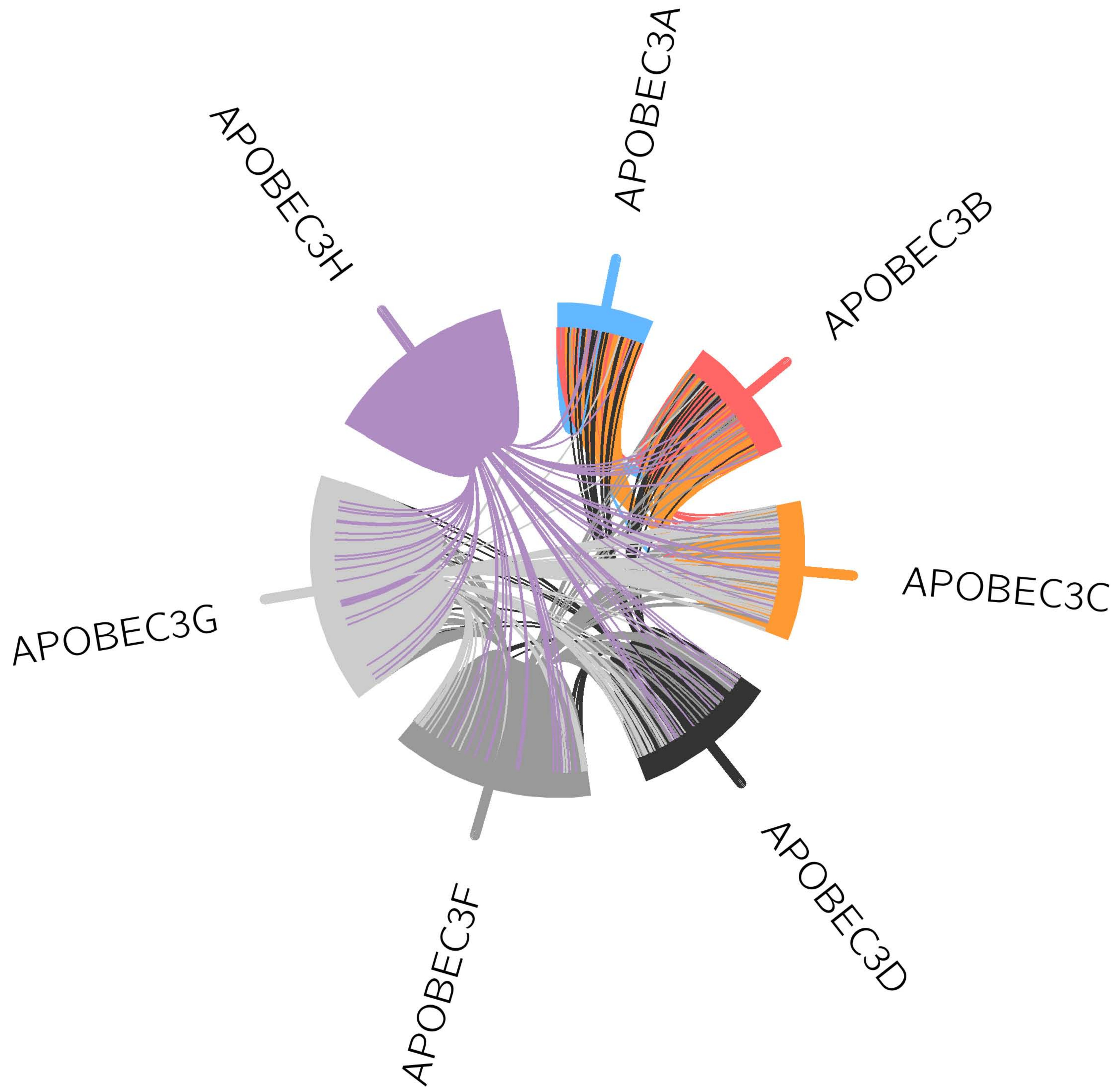

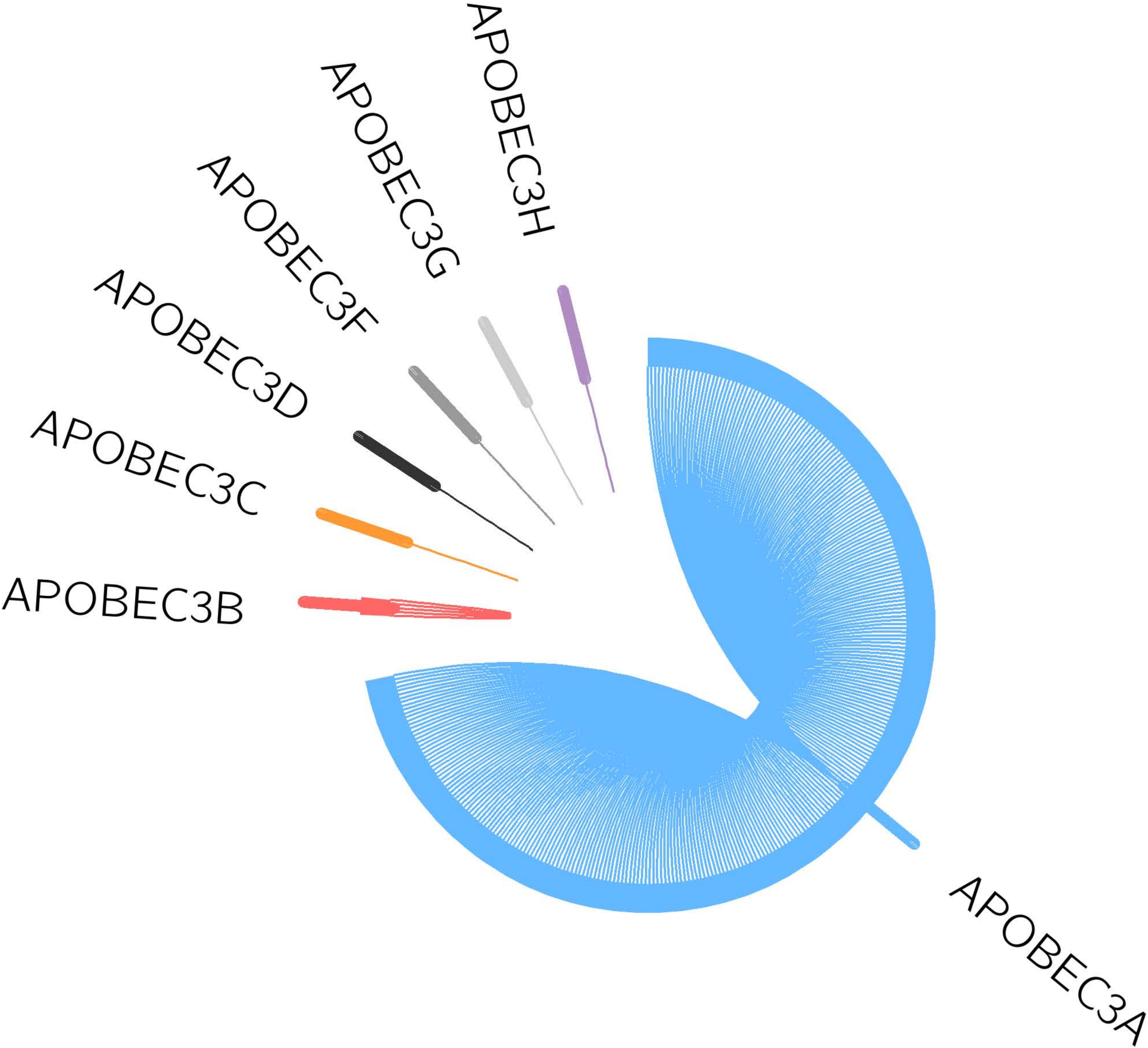

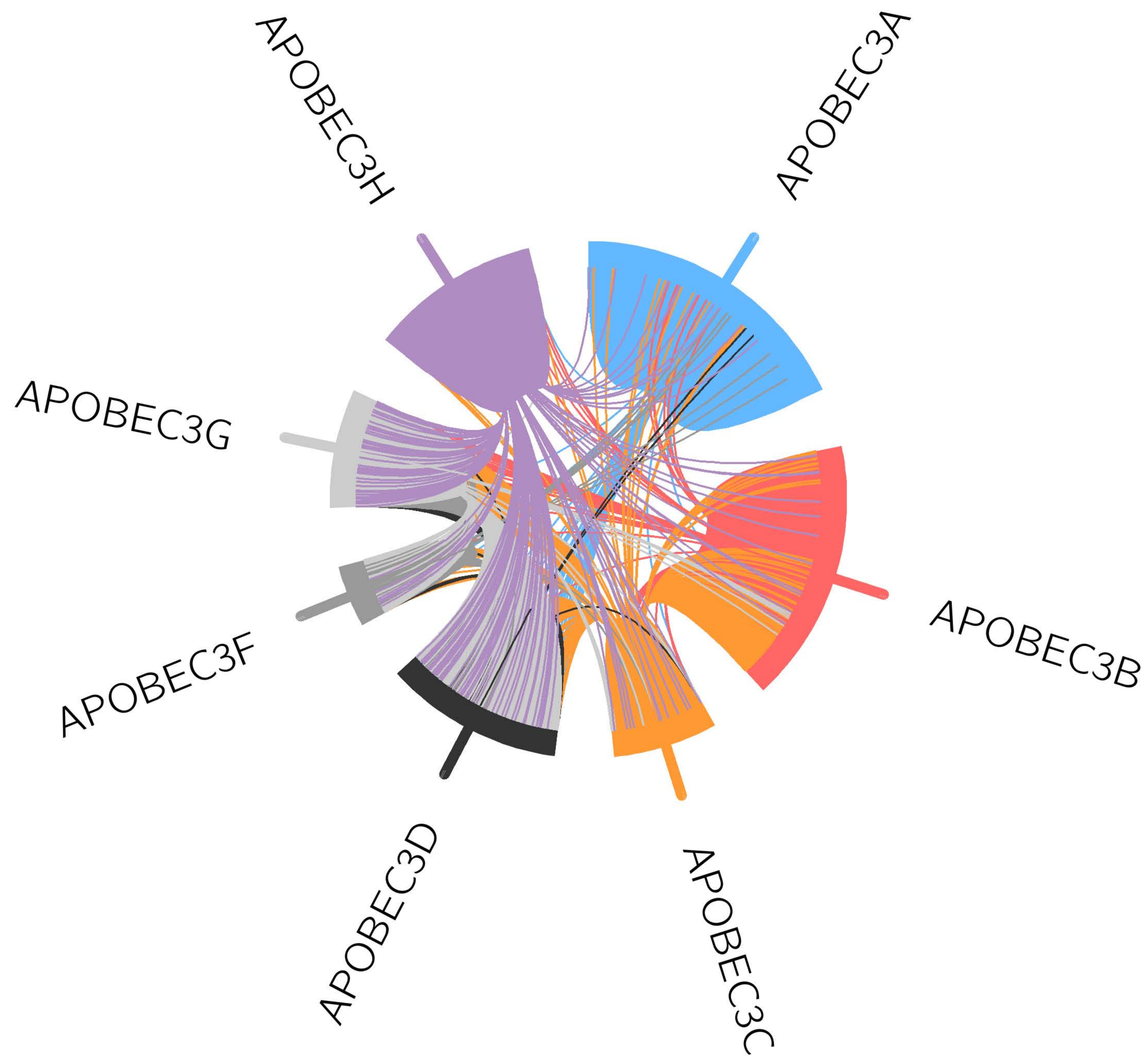

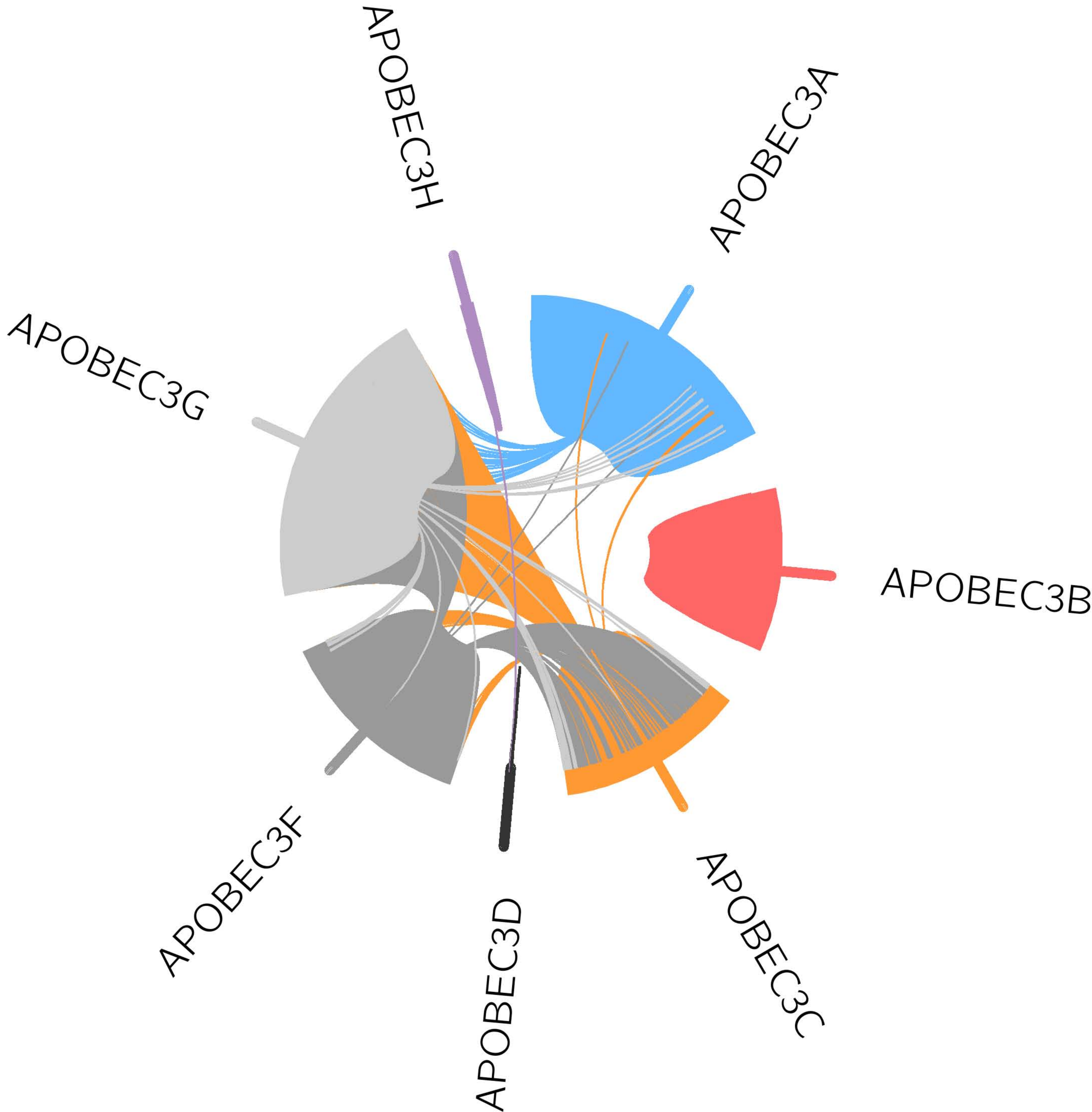

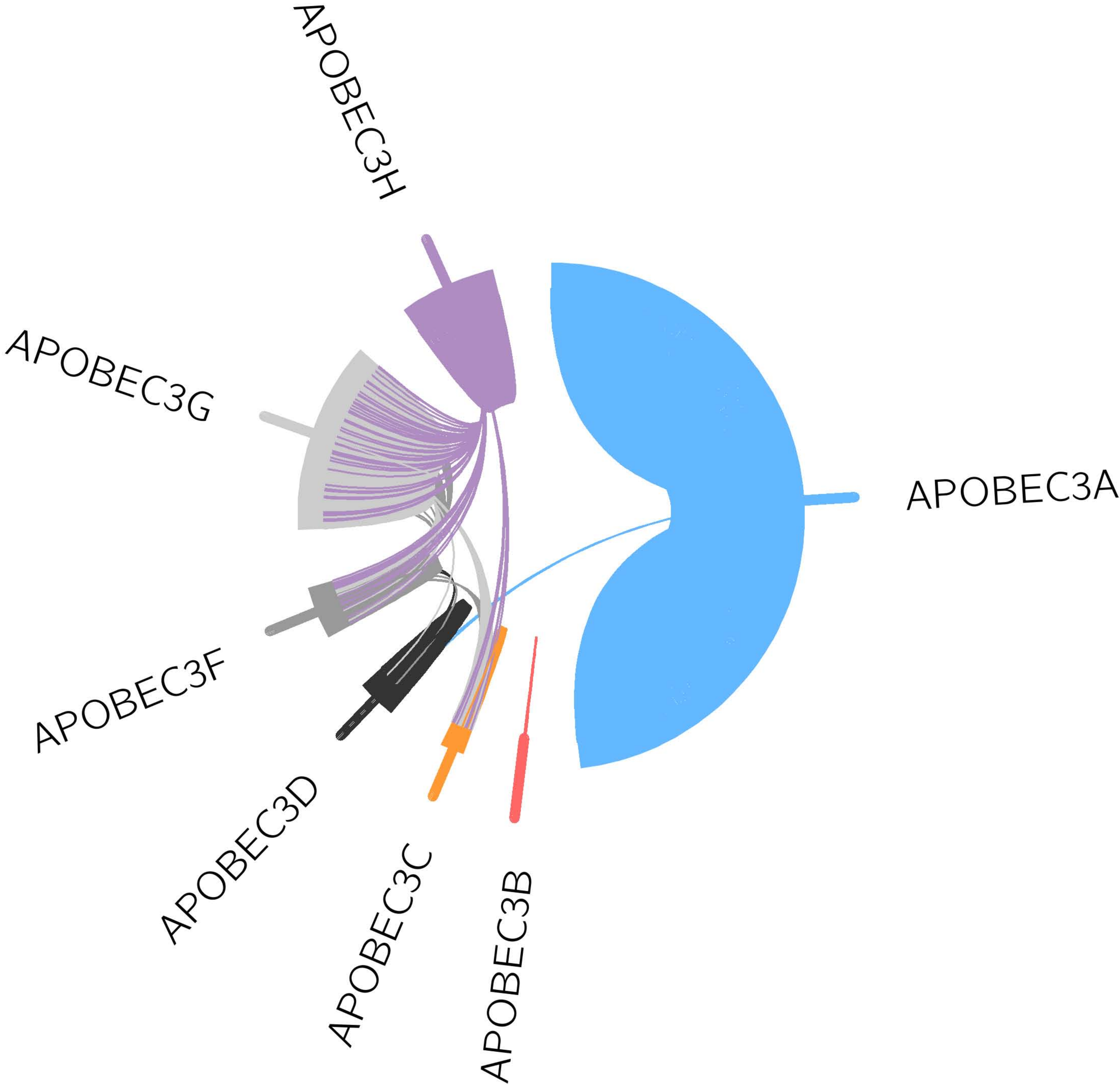

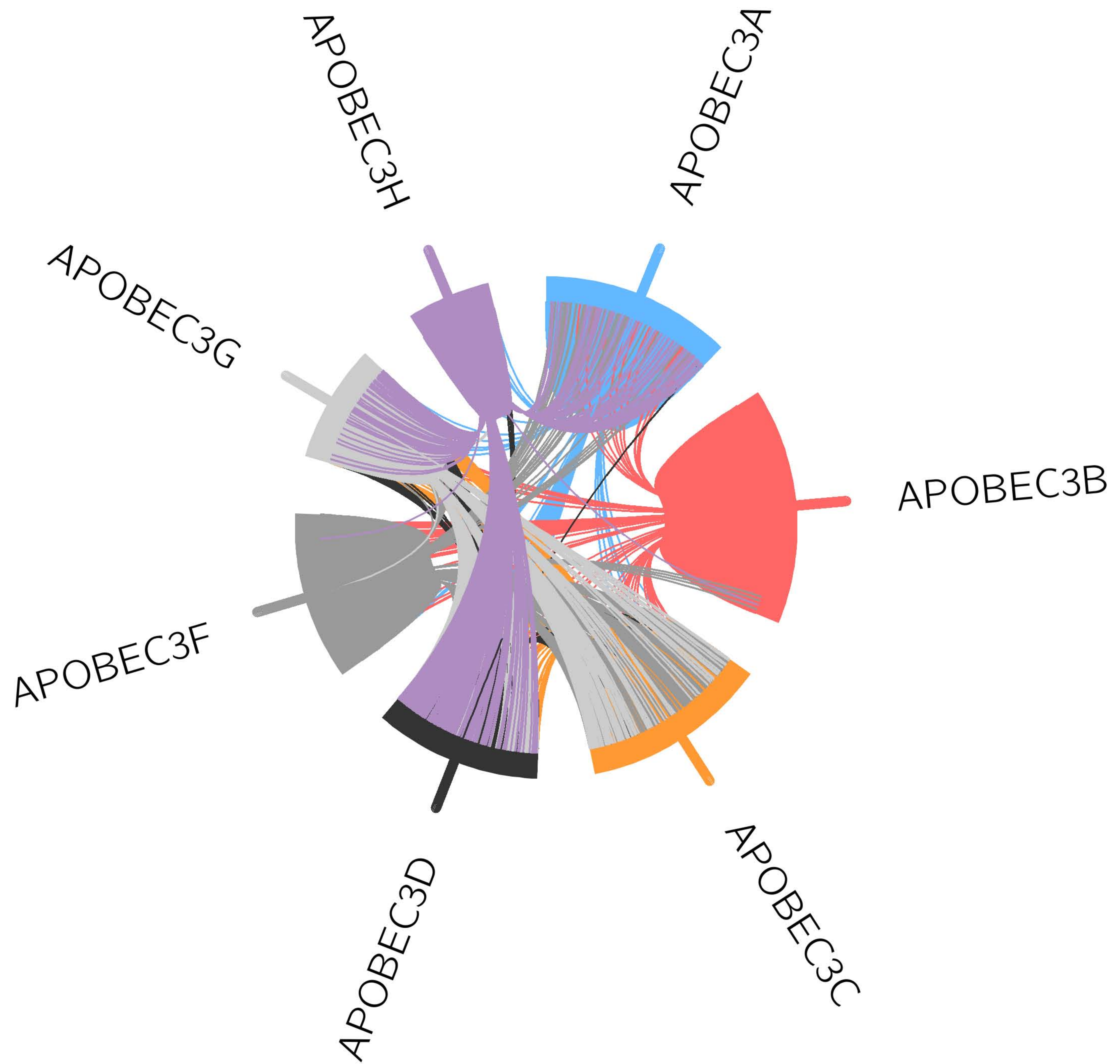

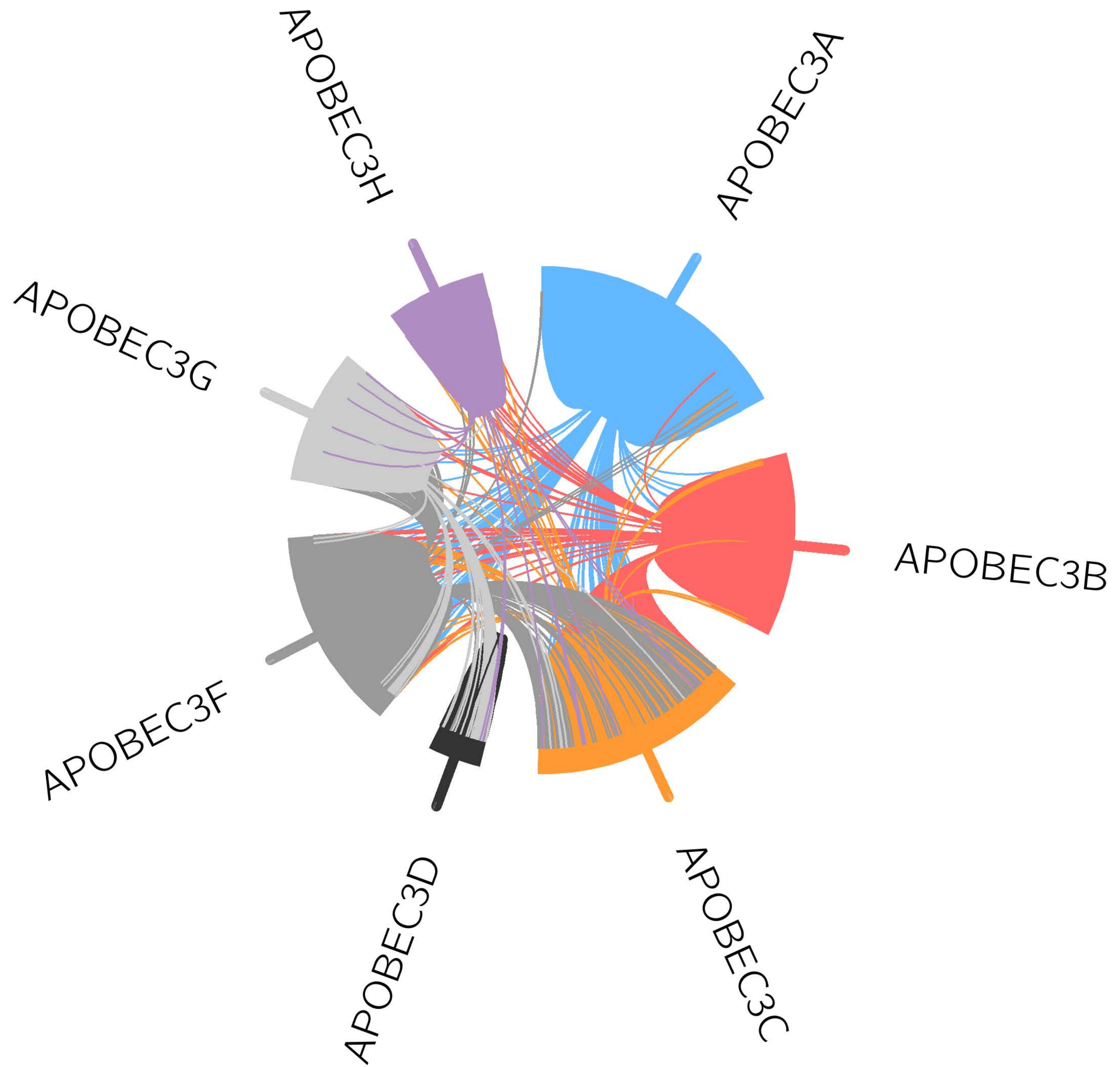

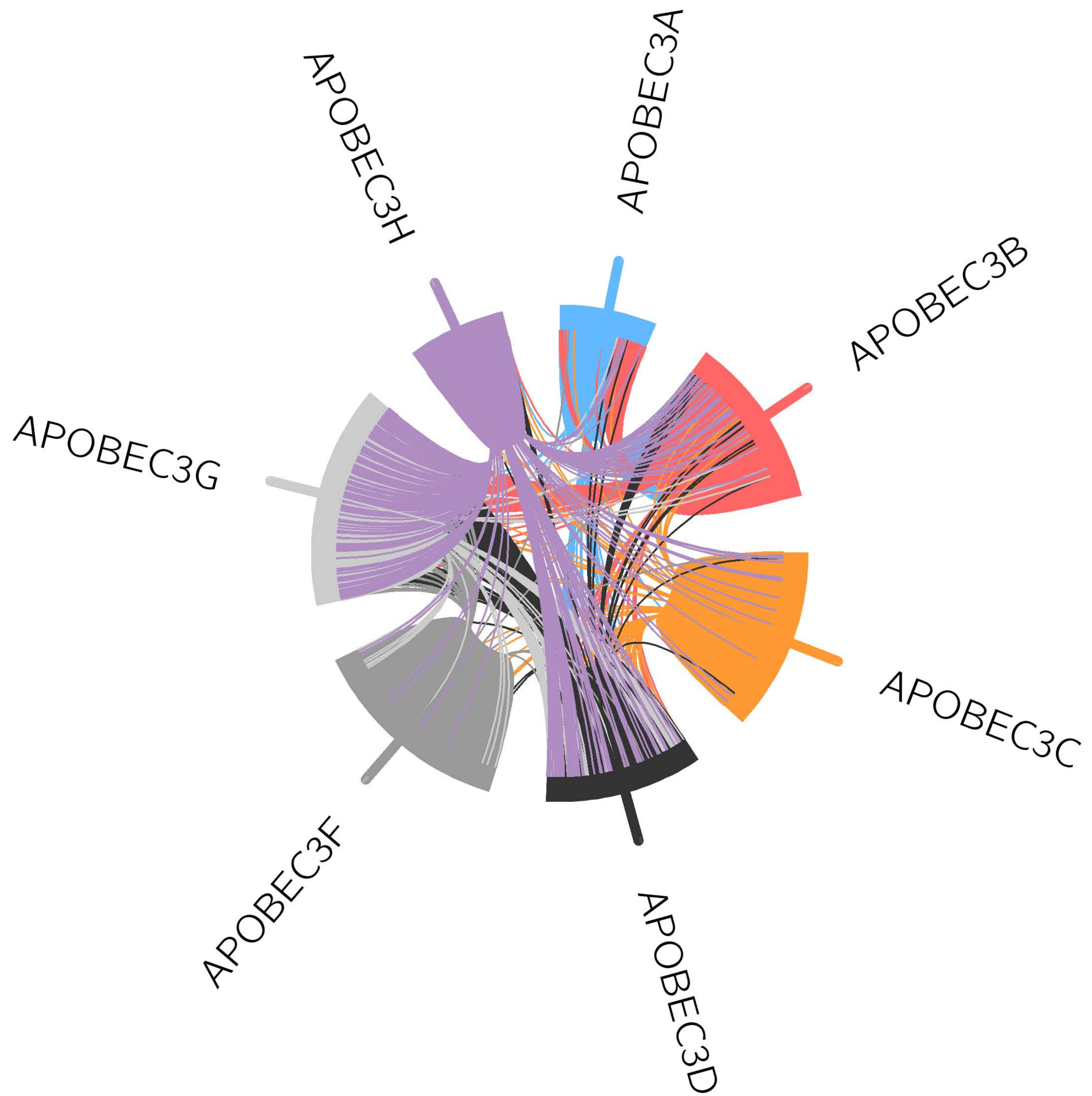

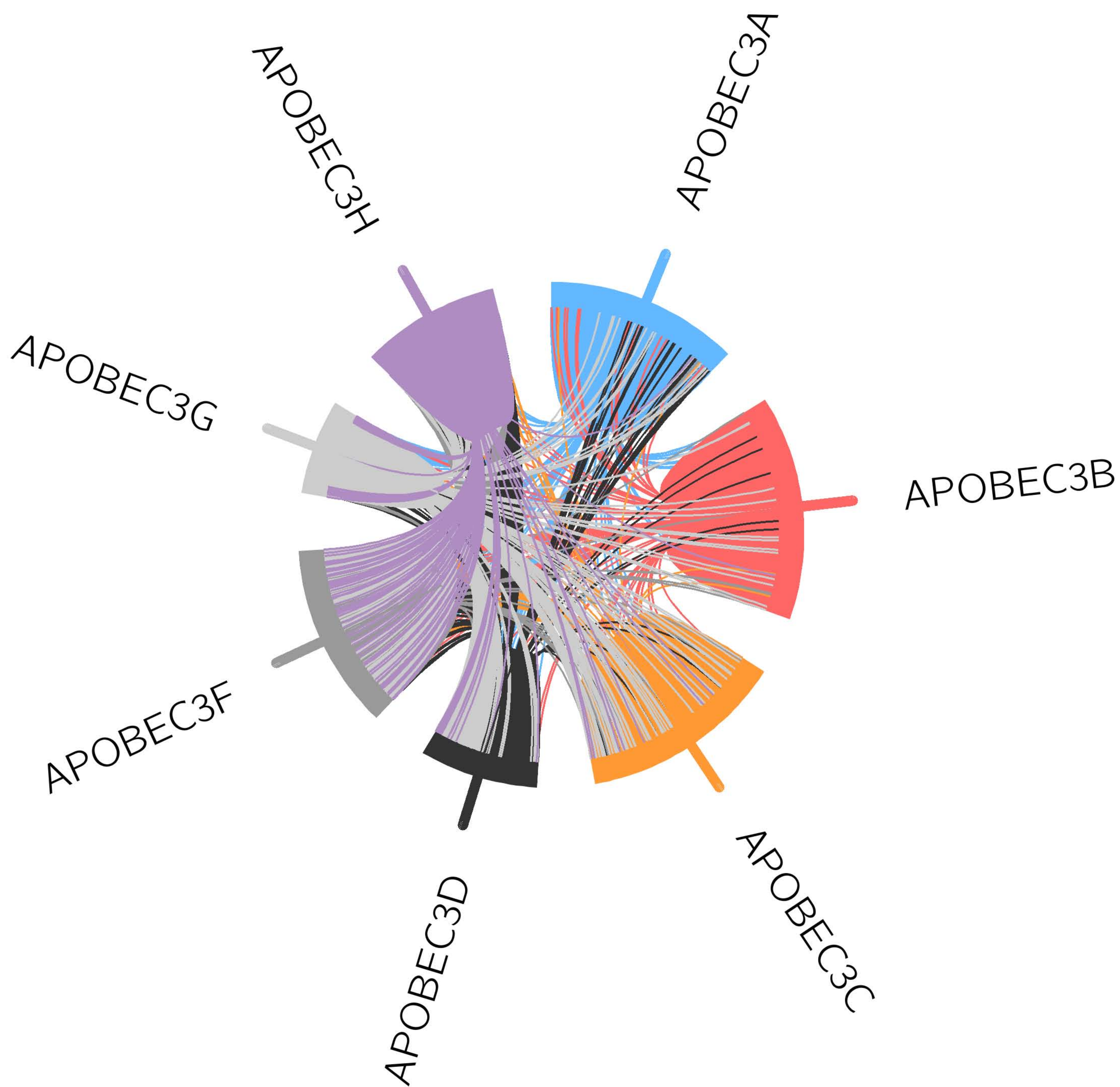

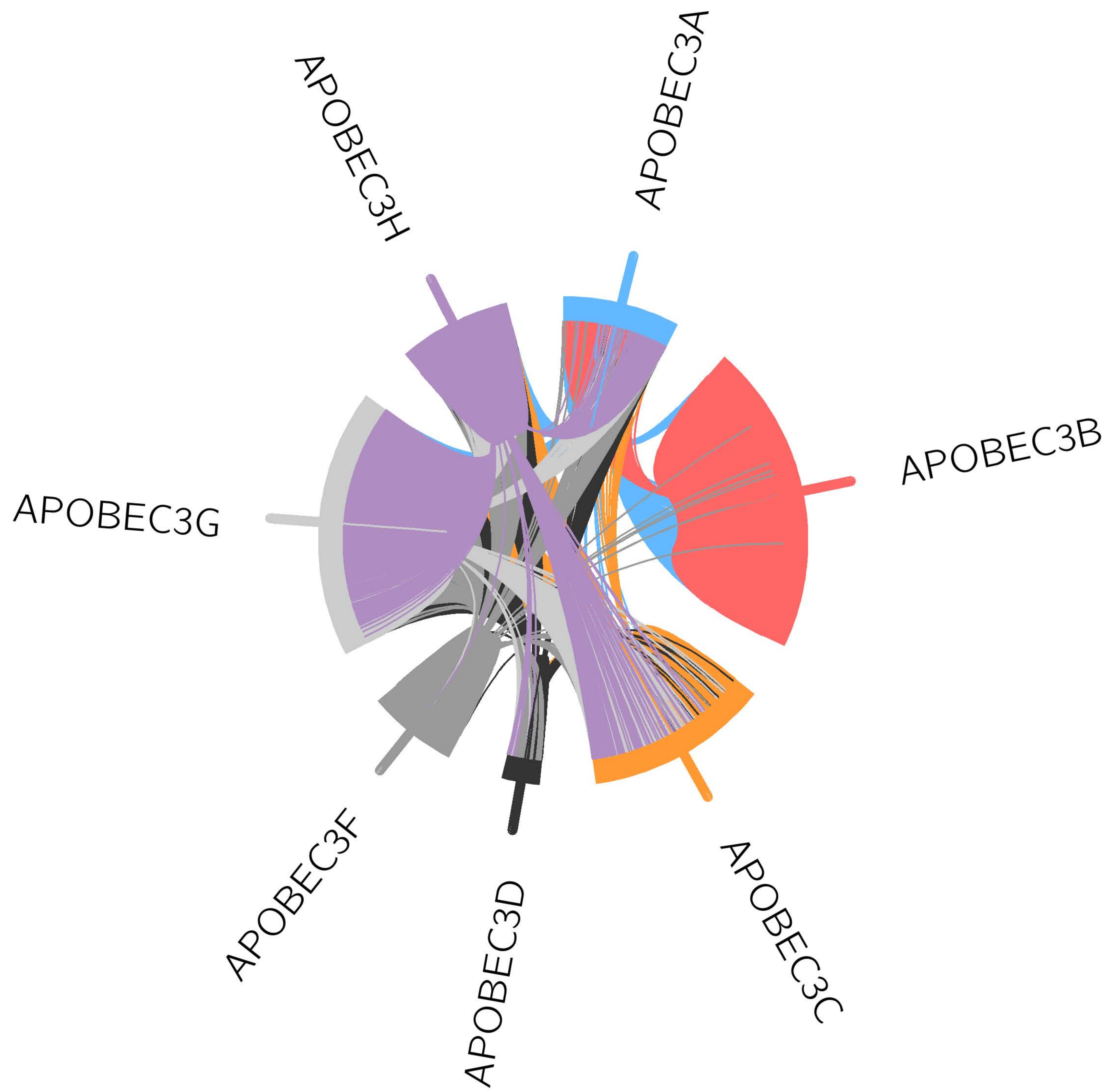

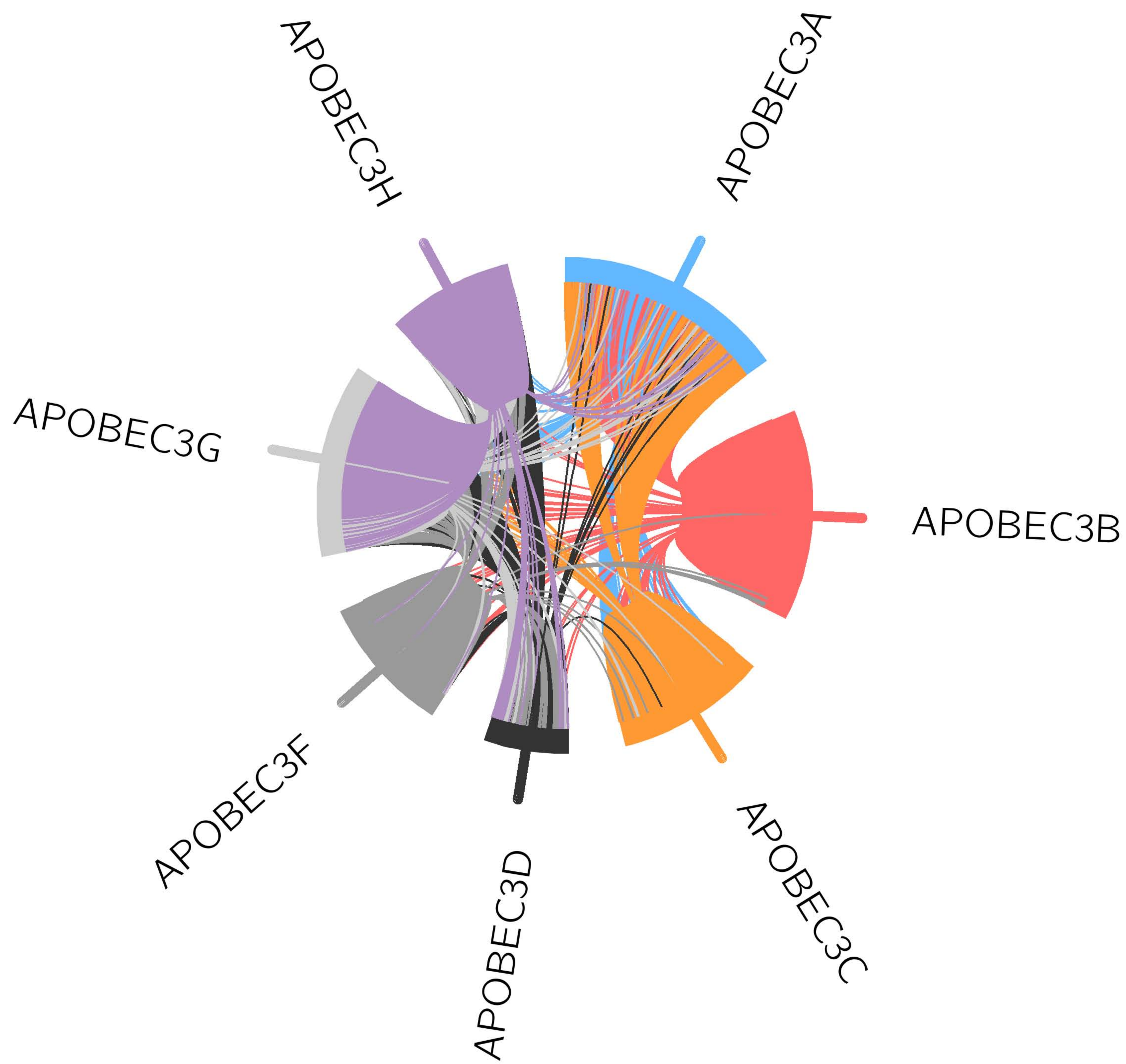

TCGA.COADREAD\_bootstrap

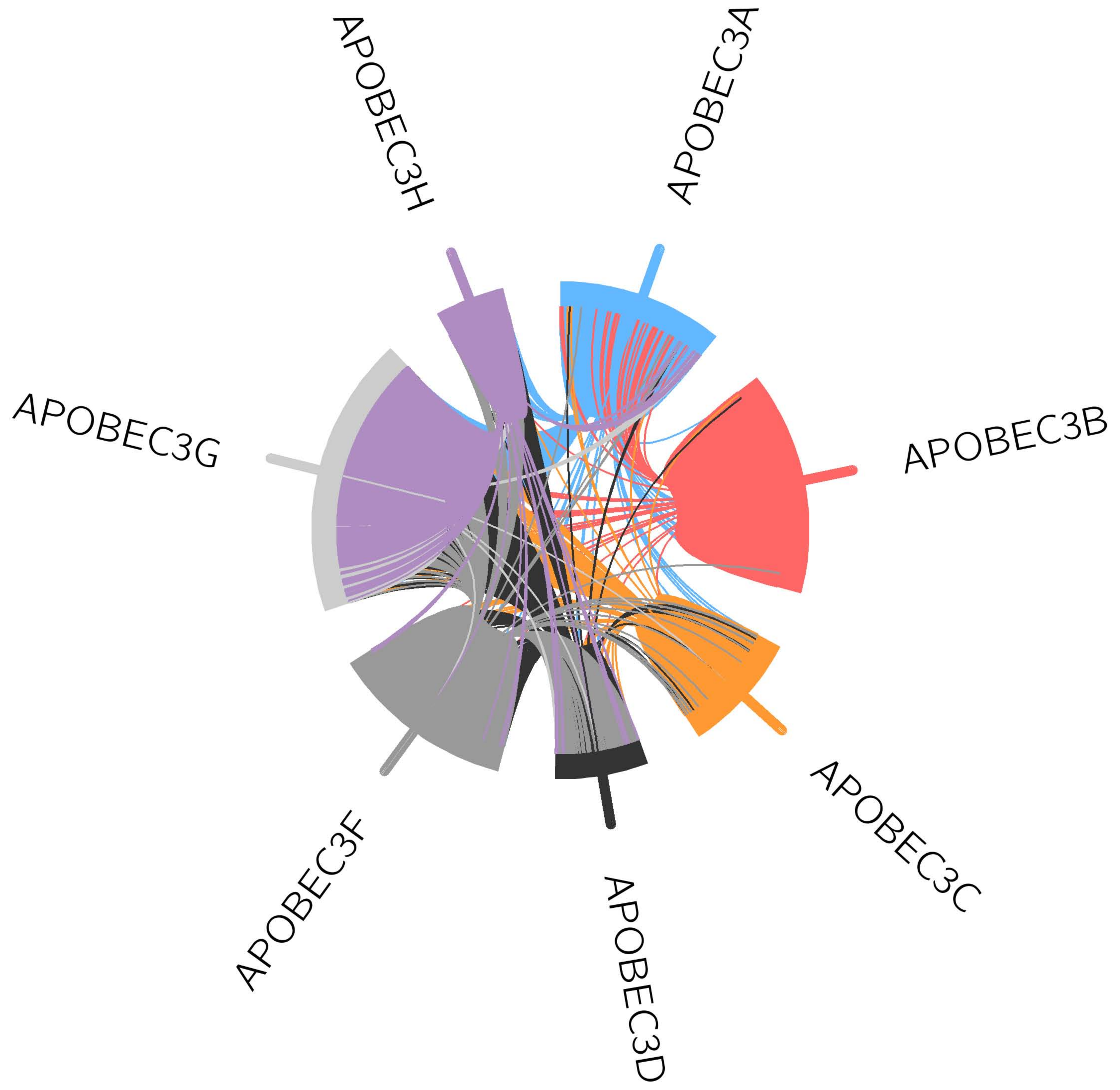

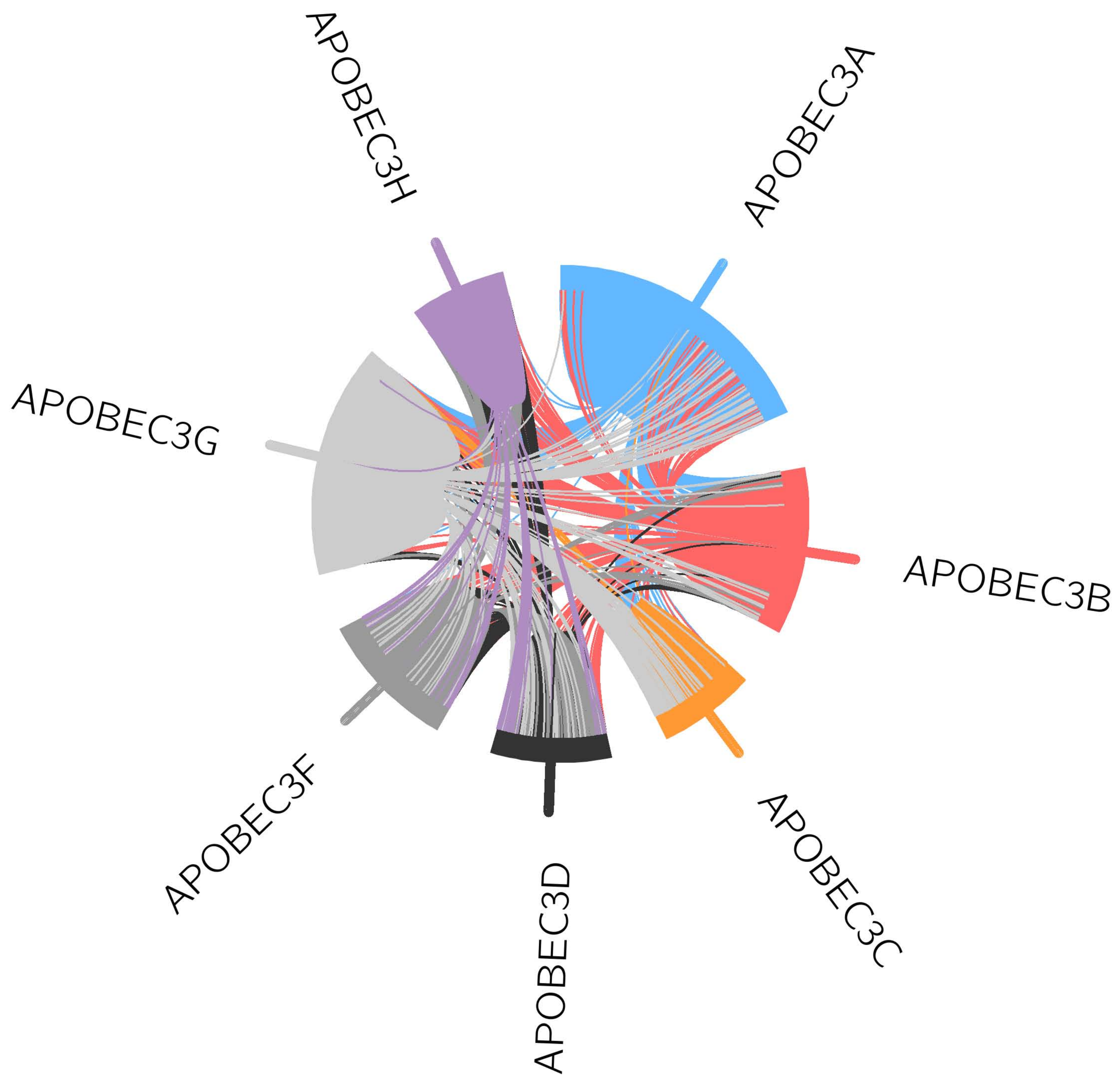

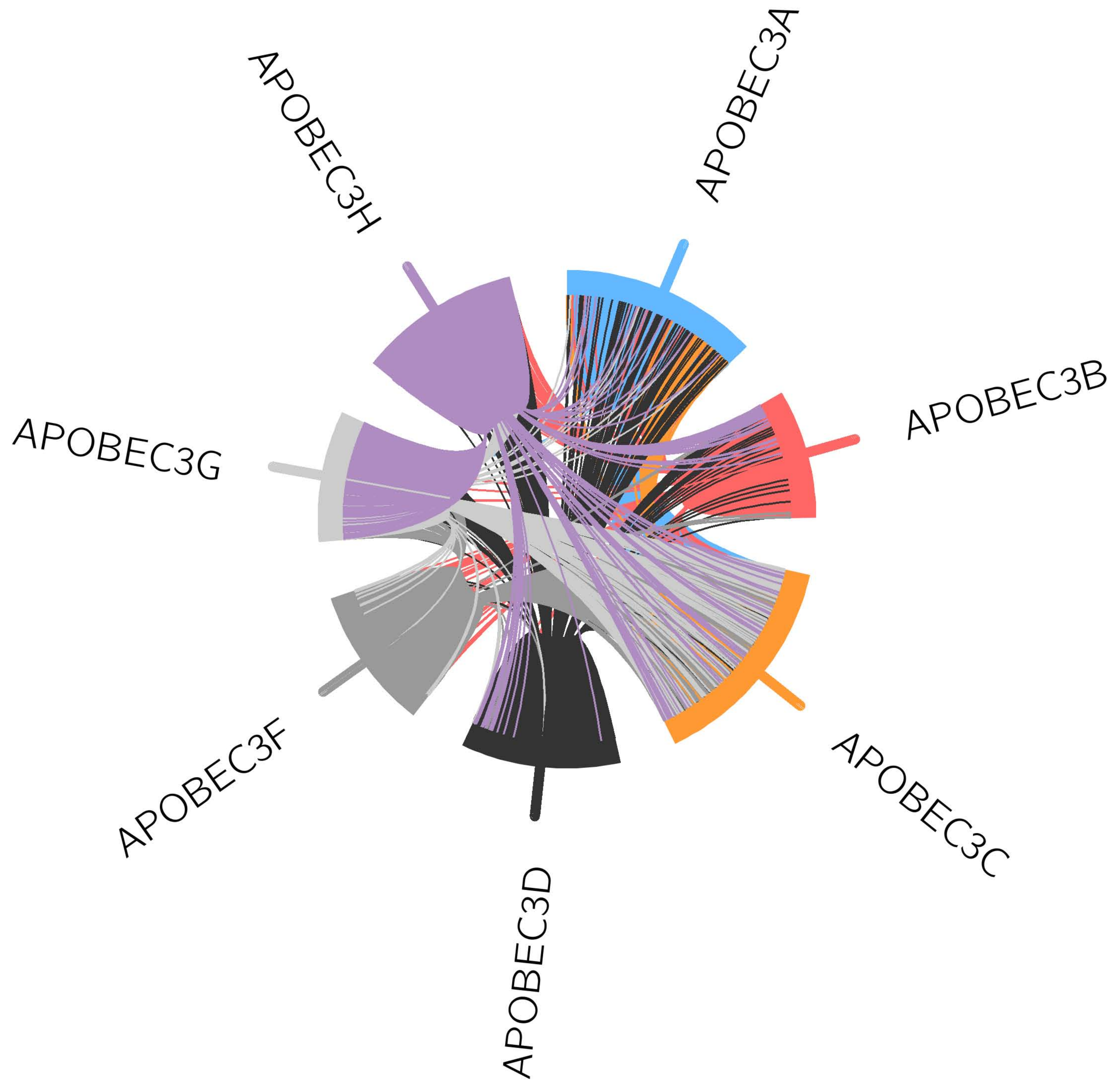

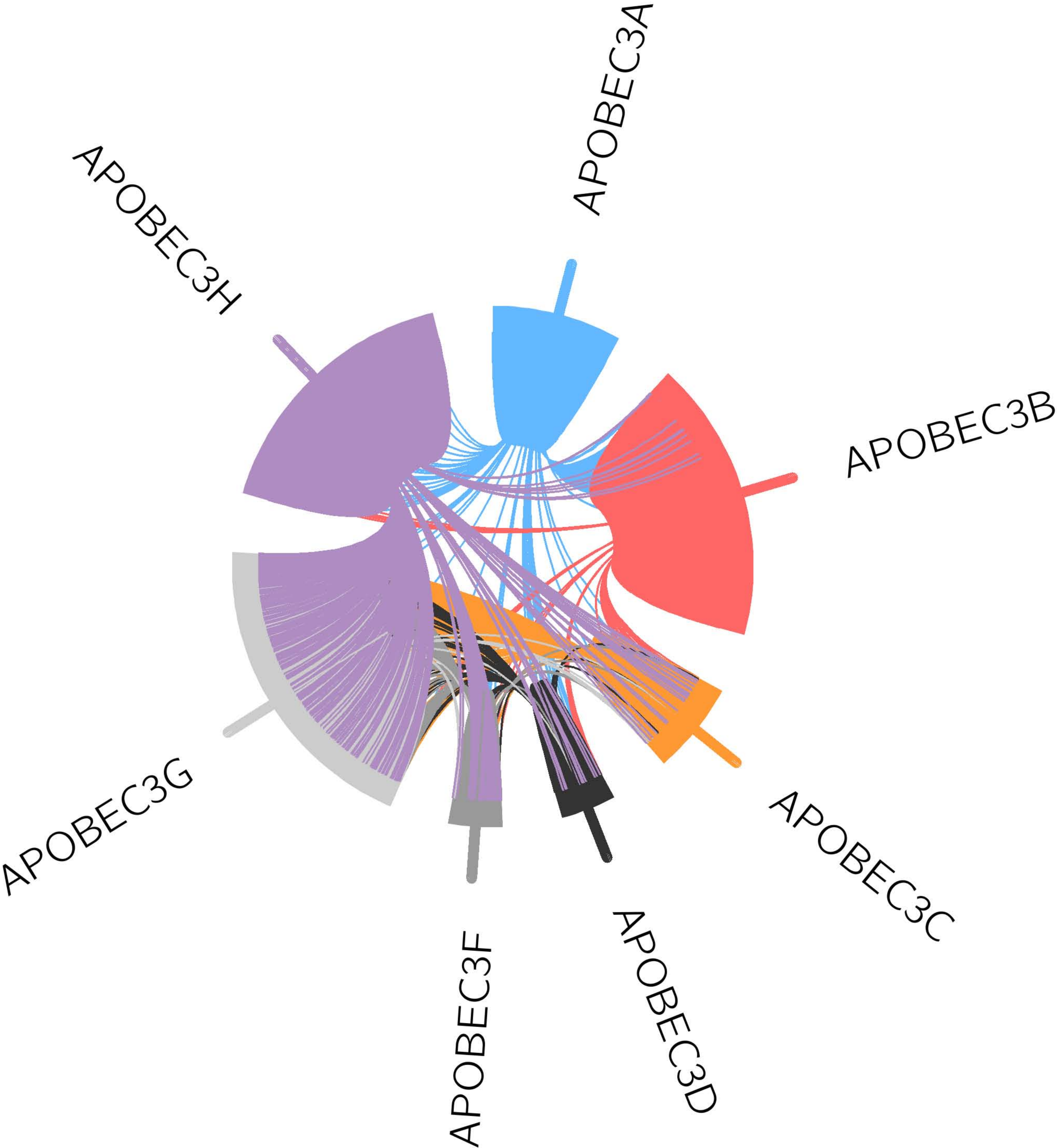

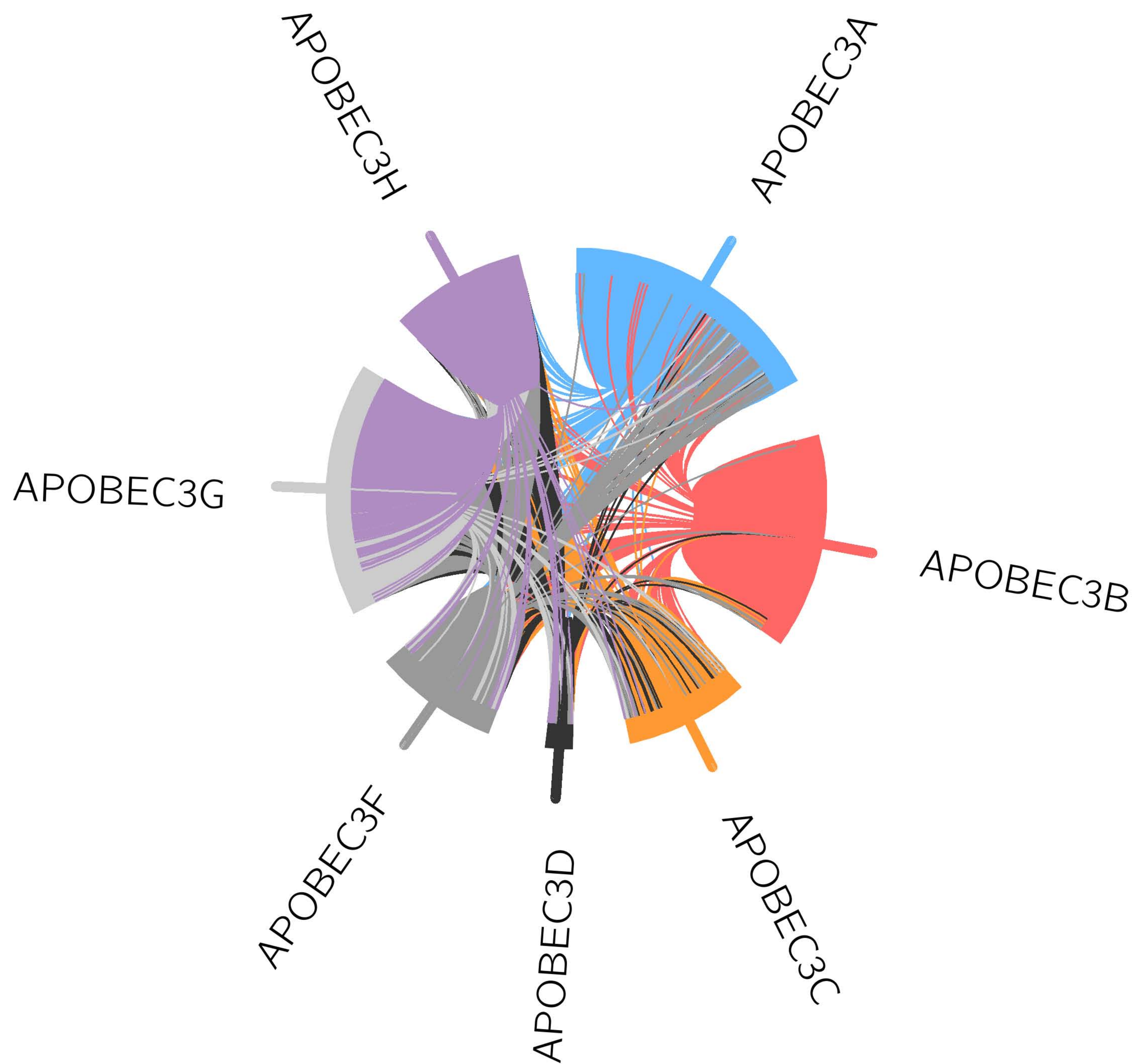

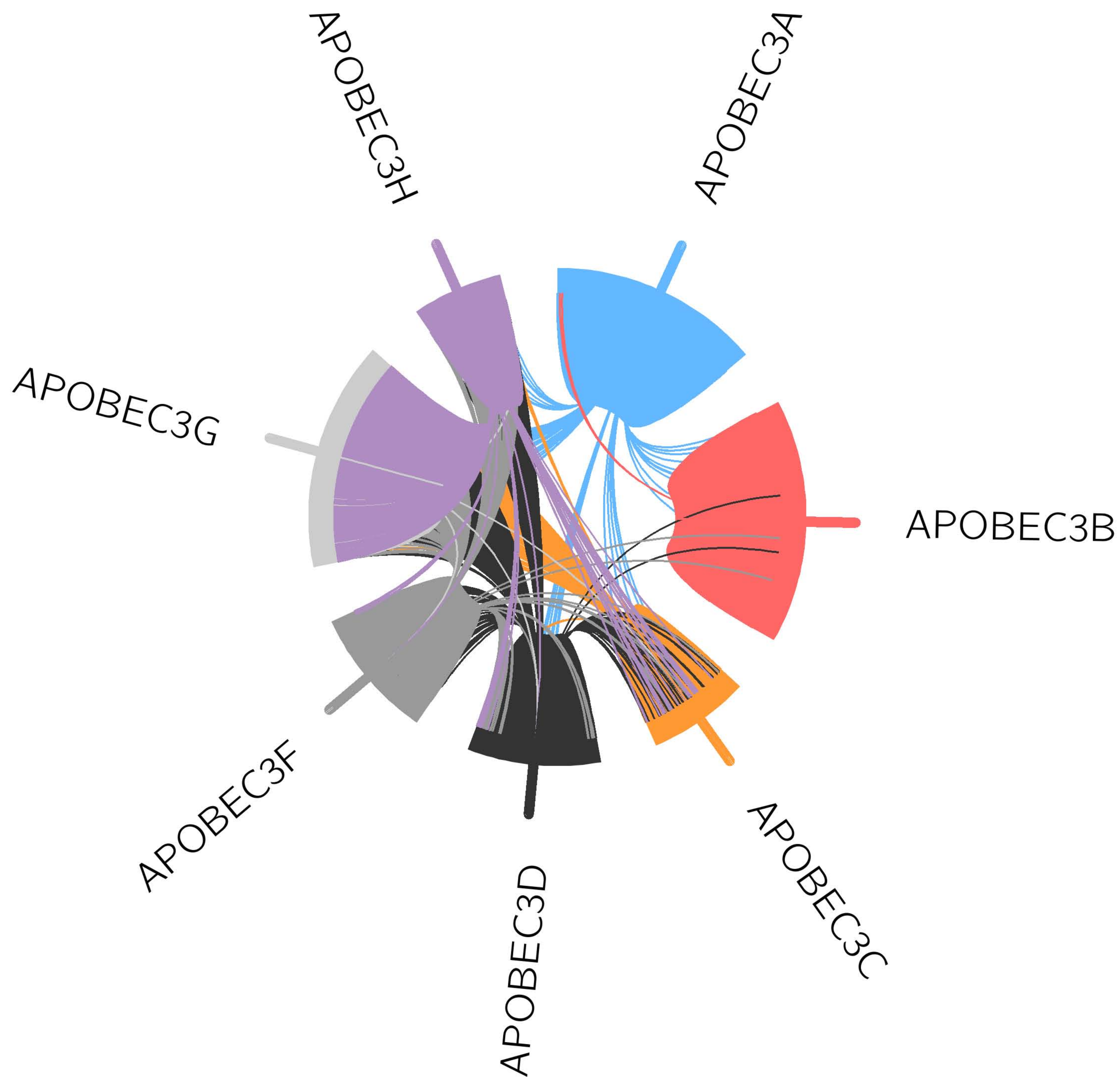

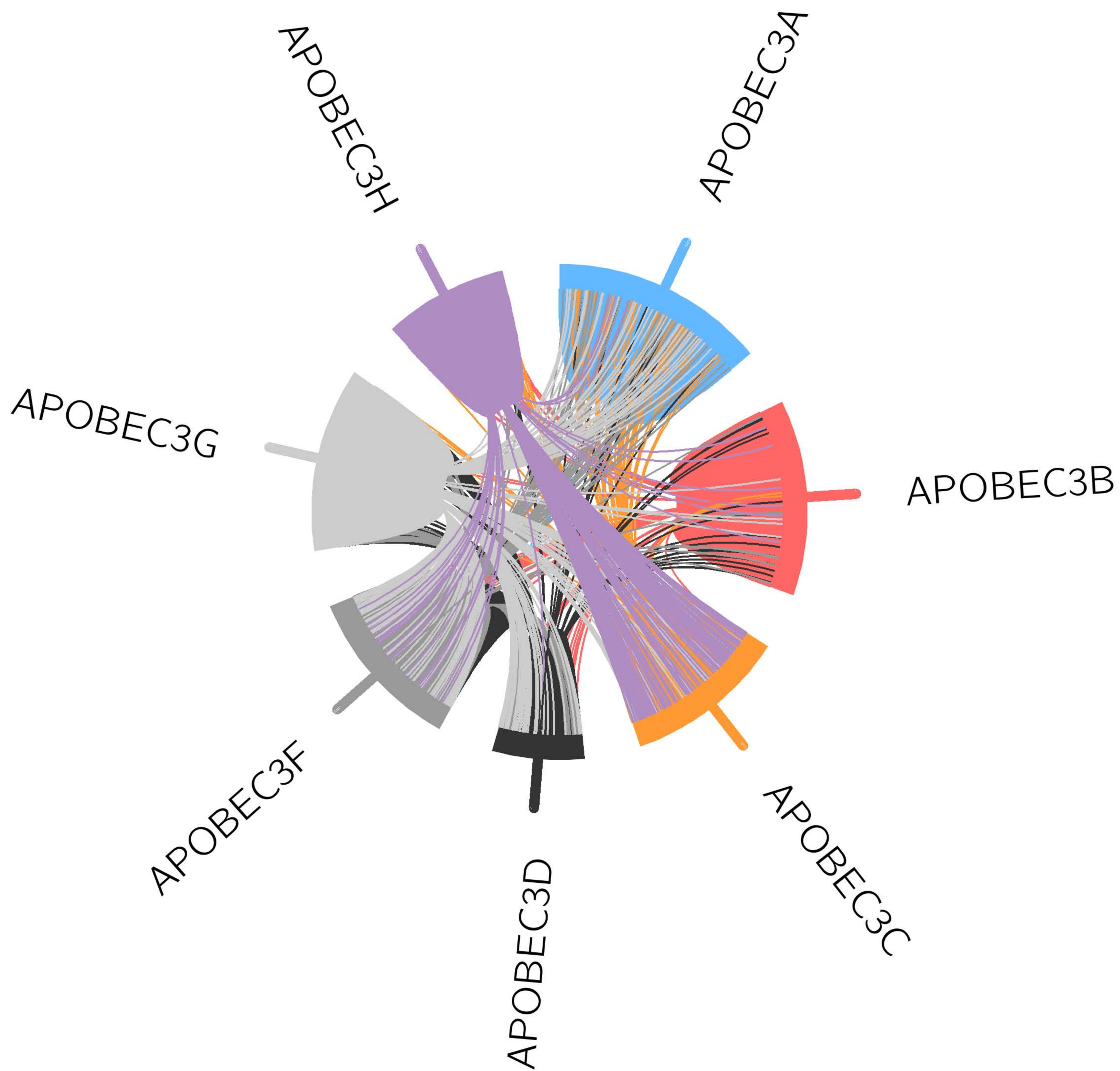

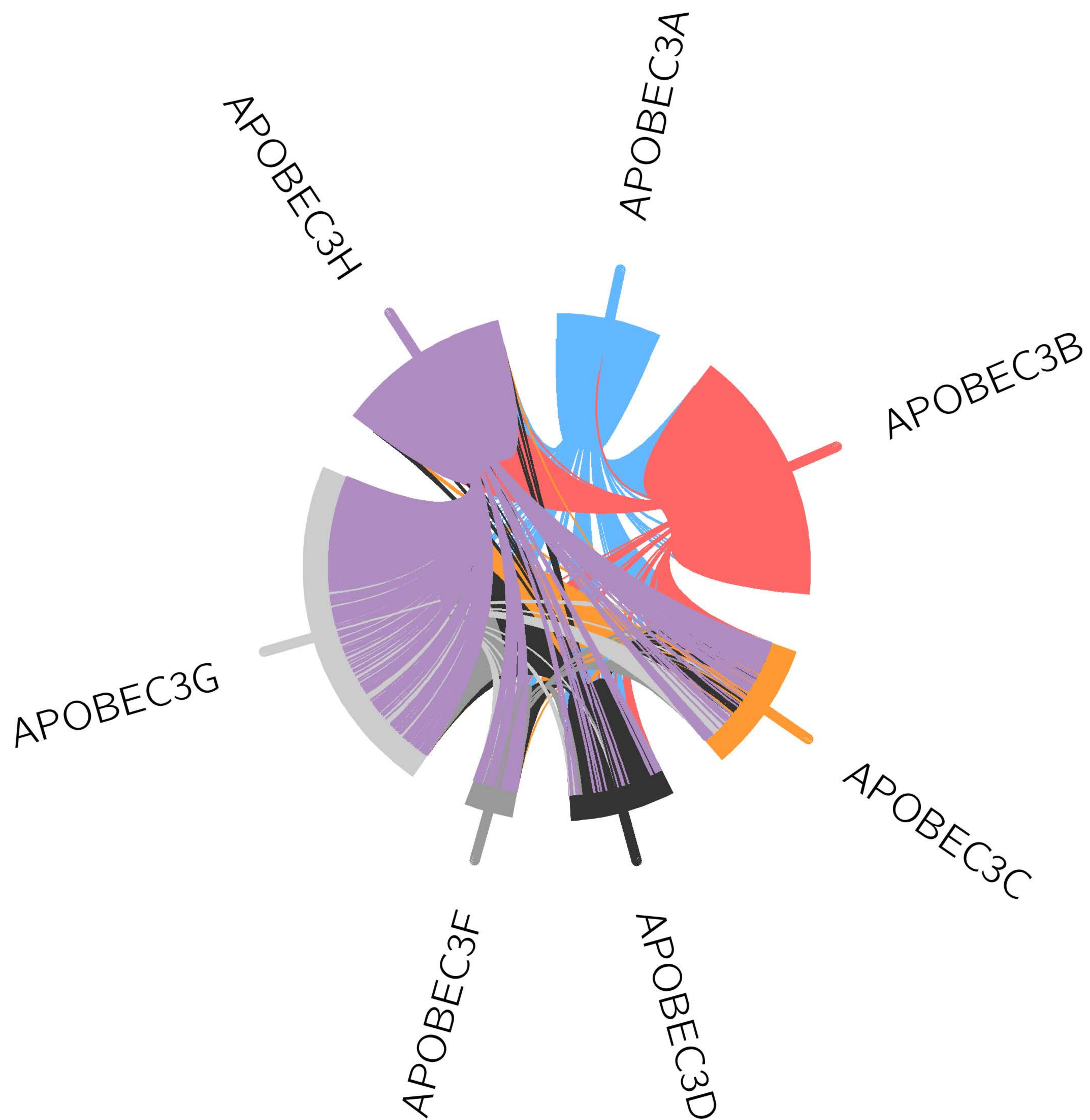

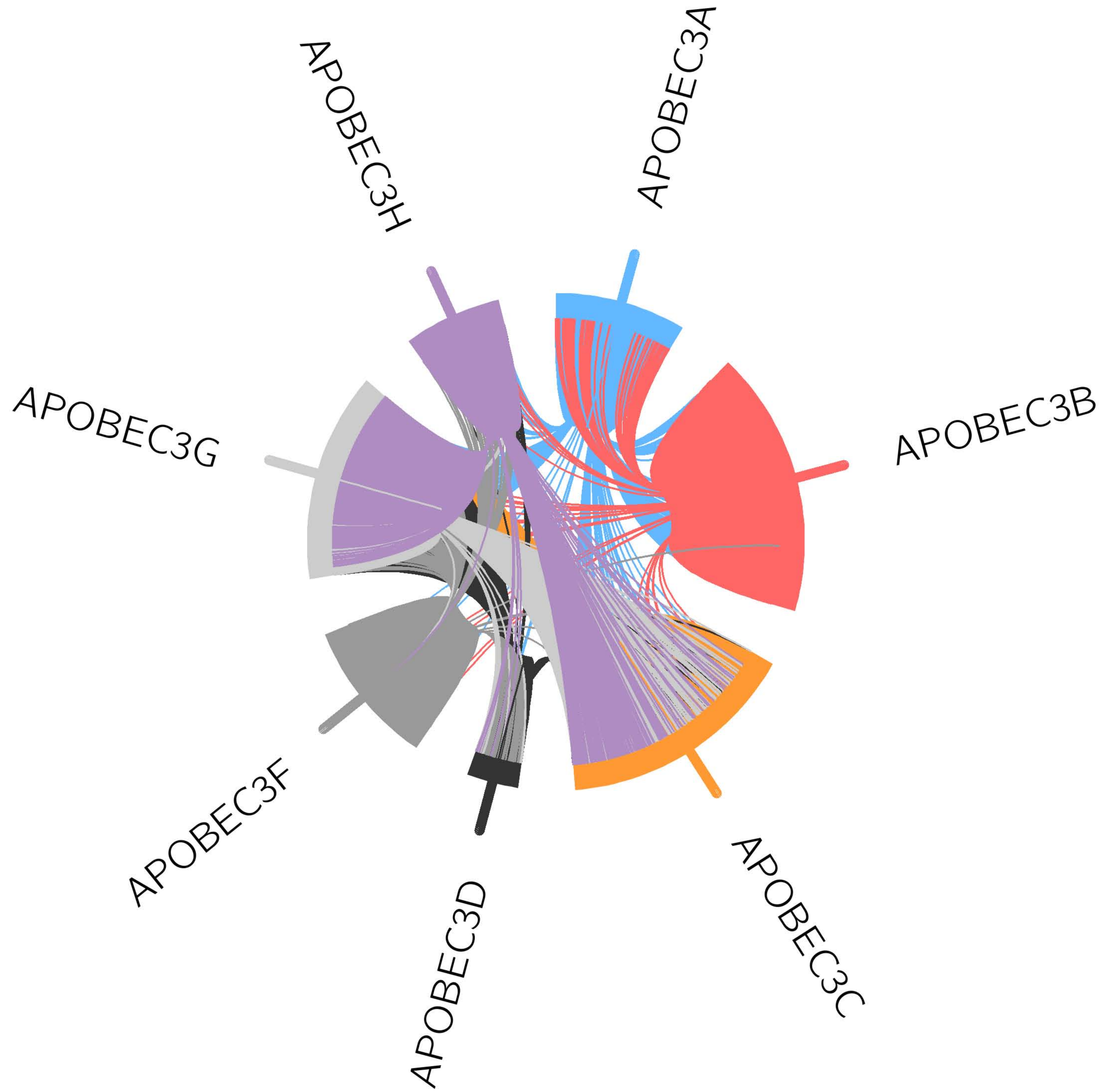

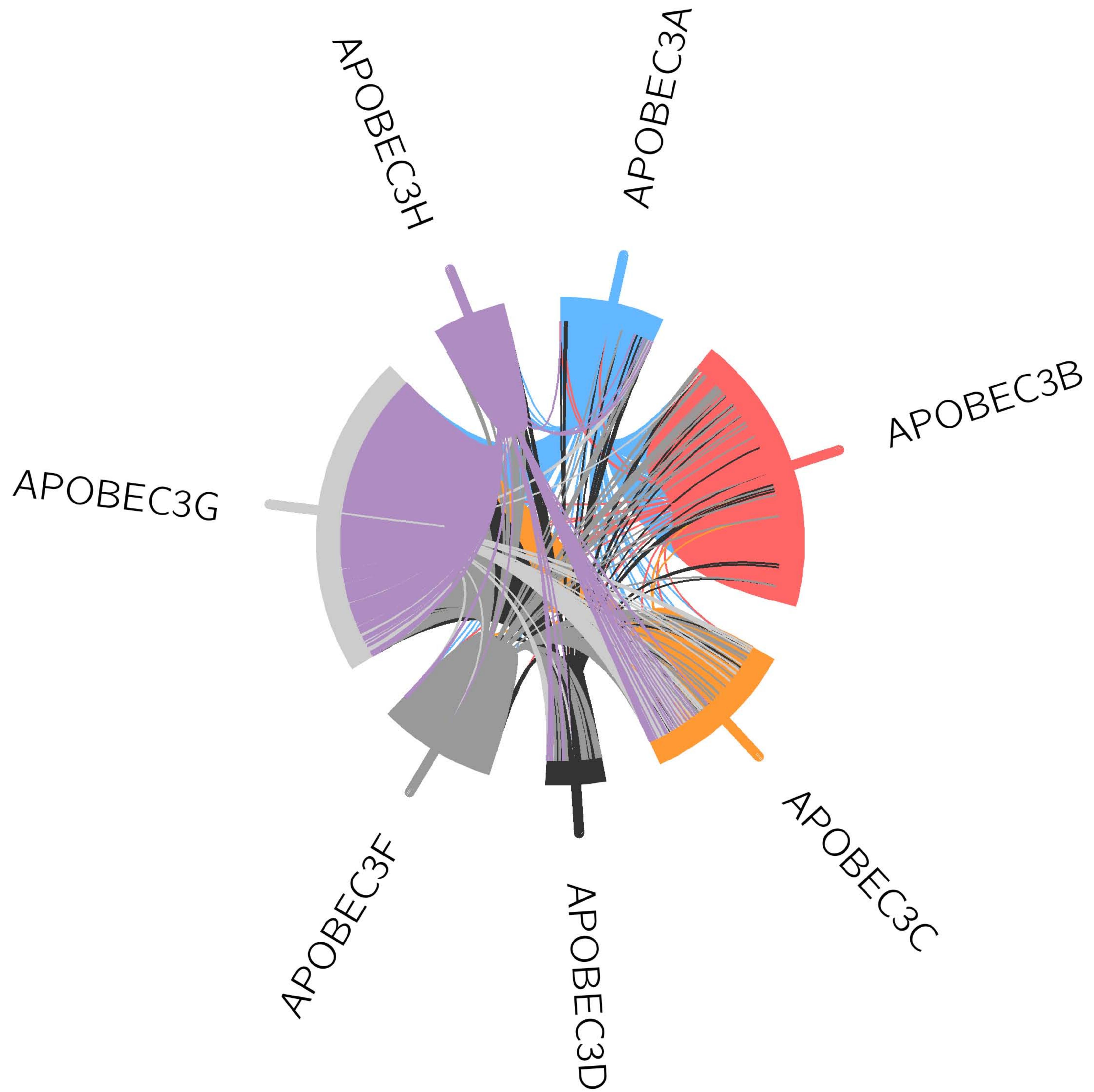

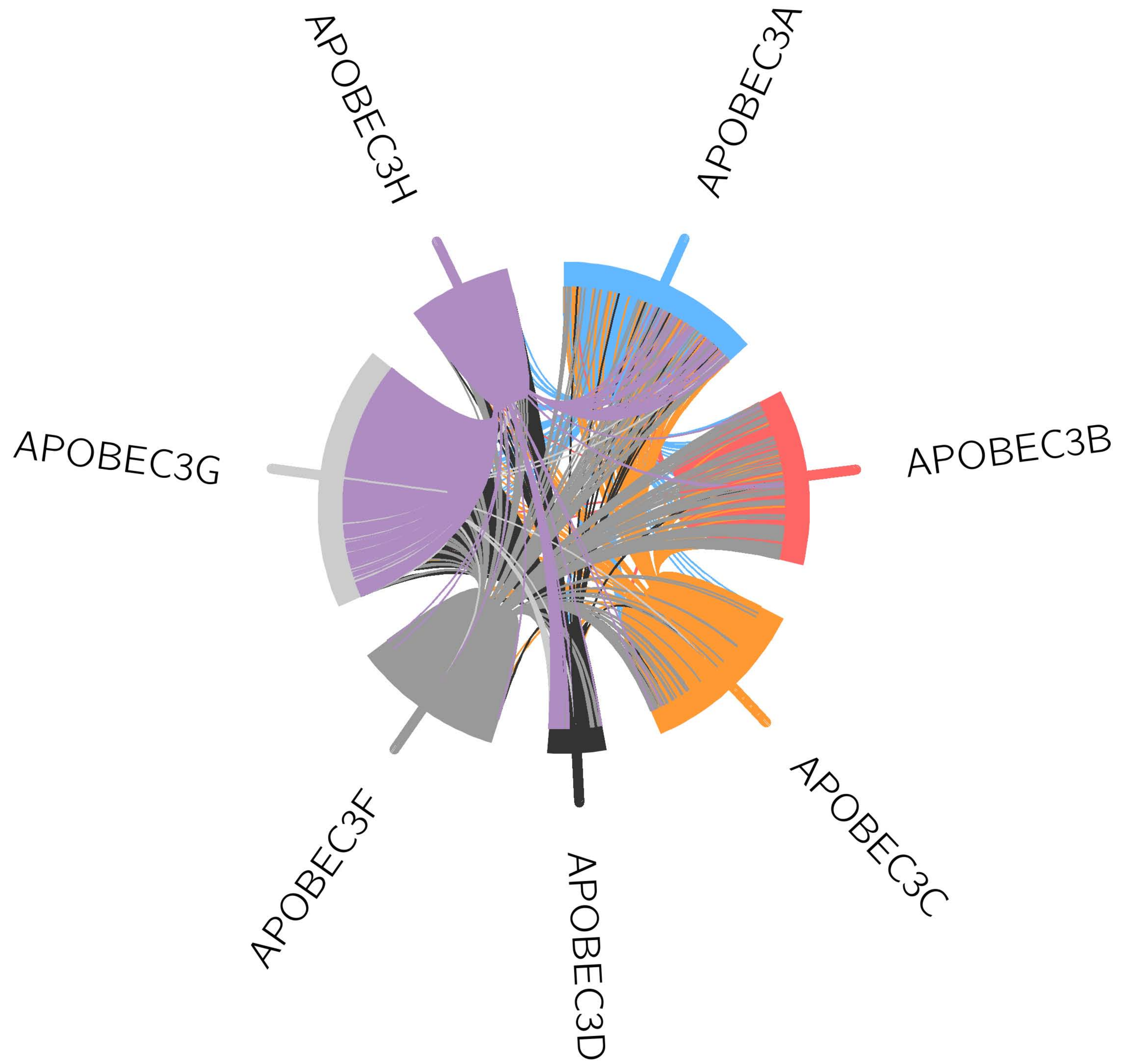

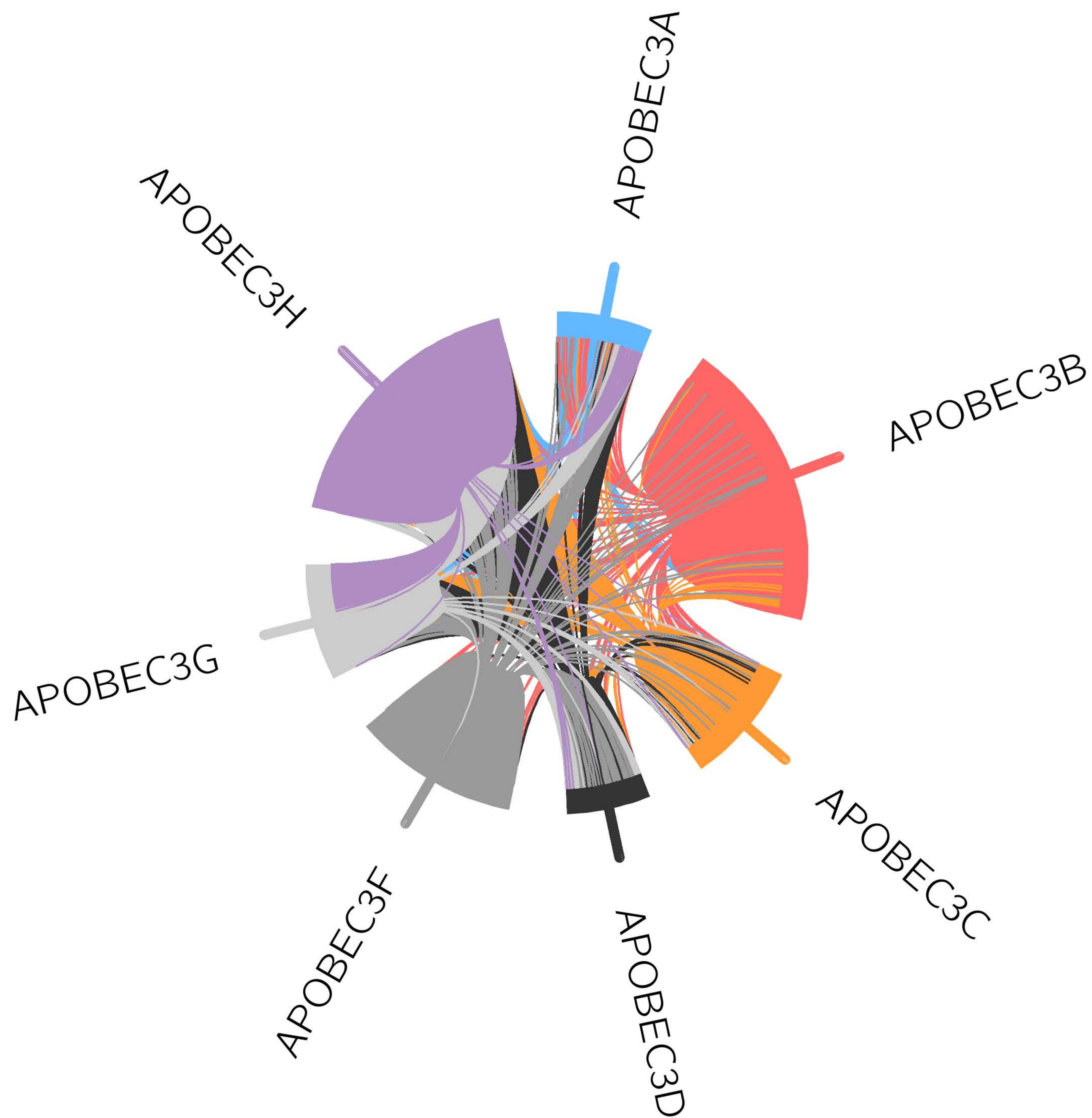

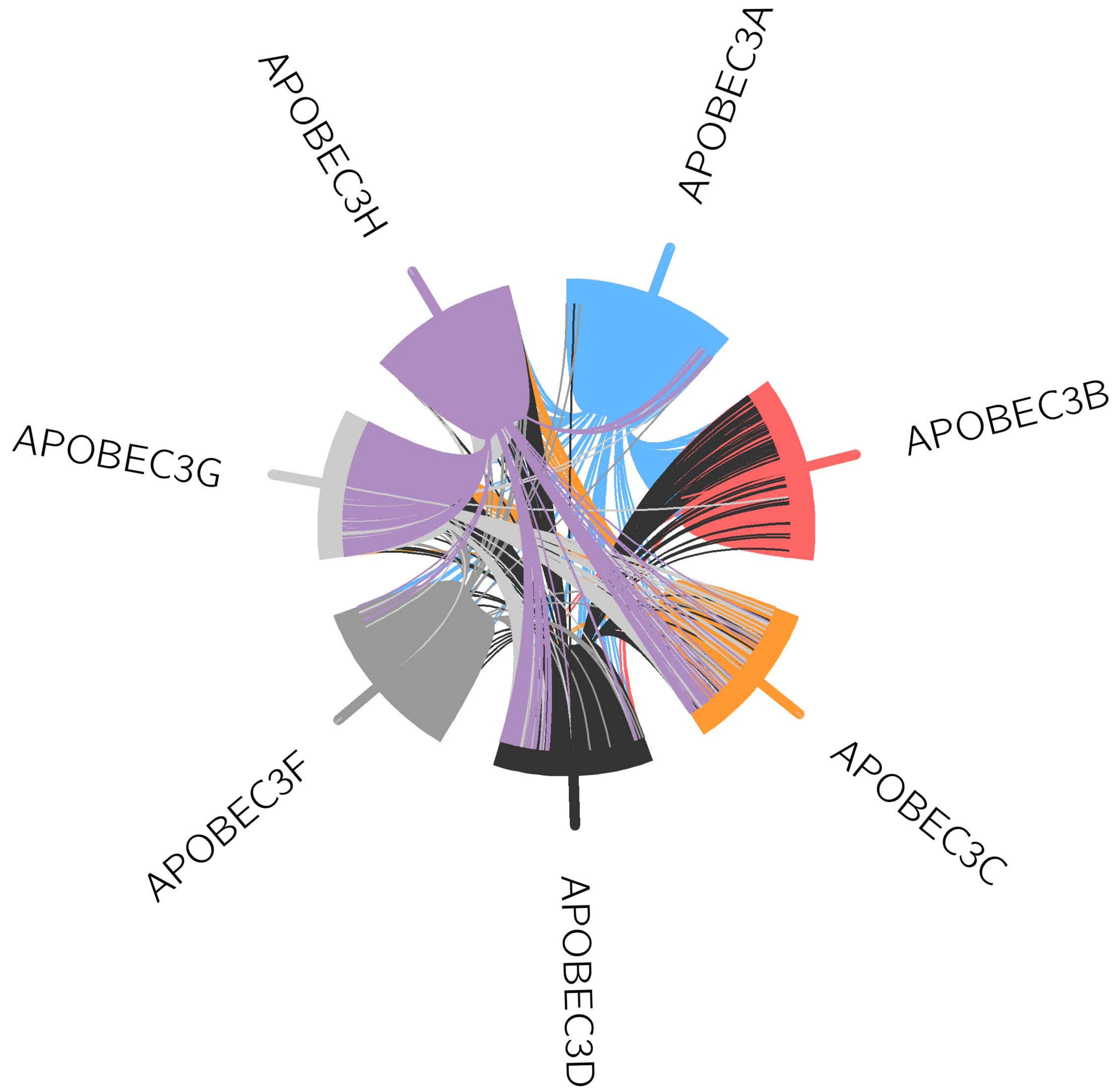

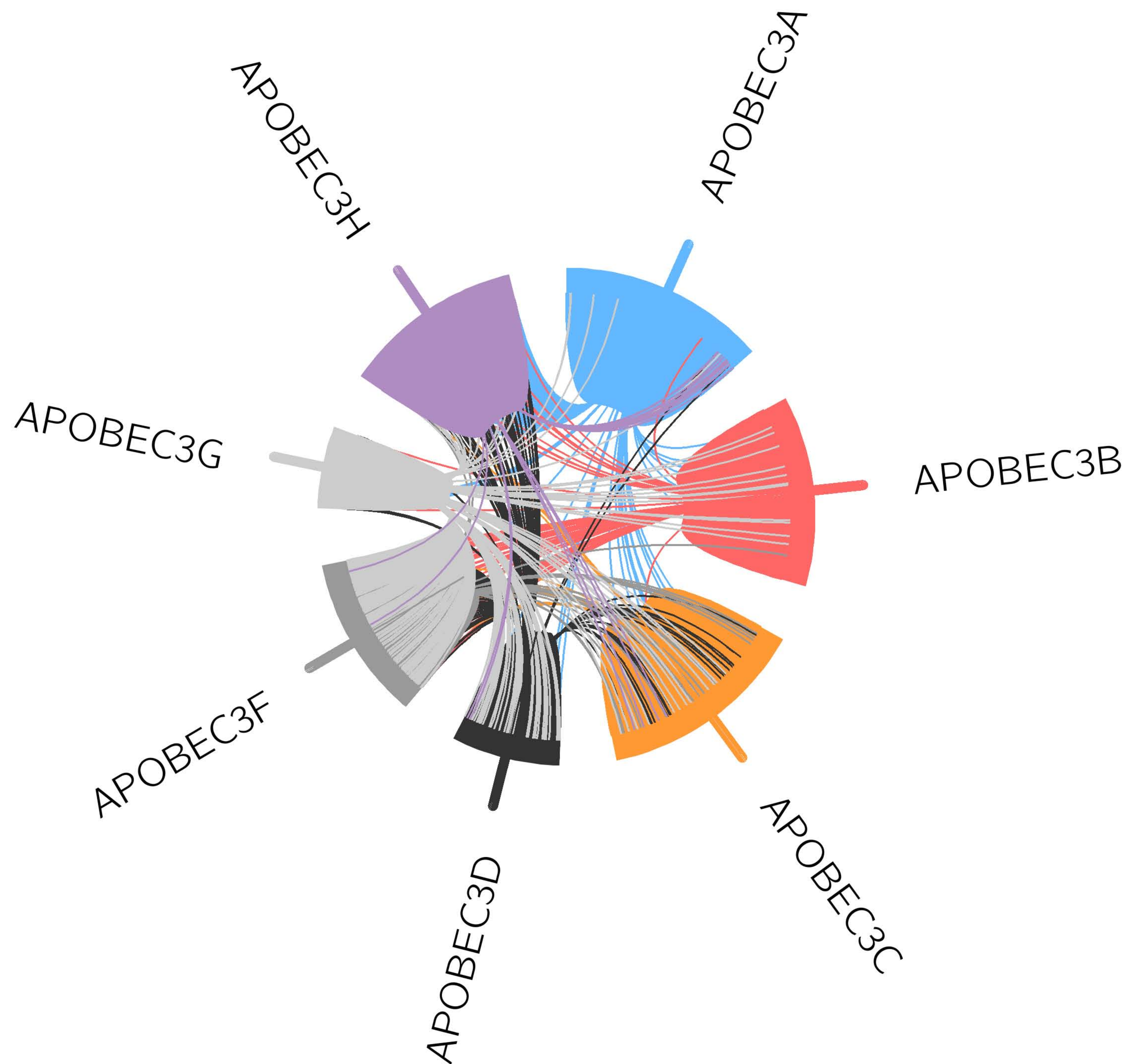

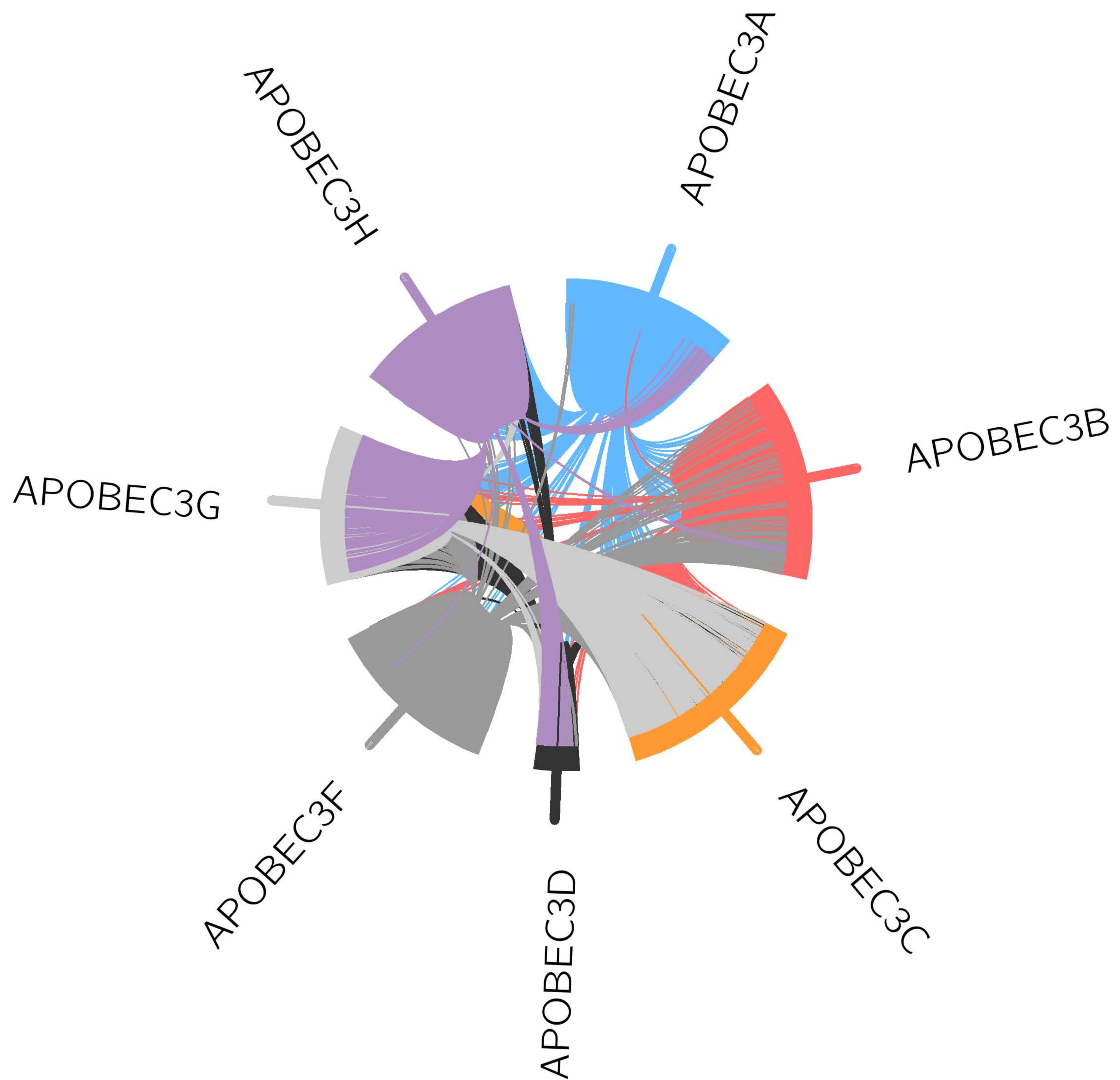

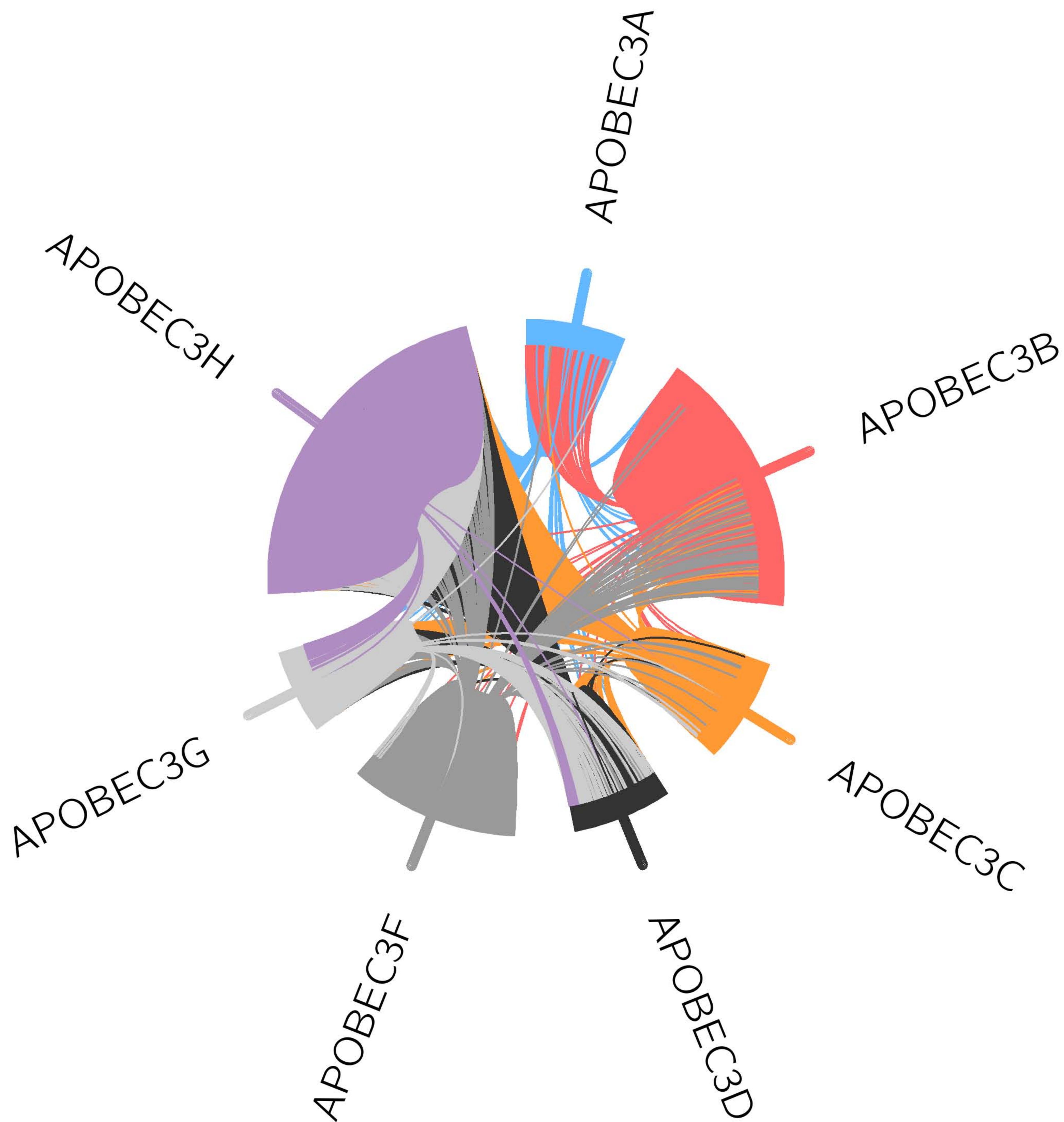

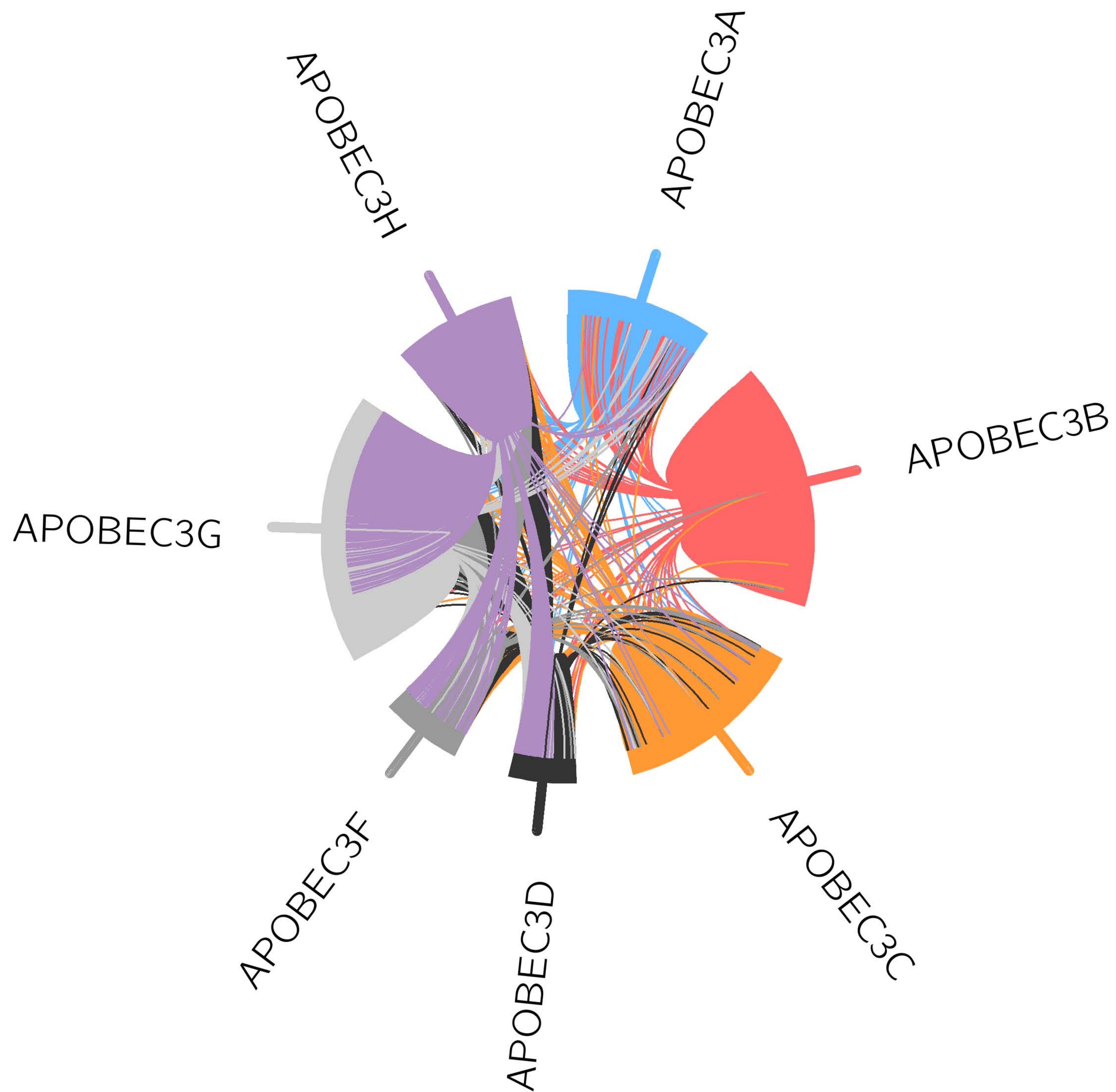

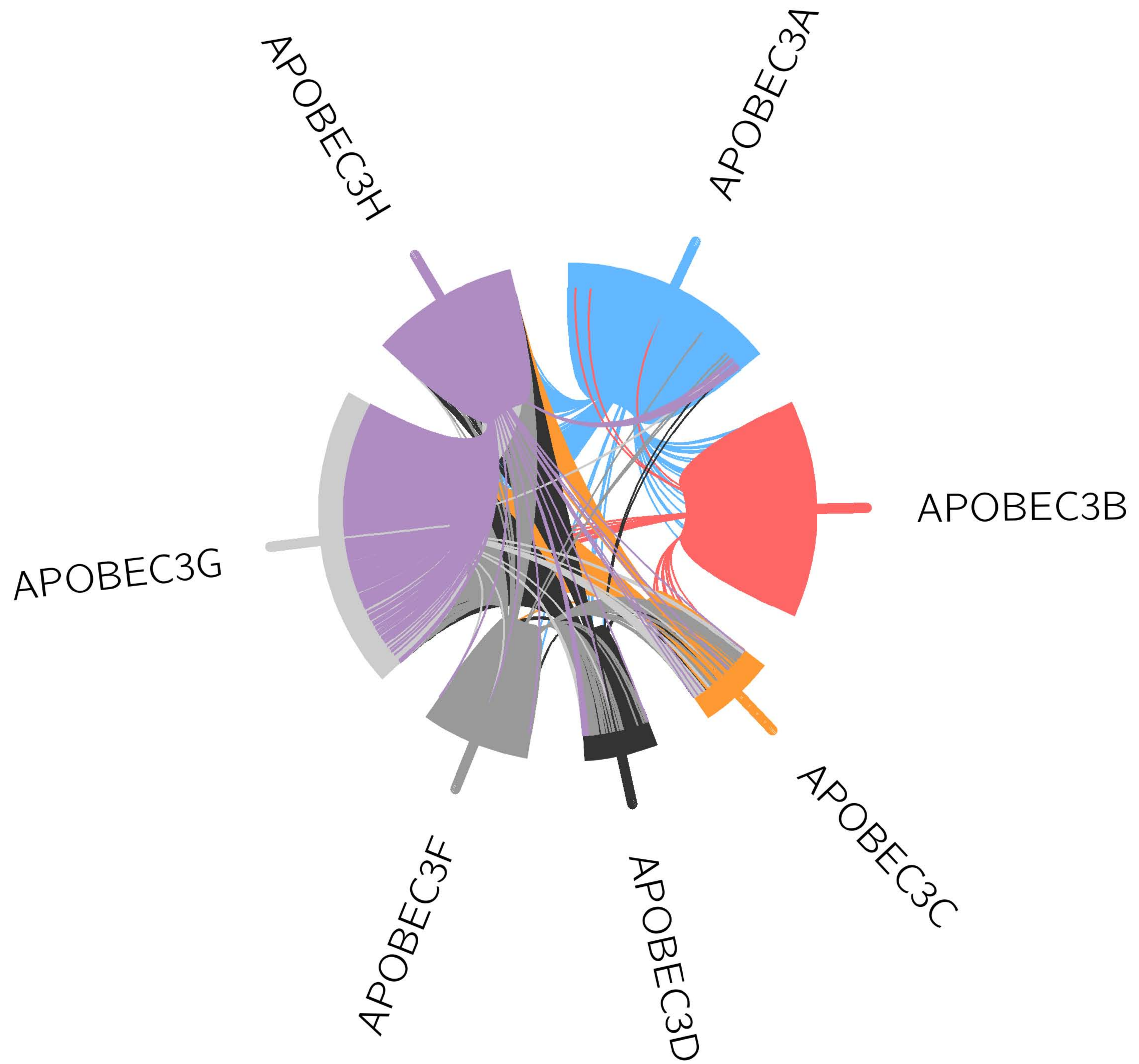

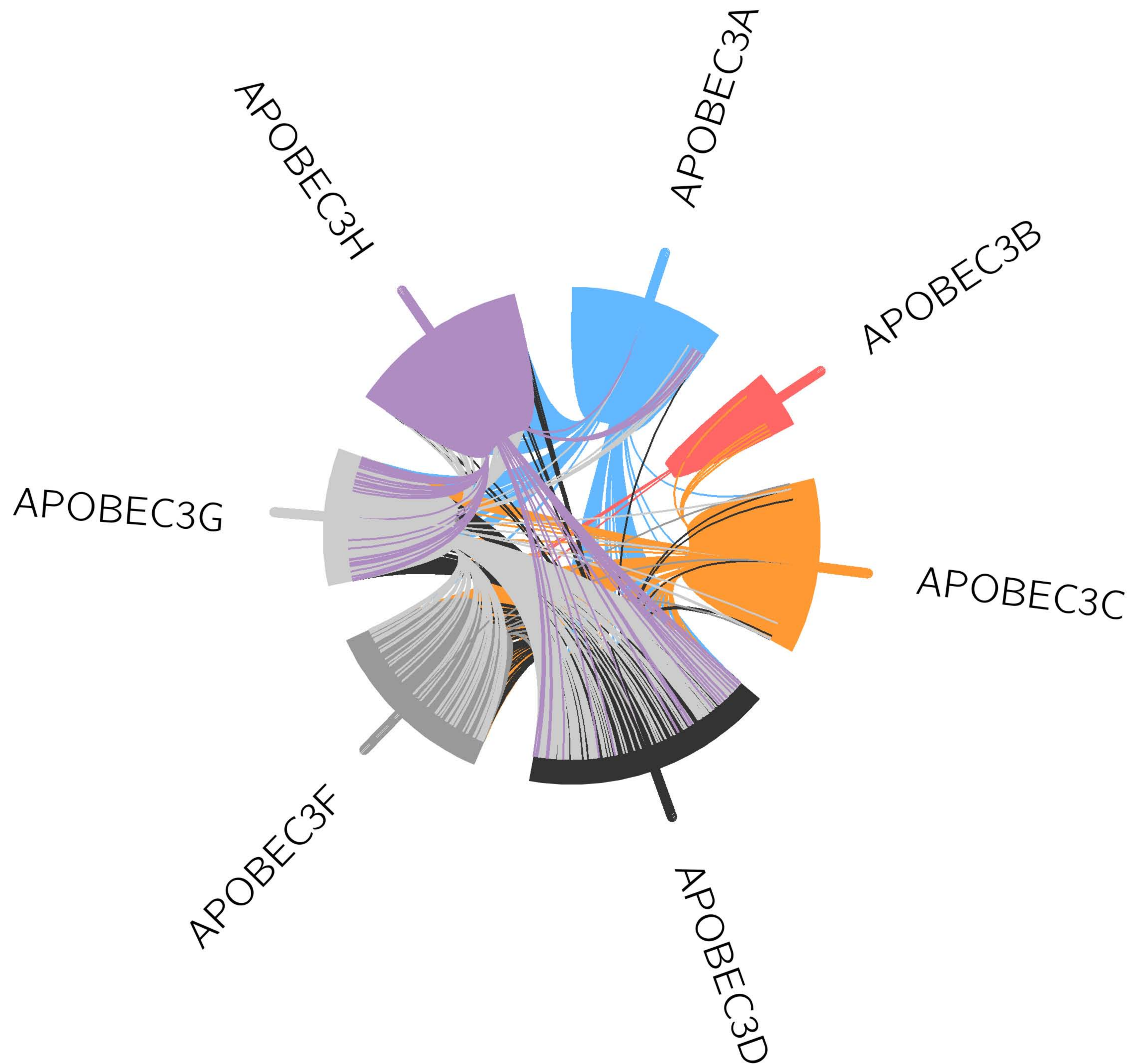

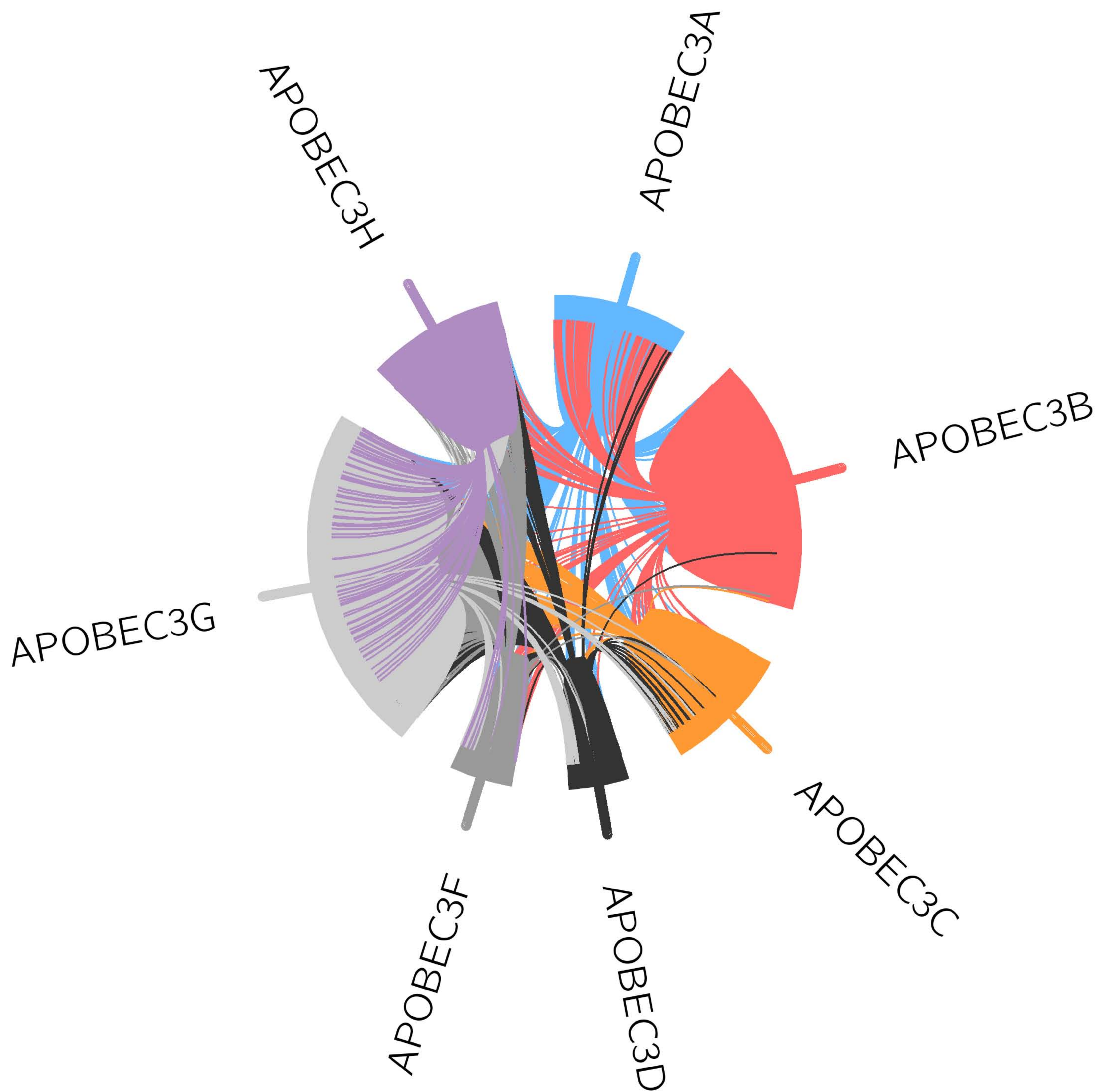

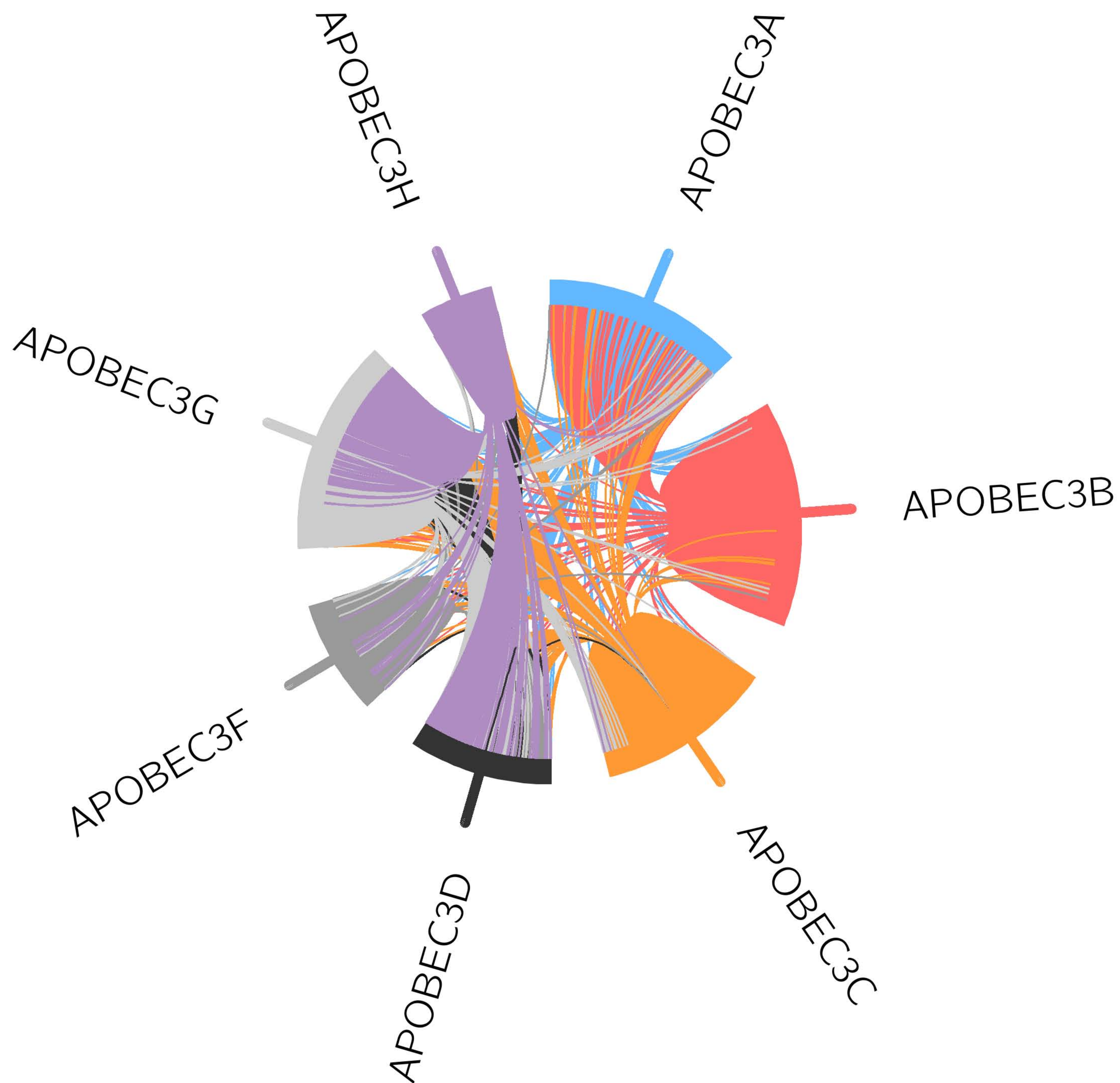

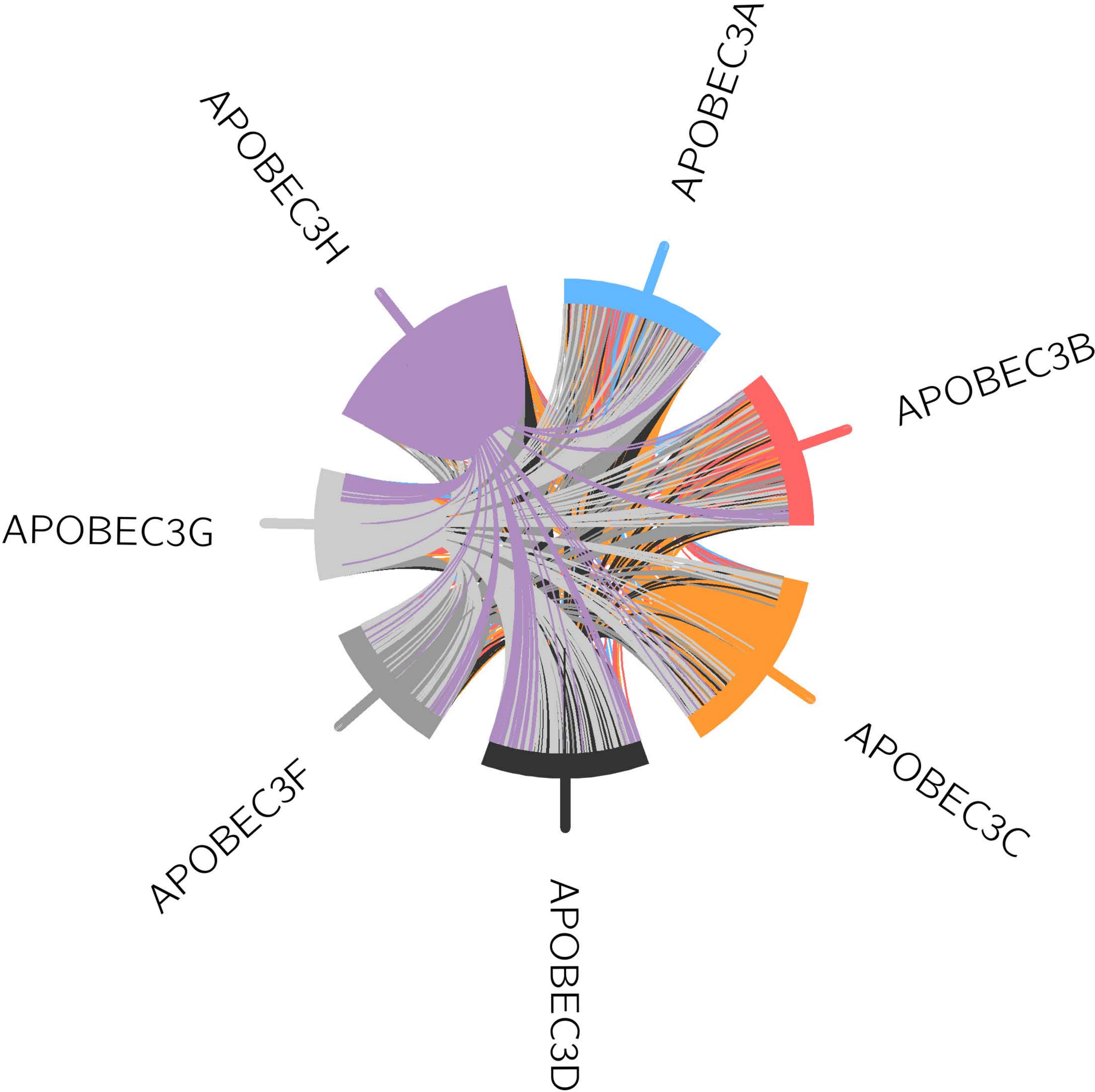

Supplement: Supplementary Data [file gky1316_supplemental_files.zip › FigureS14_CircosPlots_bs.pdf]
